# Design, Synthesis, Biological Evaluation, and Crystallographic Study of Novel Purine Nucleoside Phosphorylase Inhibitors

**DOI:** 10.1021/acs.jmedchem.2c02097

**Published:** 2023-05-03

**Authors:** Jan Skácel, Stefan Djukic, Ondřej Baszczyňski, Filip Kalčic, Tadeáš Bílek, Karel Chalupský, Jaroslav Kozák, Alexandra Dvořáková, Eva Tloušt’ová, Zuzana Král’ová, Markéta Šmídková, Jan Voldřich, Michaela Rumlová, Petr Pachl, Jiří Brynda, Tereza Vučková, Milan Fábry, Jan Snášel, Iva Pichová, Pavlína Řezáčová, Helena Mertlíková-Kaiserová, Zlatko Janeba

**Affiliations:** † Institute of Organic Chemistry and Biochemistry, The Czech Academy of Sciences, Flemingovo nám. 2, Prague 16610, Czech Republic; ‡ Institute of Molecular Genetics, The Czech Academy of Science, Vídeňská 1083, Prague 14220, Czech Republic; § University of Chemistry and Technology, Technická 5, Prague 16628, Czech Republic; ∥ Faculty of Science, Charles University in Prague, Hlavova 2030/8, Prague 2 12843, Czech Republic

## Abstract

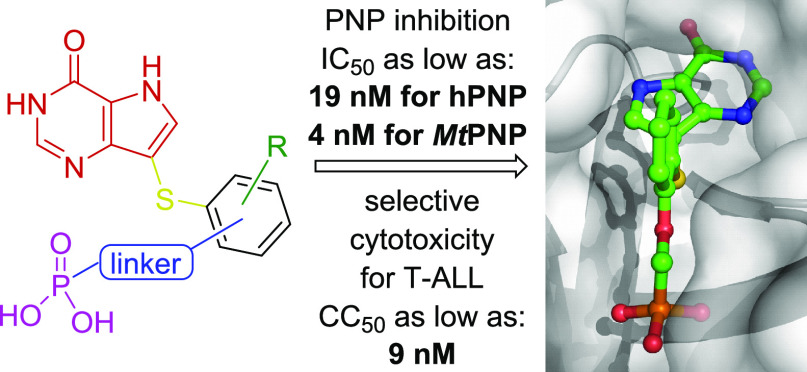

Purine nucleoside phosphorylase (PNP) is a well-known molecular target with potential therapeutic applications in the treatment of T-cell malignancies and/or bacterial/parasitic infections. Here, we report the design, development of synthetic methodology, and biological evaluation of a series of 30 novel PNP inhibitors based on acyclic nucleoside phosphonates bearing a 9-deazahypoxanthine nucleobase. The strongest inhibitors exhibited IC_50_ values as low as 19 nM (human PNP) and 4 nM (*Mycobacterium tuberculosis* (*Mt*) PNP) and highly selective cytotoxicity toward various T-lymphoblastic cell lines with CC_50_ values as low as 9 nM. No cytotoxic effect was observed on other cancer cell lines (HeLa S3, HL60, HepG2) or primary PBMCs for up to 10 μM. We report the first example of the PNP inhibitor exhibiting over 60-fold selectivity for the pathogenic enzyme (*Mt*PNP) over hPNP. The results are supported by a crystallographic study of eight enzyme-inhibitor complexes and by ADMET profiling *in vitro* and *in vivo*.

## Introduction

Purine nucleoside phosphorylases (PNPs, EC 2.4.2.1) are cytosolic enzymes involved in the purine salvage pathway metabolism in most organisms.^1,2^ The enzyme degrades (2′-deoxy)nucleosides based on 6-oxopurines to the corresponding purine bases and (2-deoxy)ribose-1-phosphate. Although PNP is a very old molecular target studied since 1960’s, there is no FDA- or EMA-approved therapy based on PNP inhibitors (PNPIs) available to date.^3^

Over the years, it has been shown that PNP inhibition leads to disruption of a nucleotide pool in cells, which affects viability of various pathogenic organisms (e.g., *Mycobacterium tuberculosis* and *Plasmodium falciparum*).^4−7^ However, some PNP knock-out experiments have suggested that PNP biological function in some pathogens might be replaced by other enzymes.^8^

Evidently, the most promising target for potential therapy based on PNP inhibitors is the human enzyme (hPNP).^9−11^ A rare autosomal-recessive metabolic disease caused by mutated and functionally deficient PNP is characterized by severe depletion of T-cells and, thus, by immunodeficiency. PNP deficiency results in an accumulation of its physiological substrate 2′-deoxyguanosine (dGuo), which is sequentially phosphorylated by deoxycytidine/deoxyguanosine kinase (dCK/dGK) and nucleotide kinases up to dGuo triphosphate (dGTP). Subsequently, dGTP induces apoptosis in developing T-cells in thymus through disrupted DNA synthesis by an allosteric inhibition of ribonucleoside-5′-diphosphate reductase.^12^

T-Cell-targeted antiproliferative properties of PNPIs open various therapeutic uses in the treatment of autoimmune diseases (e.g., inflammatory bowel disorders, multiple sclerosis, rheumatoid arthitis, psoriasis, and organ transplant rejection) and T-cell malignancies (leukemias and lymphomas).^9^ Recent research shed light on specific mutations responsible for sensitivity of cells to PNP inhibition.^13,14^ Such genetic markers potentially open other opportunities in more targeted therapies and even in a treatment of solid tumors. Moreover, it has been demonstrated that accumulation of guanosine (Guo) by PNPIs activates various toll-like receptors (TLRs), which, paradoxically, leads to immune activation effects. And so, utilization of PNPIs as immuno-oncology agents and as vaccine adjuvants has been suggested.^15^

It is beyond the scope of this article to cover more than 60 years of research in the field. Therefore, we only highlight some of the key milestones in the development of PNP inhibitors. Readers can examine a detailed review covering PNPIs’ development until 1998.^16^

**Figure 1:**
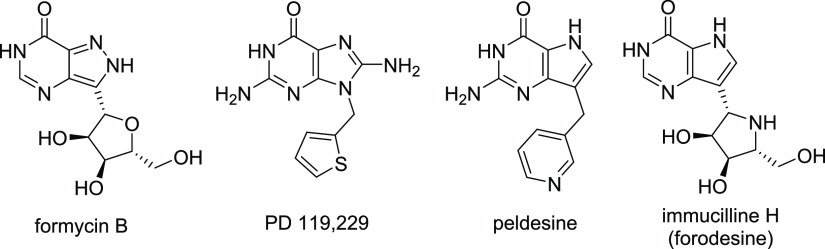
Example structures of published PNP inhibitors.

Structural and functional characterization of human PNP was first done by a pioneer work of Parks and co-workers in a series of papers published in 1968–1971.^3,17−19^ The group also described competitive inhibition of hPNP by a nucleoside analog formycin B (Figure 1).^20^ In the following years, other compounds based on nucleoside and nucleotide analogs were identified as inhibitors of hPNP.^11,21−27^

The first nonnucleoside PNPI, 8-amino-9-(2-thienylmethyl)guanine (PD 119,229, Figure 1), was developed in 1987 by Warner-Lambert Pharmaceutical Research.^28^ Although *in vivo* studies were also published, the compound has never entered clinical studies.^29^

In 1991, the first structure-based design of structurally novel PNP inhibitors was published by researchers from BioCryst Pharmaceuticals.^30^ Using a crystal structure of human erythocytic PNP, and inspired by the previous research, they identified a series of inhibitors with nanomolar IC_50_ values. The group highlighted 9-deazapurine as a strongly binding moiety and developed peldesine (BCX-34), the first PNP inhibitor that entered the clinical stage of development.^31,32^ Although the compound reached phase III clinical trials, it failed due to a lack of efficacy.^33^ Many other compounds based on peldesine’s structure with IC_50_ or K_i_ values in a low nanomolar range were published in the following years.^34−37^

In 1998, the hypothesis of transition-state inhibitors was used in the design of PNPIs by Schramm et al.^38^ The whole class of compounds, immucillins, proved to be very efficient inhibitors of PNP enzymes with subnanomolar *K_i_* values, and several generations of immucillins were published in the following years.^39^ Immucillin-H, also known as forodesine, entered several clinical trials directed at T-cell malignancies.^40−42^ Although forodesine was approved in Japan in 2017 for the treatment of refractory peripheral T-cell lymphoma, the clinical development of the compound has been discontinued in the USA and EU.^43^

In this work, we have designed novel PNP inhibitors based on phosphonates. We have developed an efficient synthetic methodology, which allowed us to combine structural features of several potent PNP inhibitors that were not accessible by previously published synthetic methods. The methodology also allowed us to significantly expand the structural space covered by previously published PNPIs. All prepared compounds were screened for inhibitory activity against recombinant PNP from three species (*Homo sapiens* (hPNP), *Mycobacterium tuberculosis* (*Mt*PNP), and *Plasmodium falciparum* (*Pf*PNP)) and were screened on three T-lymphoblastic cell lines (CCRF-CEM, MOLT-4, and Jurkat) and three non-T-cell cancer cell lines (HL60, HepG2, and HeLa S3). We provide herein an extensive crystallography study providing details on PNPI activity and selectivity, as well as on flexibility of both PNP enzymes and the inhibitors. Selected compounds were subjected to *in vitro* ADME profiling and an *in vivo* pharmacokinetic (PK) study.

## Results and Discussion

### Design

**Figure 2:**
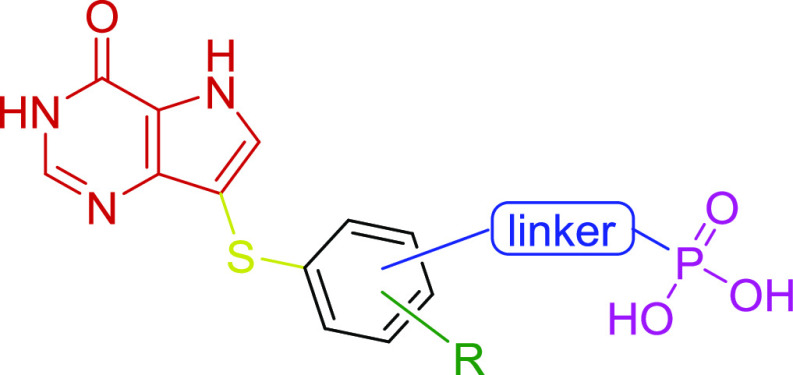
General structure of designed PNP inhibitors.

The design of our inhibitors (Figure 2) is built on the knowledge of the structural features of previously reported inhibitors. The development of peldesine led to an identification of 9-deazapurines as moieties very strongly binging to hPNP.^30,36,37^ Peldesine (Figure 1) consists of 9-deazaguanine and benzyl-like moiety attached to the “purine” base at position 9 via a methylene linker. It has also been reported that a sulfur atom instead of the methylene linker significantly increased the potency of peldesine-based inhibitors.^34^

Others described inhibitors based on natural purine bases and either acyclic, cyclic, or benzylic moieties bearing a phosphonate group.^22−24^ Such inhibitors were reported to be as potent as the inhibitors bearing 9-deazapurine moieties.

One would ask why there are no examples of inhibitors combining all these structural features in one molecule. Well, the synthesis of peldesine-like structures was based on the sequential construction of the 9-deazapurine base. Conditions used in those transformations were generally incompatible with sensitive and/or reactive functional groups like phosphonic esters. On the other hand, the inhibitors with phosphonate groups were synthesized by simple and mild alkylation of the corresponding purine nucleobases at position N9.

In our concept (Figure 2), we selected 9-deazahypoxanthine (nucleobase from forodesine) instead of 9-deazaguanine, since the extra amino group was expected to significantly increase the polarity of the moiety (potentially impairing PK properties of designed compounds) and to add an extra nucleophilic center (complicating the overall synthesis). The phenyl moiety is attached to 9-deazahypoxanthine at position C9 via a sulfur atom and is connected to the phosphonate group via a variable linker (different length, structure, and position on the phenyl ring). The phenyl moiety can be further substituted to additionally modify the properties of the PNP inhibitors and also to explore the space of the active site of PNPs.

### Chemistry

**Scheme 1:**
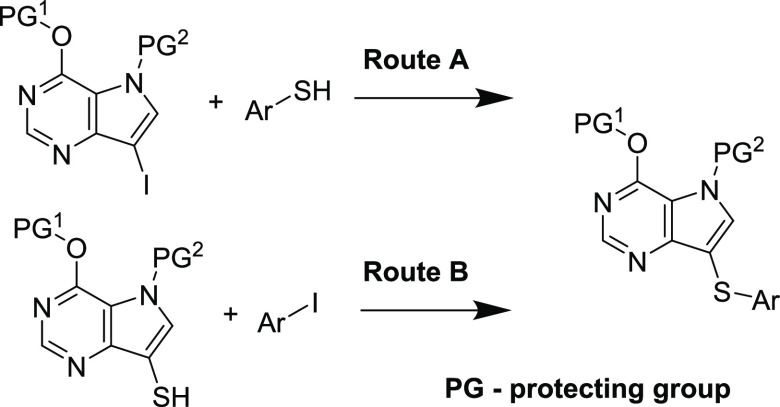
Two Strategies Show How to Perform the Key Transformation Using the Ullmann Coupling

We have designed the synthesis of our target compounds using the Ullmann coupling between aryl iodides and aryl thiols as the key reaction step (Scheme 1). Such a convergent approach would allow the screening of a larger and more diverse series of potential PNP inhibitors. Both reaction partners, i.e., the thiol and iodine groups, can be attached either to the purine or the phenyl moiety. We have successfully employed both strategies to obtain target compounds since each of them proved to be efficient in different synthetic situations.

**Scheme 2:**
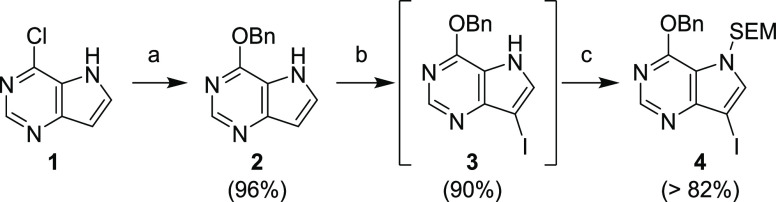
Optimized Multigram Synthesis of Compound **4** Reagents and conditions: (a) BnOH, Na, rt, 5 h, SiO_2_ filtration, crystallization; (b) NIS, THF, rt, 5 min, crystallization; (c) SEM-Cl, NaH, DMF, rt, 15 min, crystallization.

The key strategic intermediate for the synthetic **Route A** (Scheme 1) was compound **4** (Scheme 2), which was prepared by our group within a previous project.^44^ Although the originally published procedure afforded compound **4** in a high overall yield (over 70%, three steps from chloro derivative **1**), several drawbacks were identified in the sequence preventing a simple upscale of the synthesis. For instance, steps with the most expensive reagents were placed at the beginning of the synthetic sequence, and the most problematic step with the cheapest transformation was placed at the end of the sequence. Furthermore, several column chromatography runs were used with reverse-phase chromatography as the final purification step. Hence, such an approach was clearly not suitable for the preparation of desired intermediate **4** in a multigram amount necessary for the synthesis of a large series of PNP inhibitors as well as for planned *in vivo* experiments with the most promising candidates.

We optimized the procedure as presented in Scheme 2. Nucleophilic substitution of the chlorine atom in compound **1** with benzyl alcohol, to give compound **2**, was the first step of the synthesis, followed by iodination with NIS (compound **3**) and alkylation with 2-(trimethylsilyl)ethoxymethyl chloride (SEM-Cl) to obtain compound **4**. Only one silica gel filtration was required, and intermediate **2** was isolated by crystallization. The overall yield (3 steps) was over 70%, and the synthesis was performed on a 100 g scale.

**Scheme 3:**
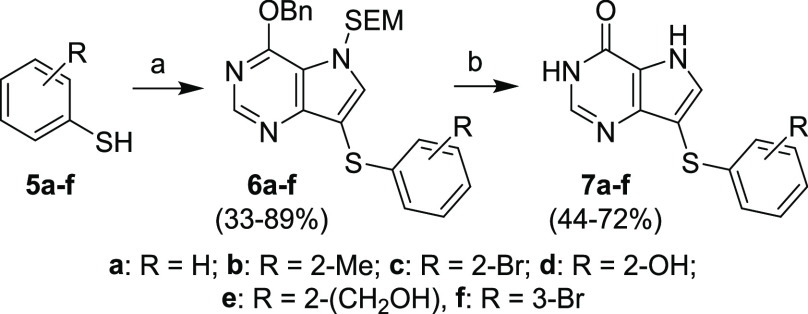
Synthesis of Compounds **7a–f** via Ullmann Coupling of Compound **4** with Substituted Thiophenols Reagents and conditions: (a) compound **4**, CuI, 1,10-phenanthroline, Et_3_N, toluene, MW, 120 °C, 1–2 h; (b) (1) TFA, rt, 15 min; (2) NH_3_/EtOH, rt, 1 min.

Ullmann coupling of compound **4** with a range of commercially available thiophenols **5a**–**f** provided desired products **6a–f** in good-to-high yields (33–89%) when performed in toluene in the presence of copper iodide as a catalyst, triethylamine as a base, and 1,10-phenanthroline as a ligand (Scheme 3). The coupling can be heated conventionally or in a microwave reactor, which reduces reaction times. The Ullmann coupling tolerated *ortho*-substituted thiophenols, polar functional groups, and halogens other than iodine. It was also possible to use various bases (e.g., Et_3_N, DIPEA, or Cs_2_CO_3_) and various solvents (e.g., toluene or DMF), and the method proved to be very robust and reproducible (optimizations not showed).

The coupling intermediates **6a–f** were deprotected to final compounds **7a–f** (Scheme 3) under acidic conditions with trifluoroacetic acid, followed by basification with an ammonia solution in ethanol. The basification was necessary to cleave the remaining *N*7-hydroxymethyl residue on the purine moiety, which was not fully cleaved during the acidic cleavage of the SEM protecting group. Lower yields of products **7a–f** (44–72%) were caused by purification difficulties due to the low solubility of the target compounds in any solvents except for DMF and DMSO. Thus, it was demonstrated that this kind of structure (without the phosphonate moiety) exhibited severe solubility issues not only in most organic solvents but in water as well, suggesting insurmountable barriers for potential pharmaceutical use.

**Scheme 4:**
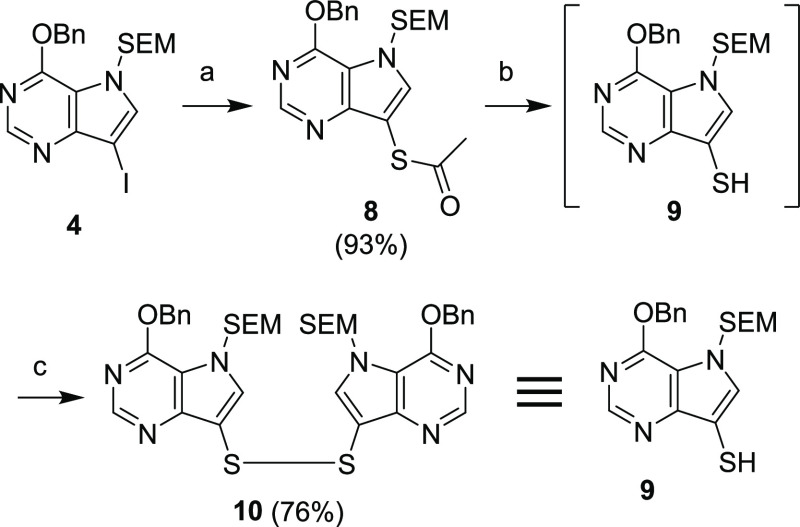
Synthesis of Compound **9** Reagents and conditions: (a) CH_3_COSK, CuI, 1,10-phenanthroline, Et_3_N, toluene, 125 °C, 4 h; (b) MeOH, K_2_CO_3_, 50 °C, 1 h; (c) air, MeOH, rt, 1 h.

Although the above-mentioned conditions coupled iodo derivative **4** with thiophenols **5a–f** efficiently (Scheme 3), synthesis of thiophenols bearing phosphonate moieties turned out to be very challenging (data not shown). In general, such thiophenols rapidly oxidized to very stable disulfides, which did not undergo Ullmann coupling. Therefore, we employed the second **Route B** (Scheme 1), in which aryl iodides bearing the phosphonate moiety are coupled with the protected 9-deazapurine scaffold with a thiol group at position C9. Such protected 9-mercapto-9-deazapurine derivative **9** (Scheme 4) was synthesized from intermediate **4** via Ullmann coupling with potassium thioacetate to give compound **8**, followed by subsequent methanolysis of the acetyl group. Interestingly, obtained compound **9** rapidly oxidized on air to the corresponding disulfide **10** (Scheme 4).

Fortunately, it was discovered that disulfide **10** is quite a labile compound and it reacted in all studied cases in the same way as the corresponding thiol **9**, without any need for prior reduction. Therefore, compound **10** was used as a direct source of compound **9** to simplify the description of the experiments.

**Scheme 5:**
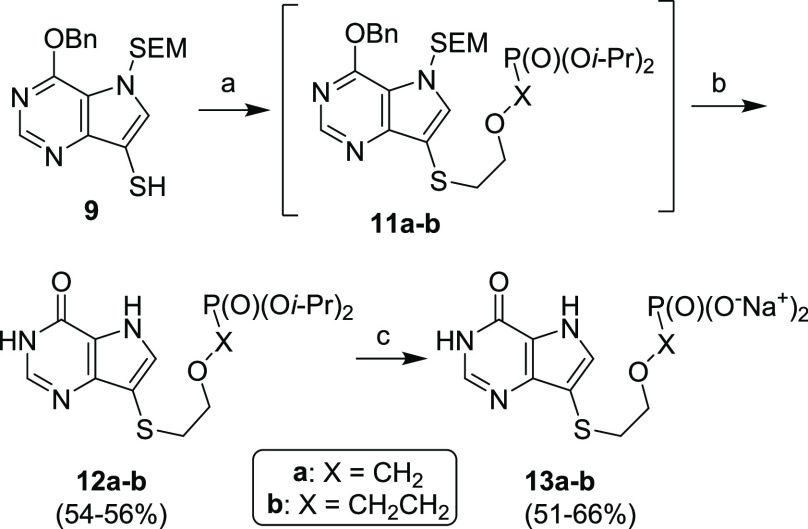
Synthesis of Aliphatic Acyclic Nucleoside Phosphonates (ANPs) 1**3a–b** from compound **9** Reagents and conditions: (a) PME-Cl or PEE-Cl, NaH, DMF, 80 °C, overnight; (b) (1) TFA, rt, 15 min; (2) NH_3_/EtOH, rt, 1 min; (c) (1) TMSBr, pyridine, rt, overnight; (2) Dowex 50-Na^+^.

For instance, thiol **9** (in the form of disulfide **10**) was directly alkylated with aliphatic alkylating agents containing phosphonate moieties PME (phosphonomethoxyethyl) and PEE (phosphonoethoxyethyl) (Scheme 5). The removal of the purine protecting groups with TFA (to get compounds **12a–b**), followed by a cleavage of the phosphonate esters with TMSBr in pyridine, afforded target compounds **13a–b** in good overall yields. Phosphonates were formulated as sodium salts using Dowex 50 in a sodium cycle. This procedure conveniently afforded analogs of acyclic nucleoside phosphonates (ANPs), represented for instance by 9-((2-phosphonylmethoxy)ethyl)guanine (PMEG). ANPs are a well-known group of antiviral drugs with a broad scope of therapeutic applications.^45,46^

**Scheme 6:**
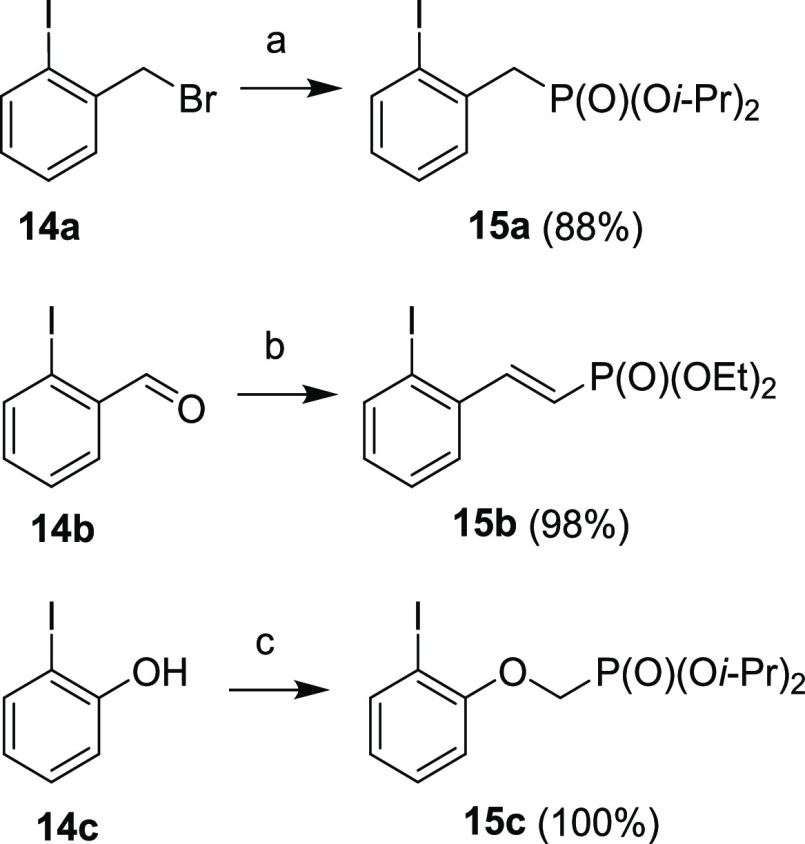
Synthesis of Aryl Iodides **15a–c** Reagents and conditions: (a) P(O*i*-Pr)_3_, toluene, MW, 160 °C, 1 h; (b) CH_2_[P(O)(OEt)_2_]_2_, 5 M NaOH (aq), DCM, rt, 48 h; (c) TfOCH_2_P(O)(O*i*-Pr)_2_, NaH, THF, 0 °C, 1 h.

In the next step, a series of aryl iodides **15a–c** (Scheme 6) bearing the phosphonate moiety were synthesized to be later coupled with compound **9**. Compound **15a** was synthesized via Arbuzov reaction from benzyl bromide **14a** and triisopropylphosphite. Compound **15b** was prepared by Horner–Wadsworth–Emmons (HWE) reaction from benzaldehyde **14b** and tetraethyl methylenediphosphonate. Finally, an alkylation of phenol **14c** with diisopropyl triflyloxymethanephosphonate afforded compound **15c**.

**Scheme 7:**
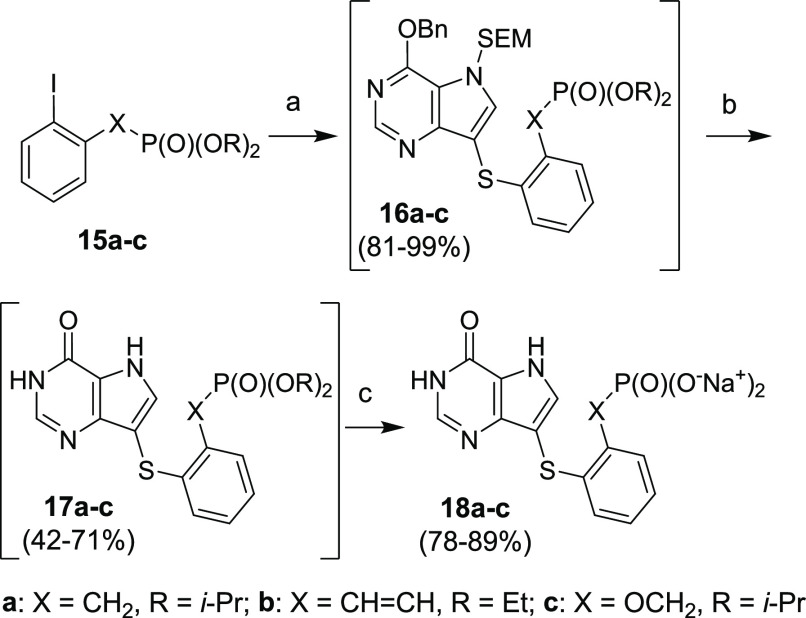
Synthesis of PNP Inhibitors Containing the Phosphonate Moiety Reagents and conditions: (a) compound **9**, CuI, 2-isobutyrylcyclohexanone, Cs_2_CO_3_, toluene, MW, 120 °C, 1–3 h; (b) (1) TFA, rt, 15 min; (2) NH_3_/EtOH, rt, 1 min; (c) (1) TMSBr, pyridine, rt, overnight; (2) Dowex 50-Na^+^.

Conditions for the subsequent Ullmann coupling between compound **9** and aryl iodides **15a–c** had to be slightly modified (Scheme 7). 1,10-Phenantroline was replaced with 2-isobutyrylcyclohexanone, and triethylamine was replaced with cesium carbonate. Such conditions afforded desired products **16a–c** in high yields. The subsequent removal of the protecting groups by TFA (giving compound **17a–c**) and with TMSBr afforded target compounds **18a–c**.

**Scheme 8:**
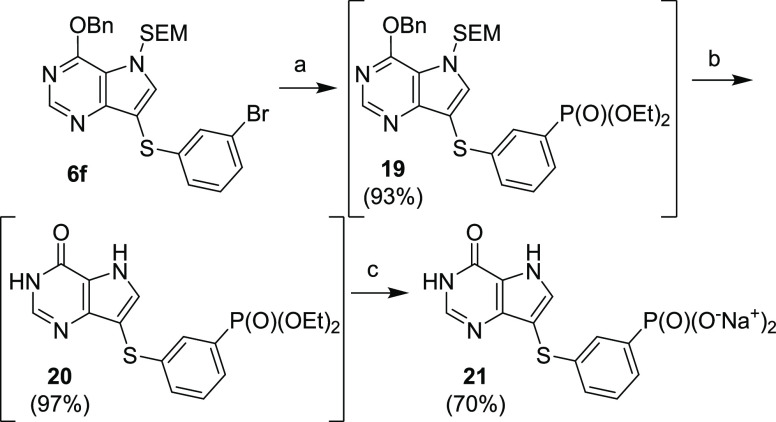
Synthesis of *meta*-Phenylphosphonate **21** Reagents and conditions: (a) HP(O)(OEt)_2_, Pd_2_(dba)_3_, xantphos, Et_3_N, dioxane, 100 °C, on; (b) (1) TFA, rt, 15 min; (2) NH_3_/EtOH, rt, 1 min; (c) (1) TMSBr, pyridine, rt, overnight; (2) Dowex 50-Na^+^.

*meta*-Phenylphosphonate **21** (Scheme 8) was synthesized from compound **6f** via Pd-catalyzed introduction of diethyl phosphite (to give compound **19**), followed by the above-mentioned deprotections.

**Scheme 9:**
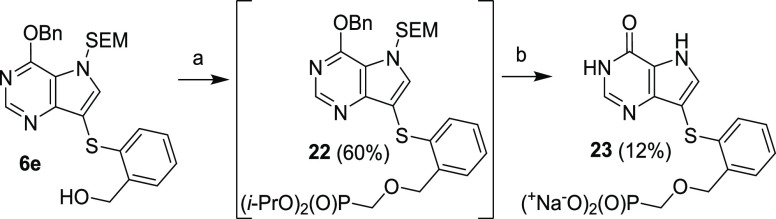
Synthesis of Phosphonate **23** Reagents and conditions: (a) TfOCH_2_P(O)(O*i*-Pr)_2_, NaH, THF, 0 °C, 1 h; (b) (1) TFA, rt, 15 min; (2) NH_3_/EtOH, rt, 1 min; (3) TMSBr, pyridine, rt, overnight; (4) Dowex 50-Na^+^.

The prolonged analog of compound **18c**, compound **23** (Scheme 9), was synthesized from compound **6e** via alkylation with diisopropyl triflyloxymethylphosphonate.

**Figure 3:**
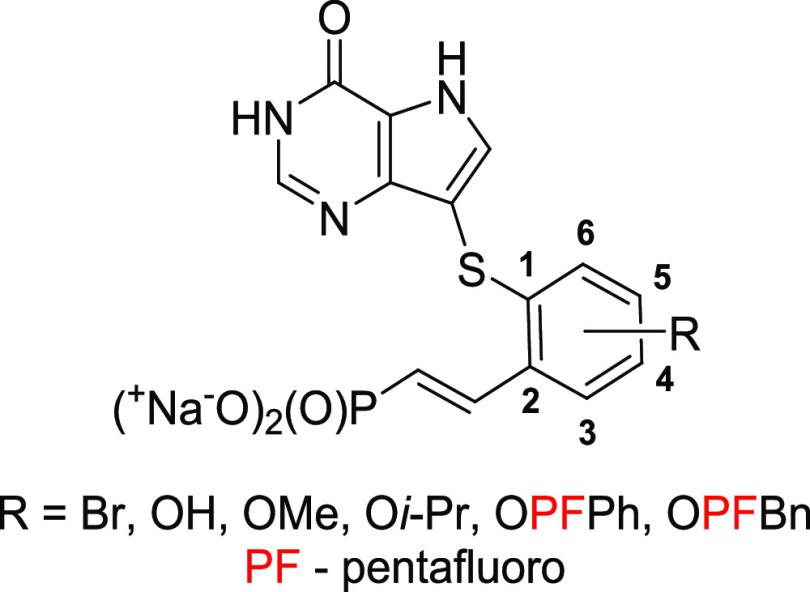
Target compounds with the extra-substituted central phenyl scaffold.

A preliminary *in vitro* screening revealed that compounds **18b** and **18c** exhibited low nanomolar inhibitory activities against hPNP (IC_50_ = 0.021 and 0.022 μM, respectively) and *Mt*PNP (0.025 and 0.031 μM, respectively) (Table 3). Encouraged by these results, we decided to focus on additional substitutions of the central phenyl ring (Figure 3), which would disclose the spatial properties of the enzymes’ binding sites. Detailed *in vitro* activity results and structure–activity relationship analysis will be discussed in the following chapters (Table 3).

We decided to derivatize the phenyl moiety with bromine, hydroxyl, methoxy, isopropoxy, pentafluorophenyloxy (PFPhO), and pentafluorobenzyloxy (PFBnO) groups. This allowed us to study the effects of different geometric and steric properties of the substituents on the binding properties of the PNP inhibitors. Fluorinated substituents (PFPhO and PFBnO) were chosen for their synthetic feasibility and stability over the corresponding nonfluorinated analogs. The binding model of compound **18b** built by molecular dynamics simulation of hPNP structure (PDB: 3BGS, resolution 2.10 Å) showed that only phenyl positions C3, C4, and C5 (Figure 3) were suitable for such derivatization since position C6 would lead to an intramolecular clash within the biding. We do not provide additional information on this model since accurate crystal structures were obtained later.

The synthetic route toward the phenyl-substituted derivatives was analogous to the synthesis presented in Scheme 7. First, suitably substituted aryl iodides were prepared, followed by the attachment of the protected 9-deazahypoxanthine and final deprotection.

**Scheme 10:**
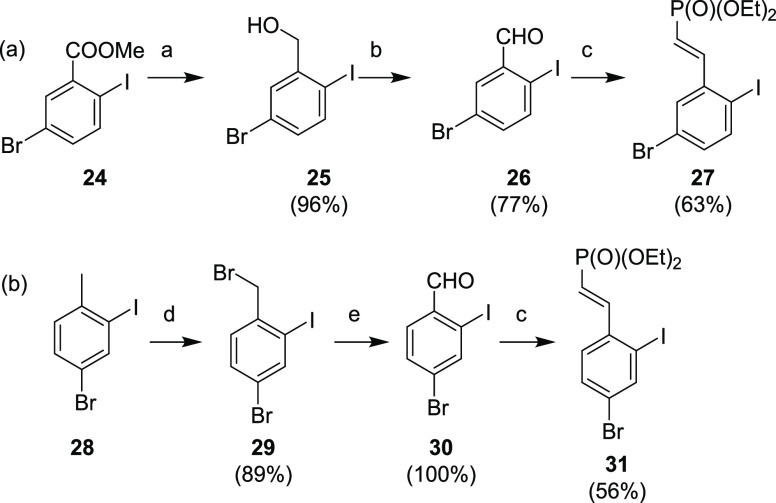
Synthesis of Brominated Analogs **27** and **31** Reagents and conditions: (a) DIBAH, DCM, 0 °C–rt, overnight; (b) PDC, DCM, rt, 4 h; (c) CH_2_[P(O)(OEt)_2_]_2_, *t*-BuO^–^K^+^, THF, rt, 1 h; (d) NBS, (BzO)_2_, DCE, reflux, 4 h; (e) NMMO, 4 Å mol sieves, ACN, 0 °C, 2 h.

4-Bromo aryl iodide **27** was prepared from commercially available ester **24** (Scheme 10a). Reduction of the ester group afforded benzyl alcohol **25**, which was oxidized to aldehyde **26**. Finally, compound **27** was prepared from aldehyde **26** via HWE-reaction using tetraethyl methylenediphosphonate. 5-Bromo analog **31** was prepared from toluene **28** (Scheme 10b), in which the methyl group was derivatized via radical bromination. Benzyl bromide **29** was then oxidized with NMMO to aldehyde **30**, which underwent the above mentioned HWE-reaction to afford compound **31**.

**Scheme 11:**
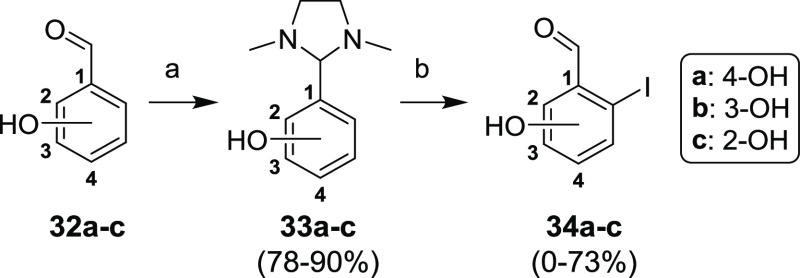
Attempted Synthesis of Hydroxybenzaldehydes **34a–c** via *ortho*-Lithiation Reagents and conditions: (a) (CH_3_NHCH_2_)_2_, toluene, reflux, 15 min; (b) (1) *t*-BuLi, Et_2_O, −78 °C–rt, overnight; (2) (CH_2_I)_2_, Et_2_O, −78 °C–rt, 30 min.

**Scheme 12:**
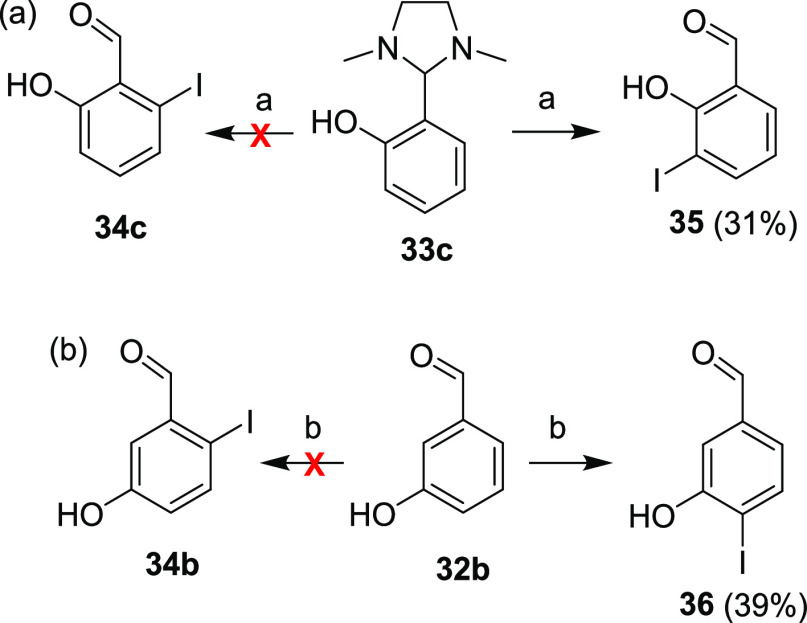
Unintended Preparation of Hydroxybenzaldehydes **35** and **36** Reagents and conditions: (a) *t*-BuLi, Et_2_O, −78 °C–rt, overnight; (2) (CH_2_I)_2_, Et_2_O, −78 °C–rt, 30 min. (b) I_2_, KI, NH_4_OH, H_2_O, rt, 3 h.

Synthesis of hydroxyl derivatives was found to be quite challenging. Our intention was to start from commercially available hydroxyl aldehydes **32a–c** (Scheme 11). Protection of the aldehyde group with *N*,*N*′-dimethylethylenediamine afforded compounds **33a–c** in high yields, and the compounds were expected to undergo an *ortho*-lithiation by *tert*-butyllithium. The reaction of lithiated intermediates with 1,2-diiodoethane provided the desired product only in the case of 4-hydroxy regioisomer **33a** in a 73% yield. The reaction with 3-hydroxy isomer **33b** leads to a mixture of regioisomers with a very low conversion of the starting material. 2-Hydroxy regioisomer **33c** directed lithiation only to the *ortho*-position with respect to the hydroxyl group to afford undesired compound **35** (Scheme 12a). It is noteworthy that diiodomethane as a source of iodine secured deprotected formyl groups without the need for subsequent deprotection, whereas elemental iodine afforded imidazolidine-protected products.

An attempt to synthesize compound **34b** was made using a procedure published in a recent patent (Scheme 12b).^47^ Electrophilic aromatic iodination of compound **32b** with nitrogen triiodide should afford isomer **34b**; however, it was proved that this procedure only led to isomer **36**.

**Scheme 13:**
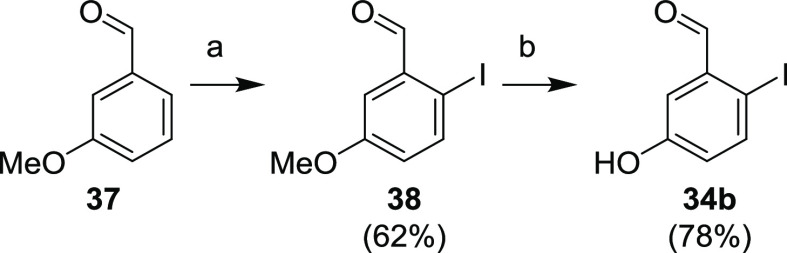
Synthesis of Benzaldehyde **34b** Reagents and conditions: (a) I_2_, AgNO_3_, MeOH, rt, 1 h; (b) (1) BBr_3_, DCM, −78 °C–rt, overnight; (2) aq NaHCO_3_.

Compound **34b** was finally synthesized from 3-methoxybenzaldehyde (**37**) via electrophilic aromatic iodination (to give **38**), followed by demethylation of the methoxy group with BBr_3_ (Scheme 13).

**Scheme 14:**
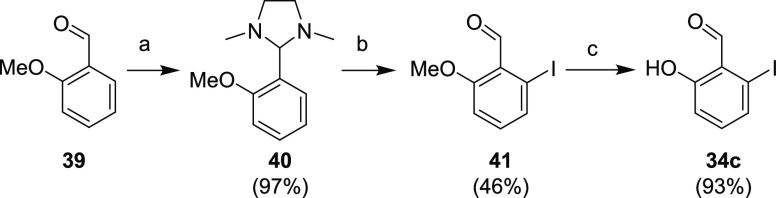
Synthesis of Benzaldehyde **34c** Reagents and conditions: (a) (CH_3_NHCH_2_)_2_, toluene, reflux, 15 min; (b) (1) *t*-BuLi, Et_2_O, −78 °C–rt, overnight; (2) (CH_2_I)_2_, Et_2_O, −78 °C–rt, 30 min; (c) (1) BBr_3_, DCM, −78 °C–rt, overnight; (2) aq. NaHCO_3_.

2-Hydroxybenzaldehyde **34c** was obtained via protection of 2-methoxybenzaldehyde (**39**), followed by consecutive *ortho*-lithiation and iodination of intermediate **40**, and final demethylation of the methoxy group in derivative **41** (Scheme 14).

**Scheme 15:**
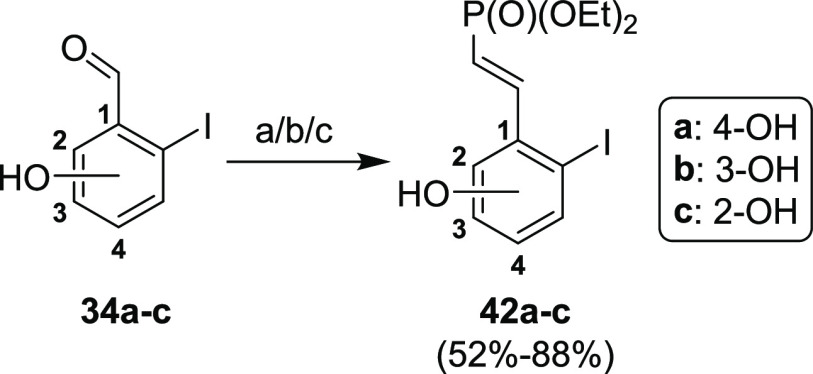
Synthesis of Vinyl Phosphonates **42a–c** Reagents and conditions: (a) [P(O)(OEt)_2_]CH_2_COOH, piperidine, acetic acid, toluene, reflux, overnight, 87%; (b) [P(O)(OEt)_2_]_2_CH_2_, K_2_CO_3_, EtOH, reflux, 1 h, 88%; (c) [P(O)(OEt)_2_]_2_CH_2_, NaH, DMF, 40 °C, overnight.

Subsequent transformation of the formyl group in compounds **34a–c** to the vinyl(diethoxyphosphoryl) moiety differed for each regioisomer (Scheme 15). 4-Hydroxy regioisomer **34a** was transformed *via* Knoevenagel condensation with (diethoxyphosphoryl)acetic acid in a piperidine/acetic acid catalytic system (conditions (a)). Conditions for HWE-reaction lead only to low conversions of the starting material. The Knoevenagel condensation with 2-hydroxy regioisomer **34c** formed a coumarin structure (data not showed) in which carboxylic acid moiety did not undergo decarboxylation but instead formed an intermolecular ester with the phenolic hydroxyl group. On the other hand, The HWE-reaction of **34c** afforded compound **42c** in a 52% yield. However, 3-hydroxybenzaldehyde **34b** did not react under any of these conditions. After a substantial optimization (data not shown), we discovered that protic solvents were needed to achieve at least some conversion of the starting material. Finally, the HWE-reaction with potassium carbonate as a base and ethanol as a solvent under heating afforded desired product **42b** in an excellent yield of 88%. Interestingly, these conditions did not afford any product when using regioisomers **34a** and **34c**.

**Scheme 16:**
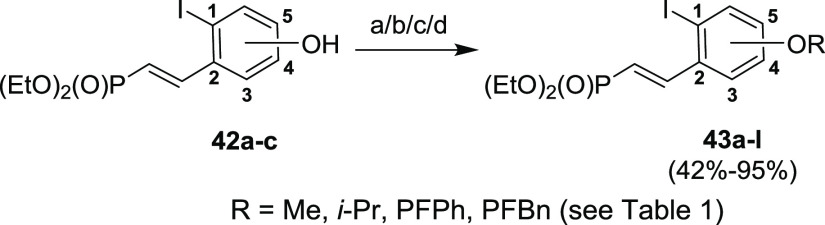
Synthesis of Vinyl Phosphonates **43a–l** Reagents and conditions: (a) MeI, NaH, DMF, 0 °C–rt, 1 h; (b) *i*-PrI, NaH, DMF, 0 °C–rt, 1 h; (c) C_6_F_5_, NaH, DMF, 70 °C, 1 h; (d) PFBnBr, NaH, DMF, 0 °C–rt, 1 h.

Phosphonates **42a–c** bearing the phenolic hydroxyl group were further converted to compounds **43a–l** (Scheme 16, Table 1) by alkylation with methyl iodide, isopropyl iodide, or pentafluorobenzyl bromide, or by arylation with hexafluorobenzene. As mentioned above, we selected the pentafluorobenzyl group (PFBn) for its stability in an acidic environment (in contrast to a benzyl group) and the pentafluorophenyl group (PFPh) for its simple synthetic feasibility using a nucleophilic aromatic substitution of hexafluorobenzene (in contrast to difficult arylations with benzenes). The reaction yields are summarized in Table 1.

**Table 1:** Synthesis of Compounds **43a–l**^a^

position	R-group	comp	conditions	yield (%)
C5	Me	**43a**	a	89
C5	*i*-Pr	**43b**	b	84
C5	PFPh	**43c**	c	74
C5	PFBn	**43d**	d	95
C4	Me	**43e**	a	63
C4	*i*-Pr	**43f**	b	72
C4	PFPh	**43g**	c	42
C4	PFBn	**43h**	d	54
C3	Me	**43i**	a	93
C3	*i*-Pr	**43j**	b	88
C3	PFPh	**43k**	c	50
C3	PFBn	**43l**	d	91

a Reaction conditions and yields.

**Scheme 17:**
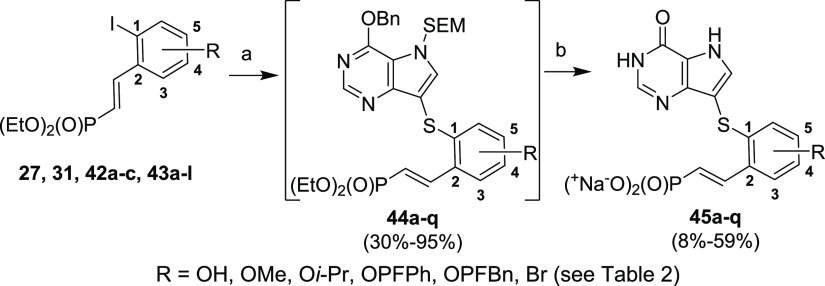
Synthesis of Target Phosphonates **45a–q** Reagents and conditions: (a) compound **9**, CuI, 2-isobutyrylcyclohexanone, Et_3_N, DMF, MW, 120 °C, 1–3 h; (b) (1) TFA, rt, 15 min; (2) NH_3_/EtOH, rt, 1 min; (3) TMSBr, pyridine, rt, overnight; (4) Dowex 50-Na^+^.

The Ullmann coupling of compounds **27**, **31**, **42a–c**, and **43a–l** with thiol **9** at 120 °C smoothly afforded protected intermediates **44a–q** usually in good yields (Scheme 17, Table 2). Only compound **44k** with the unprotected hydroxyl group next to the phosphonate linker was synthesized under milder conditions (reaction temperature 100 °C instead of 120 °C) as higher temperature caused double-bond isomerization and an intramolecular re-esterification of the phosphonate ester with the phenolic hydroxyl group, forming a coumarin-like structure (data not showed). Lower reaction temperature completely prevented the formation of such a byproduct. Protected intermediates **44a–q** were converted into final phosphonates **45a–q** by the standard (above described) procedure. Yields are summarized in Table 2.

**Table 2:** Synthetic Yields of Vinylphosphonates **44a–q** and **45a–q**

position	R-group	code	yield (%)	code	yield (%)
C5	OH	**44a**	30	**45a**	23
C5	OMe	**44b**	62	**45b**	59
C5	O*i*-Pr	**44c**	62	**45c**	28
C5	OPFPh	**44d**	60	**45d**	12
C5	OPFBn	**44e**	72	**45e**	8
C4	OH	**44f**	73	**45f**	13
C4	OMe	**44g**	95	**45g**	50
C4	O*i*-Pr	**44h**	85	**45h**	38
C4	OPFPh	**44i**	81	**45i**	51
C4	OPFBn	**44j**	69	**45j**	51
C3	OH	**44k**	60^a^	**45k**	16
C3	OMe	**44l**	70	**45l**	51
C3	O*i*-Pr	**44m**	91	**45m**	32
C3	OPFPh	**44n**	84	**45n**	59
C3	OPFBn	**44o**	59	**45o**	59
C5	Br	**44p**	34	**45p**	49
C4	Br	**44q**	40	**45q**	49

a Reaction temperature was 100 °C (instead of 120 °C).

**Scheme 18:**
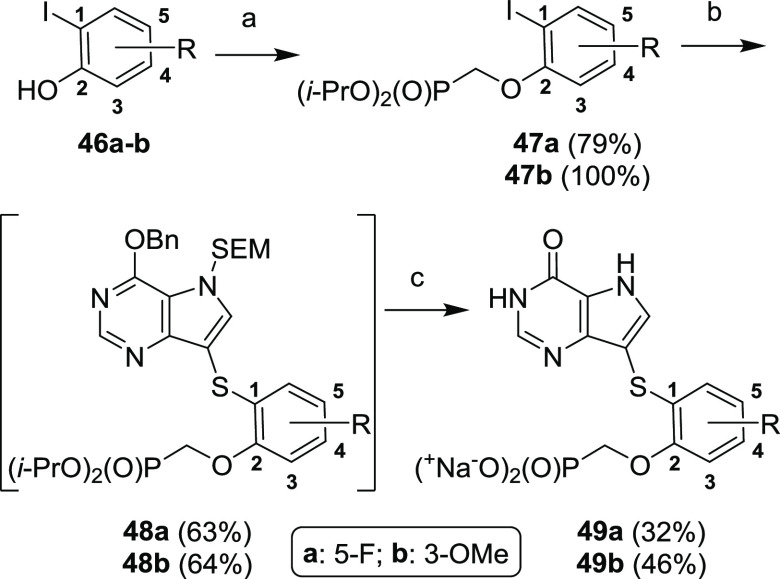
Synthesis of Oxamethylphosphonates **49a–b** Reagents and conditions: (a) TfOCH_2_P(O)(O*i*-Pr)_2_, NaH, THF, 0 °C, 1 h; (b) compound **9**, CuI, 2-isobutyrylcyclohexanone, Et_3_N, DMF, MW, 120 °C, 1–3 h; (c) (1) TFA, rt, 15 min; (2) NH_3_/EtOH, rt, 1 min; (3) TMSBr, pyridine, rt, overnight; (4) Dowex 50-Na^+^.

In addition to aryl-substituted vinylphosphonates **45a–q**, we wanted to explore some of those substitutions in combination with more flexible and more acidic oxamethylphosphonate moiety (Scheme 18). Based on SAR of the previous series, 3-methoxy and 5-fluoro substituents were selected for our target compounds. The synthesis began with alkylation of commercially available phenols **46a–b** with diisopropyl triflyloxymethanphosphonate to yield aryl iodides **47a**–**b** in a 79 and 100% yield, respectively. The subsequent Ullmann coupling (to give compounds **48a**–**b**), followed by the removal of the protecting groups afforded target compounds **49a–b** analogously to the previous synthesis.

Since phosphonic acids are ionized under physiological pH, we also synthesized several examples of prodrugs derived from phosphonate **18c** (Scheme 7), which are based on bisamidates and ester/amidate prodrugs to secure passive transport of such compounds into the cells. We chose isopropyl esters of alanine and phenylalanine as amino acid moieties and phenol as the ester moiety in analogy to approved antiviral drug tenofovir alafenamide fumarate (TAF).^48,49^

**Scheme 19:**
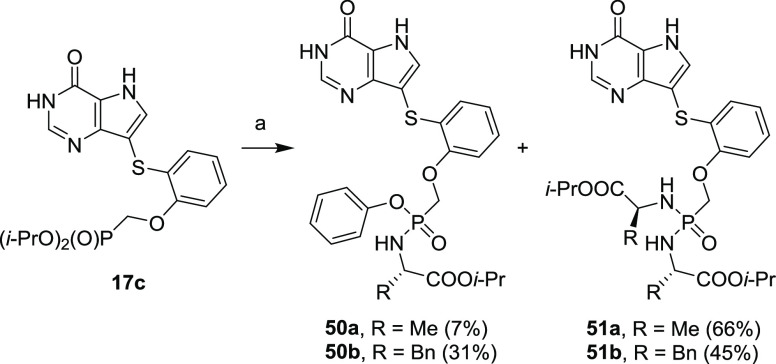
Synthesis of Phosphonate Prodrugs Reagents and conditions: (a) (1) TMSBr, pyridine, rt, overnight; (2) TEAB, rt, 5 min; (3) l-amino acid ethyl ester hydrochloride, phenol, Et_3_N, 2-Aldrithiol, PPh_3_, pyridine, 60 °C, overnight.

A one-pot approach published by Šmídková et al.,^50^ based on an ester cleavage followed by coupling of amino acid/phenol promoted by 2-Aldrithiol/triphenylphosphine, afforded a mixture of bisamidate (compounds **51a–b**) and ester/amide (compounds **50a–b**) prodrugs (which could be separated from each other) in moderate yields (Scheme 19). However, prodrug **50a**, an analog of TAF, was obtained only in a low yield of 7%.

**Scheme 20:**
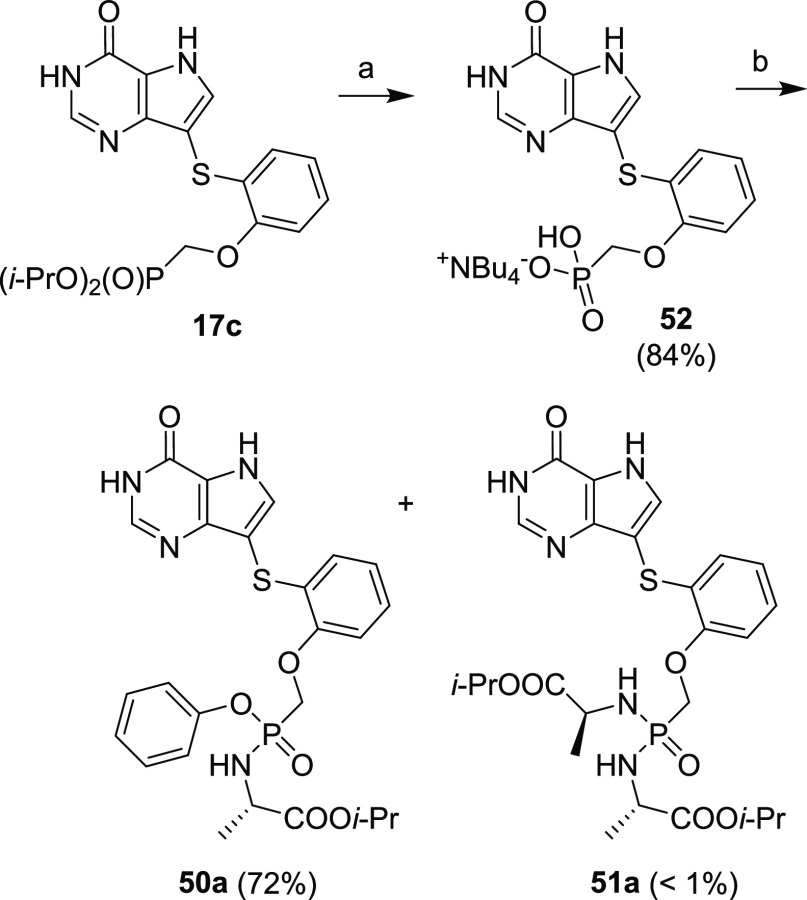
Optimized Synthesis of TAF-like Prodrug **50a** Reagents and conditions: (a) (1) TMSBr, pyridine, rt, overnight; (2) TEAB, TBAH, rt, 5 min; (b) l-alanine ethyl ester hydrochloride, phenol, Et_3_N, 2-Aldrithiol, PPh_3_, pyridine, 60 °C, overnight.

It is well-known that salt formulation of phosphonic acid greatly affects the reactivity of the phosphonate group. Therefore, we optimized the preparation of monotetrabutylammonium salt of compound **18c** (as the sodium salt, Scheme 7), i.e., compound **52** (Scheme 20). Compound **52**, obtained from phosphonate **17c** in an 84% yield, was then subjected to the amino acid/phenol coupling, which provided exclusively desired ester/amide prodrug **50a** in a 72% yield.

**Scheme 21:**
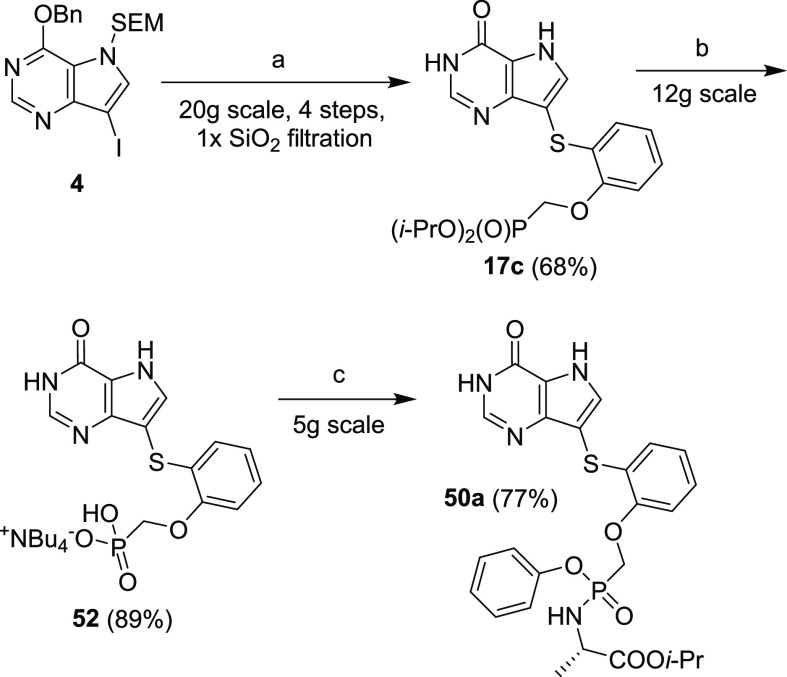
Optimized Multigram-Scale Synthesis of Compound **52** and Prodrug **50a** Reagents and conditions: (a) (1) 2-mercaptophenol, CuI, 1,10-phenanthroline, Et_3_N, toluene, 120 °C, 2 h; (2) TsOCH_2_P(O*i*-Pr)_2_, *t*-BuO^–^K^+^, DMF, 60 °C, 3 h; (3) TFA, rt, 15 min; (4) NH_3_/EtOH, rt, 1 min; (b) (1) TMSBr, pyridine, rt, overnight; (2) TEAB, TBAH, rt, 5 min; (c) l-alanine ethyl ester hydrochloride, phenol, Et_3_N, 2-Aldrithiol, PPh_3_, pyridine, 60 °C, overnight.

Finally, a multigram-scale synthesis of compounds **50a** and **52** was optimized to support DMPK and efficacy studies (Scheme 21). We manage to telescope the first four steps of the synthesis (starting from compound **4**) with only one fast chromatography (filtration) on silica gel to acquire intermediate **17c** in a 68% yield. The subsequent transformations to tetrabutylammonium salt **52** and prodrug **50a** (with yields of 89 and 77%, respectively) were achieved with optimized procedures depicted in Scheme 21.

### Biology

All prepared compounds were evaluated in the enzymatic assays for inhibition of recombinant PNP from three species—*Homo sapiens* (hPNP), *Mycobacterium tuberculosis* (*Mt*PNP), and *Plasmodium falciparum* (*Pf*PNP), and data are summarized in Table 3. Subsequently, cytotoxic effects were determined on three T-cell cell lines (CCRF-CEM, MOLT-4, and Jurkat—see Table 3) and three off-target cancerous cell lines (HL60, HepG2, and HeLa S3—see Table S9). Selected compounds were also tested for undesired immunotoxicity in primary PBMCs and their T-cell-enriched fraction isolated from healthy volunteers. No significant toxicity was observed at concentrations up to 50 μM (Table S9).

**Table 3:** Biological Evaluation of Novel PNP Inhibitors^a^

code	structural feature	hPNP IC_50_ [μM]	*Mt*PNP IC_50_ [μM]	CCRF-CEM CC_50_ [μM]	MOLT-4 CC_50_ [μM]	Jurkat CC_50_ [μM]
**7a**	Ph-S–	1.425	6.187	4.877	>10	>10
**7b**	2-MePh-S–	>10	ND^b^	>10	ND^b^	ND^b^
**7c**	2-BrPh-S–	>10	ND^b^	>10	ND^b^	ND^b^
**7d**	2-(HO)Ph-S–	0.787	0.364	1.816	>10	>10
**7e**	2-(HOCH_2_)Ph-S–	>10	4.119	>10	>10	>10
**7f**	3-BrPh-S–	2.730	ND^b^	>10	ND^b^	ND^b^
**13a**	(P(O)(OH)_2_)CH_2_OCH_2_CH_2_-S–	1.195	0.644	5.900	0.638	12.99
**13b**	(P(O)(OH)_2_)CH_2_CH_2_OCH_2_CH_2_-S–	0.494	0.166	0.045	0.093	5.700
**18a**	2-(P(O)(OH)_2_)CH_2_Ph-S–	0.097	0.176	0.104	0.037	2.110
**18b**	2-(P(O)(OH)_2_)CH=CHPh-S–	0.021	0.025	0.026	0.380	0.130
**18c**	2-(P(O)(OH)_2_)CH_2_OPh-S–	0.022	0.031	0.034	0.290	0.189
**21**	3-(P(O)(OH)_2_)Ph-S–	5.627	10.000	2.560	1.530	21.80
**23**	2-(P(O)(OH)_2_)CH_2_OCH_2_Ph-S–	0.071	0.025	0.031	0.045	0.070
**45a**	5-HO-2-(P(O)(OH)_2_)CH=CHPh-S–	0.029	0.048	0.054	0.155	0.130
**45b**	5-MeO-2-(P(O)(OH)_2_)CH=CHPh-S–	0.134	0.036	0.094	0.130	0.200
**45c**	5-*i*PrO-2-(P(O)(OH)_2_)CH=CHPh-S–	1.038	0.016	0.920	>10	>10
**45d**	5-PFPhO-2-(P(O)(OH)_2_)CH=CHPh-S–	1.770	0.029	>10	>10	>10
**45e**	5-PFBnO-2-(P(O)(OH)_2_)CH=CHPh-S–	0.831	0.028	1.460	>10	4.870
**45f**	4-HO-2-(P(O)(OH)_2_)CH=CHPh-S–	0.149	0.026	0.118	0.186	0.248
**45g**	4-MeO-2-(P(O)(OH)_2_)CH=CHPh-S–	0.153	0.007	0.035	0.126	0.102
**45h**	4-*i*PrO-2-(P(O)(OH)_2_)CH=CHPh-S–	0.180	0.004	0.033	0.110	0.147
**45i**	4-PFPhO-2-(P(O)(OH)_2_)CH=CHPh-S–	0.043	0.025	0.049	0.382	0.459
**45j**	4-PFBnO-2-(P(O)(OH)_2_)CH=CHPh-S–	0.251	0.118	0.044	0.327	0.320
**45k**	3-HO-2-(P(O)(OH)_2_)CH=CHPh-S–	0.709	0.145	0.230	3.320	1.170
**45l**	3-MeO-2-(P(O)(OH)_2_)CH=CHPh-S–	0.058	0.019	0.019	0.017	0.026
**45m**	3-*i*PrO-2-(P(O)(OH)_2_)CH=CHPh-S–	0.019	0.033	0.052	0.172	0.104
**45n**	3-PFPhO-2-(P(O)(OH)_2_)CH=CHPh-S–	0.054	0.012	0.013	0.031	0.042
**45o**	3-PFBnO-2-(P(O)(OH)_2_)CH=CHPh-S–	0.049	0.019	0.015	0.013	0.017
**45p**	5-Br-2-(P(O)(OH)_2_)CH=CHPh-S–	0.035	0.052	0.210	1.248	0.544
**45q**	4-Br-2-(P(O)(OH)_2_)CH=CHPh-S–	0.081	0.030	0.009	0.045	0.030
**49a**	5-F-2-(P(O)(OH)_2_)CH_2_OPh-S–	0.033	0.020	0.018	0.164	0.750
**49b**	3-MeO-2-(P(O)(OH)_2_)CH_2_OPh-S–	0.122	0.033	0.550	5.670	13.96
**50a**	phenoxy/alanine prodrug of **18c**	>10	>10	0.033	0.535	0.640
**50b**	**18c** phenoxy/phenylalanine prodrug	>10	>10	0.036	0.151	0.144
**51a**	**18c** bis(alanine) prodrug	>10	>10	0.565	0.543	1.200
**51b**	**18c** (bis)phenylalanine prodrug	>10	>10	ND^b^	ND^b^	ND^b^
forodesine		0.057	0.042	0.003	0.004	0.003

a Ph = phenyl.

b ND—not determined.

**Figure 4:**
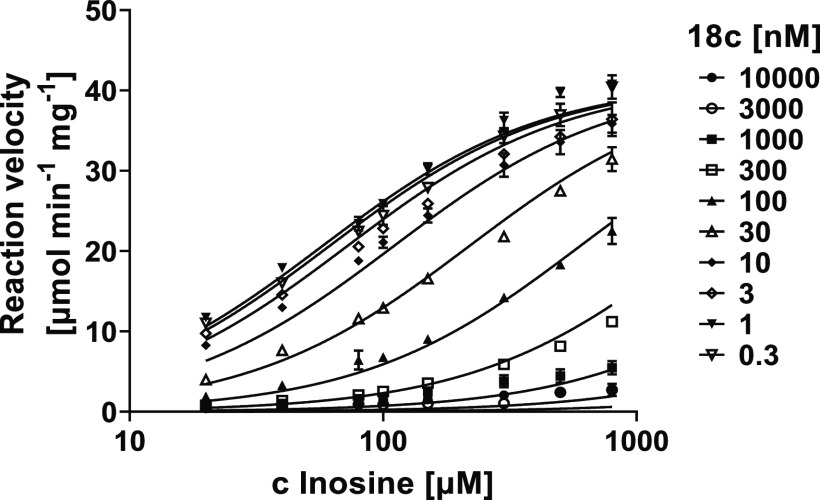
Inhibition kinetics of compound **18c** against hPNP (competitive model).

In general, none of the tested compounds showed inhibitory activity on *Pf*PNP up to 10 μM concentration (data not shown), except for forodesine with IC_50_ = 0.103 μM. Inhibitors of *h*PNP exhibited highly selective cytotoxicity toward T-cell-derived leukemia cells, while they did not affect the viability of HL60, HepG2, or HeLa S3 cell lines at up to 10 μM concentration (data not included in Table 3). Most of the inhibitors exhibited slightly better potency toward *Mt*PNP compared to hPNP, which might be attributed to different *K_m_* values of the enzymes (41 and 217 μM, respectively). All inhibitors inhibited the enzymes in a competitive manner as shown for compound **18c** (Figure 4). The estimated *K_i_* values for the selected hPNP inhibitor of the series, compound **18c**, and for the reference inhibitor (forodesine) were 12 and 33 nM, respectively.

Compounds **7a–f** containing phenyl moieties without the phosphonate group showed no or weak inhibition of hPNP, with 2-hydroxyl derivative **7d** as the best compound in the series (an IC_50_ value of 0.787 μM). Moreover, only low potencies were observed against *Mt*PNP, again with compound **7d** (2-hydroxyl derivative, IC_50_ = 0.364 μM). Some of those derivatives inhibited T-cell proliferation in a micromolar range (the most potent **7d** with IC_50_ = 1.816 μM). Such poor results were surprising since compound **7a** has been reported as an inhibitor of isolated human erythrocytic PNP with IC_50_ = 113 nM,^35^ while in our hands, it had IC_50_ = 1.429 μM against the recombinant hPNP, and a similar outcome was observed in the cellular assays (T-cell proliferation).

Most compounds bearing the phosphonate group exhibited high potencies against hPNP and *Mt*PNP, with IC_50_ values lower than 0.1 μM. It was confirmed that compounds **18b**, **18c**, and **23** (with linkers consisting of 2–3 atoms between phenyl and phosphonate moieties, linkers attached to the phenyl via *ortho*-position) exhibited a good binding as suggested by several previous reports.^24,26^ Structures with two-atom linkers, vinyl (**18b**) and oxamethyl (**18c**), were selected for further development since they inhibited hPNP, *Mt*PNP, and CCRF-CEM proliferation with comparable IC_50_ values between 21 and 35 nM.

Derivatives **45a–q** based on compound **18b** with additional substitutions at phenyl positions C3, C4, and C5 with respect to the sulfur bridge afforded new information on selectivity and steric limits of the enzymes’ binding pockets.

Substitutions at position C5 (compounds **45a–e**) led to a decrease in potency against hPNP, which correlated with the size of the group. IC_50_ values for hPNP dropped from 21 nM for compound **18b** (nonsubstituted) to 1.77 μM for compound **45d** (bearing pentafluorophenoxy group), whereas inhibitory activity toward *Mt*PNP changed negligibly within the series resulting in an 84-fold selectivity toward the pathogenic target over the human enzyme. Moreover, compound **45d** did not inhibit T-cell proliferation up to 10 μM concentration. These properties are rather promising, and the compounds possess great therapeutic potential as antimicrobial agents. To our best knowledge, this is the first example of such selectivity between human and pathogenic enzymes achieved for PNP inhibitors by specific structural modifications.

Substitutions at phenyl position C4 with respect to the sulfur bridge (compounds **45f–j**) led only to a small decrease in activity against hPNP (2–12 folds) while retaining or improving activity against *Mt*PNP. Compound **45h** (isopropoxy derivative) exhibited the best potency against *Mt*PNP among all compounds with IC_50_ = 4 nM. Also, T-cell antiproliferative activity was affected only marginally within this series of compounds.

Substituents at phenyl position C3 (compounds **45k–p**) were also well tolerated in both hPNP and *Mt*PNP enzymes. Only 3-hydroxyl derivative **45k** exhibited significantly decreased activity in all assays. One can hypothesize that this might be a result of an intramolecular interaction between the phosphonate group and hydroxyl group via intramolecular hydrogen bonding. Other derivatives exhibited comparable activities against both enzymes, while their activity in a cell-based assay was greatly improved, probably because of the increased lipophilicity of these inhibitors. Similar outcomes were observed for bromo-derivatives **45p–q** , which exhibited similar cellular activity, with compound **45q** being the most active from the series (CCRF-CEM IC_50_ = 9 nM).

Two examples of substituted structures based on compound **18c** (containing oxamethyl linker) were prepared. 5-Fluoro derivative **49a** exhibited similar IC_50_ values against hPNP and *Mt*PNP (33 and 20 nM, respectively) and slightly improved potency against CCRF-CEM cells compared to the original compound **18c** (18 vs 34 nM). Compound **49b**, the 3-methoxy analog of compound **45l**, exhibited substantially lower activity against hPNP and T-cell proliferation (0.122 and 0.550 μM). *Mt*PNP inhibitory activity was comparable to **45l** (33 vs 19 nM, respectively).

Bisamide prodrugs **51a–b** derived from compound **18c** showed significantly lower T-cell antiproliferative activities compared to the parent compound (0.565 μm vs 34 nM). This can be attributed to the high metabolic stability of bisamide prodrugs. Mixed amide-ester prodrugs **50a–b**, however, exhibited comparable activities to the parent compound (33 and 36 nM, respectively), providing noncharged lipophilic versions of compound **18c**.

### Pharmacology

Compounds **18c** and **50a** were selected for further pharmacokinetic study. Compound **18c** showed high pH-dependent aqueous kinetic solubility ranging from 174 μM at pH 3.0 to more than 400 μM at pH 9.0 (Table 4). Compound **50a**, a lipophilic prodrug, exhibited lower aqueous solubility independent on pH (ranging from 53 to 115 μM).

**Table 4:** Aqueous Kinetic Solubility of Selected Compounds at Various pHs

	aqueous kinetic solubility [μM]			
compound	glycine buffer	PBS	FaSSIF	citrate buffer
@pH	9.0	7.4	6.5	3.0
**18c**	>400	381	291	174
**50a**	115	53	60	63

The stability of the selected compounds was evaluated in blood plasma, liver microsomes, and liver S9 fraction from three species—mouse, rat, and human (Table 5). Compound **18c** proved to be metabolically stable with very low intrinsic clearance, whereas its prodrug, compound **50a**, exhibited very high microsomal and plasma clearance in all assays across all species. Although in principle, prodrugs should be metabolically cleavable to deliver the target compound, the observed clearance was too high to consider such a compound for further *in vivo* evaluation. Phosphonate **18c** also exhibited high binding to plasma proteins (95%) as expected for such an acidic compound. Plasma protein binding of prodrug **50a** was not possible to determine due to its low plasma stability.

**Table 5:** Metabolic Stability and Plasma Protein Binding Values of Compounds **18c** and **50a**

compound	plasma half-life [min]			liver microsomes Cl_int_ [μl/min/mg]			liver S9 fraction Cl_int_ [μl/min/mg]			PPB [%]
species	mouse	rat	human	mouse	rat	human	mouse	rat	human	human
**18c**	>120	>120	>120	2^a^	2^a^	3^a^	1^a^	2^a^	1^a^	95
**50a**	1.6	1.6	9.4	979	805	122	431	159	138	ND^b^

a Parameter should be considered as approximate due to the high stability of the compound.

b ND—not determined due to the low plasma stability of the compound.

**Figure 5:**
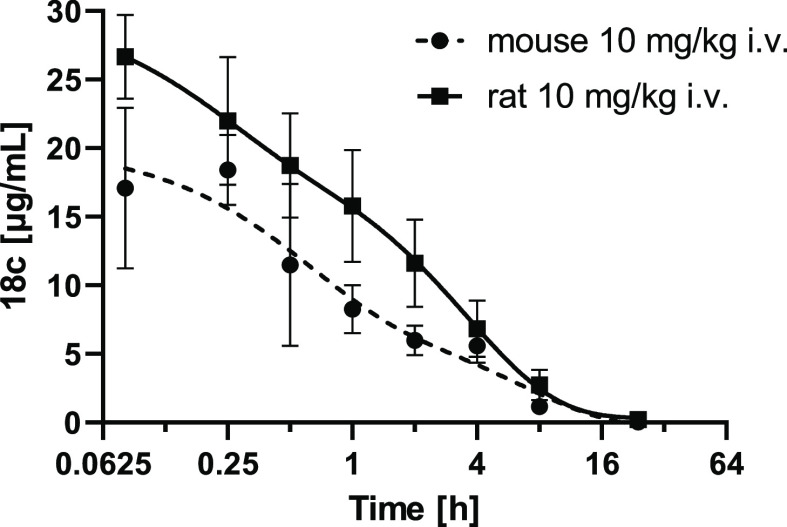
PK profile of compound **18c** in mice and rats.

Compound **18c** was then subjected to a pilot *in vivo* pharmacokinetic (PK) study in mice and rats. The PK profile was studied after intravenous (IV) administration of 10 mg/kg dose (Figure 5, Table 6). Based on the compound’s elimination half-lifes of 0.92 and 1.60 h in mouse and rat, respectively, sufficient systemic exposure can be expected upon IV administration.

**Table 6:** Pharmacokinetic Values for Compound **18c** in Mice and Rats Following 10 mg/Kg IV Administration

species	AUC [μg·h/mL]	*T*_max_ [h]	*C*_max_ [μg/ml]	*T*_1/2_ [h]
mouse	53.4	0.25	18.4	0.92
rat	92.7	0.08	25.3	1.6

### Crystallography

#### Crystal Structure of hPNP in Complex with Inhibitors **18c**, **45b**, **45i**, **45n**, and **45q**

To obtain information on the binding pose and interacting residues, crystal structures of recombinant hPNP in complex with inhibitors **18c**, **45b**, **45q**, **45i**, and **45n** were determined and refined to high resolution (Table S1). In all structures, inhibitors were modeled into well-defined electron density maps with full occupancy in the structure. A detailed description of X-ray structures is summarized in the Supporting Information.

Overall, the binding pose of inhibitors is comparable to immucilin H (ImmH),^51^ a PNP inhibitor currently approved in some countries for the treatment of peripheral T-cell lymphoma.^43^ Phosphonate moiety of compounds mimics the position of the phosphate ion in ImmH cocrystal structures and the position of the purine moiety is also conserved. A major difference is in the position and interactions of the central sugar moiety in ImmH vs phenyl moiety in our compounds (Figure S3).

**Figure 6:**
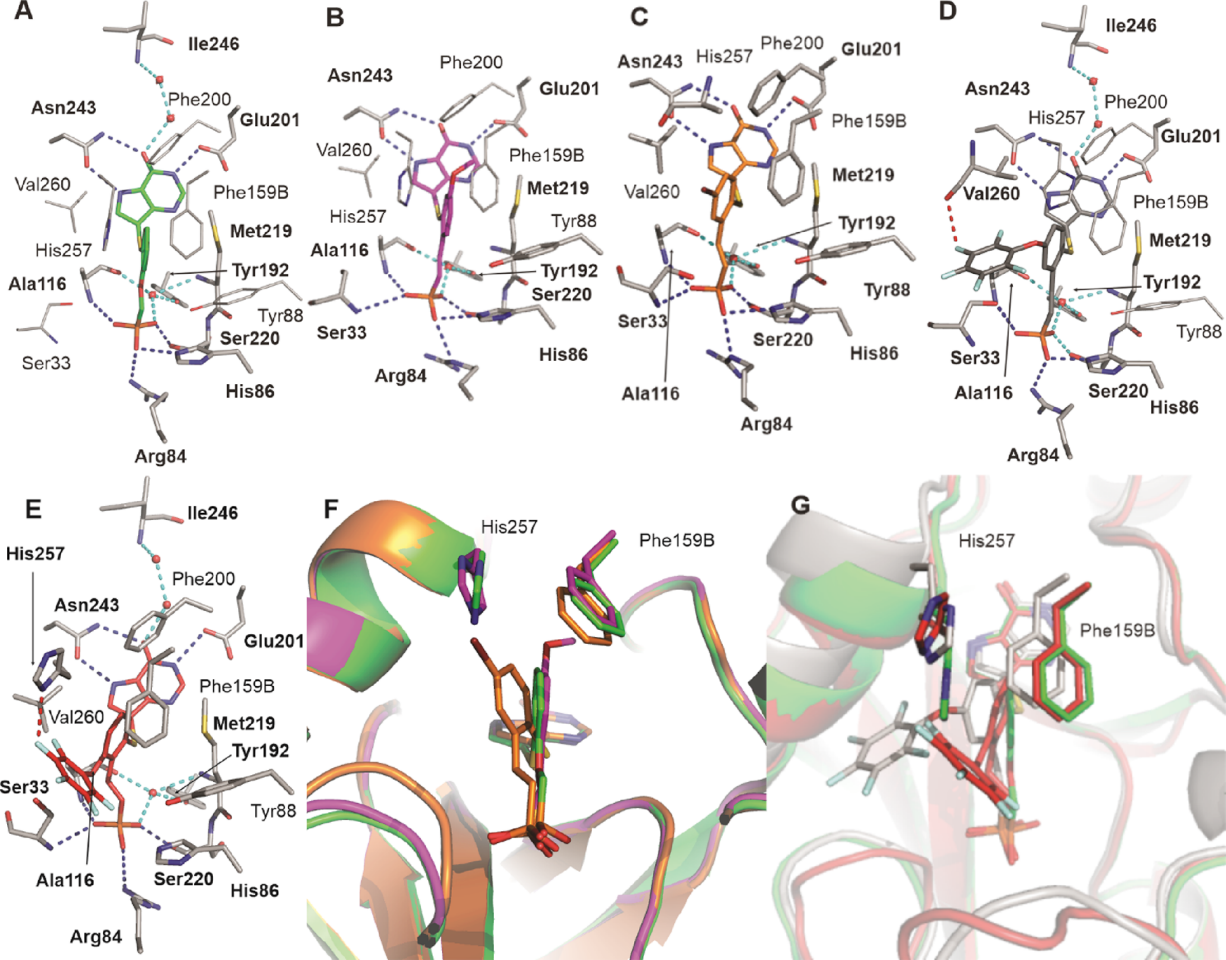
hPNP in complex with compounds **18c**, PDB 7ZSL (A), **45b**, PDB 7ZSM (B), **45q**, PDB 7ZSN, (C) **45i**, PDB 7ZSO (D), and **45n**, PDB 7ZSP (E). Hydrogen-bond forming residues are shown as sticks and highlighted in bold; other interacting residues are shown as lines and labeled. Protein carbon atoms are shown with gray color; inhibitor carbons are shown in green (A), magenta (B), orange(C), gray (D), and red (E) color, while nitrogen atoms are blue, phosphorus atoms are orange, sulfur is yellow, and oxygen is red. Direct hydrogen bonds are shown as blue-dashed lines, water-mediated—cyan-dashed lines, and halogen bonds as red-dashed lines. In panels F and G, protein carbons are shown in the same color as inhibitors, and highlighted residues are shown as sticks and labeled. Panel F shows overlay of hPNP in complex with **18c** (green), **45b** (magenta), and **45q** (orange). Panel G shows an overlay of hPNP in the complex with **18c** (green), **45i** (gray), and **45n** (red).

All five inhibitors bind to hPNP in a similar way, i.e., through seven direct hydrogen bonds and four water-mediated hydrogen bonds as well as numerous hydrophobic interactions. Purine moiety forms three direct hydrogen bonds with the Glu201 sidechain, two bonds with sidechain Asn243, and two-waters-mediated hydrogen bonds with the main chain of Ile246. Central phenyl moiety is located in the pocket formed by Phe200, His257, and Phe159 provided by the neighboring molecule within the trimer. The phosphonate moiety of the inhibitor forms direct hydrogen bonds with side chains of Ser33 (except in **18c**), Arg84, His86, and three water-mediated hydrogen bonds with Ala116, Tyr192, and Met219. Other residues that participate in the formation of the binding interface and are within the distance typical for van der Waals interactions are Gly32, Tyr88, Asn115, Ala117, Gly118, Val217, Gly218, Th242, Ala255, and Val260 (Figure 6: panels A–E).

Differences in the residues interacting with different inhibitors are minor and are the result of unique interactions of various substituents on the central phenyl moiety. The methoxy group at position C5 of phenyl moiety of **45b** interacts with both Phe200 and Phe159 from molecule B. Bromide substitution at position C4 in **45q** interacts with His257, Val260, and Leu261 of the helix_257–267_ (residues 257–267 are disordered in apoenzyme and form a helical structure, which elongates C-terminal helix upon substrate/inhibitor binding so this region will be referred to as helix_257–267_), while central phenyl moiety interacts with Val260. Bulky, fluorinated-phenyl substituent at position C4 of **45i** interacts with helix_257–267_ by forming a halogen bond with the carbonyl group of the Val260 main chain as well as van der Waals and other interactions with other residues in the helix such as Leu261 and Gly264. Similarly, fluorinated phenyl-ring substitution at position C5 in **45n** interacts with helix_257–267_ but forms a halogen bond with the side chain of His257.

To decipher structural reasons for different affinities of individual inhibitors, structure hPNP in complex with **18c** can be used as a control to which the other four inhibitors are compared (Figure 6F,G). Generally, the position of purine and phosphonate moieties in the hPNP binding site is conserved across all five inhibitors. Each moiety binds to the active site through conserved direct and water-mediated hydrogen bonds. Central phenyl moiety does not form any polar interactions but is positioned in the active site due to hydrophobic interactions as well as the overall shape of that region of the active site. There is a certain level of freedom in the position of the moiety that shifts depending on the substituents. This is allowed by the flexibility of the linker connecting central phenyl to phosphonate moiety. Inhibitor **45b** with the methoxy group substitution at phenyl position C5 has a 6-fold decrease in affinity toward hPNP. Structural analysis however shows no major differences in the structure and interactions in the active site, except for minor changes in the position of His257, Phe200, and PheB159 (Figure 6F). The methoxy group is oriented toward the subunit-binding interface, and we might speculate that unfavorable interactions and/or clashes with this rigid part of the active site can account for decreased affinity. Bromide substitution at position C4 of **45q** leads to a 2–3-fold decrease in affinity toward hPNP. The presence of a bromide substituent that interacts with helix_257–267_ causes a shift in the position of the central phenyl moiety (Figure 6C) and the substituent points toward helix_257–267_. No halogen bond interaction of bromine was detected in the crystal structure; the substitution pose however forces rearrangement of the His257 sidechain, whose position is disordered and cannot be unambiguously modeled based on the electron density map.

The addition of bulky substituents at phenyl positions C4 and C5 in **45i** and **45n** both lead to a 2-fold decrease in affinity toward hPNP, compared to **18c**. Compounds **45i** and **45n**, however, have quite distinct poses within the active site: major differences between the position of the central phenyl moiety as well as substituents are observed (Figure 6F,G). While both bulky substituents extend toward the helix_257–267_, their poses are quite different, and each makes different interactions with helix_257–267_ residues (Figure 6D,E). The structural explanation for comparable affinities of **45i** and **45n** is the flexibility of the phenyl-moiety binding pocket that enables to accommodate moiety shifts and by the substantial shift of helix_257–267_ to adjust to the position of the bulky substituents at position C4 (Figure 6G).

#### Crystal Structure of MtPNP in Complex with **18c**, **45b**, and **45q**

To obtain information on the binding pose and interacting residues, crystal structures of recombinant *Mt*PNP in complex with inhibitors **18c**, **45b**, and **45q** were determined and refined to high resolution (Table S1). The overall description of crystal structures is in the Supporting Information.

**Figure 7:**
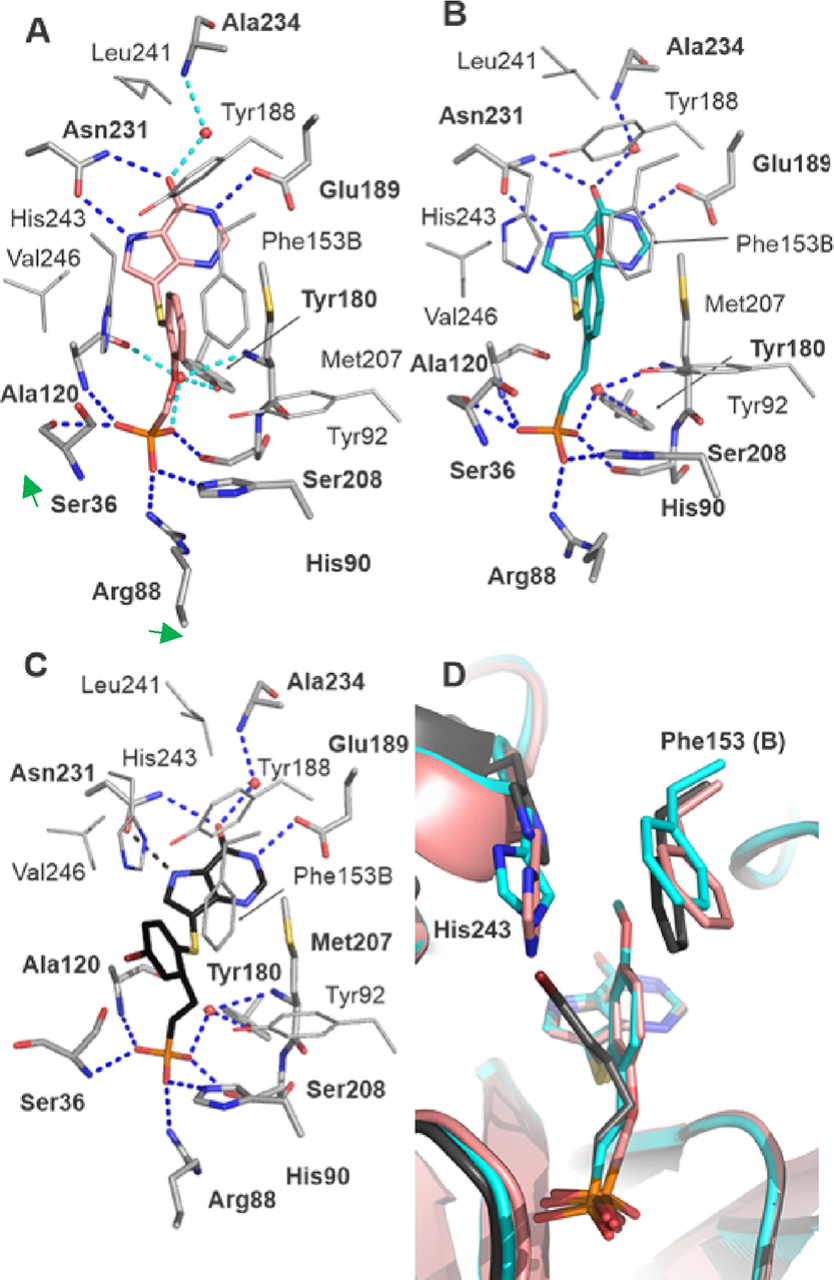
*Mt*PNP in complex with **18c**, PDB 7ZSQ (A), **45b**, PDB 8C25 (B), and **45q**, PDB 7ZSR (C). Carbon atoms are shown in salmon red (A), cyan (B), and black (C). Oxygen atoms are red, nitrogen—blue, sulfur—yellow, and phosphorus—orange. Residues that form direct hydrogen bonds are shown as sticks and labeled with bold fonts; other residues are shown as lines and labeled. Hydrogen bonds are represented by blue, and turquois dashed lines are for direct and water-mediated hydrogen bonds. The halogen bond is shown as a yellow dashed line. Panel D shows the overlay of all three ligands and the part of alpha helix 7 with His243 shown as sticks.

Inhibitors **18c**, **45b,** and **45q** bind to the active site of *Mt*PNP through eight direct and three water-mediated hydrogen bonds (Figure 7). Purine moiety forms direct hydrogen bonds with the sidechains of Glu189, two bonds with Asn231, and a water-mediated bond with the amine group of the Ala234 main chain. Central phenyl moiety interacts with a pocket formed by Tyr188, His243, and Phe153 sidechain belonging to the neighboring molecule within the enzyme trimer. Phosphonate moiety forms five potential direct hydrogen bonds with side chains of Arg88, His90, and Ser208, the main chain of Ser36 and Ala120, and two water-mediated hydrogen bonds with the main chain of Met207 and Tyr180 (Figure 7A–C). Other residues that participate in the formation of the active site (and are in proximity typical for van der Waals interactions) are Gly35, Asn119, Ala121, Pro186, Gly122, Tyr188, Val205, Gly206, Th230, Ala233, His243, and Val246.

Positions of purine and phosphonate moieties are identical in all three protein-inhibitor structures, and differences in inhibitor position are observed in the central phenyl moiety (Figure 7D). Compounds **45b** and **45q** have different substitutions at the central phenyl moiety. The methoxy group at position C5 in compound **45b** does not change the overall position of the inhibitor within the *Mt*PNP active site and only affects the position of the His243 sidechain (Figure 7D). Substitution by bromine at position C4 leads to changes in the orientation of the phenyl moiety as well as the shift of helix_243–253_ (same as in human PNP) to avoid sterically clashing with a side chain of His243. These structural changes do not affect inhibitor affinity toward *Mt*PNP since the IC_50_ values for each inhibitor are similar, leading to a conclusion that the active site is flexible and the movement of the helix_243–253_ allows the protein to accommodate inhibitors with different substitutions at positions C4 and C5 of the central phenyl moiety.

#### Comparison of Human PNP and MtPNP Complex Structures

We compared the structures of **18c**, **45b**, and **45q**, bound to both hPNP and *Mt*PNP to understand the structural basis for compound selectivity. Overall, active sites of hPNP and *Mt*PNP are similar with conserved residues occupying identical positions in both proteins. We found minor differences in inhibitor poses within the active sites (Figure S5) but uncovered substantial differences at the subunit-subunit interface in the vicinity of the active site (residues 133–165 in hPNP, residues 136–168 in *Mt*PNP, Figure S6). Structural variability and flexibility of this region affect the interaction with neighboring subunits and thus might contribute to the difference in affinity of the inhibitors toward *Mt*PNP compared to hPNP. Detailed description can be found in the Supporting Information (additional comparisons).

## Summary and Conclusion

We report the synthesis of a total of 36 potential inhibitors of purine nucleoside phosphorylase (PNP, the key purine salvage enzyme), where 30 are based on nucleoside phosphonates. Such PNP inhibitors have a great potential for the treatment of T-cell acute lymphoblastic leukemias (inhibition of hPNP) or infectious diseases (e.g., inhibition of PNP from various pathogens). All prepared compounds share the same feature, i.e., 9-deazahypoxanthine as a nucleobase, but they can be divided into three structural classes according to the moiety attached to the C9 position.

Compounds **7a–f** contain an aryl moiety without a phosphonate group, exhibit either none or poor (**7d** with IC_50_ = 0.787 μM) activity against hPNP and weak activity against *Mt*PNP (**7d** with IC_50_ = 0.364 μM), and, moreover, suffer from serious insolubility in both organic solvents and water.

Compounds **13a–b** are aliphatic acyclic nucleoside phosphonates, which are modest-to-good inhibitors of both hPNP (**13b** with IC_50_ = 0.49 μM) and *Mt*PNP (**13a** with IC_50_ = 0.64 μM).

The most extensive series is represented by 28 purposely designed PNP inhibitors, which contain an aryl moiety connected via variable linkers to the phosphonate group. Most compounds from this series are very potent inhibitors of both hPNP and *Mt*PNP with IC_50_ < 0.1 μM. The strongest inhibitors exhibited IC_50_ values as low as 19 nM for hPNP (compound **45a**) and 4 nM for *Mt*PNP (**45h**).

To our surprise, no inhibition of PNP from *Plasmodium falciparum* was observed with any of the prepared compounds at up to 10 μM. More importantly, most of the studied compounds exhibited selective cytotoxicity toward T-cell lymphoblasts (CCRF-CEM, MOLT-4, Jurkat) with CC_50_ values as low as 9, 13, and 10 nM, respectively, whereas no cytotoxic effect was observed on non-T-cells (HeLa S3, HL60) at up to 10 μM.

We also prepared four prodrugs derived from tool compound **18c**, namely, mixed phenoxy-amidates (ProTides) and bisamidates; however, the prodrugs did not improve the activity (and thus bioavailability) of the parent compound in the cell-based assays employed. For example, ProTides **50a**–**b** (with IC_50_ = 33–36 nM) had the same potency on CCRF-CEM cells as the parent compound **18c** (IC_50_ = 34 nM). A potential explanation of this phenomenon can be an efficient uptake of the parent compound **18c** (and all other phosphonate analogs from this study) *via* cell membrane transporters.

Furthermore, we described the first example of the PNP inhibitor (compound **45d**) exhibiting 60-fold selectivity for the pathogenic enzyme *Mt*PNP (IC_50_ = 29 nM) over hPNP (IC_50_ = 1.77 μM). These data strongly suggest the feasibility of future development of potential selective PNP inhibitor-based therapeutics against various pathogens.

Obtained results were supported by an extensive crystallographic study of eight complexes of prepared inhibitors with both hPNP (compounds **18c**, **45b**, **45i**, **45n**, and **45q**) and *Mt*PNP (compounds **18c**, **45b**, and **45q**). Thus, inhibitors **18c**, **45b**, and **45q** afforded crystal structures with both enzymes enabling direct comparison of ligand binding poses in both hPNP and *Mt*PNP. Crystallographic data showed significant flexibility of ligands’ biding poses depending on the position and size of substituents at the central phenyl moiety and even minor flexibility of the enzymes’ sidechains.

The tool compound **18c** also exhibited encouraging pharmacokinetic properties with excellent plasma stability. These results represent a solid ground for further targeted development of improved PNP inhibitors for the potential treatment of T-cell leukemias.

## Experiment and Methods

### Protein Crystallography

Recombinant proteins prepared by heterologous expression in *E. coli* were used for crystallization. Details of purification are in the Supporting Information. Samples for crystallization of hPNP were prepared by mixing hPNP, in a buffer containing 20 mM potassium sulfate, 200 mM NaCl, 1 mM tris(2-carboxyethyl)phosphine (TCEP), pH 7.5 at the concentration of 11.75 mg/mL, with inhibitors dissolved in water and DMSO to achieve final molar excess of inhibitor over protein 4 to 5. Samples were incubated on ice for 1 to 2 h and clarified by centrifugation. All crystallization experiments were performed at 18 °C. The crystals of hPNP/**18c**, hPNP/**45b**, hPNP/**45q**, and hPNP/**45n** were obtained using the sitting-drop vapor diffusion technique set by Orynx8 robot (Douglas Instruments) in Swissci 96-well 3-drop plates (Molecular Dimensions). The reservoir volume was 30 μL, and the drops were 300 nL in volume. For hPNP/**18c**, the reservoir contained 0.3 M magnesium chloride, 0.3 M calcium chloride, 20% w/v PEG 8000, 40% v/v ethylene glycol, 0.1 M MES/imidazole, and pH 6.0,^53^ and the drop consisted of 200 nL of protein, 80 nL of the precipitant solution, and 20 nL of 1 M ammonium sulfate (added as an additive). For hPNP/**45q**, the reservoir contained 2.00 M ammonium formate, 100 mM sodium acetate, 1 mM TEW, and pH 4.6.^54^ For hPNP/**45n**, the reservoir contained 15% w/v polyethylene glycol 8000, 500 mM lithium sulfate, 100 mM sodium acetate, and pH 4.6.^54^ Crystals appeared in 1–10 days, cryoprotected by 20–30% glycerol and flash-frozen in liquid nitrogen after 14 days. Crystals of hPNP/**45i** were prepared in a hanging drop using EasyXtal 15-Well Tool plates, with a 500 μL reservoir, which contained 1 M lithium sulfate, 100 mM tri-sodium citrate, and pH 5.4. Cubic crystals, dimensions 150 × 150 × 150 μm, appeared after 3 weeks and were harvested after 4 weeks. Crystals were cryoprotected with reservoir solutions supplemented with 20% glycerol prior to being frozen in liquid nitrogen.

Samples for crystallization of *Mt*PNP were prepared by mixing *Mt*PNP in a buffer containing 40 mM TRIS, 50 mM NaCl, pH 8.5, and at a concentration of 15 or 25 mg/mL with inhibitor dissolved in DMSO to achieve final molar excess of the inhibitor over proteins 3 to 5. Samples were incubated on ice for 2 h and clarified by centrifugation. All crystallization experiments were performed at 18 °C. The crystal of *Mt*PNP with **18c** was obtained using the sitting-drop vapor diffusion technique set by Orynx8 robot (Douglas Instruments) in Swissci 96-well 3-drop plates (Molecular Dimensions). The reservoir solution of 30 μL volume contained 0.2 M magnesium chloride, 30% v/v PEG400, 1 mM Tellurium-Centered Anderson–Evans Polyoxotungstate (TEW), 0.1 M HEPES pH 7.5,^54^ and drop volume 300 nL, made by mixing equal volumes of the sample and precipitate. Needle crystal, dimensions 300 × 30 × 30 μm appeared after 3 weeks and were harvested after 1 week. Crystals of *Mt*PNP in complex with **45b** and **45q** were obtained using the EasyXtal 15-Well Tool, hanging drop plates with reservoir volumes of 500 μL and 2 μL drops with protein:reservoir ratio 1:1. The *Mt*PNP/**45b** crystallization reservoir contained 2 M sodium chloride, 100 mM sodium acetate, and pH 4.6. Pyramid-shaped crystals grew in 2 days to the size of 100 × 100 × 100 μm were harvested after 2 days. Crystals of *Mt*PNP/**45q** were obtained using the reservoir containing 25 mM magnesium chloride, 100 mM TRIS pH 7.5, and 25% w/v PEG4000. Plate crystal dimensions 400 × 150 × 10 μm grew in 3 weeks. All crystals were cryoprotected with reservoir solutions supplemented with 20% glycerol prior to being frozen in liquid nitrogen.

Complete data sets were collected at 100 K at the beamline MX14.1 at the BESSY II electron storage ring operated by the Helmholtz-Zentrum Berlin, Germany.^55^ The dataset was processed using the program XDS.^56^ Crystal parameters and diffraction data collection statistics are summarized in Tables S1 and S2.

Crystal structures were solved using a molecular replacement method using program MolRep version 11.7.03.^57^ hPNP and *Mt*PNP monomers available under the code 3PHB (hPNP) and 1G2O (*Mt*PNP)^58^ were used as a search model. Model refinement was performed using REFMAC5 5.8.0267^59^ as part of the CCP4 package,^60^ in combination with manual refinement in Coot software version 0.9.4.1.^61^ The Molprobity server was used for model validation.^62^ All the figures representing structures were created using PyMOL [The PyMOL Molecular Graphics System, Schödinger, LLC]. Protein-ligand interactions were analyzed using LigPlot+,^63^ BIOVIA Discovery Studio, and PISA server.^52^ Information on refinement statistics is available in Tables S1 and S2 in the Supporting Information section.

### Biology

#### PNP Enzyme Inhibition Assay

To evaluate the inhibitory activity of the compounds toward PNP, recombinant PNP proteins (*h*PNP, *Pf*PNP, *Mt*PNP) were expressed in *E. coli*, purified by means of affinity chromatography (NiNTA column, Thermo Fisher Scientific, Waltham, USA), and stored in 20 mM phosphate buffer pH 7.4 containing 0.3 M NaCl in aliquots at −80 °C. All newly synthesized compounds were dissolved either in water or DMSO to yield 10 mM stock solutions. The compounds then underwent basic screening at 10 μM concentration, and in case that at least 50% inhibition was observed, the dose–response curve was generated to calculate the IC_50_ value (half-maximal inhibitory concentration). Forodesine (MedChemExpress, Monmouth Junction, USA) was used as a reference compound. The reaction mixture for PNP activity determination consisted of 1 mM *P_i_*, 200 μM [2,8-^3^H] inosine (ARC Inc., Saint Louis, USA), variable concentrations of the tested compound, 1 mM DTT, 0.2 μg/mL BSA, and 1.75 μg of PNP protein. The reaction was incubated for 10 min at 37 °C, and it was terminated by spotting a 2 μL aliquot of PEI-coated cellulose TLC plate (no. 105579, Merck Darmstadt, Germany). The plate was developed in *n*-butanol-acetic acid-water at a 10:1:3 ratio, let dry, and analyzed by means of a radio-TLC scanner RITA Star (Elysia-Raytest GmbH, Strauberhardt, Germany).

#### Cellular Cytotoxicity Assay

To evaluate the selectivity of the antitumor action of the compounds, three T-lymphoblastic cell lines (CCRF-CEM, Jurkat, MOLT-4) were employed as a surrogate for T-ALL disease. A promyelocytic leukemia cell line (HL-60) and two carcinoma-derived cell lines (HeLa, HepG2) were used to address off-target toxicity. All cell lines were from ATCC (Manassas, VA, USA). The cells were incubated in RPMI-1640 (suspension cells) or a DMEM (adherent cells) culture medium containing 10% FBS and 1% GlutaMax at 37 °C under the atmosphere containing 5% CO_2_. Experiments were performed on the cells between passage nos. 10 and 50. For the experiments, cells were seeded in 384-well transparent plates (Brand GmbH, Wertheim, Germany) at a concentration of 2000–10,000 cells per well and left to rest in an incubator overnight. On the next day, 10 μM deoxyguanosine (Sigma) and varying concentrations of the test compounds were added. The compounds were incubated with the cells in a CO_2_ incubator at 37 °C for 72 h after which XTT dye (Sigma-Aldrich, St. Louis, USA) was added. The absorbance signal at 450 nm was recorded after 1–4 h using a multimode plate reader. The signal of untreated cells was set to 100% cell viability. The effect of the compounds was expressed as IC_50_ values calculated by nonlinear regression using GraphPad Prism v. 5.04 (GraphPad Software, San Diego, USA).

Peripheral blood mononuclear cells (PBMC) were isolated from healthy donors (with their informed consent) by centrifugation of the buffy-coats through Ficoll gradient. The T-cell-enriched fraction of white blood cells was obtained with the use of RosetteSep immunodensity separation technology according to the manufacturer’s instructions (StemCell Technologies, Vancouver, Canada). The purity of the T-cells was ≥98% as determined by flow cytometry (CD3+ cells). Immediately after the isolation, cells were seeded in the 384-well white plates (Thermo Fisher Scientific, Waltham, USA) at a concentration of 50,000 cells per well, mimicking their density in whole blood. On the next day, cells were treated with the test compounds and deoxyguanosine at a concentration of 50 and 10 μM, respectively. The cells were then incubated at 37 °C and 5% CO_2_ for 72 h after which CellTiter-Glo detection reagent (Promega, Madison, USA) was added. The plate was left on a shaker (350 rpm) for 20 min at rt. Luminescence was measured by a multimode plate reader Cytation 3 (BioTek Instruments Inc., Winooski, USA). The signal of the compound-treated cells was compared to the value of the untreated control, which was set to 100% viability.

#### *In Vitro* ADME Assays

Microsomal stability assay was performed using the 0.5 mg/mL human pooled liver microsomal preparation (Thermo Scientific) and 10 μM compounds in 90 mM TRIS-Cl buffer pH 7.4 containing 2 mM NADPH and 2 mM MgCl2 for 10, 30, and 45 min at 37 °C. The reactions were terminated by the addition of four volumes of ice-cold methanol, mixed vigorously, and left at −20 °C for 1 h. After that, the samples were centrifuged and the supernatants were analyzed by means of ECHO-MS System (Sciex, Framingham, MA, USA). Zero time points were prepared by adding ice-cold methanol to the mixture of the compound with cofactors prior to the addition of microsomes. The microsomal half-lives (t1/2) were calculated using the equation t1/2 = 0.693/*k*, where *k* is the slope found in the linear fit of the natural logarithm of the fraction remaining of the parent compound vs incubation time. The intrinsic clearance (CLint) was calculated using the following formula:
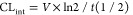
where *V* = incubation volume per milligram of microsomal protein (μL/mg) and *t*1/2 = microsomal half-life.

To determine the plasma stability of the compounds, 5 μM of these were incubated with human pooled plasma from 50 donors (Biowest) for 120 and 240 min at 37 °C. The reactions were terminated by adding four volumes of ice-cold methanol; the samples were then mixed vigorously and left at −20 °C for 1 h. After that, the samples were centrifuged, and the supernatant was analyzed by means of an ECHO-MS System (Sciex). Zero time points were prepared by adding ice-cold methanol to the compound prior to the addition of the plasma.

The binding of the tested compounds to plasmatic proteins was assessed using the equilibrium dialysis method with single-use RED device with 8K MWCO (Thermo Scientific) according to the manufacturer’s instructions. Briefly, 100 μL of samples prepared by spiking the undiluted human plasma (Biowest) with test compounds to a concentration of 100 μM was added to the sample chamber of the device and dialyzed against 300 μL of PBS in the buffer chamber for 4 h, 37 °C, on the orbital shaker (300 rpm). After that, 50 μL volumes from both sample and buffer chambers were transferred to microtubes. Then, 50 μL of the plasma was added to the buffer sample, and 50 μL of PBS was added to the collected plasma samples (to unify the matrices). The proteins in the samples were precipitated with 200 μL of MeOH after which samples were centrifuged 20,000×*g* for 10 min. The supernatants were analyzed using LC/MS (Sciex 6500+ system), and the percentage of the compound bound was calculated using the following formulas: % free = (concentration buffer chamber/concentration plasma chamber) × 100%. % bound = 100% – % free. Assay performance was quality-checked using verapamil (highly bound) and atenolol (poorly bound) as reference compounds.

#### Kinetic Solubility

Stock solutions (10 mM) of the test compounds in DMSO were used to prepare dilutions to a theoretical concentration of 400 μM in 0.1 M citrate buffer (pH 3.0), 0.1 M phosphate-buffered saline (pH 7.0), 0.1 M glycine-sodium hydroxide buffer (pH 9), and fasted state simulated intestinal fluid (FaSSIF, pH 6.5) with 2% final DMSO in duplicates. The experimental compound dilutions were further allowed to equilibrate at 25 °C on a thermostatic shaker for 2 h and then filtered through HTS filter plates using a vacuum manifold. The filtrates of test compounds were diluted 2-fold with acetonitrile with 2% DMSO before measuring. Calibration curves in 50% ACN/appropriate buffer were prepared up to 200 μM, with 2% DMSO. Each sample (200 μL) was transferred to a 96-well plate and measured in the 200–550 nm range.

#### Pharmacokinetics

C57BL6/N male mice were housed on a 12 h light and 12 h dark cycle with room temperature maintained at 22 ± 3 °C and relative humidity at 50 ± 20%. Animals were fasted for 4 h before dosing. Water was provided *ad libitum* throughout the study. The animals were dosed with 10 mg/kg JS-196 formulated in 40% polyethylene glycol 300 + 60% water by intravenous injection. Blood samples were taken via retro-orbital venous sinus under anesthesia at 5, 15, and 30 min and 1, 2, 4, and 8 after dosing and processed for analysis. All samples were stored at −70 °C until analysis for side-by-side comparison. Three volumes of 80% acetonitrile were added to one volume of plasma to precipitate proteins. Samples were centrifuged (20,000*g* for 10 min), and supernatants were subjected to the analysis by LC–MS/MS. Calibration standards were made by preparation of a 1 mg/mL stock solution in 80% acetonitrile. LC separation was done on Synergi 4um Fusion 50 × 2 mm column (Phenomenex) using a water/acetonitrile gradient with 0.1% formic acid starting from 5 to 90% for 8 min. MS/MS analysis was performed on QTRAP 5500+ (Sciex) utilizing multiple reactions monitoring 352.0–>133.9 and 352.0–>168.9 for JS-196 detection. The data analysis was based on the plasma concentrations, which were measured as described above to determine a concentration vs time profile. The area under the plasma concentration vs time curve was calculated using the linear trapezoidal method. Figures and analysis were generated in GraphPad Prism.

A pharmacokinetic study in rats was performed by a CRO according to their SOPs and permissions.

#### Ethics Statement

This study was conducted according to the principles expressed in the Declaration of Helsinki. Human blood for PBMC isolation was obtained from healthy individuals upon their informed consent following the approval of the Ethical Committee of the Institute of Hematology and Blood Transfusion No. 13/06/2012. The animal studies were ethically reviewed and performed in accordance with European directive 2010/63/EU and were approved by the Czech Central Commission for Animal Welfare project of experiments 34/2019/CZ.

### Chemistry

#### General Information

Unless otherwise stated, solvents were evaporated at 40 °C/2 kPa and prepared compounds were dried at 30 °C at 2 kPa. Starting compounds and reagents were purchased from commercial suppliers (Sigma-Aldrich, Fluorochem, Acros Organics, Carbosynth, TCI) and used without further purification or were prepared according to the published procedures.

Diethyl ether, tetrahydrofuran, dioxane, and acetonitrile were dried by activated neutral alumina (drysphere). Dimethylformamide was dried by activated molecular sieves (3 Å). Other dry solvents were purchased from commercial suppliers (Sigma-Aldrich, Acros Organics). Triethylamine was dried with potassium hydroxide under an argon atmosphere in an amber flask sealed with a septum.

Microwave experiments were performed in 10 or 30 mL vials with a CEM Discover (Explorer) microwave reactor operating at a frequency of 2.45 GHz with continuous irradiation power from 0 to 300 W.

Large-scale reactions were carried out in a Syrris Atlas Potassium system with a 2, 1, or 0.5 L jacket reactor coupled with a Julabo FP50-HL Refrigerated/Heating Circulator.

Analytical TLC was performed on silica gel precoated aluminum plates with a fluorescent indicator (Merck 60F254). Flash column chromatography was carried out by Teledyne ISCO CombiFlash Rf200. Various types of columns were used: (a) Teledyne ISCO columns RediSepRf HP Silica GOLD in sizes 12, 40, 80, and 120 g; (b) Teledyne ISCO columns RediSepRf HP C18 Aq GOLD in sizes 50 and 100 g; (c) column Chomabond Flash DL 40, DL 80, DL 120, and DL 200, filled with FLUKA silica gel 60.

Preparative HPLC purifications were performed on a Waters Delta 600 chromatography system with columns packed with C18 reversed-phase resin (Phenomenex Gemini 10 μm 21 × 250 mm, Phenomenex Gemini 5 μm 21 × 250 mm, Phenomenex Luna 10 μm 21 × 250 mm) using gradient H2O/MeOH as an eluent.

Mass spectra, UV spectra, and purity of compounds were measured on a Waters UPLC-MS system consisting of a Waters UPLC H-Class Core System (column Waters Acquity UPLC BEH C18 1.7 mm, 2.1 × 100 mm), a Waters Acquity UPLC PDA detector, and a mass spectrometer Waters SQD2. The universal LC method was used (eluent H2O/CH3CN, gradient 0–100%, run length 7 min) and MS method (ESI+ and/or ESI-, cone voltage = 30 V, mass detector range 100–1000 Da). All the compounds are >95% pure, except for **7b** (>93%), **18a** (>93%), **21** (>91%), **45e** (>93%), and **50b** (>93%).

High-resolution mass spectra were measured on an LTQ Orbitrap XL spectrometer (Thermo Fisher Scientific).

NMR spectra were recorded on Bruker Avance 400 or 500 spectrometers referenced to the residual solvent signal or a specified additive. Assignments of NMR signals are stated in the Supporting Information and are based on heteronuclear correlation experiments HSQC, HMBC, COSY, and NOESY in specific cases.

Dowex 50D resin was turned to the Na+ cycle by treatment of Dowex D50 resin in the H+ cycle with 1 M NaOH aq solution, followed by washing with water to neutral pH.

#### Synthesis

##### 4-(Benzyloxy)-5*H*-pyrrolo[3,2-*d*]pyrimidine (**2**)

The jacket reactor (2 L) was flushed with nitrogen and charged with benzyl alcohol (1 L), and the system was set to retain a temperature of 20 °C. Sodium metal (22.5 g, 977 mmol, 1.5 equiv) was added in portions, and the mixture was stirred for 20 h under the small flow of nitrogen. The mixture was then heated at 80 °C for 1 h, and then, it was cooled back to 20 °C. 4-Chloro-5*H*-pyrrolo[3,2-*d*]pyrimidine (**1**) (100 g, 651 mmol, 1 equiv) was charged, and the mixture was stirred at 80 °C until complete conversion was achieved (ca. 4 h). The mixture was cooled to 5 °C, diluted with water (400 mL), and pH was adjusted to 7 with 3 M HCl (aq) (ca. 100–130 mL). The mixture was heated to 20 °C, extracted with chloroform (3 × 400 mL), and washed with brine (500 mL). The mixture was concentrated, and benzyl alcohol was evaporated at high vacuum (<1 mBar) at ca. 90 °C. The solid was filtered through a short pad of silica gel (600 g) with an eluent (100% of chloroform, then chloroform with 5% methanol). Solvents were evaporated, the residue was dissolved in a refluxed mixture of ethyl acetate/methanol (1:1), and the solution was cooled to 30 °C. Antisolvent pentane (same volume as the mixture) was slowly added, and the mixture was cooled to −20 °C within 3 h. Then, it was stirred for 20 h, during which the product crystallized. Crystals were collected, washed with pentane, and dried, yielding 141 g (96%) of 4-(benzyloxy)-5*H*-pyrrolo[3,2-*d*]pyrimidine (**2**) as white crystals. **^1^H NMR** (401 MHz, chloroform-*d*) δ 9.33 (s, 1H), 8.58 (s, 1H), 7.49–7.43 (m, 2H), 7.41 (m, 1H), 7.38–7.32 (m, 2H), 7.35 (s, 1H), 6.67 (dd, *J* = 3.2, 2.1 Hz, 1H), 5.59 (s, 2H). **^13^C NMR** (101 MHz, chloroform-*d*) δ 155.46, 150.55, 150.11, 136.22, 128.76–128.28 (m), 115.14, 103.31, 67.94. **MS** (ESI-QMS) *m*/*z* = 226.1 [M + H]^+^.

##### 4-(Benzyloxy)-7-iodo-5-((2-(trimethylsilyl)ethoxy)methyl)-5*H*-pyrrolo[3,2-*d*]pyrimidine (**4**)

The jacket reactor (1 L) was flushed with nitrogen, 4-(benzyloxy)-5*H*-pyrrolo[3,2-*d*]pyrimidine (**2**) (40 g, 178 mmol, 1 equiv) was dissolved in tetrahydrofuran (500 mL), and *N*-iodosuccinimide (44 g, 195 mmol, 1.1 equiv) was added to the mixture. The mixture was stirred at room temperature for 1 h, during which the product crystallized. Crystals were collected, washed with tetrahydrofuran, and dried, yielding 56 g (90%) of the crude 4-(benzyloxy)-7-iodo-5*H*-pyrrolo[3,2-*d*]pyrimidine (**3**) as a white solid, which was used in the next step without further purification and characterization. The solid (56 g, 159 mmol, 1 equiv) was charged into the jacket reactor (2 L), which was flushed with nitrogen, and dry dimethylformamide (560 mL) was added. The mixture was cooled to −5 °C and sodium hydride (8 g, 199 mmol, 1.25 equiv) was added portion-wise under a small flow of nitrogen keeping the temperature under 5 °C. The mixture was stirred at 20 °C for 1 h. 2-(Trimethylsilyl)ethoxymethyl chloride (35.3 mL, 199 mmol, 1.25 equiv) was added dropwise, followed by stirring at 20 °C for 1 h. The reaction was quenched with a half-saturated aqueous solution of NH_4_Cl (560 mL), extracted with ethyl acetate (3 × 300 mL), washed with brine (1 × 500 mL), and dried with MgSO_4_. Solvents were evaporated, and the solid was filtered through a short pad of silica gel (300 g) with an eluent (100% of chloroform, then chloroform with 1% methanol). Solvents were evaporated, and the residue was just dissolved in refluxed acetonitrile. The solution was slowly cooled to 20 °C within 3 h, during which the product crystallized. Crystals were collected, washed with acetonitrile, and dried, yielding 66 g (86%) of the title compound as white flakes. **^1^H NMR** (401 MHz, chloroform-*d*) δ 8.65 (s, 1H), 7.51 (s, 1H), 7.51–7.45 (m, 2H), 7.44–7.31 (m, 3H), 5.63 (s, 2H), 5.62 (s, 2H), 3.73–3.17 (m, 2H), 0.97–0.65 (m, 2H), −0.09 (s, 9H). **^13^C NMR** (101 MHz, chloroform-*d*) δ 155.90, 151.86, 151.05, 136.44, 136.11, 128.80, 128.52, 128.33, 115.87, 77.84, 68.55, 66.30, 59.18, 17.80, −1.34. **MS** (ESI-QMS) *m*/*z*: [M + H]^+^ calcd for C_19_H_25_IN_3_O_2_Si, 482.1; found, 482.2.

#### General Procedures for Compounds **6a–f**

Compound **4** (200 mg, 0.415 mmol, 1.0 equiv), copper(I) iodide (8 mg, 0.042 mmol, 0.1 equiv), and 1,10-phenanthroline (15 mg, 0.083 mmol, 0.2 equiv) were charged into a microwave vial. The vial was flushed with argon, and dry toluene (2 mL) with triethylamine (87 μL, 0.623 mmol, 1.5 equiv) was added subsequently. A thiophenol (0.499 mmol, 1.2 equiv) was added as the last component after which the solution turned into a dark-red color. The vial was sealed and inserted into the microwave reactor for 2 h at 120 °C. The dark-brown reaction mixture was dissolved in chloroform, washed with a half-saturated aqueous solution of NaHCO_3_ (2×), 1 M HCl (aq) (2×), brine (1×), dried with MgSO_4_, filtered, and evaporated. Purification by flash chromatography on silica gel (cyclohexane to 15% of ethyl acetate modified with 10% of methanol) afforded the title compound as a white-off oil.

##### 4-(Benzyloxy)-7-(phenylthio)-5-((2-(trimethylsilyl)ethoxy)methyl)-5*H*-pyrrolo[3,2-*d*]pyrimidine (**6a**)

The general procedure afforded 150 mg (77%) of the title compound. **^1^H NMR** (401 MHz, chloroform-*d*) δ 8.68 (s, 1H), 7.68 (s, 1H), 7.55–7.47 (m, 2H), 7.44–7.33 (m, 3H), 7.24–7.14 (m, 4H), 7.12–7.03 (m, 1H), 5.67 (s, 4H), 3.47 (t, *J* = 8.0 Hz, 2H), 0.84 (t, *J* = 8.3, 7.8 Hz, 2H), −0.09 (s, 9H). **^13^C NMR** (101 MHz, chloroform-*d*) δ 156.56, 150.86, 150.75, 138.85, 137.65, 135.85, 128.99, 128.83, 128.64, 128.44, 127.45, 125.83, 116.31, 105.13, 78.07, 68.88, 66.52, 17.82, −1.35. **MS** (ESI-QMS) *m*/*z*: [M + H]^+^ calcd for C_25_H_30_N_3_O_2_SSi, 464.2; found, 464.3.

##### 4-(Benzyloxy)-7-((2-methyl)phenylthio)-5-((2-(trimethylsilyl)ethoxy)methyl)-5*H*-pyrrolo[3,2-*d*]pyrimidine (**6b**)

The general procedure afforded 160 mg (80%) of the title compound. **^1^H NMR** (401 MHz, chloroform-*d*) δ 8.64 (s, 1H), 7.59 (s, 1H), 7.55–7.47 (m, 2H), 7.44–7.32 (m, 3H), 7.10 (d, *J* = 7.3 Hz, 1H), 6.98 (ddd, *J* = 7.4, 1.4 Hz, 1H), 6.91 (ddd, *J* = 7.7, 1.8 Hz, 1H), 6.73 (dd, *J* = 7.8, 1.4 Hz, 1H), 5.66 (s, 4H), 3.48 (t, *J* = 7.9 Hz, 2H), 2.48 (s, 3H), 0.84 (t, *J* = 8.1 Hz, 2H), −0.08 (s, 9H). **^13^C NMR** (101 MHz, chloroform-*d*) δ 156.15, 151.98, 151.20, 138.37, 137.04, 136.03, 134.96, 130.02, 128.66, 128.37, 128.23, 126.33, 125.73, 125.07, 116.29, 104.15, 77.90, 68.33, 66.23, 20.00, 17.70, −1.44. **MS** (ESI-QMS) *m*/*z*: [M + H]^+^ calcd for C_26_H_32_N_3_O_2_SSi, 478.2; found, 478.3.

##### 4-(Benzyloxy)-7-((2-bromo)phenylthio)-5-((2-(trimethylsilyl)ethoxy)methyl)-5*H*-pyrrolo[3,2-*d*]pyrimidine (**6c**)

The general procedure afforded 191 mg (85%) of the title compound. **^1^H NMR** (401 MHz, chloroform-*d*) δ 8.59 (s, 1H), 7.62 (s, 1H), 7.51 (d, *J* = 6.9 Hz, 2H), 7.44–7.22 (m, 4H), 6.94 (dd, *J* = 6.6 Hz, 1H), 6.83 (dd, *J* = 6.6 Hz, 1H), 6.59 (d, *J* = 8.0 Hz, 1H), 5.64 (s, 4H), 3.47 (t, *J* = 7.9 Hz, 2H), 0.83 (t, *J* = 8.0 Hz, 2H), −0.10 (s, 9H). **^13^C NMR** (101 MHz, chloroform-*d*) δ 156.09, 151.84, 151.30, 139.28, 138.86, 135.86, 132.47, 128.54, 128.27, 128.13, 127.44, 126.27, 125.90, 119.85, 116.26, 102.97, 77.93, 68.26, 66.20, 17.59, −1.51. **MS** (ESI-QMS) *m*/*z*: [M + H]^+^ calcd for C_25_H_29_BrN_3_O_2_SSi, 542.1; found, 542.2.

##### 4-(Benzyloxy)-7-((2-hydroxy)phenylthio)-5-((2-(trimethylsilyl)ethoxy)methyl)-5*H*-pyrrolo[3,2-*d*]pyrimidine (**6d**)

The general procedure afforded 132 mg (66%) of the title compound. **^1^H NMR** (401 MHz, chloroform-*d*) δ 8.66 (s, 1H), 7.60 (s, 1H), 7.59 (dd, *J* = 7.8, 1.6 Hz, 1H), 7.48 (dd, *J* = 7.9, 1.6 Hz, 2H), 7.44–7.31 (m, 3H), 7.21 (ddd, *J* = 8.2, 7.3, 1.7 Hz, 1H), 7.00 (dd, *J* = 8.1, 1.4 Hz, 1H), 6.79 (ddd, *J* = 7.5, 1.4 Hz, 1H), 5.63 (s, 2H), 5.58 (s, 2H), 3.43 (t, *J* = 8.0 Hz, 2H), 0.81 (t, *J* = 8.2 Hz, 2H), −0.10 (s, 9H). **^13^C NMR** (101 MHz, chloroform-*d*) δ 158.52, 156.09, 150.89, 150.25, 136.69, 136.61, 135.81, 131.43, 128.73, 128.51, 128.26, 121.32, 120.64, 118.48, 115.83, 108.65, 77.74, 68.69, 66.36, 17.70, −1.41. **MS** (ESI-QMS) *m*/*z*: [M + H]^+^ calcd for C_25_H_30_N_3_O_3_SSi, 480.2; found, 480.2.

##### 4-(Benzyloxy)-7-((2-hydroxymethyl)phenylthio)-5-((2-(trimethylsilyl)ethoxy)methyl)-5*H*-pyrrolo[3,2-*d*]pyrimidine (**6e**)

The general procedure afforded 130 mg (64%) of the title compound. **^1^H NMR** (401 MHz, chloroform-*d*) δ 8.51 (s, 1H), 7.70 (s, 1H), 7.47 (dd, *J* = 7.9, 1.7 Hz, 2H), 7.42 (dd, *J* = 7.6, 1.5 Hz, 1H), 7.40–7.32 (m, 3H), 7.27 (dd, *J* = 7.8, 1.4 Hz, 1H), 7.17 (ddd, *J* = 7.4, 1.4 Hz, 1H), 7.08 (ddd, *J* = 7.5, 1.6 Hz, 1H), 5.63 (s, 2H), 5.60 (s, 2H), 5.03 (s, 2H), 3.45 (t, *J* = 8.1 Hz, 2H), 1.42 (s, 1H), 0.83 (t, *J* = 8.1 Hz, 2H), −0.08 (s, 9H). **^13^C NMR** (101 MHz, chloroform-*d*) δ 156.18, 150.62, 150.50, 142.31, 137.38, 135.83, 134.76, 133.09, 130.01, 128.69, 128.57, 128.47, 128.27, 128.02, 116.16, 107.07, 77.82, 68.56, 66.31, 63.56, 17.72, −1.40. **MS** (ESI-QMS) *m*/*z*: [M + H]^+^ calcd for C_26_H_32_N_3_O_3_SSi, 494.2; found, 494.3.

##### 4-(Benzyloxy)-7-((3-bromo)phenylthio)-5-((2-(trimethylsilyl)ethoxy)methyl)-5*H*-pyrrolo[3,2-*d*]pyrimidine (**6f**)

The general procedure afforded 82 mg (89%) of the title compound. **^1^H NMR** (401 MHz, chloroform-*d*) δ 8.63 (s, 1H), 7.66 (s, 1H), 7.56–7.48 (m, 2H), 7.43–7.33 (m, 3H), 7.22 (dd, *J* = 1.8 Hz, 1H), 7.16 (ddd, *J* = 7.7, 1.9, 1.1 Hz, 1H), 7.05 (ddd, *J* = 7.9, 1.8, 1.2 Hz, 1H), 6.99 (dd, *J* = 7.8 Hz, 1H), 5.66 (s, 2H), 5.64 (s, 2H), 3.48 (t, *J* = 7.9 Hz, 2H), 0.84 (t, *J* = 8.1 Hz, 2H), −0.09 (s, 9H). **^13^C NMR** (101 MHz, chloroform-*d*) δ 156.22, 151.63, 151.36, 140.55, 138.64, 135.93, 130.08, 128.93, 128.69, 128.44, 128.40, 128.29, 125.09, 122.87, 116.34, 103.58, 77.95, 68.47, 66.36, 17.68, −1.40. **MS** (ESI-QMS) *m*/*z*: [M + H]^+^ calcd for C_25_H_29_BrN_3_O_2_SSi, 542.1; found, 542.2.

#### General Procedures for Compounds **7a–f**

The starting compounds (**6a–f**) were dissolved in TFA (2 mL/100 mg of the material) and stirred at room temperature for 15 min. Trifluoroacetic acid was evaporated and co-evaporated with water (2×), and a cold solution of ammonia in ethanol (2 mL/100 mg of the material) was added. The mixture was evaporated to dryness, and the solid was dissolved in dimethylformamide (5 mL). The sample was subjected to purification by C18 reverse-phase flash chromatography (water to methanol) in a liquid injection mode. The product was slowly washed from the column within the second half of the gradient. Fractions were analyzed by TLC, and fractions with the pure product were collected and evaporated to obtain the title compound as a white solid.

##### 7-(Phenylthio)-3,5-dihydro-4*H*-pyrrolo[3,2-*d*]pyrimidin-4-one (**7a**)

The general procedure afforded 44 mg (56%) of the title compound. **^1^H NMR** (401 MHz, DMSO-*d*_6_) δ 7.84 (s, 1H), 7.72 (s, 1H), 7.21 (dd, *J* = 7.7 Hz, 2H), 7.13–6.97 (m, 3H). **^13^C NMR** (101 MHz, DMSO-*d*_6_) δ 153.73, 145.66, 142.84, 138.98, 133.69, 128.84, 125.60, 124.91, 119.21, 102.05. **HRMS** (ESI-FTMS) *m*/*z*: [M + H]^+^ calcd for C_12_H_10_ON_3_S, 244.0539; found, 244.0537.

##### 7-((2-Methylphenyl)thio)-3,5-dihydro-4*H*-pyrrolo[3,2-*d*]pyrimidin-4-one (**7b**)

The general procedure afforded 44 mg (51%) of the title compound. **^1^H NMR** (401 MHz, DMSO-*d*_6_) δ 7.83 (s, 1H), 7.70 (s, 1H), 7.18–7.11 (m, 1H), 7.02–6.90 (m, 2H), 6.70–6.55 (m, 1H), 2.36 (s, 3H). **^13^C NMR** (101 MHz, DMSO-*d*_6_) δ 153.79, 145.80, 142.83, 138.03, 133.77, 133.54, 129.74, 126.39, 125.07, 124.54, 119.34, 101.44, 19.44. **HRMS** (ESI-FTMS) *m*/*z*: [M + Na]^+^ calcd for C_13_H_11_N_3_NaOS, 280.0515; found, 280.0515. LC/MS purity 93% (at 254 nm).

##### 7-(((2-Bromo)phenyl)thio)-3,5-dihydro-4*H*-pyrrolo[3,2-*d*]pyrimidin-4-one (**7c**)

The general procedure afforded 58 mg (51%) of the title compound. **^1^H NMR** (401 MHz, DMSO-*d*_6_) δ 12.80 (s, 1H), 12.15 (s, 1H), 7.85 (d, *J* = 3.1 Hz, 1H), 7.79 (d, *J* = 2.7 Hz, 1H), 7.56 (dd, *J* = 7.8, 1.4 Hz, 1H), 7.15 (ddd, *J* = 7.6, 1.4 Hz, 1H), 7.01 (ddd, *J* = 7.6, 1.6 Hz, 1H), 6.56 (dd, *J* = 8.0, 1.6 Hz, 1H). **^13^C NMR** (101 MHz, DMSO-*d*_6_) δ 153.63, 145.69, 143.05, 139.98, 134.17, 132.39, 128.07, 126.16, 126.11, 119.48, 118.53, 100.71. **HRMS** (ESI-FTMS) *m*/*z*: [M – H]^−^ calcd for C_12_H_7_BrN_3_OS, 319.9499; found, 319.9494.

##### 7-((2-Hydroxyphenyl)thio)-3,5-dihydro-4*H*-pyrrolo[3,2-*d*]pyrimidin-4-one (**7d**)

The general procedure afforded 44 mg (62%) of the title compound. **^1^H NMR** (401 MHz, DMSO-*d*_6_) δ 12.62 (s, 1H), 12.09 (s, 1H), 9.87 (s, 1H), 7.83 (d, *J* = 2.1 Hz, 1H), 7.65 (d, *J* = 2.7 Hz, 1H), 6.89 (ddd, *J* = 8.0, 7.2, 1.7 Hz, 1H), 6.77 (dd, *J* = 7.9, 1.3 Hz, 1H), 6.58 (ddd, *J* = 7.6, 1.3 Hz, 1H), 6.48 (dd, *J* = 7.8, 1.6 Hz, 1H). ^13^C NMR (101 MHz, DMSO-*d*_6_) δ 153.63, 153.04, 145.88, 142.62, 133.59, 126.15, 125.50, 125.29, 119.49, 119.16, 114.50, 101.79. **HRMS** (ESI-FTMS) *m*/*z*: [M – H]^−^ calcd for C_12_H_8_N_3_O_2_S, 258.0343; found, 258.03401.

##### 7-(((2-Hydroxymethyl)phenyl)thio)-3,5-dihydro-4*H*-pyrrolo[3,2-*d*]pyrimidin-4-one (**7e**)

The general procedure for removal of protecting groups afforded 51 mg (72%) of the title compound. **^1^H NMR** (401 MHz, DMSO-*d*_6_) δ 7.84 (s, 1H), 7.70 (s, 1H), 7.42 (dd, *J* = 7.5, 1.5 Hz, 1H), 7.10 (ddd, *J* = 7.4, 1.4 Hz, 1H), 7.04 (ddd, *J* = 7.6, 1.6 Hz, 1H), 6.76 (dd, *J* = 7.8, 1.3 Hz, 1H), 4.67 (s, 2H). **^13^C NMR** (101 MHz, DMSO-*d*_6_) δ 153.83, 145.65, 142.85, 138.75, 136.11, 133.66, 127.08, 126.34, 126.05, 124.71, 119.30, 101.85, 60.52. **HRMS** (ESI-FTMS) *m*/*z*: [M + H]^+^ calcd for C_13_H_12_N_3_O_2_S, 274.0645; found, 274.0644.

##### 7-((3-Bromophenyl)thio)-3,5-dihydro-4*H*-pyrrolo[3,2-*d*]pyrimidin-4-one (**7f**)

The general procedure for removal of protecting groups afforded 52 mg (44%) of the title compound. **^1^H NMR** (401 MHz, DMSO-*d*_6_) δ 7.87 (s, 1H), 7.78 (s, 1H), 7.27 (ddd, *J* = 7.9, 2.0, 1.0 Hz, 1H), 7.17 (dd, *J* = 8.0 Hz, 1H), 7.15 (s, 1H), 7.05 (ddd, *J* = 8.0, 1.3 Hz, 1H). **^13^C NMR** (101 MHz, DMSO-*d*_6_) δ 153.71, 145.54, 143.09, 141.98, 133.97, 130.82, 127.74, 127.46, 124.52, 122.14, 119.36, 100.94. **HRMS** (ESI-FTMS) *m*/*z*: [M – H]^−^ calcd for C_12_H_7_BrN_3_OS, 319.9499; found, 319.9494.

##### 7-(Acetylthio)-4-(benzyloxy)-5-((2-(trimethylsilyl)ethoxy)methyl)-5*H*-pyrrolo[3,2-*d*]pyrimidine (**8**)

Compound **4** (9.62 g, 20.0 mmol, 1.0 equiv), copper(I) iodide (0.38 g, 2.00 mmol, 0.1 equiv), 1,10-phenanthroline (0.54 g, 3.00 mmol, 0.15 equiv), and potassium thioacetate (2.97 g, 26.0 mmol, 1.33 equiv) were charged into a round-bottom flask and dried on high vacuum for 10 min. The flask was flushed with argon, and then dry toluene (80 mL) was added (the solution turned to a dark-red color). The mixture was stirred at 125 °C for 4 h. The dark-brown reaction mixture was dissolved in dichloromethane (50 mL), washed with a half-saturated aqueous solution of NaHCO_3_ (1 × 50 mL), 1 M HCl (aq) (1 × 50 mL), and brine (1 × 50 mL), dried with MgSO_4_, filtered, and evaporated. The residue was adsorbed on silica gel in a mixture of cyclohexane/acetone and purified by flash chromatography on silica gel (cyclohexane/ethyl acetate 20–35%), yielding 7.97 g (93%) of the product as an orange solid). **^1^H NMR** (401 MHz, chloroform-*d*) δ 8.63 (s, 1H), 7.57 (s, 1H), 7.50 (d, *J* = 6.3 Hz, 2H), 7.44–7.32 (m, 3H), 5.66 (d, *J* = 9.6 Hz, 4H), 3.51–3.44 (m, 2H), 2.46 (s, 3H), 0.88–0.77 (m, 2H), −0.08 (s, 9H). **^13^C NMR** (101 MHz, DMSO-*d*_6_) δ 194.26, 156.24, 151.36, 137.78, 136.16, 128.80, 128.50, 128.34, 116.18, 100.92, 92.90, 78.06, 68.46, 66.46, 29.96, 17.82, −1.34. **MS** (ESI-QMS) *m*/*z*: [M + H]^+^ calcd for C_21_H_28_N_3_O_3_SSi, 430.2; found, 430.4.

##### 1,2-Bis(4-(benzyloxy)-5-((2-(trimethylsilyl)ethoxy)methyl)-5*H*-pyrrolo[3,2-*d*]pyrimidin-7-yl)disulfane (**9**)

Compound **8** (7.97 g, 18.0 mmol, 1 equiv) was dissolved in dry methanol (80 mL), followed by potassium carbonate (5.12 g, 37 mmol, 2.06 equiv). The mixture was stirred at 50 °C for 1 h, while air was bubbled into the reaction mixture. The reaction was quenched with water (30 mL) and stirred for 30 min. The mixture was extracted with dichloromethane (3 × 50 mL), washed with brine (1 × 60 mL), dried with MgSO_4_, and filtered, and the solvents were evaporated. The residue was adsorbed on silica gel in a mixture of cyclohexane/acetone and purified by flash chromatography on silica gel (cyclohexane/ethyl acetate 20–35%), followed by crystallization from cyclohexane/ethyl acetate (1:1, 30 mL), yielding 5.36 g (75%) of the product as a yellow solid. **^1^H NMR** (400 MHz, chloroform-*d*) δ 8.53 (s, 2H), 7.60 (s, 2H), 7.51 (dd, *J* = 8.1, 1.6 Hz, 4H), 7.44–7.29 (m, 8H), 5.64 (s, 4H), 5.61 (s, 4H), 3.45 (t, *J* = 8.0 Hz, 4H), 0.81 (t, *J* = 8.1 Hz, 4H), −0.10 (s, 18H). **^13^C NMR** (101 MHz, chloroform-*d*) δ 156.12, 151.46, 151.10, 138.34, 136.20, 128.80, 128.51, 128.34, 116.17, 110.52, 77.98, 68.42, 66.41, 17.83, −1.32. **HRMS** (ESI-FTMS) *m*/*z*: [M + H]^+^ calcd for C_38_H_49_O_4_N_6_S_2_Si_2_, 773.2790; found, 773.2780.

##### Sodium 7-((2-((Phosphonato)methoxy)ethyl)thio)-3,5-dihydro-4*H*-pyrrolo[3,2-*d*]pyrimidin-4-one (**13a**)

Step 1: compound **9** (500 mg, 1.3 mmol, 1 equiv) was dissolved in dry dimethylformamide (10 mL) under an argon atmosphere, and the solution was cooled to 0 °C. Sodium hydride (103 mg of 60% oil dispersion, 2.6 mmol, 2 equiv) was added at 0 °C, and the mixture was stirred for 30 min at room temperature. Diisopropyl 2-(chloroethoxy)methylphosphonate (1.3 g, 5.2 mmol, 4 equiv) was added, and the mixture was stirred at 80 °C overnight. The reaction was quenched with a half-saturated aqueous solution of NH_4_Cl, extracted with ethyl acetate (3×), washed with brine (1×), dried with MgSO_4_, and evaporated. The resulting oil was used in the next step without further characterization. **MS** (ESI-QMS) *m*/*z*: [M + H]^+^ calcd for C_28_H_45_N_3_O_6_PSSi, 610.3; found, 610.2. Step 2: the oil was dissolved in trifluoroacetic acid (2 mL/100 mg of the material) at room temperature and stirred for 15 min. Trifluoroacetic acid was evaporated and co-evaporated with water (2×), and a cold solution of ammonia in ethanol (2 mL/100 mg of the material) was added. The mixture was evaporated to dryness, and the solid was adsorbed on silica gel in a mixture of cyclohexane/acetone. Purification by flash chromatography on silica gel (chloroform to methanol (0–20%) afforded 208 mg (56%) of the diisopropyl phosphonate as a white solid, which was used in the next step without further purification. **MS** (ESI-QMS) *m*/*z*: [M + H]^+^ calcd for C_15_H_25_N_3_O_5_PS, 390.1; found, 390.3. Step 3: a flask charged with the diisopropyl phosphonate (100 mg, 0.2568 mmol, 1 equiv) was sealed with a septum, and flushed with argon, and then dry pyridine (10 mL/1 mmol of the starting compound) was added, followed by trimethylsilyl bromide (1 mL/1 mmol of the starting compound). The mixture was stirred at room temperature overnight and then evaporated to dryness. The residue was dissolved in a 2 M aq solution of triethylammonium bicarbonate and a small amount of methanol, and it was evaporated to dryness. The solid residue was dissolved in a small amount of water (solubility can be enhanced by the addition of several drops of aqueous ammonia) and purified by HPLC (C18, gradient H_2_O/MeOH). Product-containing fractions were filtered through a short pad of Dowex 50 in Na^+^-cycle, and solvents were evaporated. The purified product was lyophilized from water, yielding 51 mg (66%) of the title compound as a white solid. **^1^H NMR** (400 MHz, deuterium oxide) δ 8.09 (s, 1H), 7.74 (s, 1H), 3.68 (t, *J* = 6.4 Hz, 2H), 3.58 (d, *J* = 8.7 Hz, 2H), 2.95 (t, *J* = 6.4 Hz, 2H). **^13^C NMR** (101 MHz, deuterium oxide) δ 156.34, 146.99, 144.11, 135.74, 119.23, 106.35, 72.17, 68.55 (d, *J* = 155.4 Hz), 36.21. **HRMS** (ESI-FTMS) *m*/*z* for the acid: [M – H]^−^ calcd for C_9_H_11_O_5_N_3_PS, 304.0163; found, 304.0160.

##### Sodium 7-((2-(2-(Phosphonato)ethoxy)ethyl)thio)-3,5-dihydro-4*H*-pyrrolo[3,2-*d*]pyrimidin-4-one (**13b**)

Following the previous procedure with compound **9** (500 mg, 1.30 mmol, 1 equiv), dimethylformamide (10 mL), sodium hydride (103 mg of 60% oil dispersion, 2.6 mmol, 2 equiv), and diethoxy 2-(2-chloroethoxy)ethylphosphonate (1.40 g, 5.20 mmol, 4 equiv), the procedure afforded 282 mg (54%) of the diethyl phosphonate intermediate (step 2) as a white solid (**MS** (ESI-QMS) *m*/*z*: [M + H]^+^ calcd for C_15_H_25_N_3_O_5_PS, 390.1; found, 390.3) and 46 mg (51%) of the title compound as a white solid. **^1^H NMR** (400 MHz, deuterium oxide) δ 8.08 (s, 1H), 7.69 (s, 1H), 3.73–3.63 (m, 2H), 3.61 (t, *J* = 6.2 Hz, 2H), 2.91 (t, *J* = 6.2 Hz, 2H), 1.95–1.81 (m, 2H). **^13^C NMR** (101 MHz, deuterium oxide) δ 156.32, 147.10, 144.08, 135.70, 119.25, 106.41, 69.90, 67.83 (d, *J* = 2.4 Hz), 36.30, 30.45 (d, *J* = 128.8 Hz **HRMS** (ESI-FTMS) *m*/*z* for the acid: [M – H]^−^ calcd for C_10_H_13_N_3_O_5_PS, 318.0319; found 318.0321.

##### Diisopropyl (2-Iodobenzyl)phosphonate (**15a**)

2-Iodobenzyl bromide (3 g, 10.1 mmol, 1 equiv) was charged into a microwave reactor tube, and the tube was flushed with argon. Dry toluene (20 mL) and triisopropyl phosphite (3 mL, 11.1 mmol, 1.1 equiv) were added subsequently, and the mixture was heated in the microwave reactor at 160 °C for 1 h. The mixture was evaporated and purified by flash chromatography on silica gel (cyclohexane to 40% ethyl acetate modified with 10% of methanol (v/v_i_)). The procedure afforded 3.40 g (88%) of the product as a clear oil. **^1^H NMR** (401 MHz, chloroform-*d*) δ 7.83 (dd, *J* = 7.9, 1.2 Hz, 1H), 7.50 (ddt, *J* = 7.7, 2.8, 1.7 Hz, 1H), 7.29 (ddd, *J* = 7.6, 1.1 Hz, 1H), 6.91 (ddd, *J* = 7.9, 2.0 Hz, 1H), 4.82–4.33 (m, 2H), 3.36 (d, *J* = 22.0 Hz, 2H), 1.29 (d, *J* = 6.2 Hz, 6H), 1.18 (d, *J* = 6.2 Hz, 6H). **^13^C NMR** (101 MHz, chloroform-*d*) δ 139.75 (d, *J* = 2.9 Hz), 135.95 (d, *J* = 8.2 Hz), 130.79 (d, *J* = 5.1 Hz), 128.57 (d, *J* = 3.6 Hz), 128.37 (d, *J* = 3.1 Hz), 101.87 (d, *J* = 9.6 Hz), 70.97 (d, *J* = 6.7 Hz), 39.47 (d, *J* = 140.0 Hz), 24.83–23.34 (m). **^31^P NMR** (162 MHz, chloroform-*d*) δ 26.23. **MS** (ESI-QMS) *m*/*z*: [M + H]^+^ calcd for C_13_H_21_IO_3_P, 383.0; found, 383.0.

##### Diethyl (*E*)-(2-Iodostyryl)phosphonate (**15b**)

A flask was charged with 2-iodobenzaldehyde (2 g, 8.62 mmol, 1 equiv), dichloromethane (20 mL), and NaOH (20 mL of 5 M aqueous solution). Tetraethyl methylenediphosphonate (2.57 mL, 10.3 mmol, 1.2 equiv) was added, and the mixture was vigorously stirred at room temperature overnight. The reaction mixture was extracted with dichloromethane (3×), washed with brine (1×), dried with MgSO_4_, filtered, and evaporated. The oil was adsorbed on silica gel in cyclohexane and purified by flash chromatography on silica gel (chloroform to 10% of methanol), yielding 3.1 g (98%) of the product as a clear oil. **^1^H NMR** (401 MHz, chloroform-*d*) δ 7.87 (dd, *J* = 7.9, 1.2 Hz, 1H), 7.63 (dd, *J* = 22.3, 17.3 Hz, 1H), 7.52 (dd, *J* = 7.9, 1.6 Hz, 1H), 7.35 (dddd, *J* = 7.9, 7.3, 1.3, 0.6 Hz, 1H), 7.04 (ddd, *J* = 7.7, 1.7 Hz, 1H), 6.15 (dd, *J* = 18.1, 17.3 Hz, 1H), 4.24–4.09 (m, 4H), 1.37 (t, *J* = 7.1 Hz, 6H). **^13^C NMR** (101 MHz, chloroform-*d*) δ 151.33 (d, *J* = 7.5 Hz), 140.01, 138.38 (d, *J* = 23.7 Hz), 131.28, 128.68, 127.30, 117.86 (d, *J* = 190.6 Hz), 100.68, 62.26 (d, *J* = 5.7 Hz), 16.56 (d, *J* = 6.4 Hz). **^31^P NMR** (162 MHz, chloroform-*d*) δ 20.30. **MS** (ESI-QMS) *m*/*z*: [M + H]^+^ calcd for C_12_H_17_IO_3_P, 367.0; found, 367.0.

##### Diisopropyl ((2-Iodophenoxy)methyl)phosphonate (**15c**)

2-Iodophenol (2 g, 9.09 mmol, 1.0 equiv) was dissolved in dry tetrahydrofuran (20 mL) under an argon atmosphere. The solution was cooled to 0 °C, sodium hydride (382 mg of 60% oil dispersion, 9.54 mmol, 1.05 equiv) was added, and the mixture was stirred at 0 °C for 15 min. Then, TfOCH_2_P(O)(O*i*-Pr)_2_ (3.58 g, 10.9 mmol, 1.2 equiv) was added, and the mixture was stirred at 0 °C for additional 15 min. The reaction was quenched with a half-saturated aqueous solution of NH_4_Cl and extracted with ethyl acetate (3×). The organic phase was washed with brine (1×), dried with MgSO_4_, and filtered, and the solvents were evaporated. The oil was adsorbed on silica gel in a mixture of cyclohexane/acetone and purified by flash chromatography on silica gel (chloroform to 10% of methanol), yielding 2.97 g (100%) of diisopropyl ((2-iodophenoxy)methyl)phosphonate as a clear oil. **^1^H NMR** (401 MHz, chloroform-*d*) δ 7.76 (dd, *J* = 7.8, 1.6 Hz, 1H), 7.30 (ddd, *J* = 8.3, 7.4, 1.6 Hz, 1H), 6.89 (dd, *J* = 8.3, 1.3 Hz, 1H), 6.74 (ddd, *J* = 7.6, 1.3 Hz, 1H), 4.96–4.82 (m, 2H), 4.27 (d, *J* = 10.1 Hz, 2H), 1.42–1.35 (m, 12H). **^13^C NMR** (101 MHz, chloroform-*d*) δ 157.47 (d, *J* = 13.8 Hz), 139.84, 129.68, 123.69, 112.29, 86.11, 72.42 (d, *J* = 6.6 Hz), 63.58 (d, *J* = 170.5 Hz), 26.51–21.78 (m). **^31^P NMR** (162 MHz, chloroform-*d*) δ 18.31. **MS** (ESI-QMS) *m*/*z*: [M + H]^+^ calcd for C_13_H_21_IO_4_P, 399.0; found, 399.0.

##### 7-((2-(((Diisopropoxy)phosphoryl)methoxy)phenyl)thio)-3,5-dihydro-4*H*-pyrrolo[3,2-*d*]pyrimidin-4-one (**17c**)

Note: this is an optimized procedure for the telescoped synthesis of compound **17c***.* A flask was charged with compound **4** (20.0 g, 41.5 mmol, 1.0 equiv), copper(I) iodide (7.79 g, 4.15 mmol, 0.1 equiv), and 1,10-phenanthroline (1.50 g, 8.31 mmol, 0.2 equiv). The flask was sealed with a septum and flushed with argon. Dry toluene (200 mL), triethylamine (8.67 mL, 62.3 mmol, 1.5 equiv), and 2-mercaptophenol (5.16 mL, 49.9 mmol, 1.2 equiv) were added subsequently to the mixture. The mixture was heated at 120 °C for 2 h. The resulting dark brown mixture was diluted with chloroform, washed with a half-saturated aqueous NaHCO_3_ solution (2×), 1 M HCl (aq) (2×), brine (1×), and dried with MgSO_4_. Solvents were evaporated, and the crude solid was co-evaporated from toluene (2×). The solid was dissolved in dry dimethylformamide (100 mL), and the flask was flushed with argon. The mixture was stirred at 60 °C when TsOCH_2_P(O)(O*i*-Pr)_2_ (21.8 g; 62.3 mmol; 1.5 equiv) and potassium *tert*-butoxide (7.00 g; 62.3 mmol; 1.5 equiv) were added. The mixture was stirred at 60 °C for 1 h, and an additional portion of TsOCH_2_P(O)(O*i*-Pr)_2_ (21.8 g; 62.3 mmol; 1.5 equiv) with potassium *tert*-butoxide (7.00 g; 62.3 mmol; 1.5 equiv) was added. The addition was repeated after another 1 h. The mixture was cooled to 0 °C, neutralized with 1 M HCl (aq), extracted with chloroform (3×), washed with water (2×) and brine (2×), dried with MgSO_4_, filtered, and evaporated. The mixture was co-evaporated from toluene (2×), and then it was dissolved in trifluoroacetic acid (200 mL). The solution was stirred at room temperature for 15 min. The trifluoroacetic acid was evaporated to dryness and co-evaporated with toluene (2×) and with a saturated solution of ammonia in ethanol (1 × 100 mL). The mixture was adsorbed onto silica gel in a mixture of cyclohexane/acetone, and it was purified by flash chromatography on silica gel (chloroform to 10% methanol), yielding 12.3 g (68%) of the title compound as a white solid. **^1^H NMR** (401 MHz, DMSO-*d*_6_) δ 12.67 (d, *J* = 3.2 Hz, 1H), 12.09 (d, *J* = 3.5 Hz, 1H), 7.82 (d, *J* = 3.6 Hz, 1H), 7.69 (d, *J* = 3.2 Hz, 1H), 7.09 (dd, *J* = 8.2, 1.3 Hz, 1H), 7.03 (ddd, *J* = 8.2, 7.2, 1.6 Hz, 1H), 6.76 (td, *J* = 7.5, 1.3 Hz, 1H), 6.48 (dd, *J* = 7.8, 1.6 Hz, 1H), 4.86–4.72 (m, 2H), 4.43 (d, *J* = 9.9 Hz, 2H), 1.40–1.27 (m, 12H). **^13^C NMR** (101 MHz, DMSO-*d*_6_) δ 153.77 (d, *J* = 14.1 Hz), 153.64, 145.97, 142.70, 133.90, 127.86, 125.46, 125.35, 121.79, 119.32, 111.73, 100.80, 70.94 (d, *J* = 6.4 Hz), 62.49 (d, *J* = 167.0 Hz), 24.73–21.20 (m). **^31^P NMR** (162 MHz, DMSO-*d*_6_) δ 19.70. **MS** (ESI-QMS) *m*/*z*: [M + H]^+^ calcd for C_19_H_25_N_3_O_5_PS, 438.1; found, 438.2.

##### Sodium 7-((2-((Phosphonato)methyl)phenyl)thio)-3,5-dihydro-4*H*-pyrrolo[3,2-*d*]pyrimidin-4-one (**18a**)

Step 1: compound **9** (500 mg, 1.3 mmol, 1 equiv), cesium carbonate (509 mg, 1.6 mmol, 1.2 equiv), copper(I) iodide (25 mg, 0.13 mmol, 0.1 equiv), and 1,10-phenanthroline (46 mg, 0.268 mmol, 0.2 equiv) were charged into a microwave reactor tube, and the tube was flushed with argon. Dry toluene (5 mL) and compound **15a** (596 mg, 1.6 mmol, 1.2 equiv) were added subsequently, and the mixture was heated in the microwave reactor at 120 °C for 2 h. The mixture was dissolved in chloroform, washed with a half-saturated aqueous solution of NaHCO_3_ (2×), 1 M HCl (aq) (2×), and brine (1×), dried with MgSO_4_, and filtered, and solvents were evaporated. The solid was adsorbed on silica gel in a mixture of cyclohexane/acetone and purified by flash chromatography on silica gel (cyclohexane to 20% of ethyl acetate modified with 10% of methanol (v/v_i_)), yielding 754 mg (91%) if intermediate **16a** as a white-off oil. The product was used in the next step without further characterization. **MS** (ESI-QMS) *m*/*z*: [M + H]^+^ calcd for C_32_H_45_N_3_O_5_PSSi, 642.3; found, 642.2. Step 2: intermediate **16a** was dissolved in trifluoroacetic acid (2 mL/100 mg of the material) at room temperature and stirred for 15 min. Trifluoroacetic acid was evaporated and co-evaporated with water (2×), and a cold solution of ammonia in ethanol (2 mL/100 mg of the material) was added. The mixture was evaporated to dryness, and the solid was adsorbed on silica gel in a mixture of cyclohexane/acetone. Purification by flash chromatography on silica gel (chloroform to 10% of methanol) afforded 208 mg (42%) of intermediate **17a**, which was used in the next step without further characterization. **MS** (ESI-QMS) *m*/*z*: [M + H]^+^ calcd for C_19_H_25_N_3_O_4_PS 422,1; found, 422.3. Step 3: a flask charged with 100 mg of intermediate 1**7a** was sealed with a septum and flushed with argon, and then dry pyridine (10 mL/1 mmol of the starting compound) was added, followed by trimethylsilyl bromide (1 mL/1 mmol of the starting compound). The mixture was stirred at room temperature overnight, and then it was evaporated to dryness. The residue was dissolved in 2 M aq solution of triethylammonium bicarbonate and a small amount of methanol, and then it was evaporated to dryness. The solid residue was dissolved in a small amount of water (solubility can be enhanced by the addition of several drops of aqueous ammonia) and purified by HPLC (C18, gradient H_2_O/MeOH). Product-containing fractions were filtered through a short pad of Dowex 50 in Na^+^-cycle, and solvents were evaporated. The purified product was lyophilized from water, yielding 70 mg (78%) of the title compound as a white solid. **^1^H NMR** (400 MHz, deuterium oxide) δ 8.01 (s, 1H), 7.76 (s, 1H), 7.37 (d, *J* = 7.7 Hz, 1H), 7.08 (dd, *J* = 7.6 Hz, 1H), 6.87 (dd, *J* = 7.7 Hz, 1H), 6.72 (d, *J* = 7.9 Hz, 1H), 3.36 (d, *J* = 20.8 Hz, 1H). **^13^C NMR** (101 MHz, deuterium oxide) δ 156.27, 147.02, 144.37, 138.35 (d, *J* = 7.2 Hz), 136.79, 134.47 (d, *J* = 8.3 Hz), 132.15 (d, *J* = 5.1 Hz), 128.38, 127.89, 126.96, 119.57, 104.97, 35.03 (d, *J* = 128.6 Hz). **^31^P NMR** (162 MHz, deuterium oxide) δ 17.54. **HRMS** (ESI-FTMS) *m*/*z* for the acid: [M – H]^−^ calcd for C_13_H_11_O_4_N_3_PS, 336.0213; found, 336.0209. LC/MS purity 93% (at 254 nm).

##### Sodium (*E*)-7-((2-(2-(Phosphonato)vinyl)phenyl)thio)-3,5-dihydro-4*H*-pyrrolo[3,2-*d*]pyrimidin-4-one (**18b**)

Step 1: compound **9** (500 mg, 1.29 mmol, 1 equiv), copper(I) iodide (25 mg, 0.1290 mmol, 0.1 equiv), and cesium carbonate (505 mg, 1.55 mmol, 1.2 equiv) were charged into a microwave reactor tube, and the tube was flushed with argon. Dry toluene (5 mL), compound **15b** (559 mg, 1.33 mmol, 1.1 equiv), and 2-isobutyrylcyclohexanone (43 μL, 0.2580 mmol, 0.2 equiv) were added subsequently, and the mixture was heated in the microwave reactor at 120 °C for 2 h. The mixture was dissolved in chloroform, washed with a half-saturated aqueous solution of NaHCO_3_ (2×), 1 M HCl (aq) (2×), and brine (1×), dried with MgSO_4_, and filtered, and solvents were evaporated. The solid was adsorbed on silica gel in a mixture of cyclohexane/acetone and purified by flash chromatography on silica gel (cyclohexane to 15% of ethyl acetate modified with 10% of methanol (v/v_i_)), yielding 800 mg (99%) of intermediate **16b** as a clear oil. The compound was used in the next step without further characterization. **MS** (ESI-QMS) *m*/*z*: [M + H]^+^ calcd for C_31_H_41_N_3_O_5_PSSi, 626.2; found, 626.2. Step 2: the compound was dissolved in trifluoroacetic acid (2 mL/100 mg of the material) at room temperature and stirred for 15 min. Trifluoroacetic acid was evaporated and co-evaporated with water (2×), and a cold solution of ammonia in ethanol (2 mL/100 mg of the material) was added. The mixture was evaporated; the solid was adsorbed on silica gel in a mixture of cyclohexane/acetone and purified by flash chromatography on silica gel (chloroform to 15% of methanol), yielding 250 mg (48%) of intermediate **17b** as a white solid. **MS** (ESI-QMS) *m*/*z*: [M + H]^+^ calcd for C_18_H_21_N_3_O_4_PS, 406.1; found, 406.2. Step 3: a flask charged with intermediate **17b** (100 mg) was sealed with a septum and flushed with argon, and then dry pyridine (10 mL/1 mmol of the starting compound) was added, followed by trimethylsilyl bromide (1 mL/1 mmol of the starting compound). The mixture was stirred at room temperature overnight and then evaporated to dryness. The residue was dissolved in 2 M aq solution of triethylammonium bicarbonate and a small amount of methanol, and it was evaporated to dryness. The solid residue was dissolved in a small amount of water (solubility can be enhanced by the addition of several drops of aqueous ammonia) and purified by HPLC (C18, gradient H_2_O/MeOH). Product-containing fractions were filtered through a short pad of Dowex 50 in Na^+^-cycle, and solvents were evaporated. The purified product was lyophilized from water, yielding 86 mg (89%) of the title compound as a white solid. **^1^H NMR** (400 MHz, deuterium oxide) δ 8.04 (d, *J* = 3.0 Hz, 1H), 7.81 (d, *J* = 2.9 Hz, 1H), 7.81–7.70 (m, 1H), 7.62 (dd, *J* = 7.7, 2.7 Hz, 1H), 7.19 (ddd, *J* = 7.6, 2.7 Hz, 1H), 7.10 (ddd, *J* = 7.6, 2.7 Hz, 1H), 6.89 (dd, *J* = 7.9, 2.9 Hz, 1H), 6.51 (dd, *J* = 16.8, 3.0 Hz, 1H). **^13^C NMR** (101 MHz, deuterium oxide) δ 156.17, 147.22, 144.45, 138.59 (d, *J* = 6.1 Hz), 138.22, 136.89, 135.91 (d, *J* = 21.3 Hz), 130.63, 128.77, 128.08, 127.66, 127.10 (d, *J* = 175.8 Hz), 119.69, 104.05. **^31^P NMR** (162 MHz, deuterium oxide) δ 16.37. **HRMS** (ESI-FTMS) *m*/*z* for the acid: [M – H]^−^ calcd for C_14_H_11_O_4_N_3_PS, 348.0213; found, 348.0211.

##### Sodium 7-((2-((Phosphonato)methoxy)phenyl)thio)-3,5-dihydro-4*H*-pyrrolo[3,2-*d*]pyrimidin-4-one (**18c**)

Step 1: compound **9** (500 mg, 1.29 mmol, 1 equiv), copper(I) iodide (25 mg, 0.1290 mmol, 0.1 equiv), and cesium carbonate (505 mg, 1.55 mmol, 1.2 equiv) were charged into a microwave reactor tube, and the tube was flushed with argon. Dry toluene (5 mL), compound **15c** (565 mg, 1.33 mmol, 1.1 equiv), and 2-isobutyrylcyclohexanone (43 μL, 0.2580 mmol, 0.2 equiv) were added subsequently, and the mixture was heated in the microwave reactor at 120 °C for 2 h. The mixture was dissolved in chloroform, washed with a half-saturated aqueous solution of NaHCO_3_ (2×), 1 M HCl (aq) (2×), and brine (1×), dried with MgSO_4_, and filtered, and solvents were evaporated. The solid was adsorbed on silica gel in a mixture of cyclohexane/acetone and purified by flash chromatography on silica gel (cyclohexane to 15% of ethyl acetate modified with 10% of methanol (v/v_i_)), yielding 700 mg (81%) of intermediate **16c** as an oil. The compound was used in the next step without further characterization. **MS** (ESI-QMS) *m*/*z*: [M + H]^+^ calcd for C_32_H_45_N_3_O_6_PSSi, 658.3; found, 658.2. Step 2: intermediate **16c** (680 mg) was dissolved in trifluoroacetic acid (2 mL/100 mg of the material) at room temperature and stirred for 15 min. Trifluoroacetic acid was evaporated and co-evaporated with water (2×), and a cold solution of ammonia in ethanol (2 mL/100 mg of the material) was added. The mixture was evaporated; the solid was adsorbed on silica gel in a mixture of cyclohexane/acetone and purified by flash chromatography on silica gel (chloroform to 15% of methanol), yielding 320 mg (71%) of intermediate **17c** as a white solid. **MS** (ESI-QMS) *m*/*z*: [M + H]^+^ calcd for C_19_H_25_N_3_O_5_PS, 438.1; found, 438.2. Step 3: a flask charged with the intermediate **17c** (100 mg) was sealed with a septum and flushed with argon, and then dry pyridine (10 mL/1 mmol of the starting compound) was added followed by trimethylsilyl bromide (1 mL/1 mmol of the starting compound). The mixture was stirred at room temperature overnight, and then it was evaporated to dryness. The residue was dissolved in a 2 M aq solution of triethylammonium bicarbonate and a small amount of methanol, and it was evaporated to dryness. The solid residue was dissolved in a small amount of water (solubility can be enhanced by the addition of several drops of aqueous ammonia) and purified by HPLC (C18, gradient H_2_O/MeOH). Product-containing fractions were filtered through a short pad of Dowex 50 in Na^+^-cycle, and solvents were evaporated. The purified product was lyophilized from water, yielding 72 mg (79%) of the title compound as a white solid. **^1^H NMR** (401 MHz, deuterium oxide) δ 8.03 (s, 1H), 7.82 (s, 1H), 7.17 (ddd, *J* = 8.7, 7.2, 1.6 Hz, 1H), 7.11 (dd, *J* = 8.4, 1.3 Hz, 1H), 6.76 (ddd, *J* = 7.5, 1.3 Hz, 1H), 6.65 (dd, *J* = 7.8, 1.6 Hz, 1H), 4.23 (d, *J* = 9.8 Hz, 2H). **^13^C NMR** (101 MHz, deuterium oxide) δ 156.66 (d, *J* = 13.2 Hz), 156.53, 147.59, 144.48, 137.34, 128.30, 128.04, 127.66, 123.21, 119.81, 113.62, 102.97, 66.53 (d, *J* = 156.8 Hz). **^31^P NMR** (162 MHz, deuterium oxide) δ 16.07. **HRMS** (ESI-FTMS) *m*/*z* for the acid: [M – H]^−^ calcd for C_13_H_11_O_5_N_3_PS, 352.0163; found, 352.0158.

##### Sodium 7-((3-(Phosphonato)phenyl)thio)-3,5-dihydro-4*H*-pyrrolo[3,2-*d*]pyrimidin-4-one (**21**)

Step 1: Pd_2_(dba)_3_ (84 mg, 0.0922 mmol, 0.1 equiv) and xantphos (107 mg, 0.1843 mmol, 0.2 equiv) were dissolved in dry dioxane (5 mL) under an argon atmosphere, followed by triethylamine (192 μL, 1.4 mmol, 1.5 equiv). The mixture was stirred for 15 min at room temperature, and a solution of compound **6f** (500 mg, 0.9215 mmol, 1 equiv) and diethyl phosphite (130 μL, 1.0 mmol, 1.1 equiv) in dry dioxane (2 mL) was added. The mixture was stirred for 24 h at 90 °C, and then it was cooled to 0 °C, quenched with 1 M HCl (aq), extracted with ethyl acetate (3×), washed with brine (1×), dried with magnesium sulfate, and evaporated. Purification by flash chromatography on silica gel (cyclohexane to 15% of ethyl acetate modified with 10% of methanol (v/v_i_)) afforded 514 mg (93%) of intermediate **19** as a white-off oil, which was used in the next step without further characterization. **MS** (ESI-QMS) *m*/*z*: [M + H]^+^ calcd for C_29_H_39_N_3_O_5_PSSi, 600.2; found, 600.2. Step 2: The oil was dissolved in trifluoroacetic acid (2 mL/100 mg of the material) at room temperature and stirred for 15 min. Trifluoroacetic acid was evaporated and co-evaporated with water (2×), and a cold solution of ammonia in ethanol (2 mL/100 mg of the material) was added. The mixture was evaporated to dryness, and the solid was adsorbed on silica gel in a mixture of cyclohexane/acetone. Purification by flash chromatography on silica gel (chloroform to 10% of methanol) afforded 334 mg (97%) of intermediate **20**, which was used in the next step without further characterization. **MS** (ESI-QMS) *m*/*z*: [M + H]^+^ calcd for C_16_H_19_N_3_O_4_PS, 380.1; found, 380.2. Step 3: a flask charged with the intermediate **20** was sealed with a septum and flushed with argon, and then dry pyridine (10 mL/1 mmol of the starting compound) was added, followed by trimethylsilyl bromide (1 mL/1 mmol of the starting compound). The mixture was stirred at room temperature overnight and then evaporated to dryness. The residue was dissolved in a 2 M aq solution of triethylammonium bicarbonate and a small amount of methanol, and it was evaporated to dryness. The solid residue was dissolved in a small amount of water (solubility can be enhanced by the addition of several drops of aqueous ammonia) and purified by HPLC (C18, gradient H_2_O/MeOH). Product-containing fractions were filtered through a short pad of Dowex 50 in Na^+^-cycle, and solvents were evaporated. The purified product was lyophilized from water, and yielded 199 mg (70%) of the title compound as a white solid. **^1^H NMR** (401 MHz, deuterium oxide) δ 8.07 (s, 1H), 7.73 (s, 1H), 7.71 (ddd, *J* = 11.9, 1.4 Hz, 1H), 7.48 (dddd, *J* = 11.2, 7.5, 1.2 Hz, 1H), 7.21 (ddd, *J* = 7.6, 3.0 Hz, 1H), 6.94 (dddd, *J* = 7.9, 2.1, 1.1 Hz, 1H). **^13^C NMR** (101 MHz, deuterium oxide) δ 165.12, 153.38, 148.76, 143.49 (d, *J* = 165.0 Hz), 139.12 (d, *J* = 14.0 Hz), 136.15, 129.85 (d, *J* = 13.0 Hz), 129.46 (d, *J* = 9.7 Hz), 128.91 (d, *J* = 8.7 Hz), 127.34 (d, *J* = 2.4 Hz), 122.22, 101.04. **^31^P NMR** (162 MHz, deuterium oxide) δ 12.86. **HRMS** (ESI-FTMS) *m*/*z* for the acid: [M – H]^−^ calcd for C_12_H_9_O_4_N_3_PS, 322.0057; found, 322.0053. LC/MS purity 91% (at 254 nm).

##### Sodium 7-((2-(3-Phosphonato-2-oxapropyl)phenyl)thio)-3,5-dihydro-4*H*-pyrrolo[3,2-*d*]pyrimidin-4-one (**23**)

Step 1: compound **6e** (100 mg, 0.2026 mmol, 1 equiv) was dissolved in dry tetrahydrofuran (50 mL) under an argon atmosphere. The solution was cooled to 0 °C, sodium hydride (12 mg of 60% oil dispersion, 0.3039 mmol, 1.5 equiv) was added, and the mixture was stirred at 0 °C for 15 min. Then, TfOCH_2_P(O)(O*i*-Pr)_2_ (133 mg, 0.4052 mmol, 2 equiv) was added, and the mixture was stirred at 0 °C for additional 15 min. The reaction was quenched with a half-saturated aqueous solution of NH_4_Cl, extracted with ethyl acetate (3×), washed with brine, dried with MgSO_4_, and filtered, and solvents were evaporated. The solid was adsorbed on silica gel in a mixture of cyclohexane/acetone and purified by flash chromatography on silica gel (cyclohexane to 20% of ethyl acetate modified with 10% of methanol (v/v_i_)), yielding 82 mg (60%) of intermediate **22**, which was used in the next step without further characterization. **MS** (ESI-QMS) *m*/*z*: [M + H]^+^ calcd for C_33_H_47_N_3_O_6_PSSi, 672.3; found, 672.4. Step 2: intermediate **22** was dissolved in trifluoroacetic acid (2 mL/100 mg of the material) at room temperature and stirred for 15 min. Trifluoroacetic acid was evaporated and co-evaporated with water (2×), and a cold solution of ammonia in ethanol (2 mL/100 mg of the material) was added. The mixture was evaporated to dryness to afford the crude diisopropyl phosphonate. **MS** (ESI-QMS) *m*/*z*: [M + H]^+^ calcd for C_20_H_27_N_3_O_5_PS, 452.1; found, 452.2. The solid in a flask was sealed with a septum and flushed with argon, and then dry pyridine (10 mL/1 mmol of the starting compound) was added followed by trimethylsilyl bromide (1 mL/1 mmol of the starting compound). The mixture was stirred at room temperature overnight and then evaporated to dryness. The residue was dissolved in a 2 M aq solution of triethylammonium bicarbonate and a small amount of methanol, and it was evaporated to dryness. The solid residue was dissolved in a small amount of water (solubility can be enhanced by addition of several drops of aqueous ammonia) and purified by HPLC (C18, gradient H_2_O/MeOH). Product-containing fractions were filtered through a short pad of Dowex 50 in Na^+^-cycle, and solvents were evaporated. The purified product was lyophilized from water, yielding 6 mg (12%) of the title compound as a white solid. **^1^H NMR** (500 MHz, methanol-*d*_4_) δ 8.00 (s, 1H), 7.76 (s, 1H), 7.46 (dd, *J* = 7.6, 1.5 Hz, 1H), 7.16 (ddd, *J* = 7.5, 1.2 Hz, 1H), 7.07 (ddd, *J* = 7.7, 1.5 Hz, 1H), 6.78 (dd, *J* = 8.0, 1.0 Hz, 1H), 4.85 (s, 2H), 3.66 (d, *J* = 8.6 Hz, 2H). **^13^C NMR** (126 MHz, methanol-*d*_4_) δ 156.19, 147.02, 144.04, 138.53, 136.54, 135.72, 130.54, 129.56, 127.54, 126.53, 119.48, 103.99, 73.42 (d, *J* = 11.1 Hz), 68.90 (d, *J* = 152.7 Hz). **^31^P NMR** (162 MHz, deuterium oxide) δ 16.23. **HRMS** (ESI-FTMS) *m*/*z* for the acid: [M – H]^−^ calcd for C_14_H_13_O_5_N_3_PS, 366.0319; found, 366.0317.

##### (5-Bromo-2-iodophenyl)methanol (**25**)

The compound was synthesized according to the published procedure and an analysis matched to published results.^64^ Methyl 5-bromo-2-iodobenzoate (4.0 g, 11.7 mmol, 1 equiv) was dissolved in dry dichloromethane (24 mL), and the solution was cooled to 0 °C. Diisobutylaluminum hydride (23.4 mL of a 1 M toluene solution, 23.4 mmol, 2 equiv) was added, and the mixture was stirred overnight at room temperature. The reaction was quenched with an aqueous solution of citric acid (15%), extracted with dichloromethane (3×), washed with brine (1×), dried with MgSO_4_, and filtered. Evaporation of solvents afforded 3.52 g (96%) of the title compound as a yellowish solid. **^1^H NMR** (401 MHz, chloroform-*d*) δ 7.64 (d, *J* = 8.3 Hz, 1H), 7.62 (d, *J* = 2.4 Hz, 1H), 7.13 (dd, *J* = 8.4, 2.4 Hz, 1H), 4.62 (s, 2H), 2.06 (s, 1H). **^13^C NMR** (101 MHz, chloroform-*d*) δ 144.70, 140.43, 132.31, 131.27, 123.13, 94.76, 68.82. **MS** (ESI-QMS) *m*/*z*: [M + H – H_2_O + CH_3_CN]^+^ calcd for C_9_H_8_BrIN, 335.9; found, 335.9.

##### 5-Bromo-2-iodobenzaldehyde (**26**)

The compound was synthesized according to the published procedure and an analysis matched to published results.^64^ Compound **25** (3 g, 9.58 mmol, 1 equiv) and pyridinium dichromate (7.2 g, 19.2 mmol, 2 equiv) were dissolved in dry dichloromethane (40 mL), and the mixture was stirred at room temperature for 4 h. The mixture was filtered through Celite and washed with diethyl ether, and the solvents were evaporated. The solid was adsorbed on silica gel in a mixture of cyclohexane/acetone, and it was purified by flash column chromatography on silica gel (cyclohexane to 20% of ethyl acetate modified with 10% of methanol (v/v_i_)), yielding 2.3 g (77%) of the title compound as a white solid (85% NMR purity). **^1^H NMR** (401 MHz, chloroform-*d*) δ 9.99 (s, 1H), 7.99 (d, *J* = 2.5 Hz, 1H), 7.81 (d, *J* = 8.4 Hz, 1H), 7.41 (dd, *J* = 8.4, 2.5 Hz, 1H). **^13^C NMR** (101 MHz, chloroform-*d*) δ 194.45, 141.95, 138.37, 136.47, 133.24, 123.63, 98.39. **MS** (CI-QMS) *m*/*z*: [M + H]^+^ calcd for C_7_H_5_BrIO, 310.9; found, 310.9.

##### Diethyl (*E*)-(5-Bromo-2-iodostyryl)phosphonate (**27**)

Tetraethyl methylenediphosphonate (1.52 mL, 7.72 mmol, 1.2 equiv) was dissolved in dry tetrahydrofuran (40 mL) under an argon atmosphere, followed by potassium *tert*-butoxide (867 mg, 7.72 mmol, 1.2 equiv). The mixture was stirred at room temperature for 1 h, and a solution of compound **26** (2 g, 6.43 mmol, 1 equiv) in dry tetrahydrofuran (15 mL) was added. The mixture was stirred at room temperature for 1 h, and the reaction was quenched with HCl (1 M (aq)), extracted with dichloromethane (3×), washed with brine (1×), dried with MgSO_4_, and filtered. Solvents were evaporated, the solid was adsorbed on silica gel in a mixture of cyclohexane/acetone, and it was purified by flash column chromatography on silica gel (chloroform to 5% of methanol), yielding 1.82 g (63%) of the title compound as a clear oil. **^1^H NMR** (401 MHz, chloroform-*d*) δ 7.72 (dd, *J* = 8.5, 1.5 Hz, 1H), 7.63 (d, *J* = 2.0 Hz, 1H), 7.55 (ddd, *J* = 22.2, 17.2, 1.5 Hz, 1H), 7.17 (d, *J* = 8.4 Hz, 1H), 6.17 (dd, *J* = 17.3 Hz, 1H), 4.17 (p, *J* = 7.3 Hz, 2H), 1.38 (t, *J* = 7.2 Hz, 6H). **^13^C NMR** (101 MHz, chloroform-*d*) δ 149.76 (d, *J* = 8.0 Hz), 141.09, 140.26 (d, *J* = 24.1 Hz), 133.98, 130.11, 122.92, 119.44 (d, *J* = 190.0 Hz), 98.21, 62.23 (d, *J* = 5.4 Hz), 16.48 (d, *J* = 6.5 Hz). **MS** (ESI-QMS) *m*/*z*: [M + H]^+^ calcd for C_12_H_16_BrIO_3_P, 449.9; found, 444.9.

##### 4-Bromo-1-(bromomethyl)-2-iodobenzene (**29**)

The compound was synthesized according to the published procedure, and an analysis matched to published results.^65^ 4-Bromo-2-iodo-1-methylbenzene (3.94 g, 13.3 mmol, 1 equiv) was dissolved in dry 1,2-dichloroethane (19 mL) under an argon atmosphere, and *N*-bromosuccinimide (2.63 g, 14.6 mmol, 1.1 equiv) was added, followed by dibenzoyl peroxide (164 mg, 0.6630 mmol, 0.05 equiv). The mixture was refluxed for 4 h, and the reaction was quenched with a half-saturated aqueous solution of NH_4_Cl, extracted with chloroform (3×), washed with brine, dried with MgSO_4_, filtered, and evaporated. The solid was adsorbed on silica gel in a mixture of cyclohexane/acetone and purified by flash chromatography on silica gel (cyclohexane), yielding 4.35 g (89%) of the title compound as a white solid (85% UV/LC purity). **^1^H NMR** (401 MHz, chloroform-*d*) δ 8.00 (d, *J* = 2.1 Hz, 1H), 7.46 (dd, *J* = 8.3, 1.7 Hz, 1H), 7.33 (d, *J* = 8.3 Hz, 1H), 4.54 (s, 2H). **^13^C NMR** (101 MHz, chloroform-*d*) δ 141.98, 139.32, 132.04, 131.28, 122.85, 100.38, 37.65. **MS** (ESI-QMS) *m*/*z*: [M + H – HBr + H_2_O + CH_3_CN]^+^ calcd for C_9_H_10_BrINO, 353.9; found, 353.9.

##### 4-Bromo-2-iodobenzaldehyde (**30**)

The compound was synthesized according to the published procedure, and an analysis matched to published results.^65^*N*-Methylmorpholine *N*-oxide (3.74 g, 31.9 mmol, 3 equiv) was dissolved in dry acetonitrile (80 mL) under an argon atmosphere, molecular sieves (26 g, 4 Å) were added, and the suspension was cooled to 0 °C. Compound **29** (4.0 g, 10.6 mmol, 1 equiv) was added, and the mixture was stirred at 0 °C for 2 h. The mixture was filtered through a short pad of silica gel and washed with cyclohexane, yielding 3.3 g (100%) of the title compound as a white solid (65% UV/LC purity). **^1^H NMR** (401 MHz, chloroform-*d*) δ 9.98 (d, *J* = 0.8 Hz, 1H), 8.12 (d, *J* = 1.8 Hz, 1H), 7.72 (d, *J* = 8.3 Hz, 1H), 7.61 (ddd, *J* = 8.3, 1.8, 0.8 Hz, 1H). **^13^C NMR** (101 MHz, chloroform-*d*) δ 194.56, 142.67, 133.95, 132.17, 131.00, 130.04, 100.81. **MS** (CI-QMS) *m*/*z*: [M + H]^+^ calcd for C_7_H_5_BrIO, 310.9; found, 310.9.

##### Diethyl (*E*)-(4-Bromo-2-iodostyryl)phosphonate (**31**)

Tetraethyl methylenediphosphonate (2.28 mL, 11.6 mmol, 1.2 equiv) was dissolved in dry tetrahydrofuran (60 mL) under an argon atmosphere, and potassium *tert*-butoxide (1.3 g, 11.6 mmol, 1.2 equiv) was added. The mixture was stirred at room temperature for 1 h, and a solution of compound **30** (3 g, 9.65 mmol, 1 equiv) in dry tetrahydrofuran (20 mL) was added. The mixture was stirred at room temperature for 1 h, and the reaction was quenched with HCl (1 M (aq)), extracted with dichloromethane, washed with brine, dried with MgSO_4_, and filtered. The solvents were evaporated, the solid was adsorbed on silica gel in a mixture of cyclohexane/acetone, and it was purified by flash column chromatography on silica gel (chloroform to 5% of methanol), yielding 2.41 g (56%) of the title compound as a clear oil (60% UV/LC purity). **^1^H NMR** (401 MHz, chloroform-*d*) δ 8.04 (d, *J* = 1.9 Hz, 1H), 7.56 (dd, *J* = 22.3, 17.4 Hz, 1H), 7.51–7.47 (m, 1H), 7.38 (d, *J* = 8.3 Hz, 1H), 6.16 (t, *J* = 17.4 Hz, 1H), 4.21–4.11 (m, 4H), 1.38 (t, *J* = 7.1 Hz, 6H). **^13^C NMR** (101 MHz, chloroform-*d*) δ 150.08 (d, *J* = 7.8 Hz), 141.99 (d, *J* = 1.4 Hz), 131.93, 128.10 (d, *J* = 1.6 Hz), 124.43, 118.61 (d, *J* = 190.7 Hz), 100.96 (d, *J* = 15.9 Hz), 62.37 (d, *J* = 5.7 Hz), 16.92–15.90 (m). **MS** (ESI-QMS) *m*/*z*: [M + H]^+^ calcd for C_12_H_16_BrIO_3_P, 444.9; found, 445.0.

##### 4-(1,3-Dimethylimidazolidin-2-yl)phenol (**33a**)

A flask was charged with toluene (600 mL), and 4-hydroxybenzaldehyde (36 g, 295 mmol, 1 equiv) was added, followed by *N*,*N*′-dimethylethylenediamine (38 mL, 354 mmol, 1.2 equiv). The mixture was stirred at reflux for 15 min, and then 2/3 of the volume was distilled-off. The mixture was cooled in an ice bath; crystals were collected, washed with cold toluene, and dried, yielding 48.6 g (86%) of the title compound as a brownish solid. **^1^H NMR** (401 MHz, DMSO-*d*_6_) δ 9.36 (s, 1H), 7.16 (d, *J* = 8.5 Hz, 2H), 6.71 (d, *J* = 8.5 Hz, 2H), 3.24–3.15 (m, 2H), 3.09 (s, 1H), 2.47–2.38 (m, 2H), 2.01 (s, 6H). **^13^C NMR** (101 MHz, DMSO-*d*_6_) δ 157.42, 129.89, 129.85, 114.72, 91.41, 52.57, 39.10. **MS** (CI-QMS) *m*/*z*: [M + H]^+^ calcd for C_11_H_17_N_2_O, 193.1; found, 193.1.

##### 3-(1,3-Dimethylimidazolidin-2-yl)phenol (**33b**)

Following the previous procedure with 3-hydroxybenzaldehyde (36 g, 295 mmol, 1 equiv) afforded 44.5 g (78%) of the title compound as a brownish solid. **^1^H NMR** (401 MHz, DMSO-*d*_6_) δ 9.27 (s, 1H), 7.11 (dd, *J* = 7.7 Hz, 1H), 6.83 (s, 0H), 6.77 (d, *J* = 7.5 Hz, 1H), 6.69 (dd, *J* = 8.2, 1.5 Hz, 1H), 3.25–3.16 (m, 2H), 3.12 (s, 1H), 2.48–2.42 (m, 2H), 2.04 (s, 6H). **^13^C NMR** (101 MHz, DMSO-*d*_6_) δ 157.17, 141.58, 128.75, 119.64, 115.31, 91.62, 52.76, 39.17. **MS** (CI-QMS) *m*/*z*: [M + H]^+^ calcd for C_11_H_17_N_2_O, 193.1; found, 193.1.

##### 2-(1,3-Dimethylimidazolidin-2-yl)phenol (**33c**)

A flask was charged with toluene (600 mL), and 2-hydroxybenzaldehyde (36 g, 295 mmol, 1 equiv) was added, followed by *N*,*N*′-dimethylethylenediamine (38 mL, 354 mmol, 1.2 equiv). The mixture was stirred at reflux for 15 min, and then toluene was evaporated. The liquid was distilled under high vacuum at ca. 116 °C, yielding 51 g (90%) of the title compound as a yellow liquid. **^1^H NMR** (401 MHz, chloroform-*d*) δ 11.46 (s, 1H), 7.20 (dd, *J* = 7.8 Hz, 1H), 6.96 (d, *J* = 7.5 Hz, 1H), 6.83 (d, *J* = 8.3 Hz, 1H), 6.77 (dd, *J* = 7.4 Hz, 1H), 3.42 (s, 1H), 3.40–3.34 (m, 2H), 2.59–2.51 (m, 2H), 2.32–2.19 (m, 6H). **^13^C NMR** (101 MHz, chloroform-*d*) δ 158.45, 130.60, 129.84, 120.45, 118.25, 116.82, 91.85, 52.23, 39.02. **MS** (CI-QMS) *m*/*z*: [M + H]^+^ calcd for C_11_H_17_N_2_O, 193.1; found, 193.1.

##### 4-Hydroxy-2-iodobenzaldehyde (**34a**)

Compound **33a** (10 g, 52.0 mmol, 1 equiv) was dissolved in dry diethyl ether (250 mL) under an argon atmosphere. The solution was cooled to −78 °C, and a solution of *tert*-butyl lithium (92 mL of 1.7 M in pentane, 156 mmol, 3 equiv) was added keeping the temperature of the solution under −70 °C. The mixture was stirred at room temperature overnight, and then it was cooled to −78 °C again. A solution of 1,2-diiodoethane (44 g, 156 mmol, 3 equiv) and dry diethyl ether (200 mL) was added keeping the temperature under −60 °C. The mixture was stirred at room temperature for 30 min. The reaction was quenched with 1 M HCl (aq), extracted with diethyl ether (3×), washed with brine (1×), dried with MgSO_4_, filtered, and evaporated. The solid was adsorbed on silica gel in a mixture of cyclohexane/acetone, and it was purified by flash column chromatography on silica gel (cyclohexane to 20% of ethyl acetate modified with 10% of methanol (v/v_i_)), yielding 9.4 g (73%) of the title compound as a white solid (81% UV/LC purity). **^1^H NMR** (401 MHz, DMSO-*d*_6_) δ 10.86 (s, 1H), 9.74 (d, *J* = 0.8 Hz, 1H), 7.65 (d, *J* = 8.6 Hz, 1H), 7.37 (d, *J* = 2.3 Hz, 1H), 6.91 (dd, *J* = 8.6, 2.3 Hz, 1H). **^13^C NMR** (101 MHz, DMSO-*d*_6_) δ 193.53, 163.52, 132.00, 126.82, 126.79, 116.18, 103.01. **MS** (ESI-QMS) *m*/*z*: [M + H]^+^ calcd for C_7_H_6_IO_2_, 248.9; found, 249.0.

##### 2-Hydroxy-3-iodobenzaldehyde (**35**)

Following the previous procedure with compound **33c** afforded 4.0 g (31%) of the title compound. **^1^H NMR** (401 MHz, chloroform-*d*) δ 11.80 (d, *J* = 0.5 Hz, 1H), 9.75 (s, 1H), 7.97 (ddd, *J* = 7.8, 1.6, 0.5 Hz, 1H), 7.56 (dd, *J* = 7.7, 1.6 Hz, 1H), 6.83 (dd, *J* = 7.7 Hz, 1H). **^13^C NMR** (101 MHz, chloroform-*d*) δ 196.04, 160.42, 146.17, 134.09, 121.71, 120.59, 85.54. **MS** (ESI-QMS) *m*/*z*: [M + H]^+^ calcd for C_7_H_6_IO_2_, 248.9; found, 249.0.

##### 3-Hydroxy-4-iodobenzaldehyde (**36**)

The compound was synthesized according to the published procedure that describes the product as an incorrect regioisomer.^47^ 3-Hydroxybenzaldehyde (16 g, 130 mmol, 1 equiv) was dissolved in aqueous ammonia (150 mL). A solution of iodine (37.2 g, 140 mmol, 1.1 equiv), potassium iodide (70 g, 422 mmol, 3 equiv), and water (250 mL) was added dropwise at room temperature to the mixture. The mixture was stirred for 3 h at room temperature, and the reaction was quenched at 0 °C by careful addition of hydrochloric acid (5 M) to pH 1. A precipitate was formed, filtered, dissolved in diethyl ether, and filtered, and the solvent was evaporated. The solid was filtered through a short pad of silica gel in a mixture of chloroform/methanol (95:5), and solvents were evaporated. The solid was crystallized from boiling toluene. Crystals were collected, washed with toluene, pentane, and dried, yielding 12.7 g (39%) of the title compound as a white-off solid. **^1^H NMR** (401 MHz, DMSO-*d*_6_) δ 10.90 (s, 1H), 9.88 (s, 1H), 7.95 (d, *J* = 7.9 Hz, 1H), 7.29 (d, *J* = 1.8 Hz, 1H), 7.12 (dd, *J* = 7.9, 1.8 Hz, 1H). **^13^C NMR** (101 MHz, DMSO-*d*_6_) δ 192.68, 157.40, 139.91, 137.54, 122.41, 113.29, 93.85. **MS** (ESI-QMS) *m*/*z*: [M + H]^+^ calcd for C_7_H_6_IO_2_, 248.9; found, 249.0.

##### 2-Iodo-5-methoxybenzaldehyde (**38**)

The compound was synthesized according to the published procedure.^66^ A flask was subsequently charged with 3-methoxybenzaldehyde (20.0 g, 147 mmol, 1 equiv), methanol (600 mL), AgNO_3_ (24.8 g, 147 mmol, 1 equiv), and iodine (42.0 g, 162 mmol, 1.1 equiv). The flask was wrapped in aluminum foil, and the mixture was stirred at room temperature for 1 h. The mixture was filtered through Celite, excess of iodine was titrated by a saturated aqueous solution of Na_2_S_2_O_3_, and solvents were evaporated to dryness. The solid was dissolved in a mixture of chloroform and water, extracted with chloroform (2×), washed with brine (1×), dried with MgSO_4_, filtered, and evaporated. The solid was dissolved in hot ethyl acetate, and the product was precipitated with cyclohexane. Crystals were collected, yielding 24 g (62%) of the title compound as a yellow solid. **^1^H NMR** (401 MHz, DMSO-*d*_6_) δ 9.89 (s, 1H), 7.89 (d, *J* = 8.7 Hz, 1H), 7.30 (d, *J* = 3.2 Hz, 1H), 7.05 (dd, *J* = 8.6, 3.2 Hz, 1H), 3.80 (s, 3H). **^13^C NMR** (101 MHz, DMSO-*d*_6_) δ 195.16, 159.73, 141.25, 135.54, 122.87, 114.36, 89.57, 55.58. **MS** (ESI-QMS) *m*/*z*: [M + H]^+^ calcd for C_8_H_8_IO_2_, 263.0; found, 263.0.

##### 5-Hydroxy-2-iodobenzaldehyde (**34b**)

Compound **38** (5.0 g, 19.1 mmol, 1 equiv) was dissolved in dry dichloromethane (200 mL) under an argon atmosphere. The solution was cooled to −78 °C, a solution of boron tribromide (95 mL of 1 M in dichloromethane, 95.5 mmol, 5 equiv) was added, and the mixture was stirred at room temperature until full conversion was achieved (ca. 3 h). The mixture was cooled to −15 °C, and the reaction was slowly quenched by a saturated aqueous solution of NaHCO_3_, extracted with dichloromethane (3×), washed with brine (1×), dried with MgSO_4_, filtered, and evaporated. The solid was adsorbed on silica gel in a mixture of cyclohexane/acetone, and it was purified by flash column chromatography on silica gel (cyclohexane to 10% of ethyl acetate modified with 10% of methanol (v/v_i_)), yielding 4.1 g (87%) of the title compound as yellow needles. **^1^H NMR** (401 MHz, DMSO-*d*_6_) δ 10.18 (s, 1H), 9.86 (s, 1H), 7.80 (d, *J* = 8.5 Hz, 1H), 7.21 (d, *J* = 3.1 Hz, 1H), 6.89 (dd, *J* = 8.5, 3.1 Hz, 1H). **^13^C NMR** (101 MHz, DMSO-*d*_6_) δ 195.45, 158.09, 141.27, 135.54, 123.90, 116.41, 87.33. **MS** (ESI-QMS) *m*/*z*: [M + H]^+^ calcd for C_7_H_6_IO_2_, 248.9; found, 249.0.

##### 2-(2-Methoxyphenyl)-1,3-dimethylimidazolidine (**40**)

A flask was charged with toluene (600 mL) and 2-methoxybenzaldehyde (36.0 g, 295 mmol, 1 equiv), followed by *N*,*N*′-dimethylethylenediamine (31.3 mL, 325 mmol, 1.1 equiv). The mixture was stirred at reflux for 15 min, and then toluene was evaporated. The solid was recrystallized from hexane yielding 36 g (67%) of the title compound as clear large crystals. **^1^H NMR** (401 MHz, chloroform-*d*) δ 7.67 (dd, *J* = 7.6, 1.9 Hz, 1H), 7.26 (td, *J* = 6.6, 1.9 Hz, 1H), 7.00 (td, *J* = 7.4, 1.3 Hz, 1H), 6.88 (dd, *J* = 8.2, 1.1 Hz, 1H), 4.06 (s, 1H), 3.82 (s, 3H), 3.37–3.31 (m, 2H), 2.65–2.57 (m, 2H), 2.19 (s, 6H). **^13^C NMR** (101 MHz, chloroform-*d*) δ 159.04, 129.40, 129.00, 127.75, 121.26, 110.52, 82.90, 55.63, 53.67, 39.83. **MS** (CI-QMS) *m*/*z*: [M + H]^+^ calcd for C_12_H_19_N_2_O, 207.1; found, 207.1.

##### 2-Iodo-6-methoxybenzaldehyde (**41**)

Compound **40** (10 g, 52.0 mmol, 1 equiv) was dissolved in dry diethyl ether (250 mL) under an argon atmosphere. The solution was cooled to −78 °C, and a solution of *tert*-butyl lithium (61 mL of 1.7 M in pentane, 104 mmol, 2 equiv) was added keeping the temperature of the solution under −70 °C. The mixture was stirred at room temperature overnight, and then it was cooled to −78 °C again. A solution of 1,2-diiodoethane (29.3 g, 104 mmol, 2 equiv) in dry diethyl ether (200 mL) was added keeping the temperature under −60 °C. The mixture was stirred at room temperature for 30 min. The reaction was quenched with 1 M HCl (aq), extracted with diethyl ether (3×), washed with brine (1×), dried with MgSO_4_, filtered, and evaporated. The solid was adsorbed on silica gel in a mixture of cyclohexane/acetone, and it was purified by flash column chromatography on silica gel (cyclohexane to 20% of ethyl acetate modified with 10% of methanol (v/v_i_)), yielding 5.8 g (46%) of the title compound as a white solid. **^1^H NMR** (401 MHz, chloroform-*d*) δ 10.24 (s, 1H), 7.59 (d, *J* = 7.8 Hz, 0H), 7.13 (dd, *J* = 8.4, 7.8 Hz, 1H), 6.99 (dd, *J* = 8.3, 0.9 Hz, 1H), 3.91 (s, 3H). **^13^C NMR** (101 MHz, chloroform-*d*) δ 191.90, 161.98, 135.25, 133.92, 125.04, 112.08, 96.55, 56.16. **MS** (ESI-QMS) *m*/*z*: [M + H]^+^ calcd for C_8_H_8_IO_2_, 263.0; found, 263.0.

##### 2-Hydroxy-6-iodobenzaldehyde (**34c**)

Compound **41** (5.0 g, 19.1 mmol, 1 equiv) was dissolved in dry dichloromethane (200 mL) under an argon atmosphere. The solution was cooled to −78 °C, and a solution of boron tribromide (95 mL of 1 M in dichloromethane, 95.5 mmol, 5 equiv) was added, and the mixture was stirred at room temperature until full conversion was achieved (ca. 3 h). The mixture was cooled to −15 °C, and the reaction was slowly quenched by a saturated aqueous solution of NaHCO_3_. The mixture was extracted with chloroform (3×), washed with brine (1×), dried with MgSO_4_, filtered, and evaporated. The solid was adsorbed on silica gel in a mixture of cyclohexane/acetone, and it was purified by flash column chromatography on silica gel (cyclohexane to 10% of ethyl acetate modified with 10% of methanol (v/v_i_), yielding 4.4 g (93%) of the title compound as a white-off solid. **^1^H NMR** (401 MHz, DMSO-*d*_6_) δ 11.58 (s, 1H), 10.02 (s, 1H), 7.52 (dd, *J* = 7.7, 1.0 Hz, 1H), 7.22 (dd, *J* = 8.4, 7.7 Hz, 1H), 7.00 (dd, *J* = 8.4, 1.1 Hz, 1H). **^13^C NMR** (101 MHz, DMSO-*d*_6_) δ 198.92, 162.10, 137.58, 131.87, 120.06, 118.14, 101.00. **MS** (ESI-QMS) *m*/*z*: [M + H]^+^ calcd for C_7_H_6_IO_2_, 248.9; found, 248.9.

##### Diethyl (*E*)-(4-Hydroxy-2-iodostyryl)phosphonate (**42a**)

Compound **34a** (4.0 g, 16.1 mmol, 1 equiv) was dissolved in dry toluene (80 mL), followed by diethylphosphonoacetic acid (3.8 mL, 19.4 mmol, 1.2 equiv), piperidine (524 μL, 5.13 mmol, 0.33 equiv), and acetic acid (221 μL, 3.86 mmol, 0.24 equiv). The mixture was refluxed overnight in an open flask, and the reaction was quenched with a half-saturated aqueous solution of NaHCO_3_, extracted with chloroform (3×), washed with brine (1×), dried with MgSO_4_, filtered, and evaporated. Purification by C18 reverse-phase flash chromatography (water to methanol, liquid injection in dimethylformamide) afforded 5.3 g (87%) of the title compound as a yellowish oil. **^1^H NMR** (401 MHz, chloroform-*d*) δ 9.48 (s, 1H), 7.62 (dd, *J* = 23.0, 17.3 Hz, 1H), 7.50 (d, *J* = 2.4 Hz, 1H), 7.40 (d, *J* = 8.7 Hz, 1H), 6.92 (ddd, *J* = 8.7, 2.5, 0.6 Hz, 1H), 5.96 (dd, *J* = 19.0, 17.3 Hz, 1H), 4.21–4.09 (m, 4H), 1.37 (t, *J* = 7.1 Hz, 6H). **^13^C NMR** (101 MHz, chloroform-*d*) δ 160.04, 152.40 (d, *J* = 8.3 Hz), 128.63 (d, *J* = 24.2 Hz), 127.86 (d, *J* = 1.3 Hz), 126.92 (d, *J* = 1.4 Hz), 116.68, 111.99 (d, *J* = 194.2 Hz), 102.44, 62.63 (d, *J* = 5.7 Hz), 16.54 (d, *J* = 6.4 Hz). **^31^P NMR** (162 MHz, chloroform-*d*) δ 20.82. **MS** (ESI-QMS) *m*/*z*: [M + H]^+^ calcd for C_12_H_17_IO_4_P, 383.0; found, 383.1.

##### Diethyl (*E*)-(5-Hydroxy-2-iodostyryl)phosphonate (**42b**)

A flask was charged with compound **34b** (4.0 g, 16.1 mmol, 1 equiv) and absolute ethanol (80 mL). The mixture was stirred at room temperature, and potassium carbonate (11.2 g, 80.6 mmol, 5 equiv) with tetraethyl methylenediphosphonate (12.0 mL, 48.4 mmol, 3 equiv) was added subsequently. The mixture was heated at reflux until full conversion was achieved (ca. 1 h). The mixture was cooled to 0 °C; it was acidified with 1 M hydrochloric acid, extracted with chloroform (3×), washed with brine (1×), dried with MgSO4, filtered, and evaporated. The solid was adsorbed on silica gel in a mixture of acetone/cyclohexane, and it was purified by flash column chromatography on silica gel (cyclohexane to 1% of ethyl acetate modified with 10% of methanol (v/v_i_)). This separation provided an impure product containing tetraethyl methylenediphosphonate. This mixture was purified by flash chromatography on C18 silica gel (liquid injection in dimethylformamide, eluent water to methanol), yielding 5.39 g (88%) of the title product as a yellow solid. **^1^H NMR** (401 MHz, chloroform-*d*) δ 9.23 (s, 1H), 7.64 (d, *J* = 8.6 Hz, 1H), 7.54 (dd, *J* = 22.6, 17.3 Hz, 1H), 7.21 (d, *J* = 2.9 Hz, 1H), 6.68 (dd, *J* = 8.6, 2.9 Hz, 1H), 6.12 (dd, *J* = 19.6, 17.3 Hz, 1H), 4.29–4.06 (m, 4H), 1.37 (td, *J* = 7.1, 0.6 Hz, 6H). **^13^C NMR** (101 MHz, chloroform-*d*) δ 158.31, 152.31 (d, *J* = 6.9 Hz), 140.52, 138.11 (d, *J* = 23.5 Hz), 120.48, 115.74 (d, *J* = 192.6 Hz), 114.43, 87.79, 62.95 (d, *J* = 5.9 Hz), 16.49 (d, *J* = 6.5 Hz). **^31^P NMR** (162 MHz, chloroform-*d*) δ 20.49. **MS** (ESI-QMS) *m*/*z*: [M + H]^+^ calcd for C_12_H_17_IO_4_P, 383.0; found, 383.0.

##### Diethyl (*E*)-(2-Hydroxy-6-iodostyryl)phosphonate (**42c**)

Compound **34c** (1.5 g, 6.05 mmol, 1 equiv) and tetraethyl methylenediphosphonate (3 mL, 12.1 mmol, 2 equiv) were dissolved in dry dimethylformamide (30 mL) under an argon atmosphere. The solution was cooled to 0 °C. Sodium hydride (484 mg of 60% oil dispersion, 12.1 mmol, 2 equiv) was added at once, and the mixture was stirred at 40 °C overnight. The reaction was quenched with a half-saturated aqueous solution of NH_4_Cl, extracted with ethyl acetate (3×), washed with brine (1×), dried with MgSO_4_, filtered, and evaporated. The residue was adsorbed on silica gel in a mixture of cyclohexane/acetone. Purification by flash chromatography on silica gel (cyclohexane to 40% of ethyl acetate modified with 10% of methanol (v/v_i_)) afforded 1.2 g (52%) of the title compound as a clear oil. **^1^H NMR** (401 MHz, DMSO-*d*_6_) δ 10.70 (s, 1H), 7.43 (dd, *J* = 6.9, 1.9 Hz, 1H), 7.42 (dd, *J* = 24.2, 16.7 Hz, 1H), 6.96 (dd, *J* = 8.3, 1.5 Hz, 1H), 6.91 (dd, *J* = 8.2, 7.5 Hz, 1H), 6.69 (dd, *J* = 21.7, 17.5 Hz, 1H), 4.02 (dq, *J* = 8.3, 7.1 Hz, 4H), 1.27 (t, *J* = 7.1 Hz, 6H). **^13^C NMR** (101 MHz, DMSO-*d*_6_) δ 158.54–156.63 (m), 148.12 (d, *J* = 8.0 Hz), 131.89, 130.66, 123.24 (d, *J* = 22.1 Hz), 120.16 (d, *J* = 182.8 Hz), 116.74, 103.66, 61.31 (d, *J* = 5.6 Hz), 16.30 (d, *J* = 5.9 Hz). **^31^P NMR** (162 MHz, DMSO-*d*_6_) δ 21.53. **MS** (ESI-QMS) *m*/*z*: [M + H]^+^ calcd for C_12_H_17_IO_4_P, 383.0; found, 383.1.

#### General Procedures for Synthesis of Compounds **43a–l**

Corresponding starting compounds **42a–c** (500 mg, 1.31 mmol, 1 equiv) was dissolved in dry dimethylformamide (5 mL) under an argon atmosphere, and the mixture was cooled to 0 °C. Sodium hydride (79 mg of 60% oil dispersion, 1.96 mmol, 1.5 equiv) was added, and the mixture was stirred at room temperature for 30 min. An alkylating agent was added (2.62 mmol, 2 equiv), and the mixture was stirred at room temperature for 1 h. The reaction was quenched with a half-saturated aqueous solution of NH_4_Cl, extracted with ethyl acetate (3×), washed with brine (1×), dried with MgSO_4_, filtered, and evaporated. The residue was adsorbed on silica gel in a mixture of cyclohexane/acetone. Purification by flash chromatography on silica gel (cyclohexane to 40% of ethyl acetate modified with 10% of methanol (v/v_i_)) afforded a pure product.

##### Diethyl (*E*)-(2-Iodo-4-methoxystyryl)phosphonate (**43a**)

The general procedure with methyl iodide (163 μL) afforded 462 mg (89%) of the title compound as a clear oil. **^1^H NMR** (401 MHz, chloroform-d) δ 7.53 (dd, *J* = 22.4, 17.3 Hz, 1H), 7.41 (d, *J* = 8.7 Hz, 1H), 7.33 (d, *J* = 2.6 Hz, 1H), 6.85 (ddd, *J* = 8.7, 2.6, 0.7 Hz, 1H), 5.98 (dd, *J* = 18.0, 17.3 Hz, 1H), 4.09 (dq, *J* = 7.9, 7.1 Hz, 4H), 3.74 (s, 3H), 1.31 (td, *J* = 7.0, 0.5 Hz, 6H). **^13^C NMR** (101 MHz, chloroform-*d*) δ 160.86, 150.62 (d, *J* = 8.1 Hz), 130.43 (d, *J* = 23.7 Hz), 127.54, 124.58, 114.98, 114.78 (d, *J* = 192.0 Hz), 101.57, 61.95 (d, *J* = 5.6 Hz), 55.60, 16.43 (d, *J* = 6.5 Hz). **^31^P NMR** (162 MHz, chloroform-*d*) δ 21.03. **MS** (ESI-QMS) *m*/*z*: [M + H]^+^ calcd for C_13_H_19_IO_4_P, 397.0; found, 397.0.

##### Diethyl (*E*)-(2-Iodo-4-isopropoxystyryl)phosphonate (**43b**)

The general procedure with isopropyl iodide (261 μL) afforded 465 mg (84%) of the title compound as a yellowish oil. **^1^H NMR** (401 MHz, chloroform-*d*) δ 7.52 (dd, *J* = 22.4, 17.2 Hz, 1H), 7.39 (d, *J* = 8.8 Hz, 1H), 7.31 (d, *J* = 2.6 Hz, 1H), 6.80 (ddd, *J* = 8.8, 2.7, 0.7 Hz, 1H), 5.96 (dd, *J* = 18.0, 17.3 Hz, 1H), 4.47 (hept, *J* = 6.0 Hz, 1H), 4.08 (dq, *J* = 7.9, 7.1 Hz, 4H), 1.29 (td, *J* = 7.1, 0.6 Hz, 6H), 1.25 (d, *J* = 6.1 Hz, 6H). **^13^C NMR** (101 MHz, chloroform-*d*) δ 159.35, 150.71 (d, *J* = 8.0 Hz), 130.04 (d, *J* = 24.2 Hz), 127.56, 126.36, 116.15, 114.49 (d, *J* = 192.2 Hz), 101.67, 70.43, 61.93 (d, *J* = 5.8 Hz), 21.83, 16.43 (d, *J* = 6.5 Hz). **^31^P NMR** (162 MHz, chloroform-*d*) δ 21.06. **MS** (ESI-QMS) *m*/*z*: [M + H]^+^ calcd for C_15_H_23_IO_4_P, 425.0; found, 425.3.

##### Diethyl (*E*)-(2-Iodo-4-((2,3,4,5,6-pentafluorophenyl)oxy)styryl)phosphonate (**43c**)

The general procedure with hexafluorobenzene (151 μL) and heating at 70 °C afforded 530 mg (74%) of the title compound as a clear oil. **^1^H NMR** (401 MHz, chloroform-*d*) δ 7.59 (dd, *J* = 22.3, 17.3 Hz, 1H), 7.50 (d, *J* = 8.7 Hz, 1H), 7.44 (d, *J* = 2.6 Hz, 1H), 6.97 (dd, *J* = 8.7, 2.6 Hz, 1H), 6.10 (dd, *J* = 17.4 Hz, 1H), 4.16 (dq, *J* = 7.9, 7.1 Hz, 4H), 1.37 (t, *J* = 7.1 Hz, 6H). **^13^C NMR** (101 MHz, chloroform-*d*) δ 157.73, 150.02 (d, *J* = 7.9 Hz), 134.36 (d, *J* = 23.7 Hz), 128.18, 126.43, 117.52 (d, *J* = 191.5 Hz), 115.90, 100.99, 62.34 (d, *J* = 5.7 Hz), 16.59 (d, *J* = 6.6 Hz). **^19^F NMR** (377 MHz, chloroform-*d*) δ −153.37––153.56 (m), −157.93––158.31 (m), −160.86––161.12 (m). **^31^P NMR** (162 MHz, chloroform-*d*) δ 20.20. **MS** (ESI-QMS) *m*/*z*: [M + H]^+^ calcd for C_18_H_16_F_5_IO_4_P, 549.0; found, 549.2.

##### Diethyl (*E*)-(2-Iodo-4-((2,3,4,5,6-pentafluorophenyl)methoxy)styryl)phosphonate (**43d**)

The general procedure with 2,3,4,5,6-pentafluorobenzyl bromide (395 μL) and chromatography provided 700 mg (95%) of the title compound as a clear oil. **^1^H NMR** (401 MHz, chloroform-*d*) δ 7.59 (dd, *J* = 22.4, 17.3 Hz, 1H), 7.50 (d, *J* = 5.1 Hz, 1H), 7.49 (d, *J* = 1.0 Hz, 1H), 6.97 (ddd, *J* = 8.8, 2.6, 0.7 Hz, 1H), 6.07 (dd, *J* = 17.5 Hz, 1H), 5.12 (s, 2H), 4.16 (dq, *J* = 7.9, 7.1 Hz, 4H), 1.37 (td, *J* = 7.1, 0.5 Hz, 6H). **^13^C NMR** (101 MHz, chloroform-*d*) δ 159.15, 150.44 (d, *J* = 8.0 Hz), 147.58–146.63 (m), 144.97–144.02 (m), 139.40–138.40 (m), 131.99 (d, *J* = 24.1 Hz), 127.91, 125.69, 116.01 (d, *J* = 191.7 Hz), 115.61, 109.86–108.61 (m), 101.49, 62.19 (d, *J* = 5.4 Hz), 57.77, 16.59 (d, *J* = 6.5 Hz). **^19^F NMR** (377 MHz, chloroform-*d*) δ −138.39––143.87 (m), −147.08––153.58 (m), −158.12––164.61 (m). ^31^P NMR (162 MHz, chloroform-*d*) δ 20.63. **MS** (ESI-QMS) *m*/*z*: [M + H]^+^ calcd for C_19_H_18_F_5_IO_4_P, 563.0; found, 563.2.

##### Diethyl (*E*)-(2-Iodo-5-methoxystyryl)phosphonate (**43e**)

The general procedure with methyl iodide (163 μL) afforded 326 mg (63%) of the title compound as a yellowish oil. **^1^H NMR** (401 MHz, chloroform-*d*) δ 7.63 (d, *J* = 8.7 Hz, 1H), 7.50 (dd, *J* = 22.2, 17.3 Hz, 1H), 6.98 (d, *J* = 3.0 Hz, 1H), 6.58 (dd, *J* = 8.7, 3.0 Hz, 1H), 6.08 (dd, *J* = 18.0, 17.3 Hz, 1H), 4.09 (dq, *J* = 8.0, 7.1 Hz, 4H), 3.72 (s, 3H), 1.30 (t, *J* = 7.0 Hz, 6H). **^13^C NMR** (101 MHz, chloroform-*d*) δ 159.97, 150.94 (d, *J* = 7.4 Hz), 140.22, 138.82 (d, *J* = 23.5 Hz), 117.75 (d, *J* = 190.1 Hz), 117.69, 112.53 (d, *J* = 1.6 Hz), 89.12, 62.04 (d, *J* = 5.7 Hz), 55.41, 16.39 (d, *J* = 6.6 Hz). **^31^P NMR** (162 MHz, chloroform-*d*) δ 20.12. **MS** (ESI-QMS) *m*/*z*: [M + H]^+^ calcd for C_13_H_19_IO_4_P, 397.0; found, 397.1.

##### Diethyl (*E*)-(2-Iodo-5-isopropoxystyryl)phosphonate (**43f**)

The general procedure with isopropyl iodide (261 μL) afforded 402 mg (72%) of the title compound as a yellowish oil. **^1^H NMR** (401 MHz, chloroform-*d*) δ 7.56 (d, *J* = 8.7 Hz, 1H), 7.45 (dd, *J* = 22.2, 17.3 Hz, 1H), 6.93 (d, *J* = 3.0 Hz, 1H), 6.51 (dd, *J* = 8.7, 3.0 Hz, 1H), 6.01 (dd, *J* = 18.0, 17.3 Hz, 1H), 4.40 (hept, *J* = 6.0 Hz, 1H), 4.03 (dq, *J* = 8.0, 7.1 Hz, 4H), 1.24 (td, *J* = 7.1, 0.6 Hz, 6H), 1.18 (d, *J* = 6.0 Hz, 6H). **^13^C NMR** (101 MHz, chloroform-*d*) δ 158.12, 150.87 (d, *J* = 7.8 Hz), 140.09, 138.71 (d, *J* = 23.5 Hz), 119.05, 117.45 (d, *J* = 190.1 Hz), 114.57 (d, *J* = 1.4 Hz), 88.62, 70.02, 61.87 (d, *J* = 5.8 Hz), 21.66, 16.26 (d, *J* = 6.5 Hz). **^31^P NMR** (162 MHz, chloroform-*d*) δ 20.13. **MS** (ESI-QMS) *m*/*z*: [M + H]^+^ calcd for C_15_H_23_IO_4_P, 425.0; found, 425.2.

##### Diethyl (*E*)-(2-Iodo-5-((2,3,4,5,6-pentafluorophenyl)oxy)styryl)phosphonate (**43g**)

The general procedure with hexafluorobenzene (151 μL) and heating at 70 °C afforded 300 mg (42%) of the title compound as a clear oil. **^1^H NMR** (401 MHz, chloroform-*d*) δ 7.75 (d, *J* = 8.7 Hz, 1H), 7.50 (dd, *J* = 22.1, 17.3 Hz, 1H), 7.07 (d, *J* = 3.0 Hz, 1H), 6.65 (dd, *J* = 8.7, 3.0 Hz, 1H), 6.07 (dd, *J* = 34.8, 17.4 Hz, 1H), 4.12 (dq, *J* = 8.0, 7.1 Hz, 4H), 1.32 (td, *J* = 7.0, 0.6 Hz, 6H). **^13^C NMR** (101 MHz, chloroform-*d*) δ 157.56, 149.94 (d, *J* = 7.7 Hz), 143.53–142.68 (m), 140.94, 139.94 (d, *J* = 23.7 Hz), 138.30–137.58 (m), 137.44–136.53 (m), 129.22–128.40 (m), 119.29 (d, *J* = 190.2 Hz), 118.29, 114.17 (d, *J* = 1.9 Hz), 93.06, 62.27 (d, *J* = 5.6 Hz), 16.41 (d, *J* = 6.4 Hz). **^19^F NMR** (377 MHz, chloroform-*d*) δ −151.86––154.36 (m), −156.40––159.84 (m), −159.84––162.90 (m). **^31^P NMR** (162 MHz, chloroform-*d*) δ 19.40. **MS** (ESI-QMS) *m*/*z*: [M + H]^+^ calcd for C_18_H_16_F_5_IO_4_P, 549.0; found, 549.1.

##### Diethyl (*E*)-(2-Iodo-5-((2,3,4,5,6-pentafluorophenyl)methoxy)styryl)phosphonate (**43h**)

The general procedure with 2,3,4,5,6-pentafluorobenzyl bromide (395 μL) and chromatography afforded 675 mg (92%) of the title compound as a clear oil. **^1^H NMR** (401 MHz, chloroform-*d*) δ 7.63 (d, *J* = 8.7 Hz, 1H), 7.46 (dd, *J* = 22.2, 17.3 Hz, 1H), 7.06 (d, *J* = 3.0 Hz, 1H), 6.64 (dd, *J* = 8.7, 3.0 Hz, 1H), 6.09 (dd, *J* = 17.4 Hz, 1H), 5.04 (s, 2H), 4.07 (dq, *J* = 8.1, 7.1 Hz, 4H), 1.27 (t, *J* = 7.1 Hz, 6H). **^13^C NMR** (101 MHz, chloroform-*d*) δ 158.27, 150.45 (d, *J* = 7.5 Hz), 147.19–146.31 (m), 144.87–143.92 (m), 143.47–142.27 (m), 140.42, 139.10 (d, *J* = 23.5 Hz), 118.25 (d, *J* = 189.9 Hz), 118.13, 113.35, 110.60–108.34 (m), 90.43, 62.02 (d, *J* = 5.5 Hz), 57.50, 16.23 (d, *J* = 6.5 Hz). **^19^F NMR** (377 MHz, chloroform-*d*) δ −138.86––143.87 (m), −149.90––153.34 (m), −159.37––163.83 (m). **^31^P NMR** (162 MHz, chloroform-*d*) δ 19.75. **MS** (ESI-QMS) *m*/*z*: [M + H]^+^ calcd for C_19_H_18_F_5_IO_4_P, 563.0; found, 563.0.

##### Diethyl (*E*)-(2-Iodo-6-methoxystyryl)phosphonate (**43i**)

The general procedure with methyl iodide (163 μL) afforded 483 mg (93%) of the title compound as a yellowish oil. **^1^H NMR** (401 MHz, chloroform-*d*) δ 7.49 (dd, *J* = 7.6, 1.3 Hz, 1H), 7.47 (dd, *J* = 25.0, 17.7 Hz, 1H), 6.92 (dd, *J* = 8.2, 7.8 Hz, 1H), 6.86 (dd, *J* = 8.4, 1.3 Hz, 1H), 6.54 (dd, *J* = 21.3, 17.6 Hz, 1H), 4.13 (dq, *J* = 7.9, 7.1 Hz, 4H), 3.80 (s, 3H), 1.34 (t, *J* = 7.1 Hz, 6H). **^13^C NMR** (101 MHz, chloroform-*d*) δ 158.54 (d, *J* = 2.1 Hz), 148.06 (d, *J* = 7.5 Hz), 132.32, 131.19, 126.54 (d, *J* = 23.0 Hz), 121.16 (d, *J* = 184.3 Hz), 111.36, 102.64, 61.96 (d, *J* = 5.3 Hz), 55.74, 16.50 (d, *J* = 6.5 Hz). **^31^P NMR** (162 MHz, chloroform-*d*) δ 20.51. **MS** (ESI-QMS) *m*/*z*: [M + H]^+^ calcd for C_13_H_19_IO_4_P, 397.0; found, 369.9.

##### Diethyl (*E*)-(2-Iodo-6-isopropoxystyryl)phosphonate (**43j**)

The general procedure with isopropyl iodide (261 μL) afforded 488 mg (88%) of the title compound as a yellowish oil. **^1^H NMR** (401 MHz, chloroform-*d*) δ 7.46 (dd, *J* = 6.3, 2.6 Hz, 1H), 7.45 (dd, *J* = 24.9, 17.6 Hz, 1H), 6.92–6.84 (m, 2H), 6.53 (dd, *J* = 21.6, 17.6 Hz, 1H), 4.56 (hept, *J* = 6.1 Hz, 1H), 4.21–4.06 (m, 4H), 1.39–1.29 (m, 12H). **^13^C NMR** (101 MHz, chloroform-*d*) δ 156.79 (d, *J* = 2.1 Hz), 148.46 (d, *J* = 8.0 Hz), 132.03, 131.01, 127.50 (d, *J* = 22.6 Hz), 121.08 (d, *J* = 183.5 Hz), 113.75, 102.63, 71.33, 61.94 (d, *J* = 5.2 Hz), 22.05, 16.53 (d, *J* = 6.6 Hz). **^31^P NMR** (162 MHz, chloroform-*d*) δ 20.55. **MS** (ESI-QMS) *m*/*z*: [M + H]^+^ calcd for C_15_H_23_IO_4_P, 425.0; found, 425.0.

##### Diethyl (*E*)-(2-Iodo-6-((2,3,4,5,6-pentafluorophenyl)oxy)styryl)phosphonate (**43k**)

The general procedure with hexafluorobenzene (151 μL) and overnight heating at 70 °C afforded 356 mg (50%) of the title compound as a clear oil. **^1^H NMR** (401 MHz, chloroform-*d*) δ 7.62 (dd, *J* = 7.9, 1.0 Hz, 1H), 7.44 (dd, *J* = 24.1, 17.7 Hz, 1H), 6.87 (dd, *J* = 8.1 Hz, 1H), 6.61 (d, *J* = 7.9 Hz, 1H), 6.40 (dd, *J* = 19.6, 17.7 Hz, 1H), 4.11 (dq, *J* = 8.1, 7.1 Hz, 4H), 1.30 (td, *J* = 7.0, 0.6 Hz, 6H). **^13^C NMR** (101 MHz, chloroform-*d*) δ 154.61, 146.20 (d, *J* = 7.4 Hz), 135.43, 130.98, 128.21 (d, *J* = 23.5 Hz), 123.28 (d, *J* = 184.7 Hz), 113.99, 101.47 (d, *J* = 1.9 Hz), 62.15 (d, *J* = 5.3 Hz), 16.33 (d, *J* = 6.3 Hz). **^19^F NMR** (377 MHz, chloroform-*d*) δ −152.64––156.08 (m), −157.10––159.37 (m), −159.37––166.65 (m). **^31^P NMR** (162 MHz, chloroform-*d*) δ 19.80. **MS** (ESI-QMS) *m*/*z*: [M + H]^+^ calcd for C_18_H_16_F_5_IO_4_P, 549.0; found, 549.2.

##### Diethyl (*E*)-(2-Iodo-6-((2,3,4,5,6-pentafluorophenyl)methoxy)styryl)phosphonate (**43l**)

The general procedure with 2,3,4,5,6-pentafluorobenzyl bromide (395 μL) afforded 668 mg (91%) of the title compound as a clear oil. **^1^H NMR** (401 MHz, chloroform-*d*) δ 7.65–7.53 (m, 1H), 7.37 (ddd, *J* = 24.5, 17.6, 2.0 Hz, 1H), 7.03 (d, *J* = 8.4 Hz, 1H), 6.97 (dd, *J* = 8.0, 1.5 Hz, 1H), 6.29 (ddd, *J* = 20.4, 17.6, 1.7 Hz, 1H), 5.14 (d, *J* = 1.6 Hz, 2H), 4.21–3.95 (m, 4H), 1.29 (td, *J* = 7.1, 1.6 Hz, 6H). **^13^C NMR** (101 MHz, chloroform-*d*) δ 156.23, 147.38 (d, *J* = 7.7 Hz), 147.25–146.51 (m), 145.30–144.00 (m), 136.77–135.83 (m), 133.59, 131.12, 128.10 (d, *J* = 24.2 Hz), 122.08 (d, *J* = 183.1 Hz), 112.82, 110.47–108.60 (m), 102.16, 61.95 (d, *J* = 5.2 Hz), 58.18, 16.38 (d, *J* = 6.5 Hz). **^19^F NMR** (377 MHz, chloroform-*d*) δ −138.82––144.30 (m), −147.51––154.01 (m), −158.55––165.05 (m). **^31^P NMR** (162 MHz, chloroform-*d*) δ 20.15. **MS** (ESI-QMS) *m*/*z*: [M + H]^+^ calcd for C_19_H_18_F_5_IO_4_P, 563.0; found, 563.1.

#### General Procedures for Synthesis of Compounds **45a–q** and **49a–b**

Step 1: compound **9** (100 mg, 0.258 mmol, 1 equiv) with copper(I) iodide (5 mg, 0.026 mmol, 0.1 equiv) were charged into a microwave reactor tube, and the tube was flushed with argon. Dry dimethylformamide (2 mL), an aryl iodide (0.310 mmol, 1.2 equiv), triethylamine (54 μL, 0.387 mmol, 1.5 equiv), and 2-isobutyrylcyclohexanone (9 μL, 0.052 mmol, 0.2 equiv) were added subsequently, and the mixture was heated in the microwave reactor at 100 °C for 2–3 h. The mixture was dissolved in chloroform, washed with a half-saturated aqueous solution of NaHCO_3_ (2×), 1 M HCl (aq) (2×), and brine (1×), dried with MgSO_4_, filtered, and evaporated. The solid was adsorbed on silica gel in a mixture of cyclohexane/acetone and purified by flash chromatography on silica gel (cyclohexane to 30% of ethyl acetate modified with 10% of methanol (v/v_i_)), yielding a coupling intermediate **44a–g** as an oil. The compound was used in the next step without further characterization. Step 2: the intermediate **44a–g** was dissolved in trifluoroacetic acid (2 mL/100 mg of the material) at room temperature and stirred for 15 min. Trifluoroacetic acid was evaporated and co-evaporated with water (2×) and a cold solution of ammonia in ethanol (2 mL/100 mg of the material). The mixture was evaporated to dryness. Step 3: the flask was sealed with a septum and flushed with argon, and then dry pyridine (10 mL/1 mmol of the starting compound) was added followed by trimethylsilyl bromide (1 mL/1 mmol of the starting compound). The mixture was stirred at room temperature overnight, and then it was evaporated to dryness. The residue was dissolved in a 2 M aq solution of triethylammonium bicarbonate and a small amount of methanol, and it was evaporated to dryness. The solid residue was dissolved in a small amount of water (solubility can be enhanced by the addition of several drops of aqueous ammonia) and purified by HPLC (C18, gradient H_2_O/MeOH). Product-containing fractions were filtered through a short pad of Dowex 50 in Na^+^-cycle, and solvents were evaporated. The purified product was lyophilized from water. NMR samples were prepared in a mixture of deuterium oxide and dimethyl sulfoxide-*d6* (1:1), locked on the deuterium oxide signal, and referenced on the dimethyl sulfoxide signal.

##### Sodium (*E*)-7-((5-Hydroxy-2-(2-(phosphonato)vinyl)phenyl)thio)-3,5-dihydro-4*H*-pyrrolo[3,2-*d*]pyrimidin-4-one (**45a**)

The general procedure starting from 200 mg of compound **9** and compound **42a** (198 mg) afforded 100 mg (30%) of the coupling intermediate and 15 mg (23%) of the title compound as a white solid. **^1^H NMR** (401 MHz, deuterium oxide) δ 8.10 (s, 1H), 7.76 (s, 1H), 7.56 (dd, *J* = 19.0, 17.3 Hz, 1H), 7.44 (d, *J* = 8.6 Hz, 1H), 6.45 (ddd, *J* = 8.6, 2.6, 0.6 Hz, 1H), 6.25 (dd, *J* = 17.2, 14.3 Hz, 1H), 6.24 (d, *J* = 2.6 Hz, 1H). **^13^C NMR** (101 MHz, deuterium oxide) δ 166.50, 163.91, 152.70, 148.30, 139.05, 135.09 (d, *J* = 4.3 Hz), 128.81, 125.70 (d, *J* = 171.5 Hz), 124.18 (d, *J* = 20.2 Hz), 120.97, 117.99, 117.66, 102.13. **^31^P NMR** (162 MHz, deuterium oxide) δ 16.37. **HRMS** (ESI-FTMS) *m*/*z* for the acid: [M – H]^−^ calcd for C_14_H_11_O_5_N_3_PS, 364.0163; found, 364.0157.

##### Sodium (*E*)-7-((5-Methoxy-2-(2-(phosphonato)vinyl)phenyl)thio)-3,5-dihydro-4*H*-pyrrolo[3,2-*d*]pyrimidin-4-one (**45b**)

The general procedure with compound **43a** (123 mg) afforded 104 mg (62%) of the coupling intermediate and 27 mg (59%) of the title compound as a white solid. **^1^H NMR** (401 MHz, DMSO-*d*_6_) δ 7.84 (s, 1H), 7.54 (s, 1H), 7.42 (d, *J* = 8.6 Hz, 1H), 7.30 (dd, *J* = 18.9, 17.2 Hz, 1H), 6.63 (dd, *J* = 8.7, 2.7 Hz, 1H), 6.22 (dd, *J* = 17.2, 13.6 Hz, 1H), 6.16 (d, *J* = 2.6 Hz, 1H), 3.48 (s, 3H). **^13^C NMR** (101 MHz, DMSO-*d*_6_) δ 162.68, 159.96, 151.23, 147.81, 140.56, 134.39, 133.27, 130.06 (d, *J* = 167.9 Hz), 128.61, 121.22, 113.34, 111.21, 111.12, 100.56, 55.98. **^31^P NMR** (162 MHz, deuterium oxide) δ 13.38. **HRMS** (ESI-FTMS) *m*/*z* for the acid: [M – H]^−^ calcd for C_15_H_13_O_5_N_3_PS, 378.0319; found, 378.0315.

##### Sodium (*E*)-7-((5-Isopropoxy-2-(2-(phosphonato)vinyl)phenyl)thio)-3,5-dihydro-4*H*-pyrrolo[3,2-*d*]pyrimidin-4-one (**45c**)

The general procedure with compound **43b** (131 mg) afforded 170 mg (50%) of the coupling intermediate and 32 mg (28%) of the title compound as a white solid. **^1^H NMR** (401 MHz, DMSO-*d*_6_) δ 7.89 (s, 1H), 7.54 (s, 1H), 7.40 (d, *J* = 8.5 Hz, 1H), 7.28 (dd, *J* = 18.7, 17.3 Hz, 1H), 6.56 (dd, *J* = 8.6, 2.6 Hz, 1H), 6.23 (dd, *J* = 17.2, 13.9 Hz, 1H), 6.12 (d, *J* = 2.6 Hz, 1H), 4.12 (hept, *J* = 6.1 Hz, 1H), 0.93 (d, *J* = 6.0 Hz, 6H). **^13^C NMR** (101 MHz, DMSO-*d*_6_) δ 164.88, 158.53, 153.77, 148.76, 140.82, 135.30, 134.17 (d, *J* = 5.8 Hz), 130.09 (d, *J* = 155.5 Hz), 129.63 (d, *J* = 20.2 Hz), 129.19, 121.65, 114.97, 114.38, 100.94, 72.40, 22.56. **^31^P NMR** (162 MHz, deuterium oxide) δ 13.38. **HRMS** (ESI-FTMS) *m*/*z* for the acid: [M – H]^−^ calcd for C_17_H_17_O_5_N_3_PS, 406.0632; found, 406.0628.

##### Sodium (*E*)-7-((5-(2,3,4,5,6-Pentafluorophenyl)oxy-2-(2-(phosphonato)vinyl)phenyl)thio)-3,5-dihydro-4*H*-pyrrolo[3,2-*d*]pyrimidin-4-one (**45d**)

The general procedure with compound **43c** (170 mg) afforded 126 mg (78%) of the coupling intermediate and 9 mg (12%) of the title compound as a white solid. **^1^H NMR** (500 MHz, deuterium oxide) δ 7.95 (d, *J* = 0.7 Hz, 1H), 7.66 (d, *J* = 8.6 Hz, 1H), 7.59–7.52 (m, 1H), 7.42 (t, *J* = 17.9 Hz, 1H), 6.94 (dd, *J* = 8.6, 2.8 Hz, 1H), 6.50 (dd, *J* = 17.2, 14.3 Hz, 1H), 6.07 (d, *J* = 2.2 Hz, 1H). **^13^C NMR** (126 MHz, deuterium oxide) δ 165.44, 158.15, 152.47, 149.17, 142.74, 138.11, 133.53 (d, *J* = 5.6 Hz), 131.44 (d, *J* = 20.1 Hz), 131.22 (d, *J* = 168.0 Hz), 129.48, 124.10, 113.67, 112.05, 97.73. **^19^F NMR** (377 MHz, deuterium oxide) δ −155.14 (d, *J* = 18.8 Hz), −159.87 (t, *J* = 22.1 Hz), −162.17––162.35 (m). **^31^P NMR** (162 MHz, deuterium oxide) δ 13.42. **HRMS** (ESI-FTMS) *m*/*z* for the acid: [M – H]^−^ calcd for C_20_H_10_O_5_N_3_F_5_PS, 530.0004; found, 530.0000.

##### Sodium (*E*)-7-((5-(2,3,4,5,6-Pentafluorophenyl)methoxy-2-(2-(phosphonato)vinyl)phenyl)thio)-3,5-dihydro-4*H*-pyrrolo[3,2-*d*]pyrimidin-4-one (**45e**)

The general procedure with compound **43d** (174 mg) afforded 153 mg (72%) of the coupling intermediate and 9 mg (8%) of the title compound as a white solid. **^1^H NMR** (500 MHz, deuterium oxide) δ 7.98 (s, 1H), 7.71 (s, 1H), 7.69–7.59 (m, 1H), 7.53 (d, *J* = 8.6 Hz, 1H), 6.76 (d, *J* = 8.7 Hz, 1H), 6.38 (dd, *J* = 16.7 Hz, 1H), 6.23 (s, 1H), 4.97 (s, 3H). **^13^C NMR** (126 MHz, deuterium oxide) δ 158.96, 155.94, 148.25–146.09 (m), 146.99, 145.39–144.93 (m), 144.04, 140.31, 139.46–138.73 (m), 137.58 (d, *J* = 6.4 Hz), 136.63, 129.25 (d, *J* = 21.8 Hz), 129.11, 124.60 (d, *J* = 177.4 Hz), 119.64, 114.30, 113.50, 111.43–110.01 (m), 103.08, 59.01. **^19^F NMR** (377 MHz, deuterium oxide) δ −139.07––145.81 (m), −153.48 (t, *J* = 20.9 Hz), −162.37––162.67 (m). **^31^P NMR** (162 MHz, deuterium oxide) δ 13.41. **HRMS** (ESI-FTMS) *m*/*z* for the acid: [M – H]^−^ calcd for C_21_H_12_O_5_N_3_F_5_PS, 544.0161; found, 544.0154. LC/MS purity 93% (at 254 nm).

##### Sodium (*E*)-7-((4-Hydroxy-2-(2-(phosphonato)vinyl)phenyl)thio)-3,5-dihydro-4*H*-pyrrolo[3,2-*d*]pyrimidin-4-one (**45f**)

The general procedure with compound **42b** (99 mg) afforded 120 mg (73%) of the coupling intermediate and 44 mg (58%) of the title compound as a white-off solid. **^1^H NMR** (401 MHz, deuterium oxide) δ 8.06 (s, 1H), 7.83 (s, 1H), 7.71 (dd, *J* = 18.1 Hz, 1H), 7.10 (d, *J* = 2.7 Hz, 1H), 7.08 (d, *J* = 8.7 Hz, 1H), 6.65 (dd, *J* = 8.6, 2.7 Hz, 1H), 6.47 (dd, *J* = 17.3, 14.1 Hz, 1H). **^13^C NMR** (101 MHz, deuterium oxide) δ 156.75, 156.69, 147.08, 144.66, 139.97 (d, *J* = 20.3 Hz), 136.55, 136.19 (d, *J* = 5.3 Hz), 133.44, 131.15 (d, *J* = 169.1 Hz), 127.81, 119.43, 117.82, 114.33, 106.50. **^31^P NMR** (162 MHz, deuterium oxide) δ 12.90. **HRMS** (ESI-FTMS) *m*/*z* for the acid: [M – H]^−^ calcd for C_14_H_11_O_5_N_3_PS, 364.0163; found, 364.0159.

##### Sodium (*E*)-7-((4-Methoxy-2-(2-(phosphonato)vinyl)phenyl)thio)-3,5-dihydro-4*H*-pyrrolo[3,2-*d*]pyrimidin-4-one (**45g**)

The general procedure with compound **43e** (123 mg) afforded 160 mg (95%) of the coupling intermediate and 51 mg (49%) of the title compound as a white solid. **^1^H NMR** (401 MHz, deuterium oxide) δ 8.05 (s, 1H), 7.83 (s, 1H), 7.69 (dd, *J* = 18.1 Hz, 1H), 7.20 (d, *J* = 2.9 Hz, 1H), 7.01 (d, *J* = 8.7 Hz, 1H), 6.67 (dd, *J* = 8.8, 2.8 Hz, 1H), 6.54 (dd, *J* = 17.3, 13.9 Hz, 1H), 3.80 (s, 3H). **^13^C NMR** (101 MHz, deuterium oxide) δ 159.32, 156.86, 147.20, 144.87, 139.47 (d, *J* = 19.9 Hz), 136.70, 135.78 (d, *J* = 5.9 Hz), 132.38, 131.84 (d, *J* = 168.1 Hz), 129.16, 119.55, 116.30, 112.80, 105.80, 56.89. **^31^P NMR** (162 MHz, deuterium oxide) δ 12.74. **HRMS** (ESI-FTMS) *m*/*z* for the acid: [M – H]^−^ calcd for C_15_H_13_O_5_N_3_PS, 378.0319; found, 378.0315.

##### Sodium (*E*)-7-((4-Isopropoxy-2-(2-(phosphonato)vinyl)phenyl)thio)-3,5-dihydro-4*H*-pyrrolo[3,2-*d*]pyrimidin-4-one (**45h**)

The general procedure with compound **43f** (131 mg) afforded 150 mg (85%) of the coupling intermediate and 38 mg (38%) of the title compound as a white-off solid. **^1^H NMR** (401 MHz, deuterium oxide) δ 8.04 (s, 1H), 7.81 (s, 1H), 7.80 (dd, *J* = 20.2, 17.3 Hz, 1H), 7.15 (d, *J* = 2.8 Hz, 1H), 7.06 (d, *J* = 8.7 Hz, 1H), 6.64 (dd, *J* = 8.8, 2.8 Hz, 1H), 6.48 (dd, *J* = 17.3, 15.4 Hz, 1H), 4.56 (hept, *J* = 6.1 Hz, 1H), 1.27 (d, *J* = 6.2 Hz, 6H). **^13^C NMR** (101 MHz, deuterium oxide) δ 157.50, 156.34, 147.06, 144.42, 139.01 (d, *J* = 21.0 Hz), 138.17 (d, *J* = 6.4 Hz), 136.61, 133.03, 129.56, 128.57 (d, *J* = 173.6 Hz), 119.43, 118.67, 115.33, 106.07, 72.99, 22.62. **^31^P NMR** (162 MHz, deuterium oxide) δ 13.70. **HRMS** (ESI-FTMS) *m*/*z* for the acid: [M – H]^−^ calcd for C17H17O5N3PS, 406.0632; found, 406.0627.

##### Sodium (*E*)-7-((4-(2,3,4,5,6-Pentafluorophenyl)oxy-2-(2-(phosphonato)vinyl)phenyl)thio)-3,5-dihydro-4*H*-pyrrolo[3,2-*d*]pyrimidin-4-one (**45i**)

The general procedure with compound **43 g** (174 mg) afforded 169 mg (81%) of the coupling intermediate and 70 mg (58%) of the title compound as a white-off solid. **^1^H NMR** (401 MHz, deuterium oxide) δ 8.04 (s, 1H), 7.83 (s, 1H), 7.61 (dd, *J* = 17.9 Hz, 1H), 7.26 (d, *J* = 2.8 Hz, 1H), 6.89 (d, *J* = 8.8 Hz, 1H), 6.63 (dd, *J* = 8.8, 2.9 Hz, 1H), 6.48 (dd, *J* = 17.3, 13.6 Hz, 1H). **^13^C NMR** (101 MHz, deuterium oxide) δ 157.01, 147.34, 145.17, 139.43 (d, *J* = 20.2 Hz), 136.97, 134.73 (d, *J* = 6.2 Hz), 133.14 (d, *J* = 167.1 Hz), 132.97, 131.24, 119.78, 116.67, 114.54, 104.52. **^19^F NMR** (377 MHz, deuterium oxide) δ −155.26 (d, *J* = 18.8 Hz), −159.99 (t, *J* = 22.1 Hz), −162.29––162.47 (m). **^31^P NMR** (162 MHz, deuterium oxide) δ 12.22. **HRMS** (ESI-FTMS) *m*/*z* for the acid: [M – H]^−^ calcd for C_20_H_10_O_5_N_3_F_5_PS, 530.0004; found, 529.9999.

##### Sodium (*E*)-7-((4-(2,3,4,5,6-Pentafluorophenyl)methoxy-2-(2-(phosphonato)vinyl)phenyl)thio)-3,5-dihydro-4*H*-pyrrolo[3,2-*d*]pyrimidin-4-one (**45j**)

The general procedure with compound **43h** (174 mg) afforded 147 mg (69%) of the coupling intermediate and 56 mg (54%) of the title compound as a white-off solid. **^1^H NMR** (401 MHz, deuterium oxide) δ 8.05 (s, 1H), 7.83 (s, 1H), 7.67 (dd, *J* = 18.1 Hz, 1H), 7.30 (d, *J* = 2.8 Hz, 1H), 6.98 (d, *J* = 8.7 Hz, 1H), 6.69 (dd, *J* = 8.8, 2.8 Hz, 1H), 6.55 (dd, *J* = 17.3, 13.7 Hz, 1H), 5.23 (s, 2H). **^13^C NMR** (101 MHz, deuterium oxide) δ 157.67, 157.18, 147.32, 145.30, 139.47 (d, *J* = 19.8 Hz), 136.76, 135.29 (d, *J* = 5.7 Hz), 132.40 (d, *J* = 167.4 Hz), 131.97, 130.71, 119.68, 117.38, 114.08, 105.26, 59.58. **^19^F NMR** (377 MHz, deuterium oxide) δ −143.07 (dd, *J* = 23.0, 8.3 Hz), −153.03 (t, *J* = 20.9 Hz), −161.92––162.22 (m). **^31^P NMR** (162 MHz, deuterium oxide) δ 12.51. **HRMS** (ESI-FTMS) *m*/*z* for the acid: [M – H]^−^ calcd for C_21_H_12_O_5_N_3_F_5_PS, 544.0161; found, 544.0155.

##### Sodium (*E*)-7-((3-Hydroxy-2-(2-(phosphonato)vinyl)phenyl)thio)-3,5-dihydro-4*H*-pyrrolo[3,2-*d*]pyrimidin-4-one (**45k**)

The general procedure with compound **42c** (99 mg) afforded 100 mg (60%) of the coupling intermediate and 10 mg (16%) of the title compound as a white solid. **^1^H NMR** (401 MHz, deuterium oxide) δ 8.07 (s, 1H), 7.84 (s, 1H), 7.25 (dd, *J* = 20.1, 17.9 Hz, 1H), 6.96 (dd, *J* = 8.1 Hz, 1H), 6.73 (dd, *J* = 8.1, 1.1 Hz, 1H), 6.58 (dd, *J* = 17.9, 15.7 Hz, 1H), 6.36 (dd, *J* = 8.0, 1.1 Hz, 1H). **^13^C NMR** (101 MHz, deuterium oxide) δ 156.90, 155.60, 147.56, 145.09, 140.08, 137.08, 136.14 (d, *J* = 165.9 Hz), 132.47 (d, *J* = 5.2 Hz), 130.04, 124.52 (d, *J* = 19.6 Hz), 119.87, 119.31, 114.67, 104.19. **^31^P NMR** (162 MHz, deuterium oxide) δ 12.72. **HRMS** (ESI-FTMS) *m*/*z* for the acid: [M – H]^−^ calcd for C_14_H_11_O_5_N_3_PS, 364.0163; found, 364.0159.

##### Sodium (*E*)-7-((3-Methoxy-2-(2-(phosphonato)vinyl)phenyl)thio)-3,5-dihydro-4*H*-pyrrolo[3,2-*d*]pyrimidin-4-one (**45l**)

The general procedure with compound **43i** (123 mg) afforded 118 mg (70%) of the coupling intermediate and 39 mg (51%) of the title compound as a white solid. **^1^H NMR** (401 MHz, deuterium oxide) δ 8.07 (s, 1H), 7.87 (s, 1H), 7.51 (dd, *J* = 22.4, 17.8 Hz, 1H), 7.13 (dd, *J* = 8.2 Hz, 1H), 6.92 (d, *J* = 8.3 Hz, 1H), 6.71 (dd, *J* = 17.9 Hz, 1H), 6.51 (d, *J* = 8.0 Hz, 1H), 3.90 (s, 3H). **^13^C NMR** (101 MHz, deuterium oxide) δ 159.29, 156.21, 147.44, 144.51, 140.42, 137.02, 134.98 (d, *J* = 5.9 Hz), 131.39 (d, *J* = 173.0 Hz), 130.61, 124.35 (d, *J* = 20.9 Hz), 120.41, 119.79, 110.27, 104.33, 57.15. **^31^P NMR** (162 MHz, deuterium oxide) δ 13.13. **HRMS** (ESI-FTMS) *m*/*z* for the acid: [M – H]^−^ calcd for C_15_H_13_O_5_N_3_PS, 378.0319; found, 378.0311.

##### Sodium (*E*)-7-((3-Isopropoxy-2-(2-(phosphonato)vinyl)phenyl)thio)-3,5-dihydro-4*H*-pyrrolo[3,2-*d*]pyrimidin-4-one (**45m**)

The general procedure with compound **43j** (131 mg) afforded 160 mg (91%) of the coupling intermediate and 34 mg (32%) of the title compound as a white solid. **^1^H NMR** (500 MHz, deuterium oxide) δ 8.04 (s, 1H), 7.79 (s, 1H), 7.48 (dd, *J* = 22.6, 17.8 Hz, 1H), 7.05 (dd, *J* = 8.2 Hz, 1H), 6.91 (d, *J* = 8.2 Hz, 1H), 6.70 (dd, *J* = 18.1 Hz, 1H), 6.49 (d, *J* = 8.1 Hz, 1H), 4.68–4.58 (m, 1H), 1.35 (d, *J* = 6.1 Hz, 6H). **^13^C NMR** (126 MHz, deuterium oxide) δ 157.30, 156.62, 147.37, 144.61, 140.39, 137.05, 136.18 (d, *J* = 6.3 Hz), 130.71 (d, *J* = 173.7 Hz), 130.49, 129.71, 126.52 (d, *J* = 19.2 Hz), 121.04, 119.85, 115.00, 104.21, 74.90, 22.67. **^31^P NMR** (162 MHz, deuterium oxide) δ 13.16. **HRMS** (ESI-FTMS) *m*/*z* for the acid: [M – H]^−^ calcd for C_17_H_17_O_5_N_3_PS, 406.0632; found, 406.0628.

##### Sodium (*E*)-7-((3-(2,3,4,5,6-Pentafluorophenyl)oxy-2-(2-(phosphonato)vinyl)phenyl)thio)-3,5-dihydro-4*H*-pyrrolo[3,2-*d*]pyrimidin-4-one (**45n**)

The general procedure with compound **43k** (174 mg) afforded 175 mg (84%) of the coupling intermediate and 78 mg (62%) of the title compound as a white-off solid. **^1^H NMR** (401 MHz, deuterium oxide) δ 8.07 (s, 1H), 7.83 (s, 1H), 7.37 (dd, *J* = 20.0, 17.8 Hz, 1H), 6.93 (dd, *J* = 8.2 Hz, 1H), 6.75 (dd, *J* = 17.8, 14.8 Hz, 1H), 6.59 (d, *J* = 8.2 Hz, 1H), 6.55 (d, *J* = 8.0 Hz, 1H). **^13^C NMR** (101 MHz, deuterium oxide) δ 157.28, 156.29, 147.62, 145.55, 141.44, 137.79 (d, *J* = 165.0 Hz), 137.15, 130.47 (d, *J* = 5.0 Hz), 129.77, 126.13 (d, *J* = 19.3 Hz), 122.81, 120.04, 112.13, 103.44. **^19^F NMR** (377 MHz, deuterium oxide) δ −155.40 (d, *J* = 21.0 Hz), −160.34 (t, *J* = 22.2 Hz), −162.44––162.72 (m). **^31^P NMR** (162 MHz, deuterium oxide) δ 12.29. **HRMS** (ESI-FTMS) *m*/*z* for the acid: [M – H]^−^ calcd for C_20_H_10_O_5_N_3_F_5_PS, 530.0004; found, 530.0000.

##### Sodium (*E*)-7-((3-(2,3,4,5,6-Pentafluorophenyl)methoxy-2-(2-(phosphonato)vinyl)phenyl)thio)-3,5-dihydro-4*H*-pyrrolo[3,2-*d*]pyrimidin-4-one (**45o**)

The general procedure with compound **43l** (174 mg) afforded 125 mg (59%) of the coupling intermediate and 54 mg (60%) of the title compound as a white solid. **^1^H NMR** (500 MHz, deuterium oxide) δ 8.45 (s, 1H), 8.19 (s, 1H), 7.90 (dd, *J* = 22.4, 17.8 Hz, 1H), 7.48 (dd, *J* = 8.1 Hz, 1H), 7.34 (d, *J* = 8.1 Hz, 1H), 7.03 (dd, *J* = 17.8 Hz, 1H), 6.99 (d, *J* = 7.9 Hz, 2H), 5.69 (s, 2H). **^13^C NMR** (126 MHz, deuterium oxide) δ 157.65, 156.44, 148.38–147.69 (m), 147.53, 146.48–145.66 (m), 144.48, 141.04, 140.16–139.67 (m), 136.74, 135.20 (d, *J* = 6.2 Hz), 131.58 (d, *J* = 172.3 Hz), 130.14, 126.41 (d, *J* = 23.9 Hz), 122.24, 120.00, 112.92, 111.83–111.25 (m), 105.02, 60.24. **^19^F NMR** (377 MHz, deuterium oxide) δ −138.45––145.19 (m), −152.86 (t, *J* = 20.9 Hz), −161.75––162.05 (m). **^31^P NMR** (162 MHz, deuterium oxide) δ 14.84. **HRMS** (ESI-FTMS) *m*/*z* for the acid: [M – H]^−^ calcd for C_21_H_12_O_5_N_3_F_5_PS, 544.0161; found, 544.0155.

##### Sodium (*E*)-7-((5-Bromo-2-(2-(phosphonato)vinyl)phenyl)thio)-3,5-dihydro-4*H*-pyrrolo[3,2-*d*]pyrimidin-4-one (**45p**)

The general procedure starting with 300 mg of compound **9** and compound **31** (414 mg) afforded 184 mg (34%) of the coupling product and 60 mg (49%) of the title compound as a white solid. **^1^H NMR** (401 MHz, deuterium oxide) δ 8.05 (s, 1H), 7.70 (s, 1H), 7.54 (d, *J* = 8.1 Hz, 1H), 7.50 (dd, *J* = 18.0 Hz, 1H), 7.33 (dd, *J* = 8.5, 2.0 Hz, 1H), 6.89 (d, *J* = 2.0 Hz, 1H), 6.55 (dd, *J* = 17.2, 13.7 Hz, 1H). **^13^C NMR** (101 MHz, deuterium oxide) δ 165.78, 152.87, 149.40, 142.40, 139.37, 135.28 (d, *J* = 20.1 Hz), 133.99 (d, *J* = 6.1 Hz), 132.38 (d, *J* = 167.3 Hz), 129.48, 129.16, 124.57, 123.18, 97.51. **^31^P NMR** (162 MHz, deuterium oxide) δ 12.59. **HRMS** (ESI-FTMS) *m*/*z* for the acid: [M – H]^−^ calcd for C_14_H_10_O_4_N_3_BrPS, 425.9319; found, 425.9316.

##### Sodium (*E*)-7-((4-Bromo-2-(2-(phosphonato)vinyl)phenyl)thio)-3,5-dihydro-4*H*-pyrrolo[3,2-*d*]pyrimidin-4-one (**45q**)

The general procedure starting with 300 mg of compound **9** and compound **27** (414 mg) afforded 216 mg (40%) of the coupling product and 71 mg (49%) of the title compound as a white solid. NMR spectra were collected as a diastereomeric mixture of *cis*- and *trans*-isomers because of light-induced isomerization in solution in an NMR-tube. **^1^H NMR** (401 MHz, deuterium oxide) δ 8.14 (d, *J* = 2.2 Hz, 1H), 8.03 (d, *J* = 1.5 Hz, 1H), 7.81 (d, *J* = 2.2 Hz, 1H), 7.67 (d, *J* = 13.6 Hz, 1H), 7.51 (dd, *J* = 18.5, 17.2 Hz, 1H), 7.23 (ddd, *J* = 12.3, 8.5, 2.2 Hz, 1H), 7.10 (dd, *J* = 39.2, 14.2 Hz, 1H), 6.70 (dd, *J* = 31.4, 8.5 Hz, 1H), 6.55 (dd, *J* = 17.2, 13.4 Hz, 1H), 6.21 (dd, *J* = 14.2, 10.8 Hz, 1H). **^13^C NMR** (101 MHz, deuterium oxide) δ 165.68, 152.68, 149.36 (d, *J* = 6.1 Hz), 139.42, 139.36, 139.17, 138.82, 138.26 (d, *J* = 19.6 Hz), 137.96 (d, *J* = 7.7 Hz), 132.11, 131.69, 129.34, 127.85, 124.47, 124.40, 119.70, 118.64, 98.08, 97.84. **^31^P NMR** (162 MHz, deuterium oxide) δ 12.23, 9.47. **HRMS** (ESI-FTMS) *m*/*z* for the acid: [M – H]^−^ calcd for C_14_H_10_O_4_N_3_BrPS, 425.9319; found, 425.9315.

##### Diisopropyl ((4-Fluoro-2-iodophenoxy)methyl)phosphonate (**47a**)

4-Fluoro-2-iodophenol (1.00 g, 4.20 mmol, 1 equiv) was dissolved in dry tetrahydrofuran (20 mL) under an argon atmosphere. The solution was cooled to 0 °C; sodium hydride (210 mg of 60% oil dispersion, 5.25 mmol, 1.25 equiv) was added, and the mixture was stirred at 0 °C for 15 min. Then, TfOCH_2_P(O)(O*i*-Pr)_2_ (1.72 g, 5.25 mmol, 1.25 equiv) was added, and the mixture was stirred at 0 °C for additional 15 min. The reaction was quenched with a half-saturated aqueous solution of NH_4_Cl, extracted with ethyl acetate (3×), washed with brine (1×), dried with MgSO_4_, filtered, and evaporated. The solid was adsorbed onto silica gel in a mixture of cyclohexane/acetone and purified by flash chromatography on silica gel (cyclohexane to 20% of ethyl acetate modified with 10% of methanol (v/v_i_)), yielding 1.39 g (79%) of the title compound as a clear oil. **^1^H NMR** (401 MHz, chloroform-*d*) δ 7.49 (dd, *J* = 7.5, 3.0 Hz, 1H), 7.02 (ddd, *J* = 9.0, 7.7, 3.0 Hz, 1H), 6.86 (dd, *J* = 9.1, 4.5 Hz, 1H), 4.87 (dhept, *J* = 7.5, 6.2 Hz, 2H), 4.24 (d, *J* = 9.9 Hz, 2H), 1.42–1.31 (m, 12H). **^13^C NMR** (101 MHz, chloroform-*d*) δ 157.48 (d, *J* = 245.0 Hz), 154.31 (dd, *J* = 13.2, 2.3 Hz), 126.47 (d, *J* = 25.0 Hz), 115.87 (d, *J* = 22.9 Hz), 112.95 (d, *J* = 8.2 Hz), 85.73 (d, *J* = 8.6 Hz), 72.26 (d, *J* = 6.6 Hz), 64.58 (d, *J* = 170.1 Hz), 26.53–22.24 (m). **^19^F NMR** (377 MHz, chloroform-*d*) δ −116.31––124.37 (m). **^31^P NMR** (162 MHz, chloroform-*d*) δ 18.12. **MS** (ESI-QMS) *m*/*z*: [M + H]^+^ calcd for C_13_H_20_FIO_4_P, 417.0; found, 417.0.

##### Diisopropyl ((2-Iodo-6-methoxyphenoxy)methyl)phosphonate (**47b**)

Following the previous procedure with 2-iodo-6-methoxyphenol (1 g, 4.0 mmol, 1 equiv), sodium hydride (200 mg of 60% oil dispersion, 5.0 mmol, 1.25 equiv), and TfOCH_2_P(O)(O*i*-Pr)_2_ (1.64 g, 5.0 mmol, 1.25 equiv) afforded 1.70 g (99%) of the title compound as a clear oil. **^1^H NMR** (401 MHz, chloroform-*d*) δ 7.33 (dd, *J* = 7.9, 1.5 Hz, 1H), 6.87 (dd, *J* = 8.2, 1.6 Hz, 1H), 6.80 (dd, *J* = 8.0 Hz, 1H), 4.88 (dhept, *J* = 7.3, 6.2 Hz, 2H), 4.31 (d, *J* = 9.7 Hz, 2H), 3.83 (s, 3H), 1.41 (d, *J* = 6.2 Hz, 12H). **^13^C NMR** (101 MHz, chloroform-*d*) δ 152.56, 147.76 (d, *J* = 14.8 Hz), 130.87, 126.59, 112.90, 91.80, 72.17 (d, *J* = 6.6 Hz), 66.10 (d, *J* = 166.6 Hz), 55.97, 26.07–22.47 (m). **^31^P NMR** (162 MHz, chloroform-*d*) δ 19.60. **MS** (ESI-QMS) *m*/*z*: [M + H]^+^ calcd for C_14_H_23_IO_5_P, 429.0; found, 429.2.

##### Sodium 7-((5-Fluoro-2-((phosphonato)methoxy)phenyl)thio)-3,5-dihydro-4*H*-pyrrolo[3,2-*d*]pyrimidin-4-one (**49a**)

The general procedure with compound **47a** (129 mg) afforded 105 mg (63%) of the coupling intermediate and 21 mg (32%) of the title compound as a white solid. **^1^H NMR** (401 MHz, deuterium oxide) δ 8.07 (s, 1H), 7.86 (s, 1H), 7.10 (dd, *J* = 9.1, 4.5 Hz, 1H), 6.89 (ddd, *J* = 8.7, 3.0 Hz, 1H), 6.31 (dd, *J* = 9.1, 3.0 Hz, 1H), 4.16 (d, *J* = 9.6 Hz, 2H). **^13^C NMR** (101 MHz, deuterium oxide) δ 158.59 (d, *J* = 239.1 Hz), 156.30, 152.89 (d, *J* = 13.0 Hz), 147.44, 144.63, 137.23, 130.18 (d, *J* = 7.8 Hz), 119.96, 114.57 (d, *J* = 8.5 Hz), 113.87 (d, *J* = 26.4 Hz), 113.62 (d, *J* = 23.3 Hz), 102.18, 67.67 (d, *J* = 154.7 Hz). **^19^F NMR** (377 MHz, deuterium oxide) δ −122.28 (d, *J* = 5.4 Hz). **^31^P NMR** (162 MHz, deuterium oxide) δ 15.10. **HRMS** (ESI-FTMS) *m*/*z* for the acid: [M – H]^−^ calcd for C_13_H_10_O_5_N_3_FPS, 370.0068; found, 370.0064.

##### Sodium 7-((3-Methoxy-2-((phosphonato)methoxy)phenyl)thio)-3,5-dihydro-4*H*-pyrrolo[3,2-*d*]pyrimidin-4-one (**49b**)

The general procedure with compound **47b** (133 mg) afforded 114 mg (64%) of the coupling intermediate and 32 mg (46%) of the title compound as a white solid. **^1^H NMR** (401 MHz, deuterium oxide) δ 7.99 (s, 1H), 7.63 (s, 1H), 6.92 (dd, *J* = 8.0 Hz, 1H), 6.87 (dd, *J* = 8.3, 1.7 Hz, 1H), 6.25 (dd, *J* = 7.8, 1.7 Hz, 1H), 4.11 (d, *J* = 9.5 Hz, 2H), 3.92 (s, 3H). **^13^C NMR** (101 MHz, deuterium oxide) δ 166.33, 153.30, 152.04, 150.15, 145.13 (d, *J* = 13.6 Hz), 141.85, 136.73, 126.36, 126.14, 119.40, 110.71, 95.72, 72.23 (d, *J* = 145.9 Hz), 57.38. **^31^P NMR** (162 MHz, deuterium oxide) δ 14.84. **HRMS** (ESI-FTMS) *m*/*z* for the acid: [M – H]^−^ calcd for C_14_H_13_O_6_N_3_PS, 382.0268; found, 382.0265.

##### 7-((2-(((((*S*)-1-(Isopropoxycarbonyl)ethyl)amino)(phenoxy)phosphoryl)methoxy)phenyl)thio)-3,5-dihydro-4*H*-pyrrolo[3,2-*d*]pyrimidin-4-one (**50a**)

**Method 1:** a flask was charged with the starting compound **17c** (300 mg, 0.6858 mmol, 1 equiv). It was sealed with a septum, and flushed with argon, and then dry pyridine (10 mL/1 mmol of the starting compound) was added followed by trimethylsilyl bromide (1 mL/1 mmol of the starting compound). The mixture was stirred at room temperature overnight and then evaporated to dryness under an argon atmosphere. l-Alanine isopropyl ester hydrochloride (230 mg, 1.37 mmol, 2 equiv) was added, the flask was sealed with a septum and flushed with argon, and then dry pyridine (10 mL/1 mmol of the starting compound) and triethylamine (572 μL, 4.11 mmol, 6 equiv) were added. The mixture was stirred for 15 min at 70 °C, and a solution of triphenylphosphine (1.08 g, 4.11 mmol, 6 equiv) and Aldrithiol-2 (905 mg, 4.11 mmol, 6 equiv) in dry pyridine (10 mL/1 mmol of the starting compound) was added. The mixture was stirred overnight at 70 °C. The reaction was quenched by evaporation of solvents, and the residue was co-evaporated with toluene. The solid residue was adsorbed onto silica gel in a mixture of cyclohexane/acetone and purified by flash chromatography on silica gel (chloroform to 20% of methanol), followed by purification on HPLC (C18, gradient H_2_O/MeOH as an eluent). The purified product was lyophilized from dioxane, yielding 26 mg (7%) of the title compound as a white solid. **Method 2:** a flask charged with compound **52** (5.00 g, 8.40 mmol, 1 equiv), l-alanine isopropyl ester hydrochloride (2.50 g, 14.9 mmol, 1.77 equiv), and phenol (3.49 g, 37.1 mmol, 4.42 equiv) was sealed with a septum and flushed with argon. Then, dry pyridine (40 mL/1 mmol of the starting compound) and triethylamine (12.4 mL, 89.0 mmol, 10.6 equiv) were added. The mixture was stirred for 15 min at 60 °C. A solution of triphenylphosphine (11.5 g, 44.0 mmol, 5.24 equiv) and Aldrithiol-2 (13.6 g, 61.7 mmol, 7.34 equiv) in dry pyridine (20 mL/1 mmol of the starting compound) was added. The mixture was stirred overnight at 60 °C. The reaction was quenched by evaporation of solvents and the residue was co-evaporated with toluene (2×). The solid residue was adsorbed on silica gel in a mixture of cyclohexane/acetone and purified by flash chromatography on silica gel (chloroform to 10% methanol). This purification was repeated once more, yielding 3.52 g (77%) of the title compound as a white solid. **^1^H NMR** (401 MHz, DMSO-*d*_6_) δ 12.66 (s, 1H), 12.09 (s, 1H), 7.83 (d, *J* = 1.6 Hz, 1H), 7.69 (d, *J* = 8.0 Hz, 1H), 7.44–7.34 (m, 2H), 7.34–7.22 (m, 2H), 7.22–7.13 (m, 1H), 7.13–7.07 (m, 1H), 7.08–7.00 (m, 1H), 6.82–6.71 (m, 1H), 6.57–6.47 (m, 1H), 5.97–5.76 (m, 1H), 4.93–4.72 (m, 1H), 4.59–4.37 (m, 2H), 4.21–4.04 (m, 1H), 1.37–1.19 (m, 3H), 1.16–1.04 (m, 6H). **^13^C NMR** (101 MHz, DMSO-*d*_6_) δ 173.13–172.79 (m), 154.10–153.85 (m), 153.67, 150.29–149.99 (m), 146.00, 142.74, 134.08–133.74 (m), 129.68, 128.16–127.88 (m), 125.75–125.58 (m), 125.47, 124.76–124.53 (m), 122.02–121.78 (m), 120.95–120.73 (m), 119.35, 112.13–111.70 (m), 101.06–100.79 (m), 68.01, 65.72–63.04 (m), 49.93–48.85 (m), 21.73–21.04 (m), 20.56–19.98 (m). **^31^P NMR** (162 MHz, DMSO-*d*_6_) δ 23.09, 22.75. **HRMS** (ESI-FTMS) *m*/*z*: [M + H]^+^ calcd for C_25_H_28_O_6_N_4_PS, 543.1462; found, 543.1462.

##### 7-((2-(((((*S*)-1-(Isopropoxycarbonyl)-2-(phenyl)ethyl)amino)(phenoxy)phosphoryl)methoxy)phenyl)thio)-3,5-dihydro-4*H*-pyrrolo[3,2-*d*]pyrimidin-4-one (**50b**)

Following the previous procedure with l-phenylalanine isopropyl ester hydrochloride (335 mg, 1.37 mmol, 2 equiv) as the amino acid afforded 130 mg (31%) of the title compound as a white solid. **^1^H NMR** (401 MHz, DMSO-*d*_6_) δ 12.71–12.62 (m, 1H), 12.14–12.03 (m, 1H), 7.85–7.78 (m, 1H), 7.71–7.61 (m, 1H), 7.37–7.11 (m, 10H), 7.09–6.99 (m, 1H), 6.97–6.84 (m, 1H), 6.82–6.73 (m, 1H), 6.53–6.46 (m, 1H), 4.87–4.65 (m, 1H), 4.28–4.23 (m, 1H), 4.36–4.12 (m, 1H), 3.04–2.80 (m, 2H), 1.12–0.94 (m, 6H). **^13^C NMR** (101 MHz, DMSO-*d*_6_) δ 171.91, 153.86 (d, *J* = 13.0 Hz), 153.68–153.59 (m), 150.24–149.91 (m), 146.16–145.82 (m), 142.71, 137.24–136.61 (m), 133.82, 129.82–129.21 (m), 128.38–128.04 (m), 128.04–127.70 (m), 126.76–126.38 (m), 125.71–125.49 (m), 125.45–125.32 (m), 124.55, 121.85, 120.92–120.45 (m), 119.43–119.15 (m), 111.75, 100.94, 68.29–67.83 (m), 64.03 (d, *J* = 158.1 Hz), 55.50–55.02 (m), 39.52, 21.60–21.01 (m). **^31^P NMR** (162 MHz, DMSO-*d*_6_) δ 22.97, 22.91. **HRMS** (ESI-FTMS) *m*/*z*: [M + H]^+^ calcd for C_31_H_32_O_6_N_4_PS, 619.1775; found, 619.1773. LC/MS purity 93% (at 254 nm).

##### 7-((2-((Bis(((*S*)-1-(isopropoxycarbonyl)ethyl)amino)phosphoryl)methoxy)phenyl)thio)-3,5-dihydro-4*H*-pyrrolo[3,2-*d*]pyrimidin-4-one (**51a**)

The title compound was recovered as a byproduct of the synthesis of compound **50a** with l-alanine isopropyl ester hydrochloride (230 mg, 1.37 mmol, 2 equiv) as an amino acid. The purified product was lyophilized from dioxane, yielding 262 mg (66%) of the title compound as a white solid. **^1^H NMR** (401 MHz, DMSO-*d*_6_) δ 12.67 (s, 1H), 12.09 (s, 1H), 7.82 (d, *J* = 2.8 Hz, 1H), 7.66 (d, *J* = 2.2 Hz, 1H), 7.11–6.98 (m, 2H), 6.75 (ddd, *J* = 7.8, 5.7, 2.8 Hz, 1H), 6.55–6.44 (m, 1H), 4.93–4.83 (m, 2H), 4.81–4.72 (m, 1H), 4.71–4.62 (m, 1H), 4.30–4.16 (m, 2H), 4.11–3.99 (m, 1H), 3.99–3.86 (m, 1H), 1.37–1.28 (m, 6H), 1.21–1.14 (m, 12H). **^13^C NMR** (101 MHz, DMSO-*d*_6_) δ 173.47 (d, *J* = 5.1 Hz), 154.36 (d, *J* = 12.4 Hz), 153.65, 145.97, 142.67, 133.77, 127.99, 125.66, 125.46, 121.59, 119.29, 111.86, 101.18, 67.85, 65.71 (d, *J* = 135.0 Hz), 48.40 (d, *J* = 10.3 Hz), 21.43, 20.88 (dd, *J* = 13.6, 4.9 Hz). **^31^P NMR** (162 MHz, DMSO-d_6_) δ 21.41. **HRMS** (ESI-FTMS) *m*/*z*: [M + H]^+^ calcd for C_25_H_35_O_7_N_5_PS, 580.1989; found, 580.1985.

##### 7-((2-((Bis(((*S*)-1-(isopropoxycarbonyl)-2-(phenyl)ethyl)amino)phosphoryl)methoxy)phenyl)thio)-3,5-dihydro-4*H*-pyrrolo[3,2-*d*]pyrimidin-4-one (**51b**)

The title compound was recovered as a byproduct of the synthesis of compound **50b**. The purified product was lyophilized from dioxane, yielding 224 mg (45%) of the title compound as a white solid. **^1^H NMR** (401 MHz, DMSO-*d*_6_) δ 7.82 (s, 1H), 7.64 (s, 1H), 7.30–7.10 (m, 10H), 7.04 (ddd, *J* = 8.3, 7.4, 1.6 Hz, 1H), 6.80 (dd, *J* = 8.3, 1.2 Hz, 1H), 6.75 (ddd, *J* = 7.6, 1.1 Hz, 1H), 6.51 (dd, *J* = 7.8, 1.6 Hz, 1H), 4.88–4.73 (m, 2H), 4.71–4.59 (m, 1H), 4.48–4.34 (m, 1H), 4.22–4.10 (m, 1H), 4.10–3.99 (m, 1H), 3.82 (d, *J* = 8.9 Hz, 2H), 3.04–2.81 (m, 4H), 1.19–0.98 (m, 12H). **^13^C NMR** (101 MHz, DMSO-*d*_6_) δ 172.10, 154.18 (d, *J* = 12.7 Hz), 153.65, 146.01, 142.68, 137.00 (d, *J* = 6.7 Hz), 133.73, 129.58, 128.12, 127.96, 126.47 (d, *J* = 4.8 Hz), 125.63, 125.37, 121.58, 119.30, 111.70, 101.17, 68.02, 65.52 (d, *J* = 169.5 Hz), 54.09 (d, *J* = 14.7 Hz), 21.59–21.11 (m). **^31^P NMR** (162 MHz, DMSO-*d*_6_) δ 21.33. **HRMS** (ESI-FTMS) *m*/*z*: [M + H]^+^ calcd for C_37_H_43_O_7_N_5_PS, 732.2615; found, 732.2613.

##### Mono(tetrabutylammonium) 7-((2-((Phosphonato)methoxy)phenyl)thio)-3,5-dihydro-4*H*-pyrrolo[3,2-*d*]pyrimidin-4-one (**52**)

A flask charged with compound **17c** (12.0 g, 27.4 mmol, 1 equiv) was sealed with a septum and flushed with argon, and then dry pyridine (10 mL/1 mmol of the starting compound) was added followed by trimethylsilyl bromide (1 mL/1 mmol of the starting compound). The mixture was stirred at room temperature overnight. The mixture was evaporated to dryness and co-evaporated with toluene (2×). The residue was dissolved in an aqueous solution of triethylammonium bicarbonate (2 M) followed by the addition of tetrabutylammonium hydroxide (2 mL of 1.5 M aq solution/1 mmol of the starting compound). The solution was filtered through P2 and P4 glass filters, and it was evaporated to dryness. The resulting oil was dissolved in methanol, adsorbed onto C18 silica gel, and purified by C18 reverse-phase flash chromatography (water to methanol, 0–50%), yielding 14.5 g (89%) of the title mono(tetrabutylammonium) salt as a white solid. **^1^H NMR** (401 MHz, DMSO-*d*_6_) δ 7.83 (s, 1H), 7.72 (s, 1H), 7.07 (dd, *J* = 8.3, 1.2 Hz, 1H), 6.94 (dd, *J* = 8.2, 7.8, 1.7 Hz, 1H), 6.61 (dd, *J* = 7.5, 1.2 Hz, 1H), 6.51 (dd, *J* = 7.7, 1.7 Hz, 1H), 3.87 (d, *J* = 9.2 Hz, 2H), 3.24–3.09 (m, 8H), 1.63–1.46 (m, 8H), 1.37–1.20 (m, 8H), 0.92 (t, *J* = 7.3 Hz, 12H). **^13^C NMR** (101 MHz, DMSO-*d*_6_) δ 155.84 (d, *J* = 9.4 Hz), 154.81, 146.22, 143.25, 134.38, 127.71, 125.51, 125.17, 120.05, 119.42, 111.78, 100.63, 67.56 (d, *J* = 151.6 Hz), 57.51, 23.08, 19.20, 13.50. **^31^P NMR** (162 MHz, DMSO-*d*_6_) δ 10.93. **MS** (ESI-QMS) *m*/*z* for the acid: [M + H]^+^ calcd for C_13_H_13_O_5_N_3_PS, 354.0; found, 354.0.

## Supporting Information Available

Copies of the NMR (^1^H, ^13^C{^1^H}, ^19^F{^1^H}, and ^31^P{^1^H}) and UPLC/MS spectra, crystallography data, and pharmacology data (PDF)
Molecular formula strings (Skacel et al.) (CSV)

## Abbreviations

ANP: acyclic nucleoside phosphonates
dCK/dGK: deoxycytidine/deoxyguanosine kinase
dGuo: 2′-deoxyguanosine
DMPK: drug metabolism and pharmacokinetics
HWE: Horner–Wadsworth–Emmons
ImmH: immucillin-H
MW: microwave irradiation
NMR: nuclear magnetic resonance
PBMC: peripheral blood mononuclear cells
PEE: phosphonoethoxyethyl
PFBn: pentafluorobenzyl
PFPh: pentafluorophenyl
PK: pharmacokinetic
PME: phosphonomethoxyethyl
PMEG: 9-((2-phosphonylmethoxy)ethyl)guanine
PNP: purine nucleoside phosphorylase
PNPI: purine nucleoside phosphorylase inhibitor
rt: room temperature
SEM: 2-(trimethylsilyl)ethoxymethyl
T-ALL: T-cell lymphoblastic leukemia
TCEP: tris(2-carboxyethyl)phosphine
TLC: thin-layer chromatography
TLR: toll-like receptors
TMS: trimethylsilyl.
