# Supplementary material for: Design, Synthesis, Biological Evaluation, and Crystallographic Study of Novel Purine Nucleoside Phosphorylase Inhibitors
Source: J Med Chem. 2023 May 3;66(10):6652–81. doi: 10.1021/acs.jmedchem.2c02097 (PMC10226123; doi:10.1021/acs.jmedchem.2c02097)
Supplement: Supplementary file 1 — jm2c02097_si_001.pdf [file jm2c02097_si_001.pdf]

Supporting Information

Design, Synthesis, Biological Evaluation, and Crystallographic Study of Novel Purine Nucleoside Phosphorylase Inhibitors

Jan Skácel,[1‡] Stefan Djukic,[1‡] Ondřej Baszczyňski,[1,4] Filip Kalčic,[1] Tadeáš Bílek,[1] Karel Chalupský,[1] Jaroslav Kozák,[1] Alexandra Dvořáková,[1] Eva Tloušťová,[1] Zuzana Kráľová,[1,4] Markéta Šmídková,[1] Jan Voldřich,[1,3] Michaela Rumlová,[3] Petr Pachl,[1] Jiří Brynda,[1] Tereza Vučková,[1] Milan Fabry,[1,2] Jan Snášel,[1] Iva Pichová,[1] Pavlína Řezáčová,[1*] Helena Mertlíková-Kaiserová,[1*] and Zlatko Janeba,[1*]

*pavlina.rezacova@uochb.cas.cz

*helena.kaiserova@uochb.cas.cz

*zlatko.janeba@uochb.cas.cz

[1] Institute of Organic Chemistry and Biochemistry, The Czech Academy of Sciences, Flemingovo nám. 2, 16610 Prague, Czech Republic.

[2] Institute of Molecular Genetics, The Czech Academy of Sciences, Vídeňská 1083, 14220 Prague, Czech Republic.

[3] University of Chemistry and Technology, Technická 5, 166 28, Prague, Czech Republic.

[4] Faculty of Science, Charles University in Prague, Hlavova 2030/8, 128 43 Prague 2, Czech Republic.

Table of Contents

1 Crystallography S2
1.1 Methods S2
1.2 Description of crystal structures S2
1.3 Supplementary tables S4
1.4 Supplementary figures S9
1.5 Additional comparisons S10
2 Pharmacology S14
3 Chemistry S15
3.1 List of inhibitors S15
3.2 Copies of the NMR and UPLC/MS Spectra S15
4 References S173

1 Crystallography

1.1 Methods

1.1.1 Protein purification for crystallography

For crystallization purposes, hPNP gene was cloned into T7-driven pET-like expression plasmid. Protein was equipped with N-terminal His6 tag followed by a recognition site for TEV protease (TEV PR). A five-residues long cloning artefact (SNAAS) remains on recombinant protein N-terminus product upon cleavage by TEV PR. The protein was overexpressed in E. coli BL21 (DE3) at 37 °C in LB medium supplemented by 0.5% glycerol and 100 µg/mL ampicillin. At OD595 nm value of 0.7, temperature was decreased to 18 °C and the protein expression was induced with an addition of ethyl-β-D-thiogalactopyranoside at a final concentration of 400 µM and cultivation proceeded for 20 h before cells were harvested by centrifugation. The bacterial cells were lysed by sonication at 4 °C in 10 volumes of lysis buffer (20 mM Potassium phosphate, 400 mM NaCl, 0.05% β-mercaptoethanol, pH 7.5) supplemented with protease inhibitors (ETDA-free cOmplete™, Sigma Aldrich). Clarified lysate was loaded on to 5 mL HisTrap Ni-NTA column (Sigma Aldrich) equilibrated in lysis buffer. To elute His6-tagged protein, subsequent elution by 5 mL of 50 mM, 250 mM, and 500 mM imidazole in lysis buffer was used. Three 5 mL fractions were collected for each elution, and these were pooled and dialyzed against buffer A for 8 hours at 4°C. The His6 tag was cleaved off by recombinant His6 tagged TEV PR during additional dialysis against lysis buffer for 24 h at 4 °C. The TEV PR and the cleaved His6 tag were removed using a second nickel-chelation chromatography step. Last purification step involved gel filtration on HiLoad 10/300 Superdex 200 pg column (Sigma Aldrich) using the FPLC system Äkta Basic (GE Healthcare, USA) and running buffer 20 mM potassium phosphate, 150 mM NaCl, 1 mM Tris (2-carboxyethyl)phosphine (TCEP), pH 7.4. Protein was concentrated using Vivaspin MWCO 10 kDa concentrators (Millipore, USA), aliquoted and stored at -80 °C. Purification yield was 10.3 mg of the protein from 1 L of bacterial culture. The protein purity was assessed by a silver-stained SDS-PAGE to be greater than 95%.

The coding sequence for *MtPNP* was inserted in the pET 15b (NovagenTM) vector containing N-terminal His6 tag followed by thrombin cleavage site. *MtPNP* was expressed in E. coli BL21 (DE3) Nutrient broth medium (HIMEDIA®) supplemented with ampicillin (50 µg/mL). The inoculum was grown at 37 °C overnight and large-scale culture media supplemented with ampicillin (50 µg/mL) were cultivated at 30 °C until the optical density OD600nm reached the value 0.5. Then IPTG was added to the final concentration 1 mM and cultivation of the cells continued for another 20 hours at 30 °C. The harvested cells were resuspended in a buffer (50 mM Tris.HCl, pH 8.0, 500 mM NaCl) containing Sigma cOmpleteTM protease inhibitor cocktail (EDTA-free). Lysozyme was added to the concentration 0.5 mg/mL and the mixture was incubated for 30 min at room temperature. The cells were sonicated in several short bursts and centrifuged at 20,000 G for 45 min (4 °C). The supernatant was loaded onto TalonTM metal affinity resin (TaKaRaTM). The column was washed with the buffer containing 20 mM imidazole and protein eluted from the resin with 500 mM imidazole in 50 mM Tris.HCl, pH 8.0. The collected fractions were dialyzed against the buffer 50 mM Tris, pH 8.0, 100 mM NaCl, 5 mM β-mercaptoethanol. Next, the protein was purified via Superdex 200 TM column in 40 mM HEPES, 100 mM NaCl, pH 7.5, 5 mM 2-mercaptoethanol. $CaCl_2$ was added to the final concentration 25 mM and the His6 tag was removed by adding biotinylated Thrombin (NovagenTM) to the final concentration (1 mg/mL) and incubation of 10 hours at room temperature. Biotinylated thrombin was removed by passing the reaction mixture through Strepavidin-Agarose beads (PierceTM) containing minicolumn. The sample was concentrated using Vivaspin MWCO 10 kDa concentrators (Millipore, USA), aliquoted, and stored at -80 °C. Protein purity was assessed by Comassie blue-stained SDS-PAGE to be greater than 95%. Yield was 20 mg/L of bacterial culture.

1.2 Description of crystal structures

1.2.1 Overview of hPNP structures

Structure of a hPNP/compound **18c** complex was solved to the resolution of 1.8 Å. The asymmetric unit contained six molecules which formed two biological trimers. Superposition of all three molecules showed

the average displacement of Cα of 0.125- 0.230 Å. The ligand was modeled into well-defined electron density map with full occupancy in all six molecules. Molecule A was used in structural analysis. Disordered residues and side chains that were not modeled into electron density map are listed in Table S3**.**

Structure of hPNP/**45b** complex was determined at the resolution 2.65 Å. The asymmetric unit contained one molecule. The inhibitor was modeled into electron density map with full occupancy. Disordered residues and side chains which were not modeled into electron density map are listed in Table S3.

Structure of hPNP/**45q** complex was determined at the resolution 2.36 Å. The asymmetric unit contained one molecule. The inhibitor was modeled into well-defined electron density map with high occupancy (0.95). Disordered residues and side chains which were not modeled into electron density map are listed in Table S3.

Structure of hPNP/**45i** complex was determined at the resolution 1.95 Å. The asymmetric unit contained three molecules which formed a biological trimer. The inhibitor was modeled with full occupancy in molecule A, while the position of fluorinated phenyl ring was unclear in molecules B and C. Molecule A was used for structural analysis. Superposition of all three molecules showed the average displacement of Cα of 0.617 Å Disordered residues and side chains which were not modeled into electron density map are listed in Table S3.

Structure of hPNP/**45n** complex was determined at the resolution 2.3 Å. The asymmetric unit contained one molecule. The inhibitor was modeled with full occupancy in the molecule. Disordered residues and side chains which were not modeled into electron density map are listed in Table S3.

1.2.2 Overview of *Mt*PNP structures

Structure of *Mt*PNP in complex with compound **18c** was determined at a resolution of 1.77 Å. The asymmetric unit contained three molecules which formed a biological trimer. Superposition of all three molecules showed the average displacement of Cα of 1.176 Å. All the residues were modeled into electron density map except disordered residues N-terminal and C-terminal regions as well as disorder residues 63-68 of chain C (Table S4). Compound **18c** was modelled into well-defined electron density map into the active sites of each molecule with full occupancy (Figure S2A).

Structure of *Mt*PNP in complex with compound **45b** was determined at resolution of 1.58 Å. The asymmetric unit contained two molecules with each belonging to separate biological trimer. Average displacement of all the Cα atoms between two molecules is 0.392. All the residues were modeled into electron density map except for disordered N-terminal as well as region 62-69 (Table S4). Inhibitor was modeled into well-defined electron density map with full occupancy (Figure S2B).

Structure of *Mt*PNP in complex with compound **45q** was solved at resolution of 1.9 Å. The asymmetric unit contained three molecules which formed a biological trimer. Average displacement of all the Cα atoms between three molecules is 0.685 Å. The inhibitor was modelled into well-defined electron density map with full occupancy (Figure S2C). All the residues were modeled into electron density map except for disordered N-terminal residues (Table S4).

For structural analyses and comparisons, chain A of all crystal structures was used.

1.3 Supplementary tables

Table S1: hPNP structures statistics.

**Ligand**	**18c**	**45b**	**45i**	**45q**	**45n**
**Data collection statistics**					
**Space group**	**$P2_1$**	**R32**	**$P2_1$**	**R32**	**R32**
**Cell parameters (Å; °)**	79.12<br>185.10<br>79.18<br>90.00<br>119.94<br>90.00	141.85<br>141.85<br>164.34<br>90.00<br>90.00<br>120.00	58.48<br>119.33<br>130.18<br>90.00<br>90.00<br>90.00	97.80<br>97.83<br>97.84<br>92.44<br>92.52<br>92.49	98.13<br>98.20 98.16<br>92.33<br>92.38<br>92.40
**Wavelength (Å)**	0.918	0.918	1.5	0.918	0.918
**Resolution (Å)**	50-1.8<br>(1.85-1.8)	50-2.65<br>(2.81-2.65)	50-1.95<br>(2-1.95)	50-2.35<br>(2.50-2.35)	50-2.29<br>(2.43-2.29)
**Number of unique reflections**	156,419<br>(6,131)	18,872<br>(2,997)	65,544<br>(4,612)	50135<br>(8049)	55748<br>(8856)
**Multiplicity**	3.55 (2.01)	19 (19.9)	6.64 (4.57)	10.18 (10.25)	10.1 (10)
**Completeness (%)**	85.8<br>(45.5)	99.9 (99.7)	97.5<br>(94.3)	99.9<br>(99.3)	99.7<br>(98.2)
**R_{merge}[a]**	7.7<br>(88.9)	11.4<br>(360.8)	15.0<br>(236.3)	13.4<br>(287.2)	11.6<br>(282.8)
**$CC_{(1/2)}$ (%)[b]**	99.7 (44.2)	100 (41.7)	99.6 (29.0)	99.9 (35.1)	99.9 (30.7)
**Average I/s(I)**	13.26 (1.25)	22.38(0.87)	9.29 (0.94)	7.89 (0.26)	13.87 (0.70)
**Wilson B ($Å^2$)**	29.98	88.307	33.22	67.16	65.59
**Refinement statistics**					
**Resolution range (Å)**	38.86-1.8<br>(1.846-1.8)	49.3-2.65<br>(2.716-2.65)	40 -1.95<br>(2.04-1.95)	49.01-2.36<br>(2.42-2.36)	49.28-2.29<br>(2.35-2.29)
**No. of reflections in working set**	154,865<br>(6,071)	17,927<br>(1287)	63,905<br>(4,496)	24,922<br>(1,824)	27795<br>(1956)
**No. of reflections in test set**	1565 (62)	944 (68)	1639 (115)	1090 (80)	1088 (76)
**R value (%)[c]**	22.5 (34.8	22.1 (68.9)	22.0 (33.0)	22.0 (61.7)	21.35 (60.2)
**R_{free} value (%)[d]**	26.7 (33.1)	24.4 (78.5)	27.0 (29.7)	25.7 (66.0)	25.58(57.3)
**RMSD bond length (Å)**	0.01	0.005	0.008	0.006	0.007
**RMSD angle (°)**	1.682	1.350	1.480	1.598	1.634
**Number of atoms in AU (protein/ligand/water molecules)**	13921<br>12718/161/955	2,205<br>2125/25/10	6,880<br>6388/105/394	2,299<br>2138/24/40	2360<br>2183/35/49
**Mean B value ($Å^2$)**	27.50	84.84	30.61	66.379	66.95
**Residues in favored regions (%)**	98.64	96.36	94.88	95.71	94.62
**Residues in allowed regions (%)**	100	98.18	98.8	96.42	95.32
**PDB code**	7ZSL	7ZSM	7ZSN	7ZSO	7ZSP

The data in parentheses refer to the highest-resolution shell.

[a] $R_{merge} = (|I_{hkl} - \langle I \rangle)/I_{hkl}$, where the average intensity $\langle I \rangle$ is taken over all symmetry equivalent measurements and I_{hkl} is the measured intensity for any given reflection.[1]

[b] $CC_{(1/2)}$ is the correlation coefficient between random half data sets and from its value the Pearson correlation coefficient of the true level of signal can be calculated: $CC^* = \sqrt{2CC_{1/2}/1 + CC_{1/2}}$.[2]

[c] R-value = $||F_o| - |F_c||/|F_o|$, where F_o and F_c are the observed and calculated structure factors, respectively

[d] R_{free} is equivalent to R-value but is calculated for 5% of the reflections chosen at random and omitted from the refinement process.[3]

[e] As determined by Molprobity.[4]

Table S2: *Mt*PNP structures statistics.

**Ligand**	**18c**	**45b**	**45q**
**Data collection statistics**			
**Space group**	**P222**	**$P2_13$**	**$P2_1$**
**Cell parameters (Å; °)**	57.05<br>90.91<br>143.03<br>90.00<br>90.00<br>90.00	125.13<br>125.13<br>125.13<br>90.00<br>90.00<br>90.00	50.57<br>84.58<br>93.97<br>90.00<br>94.44<br>90.00
**Wavelength (Å)**	0.918	0.918	0.918
**Resolution (Å)**	50.00-1.77<br>(1.88-1.77)	50-1.56<br>(1.56-1.65)	50-1.90<br>(1.95-1.9)
**Number of unique reflections**	140,249<br>(22,599)	180,675<br>(29,016)	58,542<br>(3,729)
**Multiplicity**	6.96 (7.19)	19.2 (19.3)	3.8 (3.71)
**Completeness (%)**	99.9 (99.6)	99.8 (98.8)	81.5 (81.5)
**R_{merge}[a]**	17.4 (271.0)	27.1 (284.2)	16.5 (247.0)
**$CC_{(1/2)}$ (%)[b]**	99.7 (25.3)	99.8 (39.7)	99.6 (44.0)
**Average I/s(I)**	8.39 (0.62)	12.83 (0.88)	7.15 (0.65)
**Wilson B (Å^2)**	34.63	24.21	36.672
**Refinement statistics**			
**Resolution range (Å)**	48.38-1.77<br>(1.82-1.77)	44.24-1.56<br>(1.6-1.56)	45.0-1.97<br>(2.03-1.97)
**No. of reflections in working set**	72,306 (5239)	91,714 (6674)	40,903 (3540)
**No. of reflections in test set**	1,099 (79)	1,079 (79)	2,148 (186)
**R value (%)[c]**	17.9 (36.4)	15.6 (36.7)	18.9 (34.9)
**R_{free} value (%)[d]**	21.7 (35.7)	18.7 (32.7)	25.26 (36.2)
**RMSD bond length (Å)**	0.011	0.013	0.007
**RMSD angle (°)**	1.894	1.846	1.542
**Number of atoms in AU (protein/ligand/water molecules)**	6,296<br>5653/81/426	4,529<br>3946/50/626	6,019<br>5667/72/257
**Mean B value (Å^2)**	30.07	19.0	36.707

**Residues in favored regions (%)**	98.22	98.21	96.03
**Residues in allowed regions (%)**	1.65	1.79	3.71
**PDB code**	7ZSQ	8C25	7ZSR

The data in parentheses refer to the highest-resolution shell.

[a] R_{merge} = (|I_{hkl} - ⟨I⟩)/I_{hkl}, where the average intensity ⟨I⟩ is taken over all symmetry equivalent measurements and I_{hkl} is the measured intensity for any given reflection.[1]

[b] $CC_{(1/2)}$ is the correlation coefficient between random half data sets and from its value the Pearson correlation coefficient of the true level of signal can be calculated: $CC^{*} = \sqrt{2CC_{1/2}/1 + CC_{1/2}}$.[2]

[c] R-value = ||F_o| - |F_c||/|F_o|, where F_o and F_c are the observed and calculated structure factors, respectively

[d] R_{free} is equivalent to R-value but is calculated for 5% of the reflections chosen at random and omitted from the refinement process.[3]

[e] As determined by Molprobity.[4]

Table S3: Residues missing in crystallographic model of hPNP.

**Structure**	**Chain**	**N-terminus**	**other**	**C-terminus**	**missing side-chains**
hPNP/**18c**	A	1-2	59-65	286-300	11, 179, 184, 265
	B	1-2	58-65	285-300	11, 168, 179, 211, 250, 258, 269
	C	1-3	59-65;182-183	286-300	179-181, 211, 252, 269, 278, 284
	D	1-3	59-65	285-300	11, 168, 184, 211, 250, 269
	E	1-3	58-65	285-300	133, 180, 184, 250, 252, 259, 269
	F	1-3	59-65;179-182	285-300	52, 58, 123, 184, 245, 259, 266, 269
hPNP/**45b**	A	1-2		285-300	11, 22, 67, 122, 168, 179, 184, 186, 211, 252, 254, 258, 265
hPNP/**45q**	A	1-3		285-300	11, 64, 123, 126, 168, 179, 181, 184, 244, 250, 252, 254, 257, 258, 265, 269
hPNP/**45i**	A	1-2		288-300	3, 8, 22, 123, 179, 287
	B	1-3		287-300	180, 184, 185, 250, 253, 265, 286
	C	1-3		286-300	8, 22, 123, 180, 185, 186
hPNP/**45n**	A	1-3		285-300	58, 64, 168, 179

Table S4: Residues missing in crystallographic model of *Mt*PNP.

**Structure**	**Chain**	**N-terminus**	**other**	**C-terminus**	**missing side-chains**

*Mt*PNP/**18c**	A				13
	B	1-6		267-268	13, 267
	C	1-6	63-68		
*Mt*PNP/**45b**	A	1-6	63-67		7, 61
	B	1-5	62-69		7
*Mt*PNP/**45q**	A	1-6			10
	B	1-6			
	C	1-6			256

Table S5: Subunit-subunit interface analysis in crystal structures of hPNP.

**Accessible surface area for interaction of individual monomers in Å^2**				
**Structure**	**Molecule A-B**	**Molecule B-C**	**Molecule C-A**	**Average**
hPNP-**18c***	1188.2/1179.6	1212.5/1222.5	1166.2/1173.4	1189.0/1191.8
hPNP-**45b**	1155			1155
hPNP-**45q**	1141.8			1141.8
hPNP-**45i**	1249.4	1214.3	1213.2	1225.6
hPNP-**45n**	1201.8			1201.8
**Average**				**1187.24**

* Second values given are for molecules in the other trimer in the asymmetric unit (D-E, E-F, F-D)

Table S6: Subunit-subunit interface analysis in crystal structures of *Mt*PNP.

**Accessible surface area for interaction of individual monomers in Å2**				
**Structure**	**Molecule A-B**	**Molecule B-C**	**Molecule C-A**	**Average**
*Mt*PNP-**18c**	870.4	766.8	766.8	802.0
*Mt*PNP-**45b****	863.7	852.0		857.9
*Mt*PNP-**45q**	914.5	914.8	924.8	918.9
**Average**				**882.4**

***Mt*PNP-**45b** structure contains two molecules which belong to two different biological trimers. Number in **Molecule A-B** column is for A-A', and value in **Molecule B-C** column is for B-B'.

Table S7: Analysis of residues buried in subunit-subunit interface in crystal structures of hPNP.

**Percentage of total accessible surface area buried in subunit-subunit interaction for residues 133-166**					
Residue	**18c**	**45b**	**45q**	**45i**	**45n**

ARG 133	38.5	37.25	27.25	33.45	32.84
ASP 134	94.1	99.34	98.32	94.71	96.52
HIS 135	94.79	93.18	91.84	94.48	92.81
ILE 136	100	100	96.98	100	97.48
ASN 137	50.05	56.53	59.57	56.37	53.94
LEU 138	1	1.987	1.73	0.76	1.33
PRO 139	0	0	0.00	0	0
GLY 140	58.49	66.52	66.02	61.53	62.41
PHE 141	89.98	95.15	90.9	90.31	90.91
SER 142	79.35	77.36	77.82	66.53	78.39
GLY 143	78.27	78.21	77.24	74.66	79.35
GLN 144	0.00	0.00	0	0	0
ASN 145	80.24	82.12	82.1	82.43	80.61
PRO 146	0.00	0.00	0	0	0
LEU 147	56.40	58.64	58.124	68	58.89
ARG 148	65.84	59.06	51.1	58.48	61.72
GLY 149	81.19	71.86	72.12	83.84	69.21
PRO 150	7.49	23.10	15.26	5	16.45
ASN 151	0.00	2.95	0	0	0
ASP 152	0.00	0.00	0	0	0
GLU 153	0.00	0.00	0	0.8	0
ARG 154	0.00	0.00	0	0	0
PHE 155	0.00	0.00	0	0	0
GLY 156	0.00	0.00	0	0	0
ASP 157	0.00	0.00	23.55	36.86	35.18
ARG 158	49.81	52.51	52.64	58.2	55.42
PHE 159	72.62	72.08	69.14	70.64	62.94
PRO 160	61.24	60.45	56.72	64.72	57.1
ALA 161	82.51	71.54	68.48	81.94	83.14
MET 162	91.83	95.84	95.99	92.86	94.01
SER 163	79.11	81.27	76.22	79.24	79.38
ASP 164	34.73	12.24	9.79	31.9	35.81
ALA 165	13.74	11.58	10.93	4.9	10.28
TYR 166	2.09	1.88	5.41	1.65	0
ASP 167	0.00	0.00	0	0	0
ARG 168	0.00	0.00	0	0	0

Table S8: Analysis of residues buried in subunit-subunit interface in crystal structures of *Mt*PNP.

**Percentage of total accessible surface area buried in subunit-subunit interaction for residues 138-160**			
Residues	**18c**	**45b**	**45q**
ASP 138	80.65	75.11	80.46
HIS 139	99.39	99.34	100.00
LEU 140	98.55	99.29	99.63
ASN 141	58.49	58.39	59.54
LEU 142	23.06	27.80	27.92
THR 143	0	0.00	0.00

ALA 144	57.87	64.34	56.81
ARG 145	9.28	7.89	8.20
SER 146	62.9	63.51	66.77
PRO 147	0	0.00	0.00
LEU 148	73.97	75.07	82.52
VAL 149	14.68	24.41	27.94
GLY 150	58.2	48.32	81.43
GLY 151	0.49	0.00	27.40
GLU 152	10.07	9.76	10.65
PHE 153	66.3	73.98	67.67
VAL 154	47.22	52.60	61.12
ASP 155	75.08	64.22	68.76
LEU 156	99.81	98.29	100.00
THR 157	83.78	45.58	85.20
ASP 158	6.51	3.40	7.34
ALA 159	39.39	29.32	37.38
TYR 160	6.49	13.89	27.12

1.4 Supplementary figures

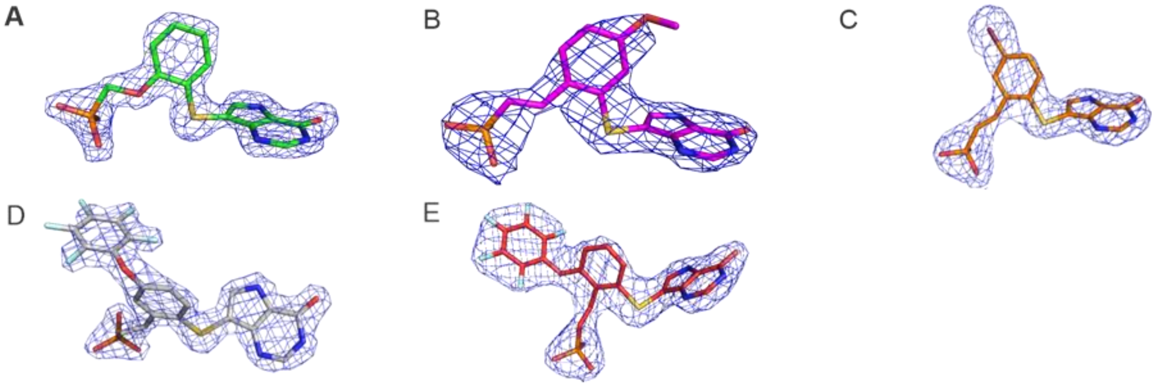

Figure S1: Structure of compounds **18c** (A), **45b** (B), **45q** (C), **45i** (D), and **45n** (E) bound to hPNP with their 2*Fo*-*Fc* map contoured at 1.6 σ for each inhibitor is shown as a blue mesh. Carbon atoms are green (A), magenta (B), orange (C), gray (D), and red (E), nitrogen atoms are blue, oxygen red, phosphorus orange and sulfur yellow.

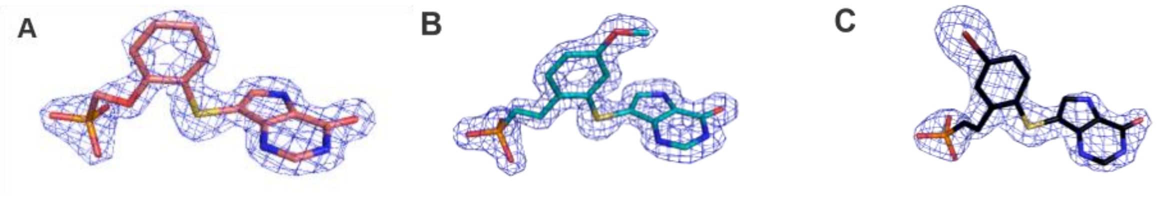

Figure S2: Structure of compounds **18c** (A), **45b** (B), and **45q** (C) bound *Mt*PNP with their 2*Fo*-*Fc* map contoured at 1.6 σ for each inhibitor is shown as a blue mesh. Carbon atoms are salmon (A), cyan (B) and black (C), oxygen atoms red, nitrogen blue and phosphorus orange and sulfur yellow.

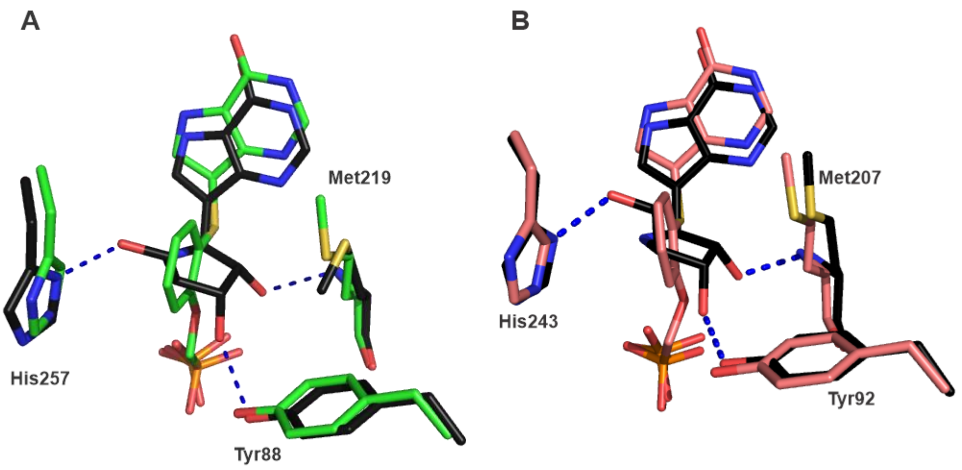

Figure S3: Comparison between compound **18c** and ImmucilinH binding in hPNP **(A)** and *Mt*PNP **(B)**. Superposition of compound **18c** (green carbon atoms) and ImmH (black carbon atoms) bound to hPNP (**A**, 1RR6, 10.1074/jbc.C400068200) and *Mt*PNP (**B**, 1G2O, 10.1021/bi010585p). Overall, binding of these two inhibitors is similar in both hPNP and *Mt*PNP. All the direct and water-mediated hydrogen bonds are conserved in ImmH structures with three additional hydrogen bonds formed between protein residues and central sugar moiety. Inhibitor and residues interacting differently are shown as sticks. Direct hydrogen bonds are shown as blue-dashed lines, water-mediated hydrogen bonds with cyan-dashed lines, oxygen atoms are red, nitrogen blue, phosphate yellow, sulphate orange and waters are shown as red spheres.

1.5 Additional comparisons

Comparison of human PNP and MtPNP complex structures. We compared the structures of **18c** and **45b**, bound to both hPNP and *Mt*PNP to understand structural basis for compound selectivity.

Overall, active sites of hPNP and *Mt*PNP are similar with conserved residues occupying the identical positions in both proteins. There are minor differences in position of the loop 31-36 (hPNP) and 34-39 (*Mt*PNP) but it does not affect formation of the hydrogen bond between Ser33/36 and phosphonate group of the ligand. The large loop preceding helix$_{257\text{-}267}$/helix243$_{\text{-}253}$ 241-256 (hPNP) or 231-242 (*Mt*PNP) have a flexible position (**Figure S3**). Single amino acid substitution within the active site is position Phe200 vs. Tyr188 in hPNP and *Mt*PNP, respectively. In hPNP, carbonyl of Ile 246 interacts with the purine moiety of inhibitors through two-water-mediated hydrogen bond (Figure 6 A-E). Analogous interaction in *Mt*PNP is present as a single-water mediated hydrogen bond with Ala234 carbonyl (Figure 7A-C).

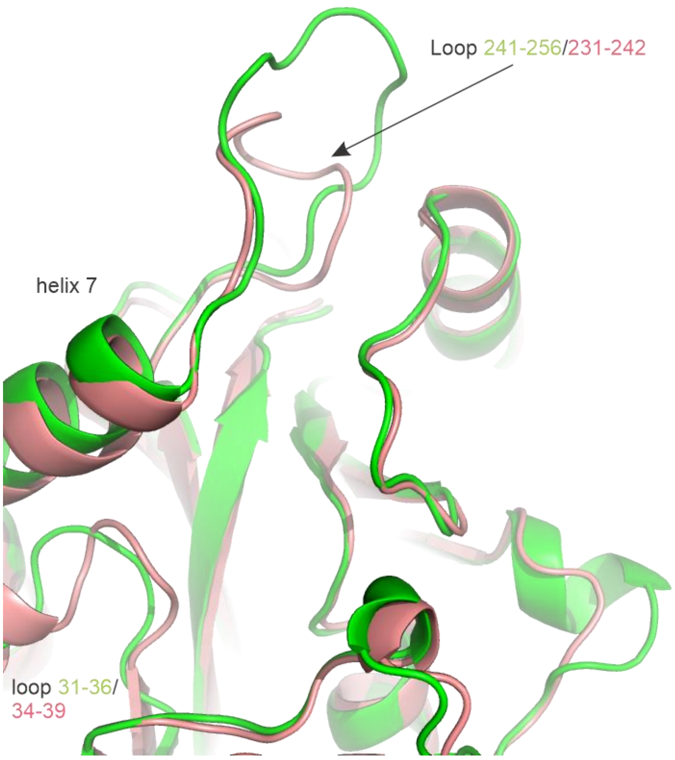

Figure S4: Overall comparison of the active sites of hPNP (green) and *Mt*PNP (salmon) in complex with compound **18c**. Protein is shown in cartoon representation while inhibitors are omitted from this view. Loops that differ in the active sites are labeled.

Inhibitor **18c** binds to both enzymes with similar affinity (IC_{50} 0.022 vs 0.030 µM). Structural comparison shows only a minor difference in the position of the central phenyl moiety of the inhibitor and corresponding change in linker conformation (Figure 8A). Consequently, minor shifts in positions of His257/243 and Phe159/153 (Figure 8B) follow the ligand shift to maintain interactions. Position of the purine and phosphonate moieties is fully conserved (Figure 8A).

Inhibitor **45b** has almost 4-fold higher affinity for *Mt*PNP then hPNP. Similarly to **18c**, differences in the position of the central phenyl moiety, linker and corresponding interacting residues are observed (Figure 8B). Additionally, alteration in orientation of methoxy group substitution at the position 5 of the central phenyl moiety can be noticeable. Resulting difference in the affinity is probably due to the presence more favorable Pi-stacking interactions and/or lack of clashes of the methoxy group with Tyr188 in *Mt*PNP compared to hPNP (Figure 7).

Positions of purine and phosphonate moieties of inhibitor **45q** are conserved, however, presence of the bromide substitution leads to changes in the position of the central phenyl moiety as well as the linker. In hPNP, there is a minor shift in the position of the C-terminal helix that accommodates the shift in the position of the central phenyl moiety. Bromide substituent affects the position of His257 sidechain that is disordered in hPNP. Minor changes in the position of Phe159/153 from the neighboring molecule, as well as minor changes in the position of Phe200/Tyr188 are also observed (Figure 8C). The changes in the position of the central phenyl moiety with bromide substituent leads to 2-3-fold decrease in affinity of **45q** towards hPNP compared to *Mt*PNP.

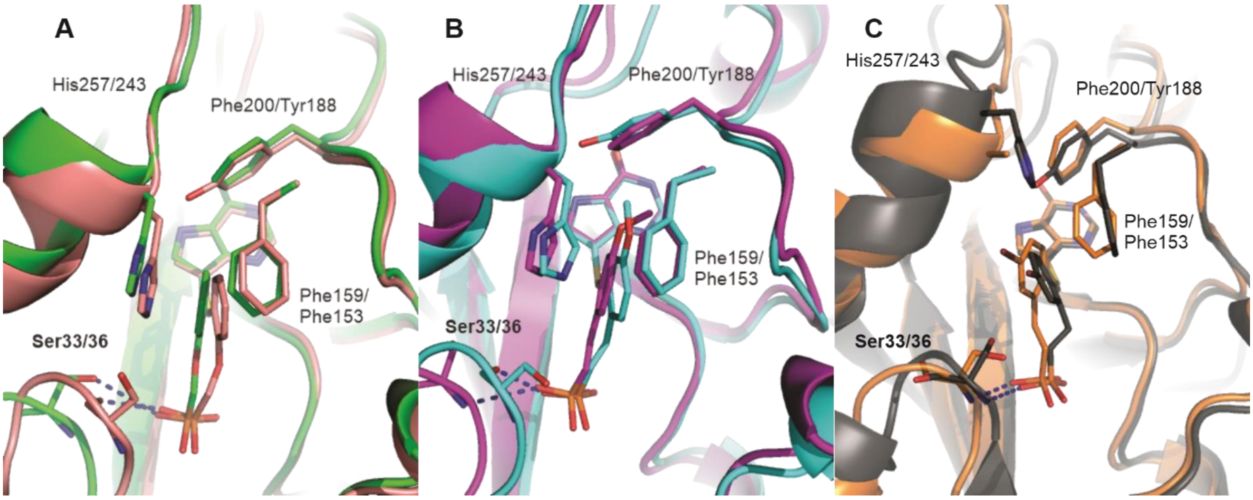

Figure S5: Comparison of interactions of inhibitors with active sites of hPNP and *Mt*PNP. (**A**) overlay of **18c** bound to hPNP, PDB 7ZSL (green) and *Mt*PNP, PDB 7ZSQ (salmon); (**B**) overlay of **45b** bound to hPNP, PDB 7ZSM (magenta) and *Mt*PNP, PDB 8C25 (cyan); (**C**) overlay of **45q** bound to hPNP, PDB 7ZSN (orange) and *Mt*PNP, PDB 7ZSR (dark gray). Relevant residues are shown as sticks and residues that form hydrogen bonds are labeled bold, other residues are labeled with normal font. Hydrogen bonds are shown as blue-dashed lines. Nitrogen atoms are shown with blue, oxygen with red and phosphorus with orange.Further, we analyzed the subunit-subunit interaction in the region of the enzyme's active site. Results for each active site can be found in Table S5. Even though the position of Phe159/153 is relatively conserved, there are differences in the loop preceding this residue. In hPNP, the loop is formed of residues 133-165, while in *Mt*PNP it is composed of residues 136-168. Major structural differences can be found in the region preceding Phe159/153 residues which is seven residues longer in hPNP compared to *Mt*PNP (Figure 9). Average surface area of hPNP subunit-subunit interface is 25% larger then *Mt*PNP surface area (1187.24 Å^{2}compared to 882.4 Å^2) (Table S6). Analysis if interacting residues by PISA[52] revealed that residues that participate in the formation of the subunit-subunit interface near the active site are relatively conserved among analyzed hPNP structures (Table S7). On the other hand, variability in involvement of individual residues in subunit-subunit interaction interface can be noticed for *Mt*PNP structures (Table S7). Structural variability and flexibility of this region affect interaction with neighboring subunit and thus might contribute to difference in affinity of the inhibitors towards *Mt*PNP compared to hPNP.

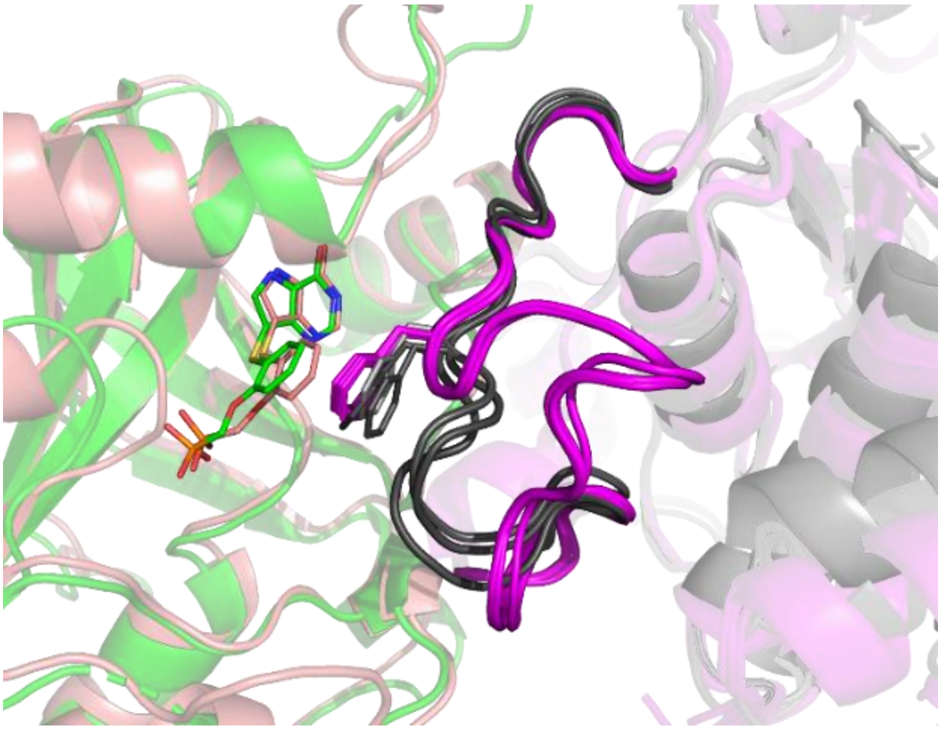

Figure S6: Comparison of the loop surrounding Phe159/153 of the neighboring molecule. On the left, active site regions of both hPNP, PDB 7ZSL (green) and *Mt*PNP, PDB 7ZSQ (salmon) in complex with inhibitor **18c**. On the right, overlay of all the neighboring molecules with the Phe159/153-surrounding the loop. hPNP molecules are shown in magenta, *Mt*PNP molecules in dark gray.

2 Pharmacology

Table S9. Cytotoxicity of PNP inhibitors towards off-target cells represented by non-T-cell-derived tumor cell lines, resting PBMC and purified T-lymphocytes. Data are expressed as % viable cells compared to untreated controls.

**Compound**	**HL-60** (10 µM) [%]	**HeLaS3** (10 µM) [%]	**HepG2** (10 µM) [%]	**PBMC** (50 µM) [%]	**T-cells** (50 µM) [%]
**7a**	85	70	92	89	ND*
**7b**	ND*	ND*	ND*	ND*	ND*
**7c**	ND*	ND*	ND*	ND*	ND*
**7d**	60	91	84	80	ND*
**7e**	49	108	104	36	ND*
**7f**	ND*	ND*	ND*	ND*	ND*
**13a**	89	87	89	89	ND*
**13b**	82	87	92	88	ND*
**18a**	90	110	86	67	ND*
**18b**	85	127	83	85	ND*
**18c**	82	107	85	76	ND*
**21**	81	95	78	101	ND*
**23**	75	133	95	108	ND*
**45a**	73	84	96	96	ND*
**45b**	89	104	97	94	ND*
**45c**	81	109	100	114	ND*
**45d**	84	105	104	123	ND*
**45e**	73	102	98	119	ND*
**45f**	89	70	100	ND*	ND*
**45g**	84	52	109	ND*	ND*
**45h**	77	62	102	ND*	ND*
**45i**	93	62	103	ND*	ND*
**45j**	96	56	107	ND*	ND*
**45k**	61	107	82	92	ND*
**45l**	96	97	83	51	ND*
**45m**	114	93	88	85	ND*
**45n**	92	60	111	ND	ND*
**45o**	82	98	92	100	ND*
**45p**	72	93	87	98	ND*
**45q**	74	100	82	80	ND*
**49a**	100	92	93	ND	ND*
**49b**	100	92	99	ND	ND*
**50a**	105	96	95	58	76
**50b**	99	80	84	54	65
**51a**	107	98	97	70	83
**51b**	ND*	ND*	ND*	ND*	ND*
**Forodesine**	107	98	90	84	81

*ND – not determined.

3 Chemistry

3.1 List of inhibitors

Table S10. List of prepared inhibitors with corresponding smiles string.

Compound	Smiles
**7a**	O=C1NC=Nc2c1[nH]cc2Sc1ccccc1
**7b**	Cc(cccc1)c1Sc1c[nH]c2c1N=CNC2=O
**7c**	O=C1NC=Nc2c1[nH]cc2Sc(cccc1)c1Br
**7d**	Oc(cccc1)c1Sc1c[nH]c2c1N=CNC2=O
**7e**	OCc(cccc1)c1Sc1c[nH]c2c1N=CNC2=O
**7f**	O=C1NC=Nc2c1[nH]cc2Sc1cccc(Br)c1
**13a**	OP(COCCSc1c[nH]c2c1N=CNC2=O)(O)=O
**13b**	OP(CCOCCSc1c[nH]c2c1N=CNC2=O)(O)=O
**18a**	OP(Cc(cccc1)c1Sc1c[nH]c2c1N=CNC2=O)(O)=O
**18b**	OP(/C=C/c(cccc1)c1Sc1c[nH]c2c1N=CNC2=O)(O)=O
**18c**	OP(COc(cccc1)c1Sc1c[nH]c2c1N=CNC2=O)(O)=O
**21**	OP(c1cccc(Sc2c[nH]c3c2N=CNC3=O)c1)(O)=O
**23**	OP(COCc(cccc1)c1Sc1c[nH]c2c1N=CNC2=O)(O)=O
**45a**	Oc1cc(Sc2c[nH]c3c2N=CNC3=O)c(/C=C/P(O)(O)=O)cc1
**45b**	COc1cc(Sc2c[nH]c3c2N=CNC3=O)c(/C=C/P(O)(O)=O)cc1
**45c**	CC(C)Oc1cc(Sc2c[nH]c3c2N=CNC3=O)c(/C=C/P(O)(O)=O)cc1
**45d**	OP(/C=C/c(ccc(Oc(c(F)c(c(F)c1F)F)c1F)c1)c1Sc1c[nH]c2c1N=CNC2=O)(O)=O
**45e**	OP(/C=C/c(ccc(OCc(c(F)c(c(F)c1F)F)c1F)c1)c1Sc1c[nH]c2c1N=CNC2=O)(O)=O
**45f**	Oc(cc1)cc(/C=C/P(O)(O)=O)c1Sc1c[nH]c2c1N=CNC2=O
**45g**	COc(cc1)cc(/C=C/P(O)(O)=O)c1Sc1c[nH]c2c1N=CNC2=O
**45h**	CC(C)Oc(cc1)cc(/C=C/P(O)(O)=O)c1Sc1c[nH]c2c1N=CNC2=O
**45i**	OP(/C=C/c(cc(cc1)Oc(c(F)c(c(F)c2F)F)c2F)c1Sc1c[nH]c2c1N=CNC2=O)(O)=O
**45j**	OP(/C=C/c(cc(cc1)OCc(c(F)c(c(F)c2F)F)c2F)c1Sc1c[nH]c2c1N=CNC2=O)(O)=O
**45k**	Oc1cccc(Sc2c[nH]c3c2N=CNC3=O)c1/C=C/P(O)(O)=O
**45l**	COc1cccc(Sc2c[nH]c3c2N=CNC3=O)c1/C=C/P(O)(O)=O
**45m**	CC(C)Oc1cccc(Sc2c[nH]c3c2N=CNC3=O)c1/C=C/P(O)(O)=O
**45n**	OP(/C=C/c(c(Oc(c(F)c(c(F)c1F)F)c1F)ccc1)c1Sc1c[nH]c2c1N=CNC2=O)(O)=O
**45o**	OP(/C=C/c(c(OCc(c(F)c(c(F)c1F)F)c1F)ccc1)c1Sc1c[nH]c2c1N=CNC2=O)(O)=O
**45p**	OP(/C=C/c(ccc(Br)c1)c1Sc1c[nH]c2c1N=CNC2=O)(O)=O
**45q**	OP(/C=C/c(cc(cc1)Br)c1Sc1c[nH]c2c1N=CNC2=O)(O)=O
**49a**	OP(COc(ccc(F)c1)c1Sc1c[nH]c2c1N=CNC2=O)(O)=O
**49b**	COc1cccc(Sc2c[nH]c3c2N=CNC3=O)c1OCP(O)(O)=O
**50a**	CC(C)OC([C@H](C)NP(COc(cccc1)c1Sc1c[nH]c2c1N=CNC2=O)(Oc1ccccc1)=O)=O
**50b**	CC(C)OC([C@H](Cc1ccccc1)NP(COc(cccc1)c1Sc1c[nH]c2c1N=CNC2=O)(Oc1ccccc1)=O)=O
**51a**	CC(C)OC([C@H](C)NP(COc(cccc1)c1Sc1c[nH]c2c1N=CNC2=O)(N[C@@H](C)C(OC(C)C)=O)=O)=O
**51b**	CC(C)OC([C@H](Cc1ccccc1)NP(COc(cccc1)c1Sc1c[nH]c2c1N=CNC2=O)(N[C@@H](Cc1ccccc1)C(OC(C)C)=O)=O)=O
**Forodesine**	OC[C@H]([C@@H](C1O)O)N[C@H]1C1C(N=CNC2=O)=C2N=C1

3.2 Copies of the NMR and UPLC/MS Spectra

3.2.1 General information

NMR spectra are present as ^{1}H, $^{13}C\{^{1}H\}$, $^{19}F\{^{1}H\}$, and $^{31}P\{^{1}H\}$. LC/MS spectra are present as absorbance of PDA, at 254 nm, and molecular mass peak. Purity of compounds is calculated from peaks at 254 nm and

elution times between 1 – 7 min. If purity of a compound is <95%, it was caused by solubility issues that some compounds exhibited and prevented complete purification of the sample.

3.2.2 4-(Benzyloxy)-5*H*-pyrrolo[3,2-*d*]pyrimidine (2)

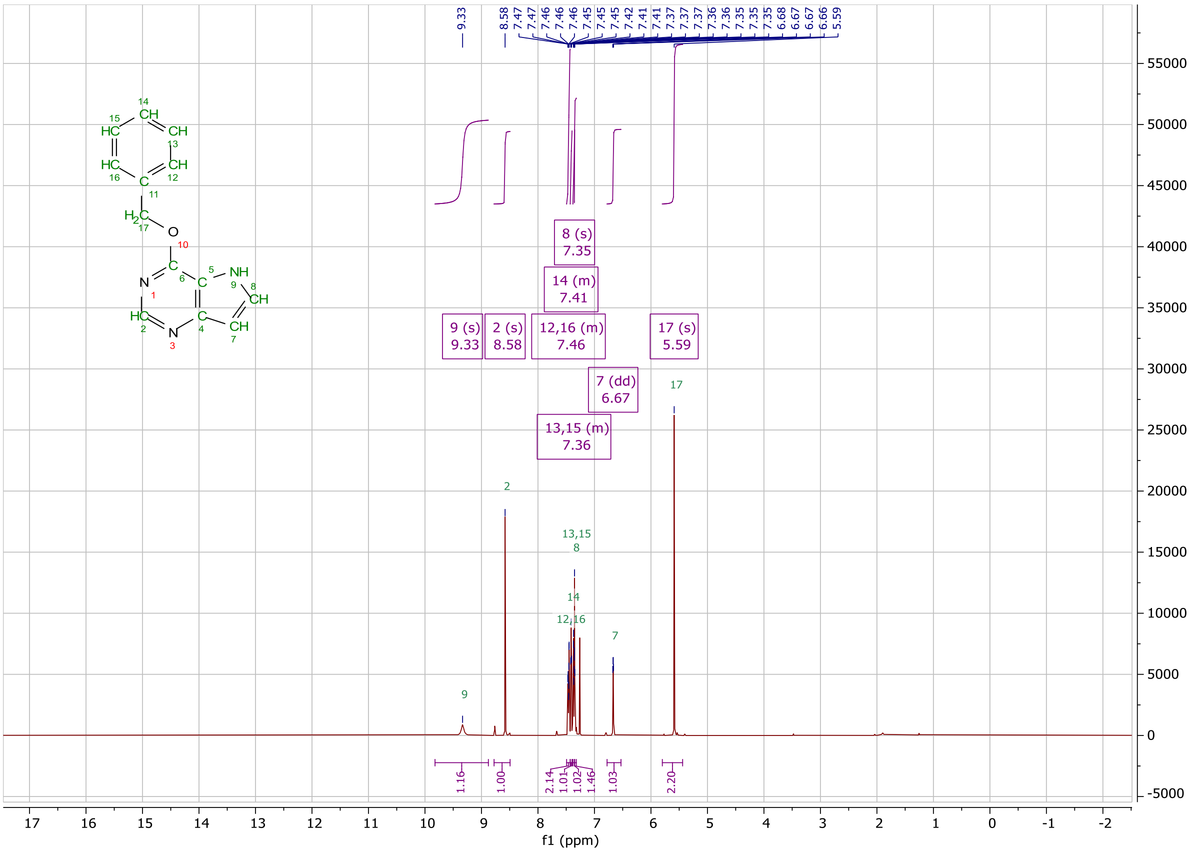

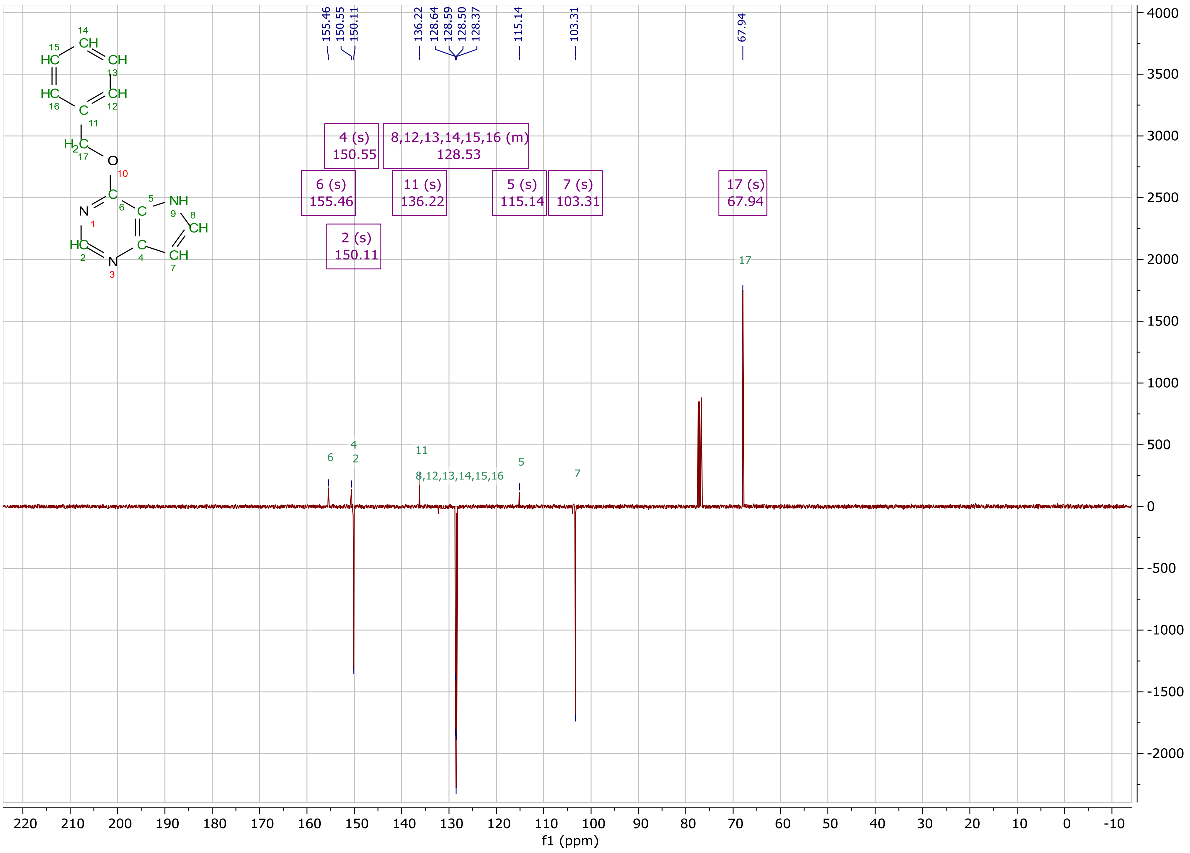

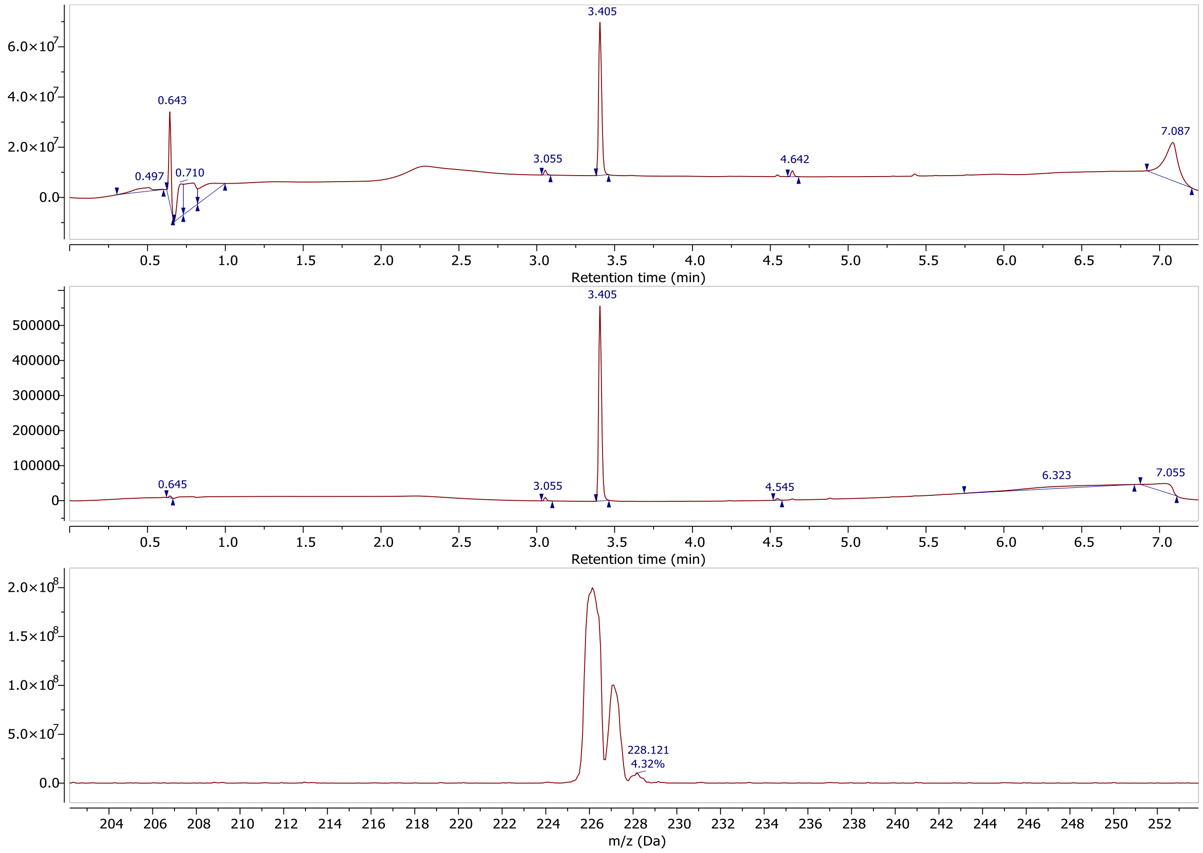

3.2.3 4-(Benzyloxy)-7-iodo-5-((2-(trimethylsilyl)ethoxy)methyl)-5*H*-pyrrolo[3,2-*d*]pyrimidine (4)

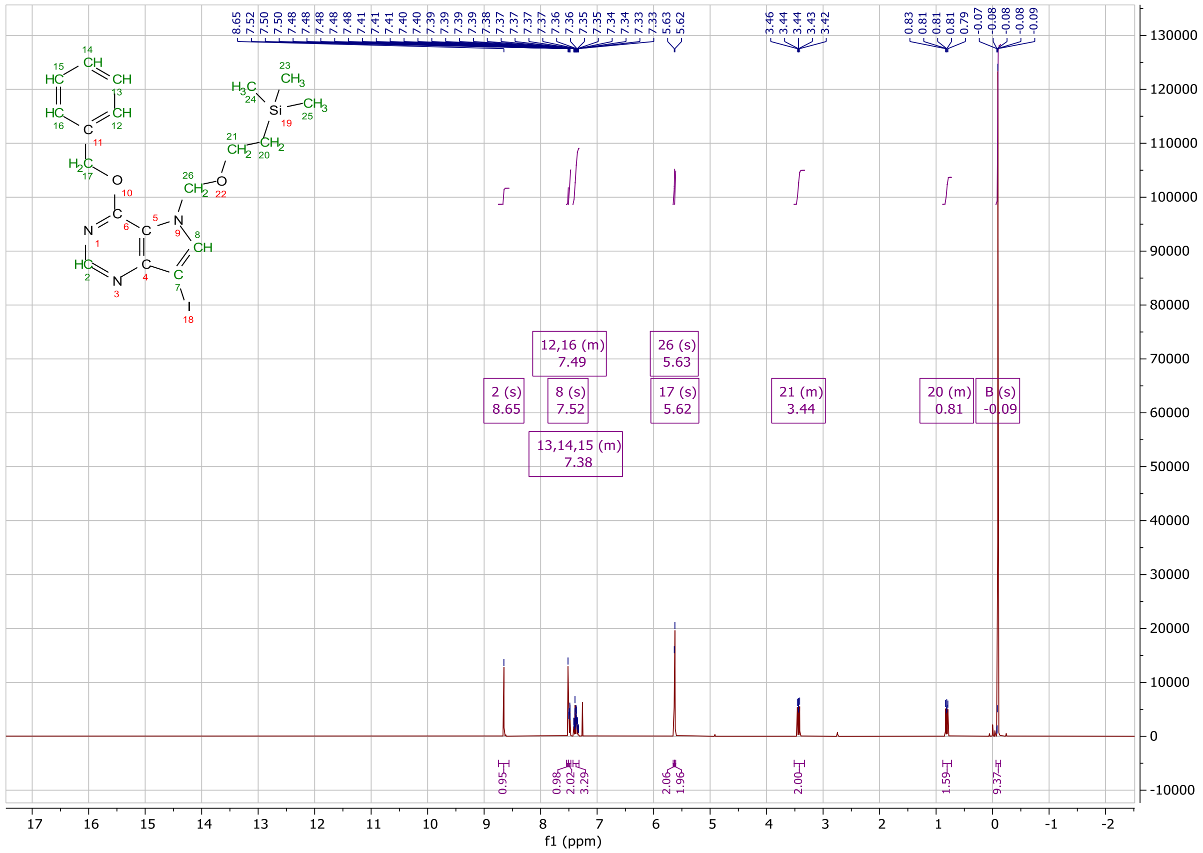

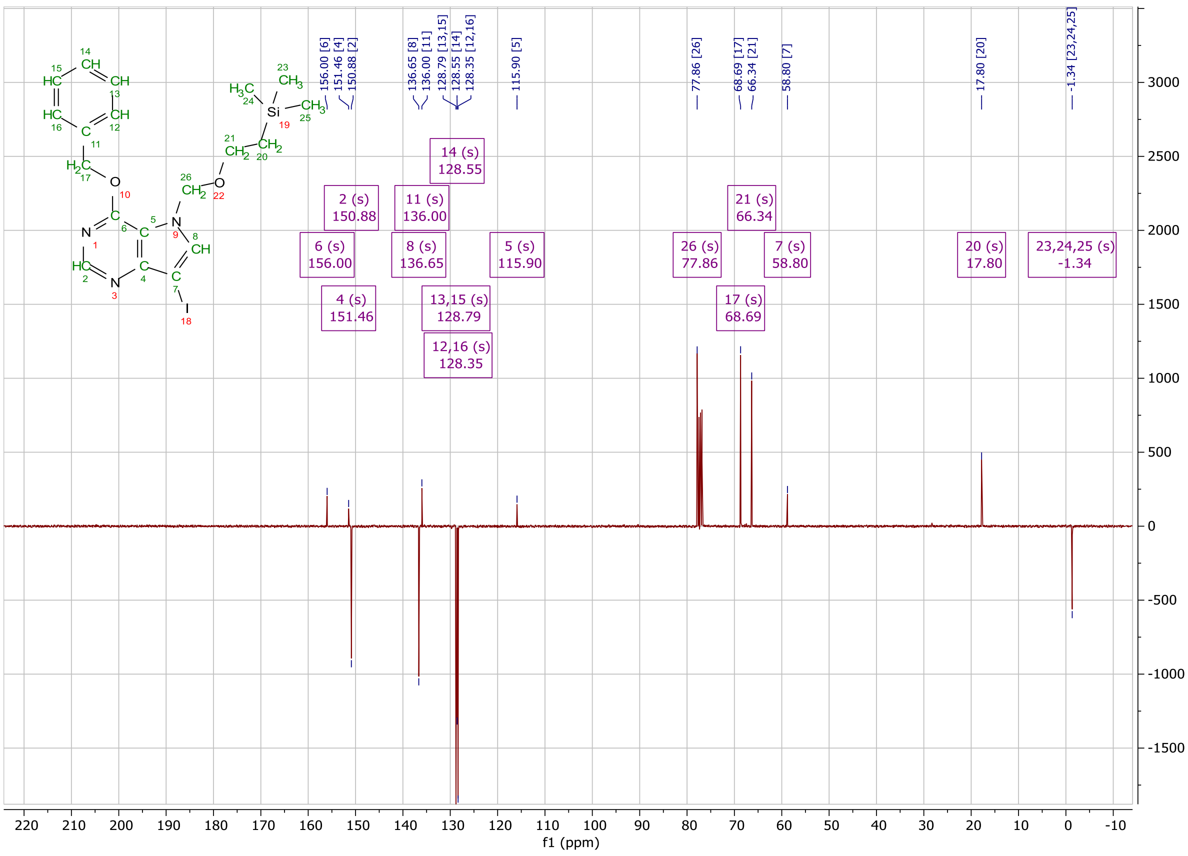

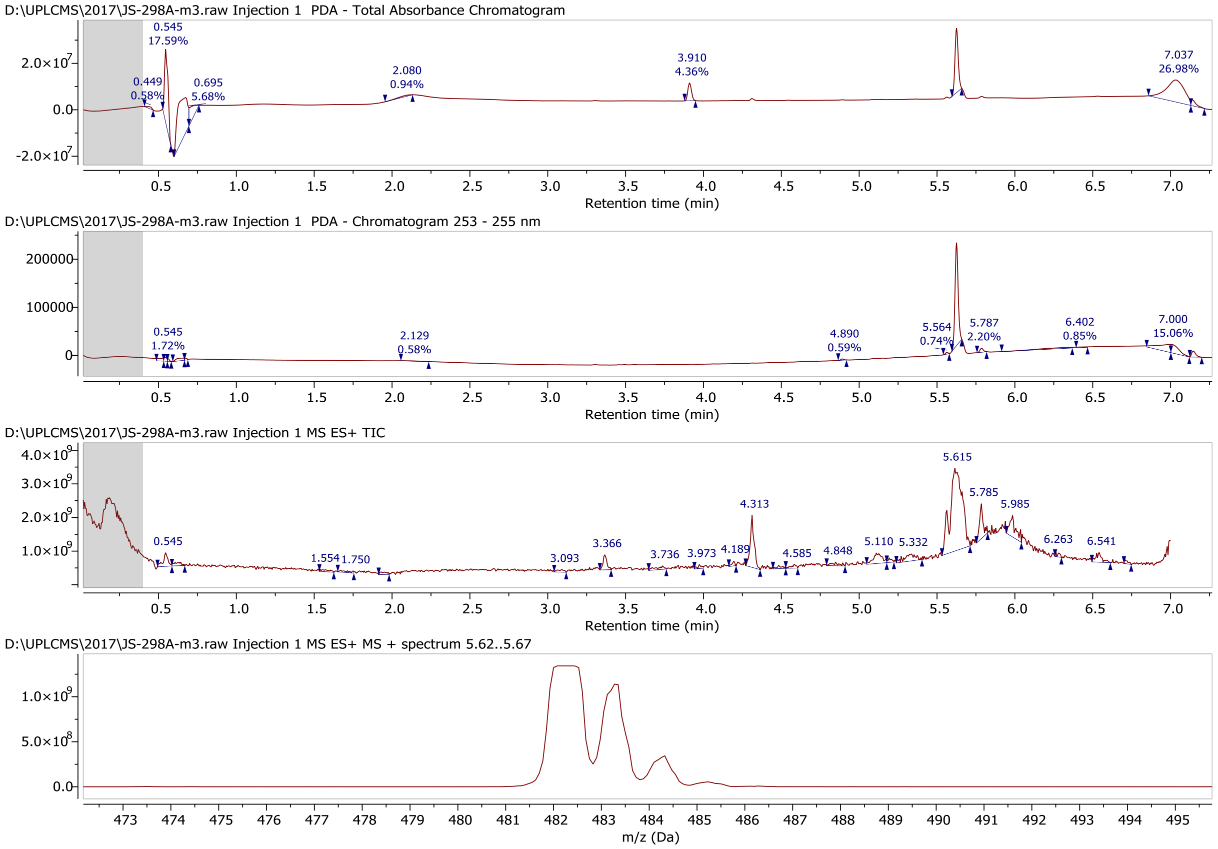
D:\UPLCMS\2017\JS-298A-m3.raw Injection 1 PDA - Total Absorbance Chromatogram
D:\UPLCMS\2017\JS-298A-m3.raw Injection 1 PDA - Chromatogram 253 - 255 nm
D:\UPLCMS\2017\JS-298A-m3.raw Injection 1 MS ES+ TIC
D:\UPLCMS\2017\JS-298A-m3.raw Injection 1 MS ES+ MS + spectrum 5.62..5.67
Retention time (min)
m/z (Da)

3.2.4 4-(Benzyloxy)-7-(phenylthio)-5-((2-(trimethylsilyl)ethoxy)methyl)-5*H*-pyrrolo[3,2-*d*]pyrimidine (6a)

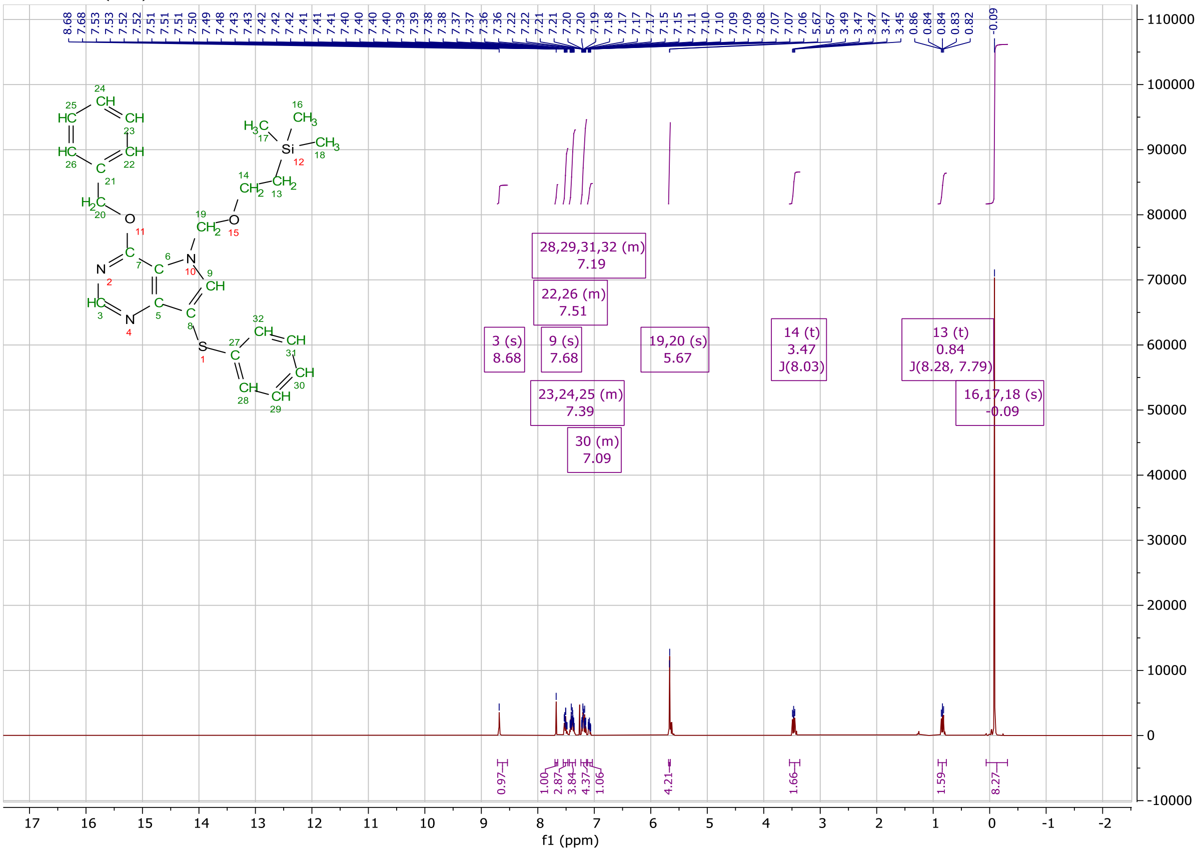

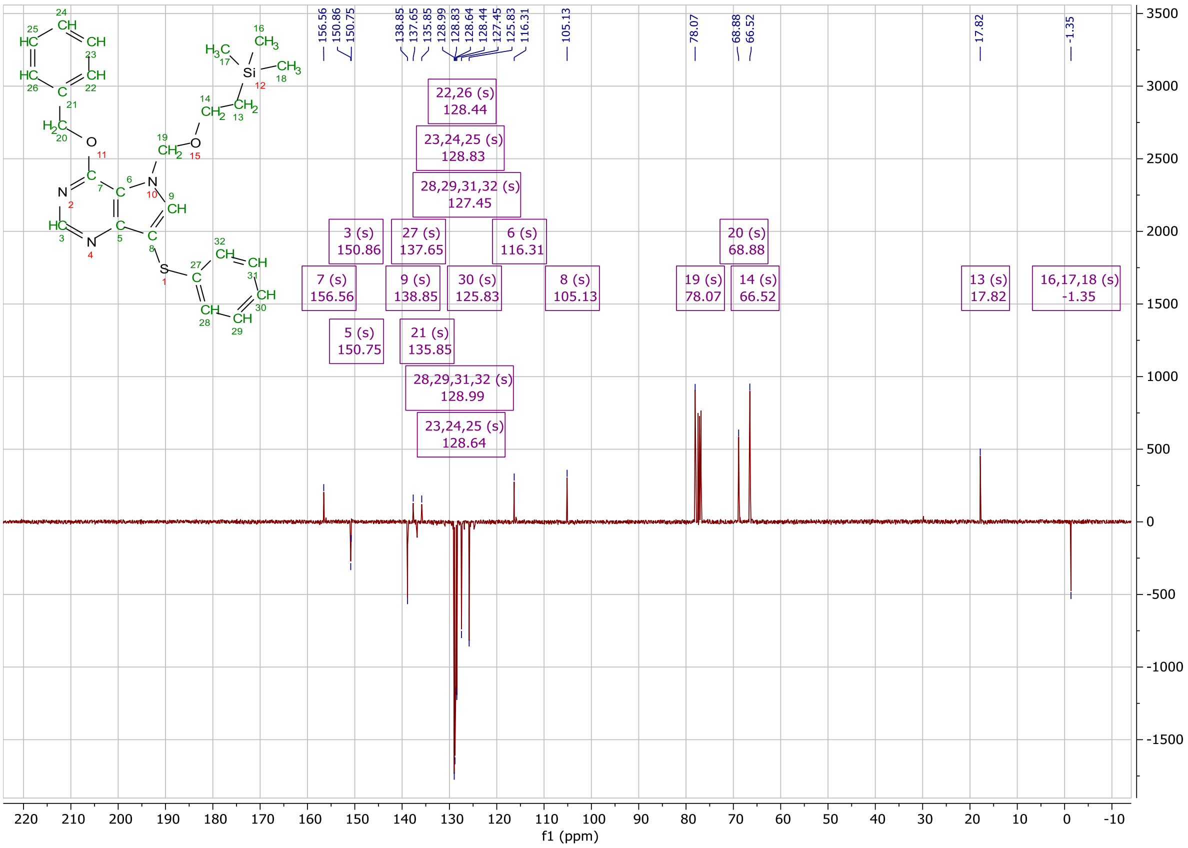

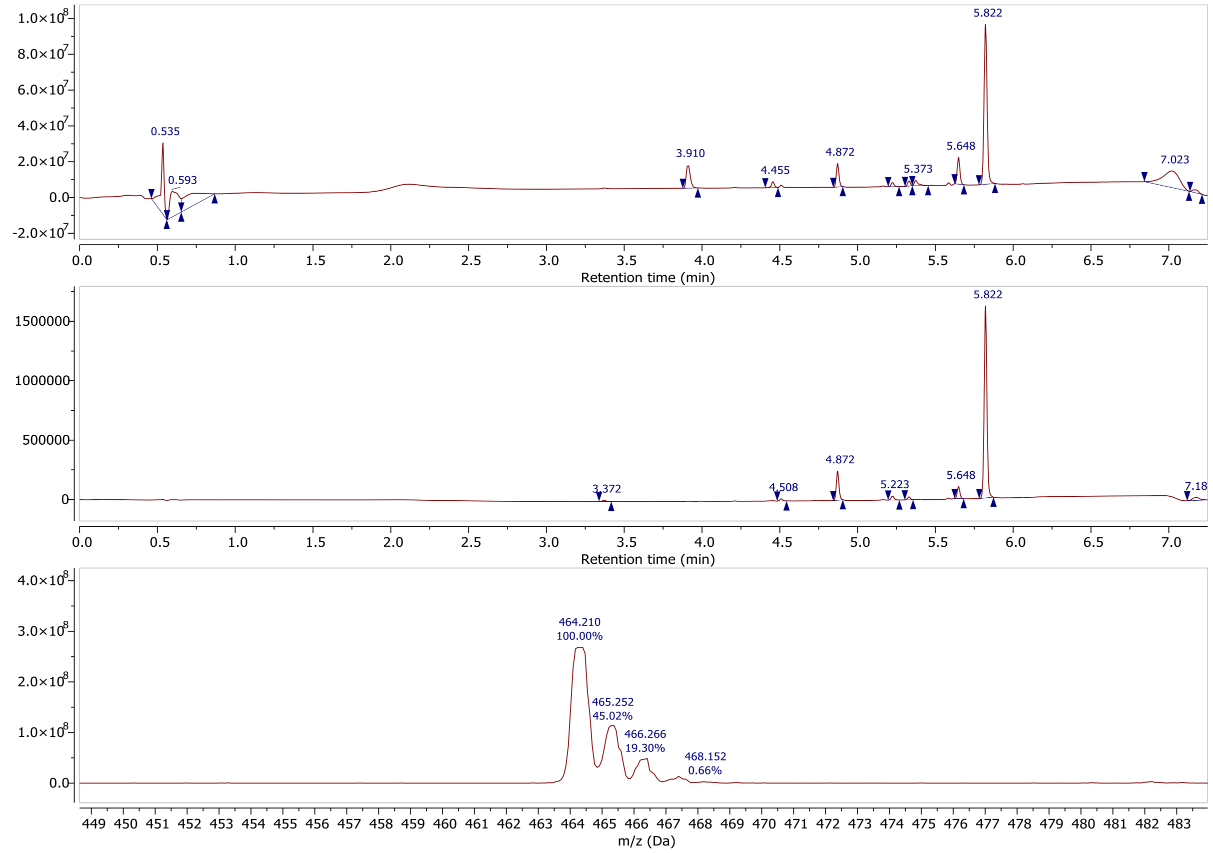

3.2.5 4-(Benzyloxy)-7-((2-methyl)phenylthio)-5-((2-(trimethylsilyl)ethoxy)methyl)-5*H*-pyrrolo[3,2-*d*]pyrimidine (6b)

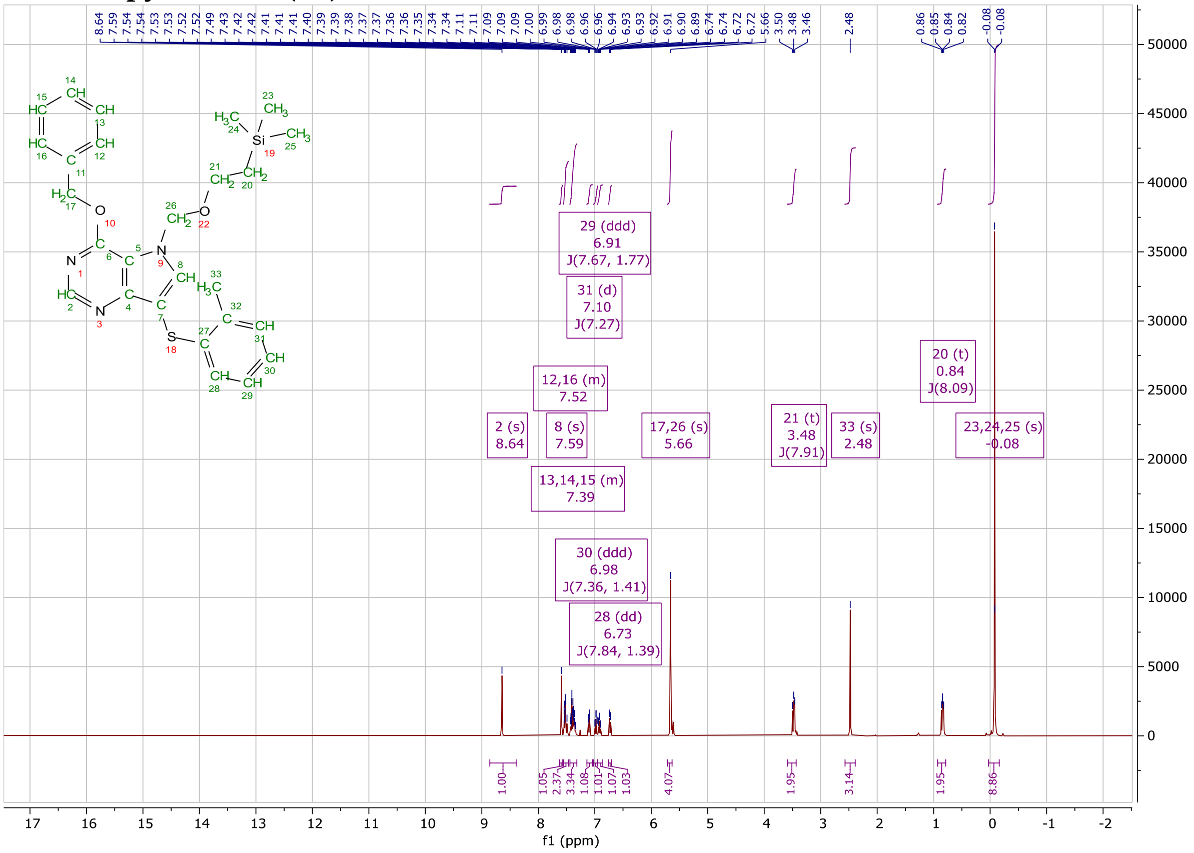

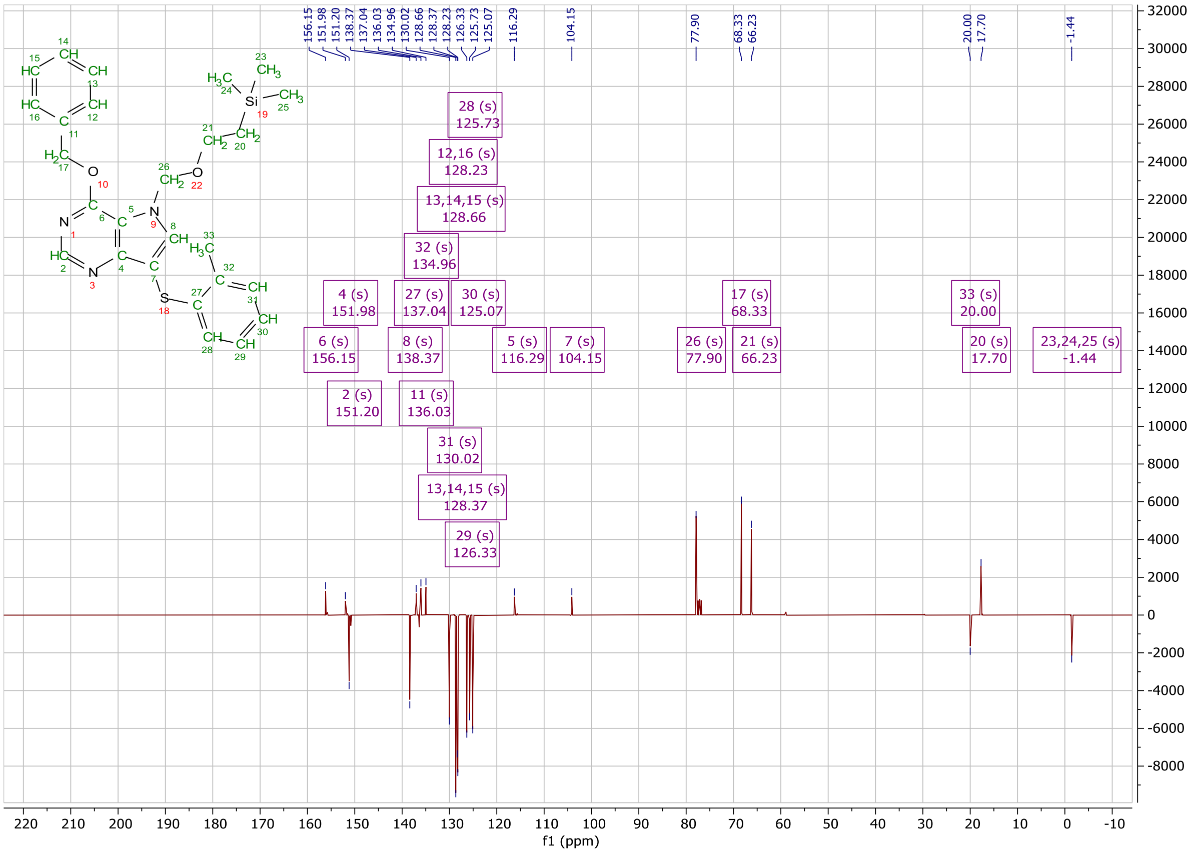
156.15
151.98
151.20
138.37
137.04
136.03
134.96
130.02
128.66
128.37
128.23
126.33
125.73
125.07
116.29
104.15
77.90
68.33
66.23
20.00
17.70
-1.44
28 (s) 125.73
12,16 (s) 128.23
13,14,15 (s) 128.66
32 (s) 134.96
4 (s) 151.98
27 (s) 137.04
30 (s) 125.07
17 (s) 68.33
33 (s) 20.00
6 (s) 156.15
8 (s) 138.37
5 (s) 116.29
7 (s) 104.15
26 (s) 77.90
21 (s) 66.23
20 (s) 17.70
23,24,25 (s) -1.44
2 (s) 151.20
11 (s) 136.03
31 (s) 130.02
13,14,15 (s) 128.37
29 (s) 126.33
f1 (ppm)

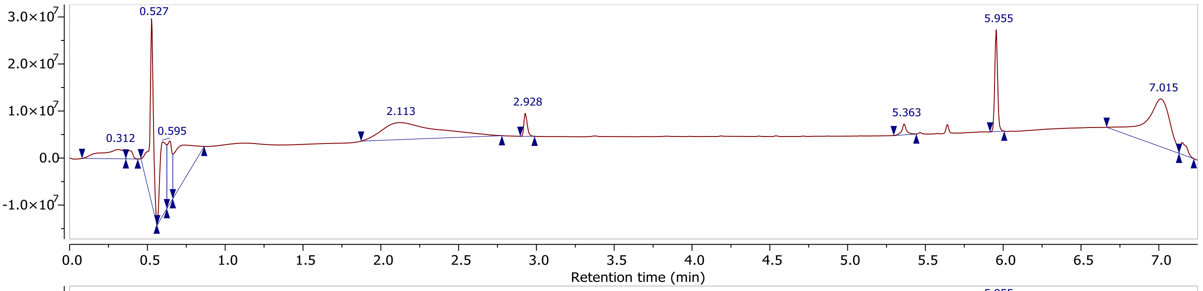
0.527
0.312
0.595
2.113
2.928
5.363
5.955
7.015
Retention time (min)

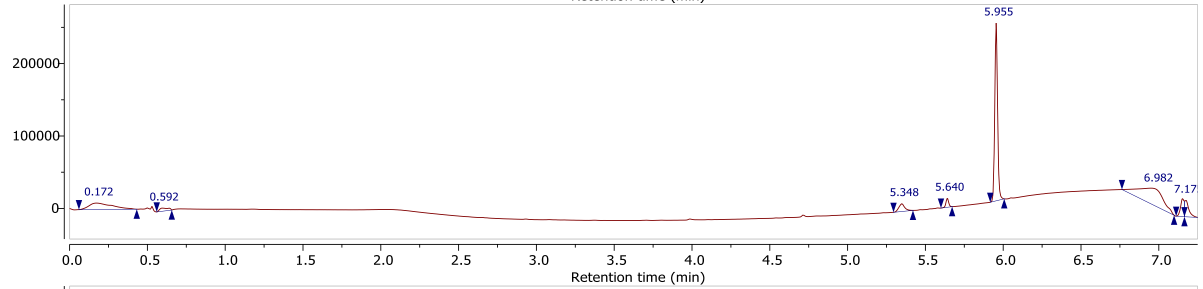
5.955
0.172
0.592
5.348
5.640
6.982
Retention time (min)

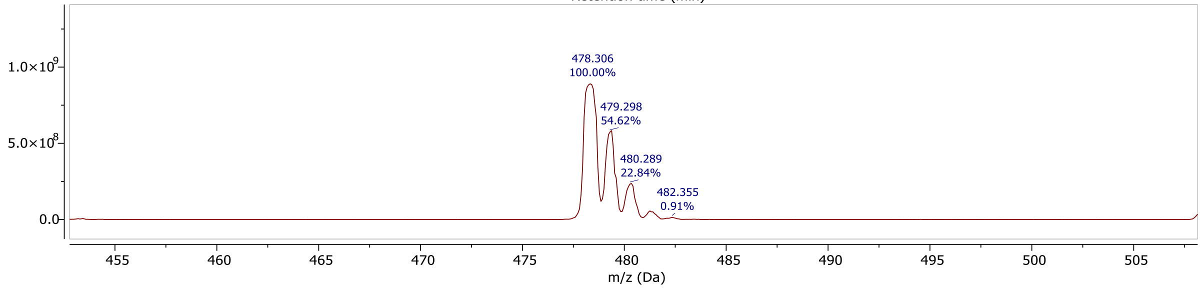
478.306
100.00%
479.298
54.62%
480.289
22.84%
482.355
0.91%
m/z (Da)

3.2.6 4-(Benzyloxy)-7-((2-bromo)phenylthio)-5-((2-(trimethylsilyl)ethoxy)methyl)-5*H*-pyrrolo[3,2-*d*]pyrimidine (6c)

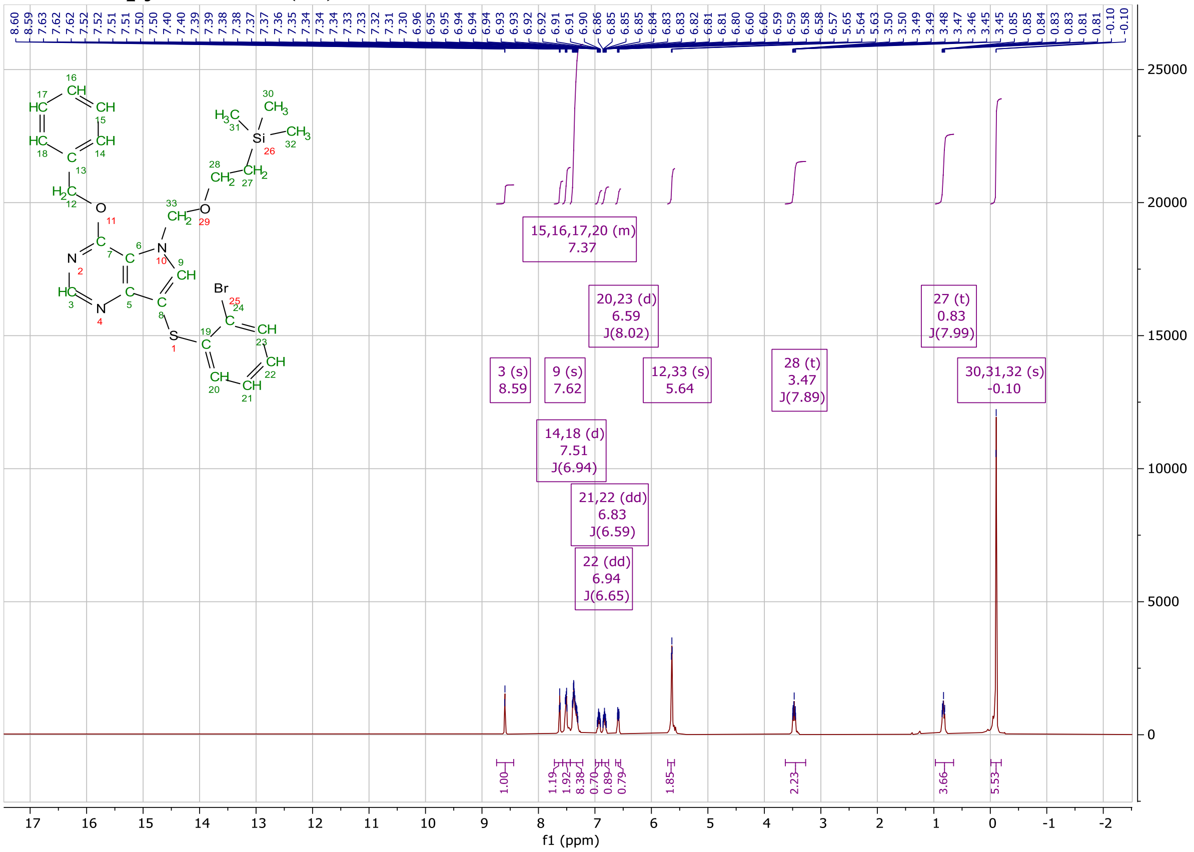

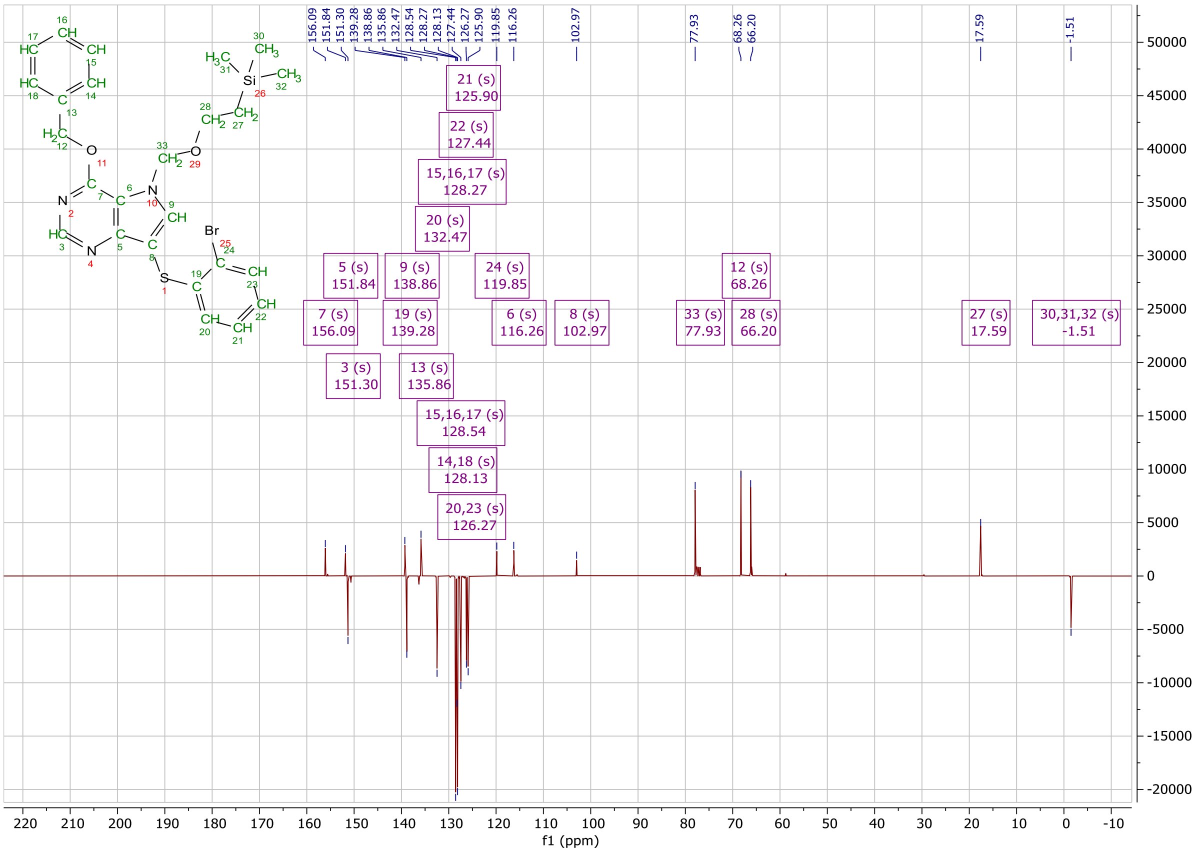

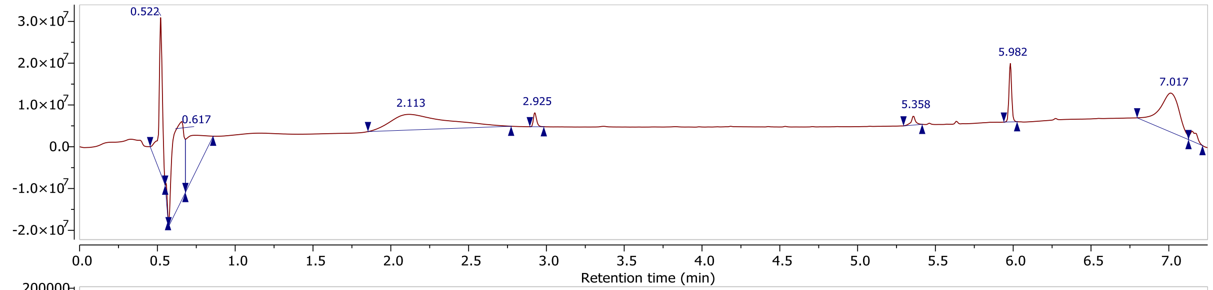

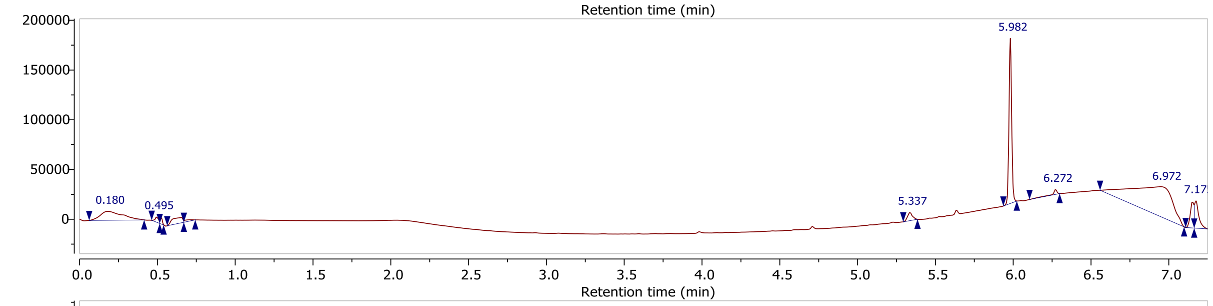

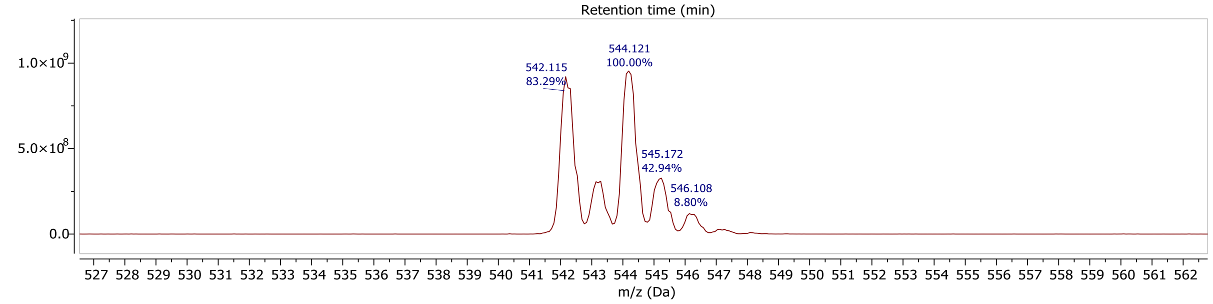

3.2.7 4-(Benzyloxy)-7-((2-hydroxy)phenylthio)-5-((2-(trimethylsilyl)ethoxy)methyl)-5*H*-pyrrolo[3,2-*d*]pyrimidine (6d)

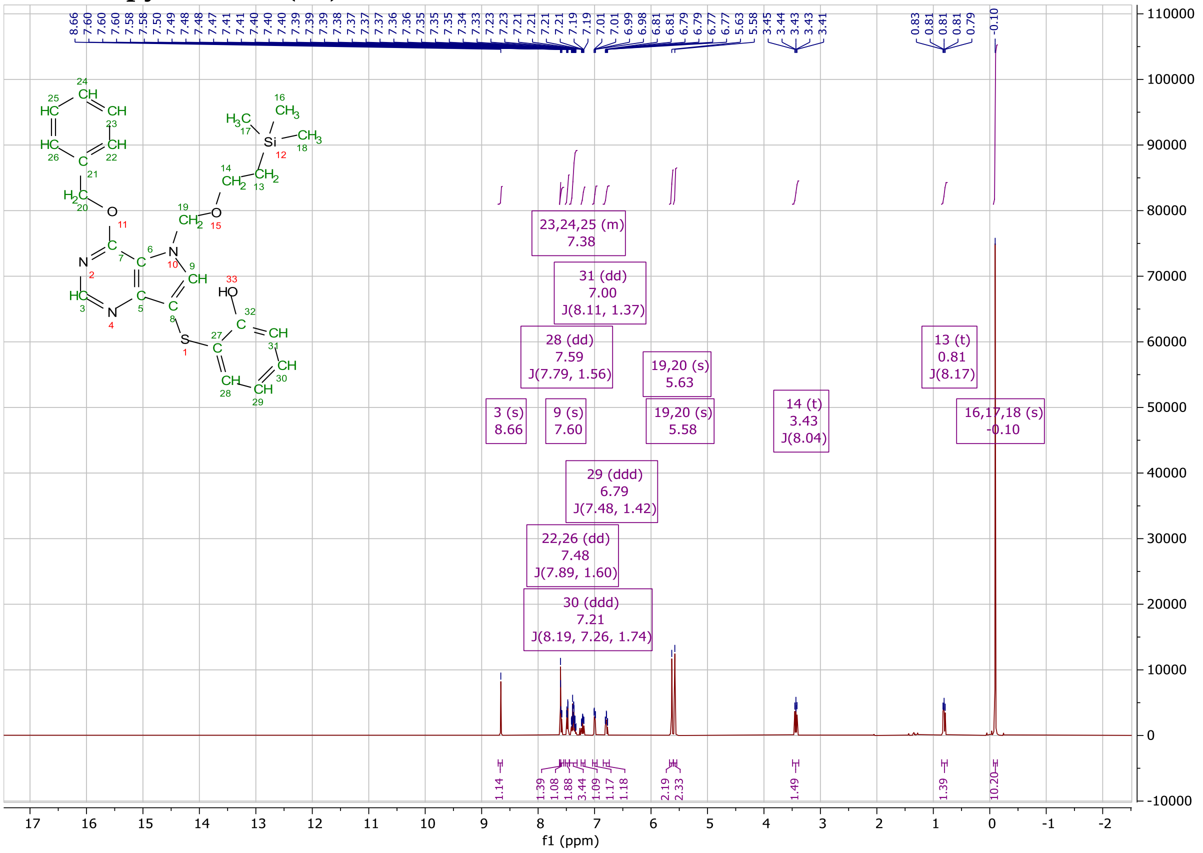

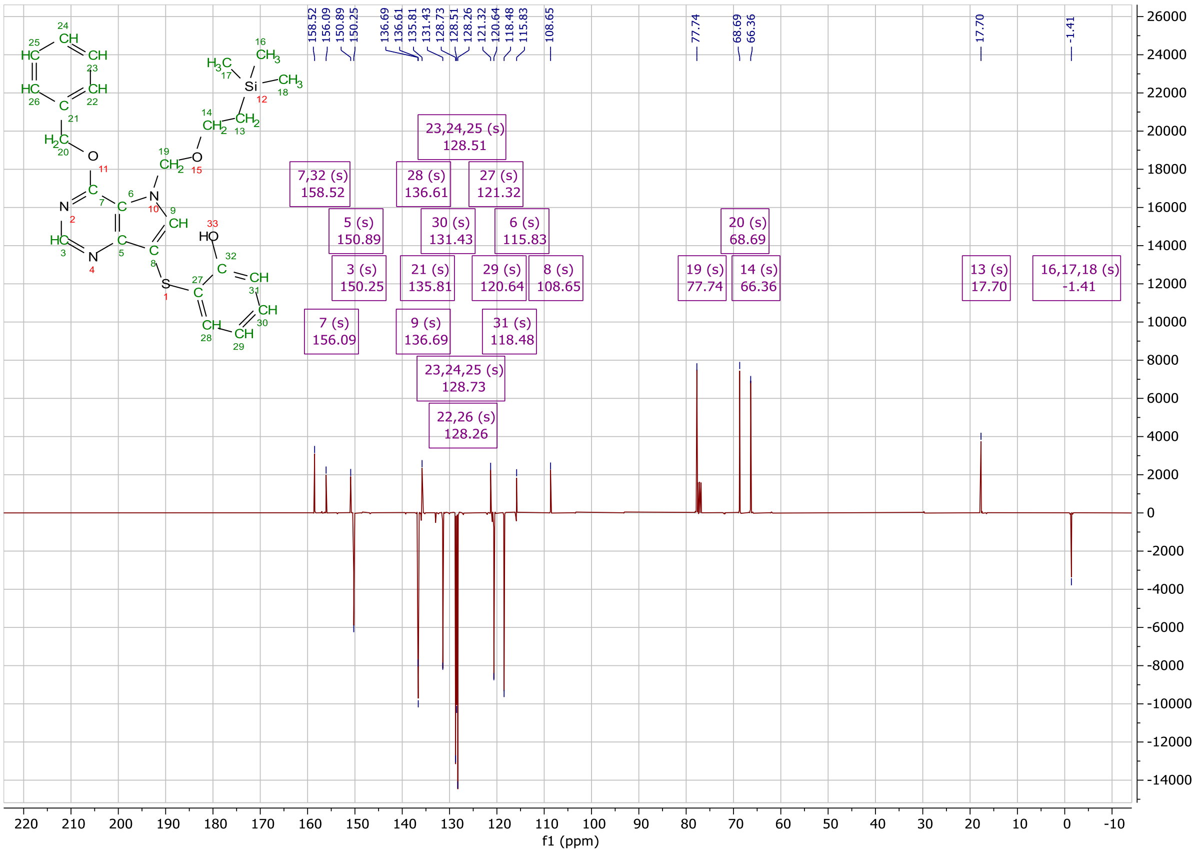

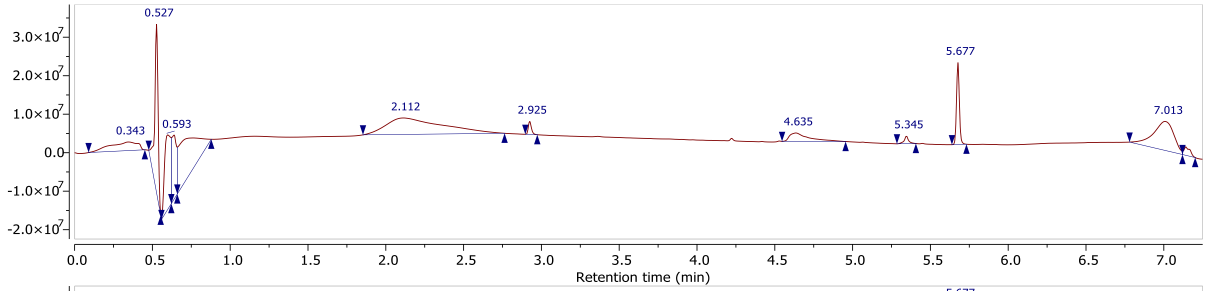

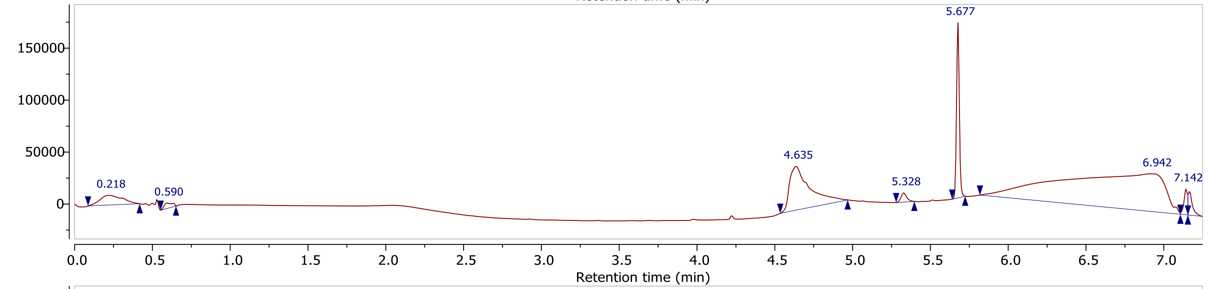

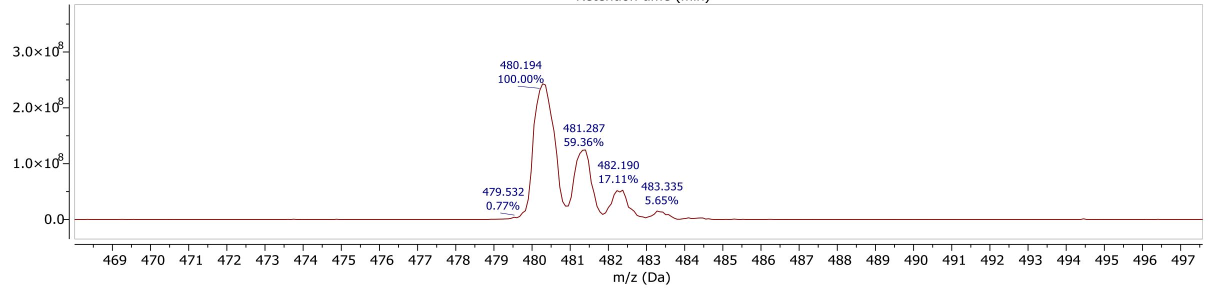

3.2.8 4-(Benzyloxy)-7-((2-hydroxymethyl)phenylthio)-5-((2-(trimethylsilyl)ethoxy)methyl)-5*H*-pyrrolo[3,2-*d*]pyrimidine (6e)

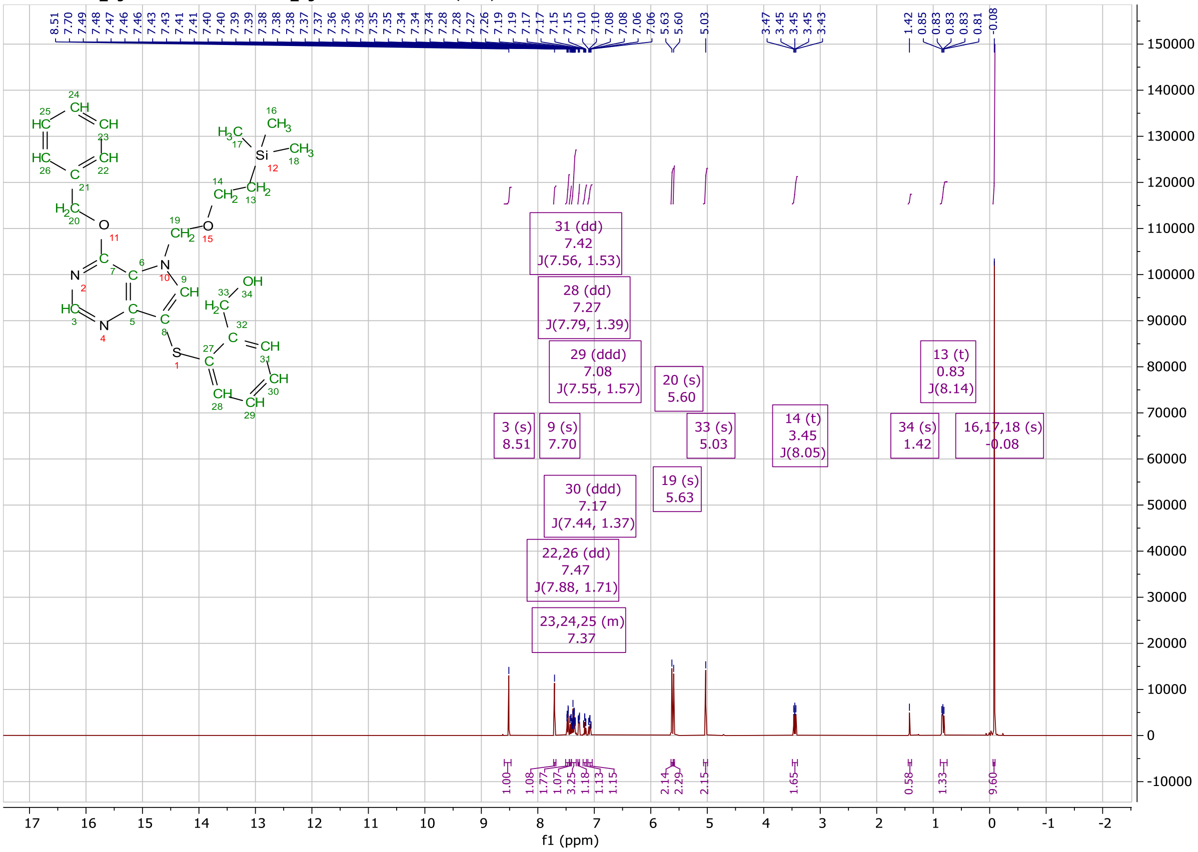

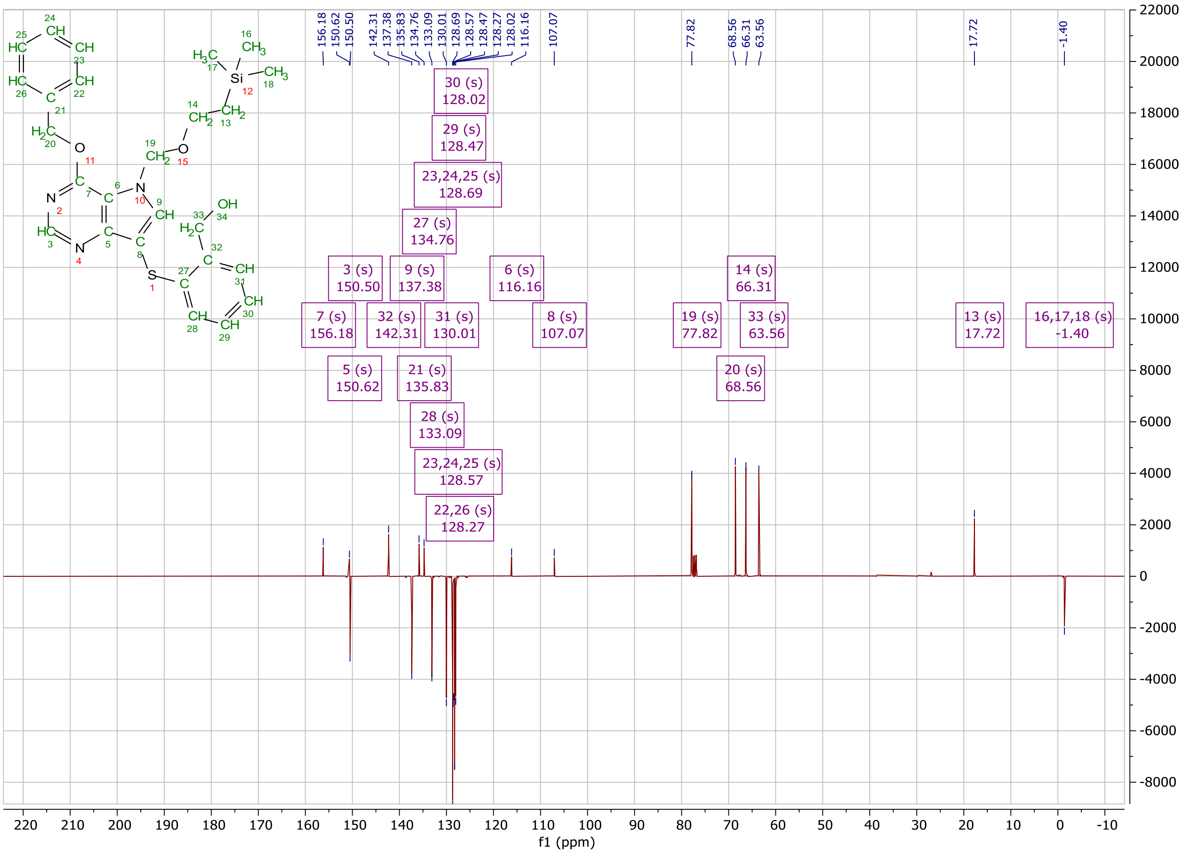

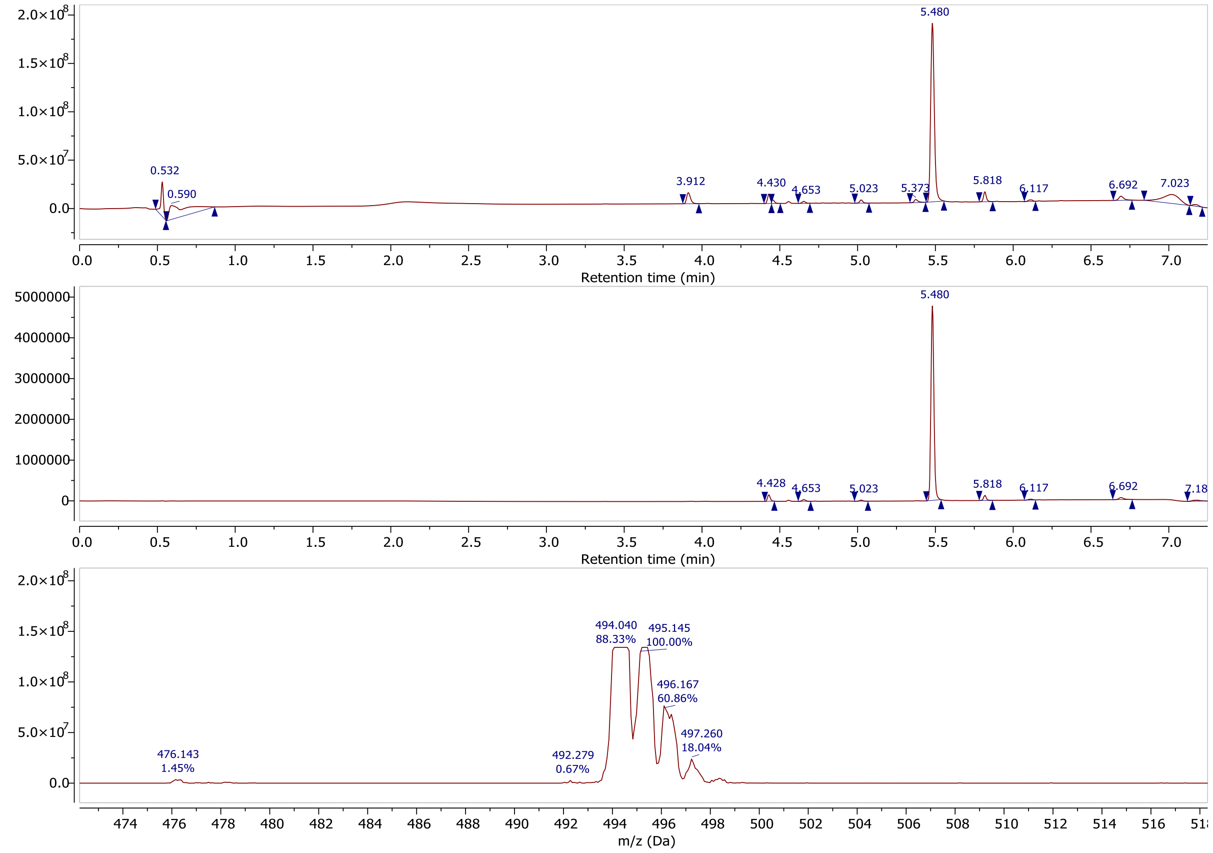

3.2.9 4-(Benzyloxy)-7-((3-bromo)phenylthio)-5-((2-(trimethylsilyl)ethoxy)methyl)-5*H*-pyrrolo[3,2-*d*]pyrimidine (6f)

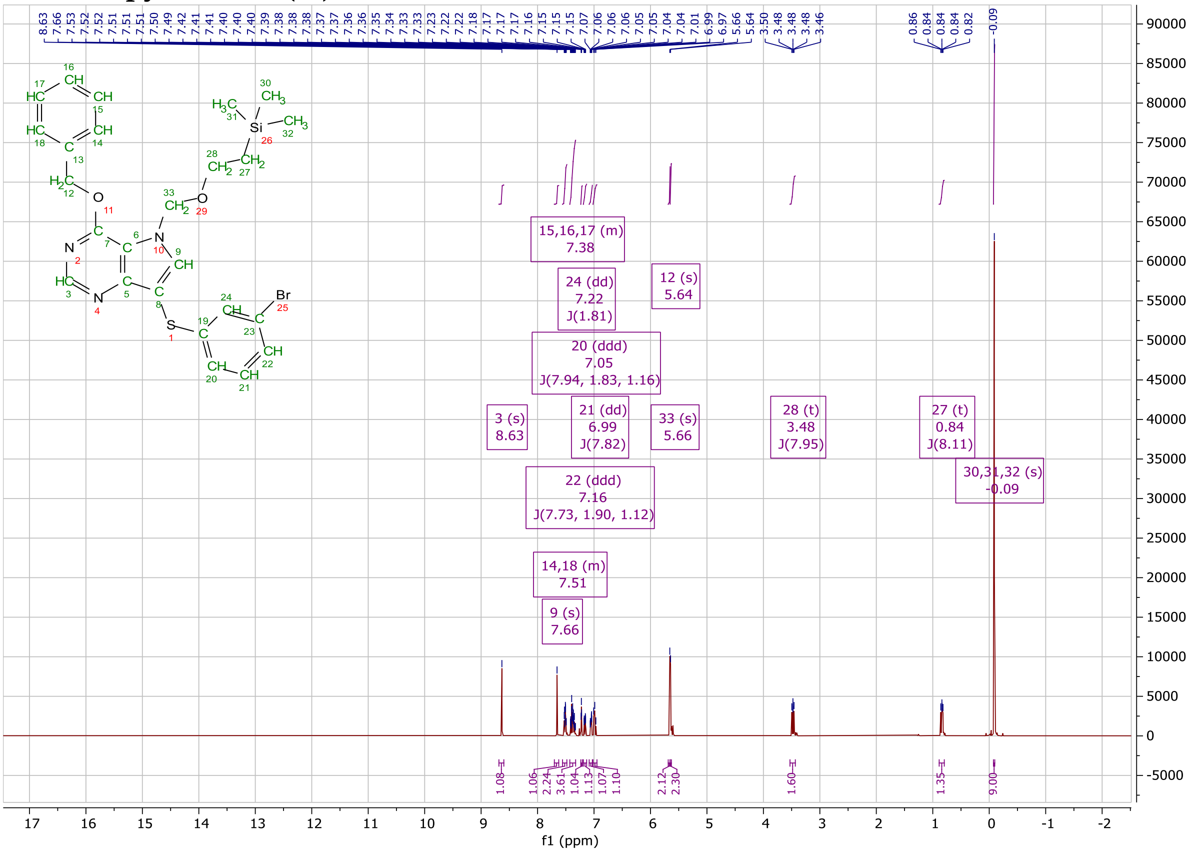

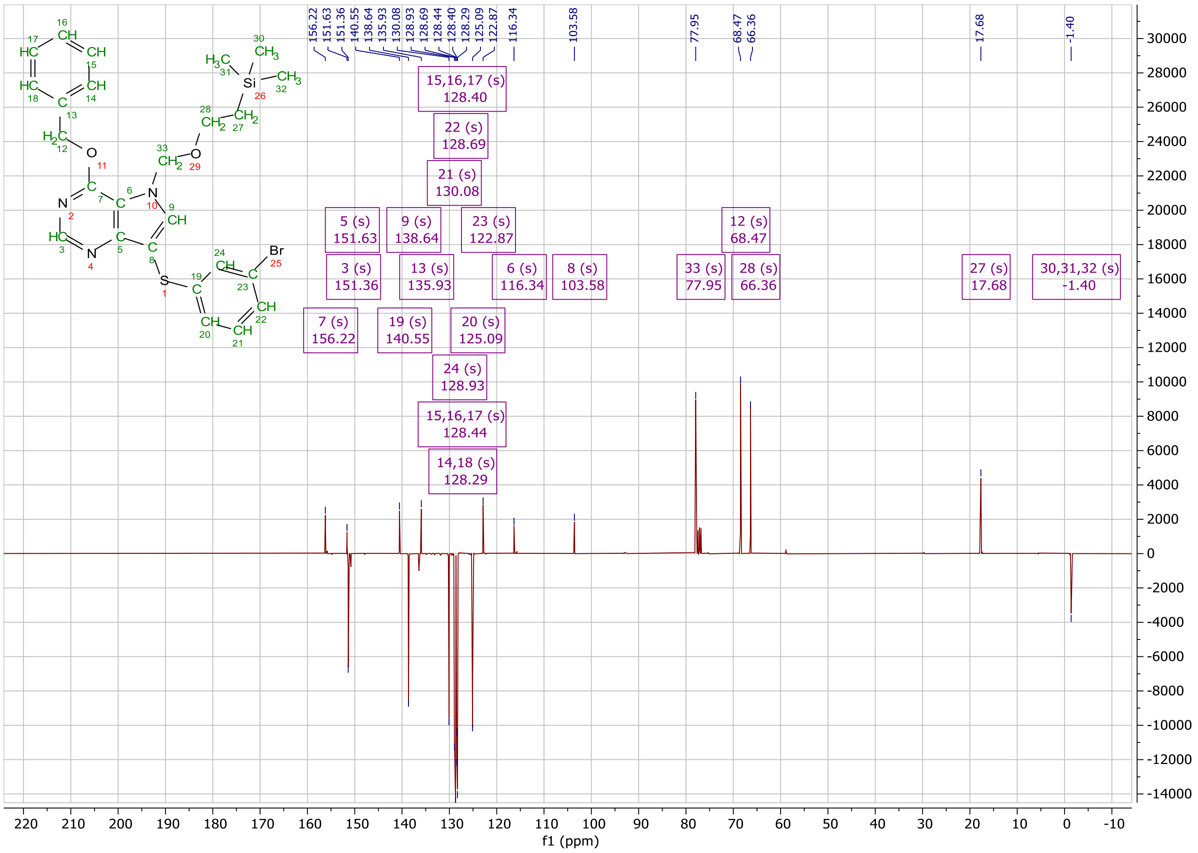

156.22
151.63
151.36
140.55
138.64
135.93
130.08
128.93
128.69
128.44
128.40
128.29
125.09
122.87
116.34
103.58
77.95
68.47
66.36
17.68
-1.40
15,16,17 (s) 128.40
22 (s) 128.69
21 (s) 130.08
5 (s) 151.63
9 (s) 138.64
23 (s) 122.87
12 (s) 68.47
3 (s) 151.36
13 (s) 135.93
6 (s) 116.34
8 (s) 103.58
33 (s) 77.95
28 (s) 66.36
27 (s) 17.68
30,31,32 (s) -1.40
7 (s) 156.22
19 (s) 140.55
20 (s) 125.09
24 (s) 128.93
15,16,17 (s) 128.44
14,18 (s) 128.29
Si
Br
f1 (ppm)

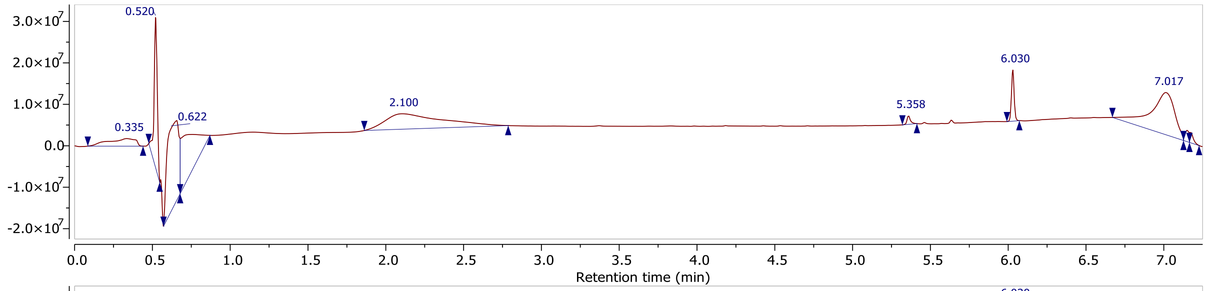

0.520
0.335
0.622
2.100
5.358
6.030
7.017
Retention time (min)

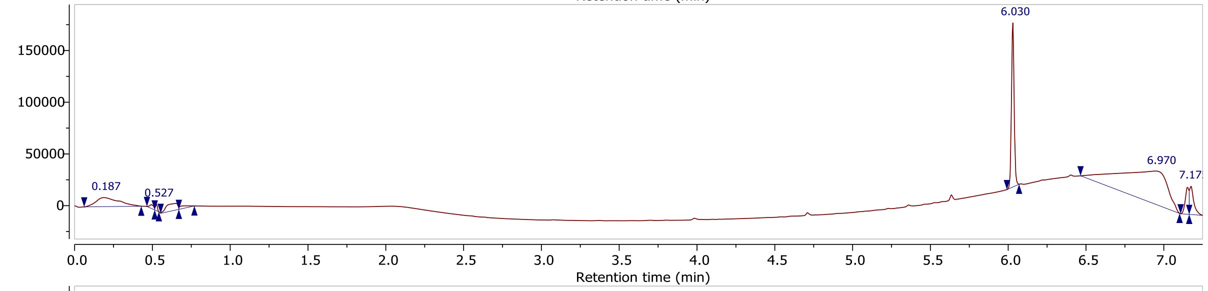

6.030
0.187
0.527
6.970
Retention time (min)

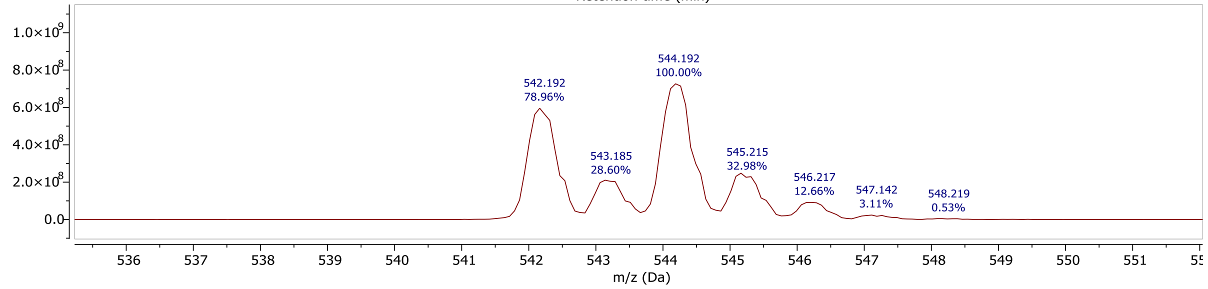

542.192
78.96%
543.185
28.60%
544.192
100.00%
545.215
32.98%
546.217
12.66%
547.142
3.11%
548.219
0.53%
m/z (Da)

3.2.10 7-((Phenyl)thio)-3,5-dihydro-4*H*-pyrrolo[3,2-*d*]pyrimidin-4-one (7a)

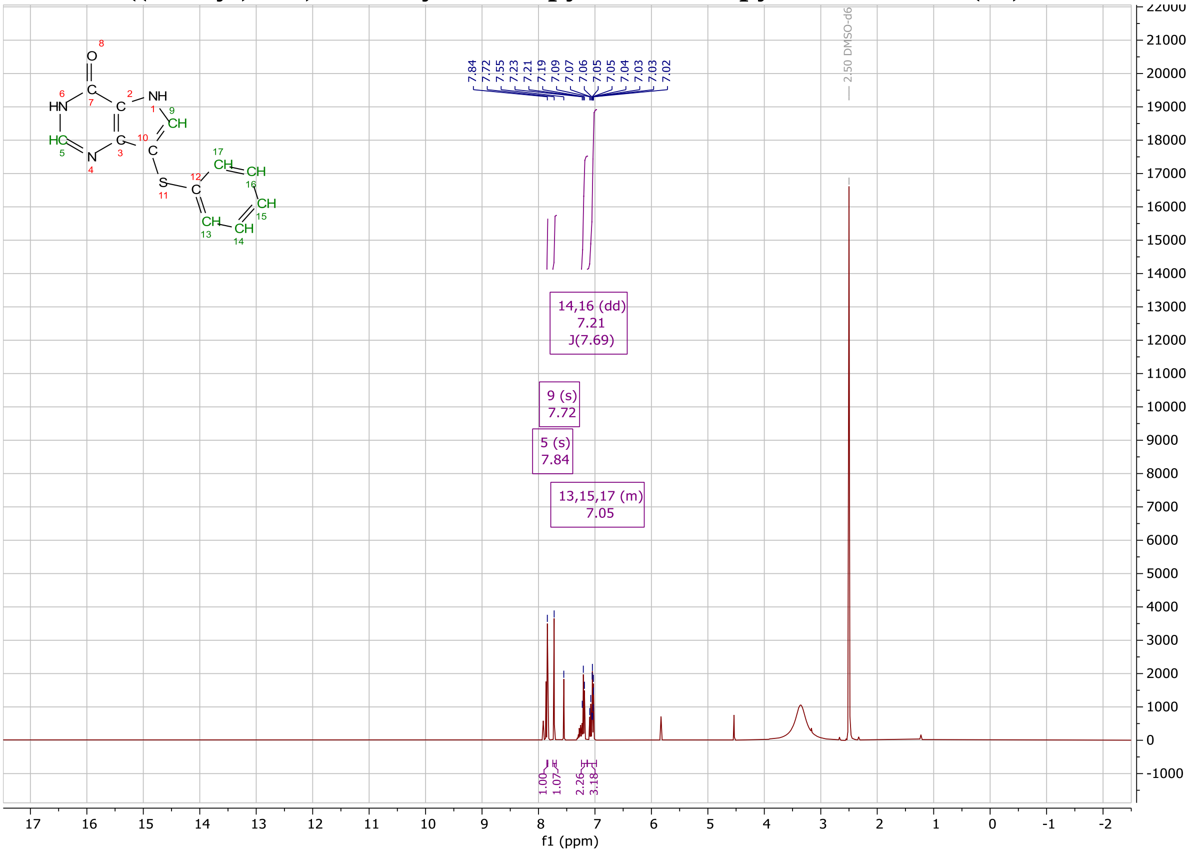

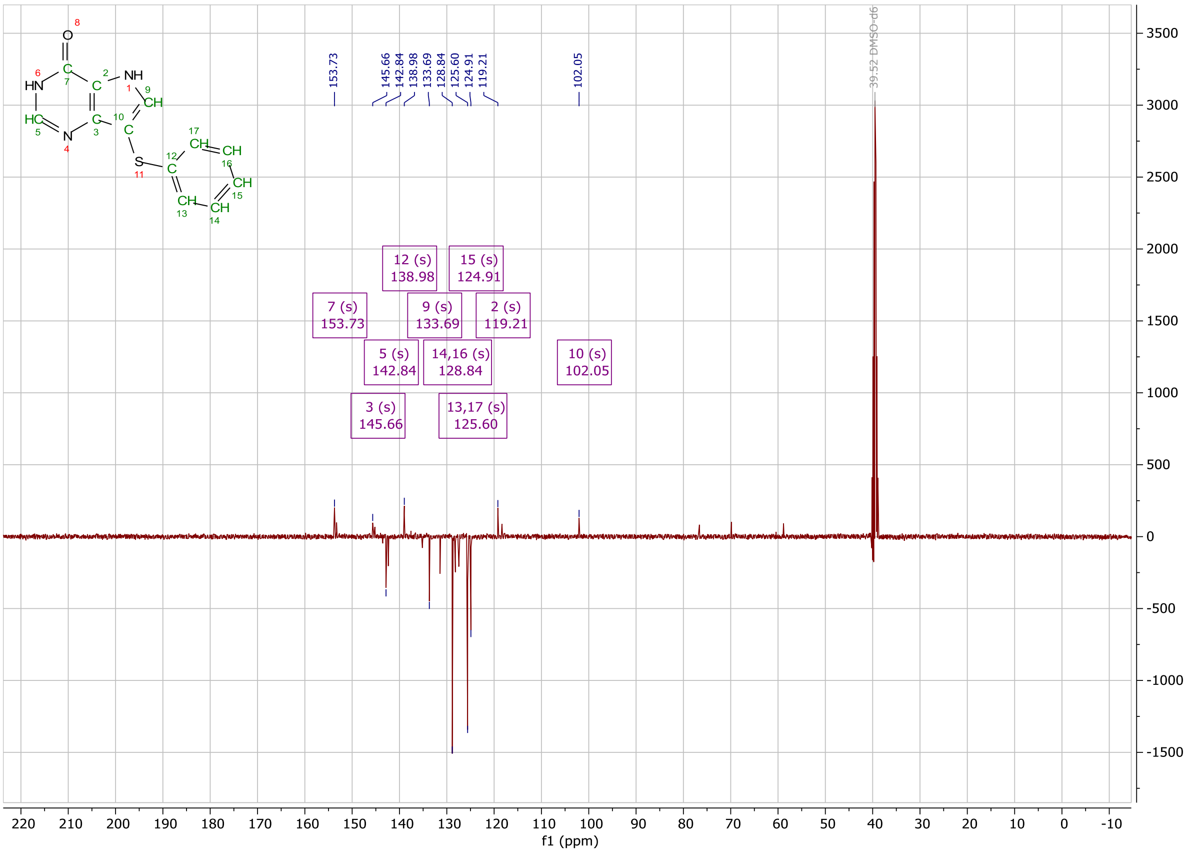

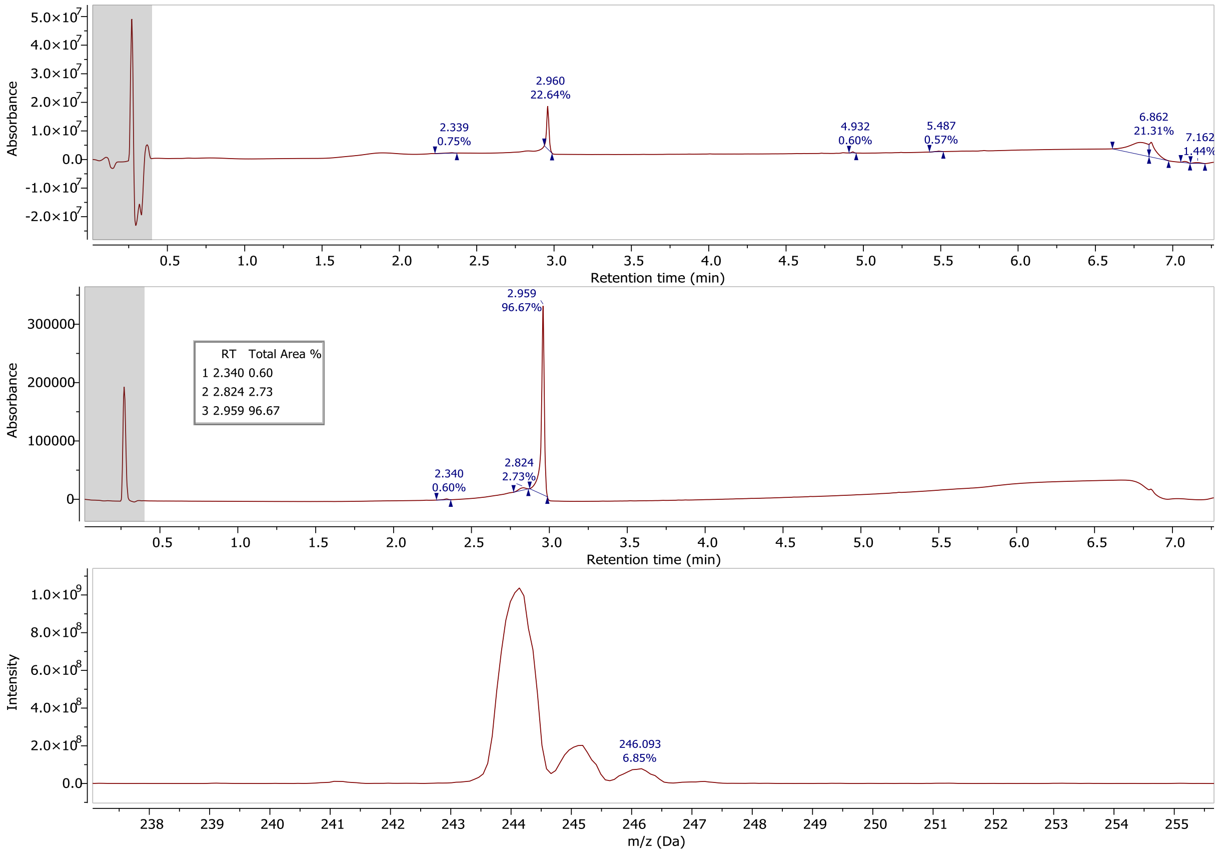

3.2.11 7-(((2-Methyl)phenyl)thio)-3,5-dihydro-4*H*-pyrrolo[3,2-*d*]pyrimidin-4-one (7b)

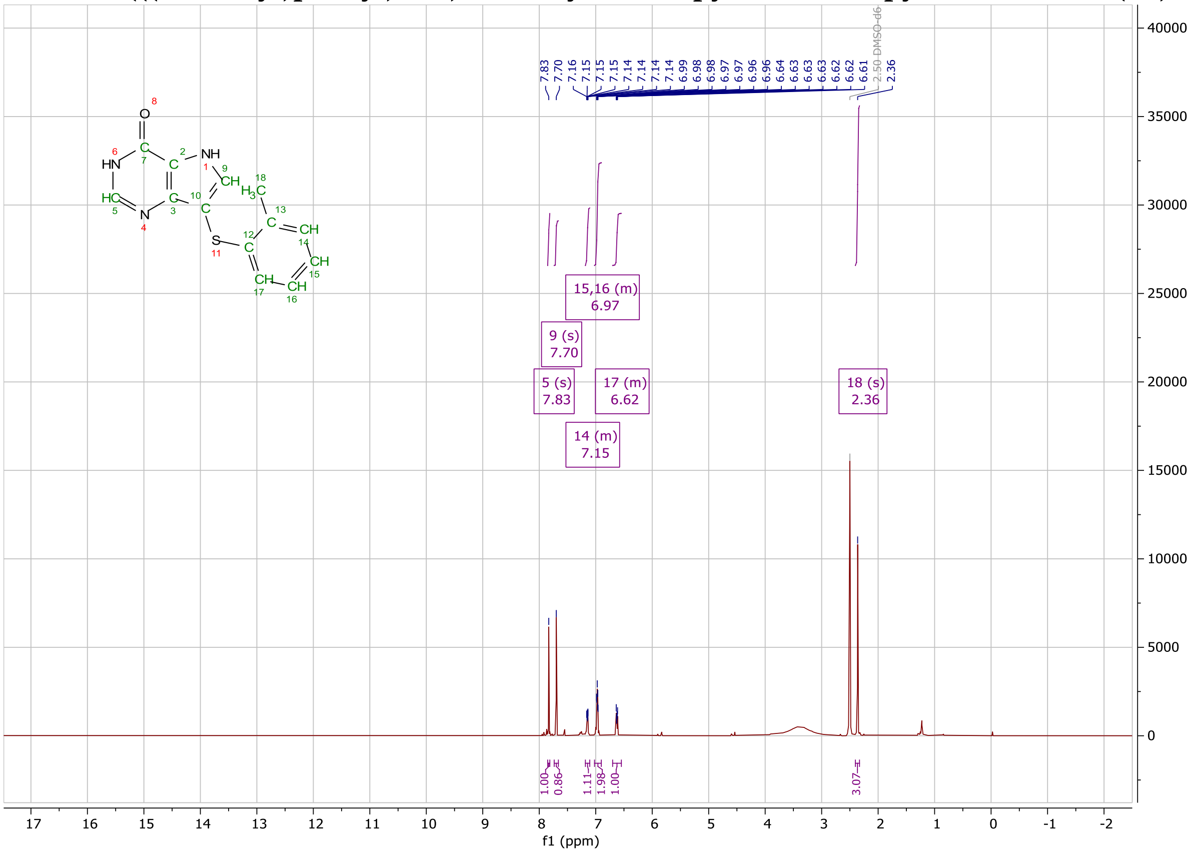

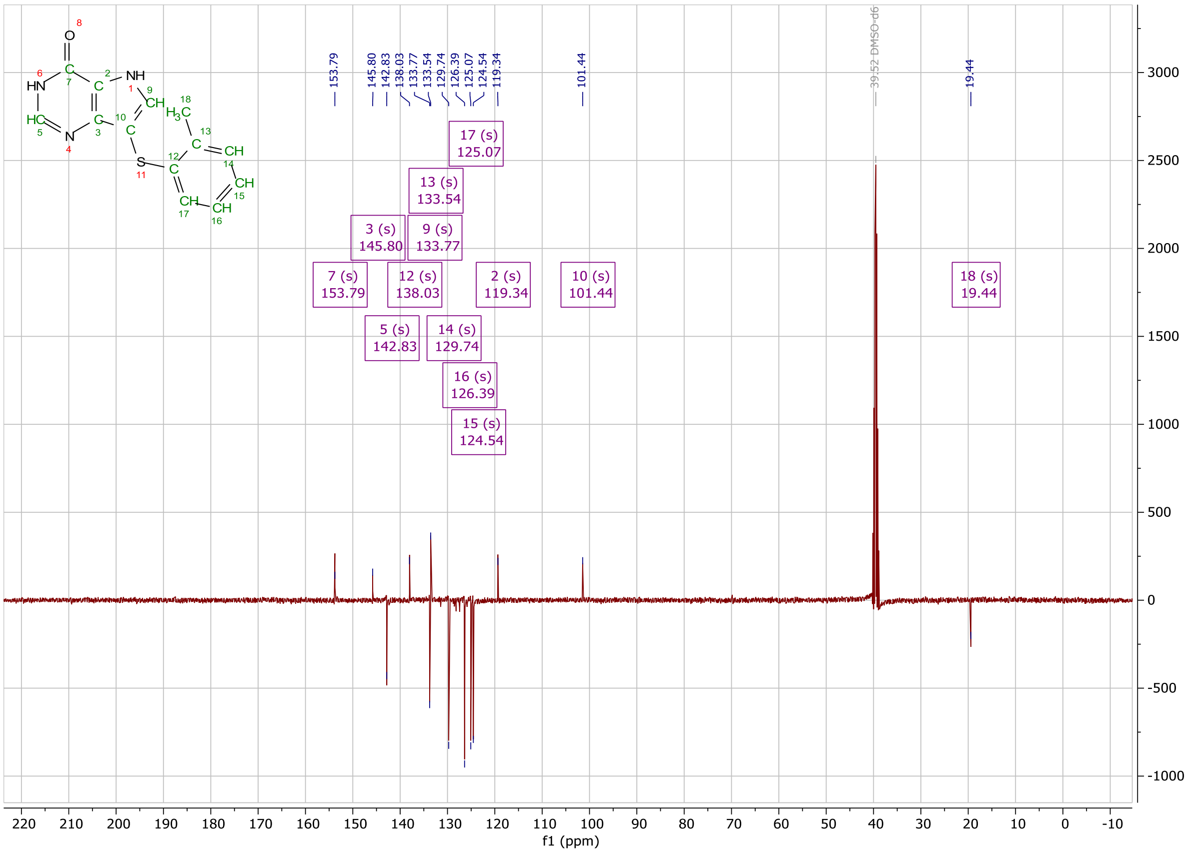
153.79
145.80
142.83
138.03
133.77
133.54
129.74
126.39
125.07
124.54
119.34
101.44
39.52 DMSO-d6
19.44
17 (s) 125.07
13 (s) 133.54
3 (s) 145.80
9 (s) 133.77
7 (s) 153.79
12 (s) 138.03
2 (s) 119.34
10 (s) 101.44
18 (s) 19.44
5 (s) 142.83
14 (s) 129.74
16 (s) 126.39
15 (s) 124.54
f1 (ppm)

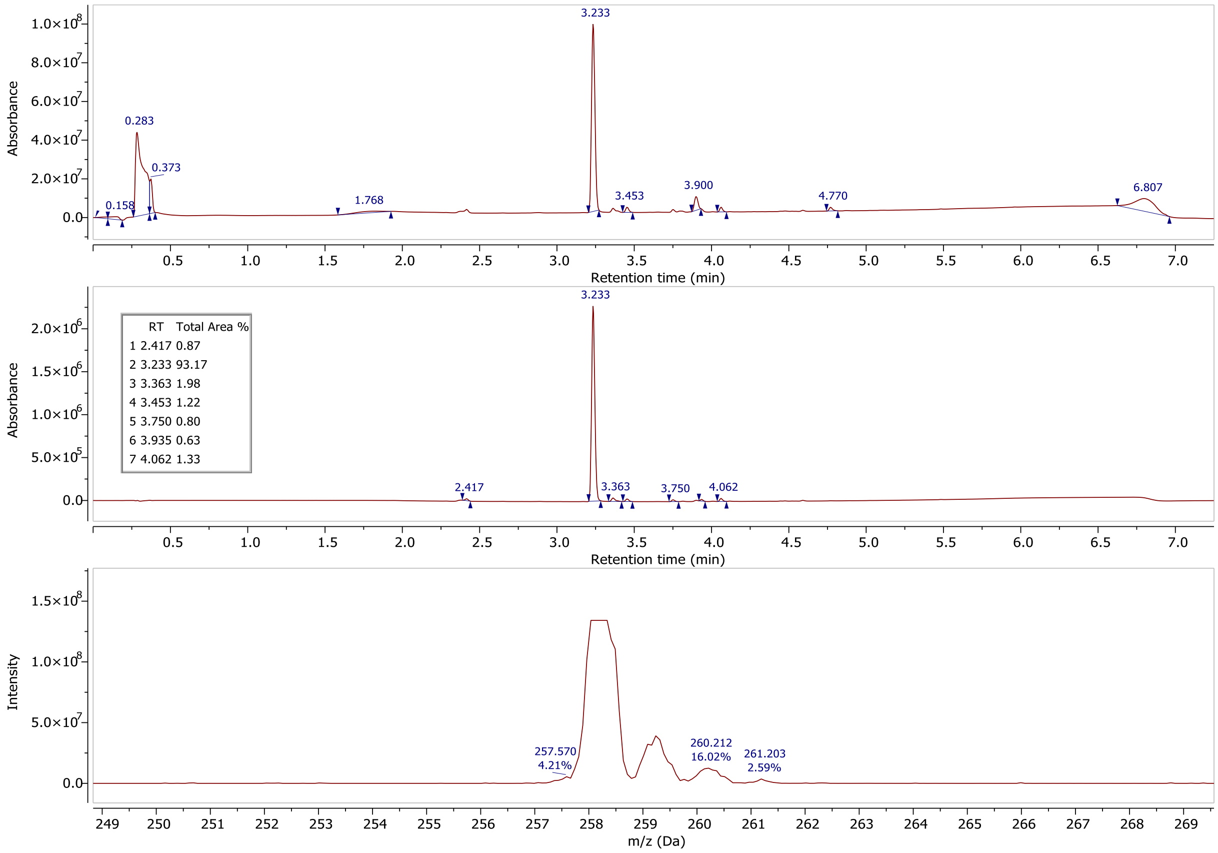
Absorbance
Retention time (min)
3.233
0.283
0.373
0.158
1.768
3.453
3.900
4.770
6.807
RT Total Area %
1 2.417 0.87
2 3.233 93.17
3 3.363 1.98
4 3.453 1.22
5 3.750 0.80
6 3.935 0.63
7 4.062 1.33
2.417
3.363
3.750
4.062
Intensity
m/z (Da)
257.570
4.21%
260.212
16.02%
261.203
2.59%

3.2.12 7-(((2-Bromo)phenyl)thio)-3,5-dihydro-4*H*-pyrrolo[3,2-*d*]pyrimidin-4-one (7c)

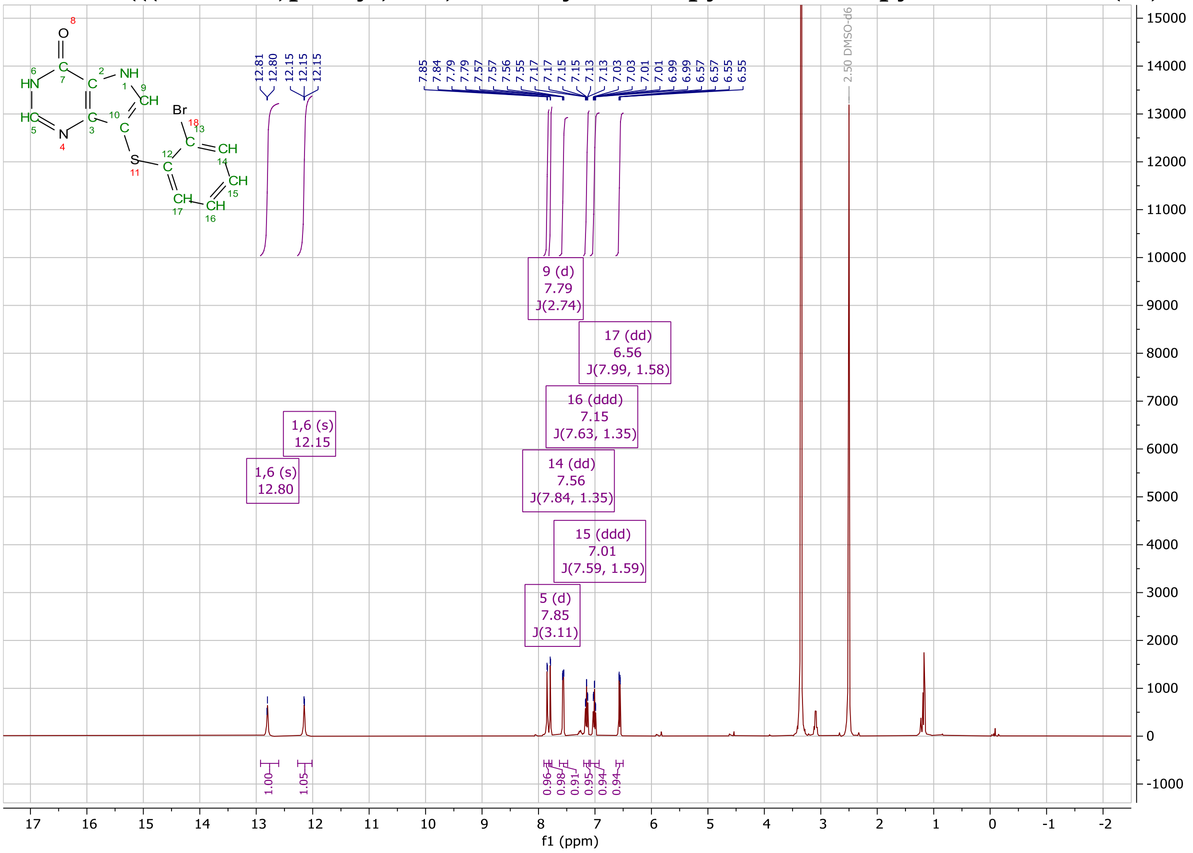

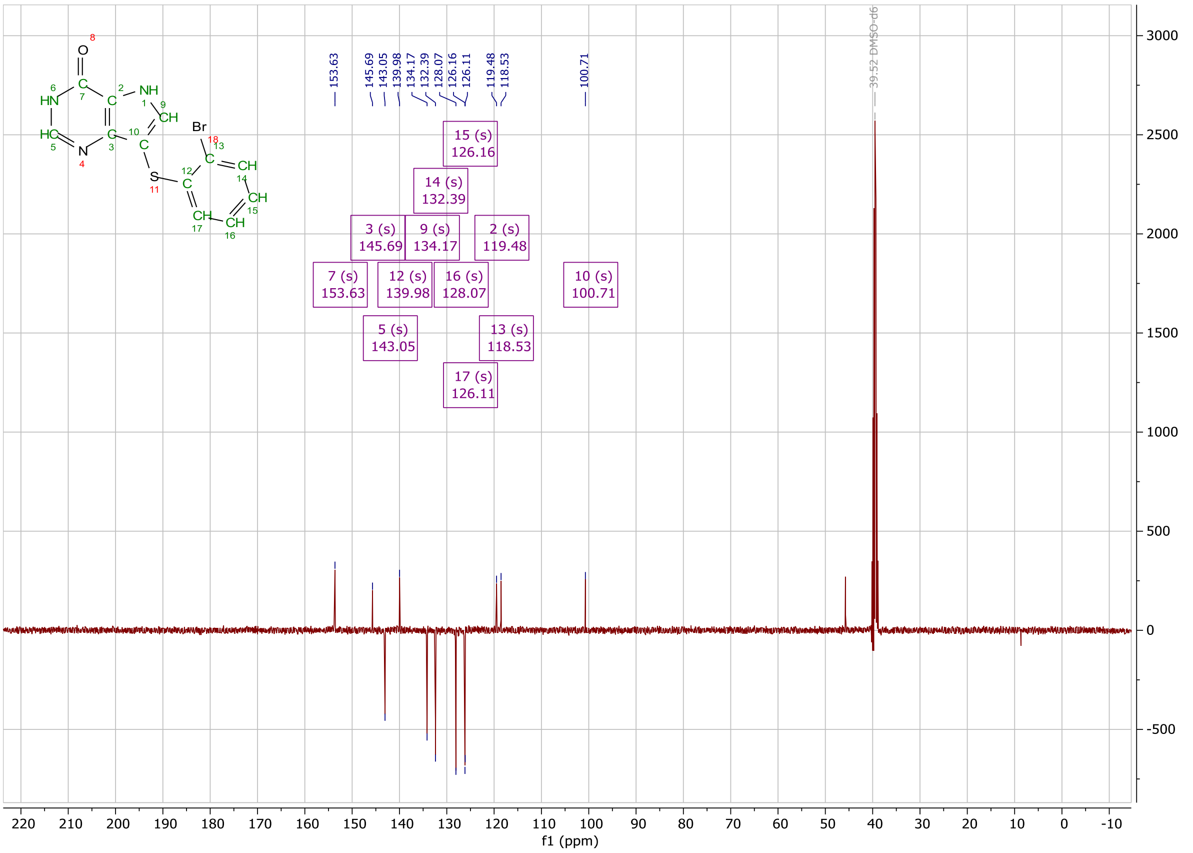

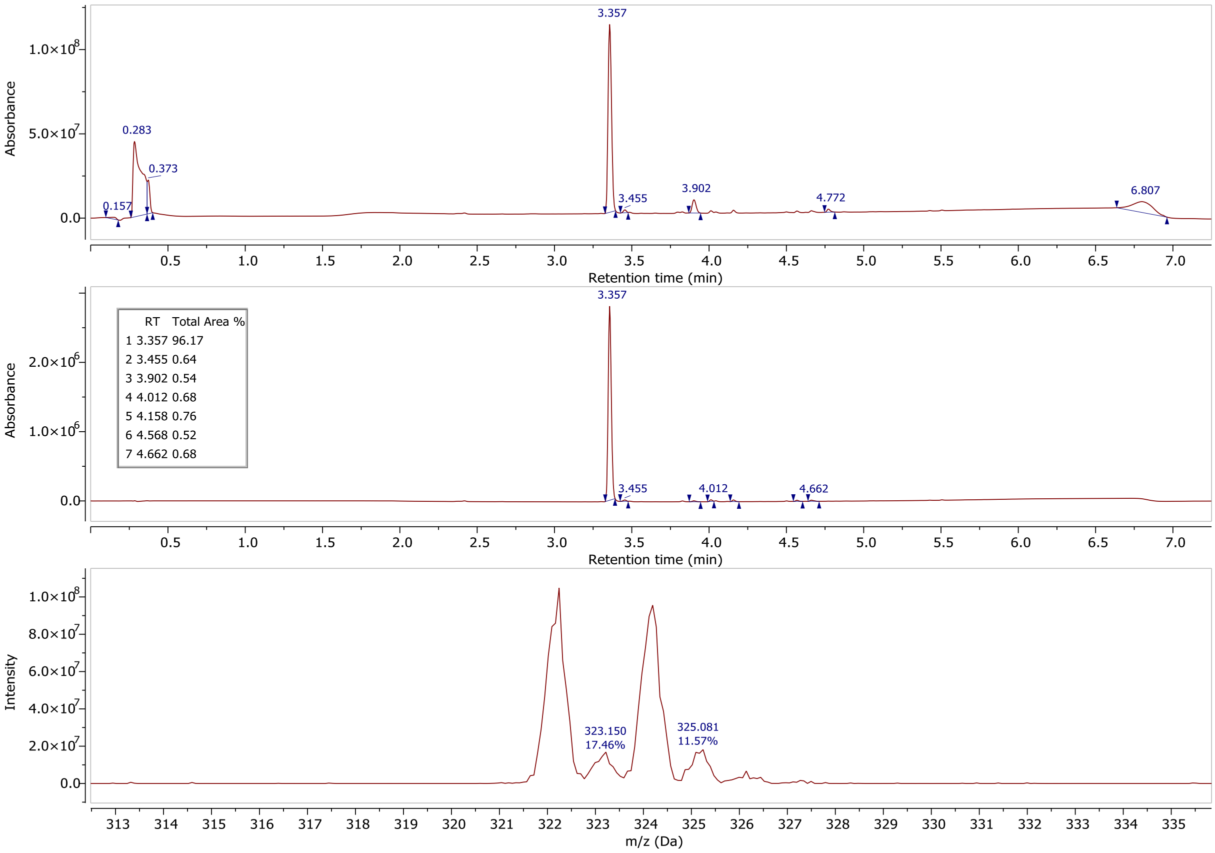

3.2.13 7-(((2-Hydroxy)phenyl)thio)-3,5-dihydro-4*H*-pyrrolo[3,2-*d*]pyrimidin-4-one (7d)

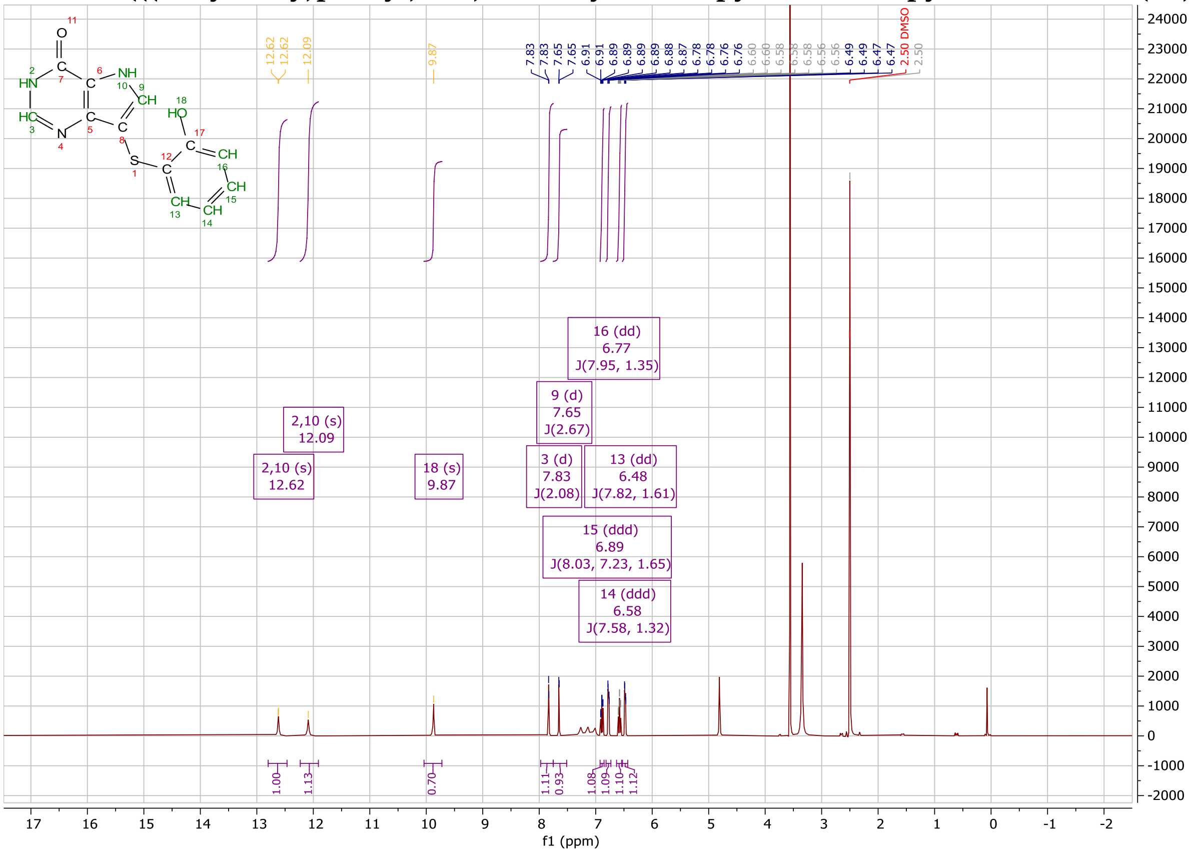

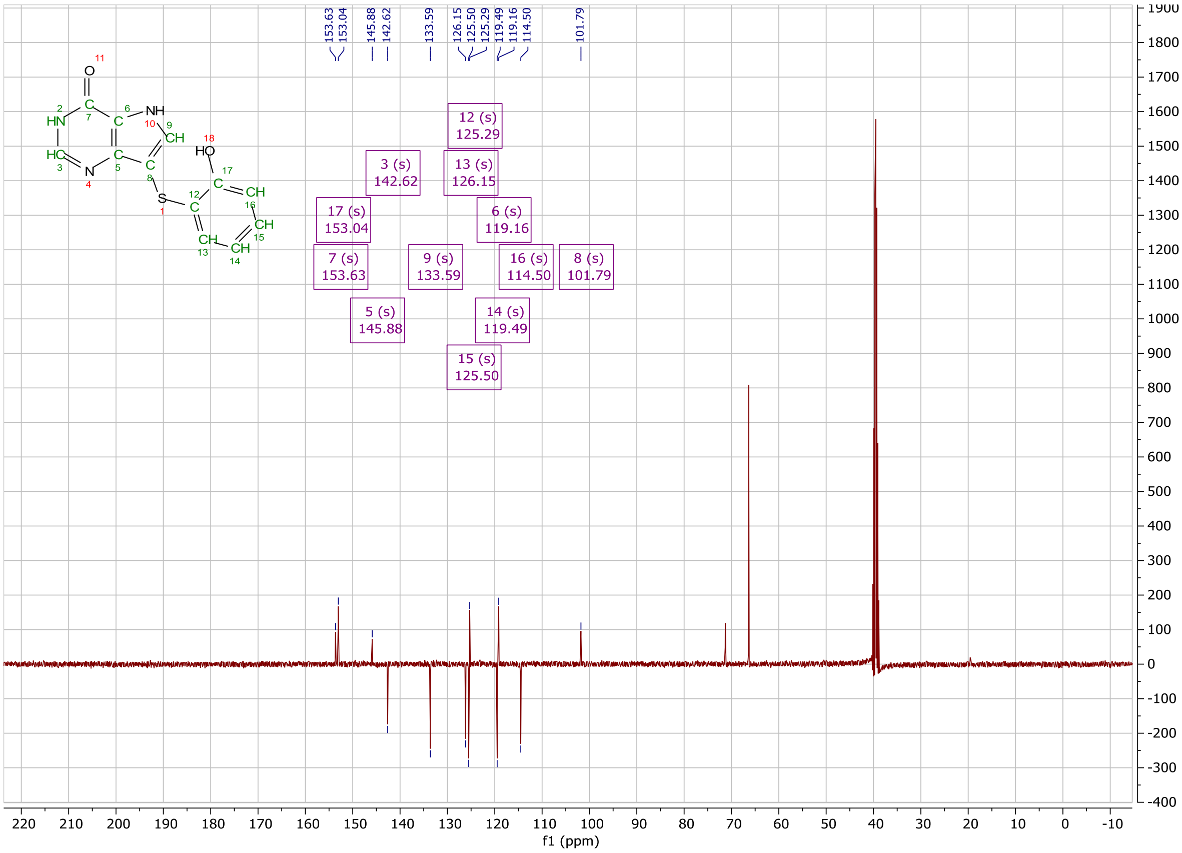
153.63
153.04
145.88
142.62
133.59
126.15
125.50
125.29
119.49
119.16
114.50
101.79
12 (s) 125.29
3 (s) 142.62
13 (s) 126.15
17 (s) 153.04
6 (s) 119.16
7 (s) 153.63
9 (s) 133.59
16 (s) 114.50
8 (s) 101.79
5 (s) 145.88
14 (s) 119.49
15 (s) 125.50
f1 (ppm)

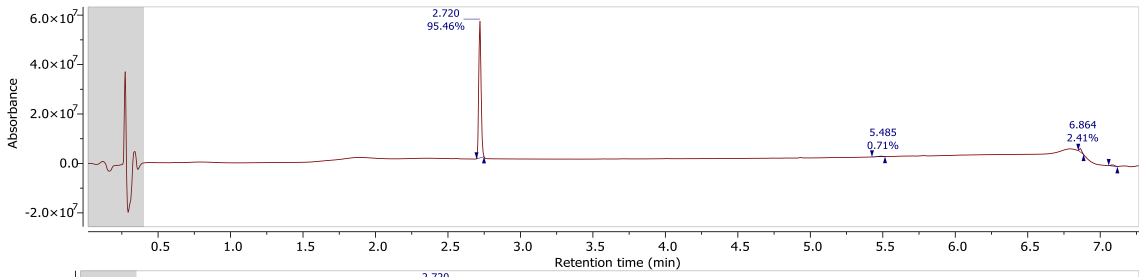
2.720
95.46%
5.485
0.71%
6.864
2.41%
Absorbance
Retention time (min)

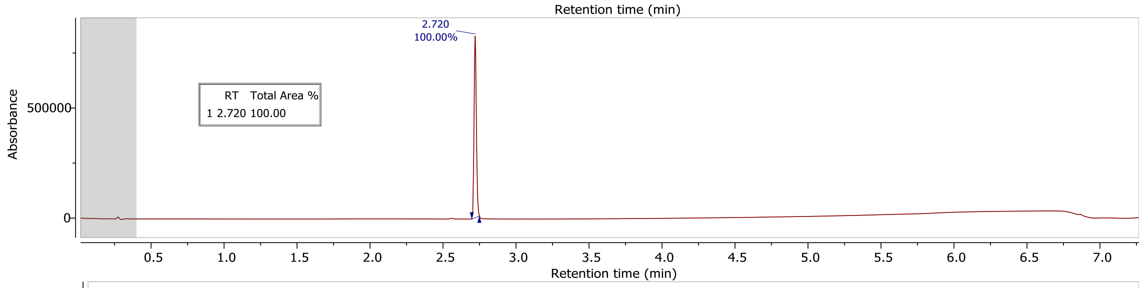
2.720
100.00%
RT Total Area %
1 2.720 100.00
Absorbance
Retention time (min)

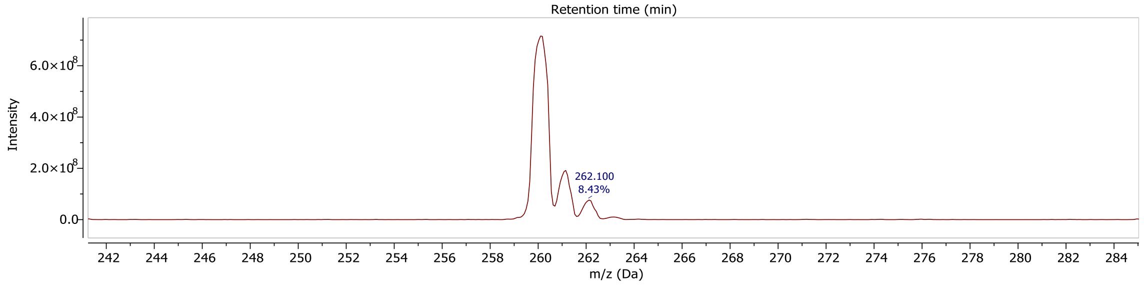
262.100
8.43%
Intensity
m/z (Da)

3.2.14 7-(((2-Hydroxymethyl)phenyl)thio)-3,5-dihydro-4*H*-pyrrolo[3,2-*d*]pyrimidin-4-one (7e)

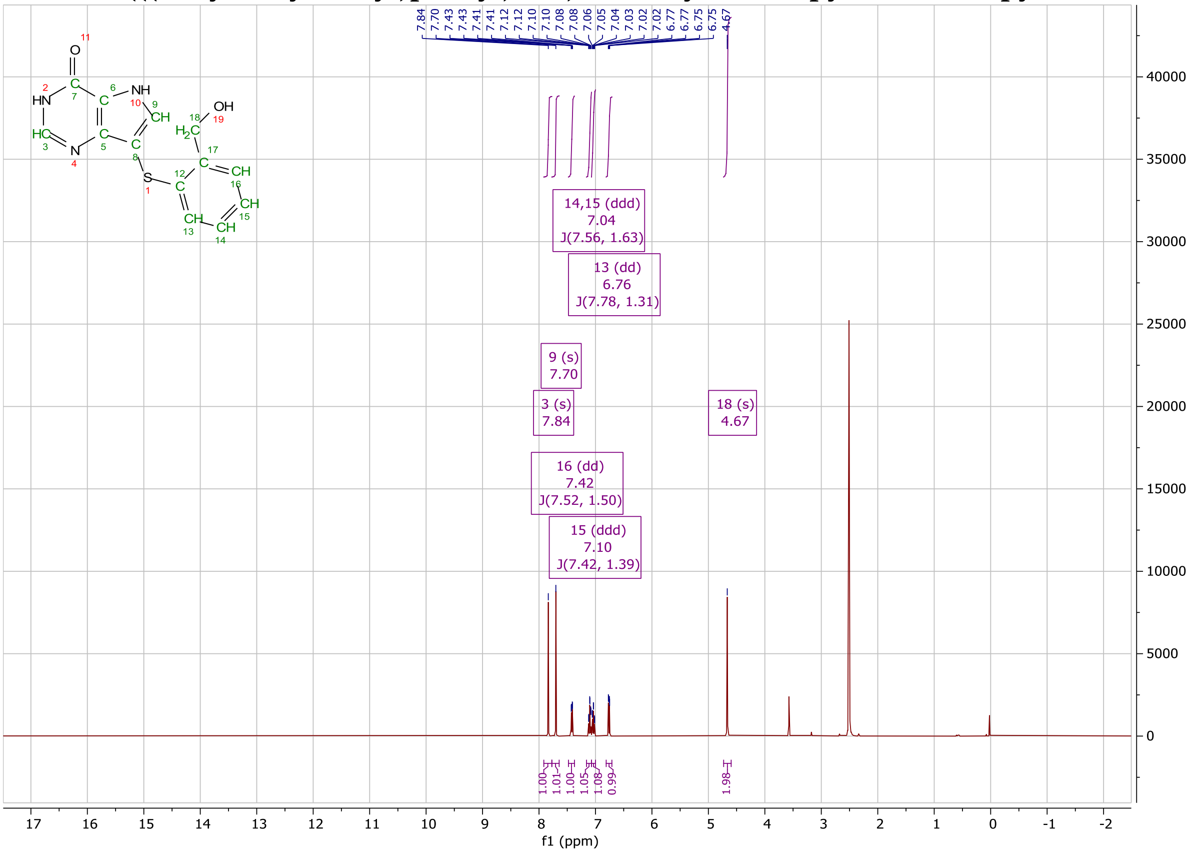

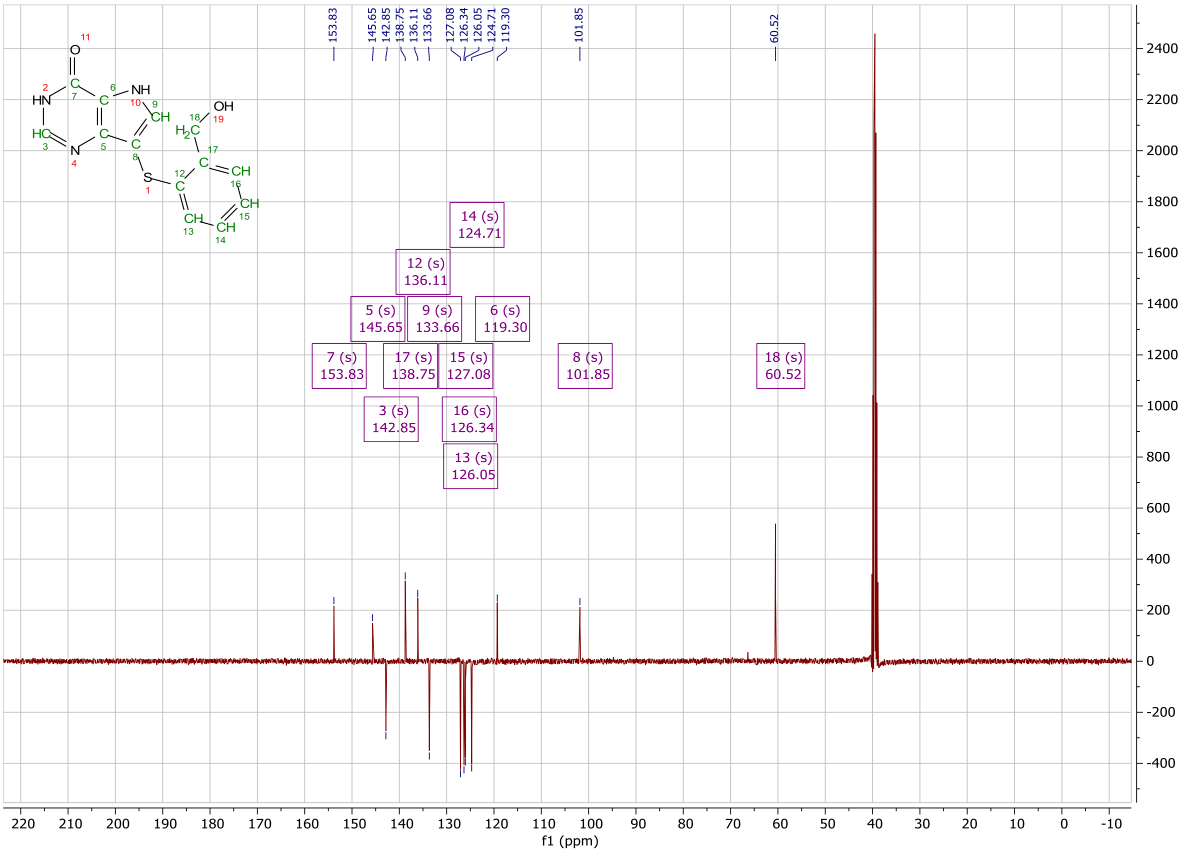

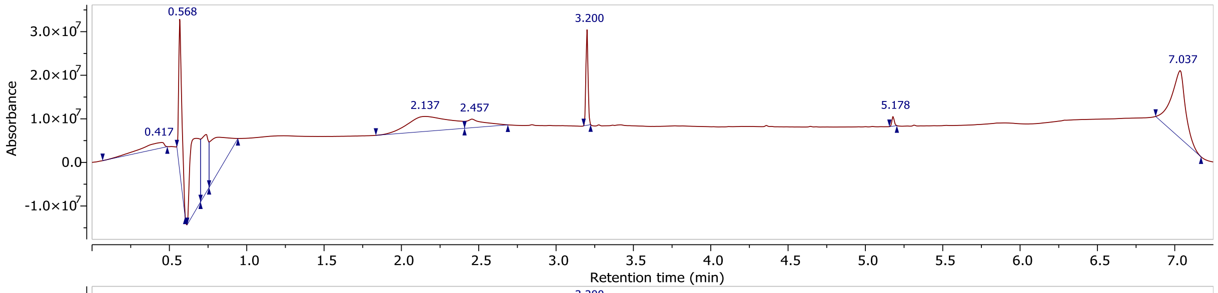

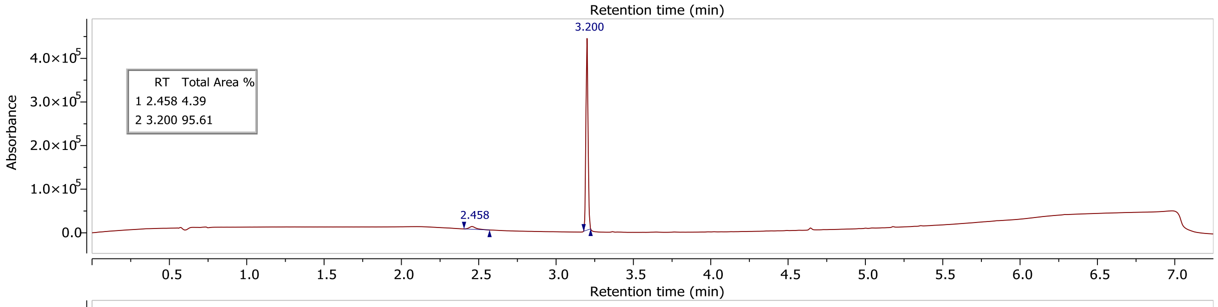

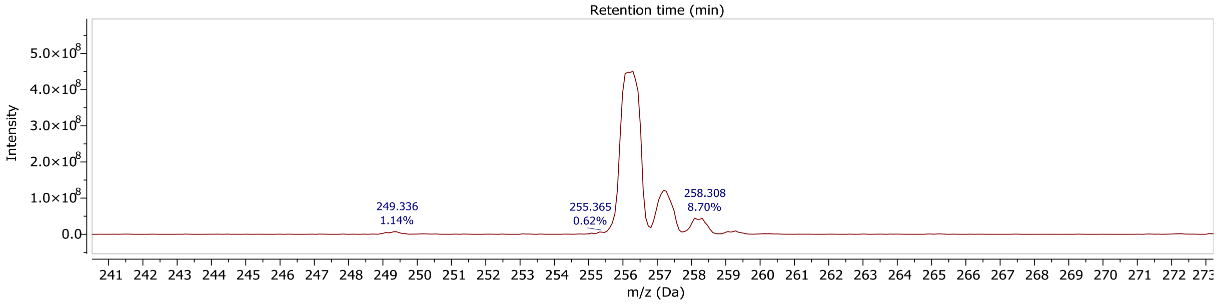

3.2.15 7-(((3-Bromo)phenyl)thio)-3,5-dihydro-4*H*-pyrrolo[3,2-*d*]pyrimidin-4-one (7f)

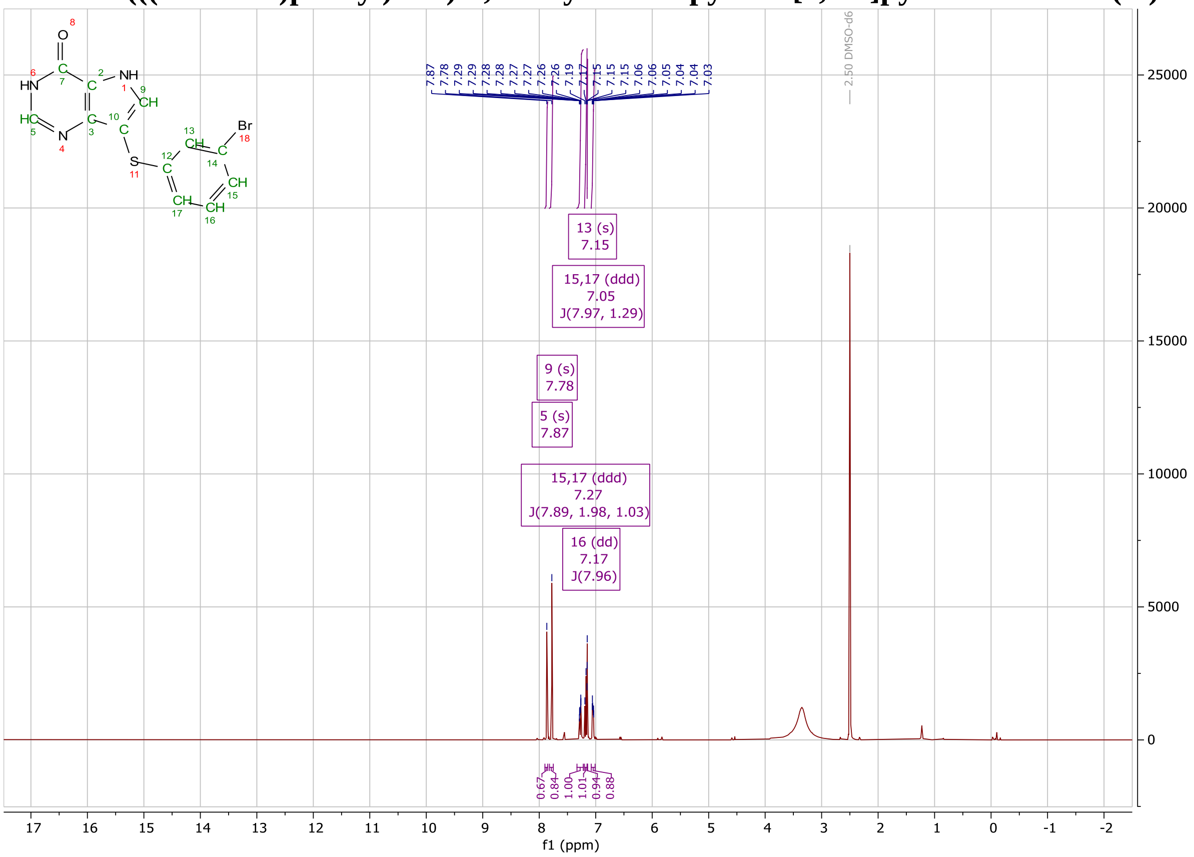

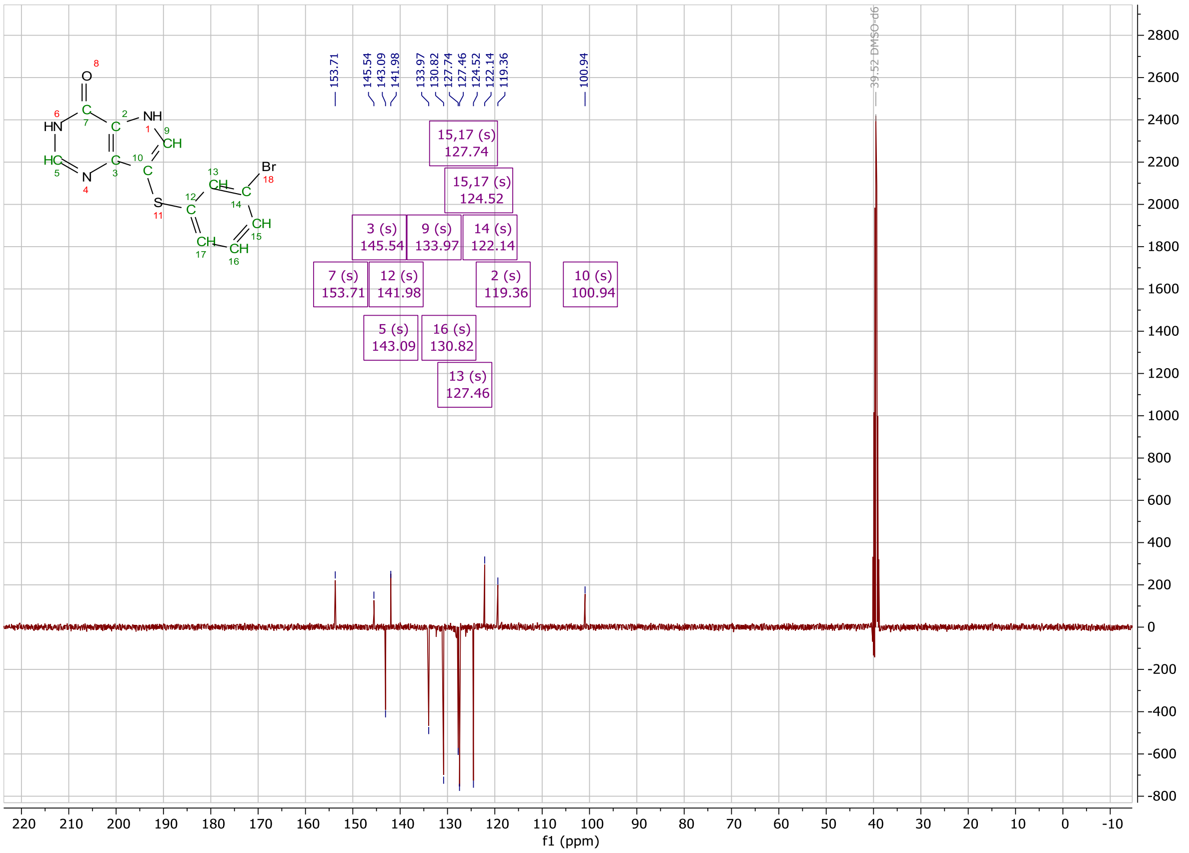

153.71
145.54
143.09
141.98
133.97
130.82
127.74
127.46
124.52
122.14
119.36
100.94
39.52 DMSO-d6
15,17 (s) 127.74
15,17 (s) 124.52
3 (s) 145.54
9 (s) 133.97
14 (s) 122.14
7 (s) 153.71
12 (s) 141.98
2 (s) 119.36
10 (s) 100.94
5 (s) 143.09
16 (s) 130.82
13 (s) 127.46
f1 (ppm)

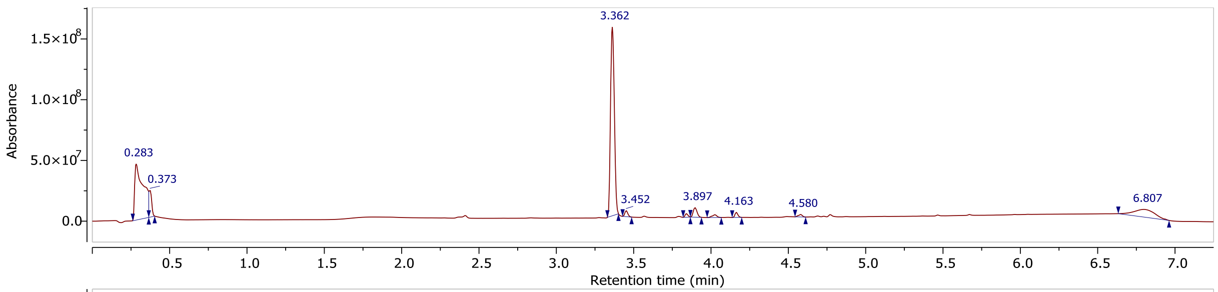

Absorbance
Retention time (min)
3.362
0.283
0.373
3.452
3.897
4.163
4.580
6.807

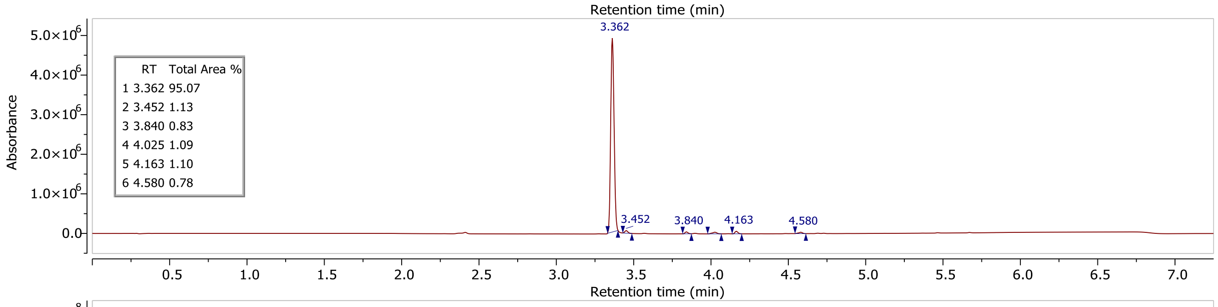

Absorbance
Retention time (min)
3.362
RT Total Area %
1 3.362 95.07
2 3.452 1.13
3 3.840 0.83
4 4.025 1.09
5 4.163 1.10
6 4.580 0.78
3.452
3.840
4.163
4.580

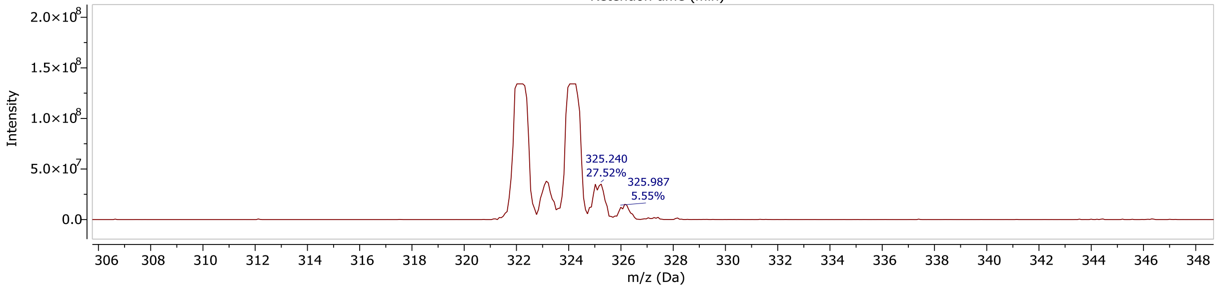

Intensity
m/z (Da)
325.240
27.52%
325.987
5.55%

3.2.16 7-(Acetylthio)-4-(benzyloxy)-5-((2-(trimethylsilyl)ethoxy)methyl)- 5*H*-pyrrolo[3,2-*d*]pyrimidine (8)

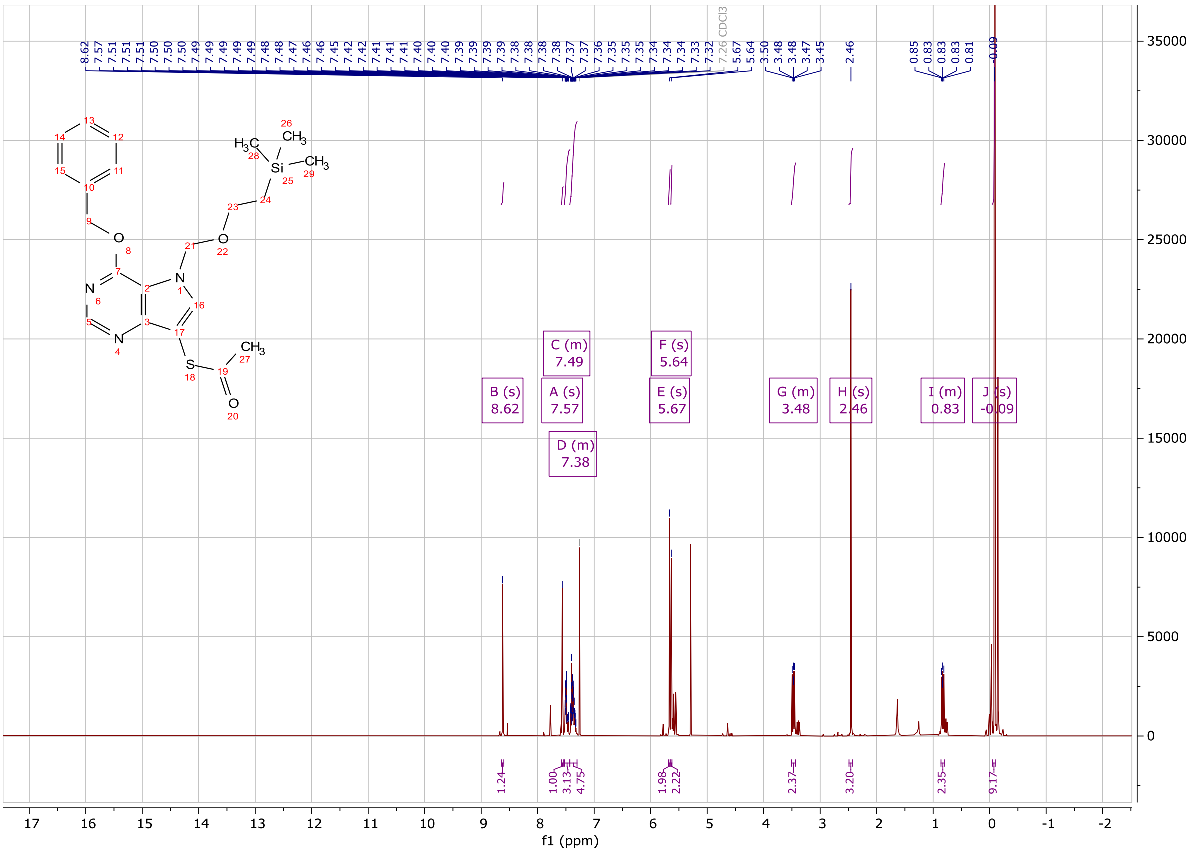

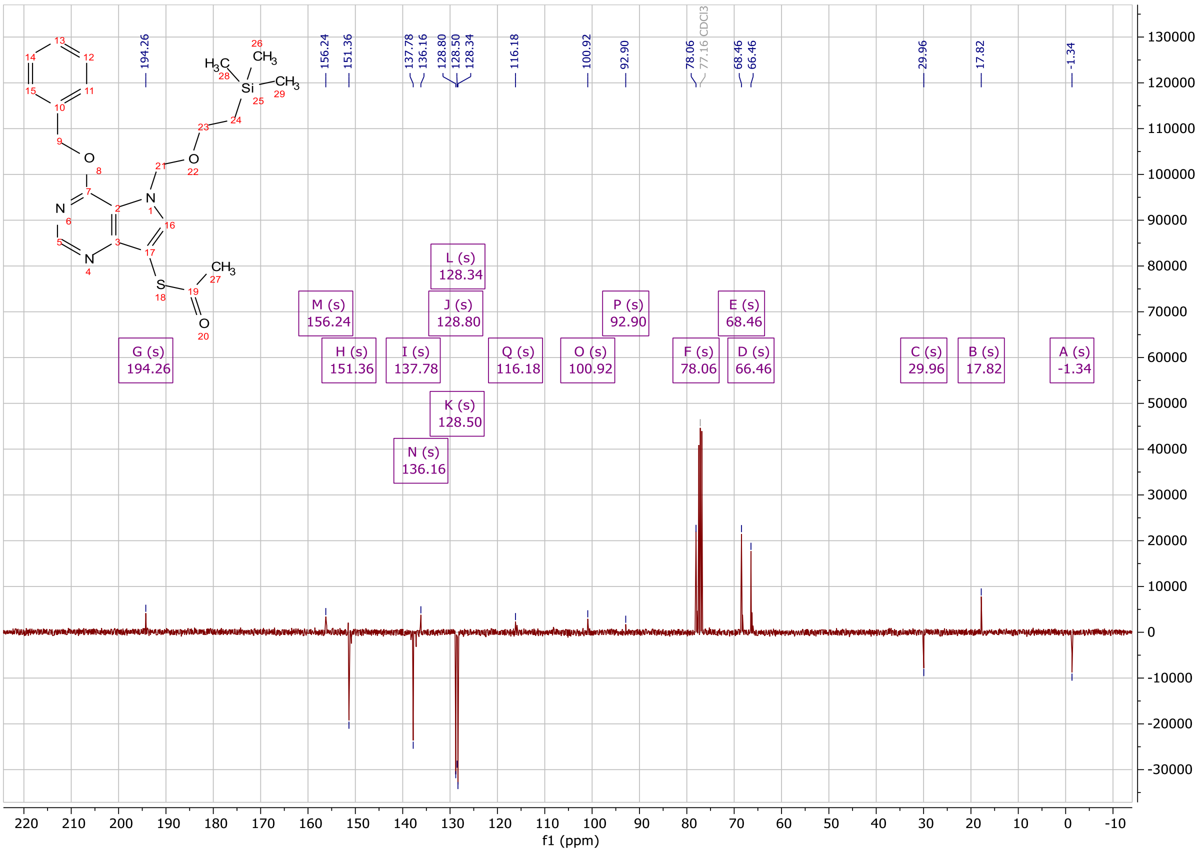

194.26
156.24
151.36
137.78
136.16
128.80
128.50
128.34
116.18
100.92
92.90
78.06
77.16 CDCl3
68.46
66.46
29.96
17.82
-1.34
G (s) 194.26
M (s) 156.24
H (s) 151.36
I (s) 137.78
N (s) 136.16
J (s) 128.80
K (s) 128.50
L (s) 128.34
Q (s) 116.18
O (s) 100.92
P (s) 92.90
F (s) 78.06
E (s) 68.46
D (s) 66.46
C (s) 29.96
B (s) 17.82
A (s) -1.34
f1 (ppm)

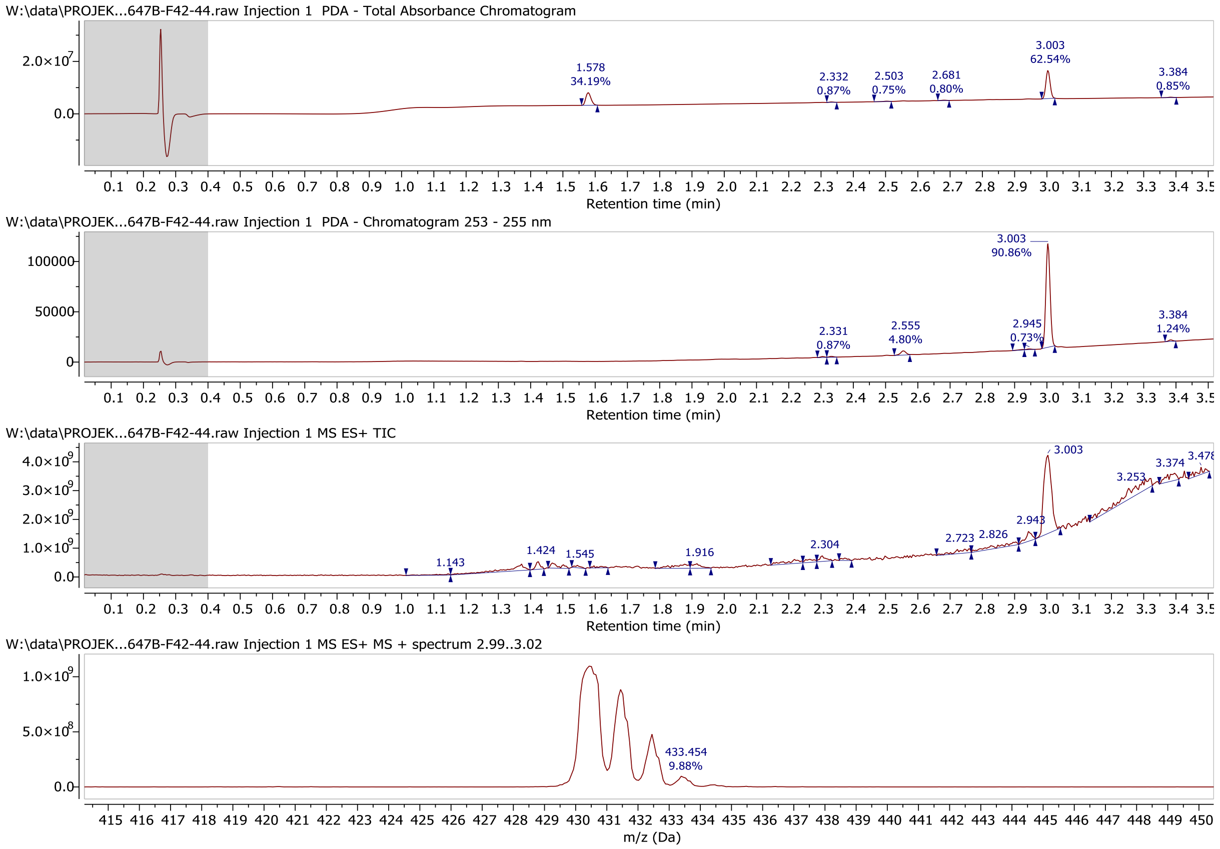

W:\data\PROJEK...647B-F42-44.raw Injection 1 PDA - Total Absorbance Chromatogram
1.578 34.19%
2.332 0.87%
2.503 0.75%
2.681 0.80%
3.003 62.54%
3.384 0.85%
Retention time (min)
W:\data\PROJEK...647B-F42-44.raw Injection 1 PDA - Chromatogram 253 - 255 nm
2.331 0.87%
2.555 4.80%
2.945 0.73%
3.003 90.86%
3.384 1.24%
Retention time (min)
W:\data\PROJEK...647B-F42-44.raw Injection 1 MS ES+ TIC
1.143
1.424
1.545
1.916
2.304
2.723
2.826
2.943
3.003
3.253
3.374
Retention time (min)
W:\data\PROJEK...647B-F42-44.raw Injection 1 MS ES+ MS + spectrum 2.99..3.02
433.454 9.88%
m/z (Da)

3.2.17 1,2-bis(4-(benzyloxy)-5-((2-(trimethylsilyl)ethoxy)methyl)-5*H*-pyrrolo[3,2-*d*]pyrimidin-7-yl)disulfane (9)

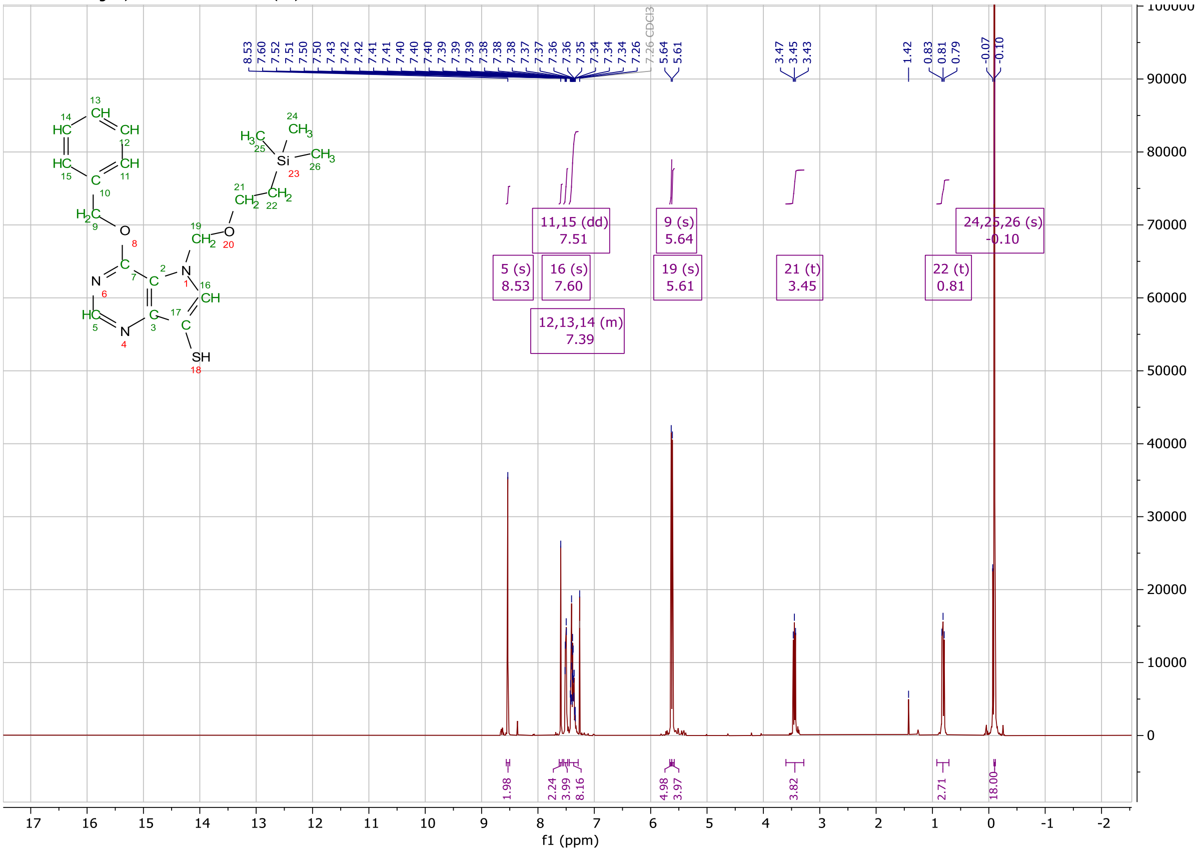

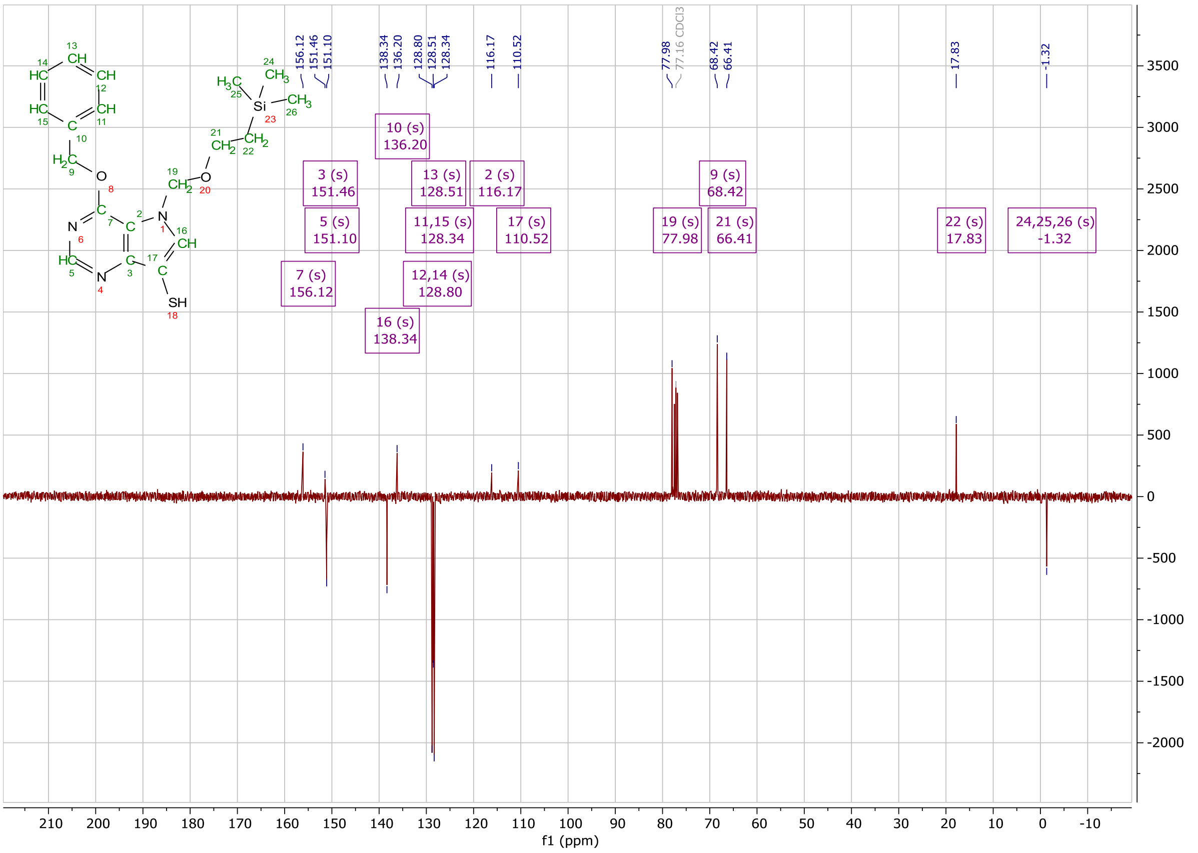

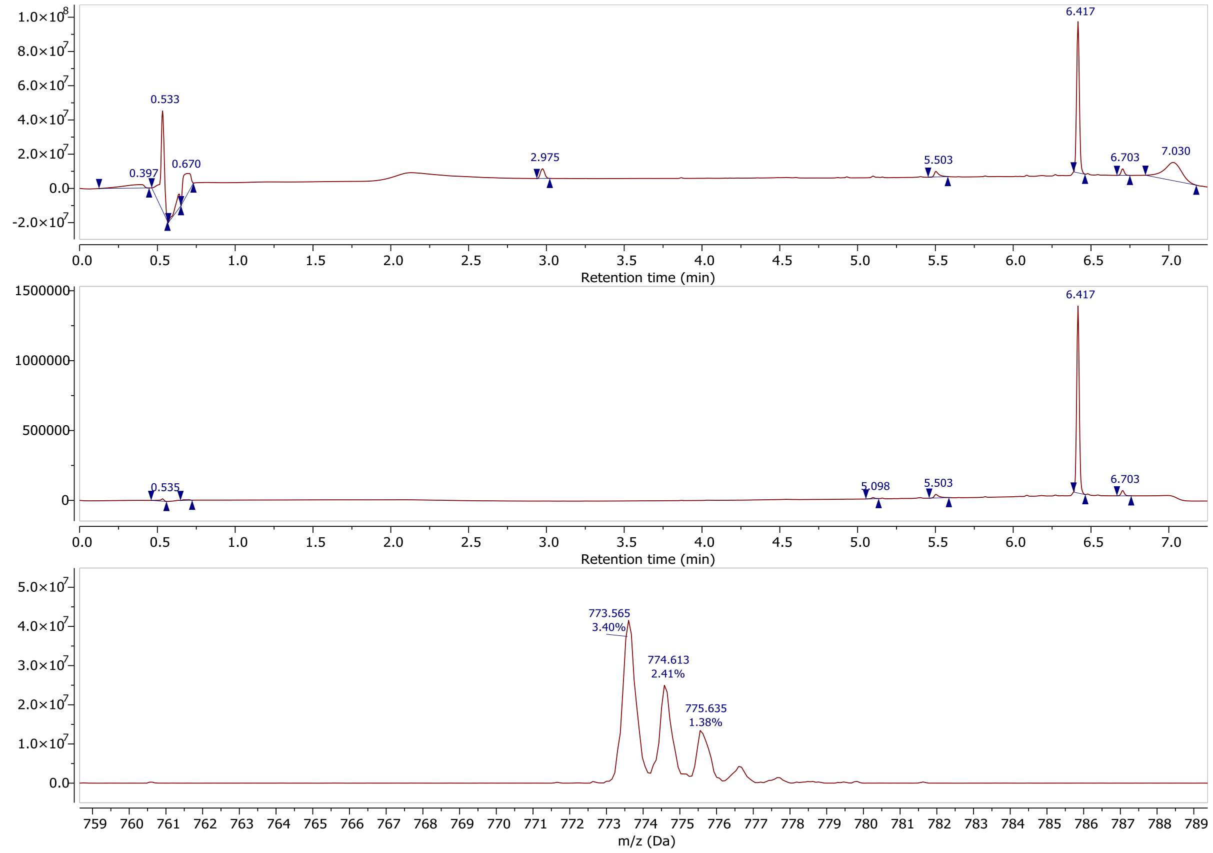

3.2.18 Sodium 7-((2-((phosphonato)methoxy)ethyl)thio)-3,5-dihydro-4*H*-pyrrolo[3,2-*d*]pyrimidin-4-one (13a)

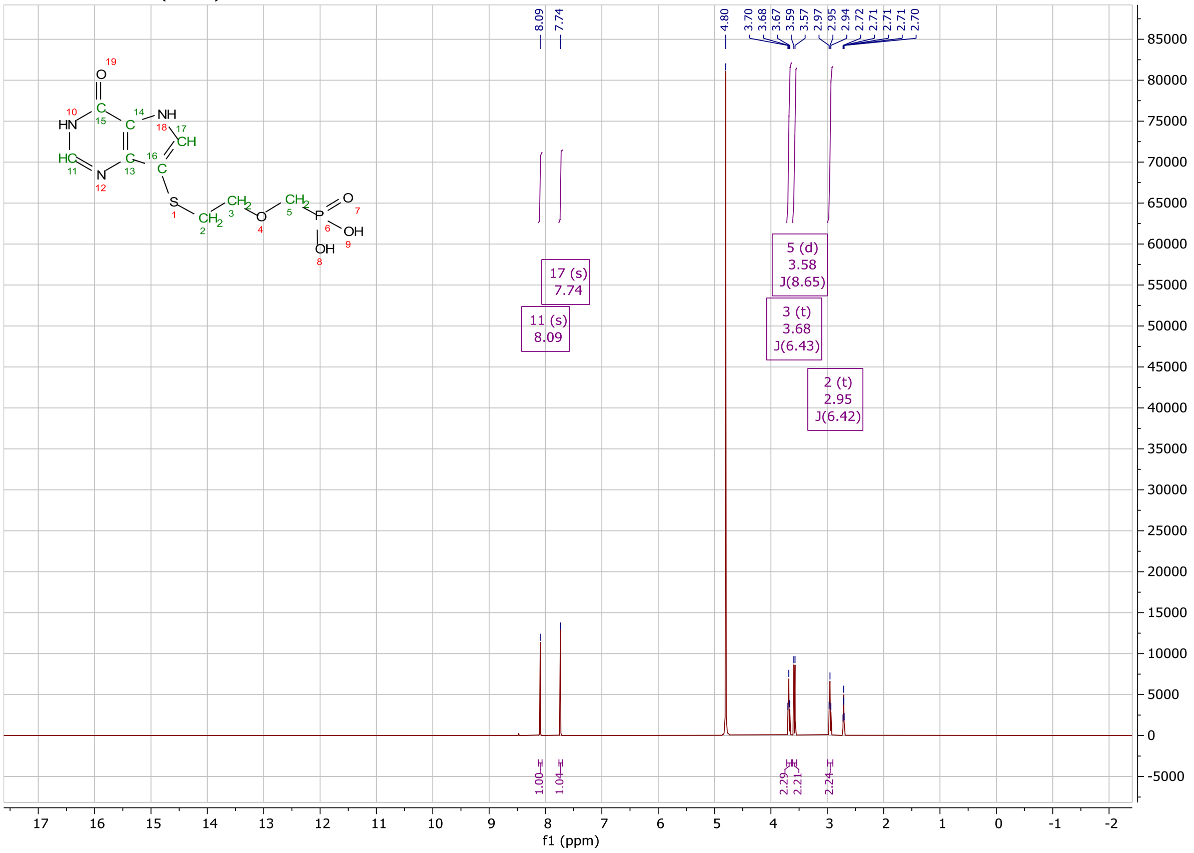

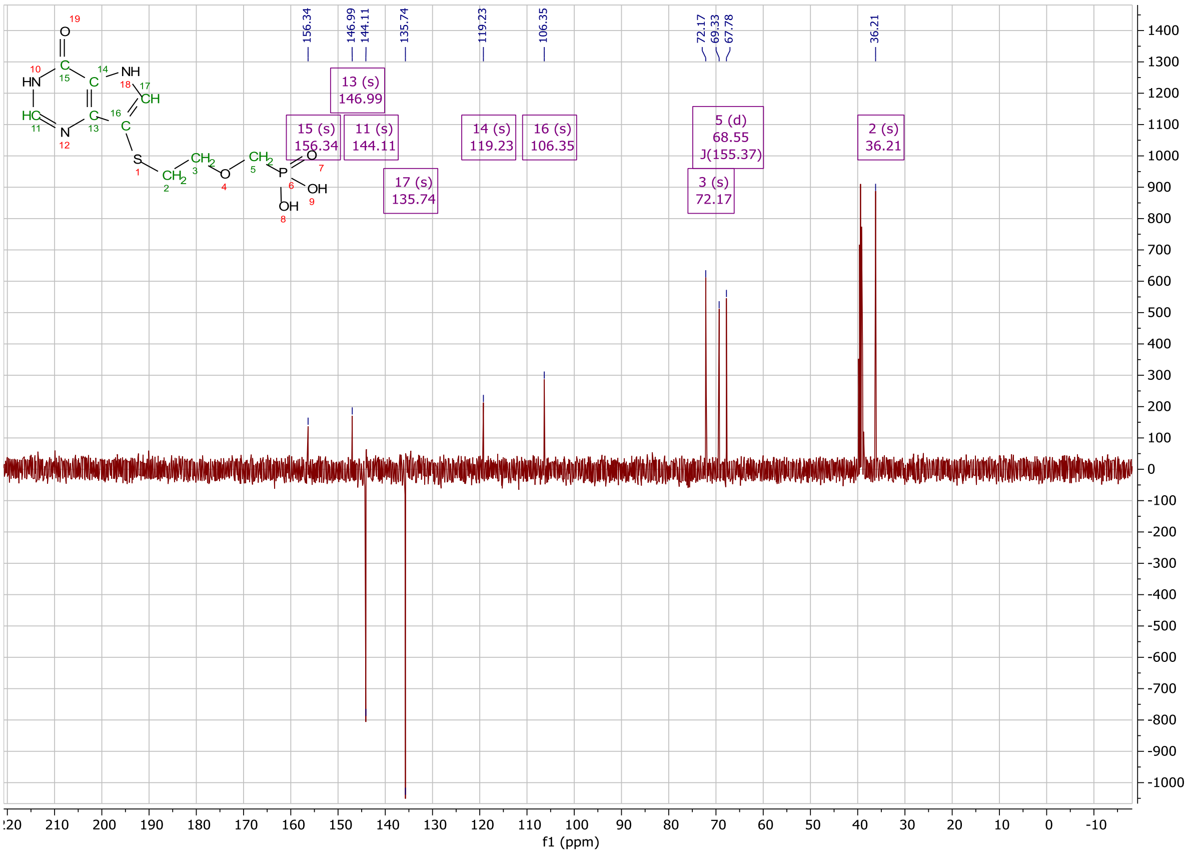
156.34
146.99
144.11
135.74
119.23
106.35
72.17
69.33
67.78
36.21
13 (s)
146.99
15 (s)
156.34
11 (s)
144.11
17 (s)
135.74
14 (s)
119.23
16 (s)
106.35
5 (d)
68.55
J(155.37)
3 (s)
72.17
2 (s)
36.21
f1 (ppm)

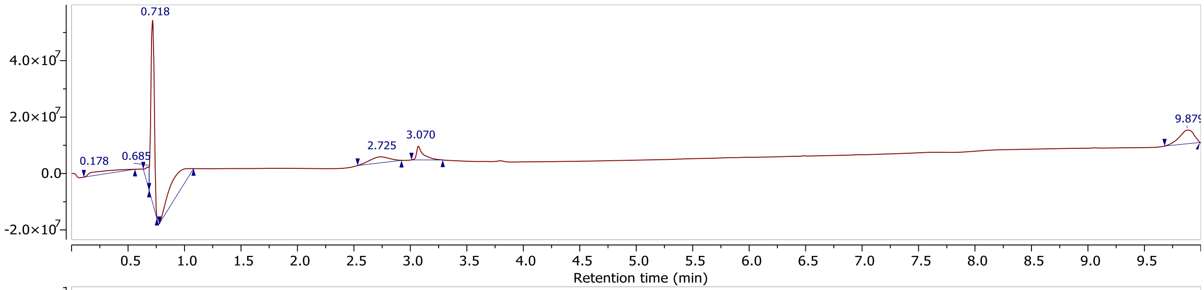
0.718
0.178
0.685
2.725
3.070
Retention time (min)

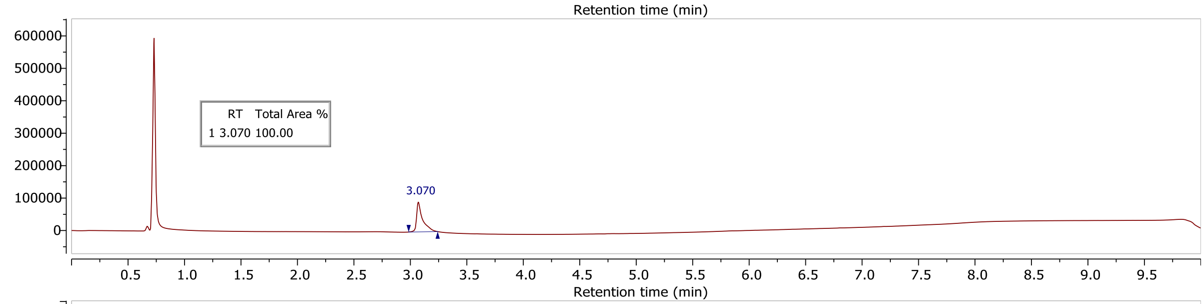
RT Total Area %
1 3.070 100.00
3.070
Retention time (min)

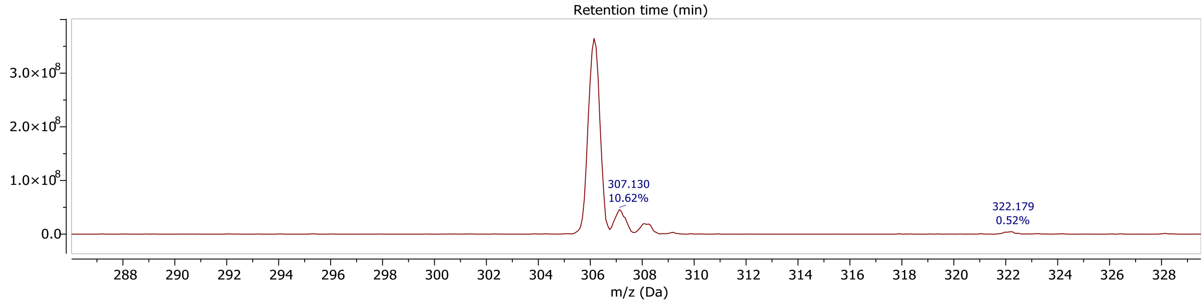
307.130
10.62%
322.179
0.52%
m/z (Da)

3.2.19 Sodium 7-((2-(2-(phosphonato)ethoxy)ethyl)thio)-3,5-dihydro-4*H*-pyrrolo[3,2-*d*]pyrimidin-4-one (13b)

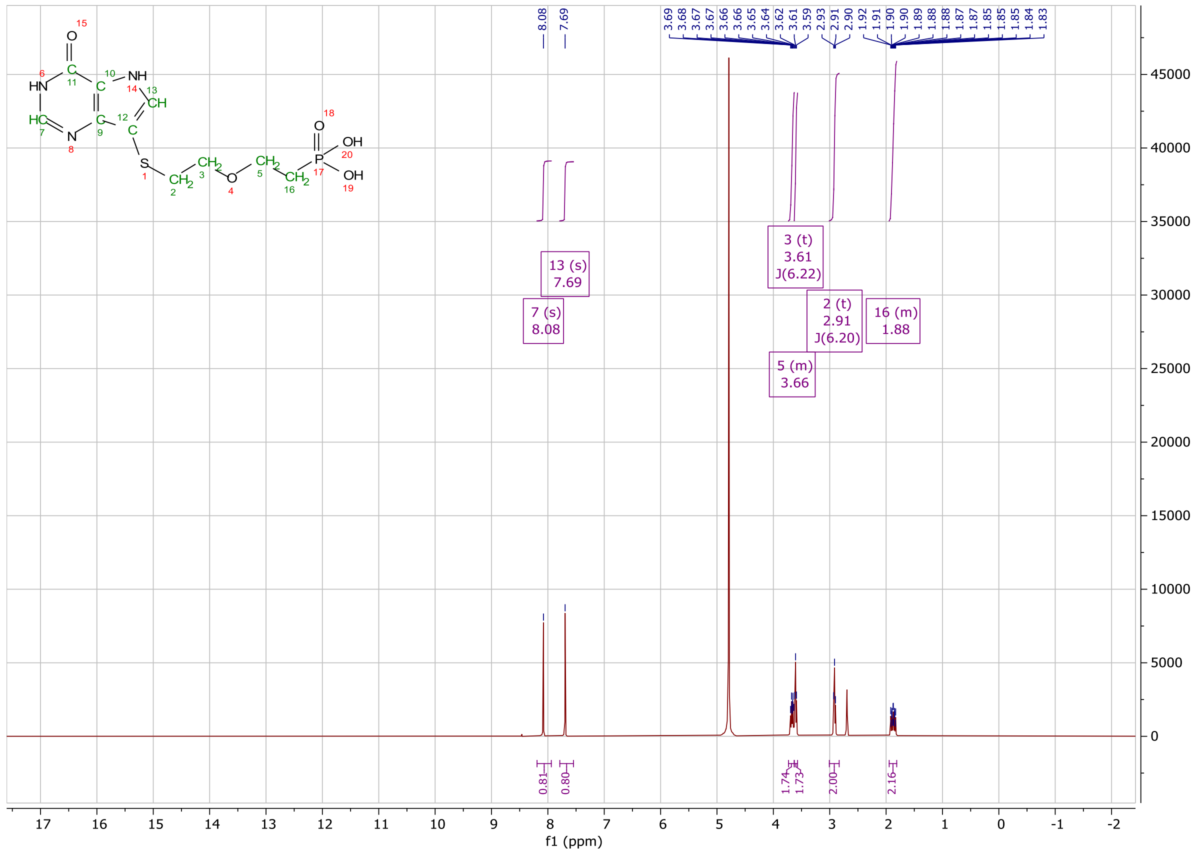

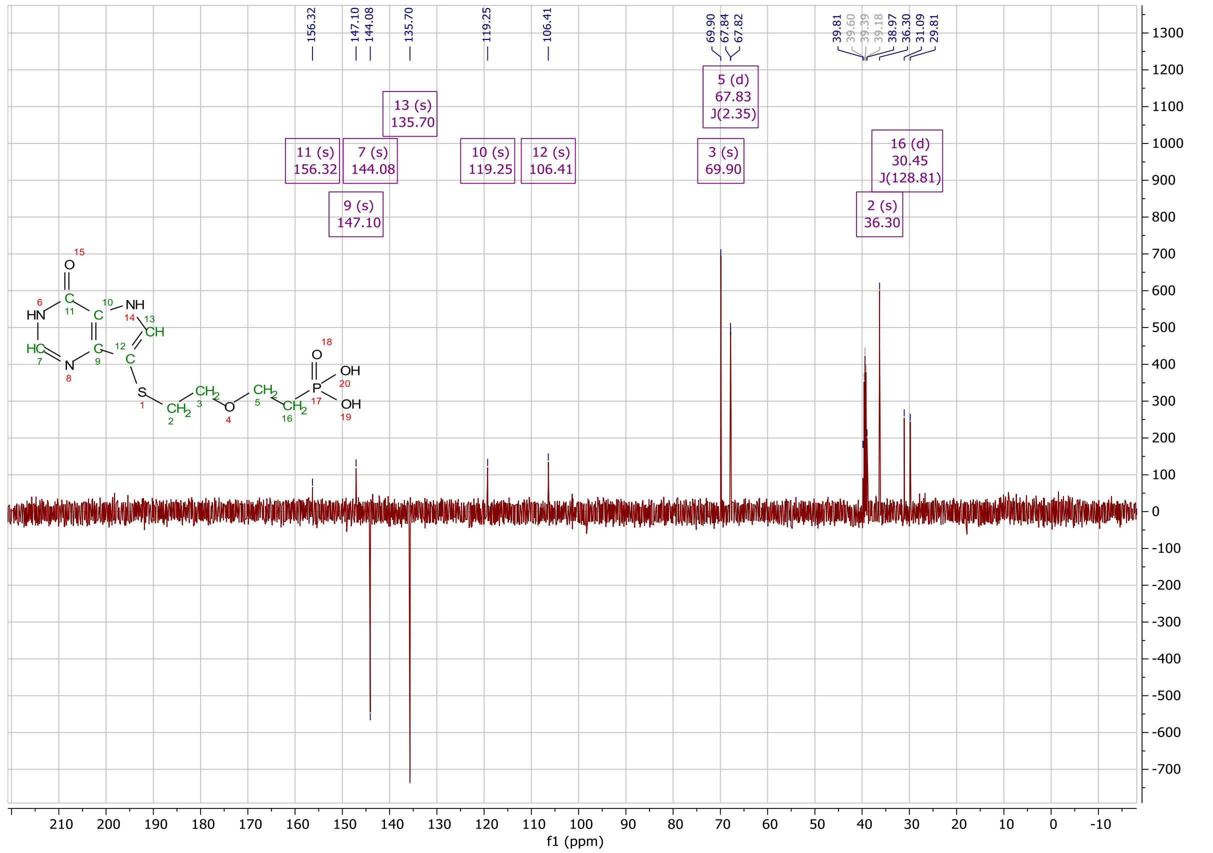

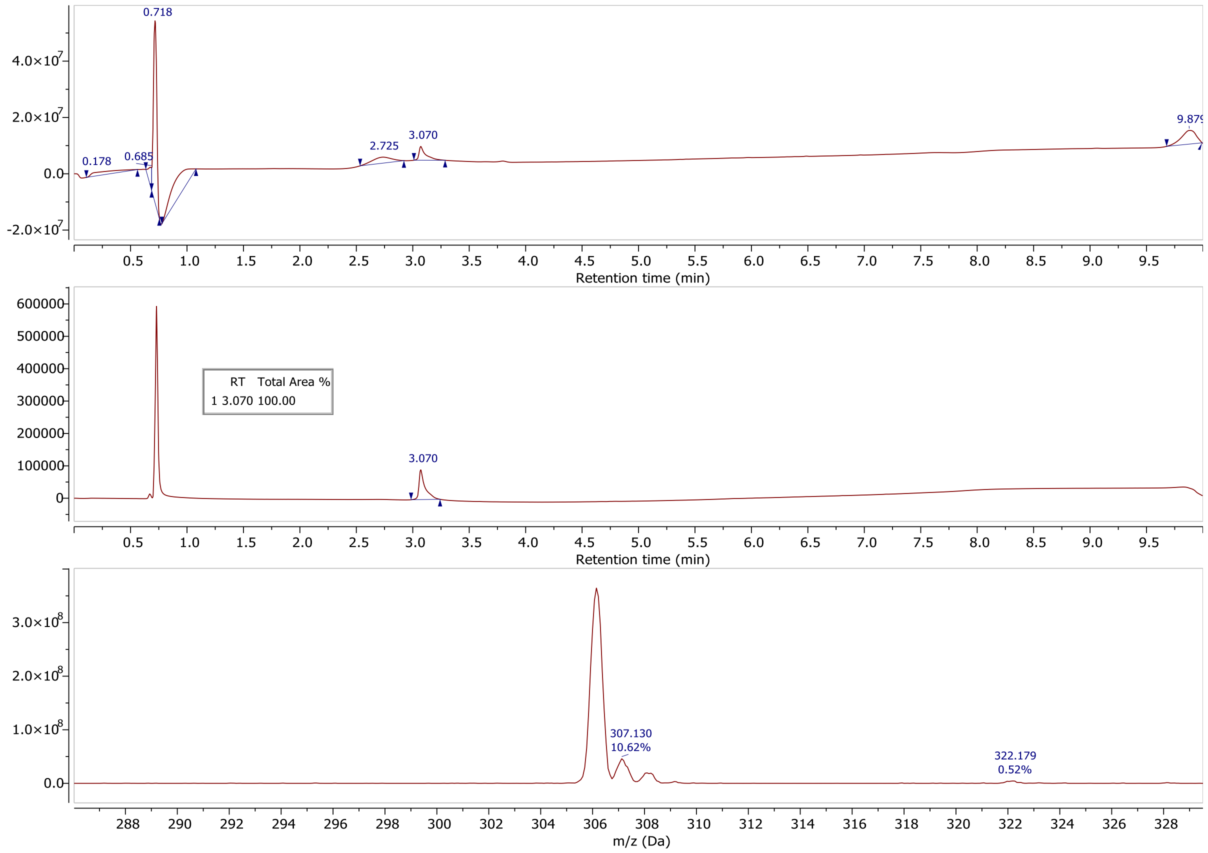

3.2.20 Diisopropyl (2-iodobenzyl)phosphonate (15a)

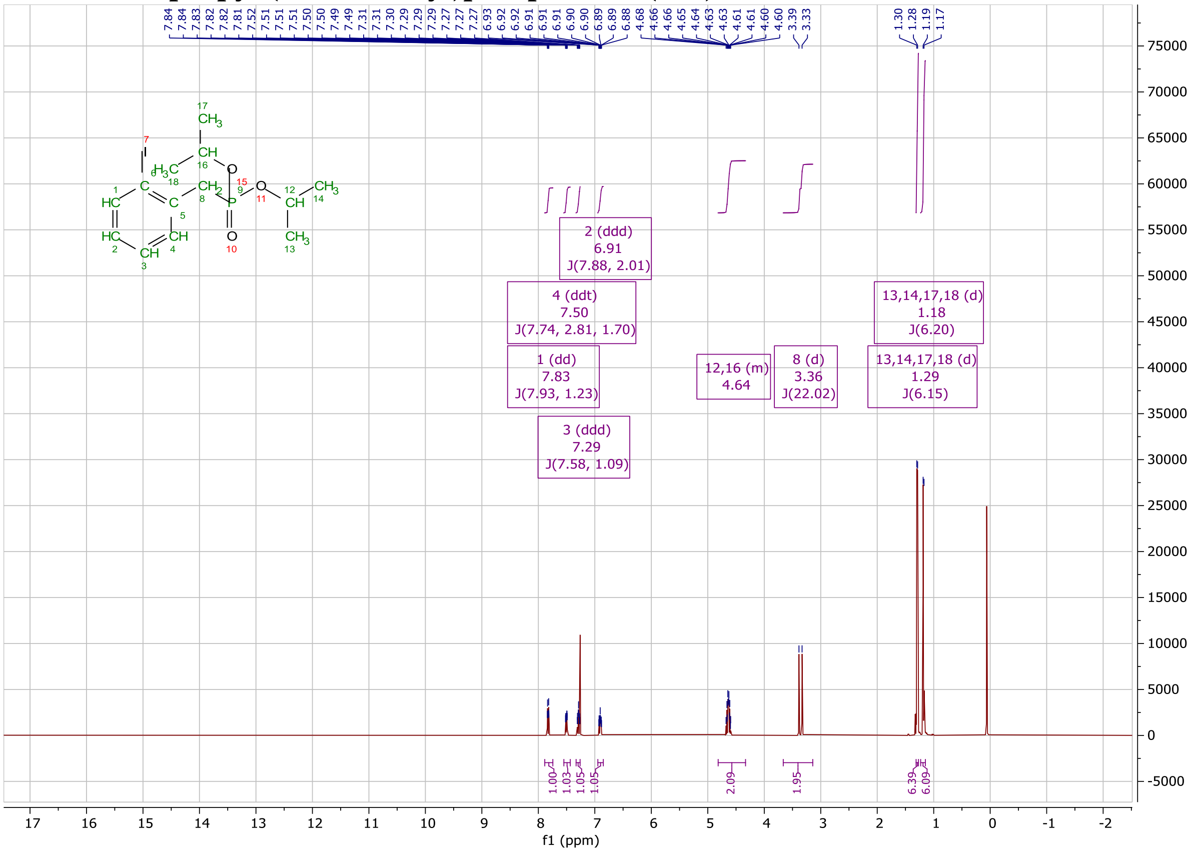

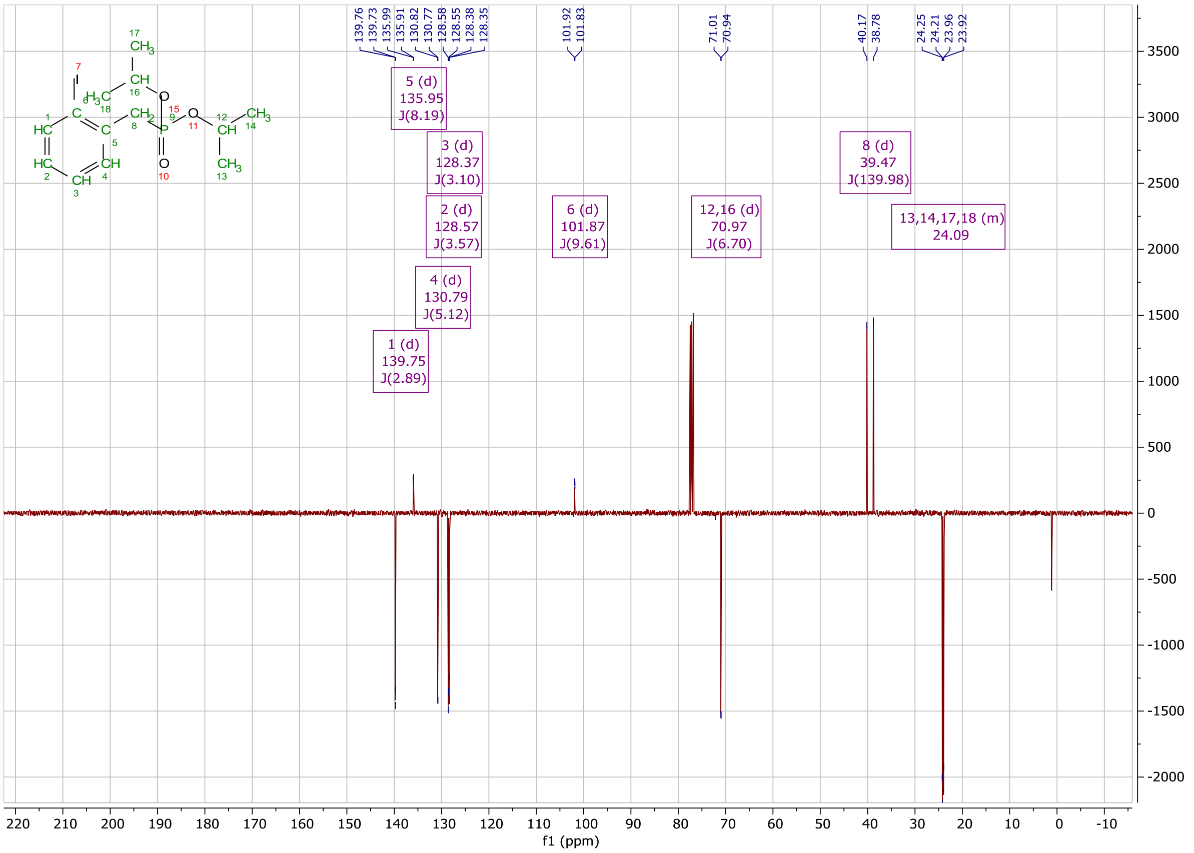
139.76
139.73
135.99
135.91
130.82
130.77
128.58
128.55
128.38
128.35
101.92
101.83
71.01
70.94
40.17
38.78
24.25
24.21
23.96
23.92
5 (d)
135.95
J(8.19)
3 (d)
128.37
J(3.10)
2 (d)
128.57
J(3.57)
4 (d)
130.79
J(5.12)
1 (d)
139.75
J(2.89)
6 (d)
101.87
J(9.61)
12,16 (d)
70.97
J(6.70)
8 (d)
39.47
J(139.98)
13,14,17,18 (m)
24.09
f1 (ppm)

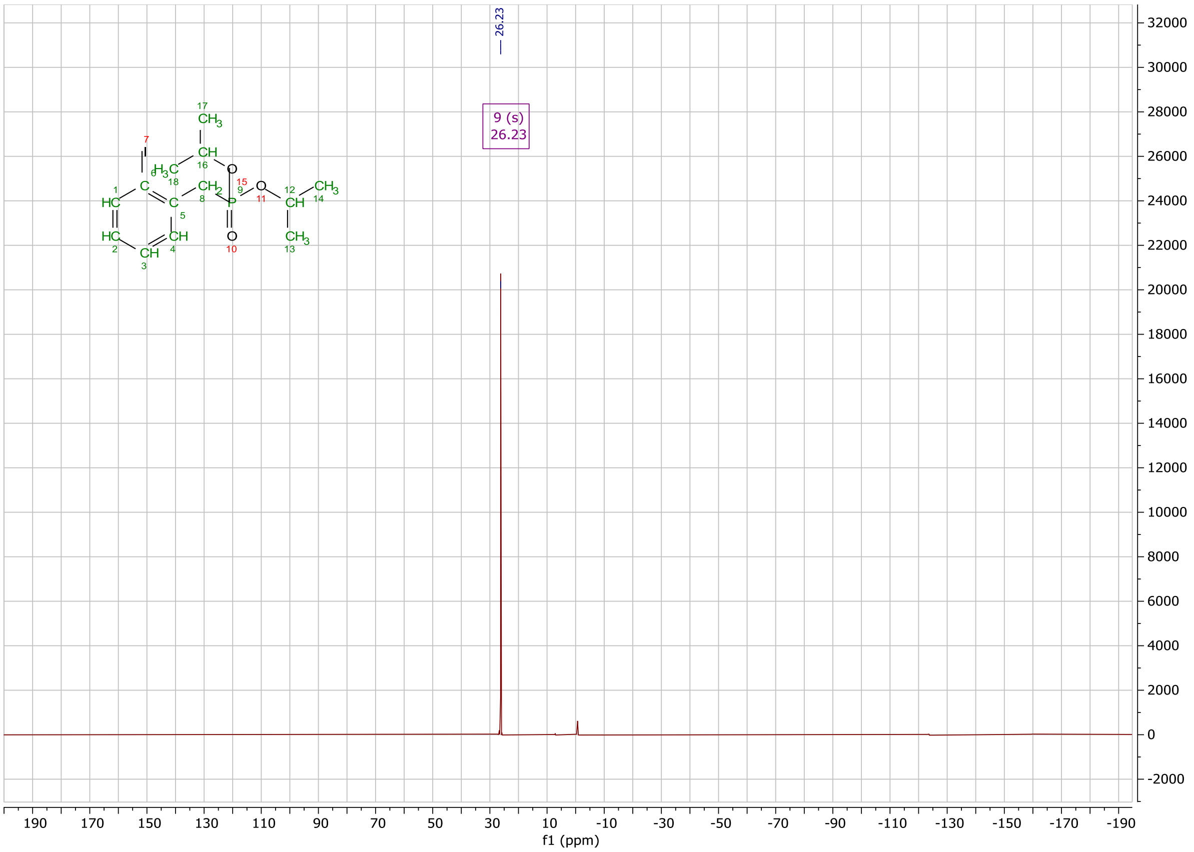
26.23
9 (s)
26.23
f1 (ppm)

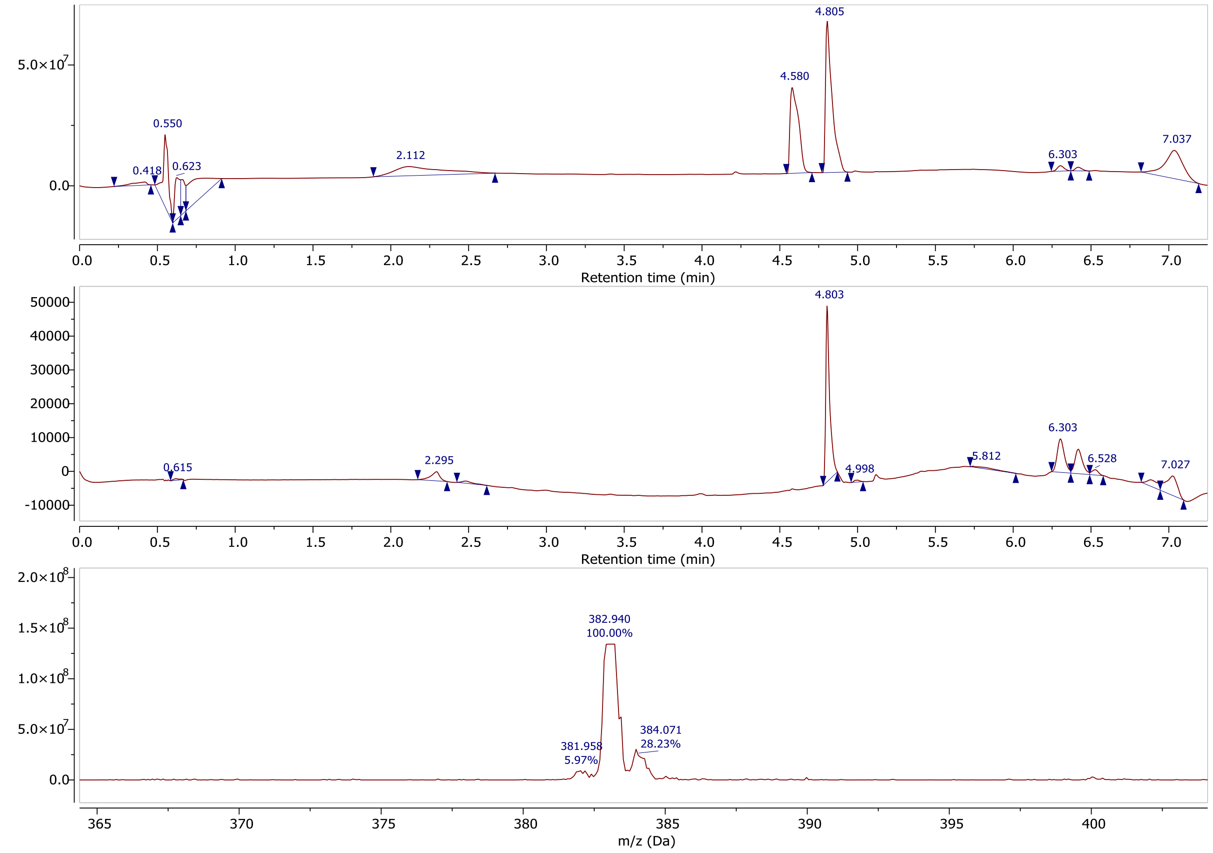

3.2.21 Diethyl (*E*)-(2-iodostyryl)phosphonate (15b)

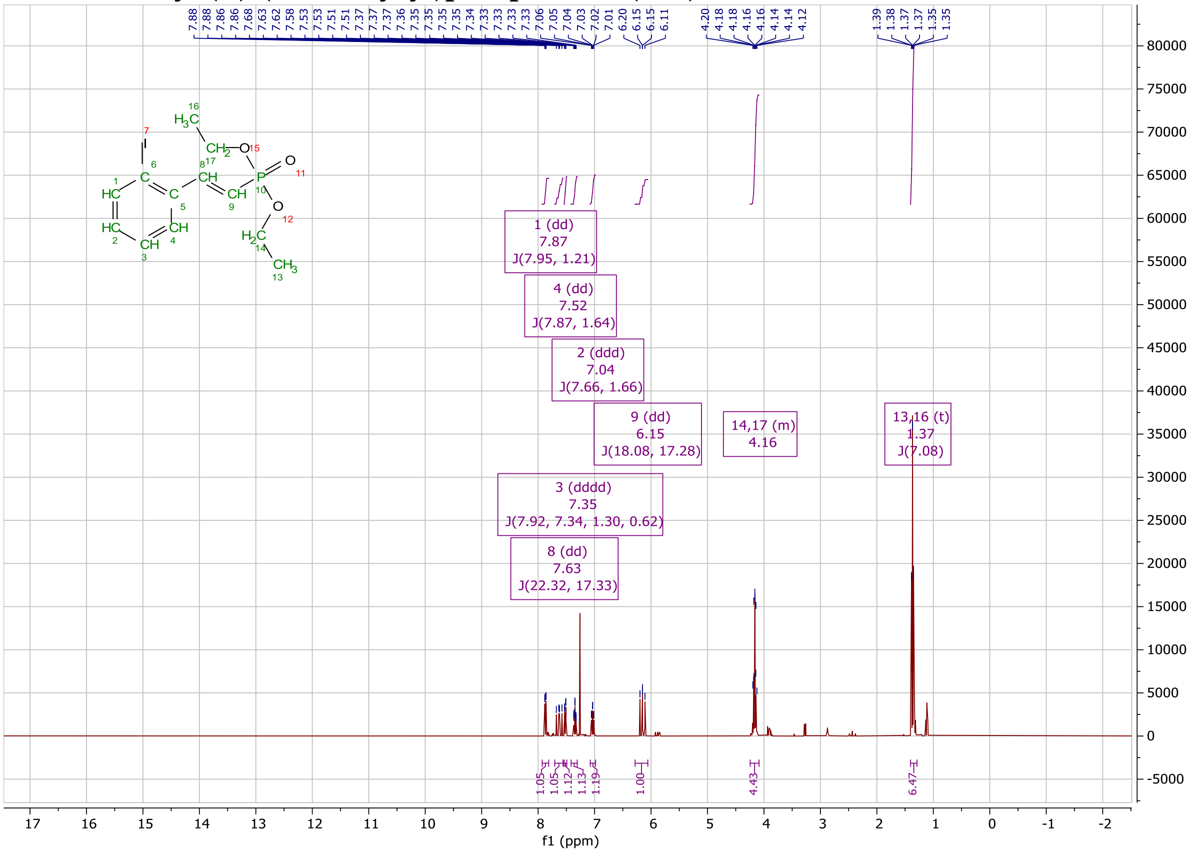

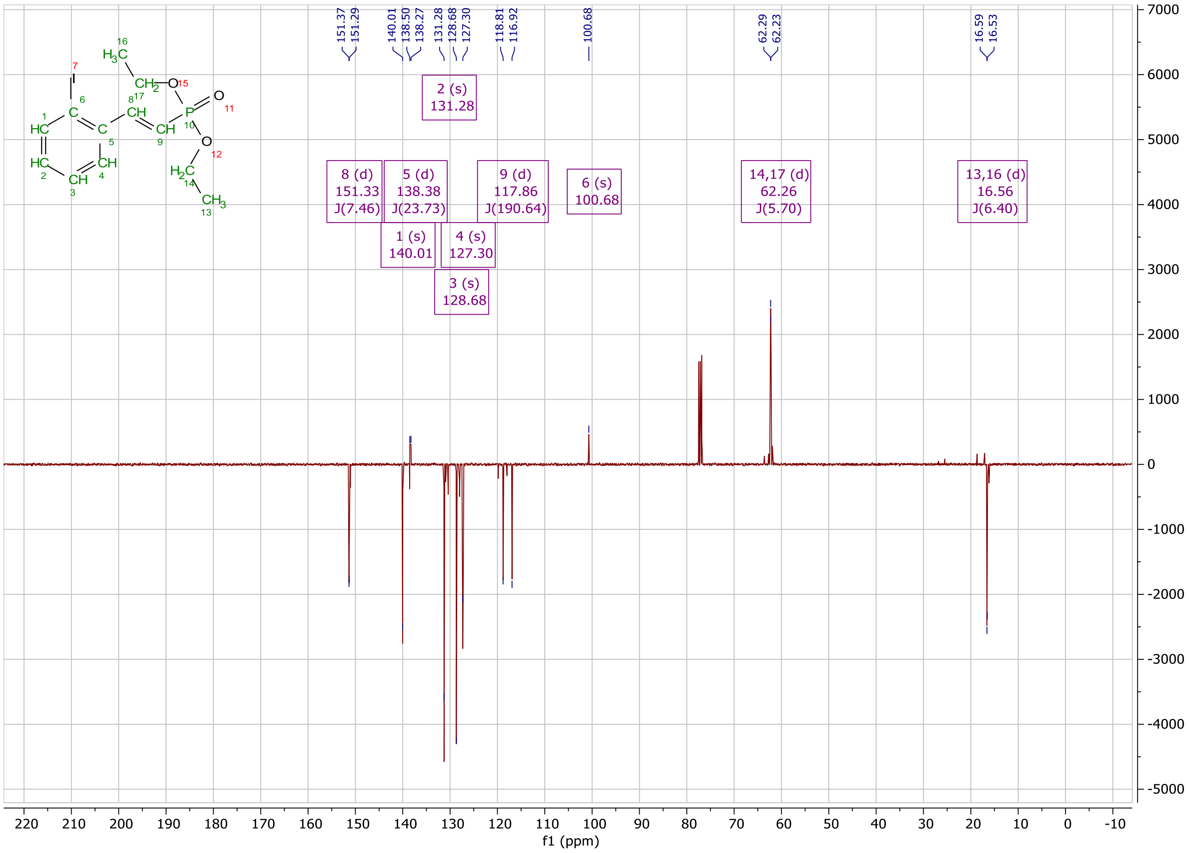

151.37
151.29
140.01
138.50
138.27
131.28
128.68
127.30
118.81
116.92
100.68
62.29
62.23
16.59
16.53
2 (s) 131.28
8 (d) 151.33 J(7.46)
5 (d) 138.38 J(23.73)
9 (d) 117.86 J(190.64)
6 (s) 100.68
14,17 (d) 62.26 J(5.70)
13,16 (d) 16.56 J(6.40)
1 (s) 140.01
4 (s) 127.30
3 (s) 128.68
f1 (ppm)

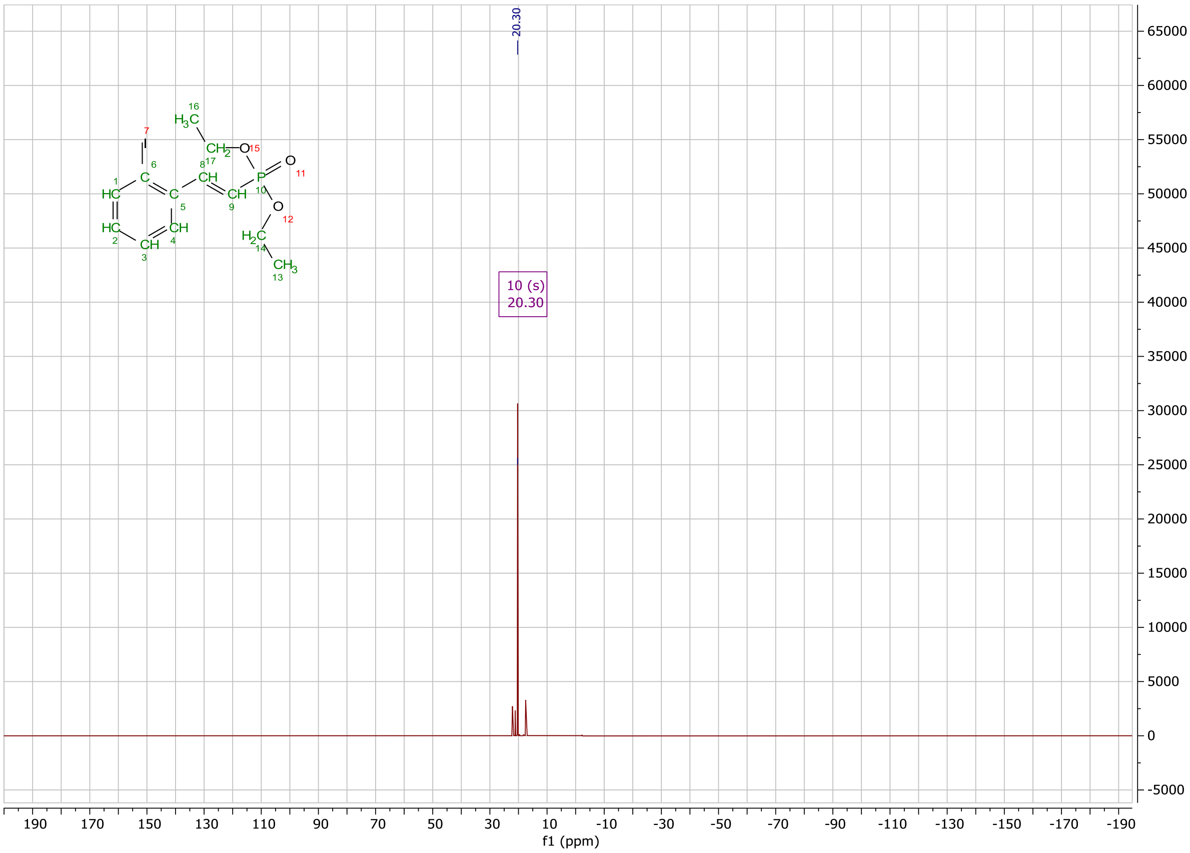

20.30
10 (s) 20.30
f1 (ppm)

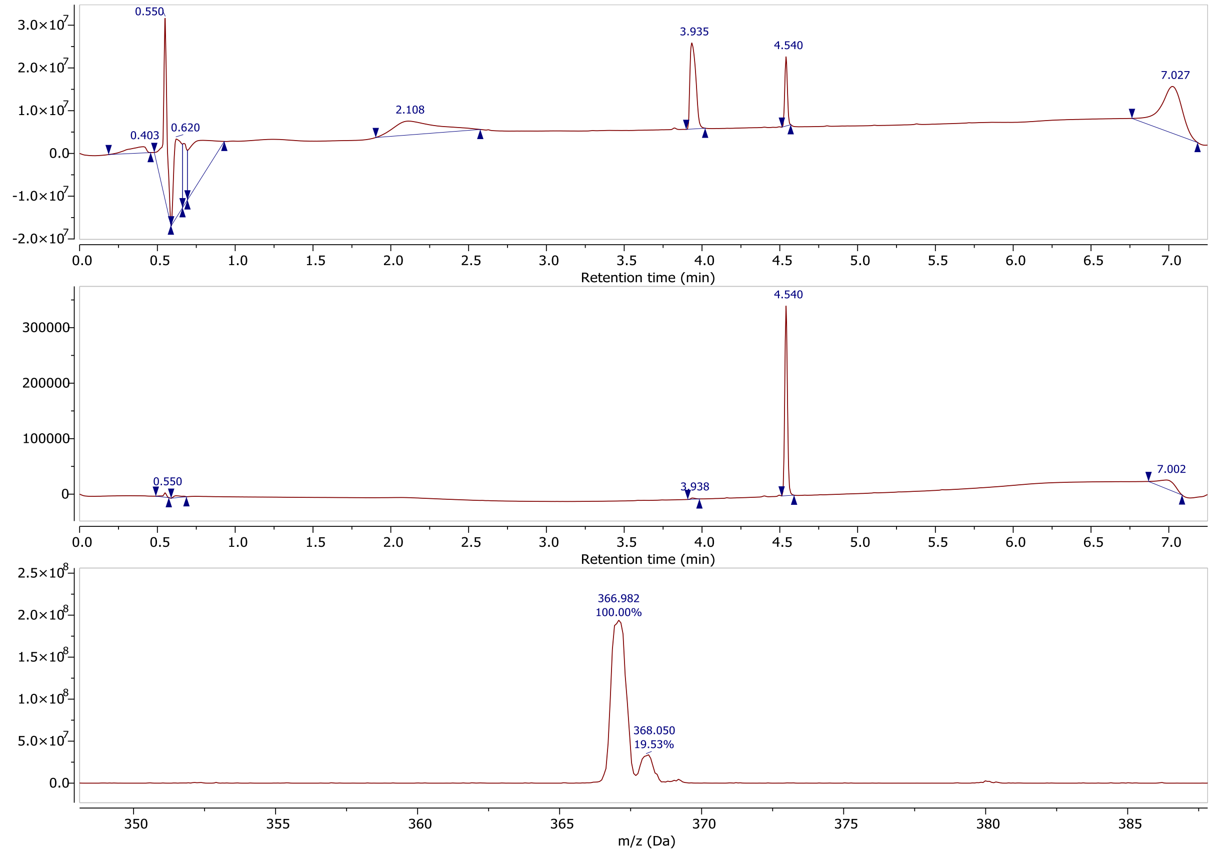

3.2.22 Diisopropyl ((2-iodophenoxy)methyl)phosphonate (15c)

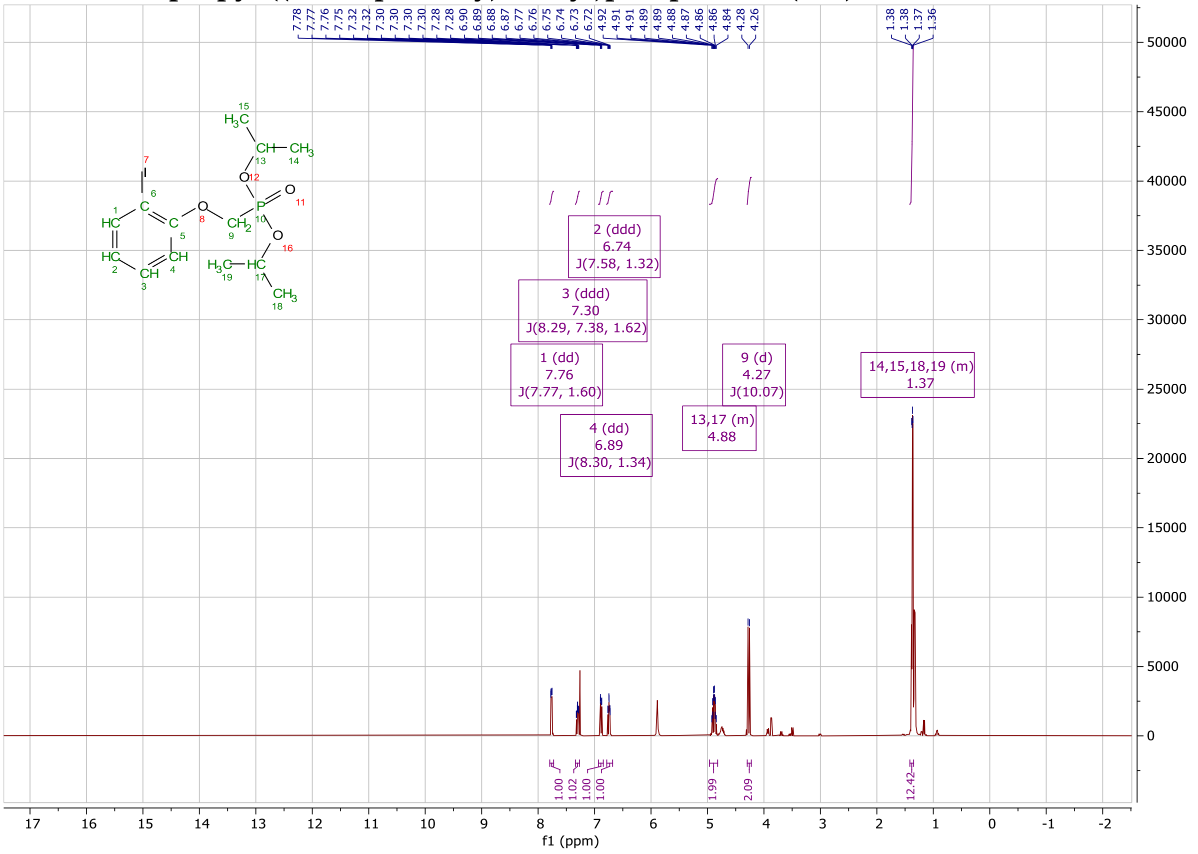

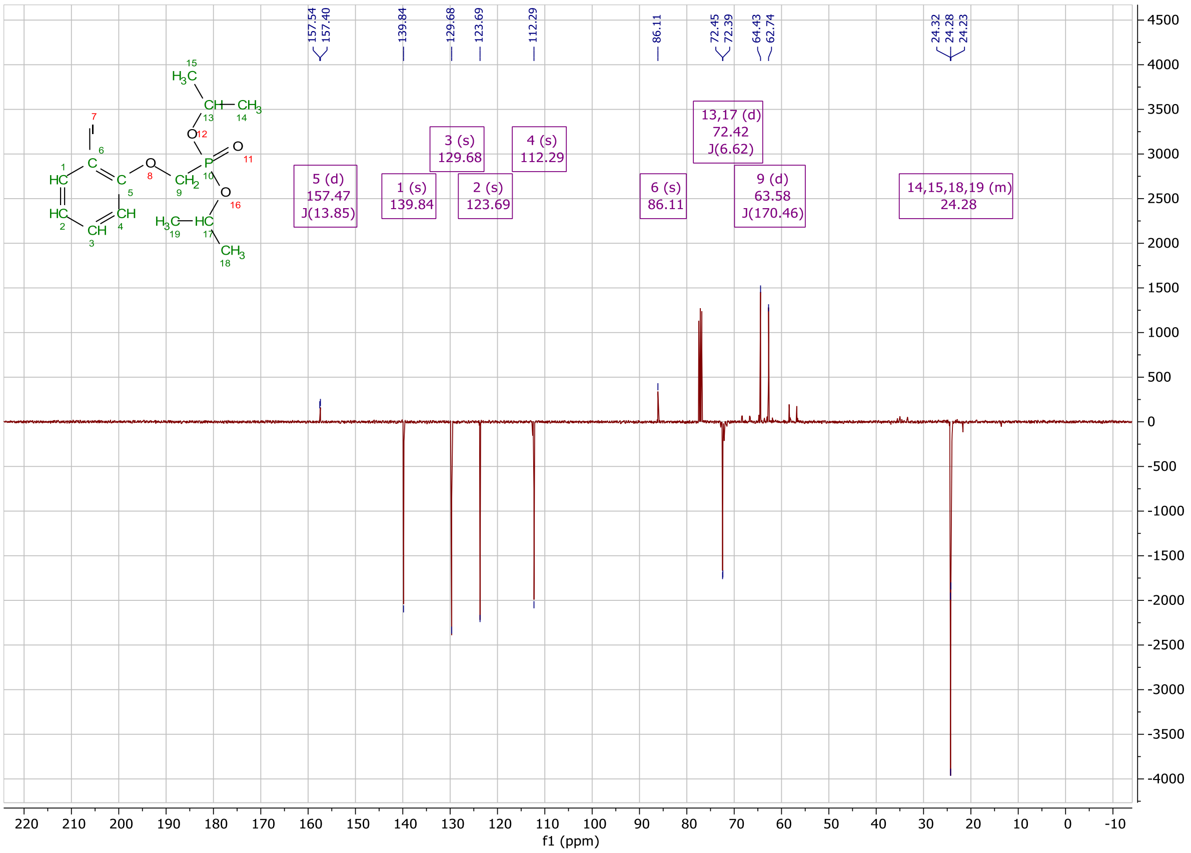

157.54
157.40
139.84
129.68
123.69
112.29
86.11
72.45
72.39
64.43
62.74
24.32
24.28
24.23
5 (d) 157.47 J(13.85)
1 (s) 139.84
3 (s) 129.68
2 (s) 123.69
4 (s) 112.29
6 (s) 86.11
13,17 (d) 72.42 J(6.62)
9 (d) 63.58 J(170.46)
14,15,18,19 (m) 24.28
f1 (ppm)

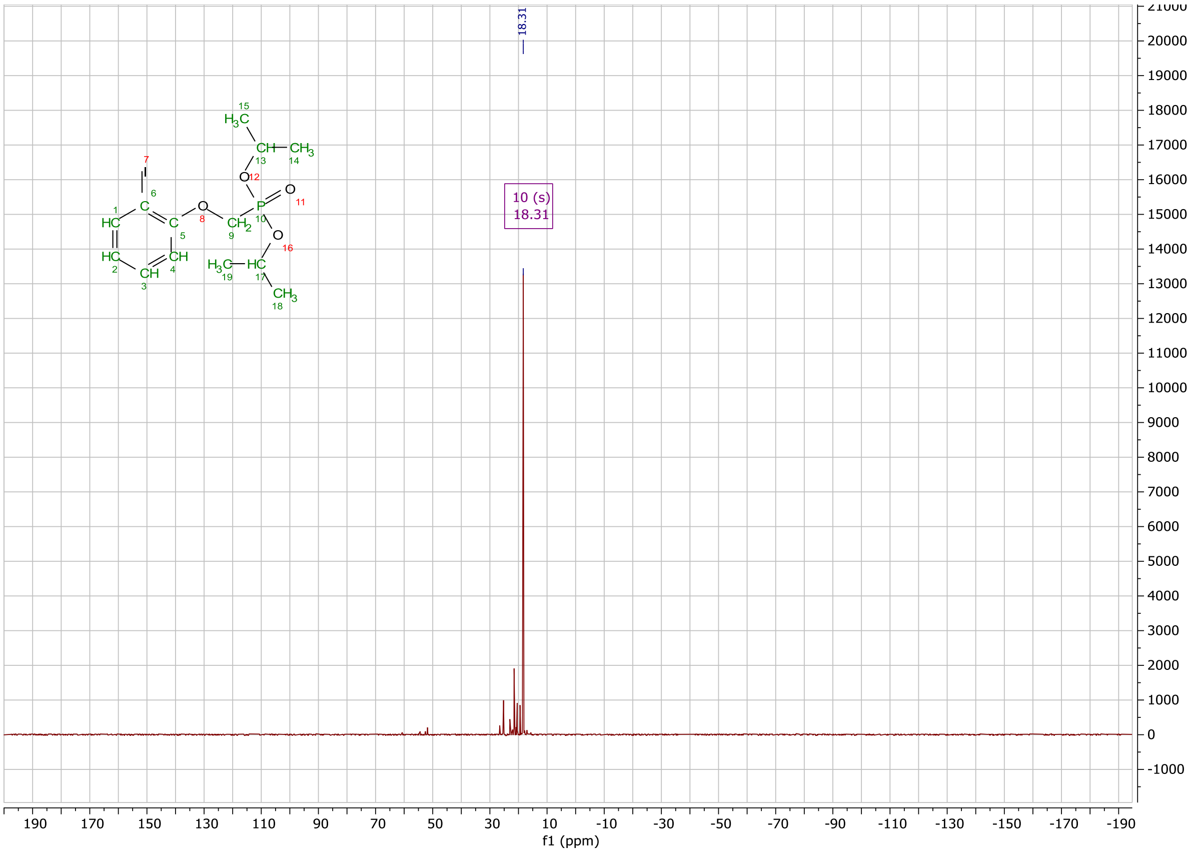

18.31
10 (s) 18.31
f1 (ppm)

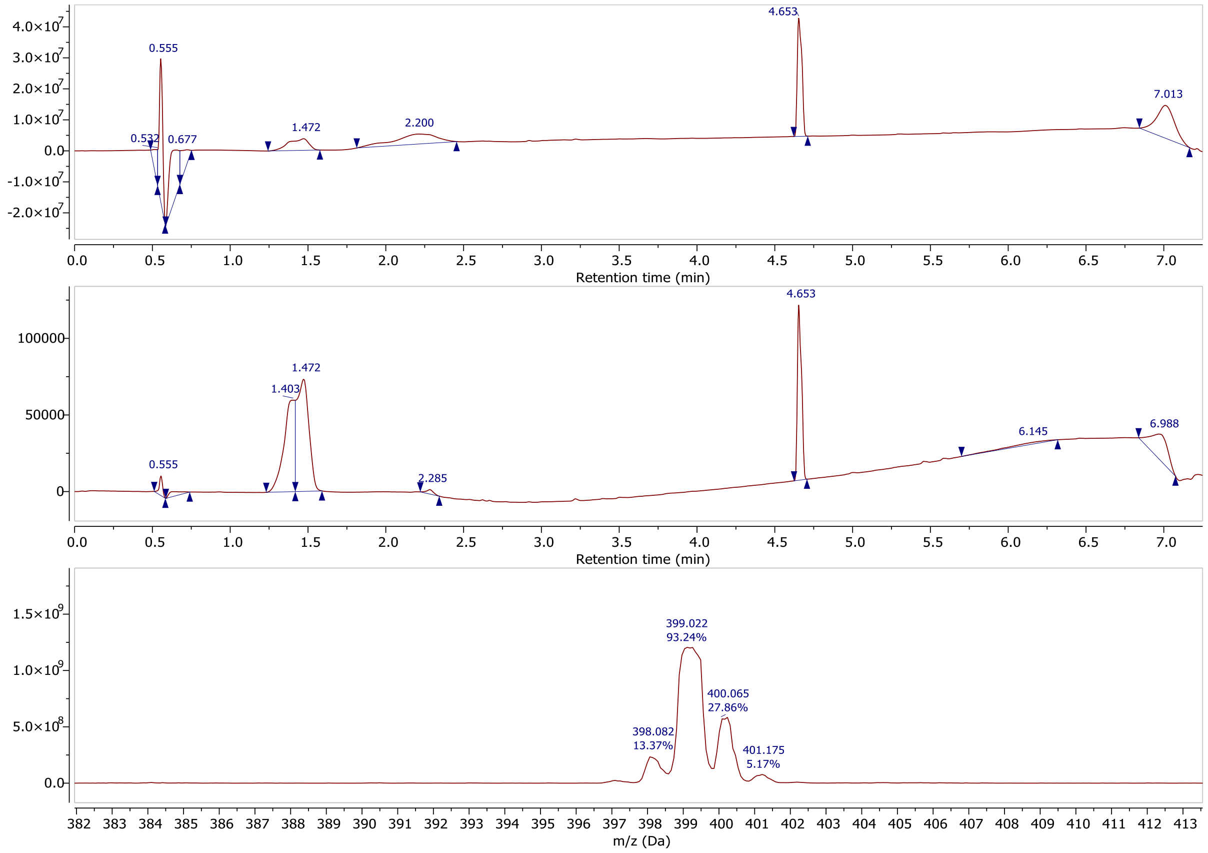

3.2.23 Sodium 7-((2-((phosphonato)methyl)phenyl)thio)-3,5-dihydro-4*H*-pyrrolo[3,2-*d*]pyrimidin-4-one (18a)

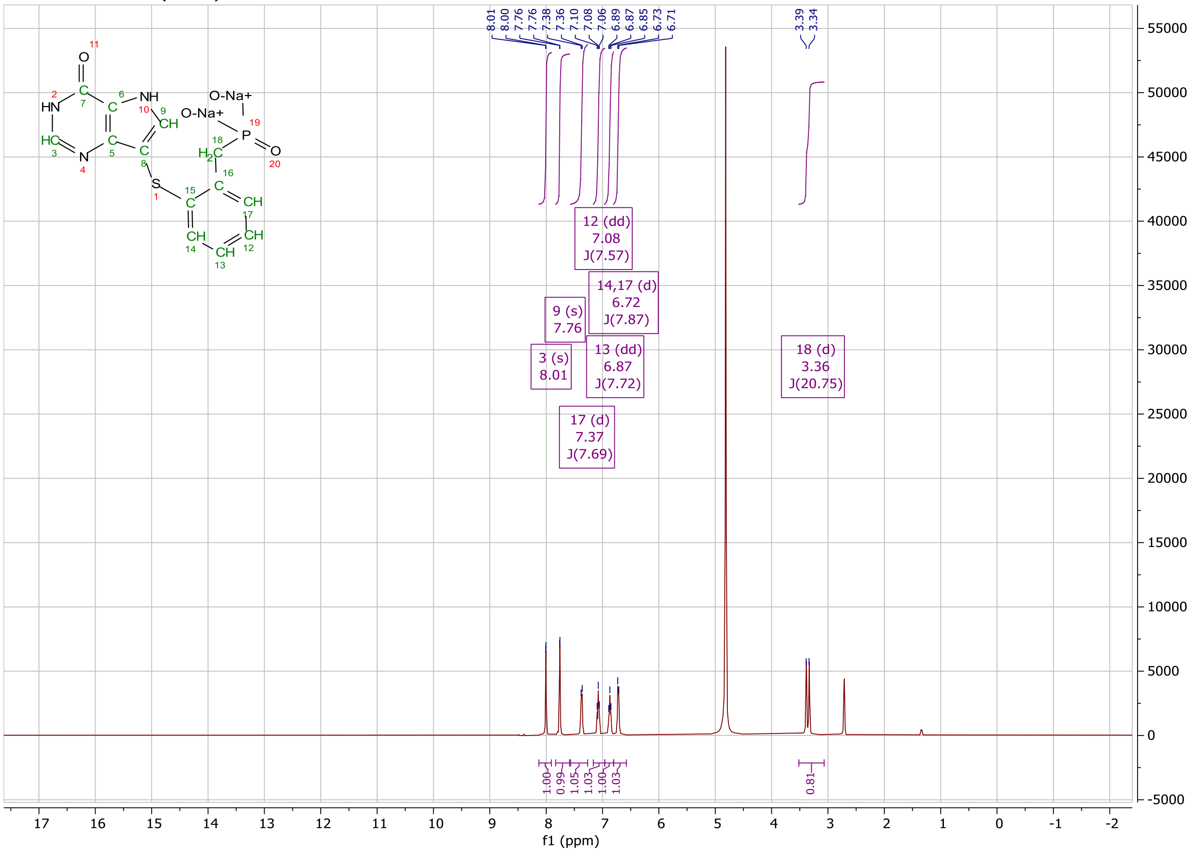

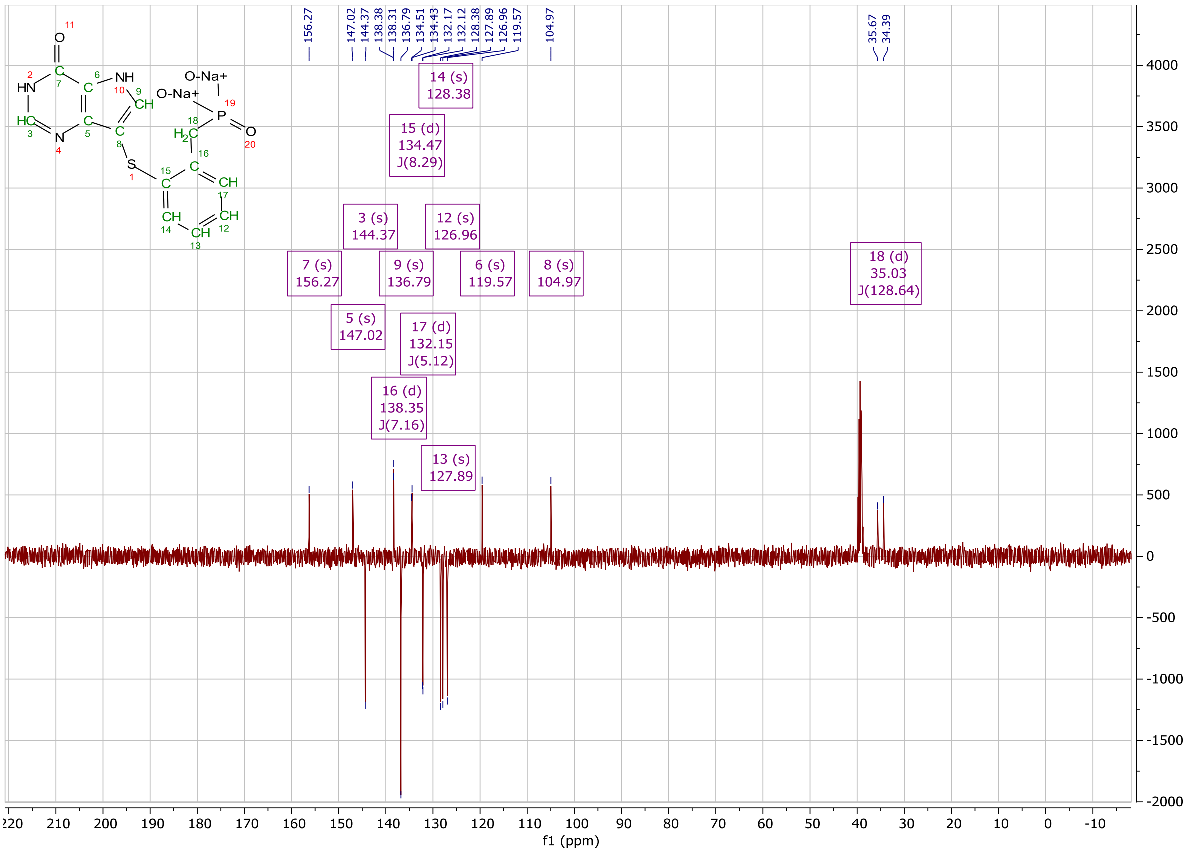

156.27
147.02
144.37
138.38
138.31
136.79
134.51
134.43
132.17
132.12
128.38
127.89
126.96
119.57
104.97
35.67
34.39
14 (s) 128.38
15 (d) 134.47 J(8.29)
3 (s) 144.37
12 (s) 126.96
7 (s) 156.27
9 (s) 136.79
6 (s) 119.57
8 (s) 104.97
18 (d) 35.03 J(128.64)
5 (s) 147.02
17 (d) 132.15 J(5.12)
16 (d) 138.35 J(7.16)
13 (s) 127.89
O-Na+
O-Na+
f1 (ppm)

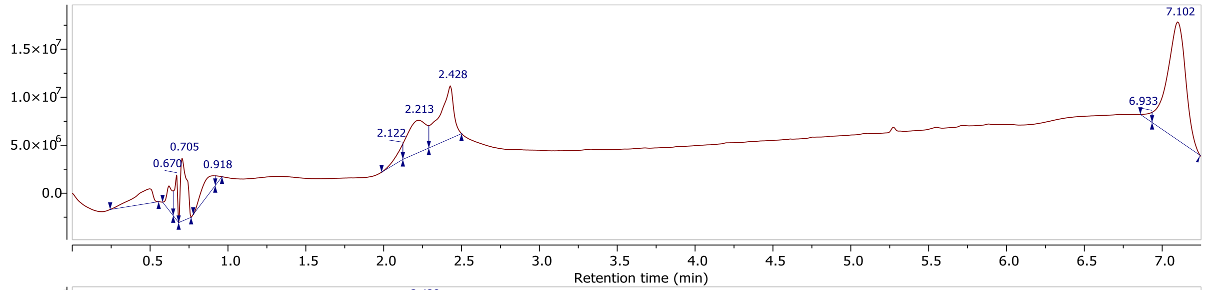

7.102
2.428
6.933
2.213
2.122
0.705
0.670
0.918
Retention time (min)

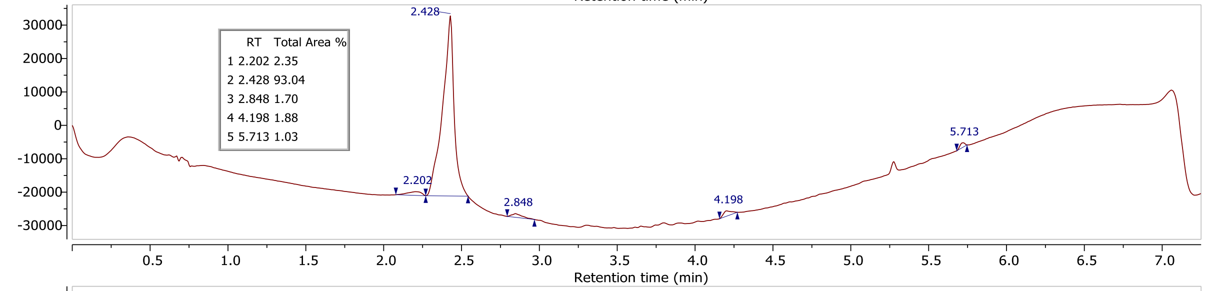

RT Total Area %
1 2.202 2.35
2 2.428 93.04
3 2.848 1.70
4 4.198 1.88
5 5.713 1.03
2.428
2.202
2.848
4.198
5.713
Retention time (min)

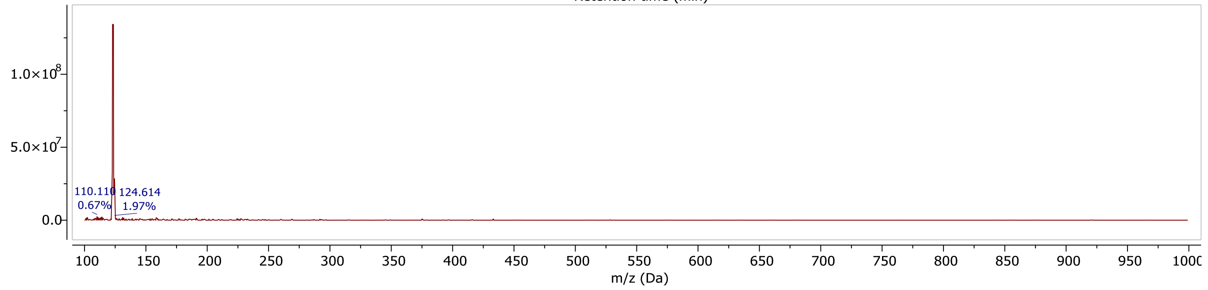

110.110
0.67%
124.614
1.97%
m/z (Da)

3.2.24 Sodium (*E*)-7-((2-(2-(phosphonato)vinyl)phenyl)thio)-3,5-dihydro-4*H*-pyrrolo[3,2-*d*]pyrimidin-4-one (18b)

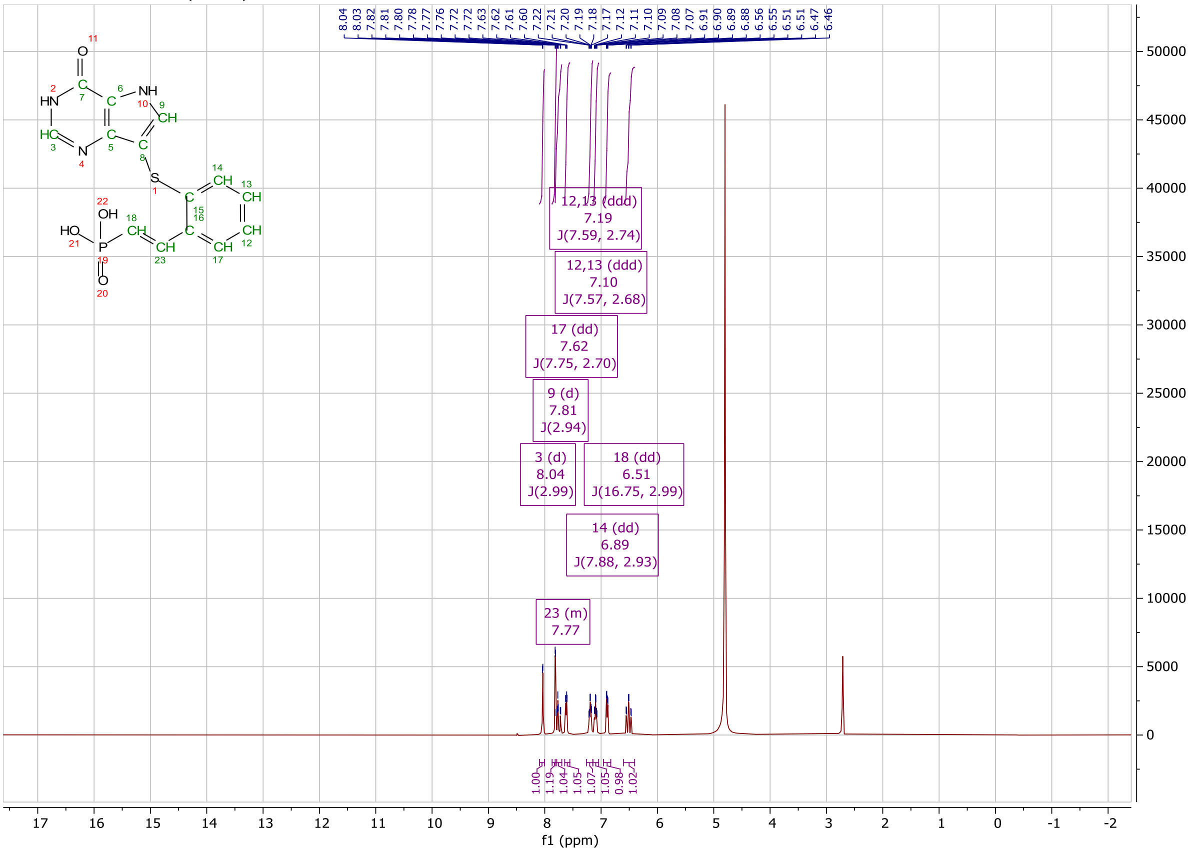

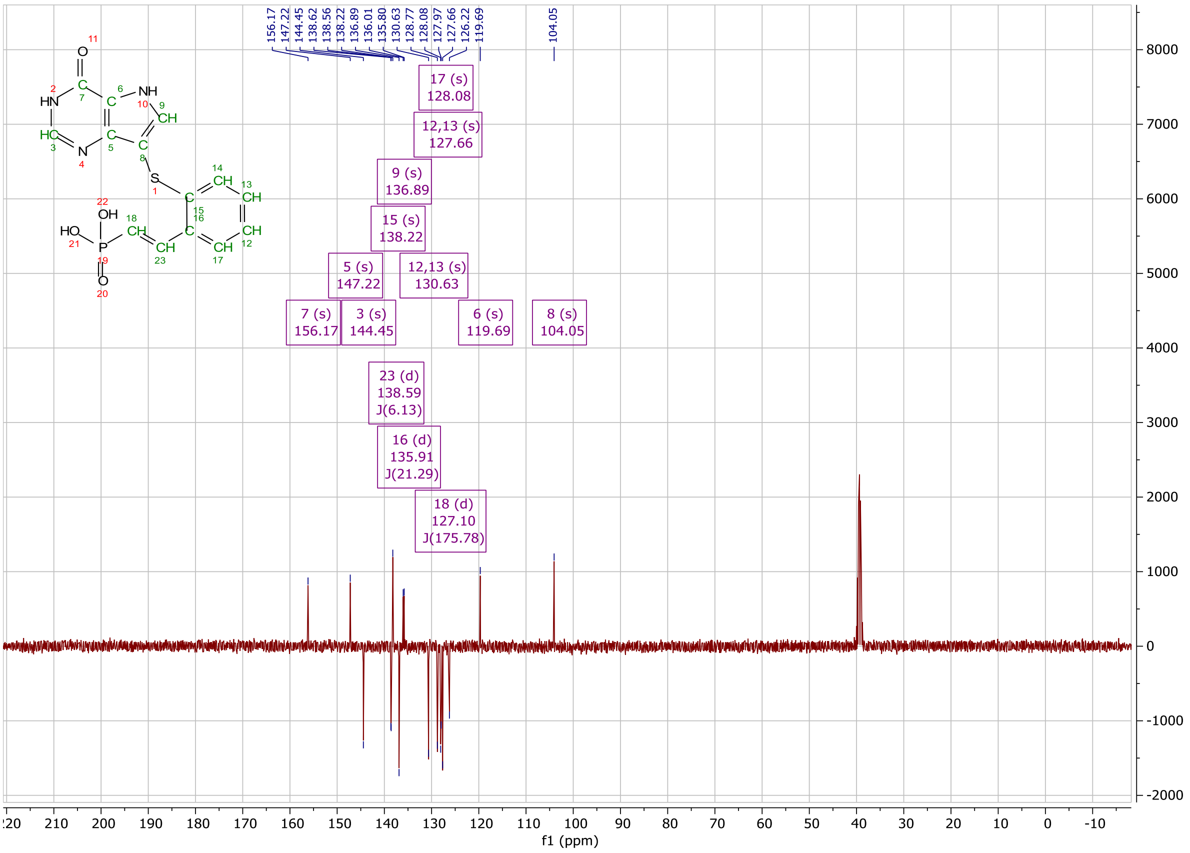

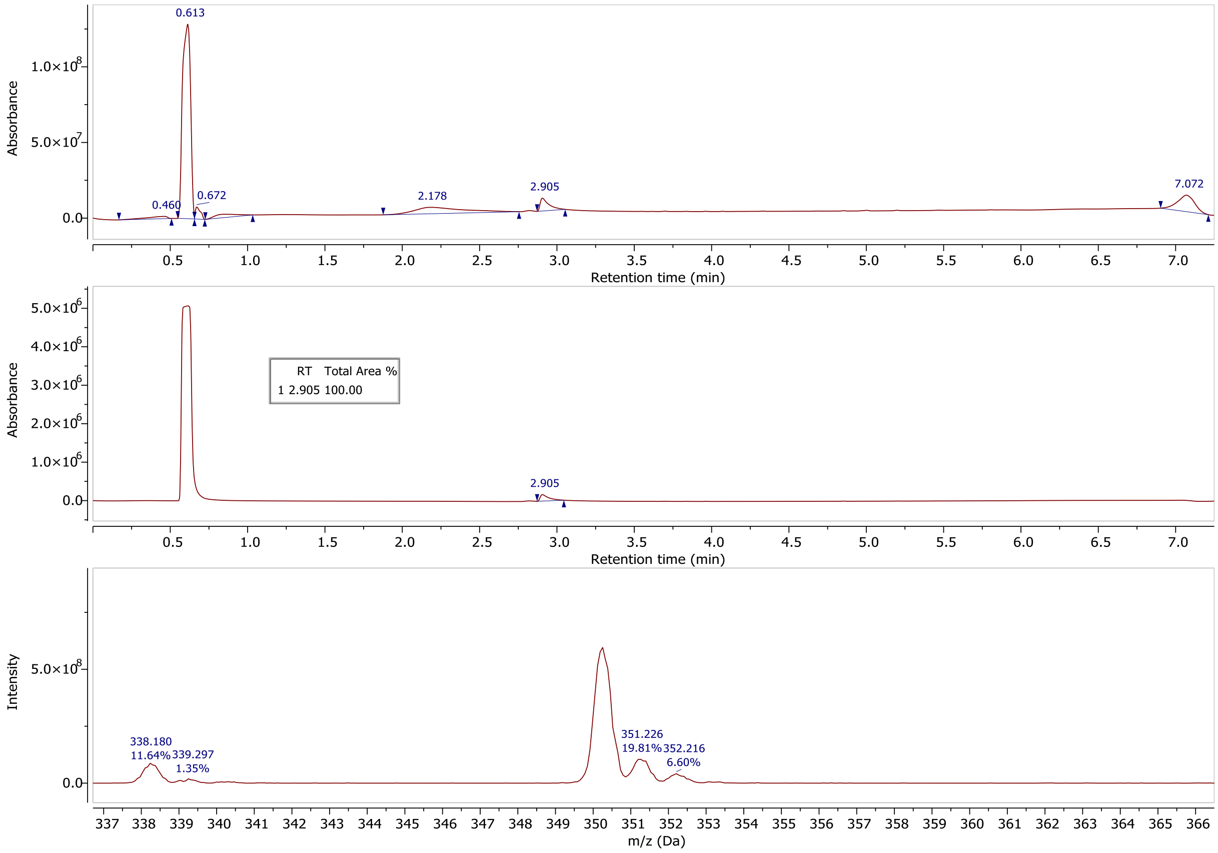

3.2.25 Sodium 7-((2-((phosphonato)methoxy)phenyl)thio)-3,5-dihydro-4*H*-pyrrolo[3,2-*d*]pyrimidin-4-one (18c)

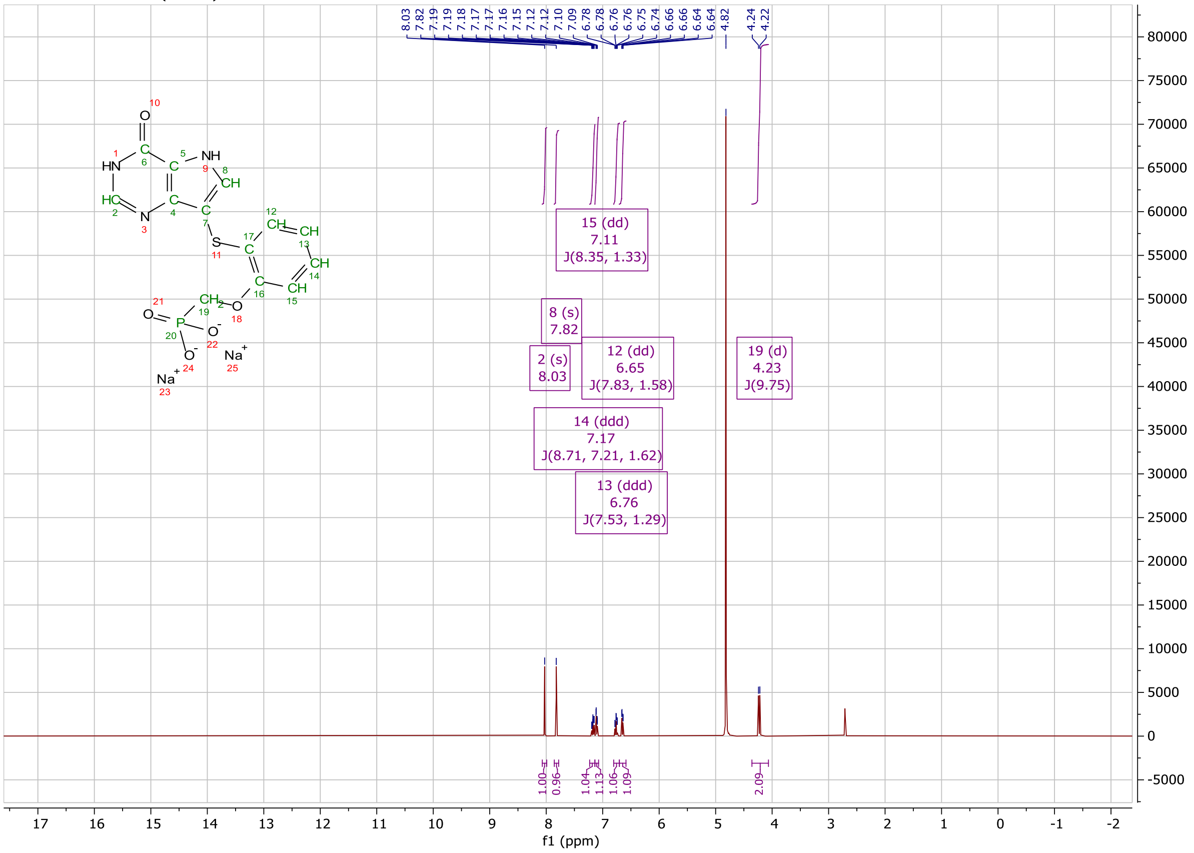

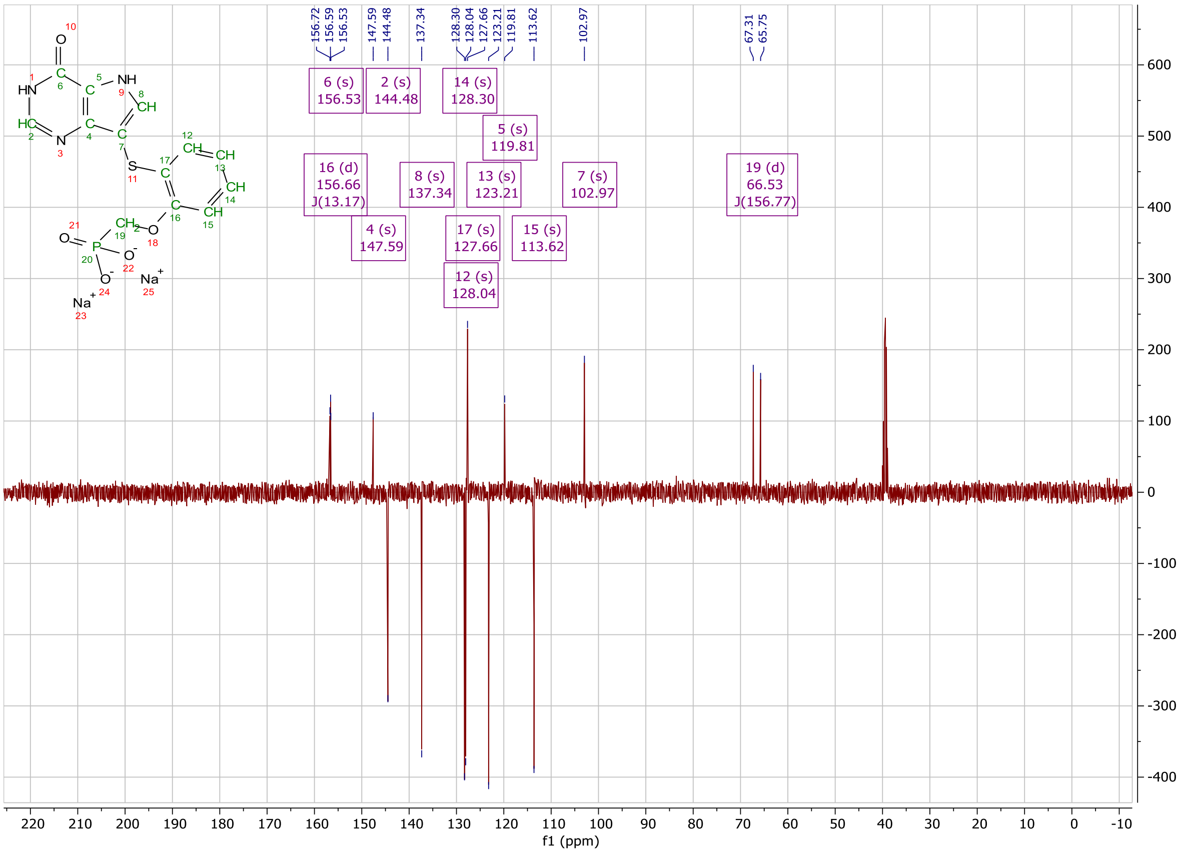
156.72
156.59
156.53
147.59
144.48
137.34
128.30
128.04
127.66
123.21
119.81
113.62
102.97
67.31
65.75
6 (s) 156.53
2 (s) 144.48
14 (s) 128.30
5 (s) 119.81
16 (d) 156.66 J(13.17)
8 (s) 137.34
13 (s) 123.21
7 (s) 102.97
19 (d) 66.53 J(156.77)
4 (s) 147.59
17 (s) 127.66
15 (s) 113.62
12 (s) 128.04
f1 (ppm)

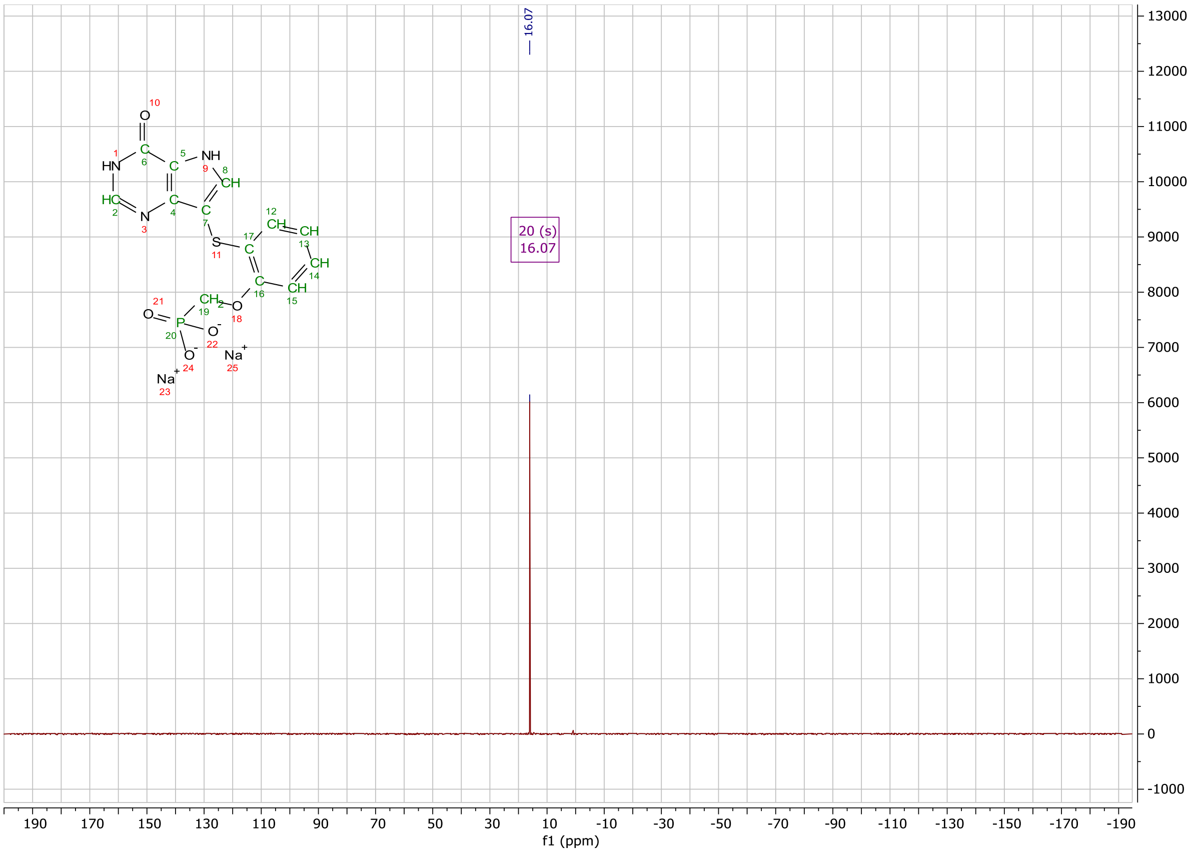
16.07
20 (s) 16.07
f1 (ppm)

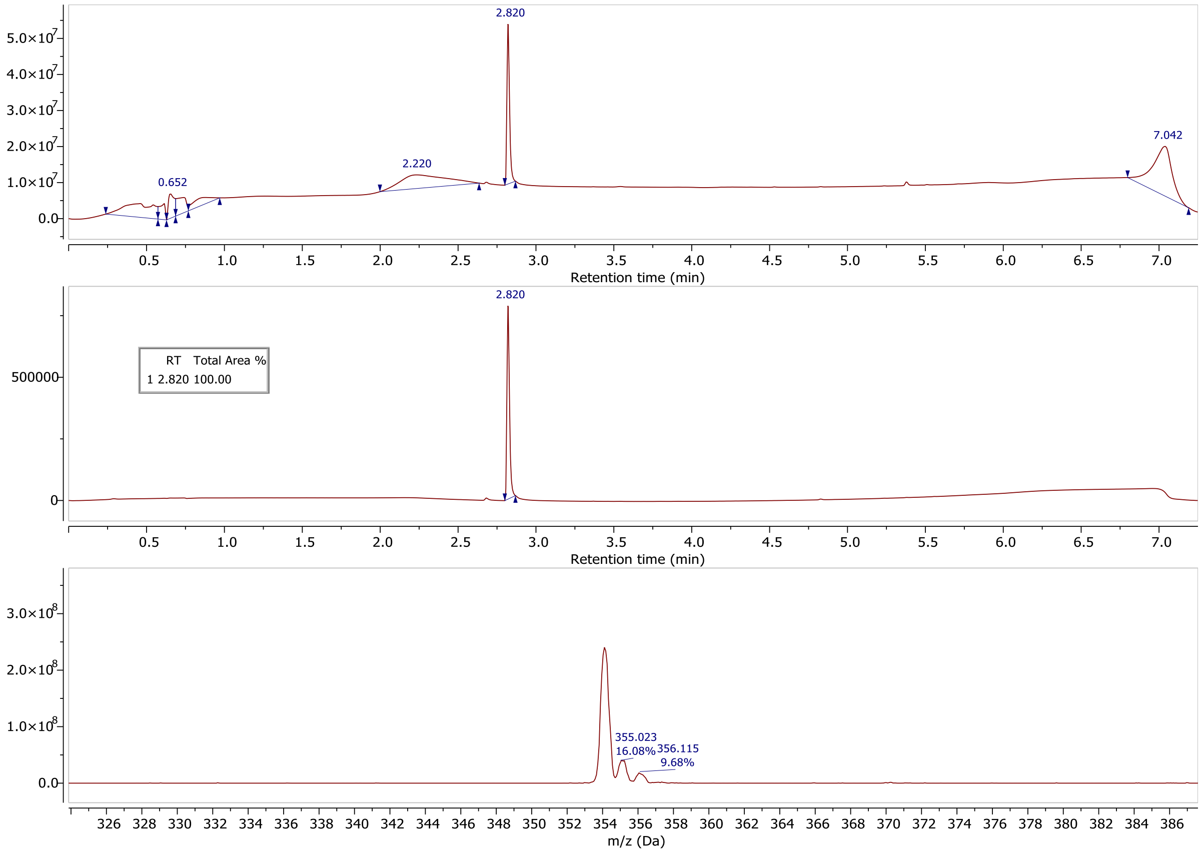

3.2.26 Sodium 7-((3-(phosphonato)phenyl)thio)-3,5-dihydro-4*H*-pyrrolo[3,2-*d*]pyrimidin-4-one (21)

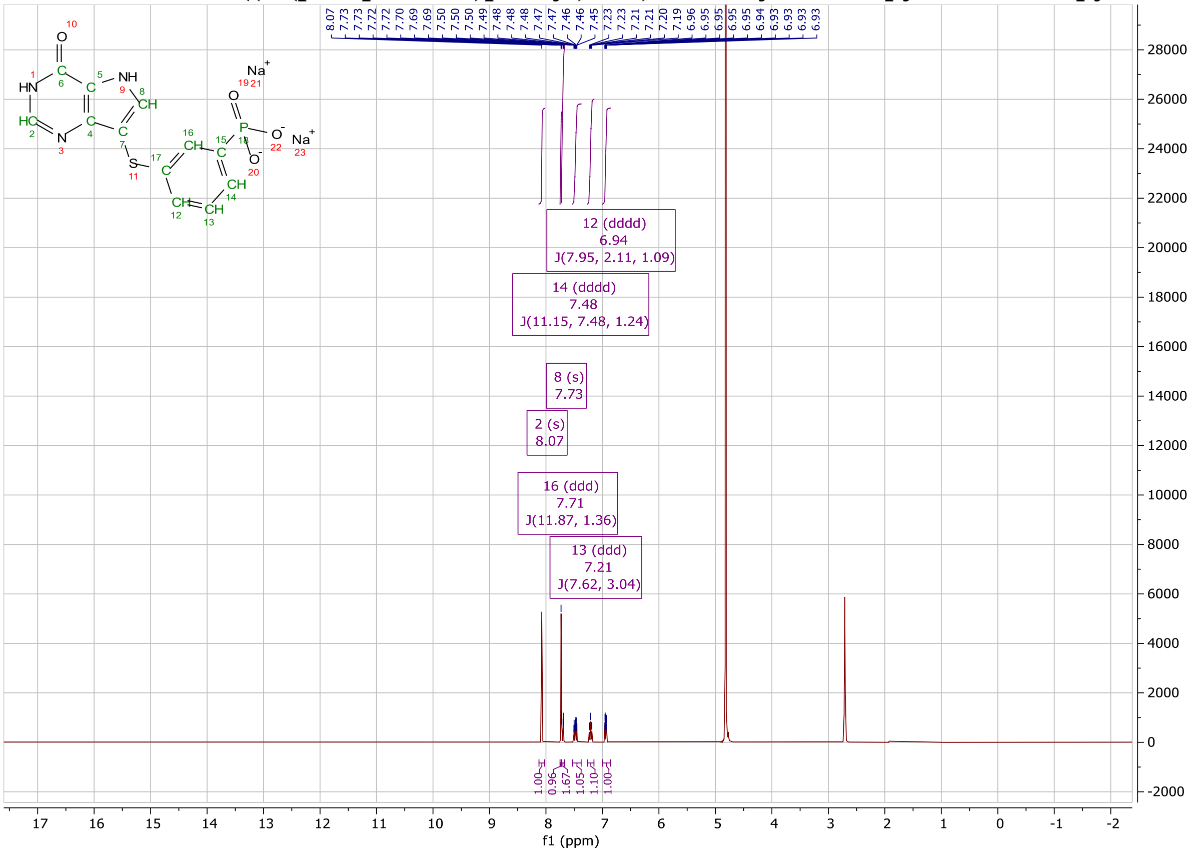

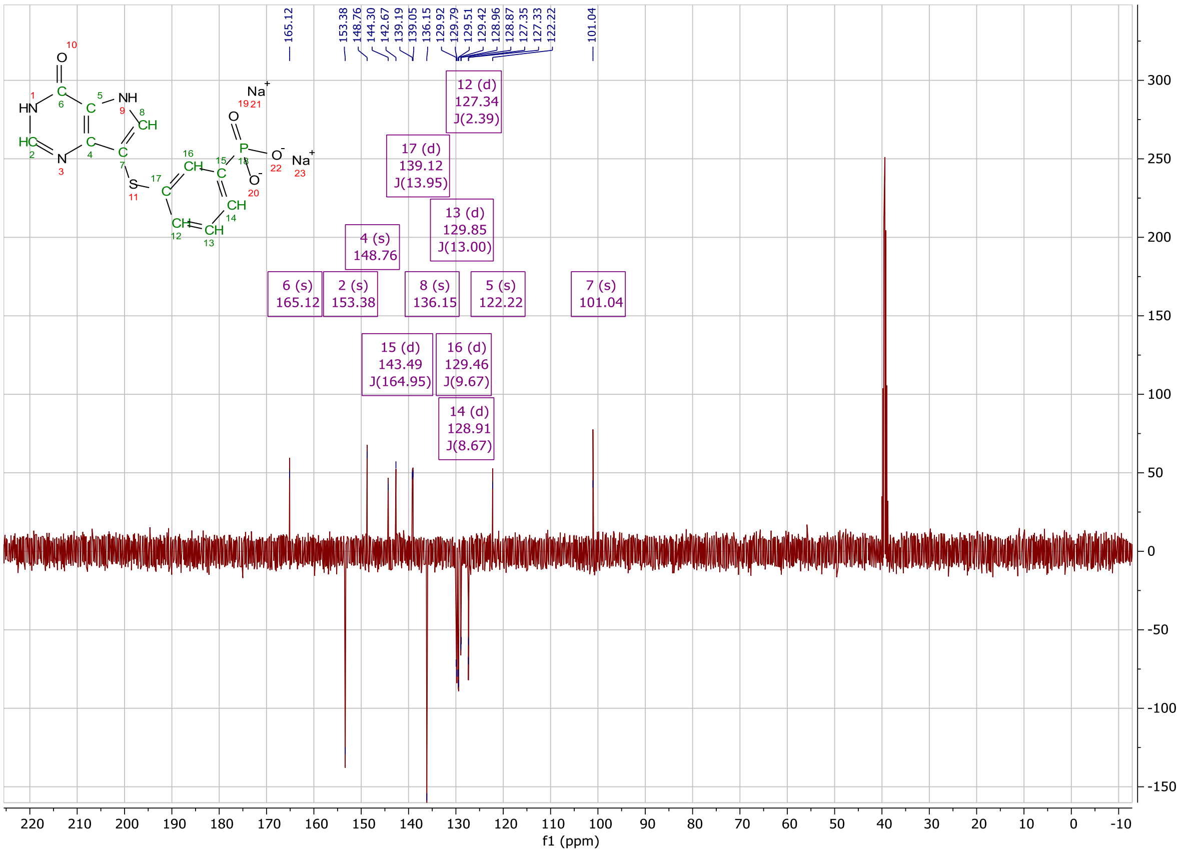
165.12
153.38
148.76
144.30
142.67
139.19
139.05
136.15
129.92
129.79
129.51
129.42
128.96
128.87
127.35
127.33
122.22
101.04
12 (d)
127.34
J(2.39)
17 (d)
139.12
J(13.95)
13 (d)
129.85
J(13.00)
4 (s)
148.76
6 (s)
165.12
2 (s)
153.38
8 (s)
136.15
5 (s)
122.22
7 (s)
101.04
15 (d)
143.49
J(164.95)
16 (d)
129.46
J(9.67)
14 (d)
128.91
J(8.67)
f1 (ppm)

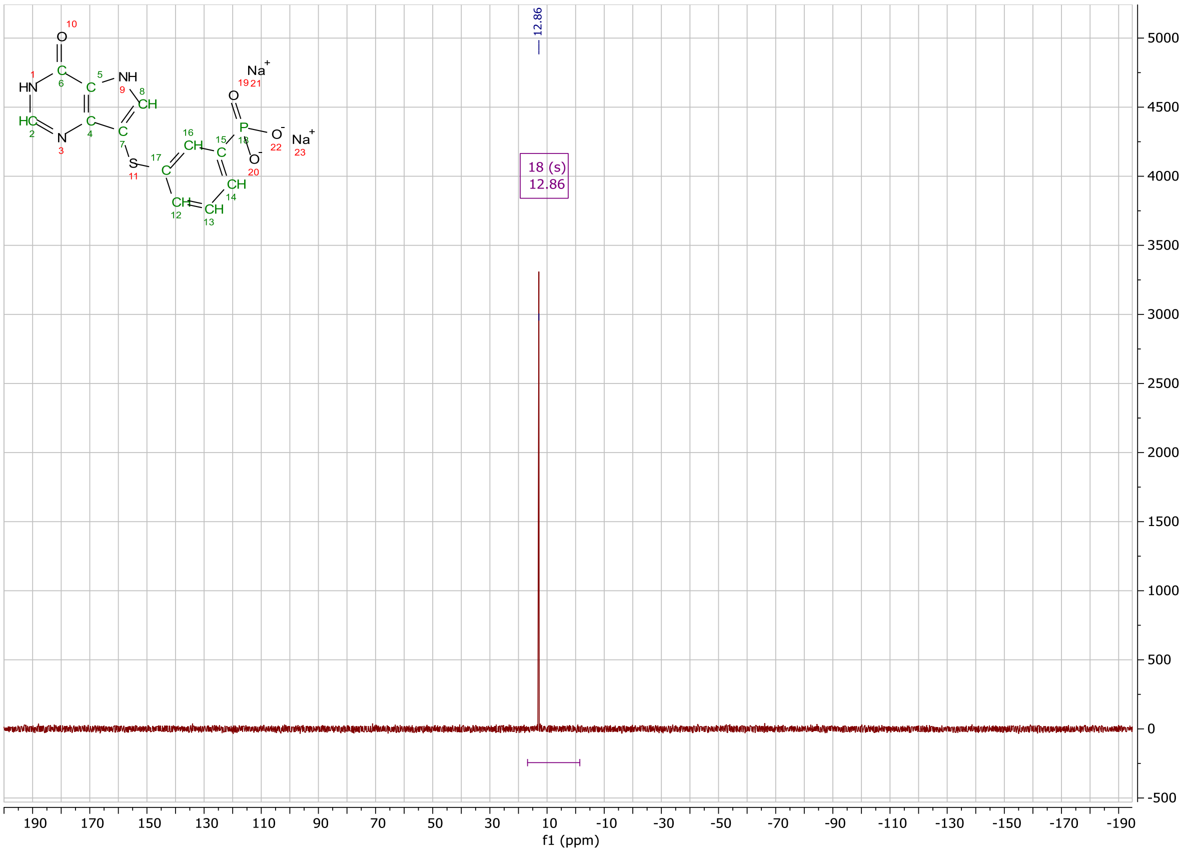
12.86
18 (s)
12.86
f1 (ppm)

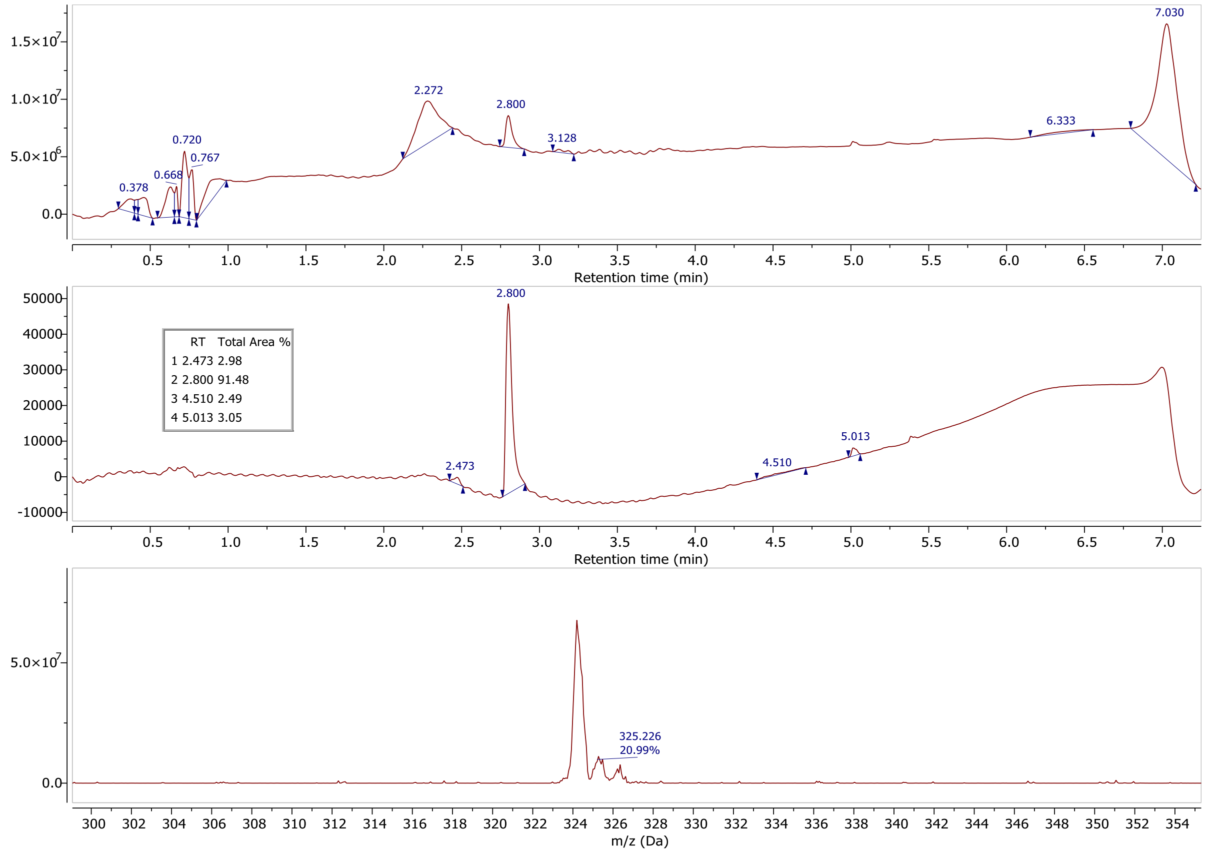

3.2.27 Sodium 7-((2-(3-phosphonato-2-oxapropyl)phenyl)thio)-3,5-dihydro-4*H*-pyrrolo[3,2-*d*]pyrimidin-4-one (23)

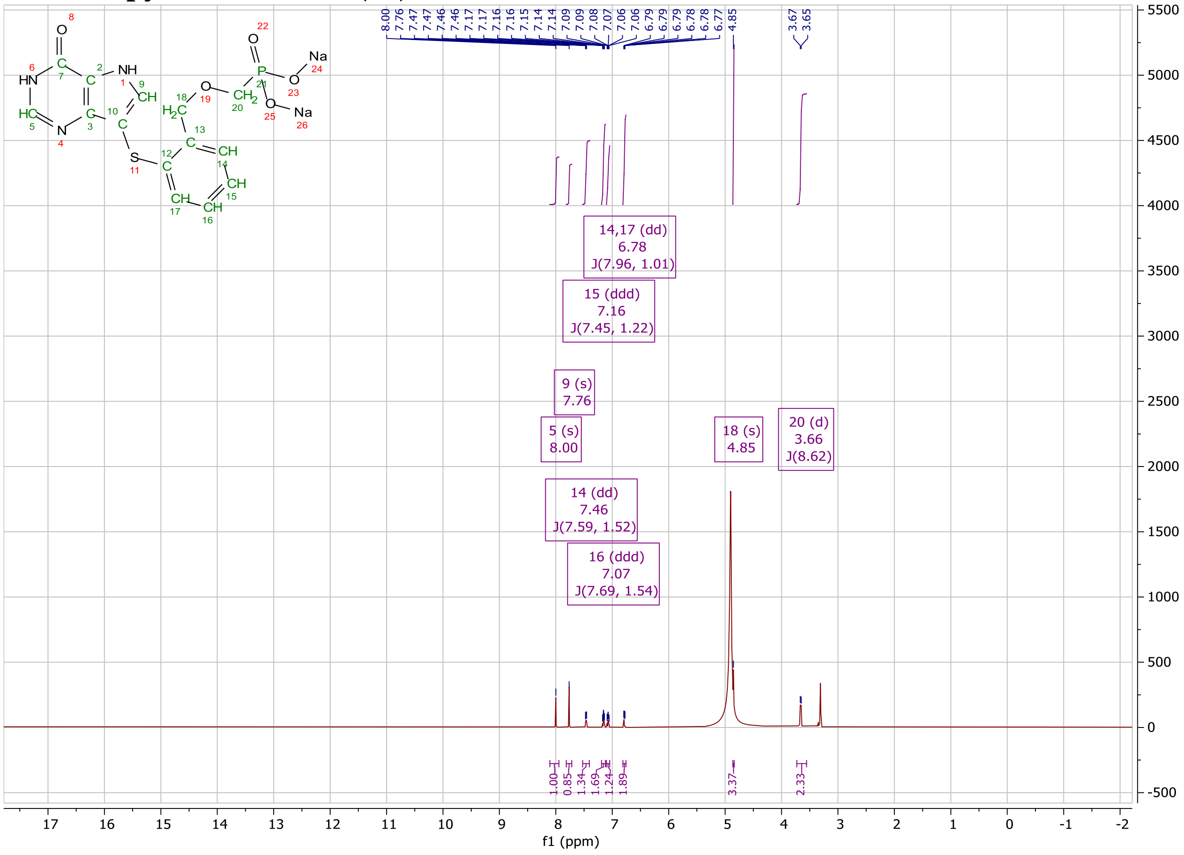

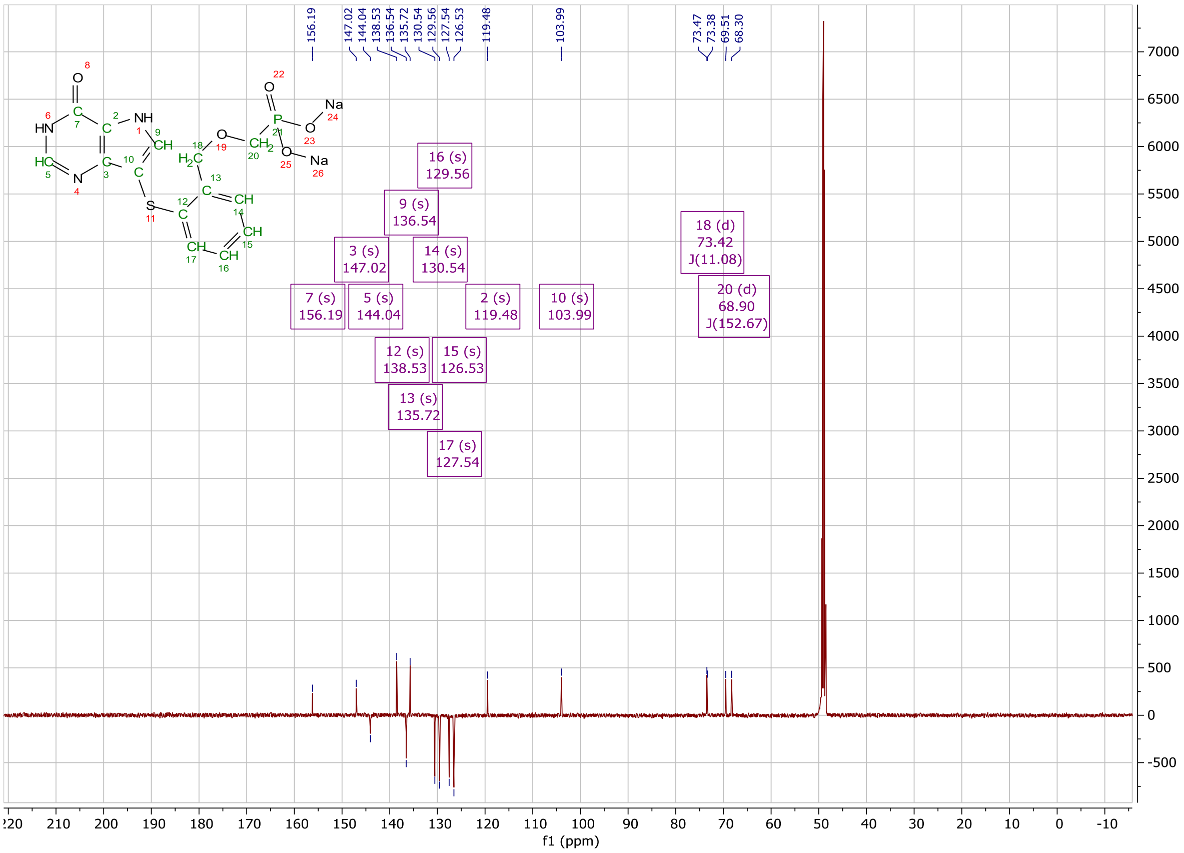
156.19
147.02
144.04
138.53
136.54
135.72
130.54
129.56
127.54
126.53
119.48
103.99
73.47
73.38
69.51
68.30
16 (s) 129.56
9 (s) 136.54
3 (s) 147.02
14 (s) 130.54
7 (s) 156.19
5 (s) 144.04
2 (s) 119.48
10 (s) 103.99
12 (s) 138.53
15 (s) 126.53
13 (s) 135.72
17 (s) 127.54
18 (d) 73.42 J(11.08)
20 (d) 68.90 J(152.67)
f1 (ppm)

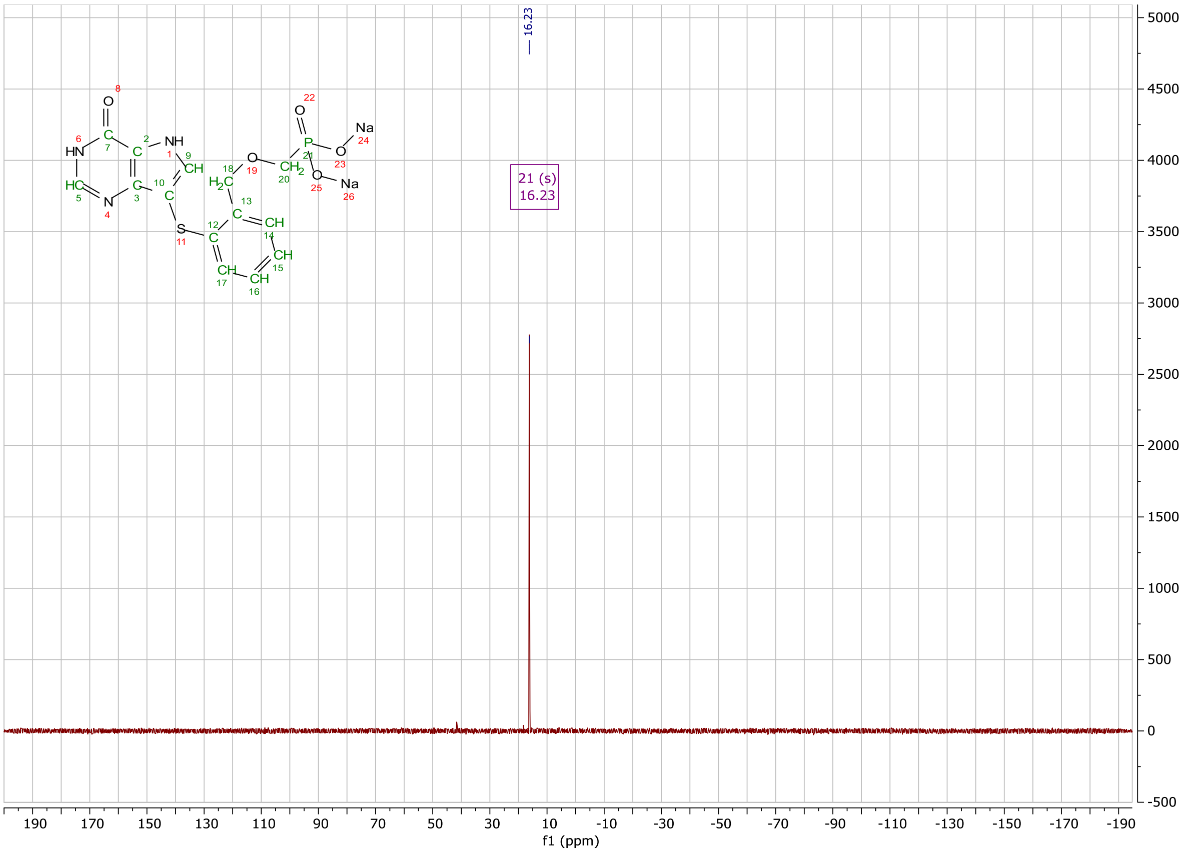
16.23
21 (s) 16.23
f1 (ppm)

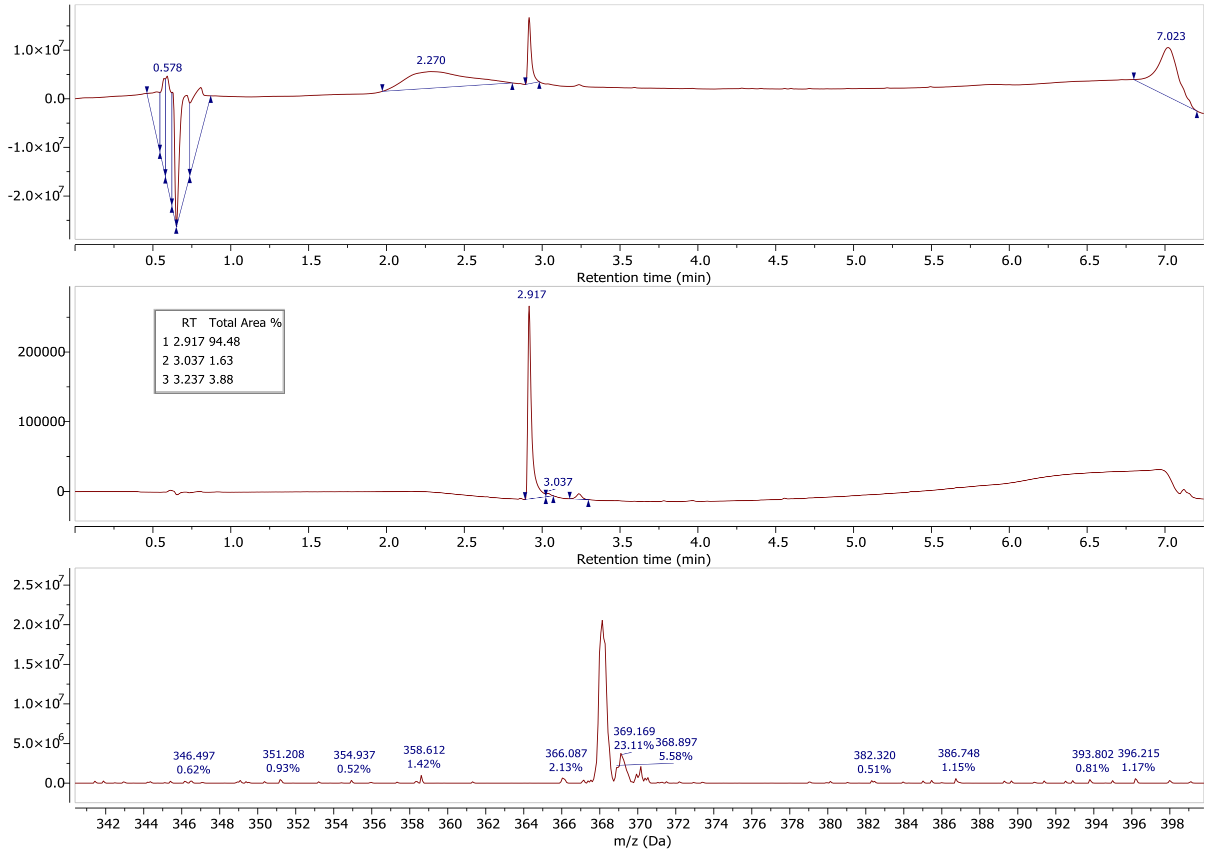

3.2.28 (5-Bromo-2-iodophenyl)methanol (25)

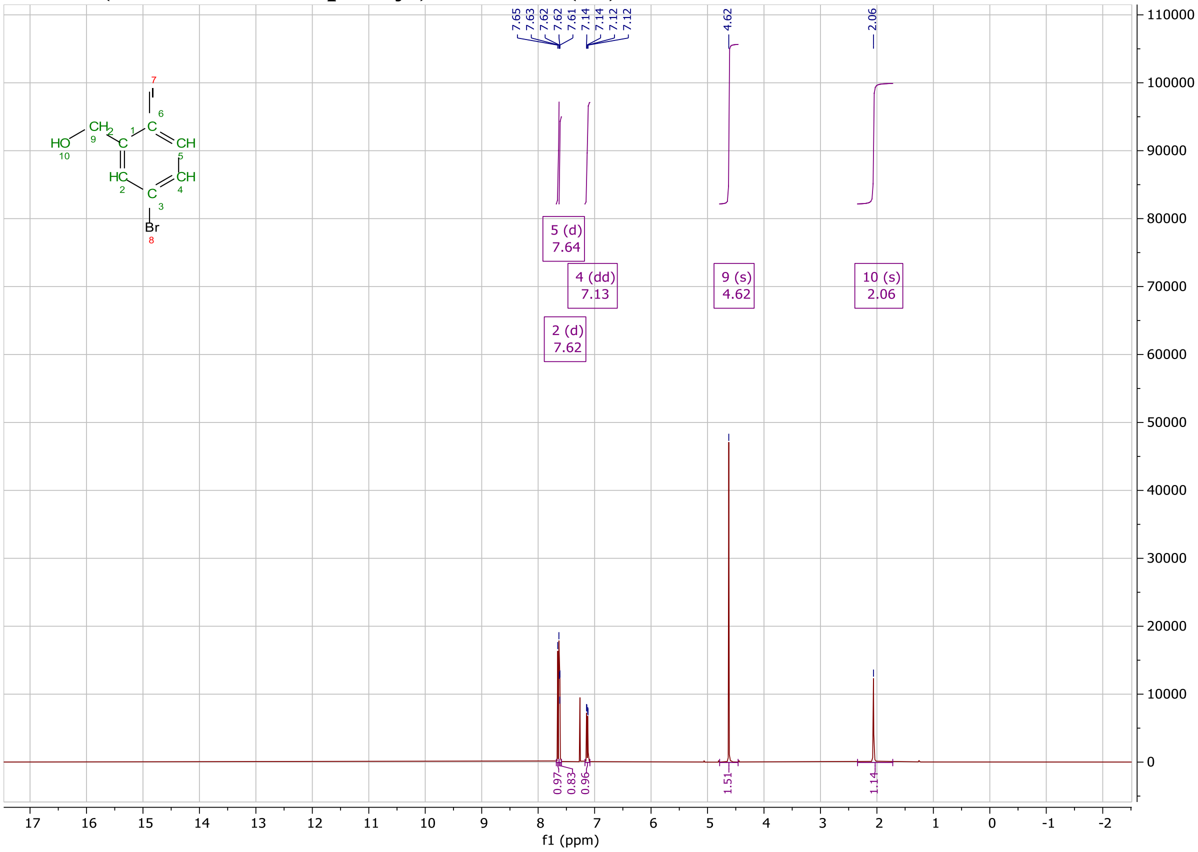

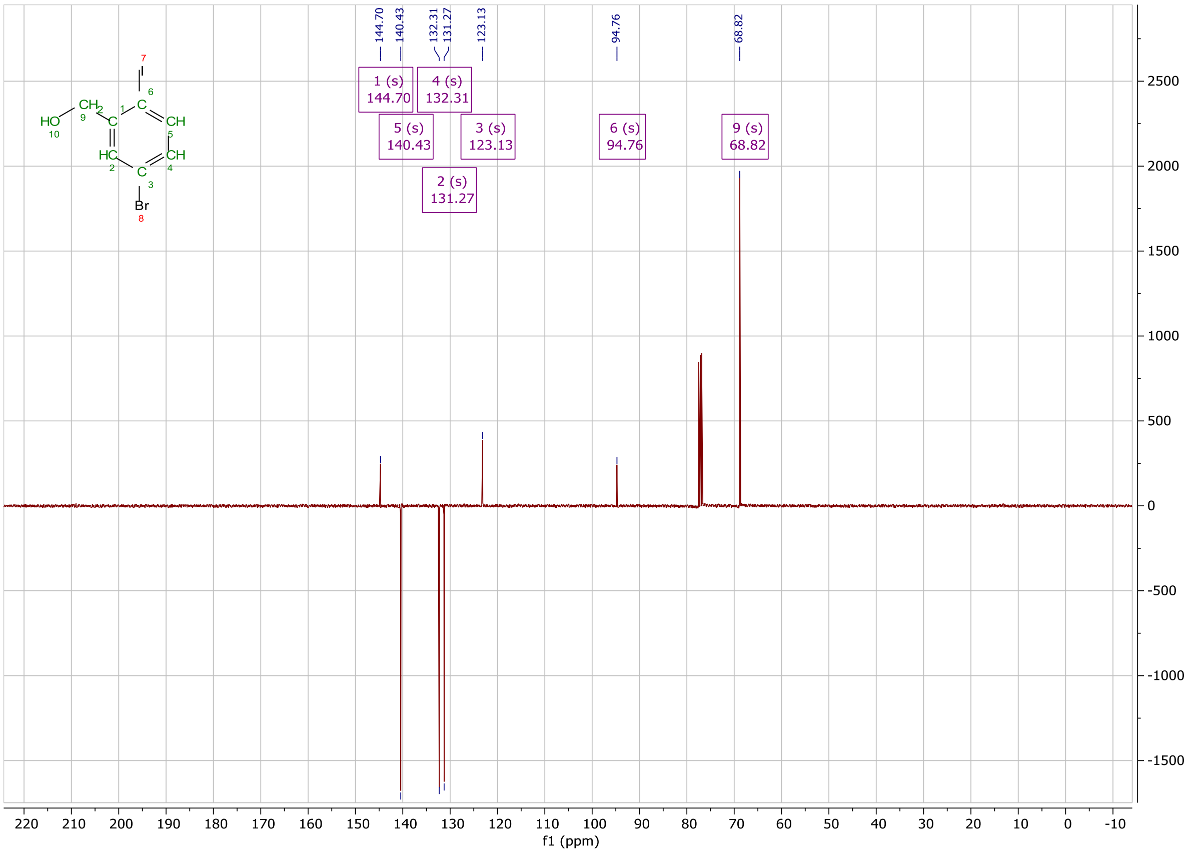
144.70
140.43
132.31
131.27
123.13
94.76
68.82
1 (s) 144.70
4 (s) 132.31
5 (s) 140.43
3 (s) 123.13
6 (s) 94.76
9 (s) 68.82
2 (s) 131.27
f1 (ppm)

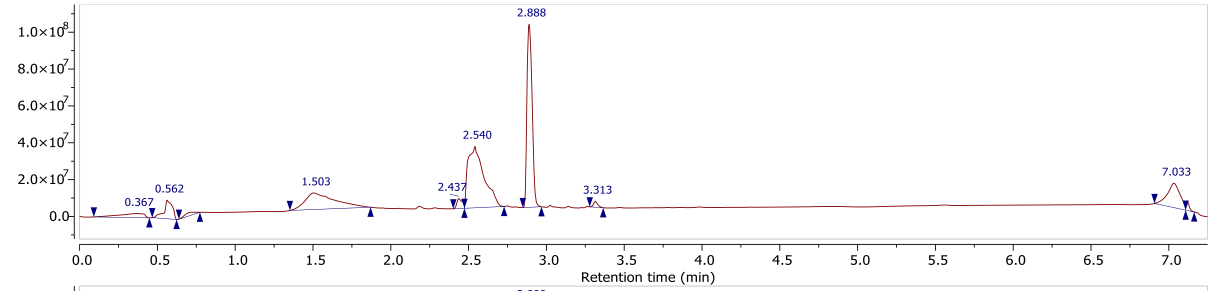
2.888
2.540
1.503
0.562
0.367
2.437
3.313
7.033
Retention time (min)

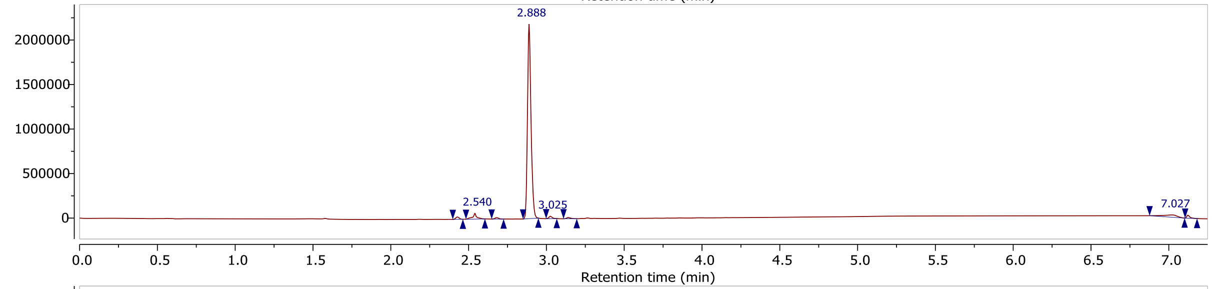
2.888
2.540
3.025
7.027
Retention time (min)

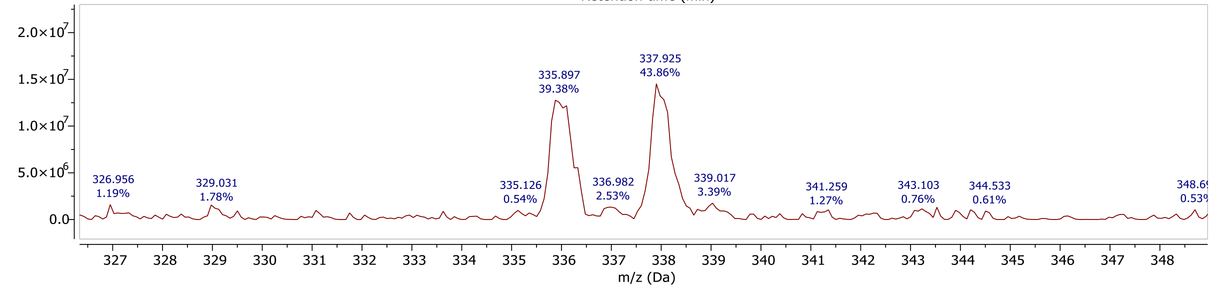
337.925 43.86%
335.897 39.38%
326.956 1.19%
329.031 1.78%
335.126 0.54%
336.982 2.53%
339.017 3.39%
341.259 1.27%
343.103 0.76%
344.533 0.61%
m/z (Da)

3.2.29 5-Bromo-2-iodobenzaldehyde (26)

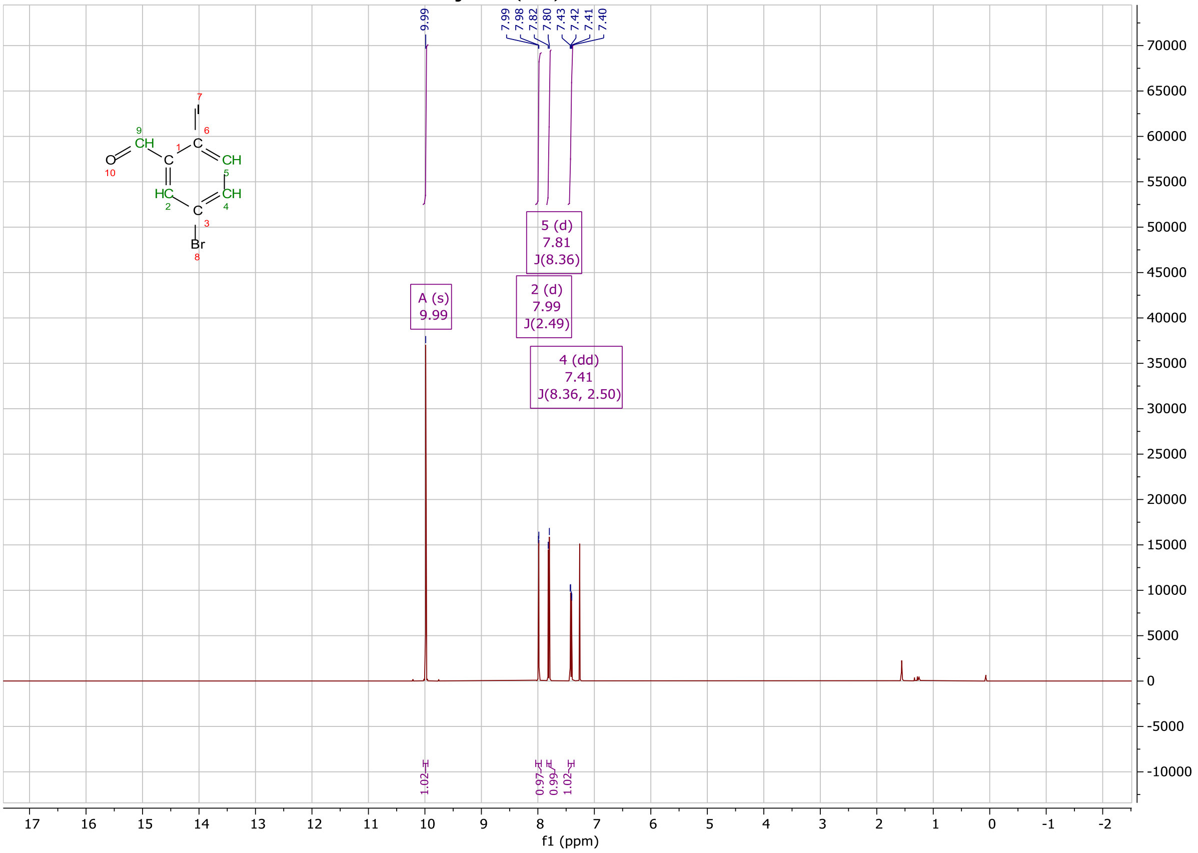

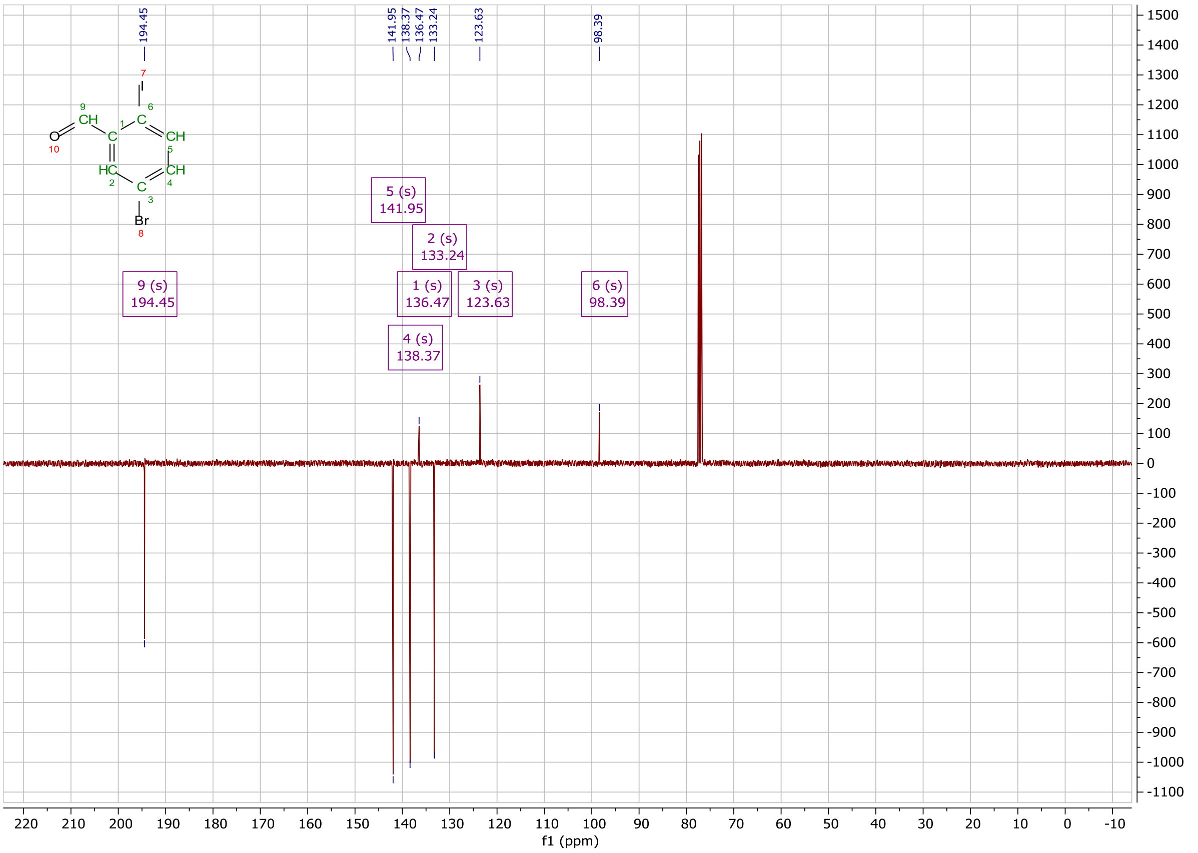

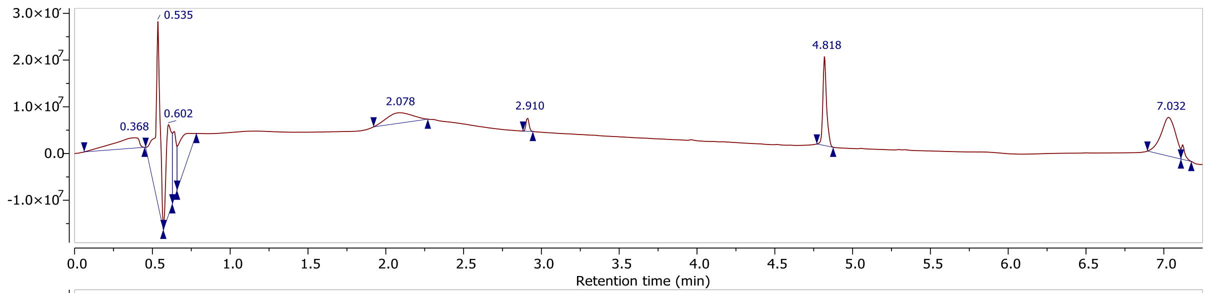

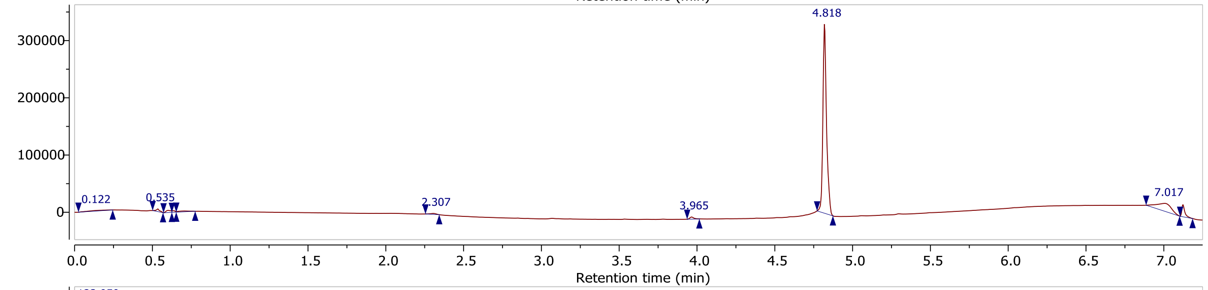

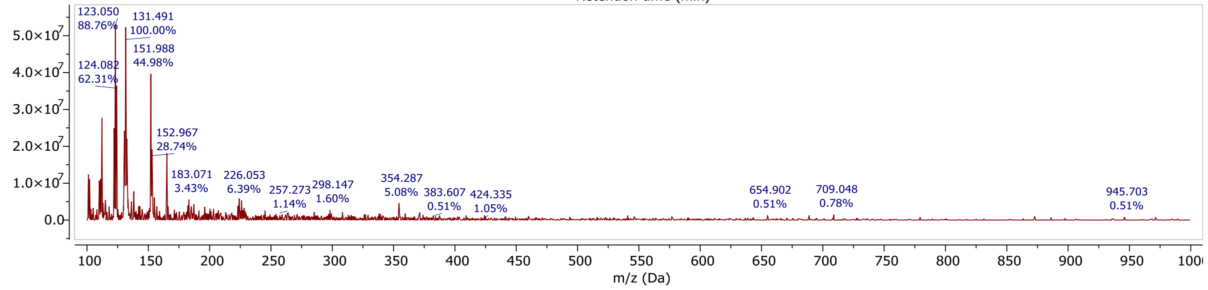

3.2.30 Diethyl (*E*)-(5-bromo-2-iodostyryl)phosphonate (27)

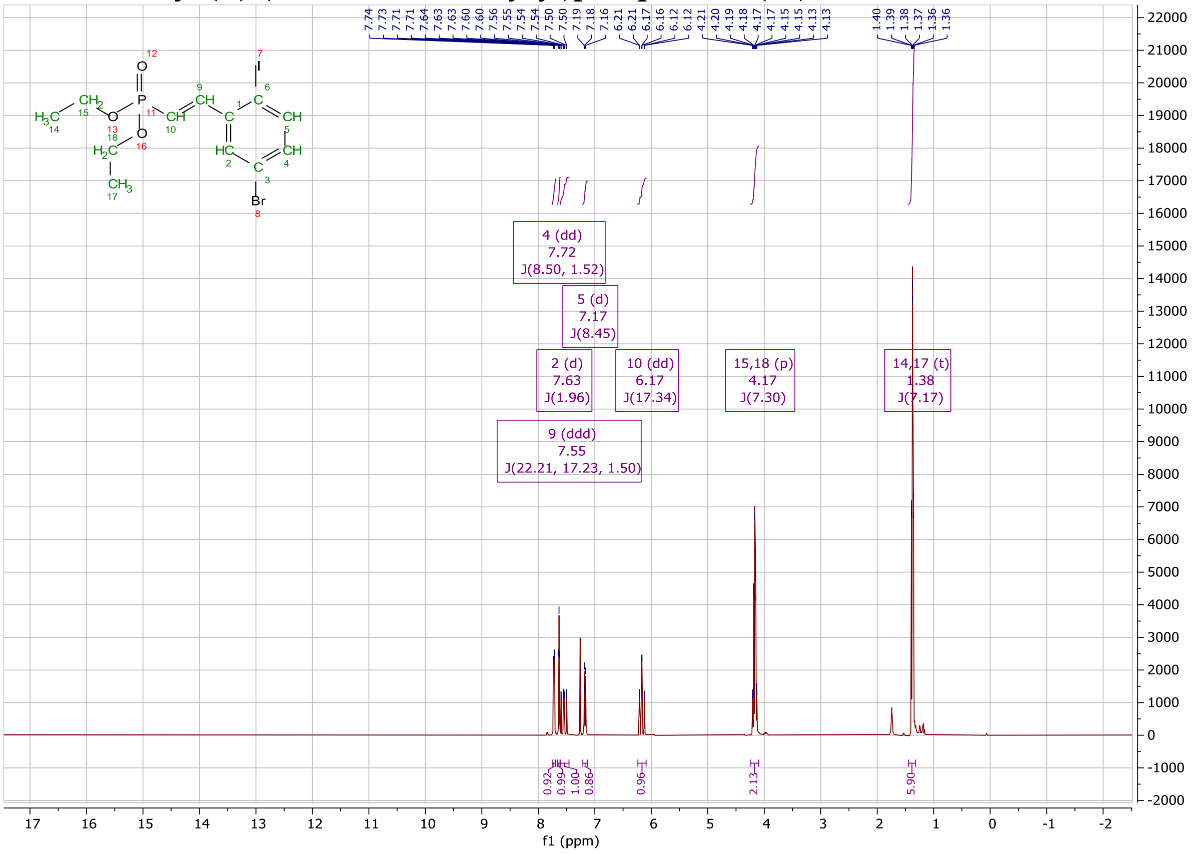

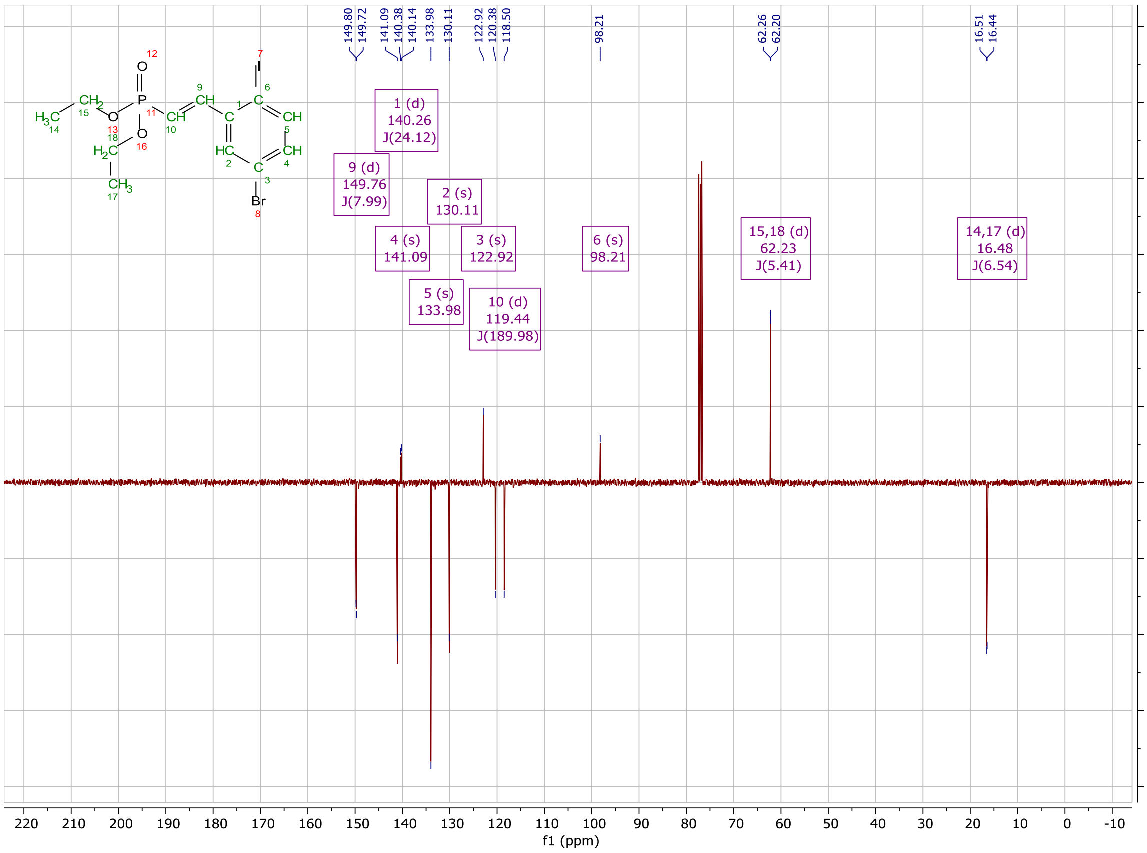
149.80
149.72
141.09
140.38
140.14
133.98
130.11
122.92
120.38
118.50
98.21
62.26
62.20
16.51
16.44
1 (d) 140.26 J(24.12)
9 (d) 149.76 J(7.99)
2 (s) 130.11
4 (s) 141.09
3 (s) 122.92
6 (s) 98.21
15,18 (d) 62.23 J(5.41)
14,17 (d) 16.48 J(6.54)
5 (s) 133.98
10 (d) 119.44 J(189.98)
f1 (ppm)

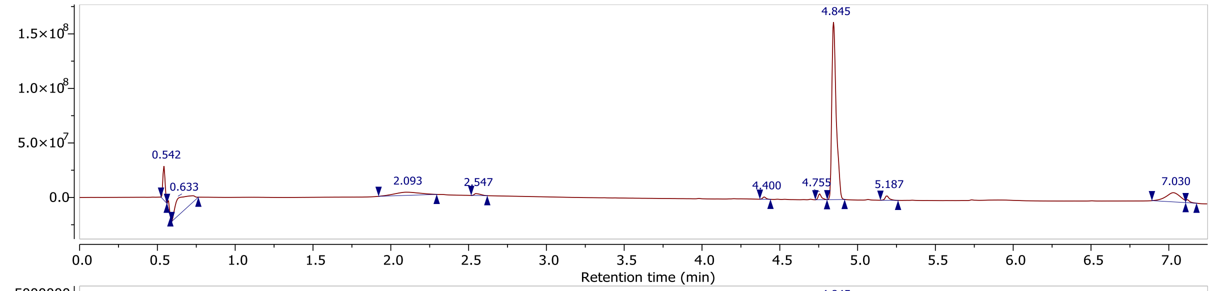
4.845
0.542
0.633
2.093
2.547
4.400
4.755
5.187
7.030
Retention time (min)

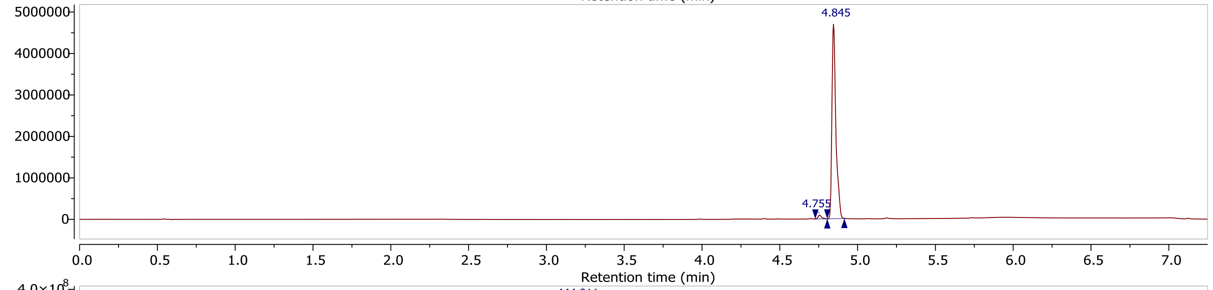
4.845
4.755
Retention time (min)

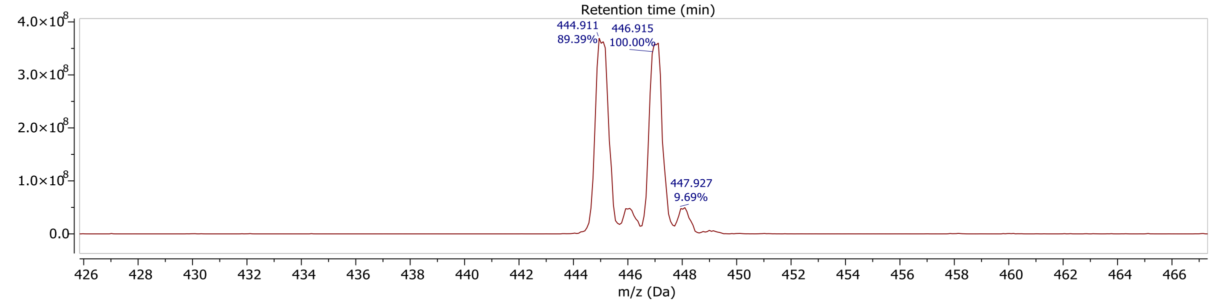
444.911
89.39%
446.915
100.00%
447.927
9.69%
m/z (Da)

3.2.31 4-Bromo-1-(bromomethyl)-2-iodobenzene (29)

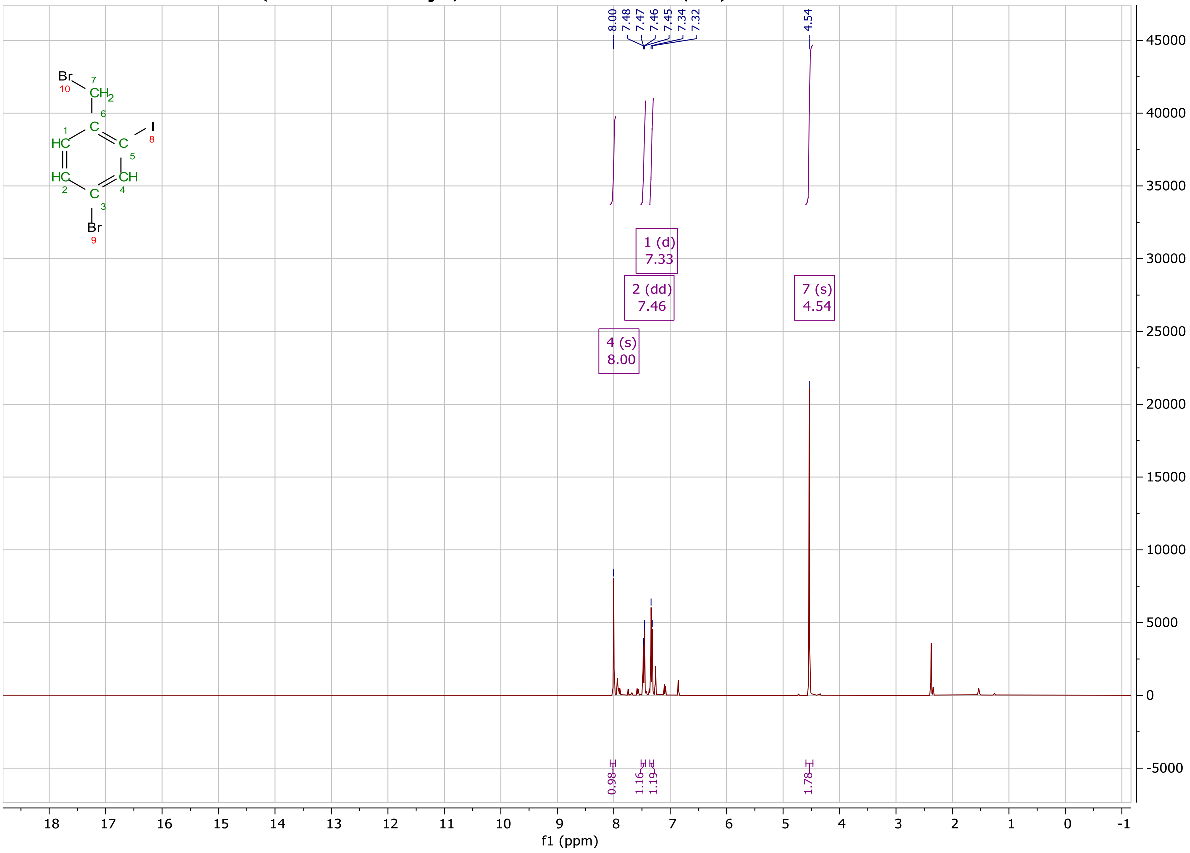

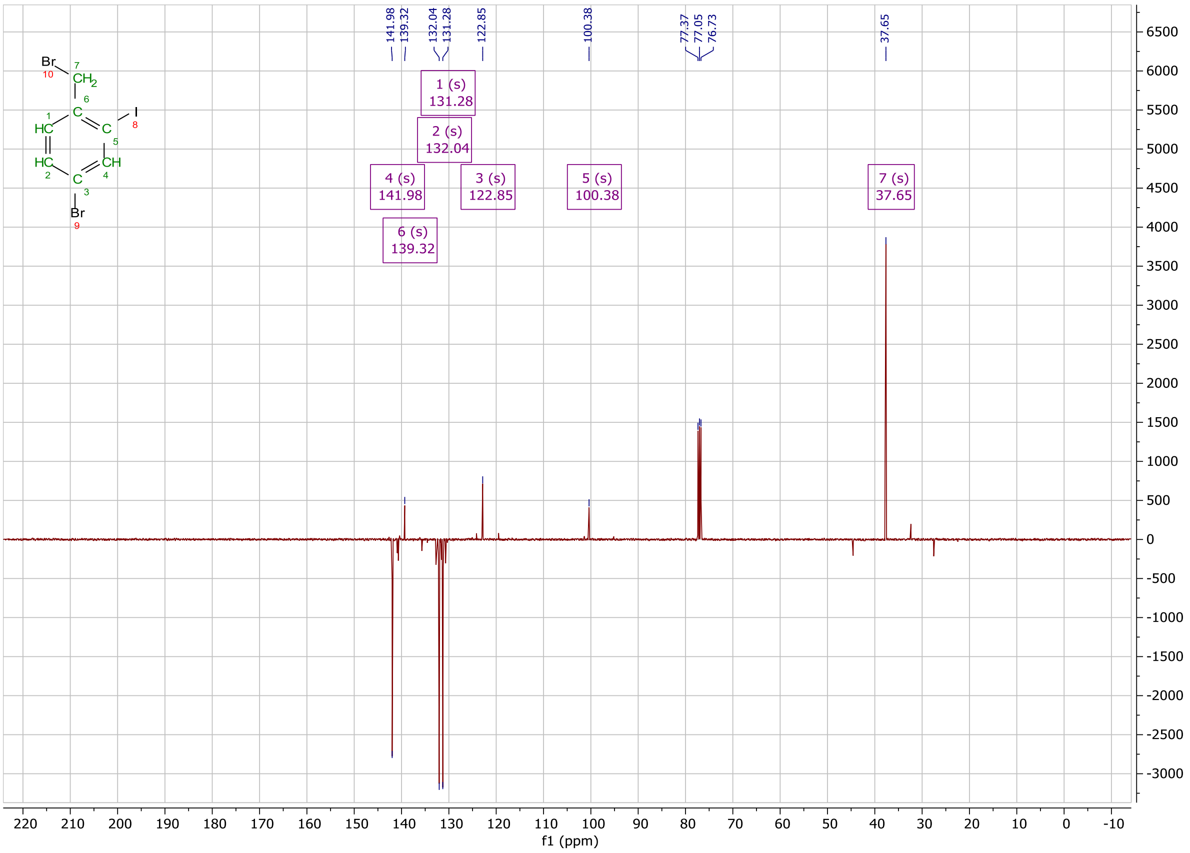

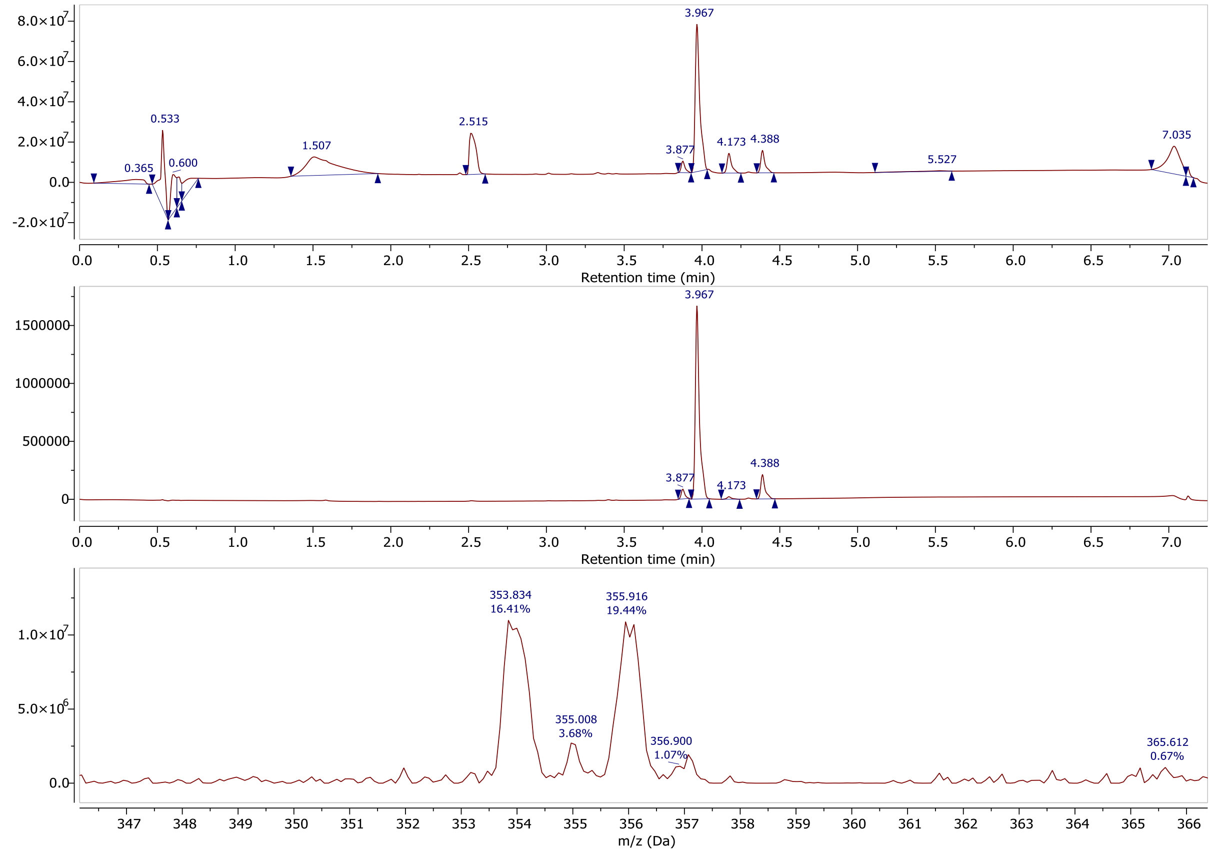

3.2.32 4-Bromo-2-iodobenzaldehyde (30)

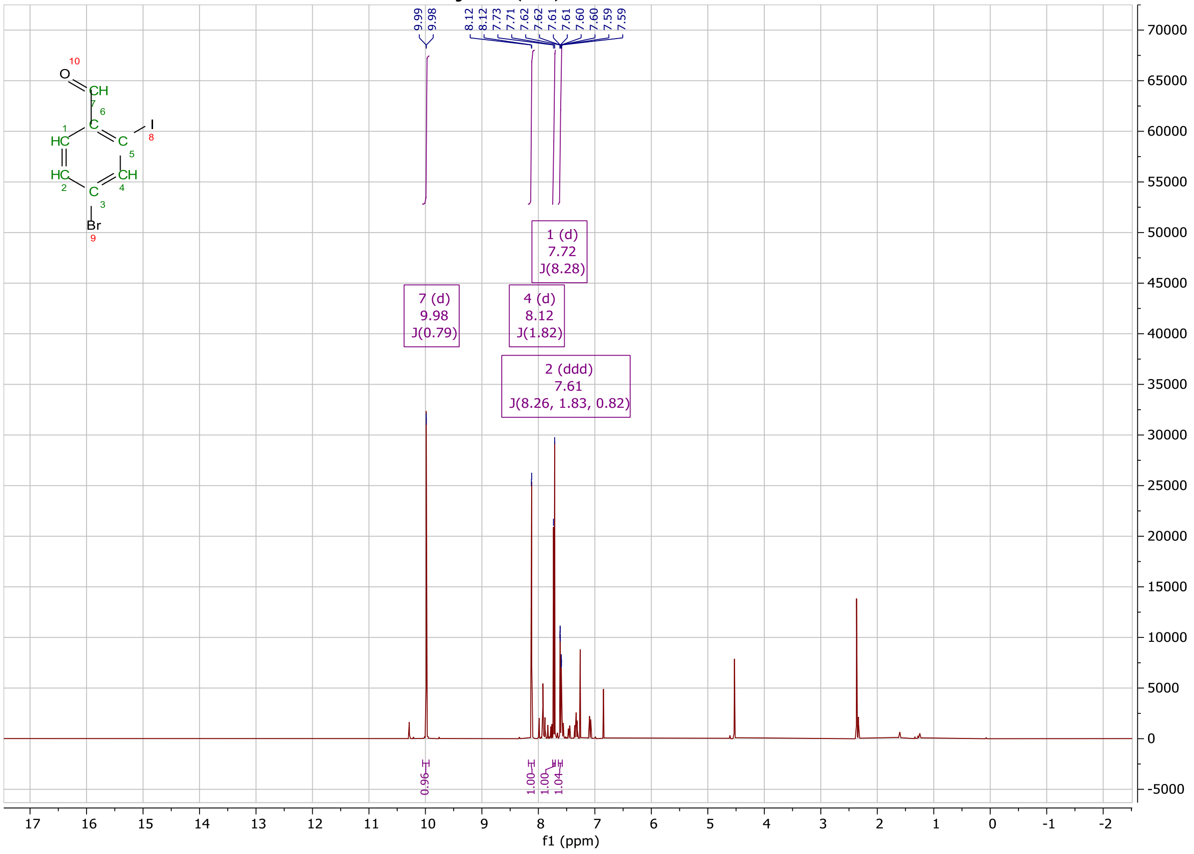

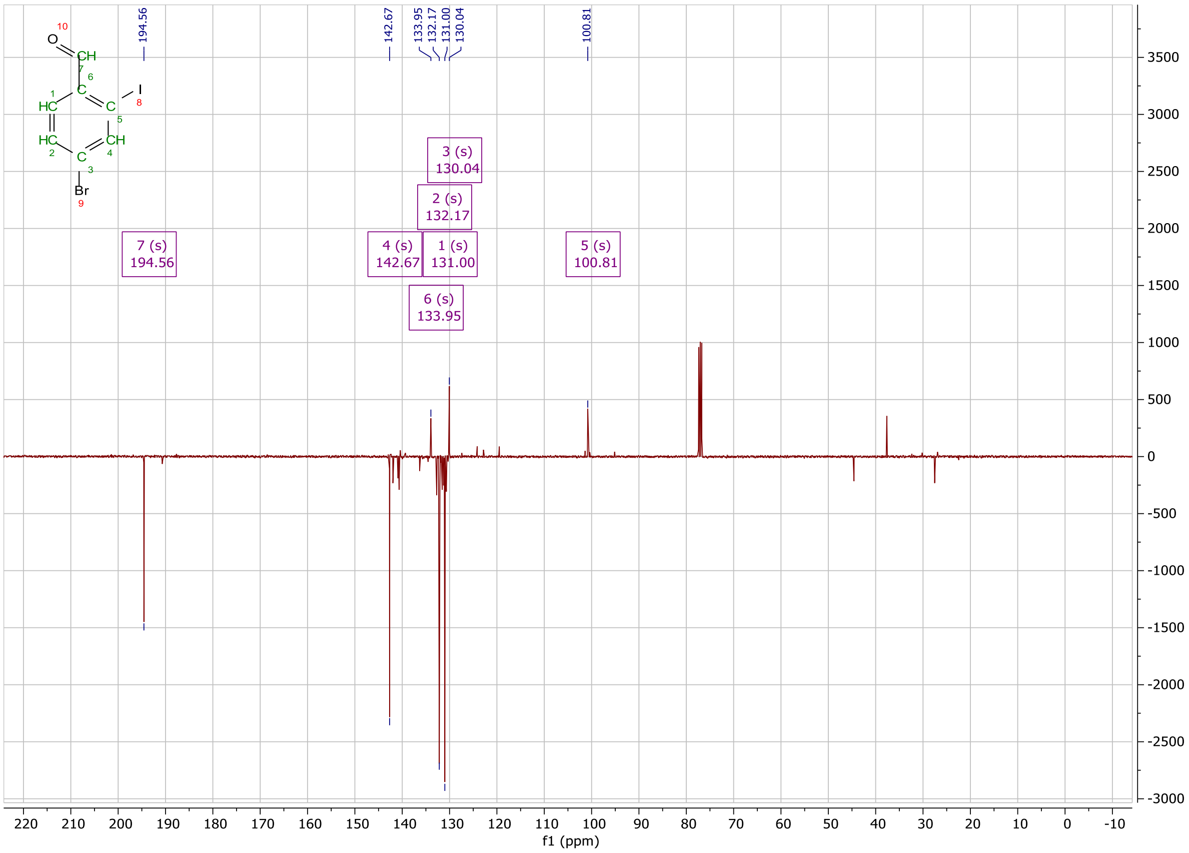
194.56
142.67
133.95
132.17
131.00
130.04
100.81
O
CH
I
HC
C
HC
CH
Br
3 (s)
130.04
2 (s)
132.17
7 (s)
194.56
4 (s)
142.67
1 (s)
131.00
5 (s)
100.81
6 (s)
133.95
f1 (ppm)

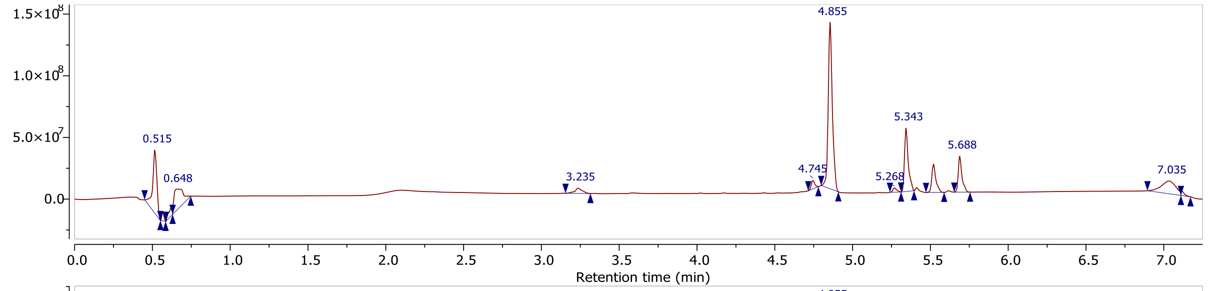
4.855
5.343
5.688
0.515
0.648
3.235
4.745
5.268
7.035
Retention time (min)

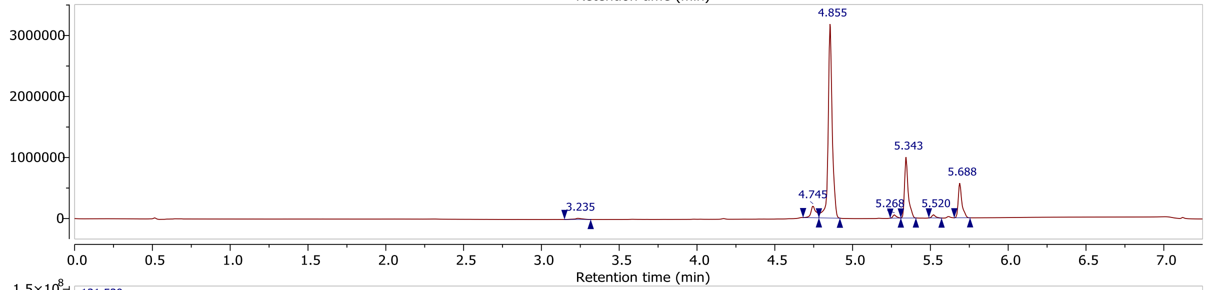
4.855
5.343
5.688
3.235
4.745
5.268
5.520
Retention time (min)

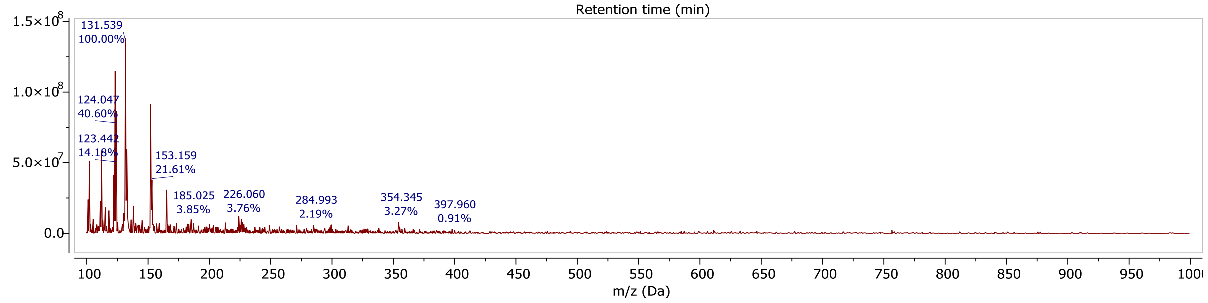
131.539
100.00%
124.047
40.60%
123.442
14.18%
153.159
21.61%
185.025
3.85%
226.060
3.76%
284.993
2.19%
354.345
3.27%
397.960
0.91%
m/z (Da)

3.2.33 Diethyl (*E*)-(4-bromo-2-iodostyryl)phosphonate (31)

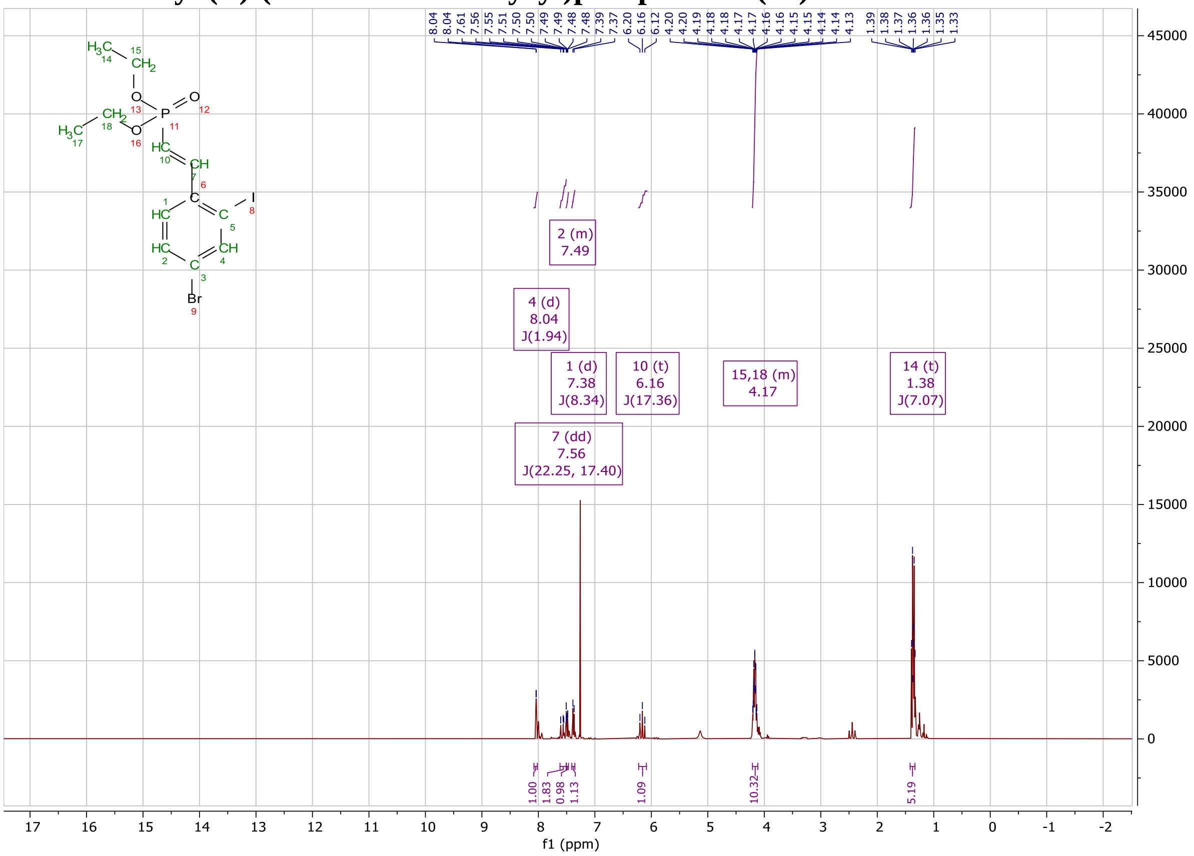

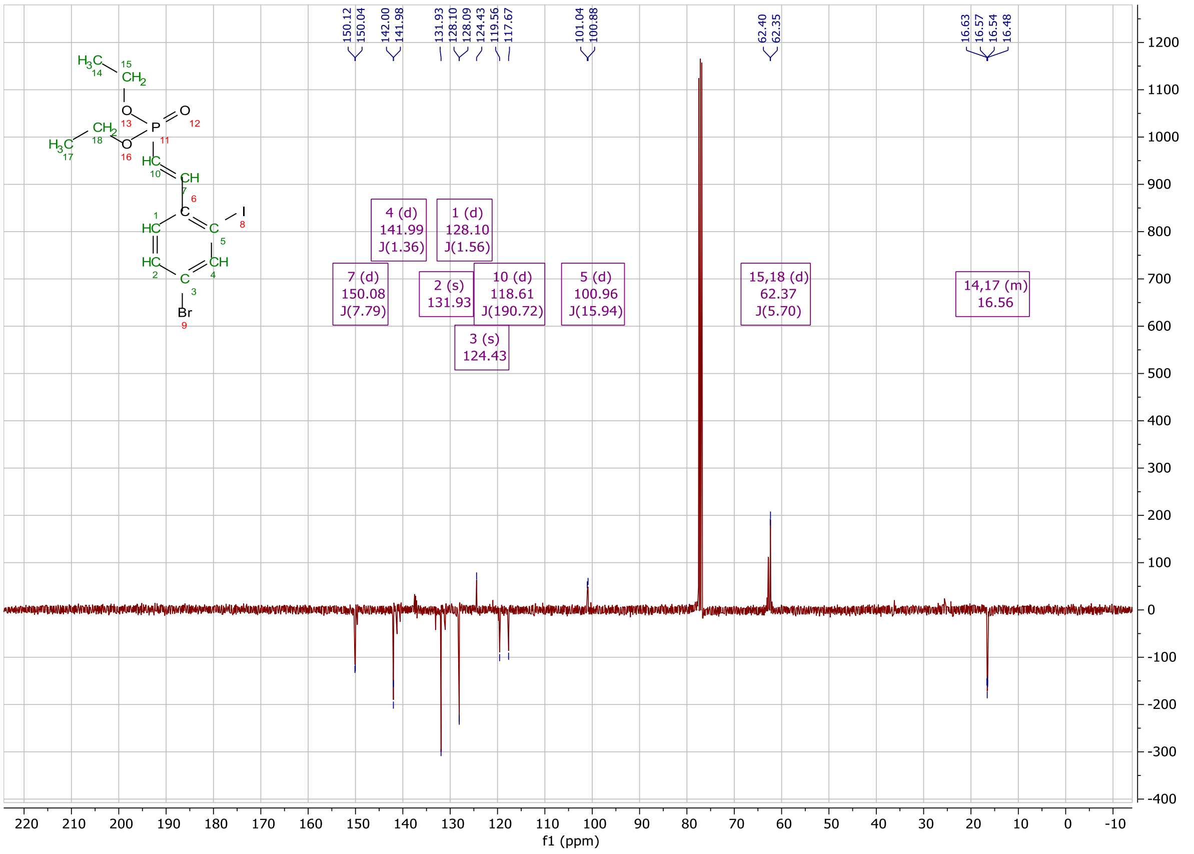

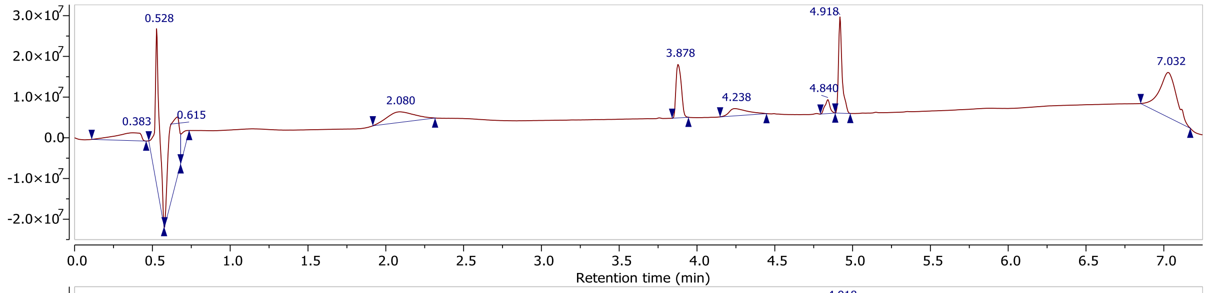

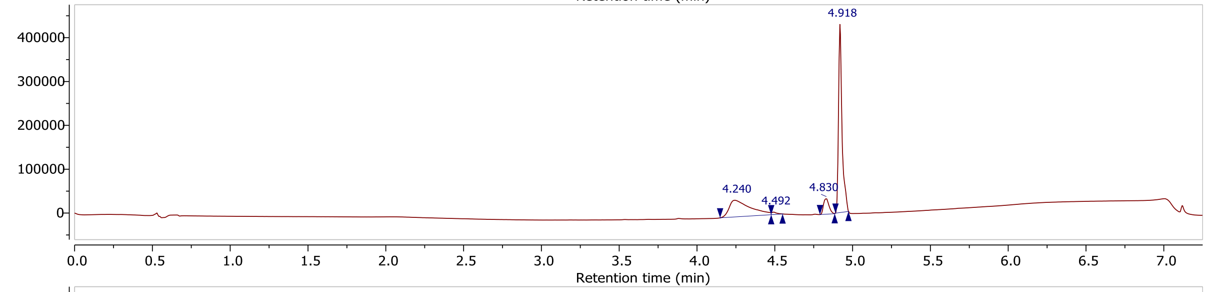

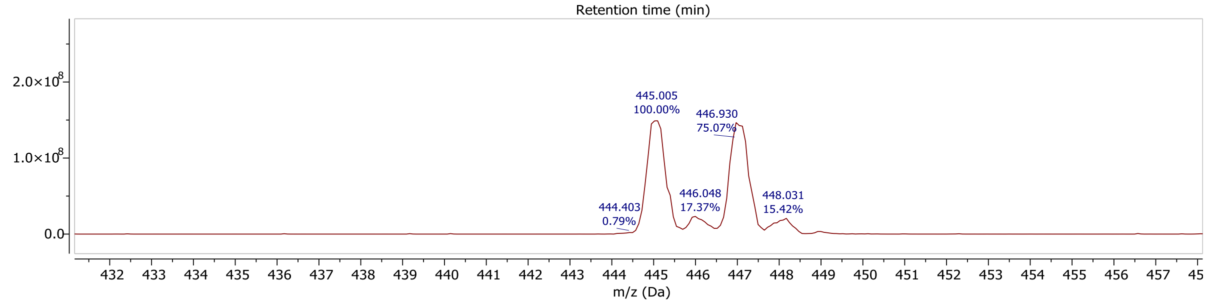

3.2.34 4-(1,3-Dimethylimidazolidin-2-yl)phenol (33a)

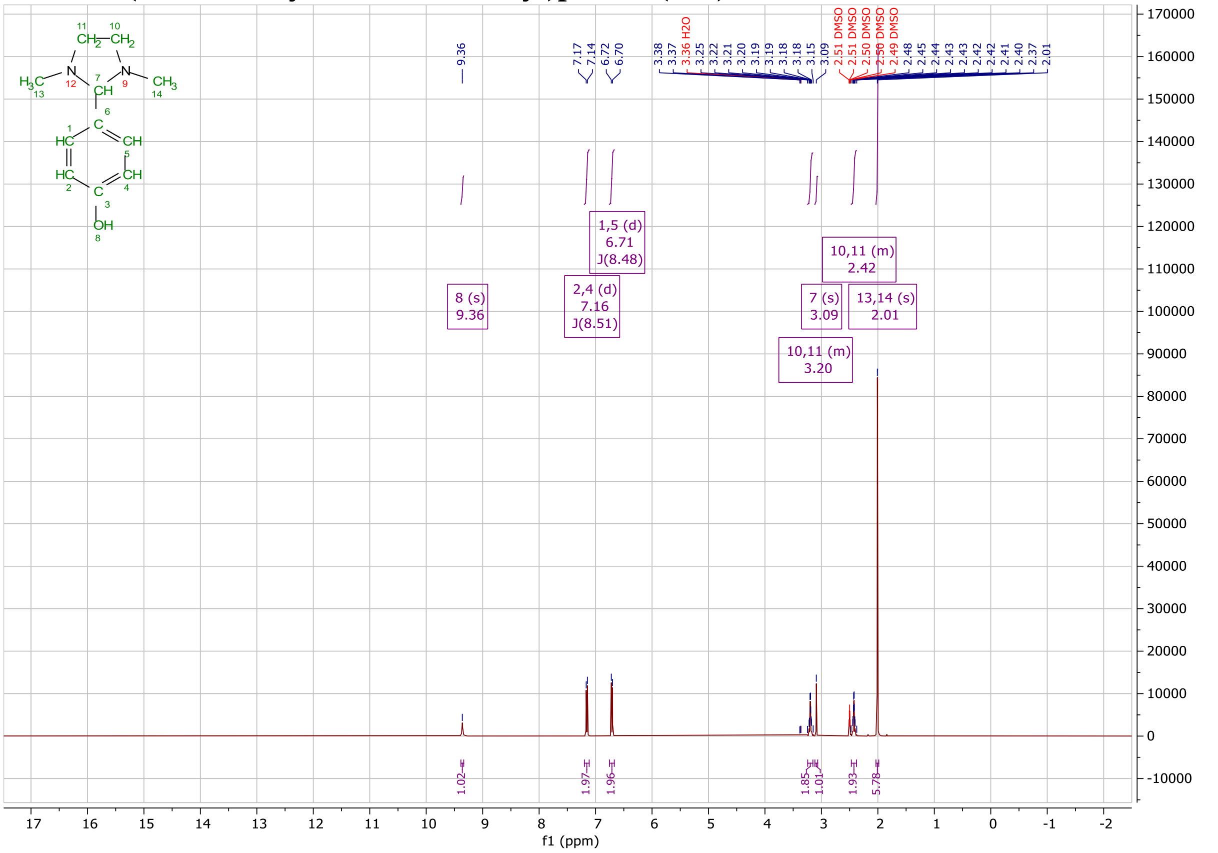

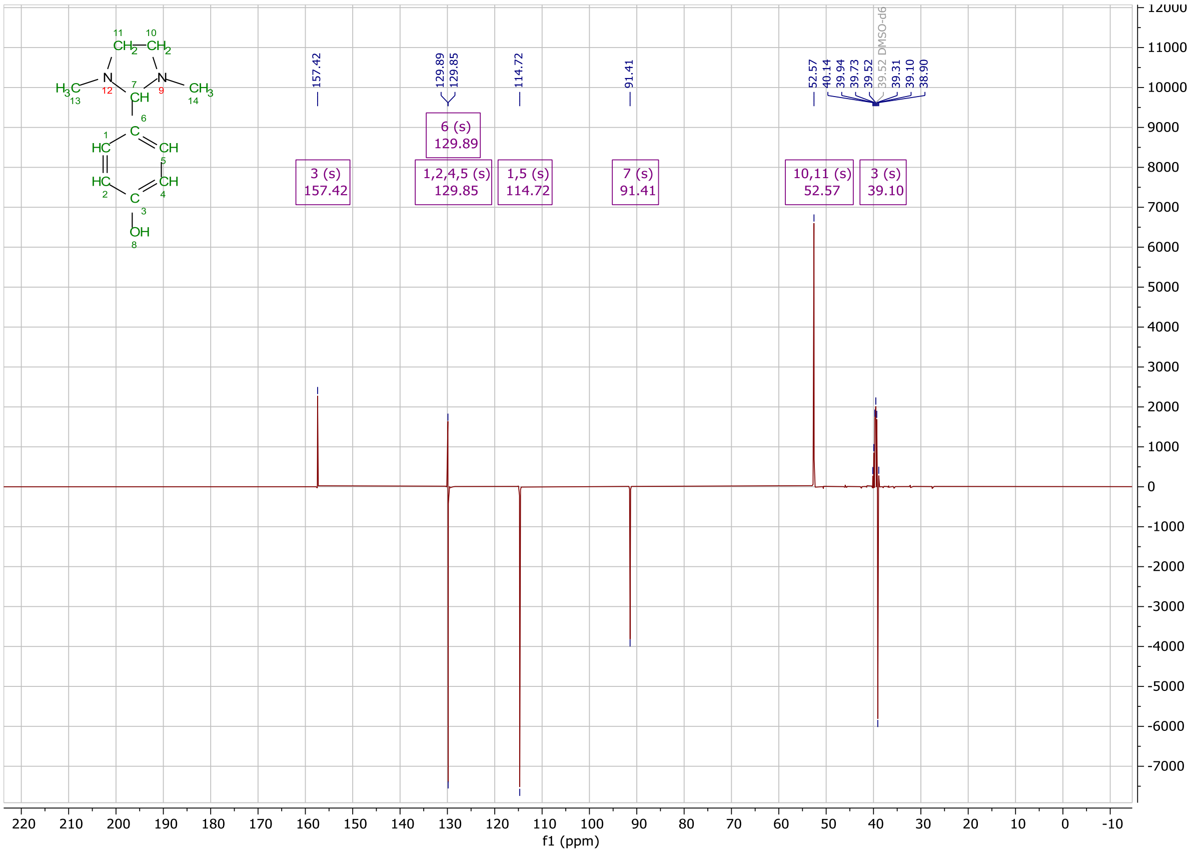
157.42
129.89
129.85
114.72
91.41
52.57
40.14
39.94
39.73
39.52
39.52 DMSO-d6
39.31
39.10
38.90
6 (s)
129.89
3 (s)
157.42
1,2,4,5 (s)
129.85
1,5 (s)
114.72
7 (s)
91.41
10,11 (s)
52.57
3 (s)
39.10
f1 (ppm)

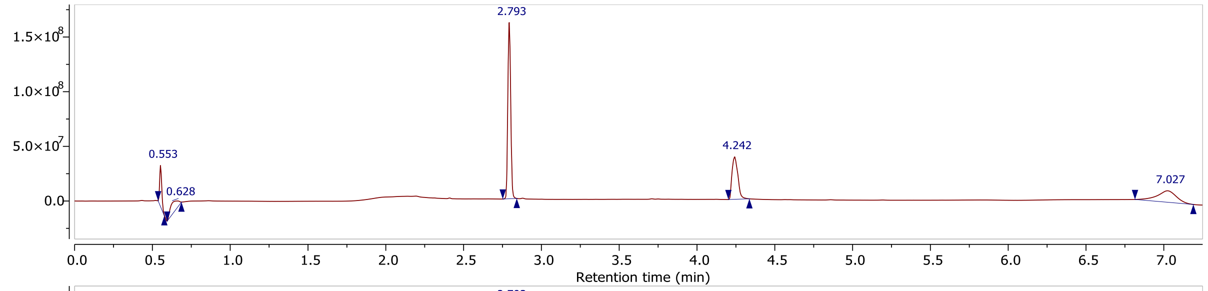
2.793
0.553
0.628
4.242
7.027
Retention time (min)

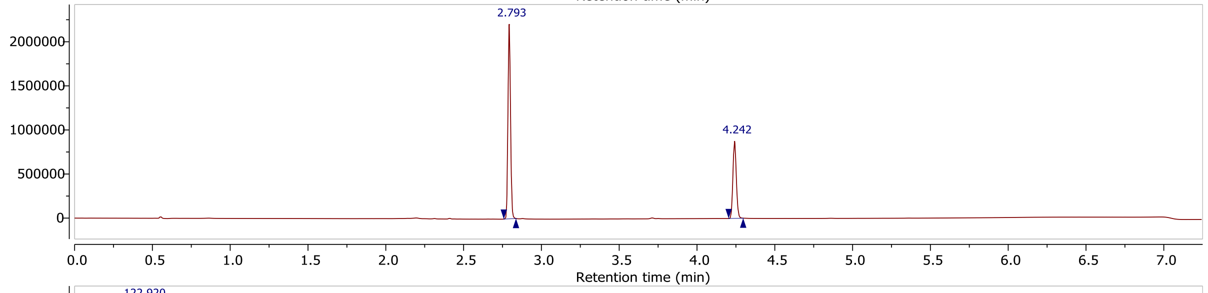
2.793
4.242
Retention time (min)

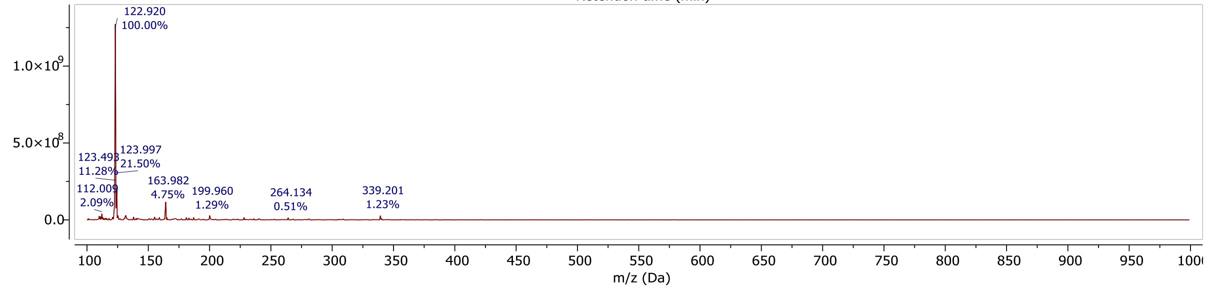
122.920
100.00%
123.493
11.28%
123.997
21.50%
112.009
2.09%
163.982
4.75%
199.960
1.29%
264.134
0.51%
339.201
1.23%
m/z (Da)

3.2.35 3-(1,3-Dimethylimidazolidin-2-yl)phenol (33b)

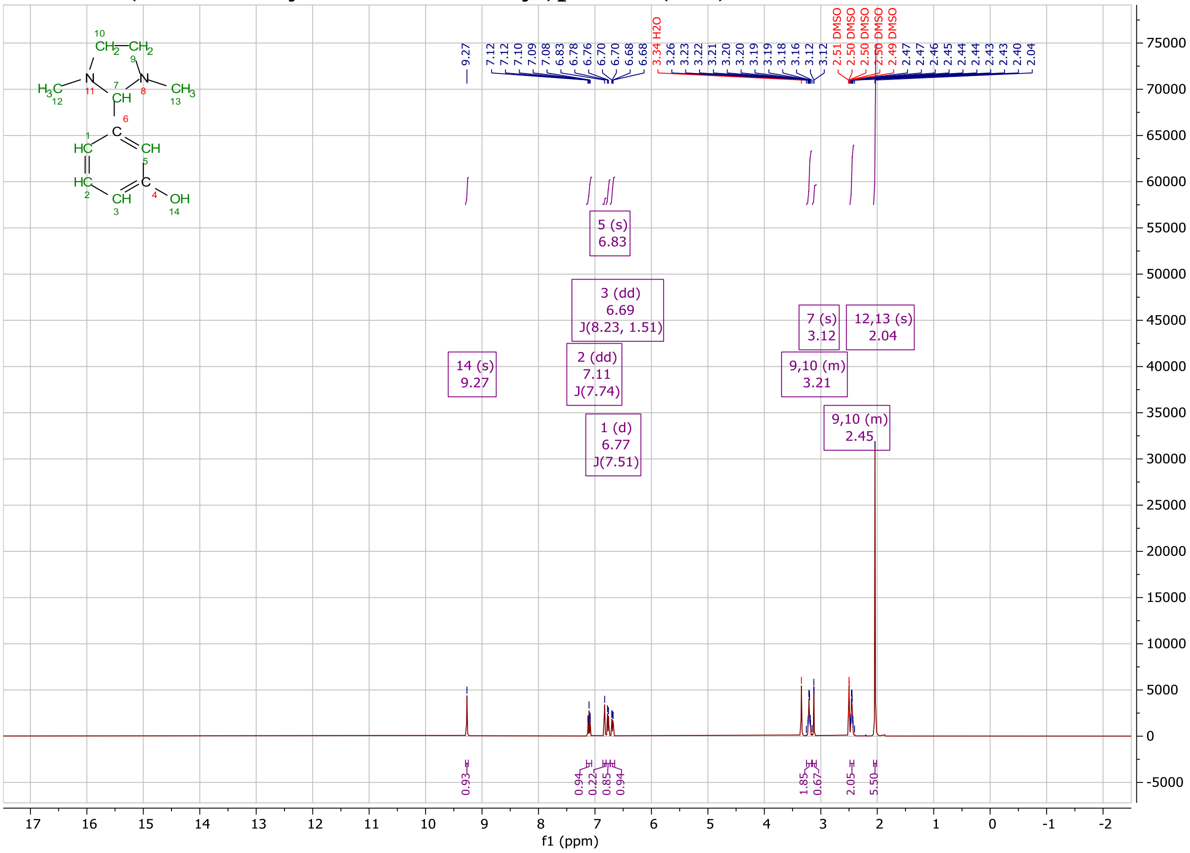

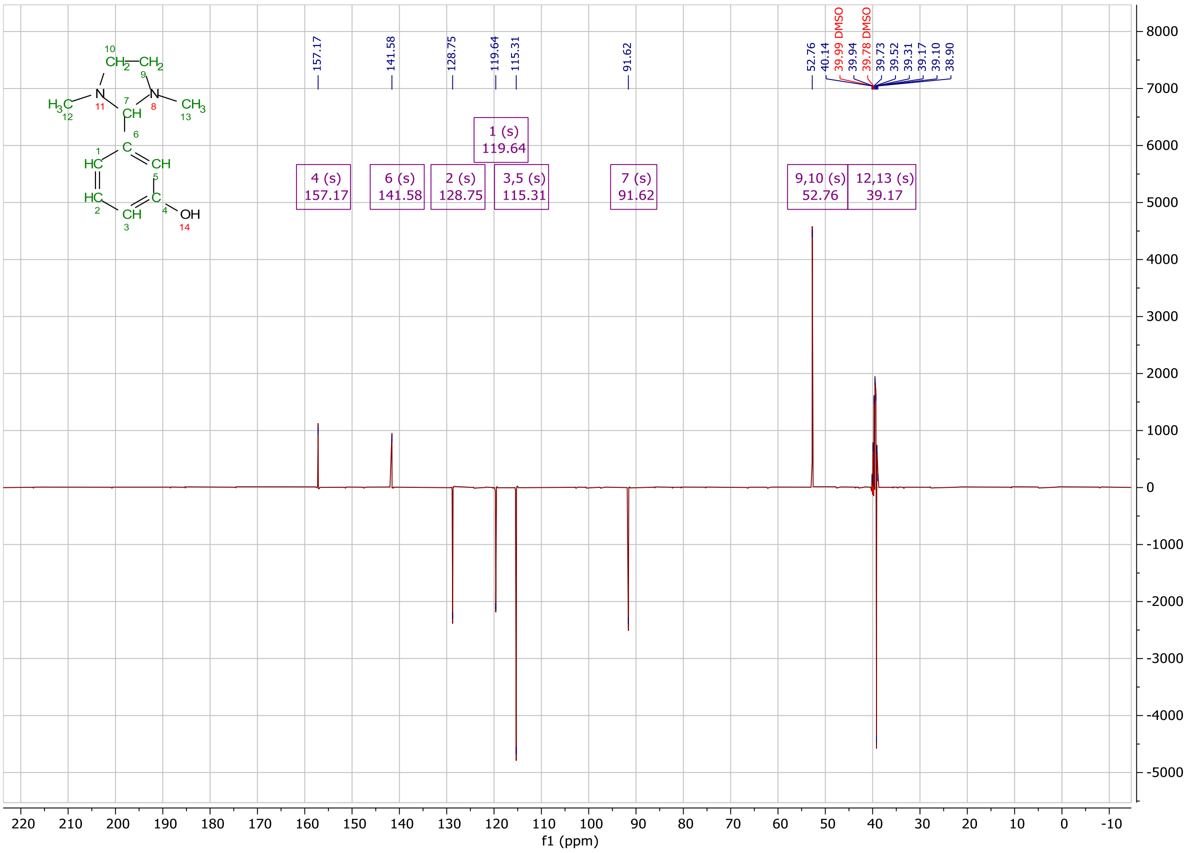

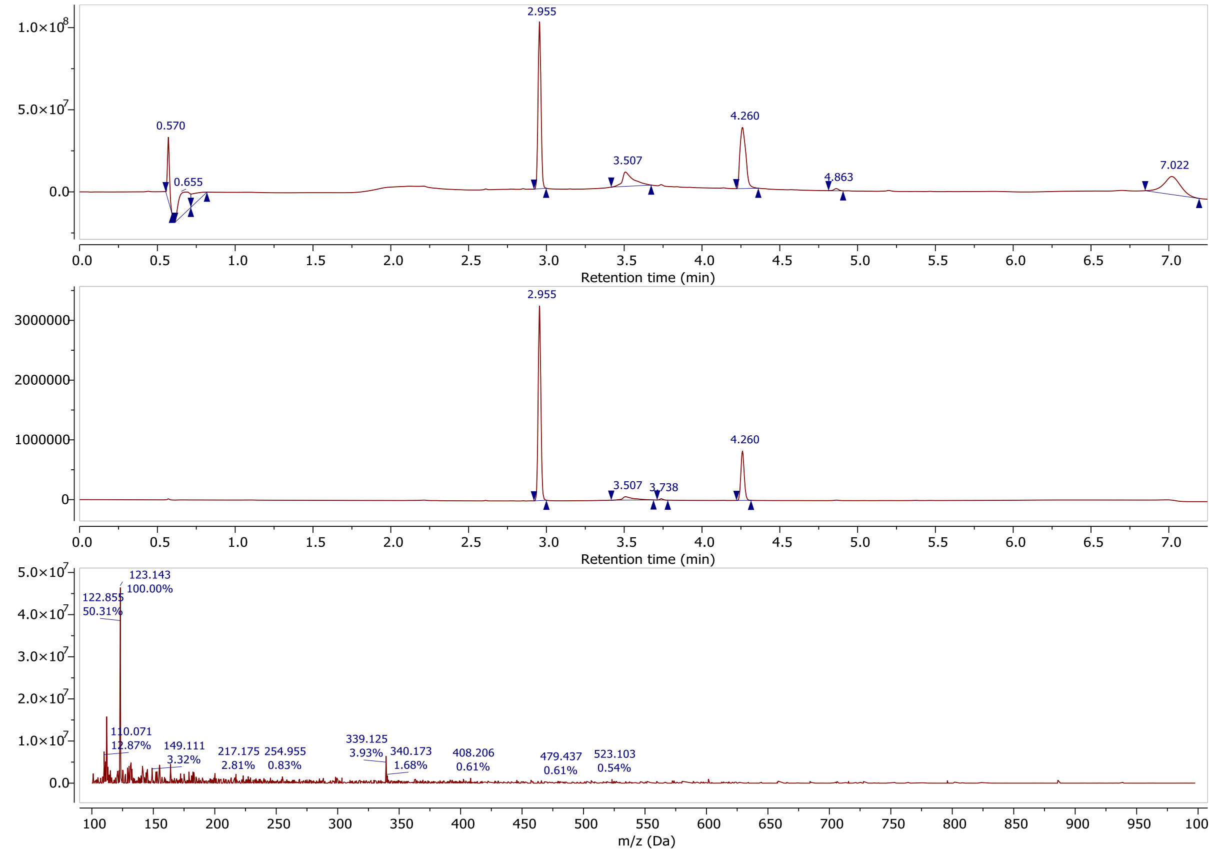

3.2.36 2-(1,3-Dimethylimidazolidin-2-yl)phenol (33c)

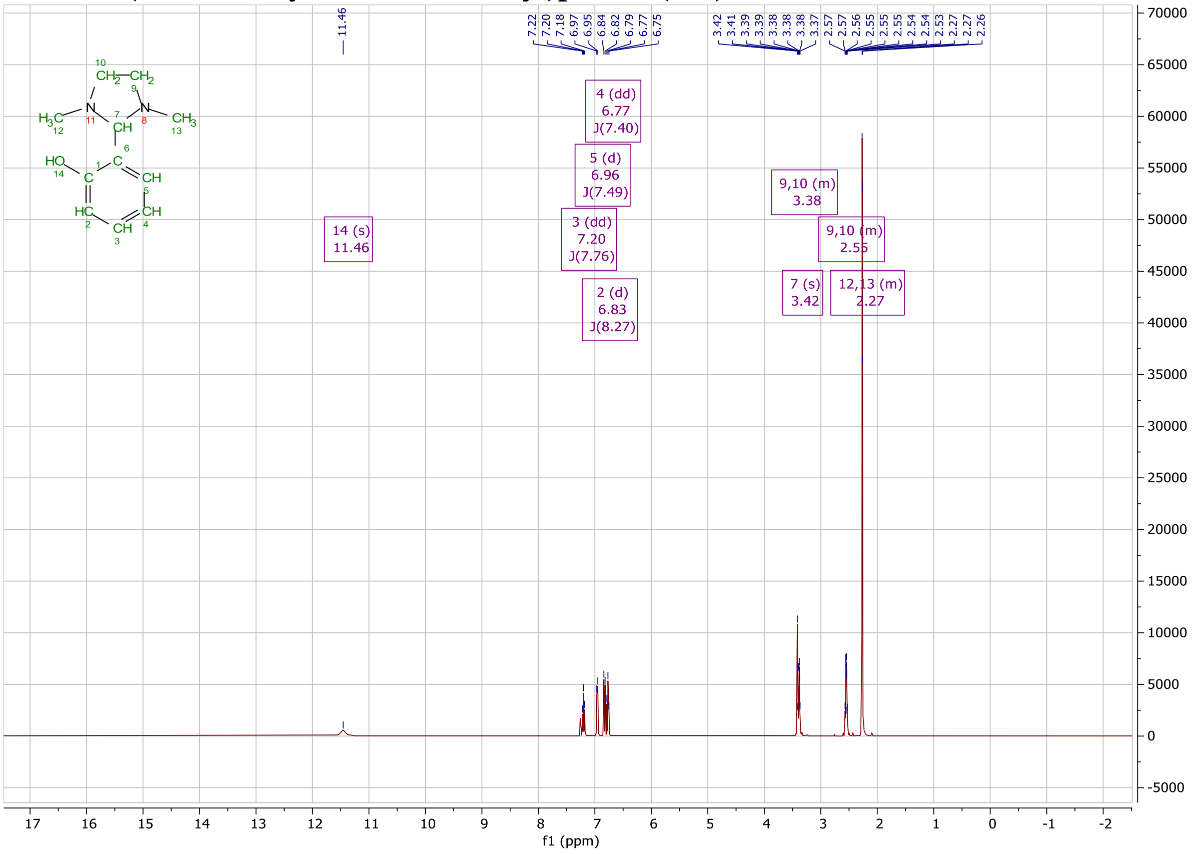

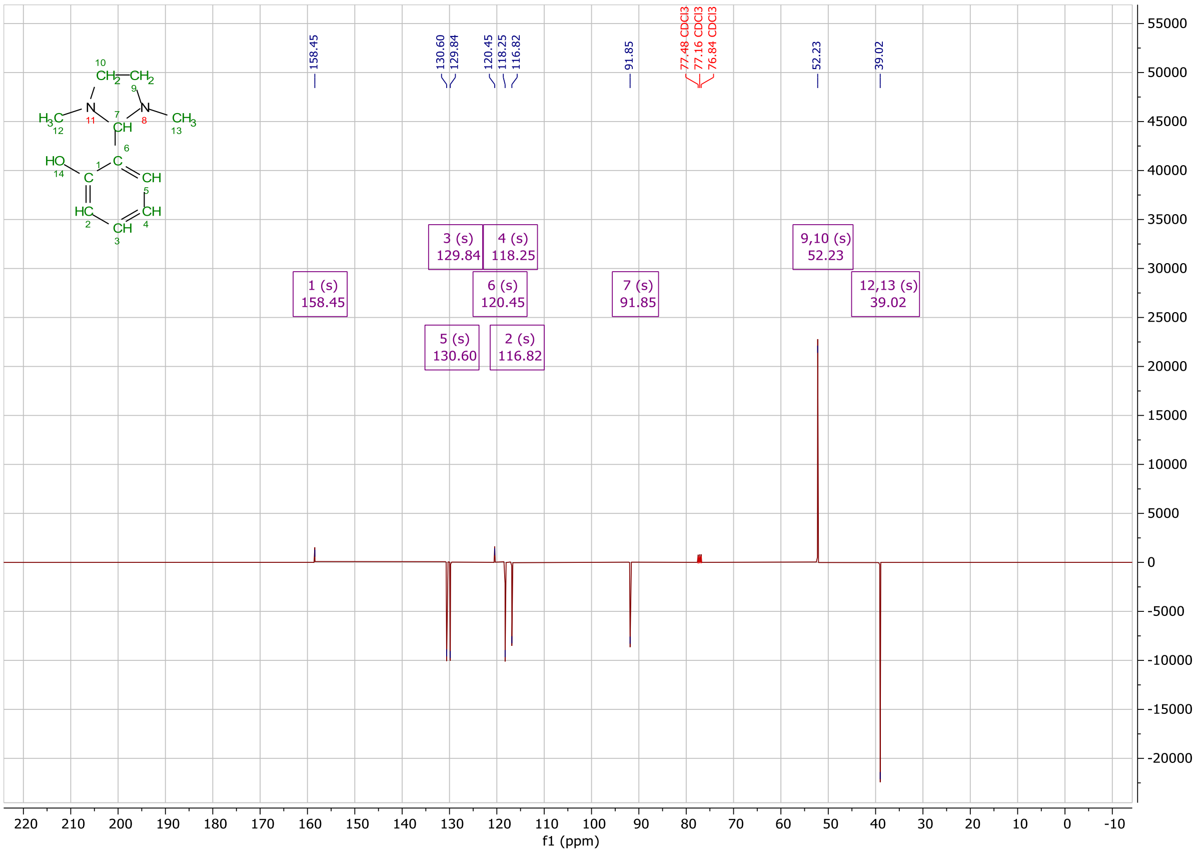

158.45
130.60
129.84
120.45
118.25
116.82
91.85
77.48 CDCl3
77.16 CDCl3
76.84 CDCl3
52.23
39.02
1 (s) 158.45
3 (s) 129.84
4 (s) 118.25
5 (s) 130.60
6 (s) 120.45
2 (s) 116.82
7 (s) 91.85
9,10 (s) 52.23
12,13 (s) 39.02
f1 (ppm)

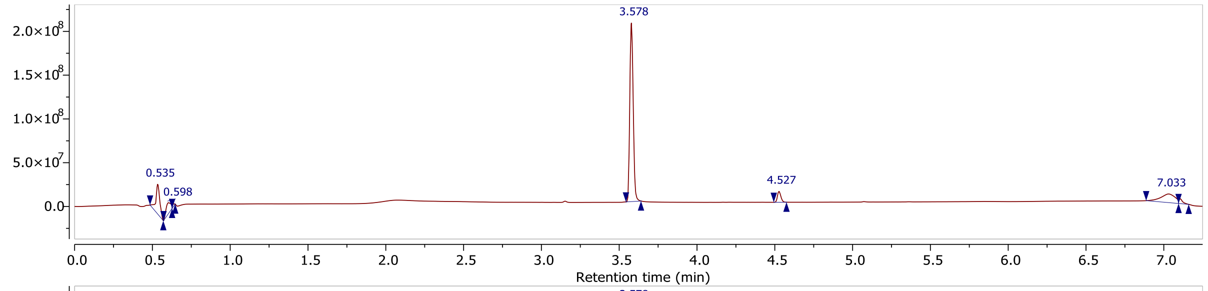

3.578
0.535
0.598
4.527
7.033
Retention time (min)

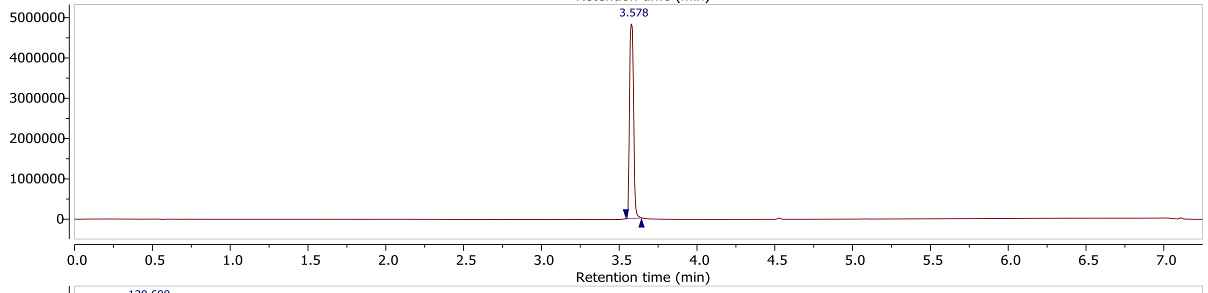

3.578
Retention time (min)

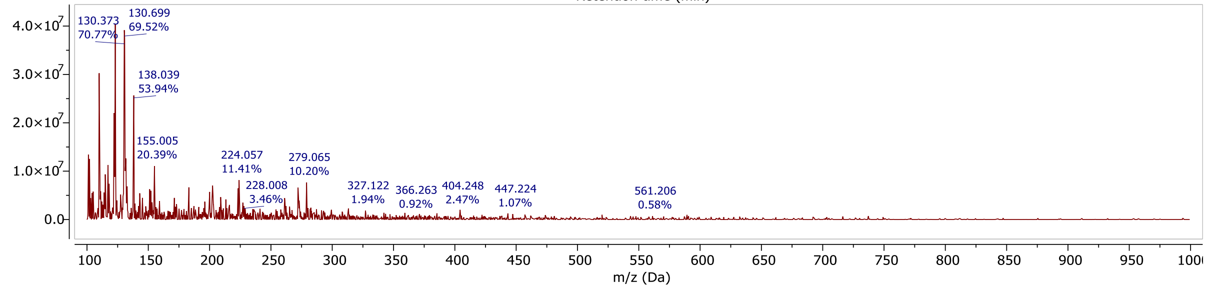

130.373 70.77%
130.699 69.52%
138.039 53.94%
155.005 20.39%
224.057 11.41%
228.008 3.46%
279.065 10.20%
327.122 1.94%
366.263 0.92%
404.248 2.47%
447.224 1.07%
561.206 0.58%
m/z (Da)

3.2.37 4-Hydroxy-2-iodobenzaldehyde (34a)

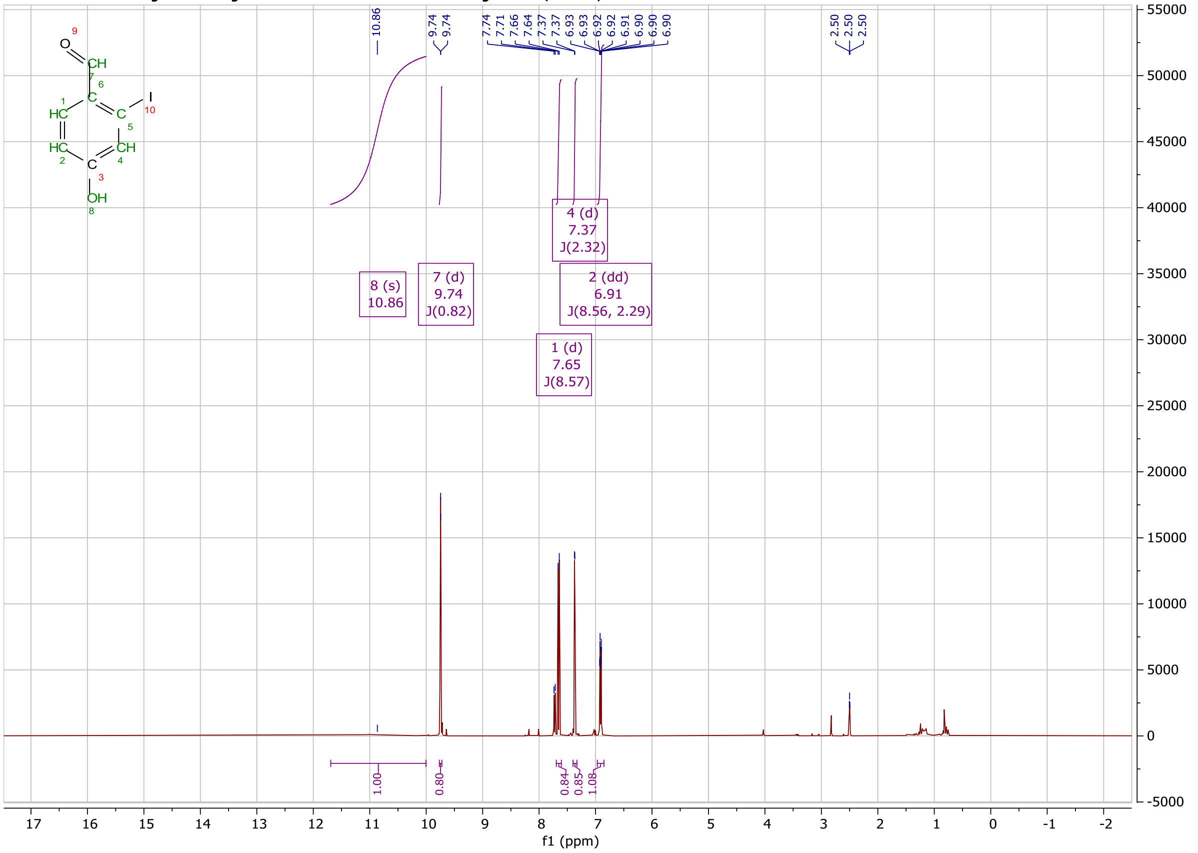

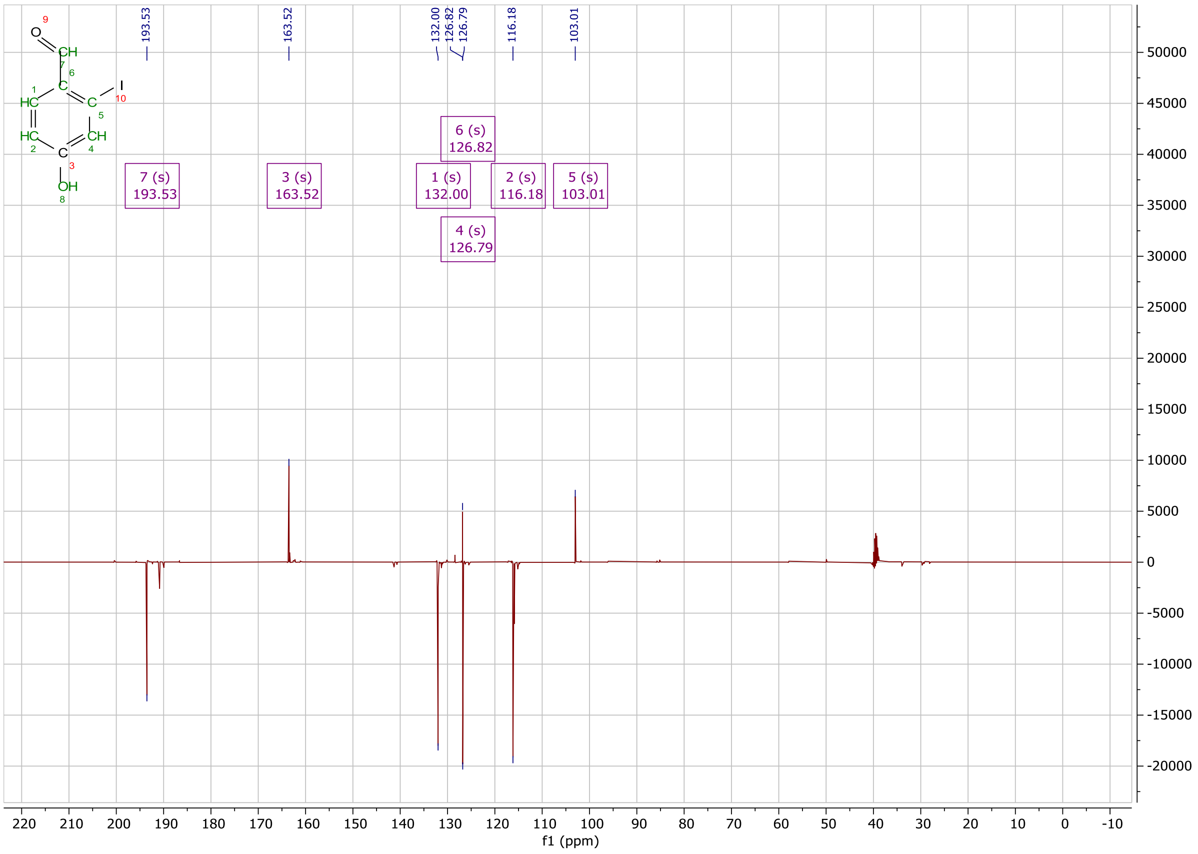

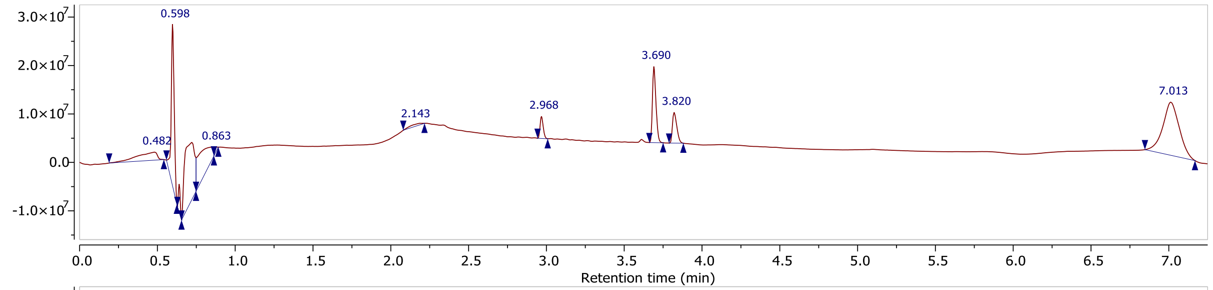

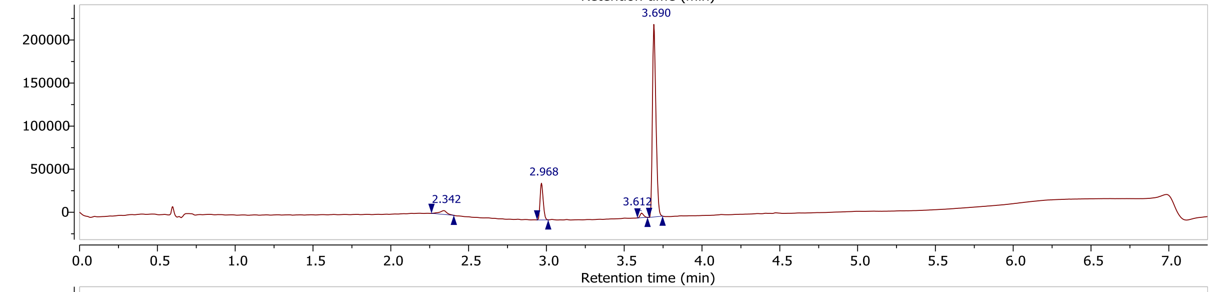

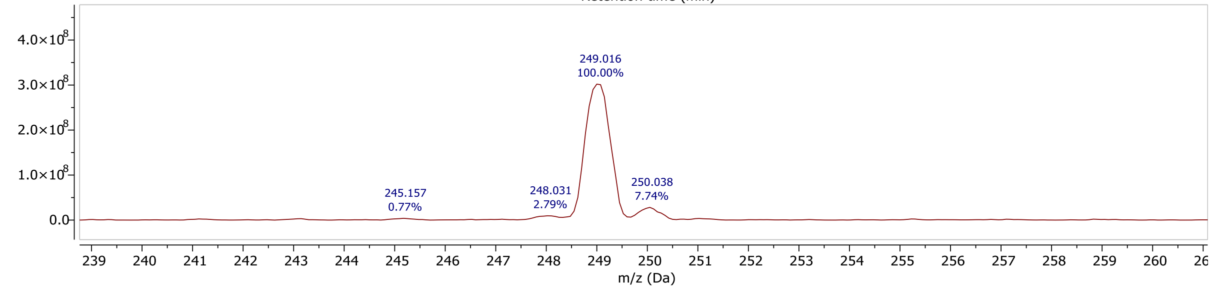

3.2.38 2-Hydroxy-3-iodobenzaldehyde (35)

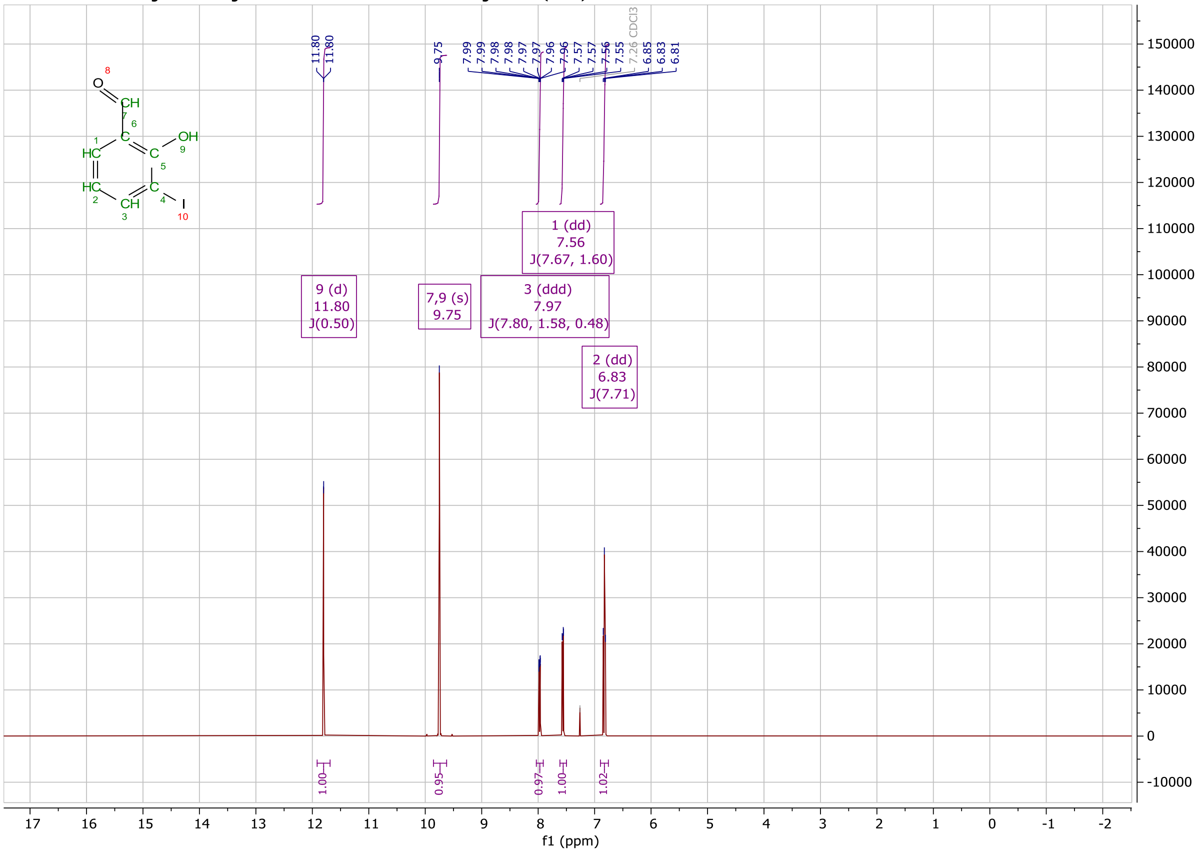

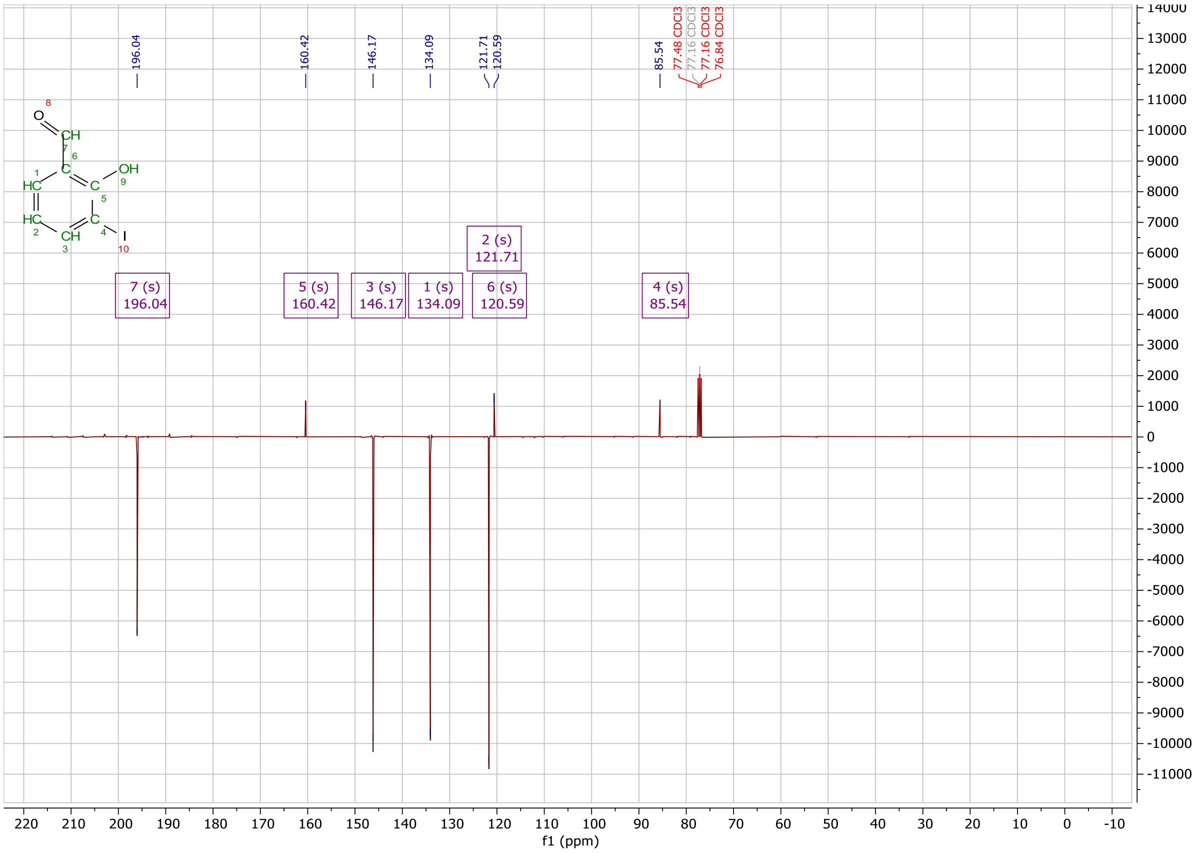
196.04
160.42
146.17
134.09
121.71
120.59
85.54
77.48 CDCl3
77.16 CDCl3
77.16 CDCl3
76.84 CDCl3
O
CH
C
OH
HC
C
HC
C
CH
I
7 (s)
196.04
5 (s)
160.42
3 (s)
146.17
1 (s)
134.09
2 (s)
121.71
6 (s)
120.59
4 (s)
85.54
f1 (ppm)

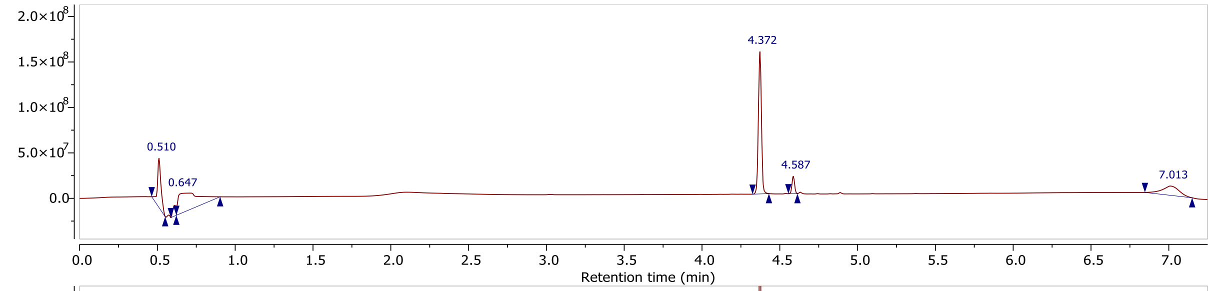
4.372
0.510
0.647
4.587
7.013
Retention time (min)

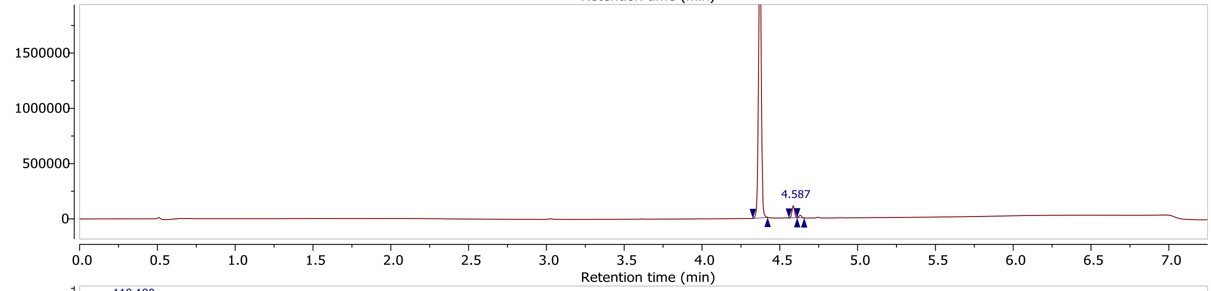
4.587
Retention time (min)

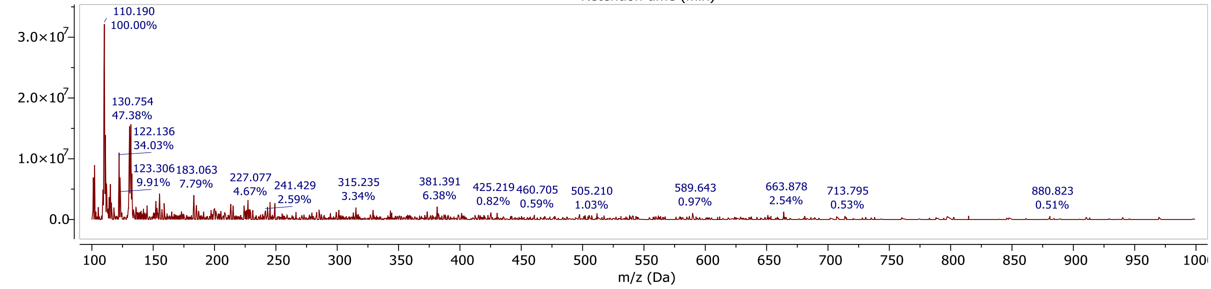
110.190
100.00%
130.754
47.38%
122.136
34.03%
123.306
9.91%
183.063
7.79%
227.077
4.67%
241.429
2.59%
315.235
3.34%
381.391
6.38%
425.219
0.82%
460.705
0.59%
505.210
1.03%
589.643
0.97%
663.878
2.54%
713.795
0.53%
880.823
0.51%
m/z (Da)

3.2.39 3-Hydroxy-4-iodobenzaldehyde (36)

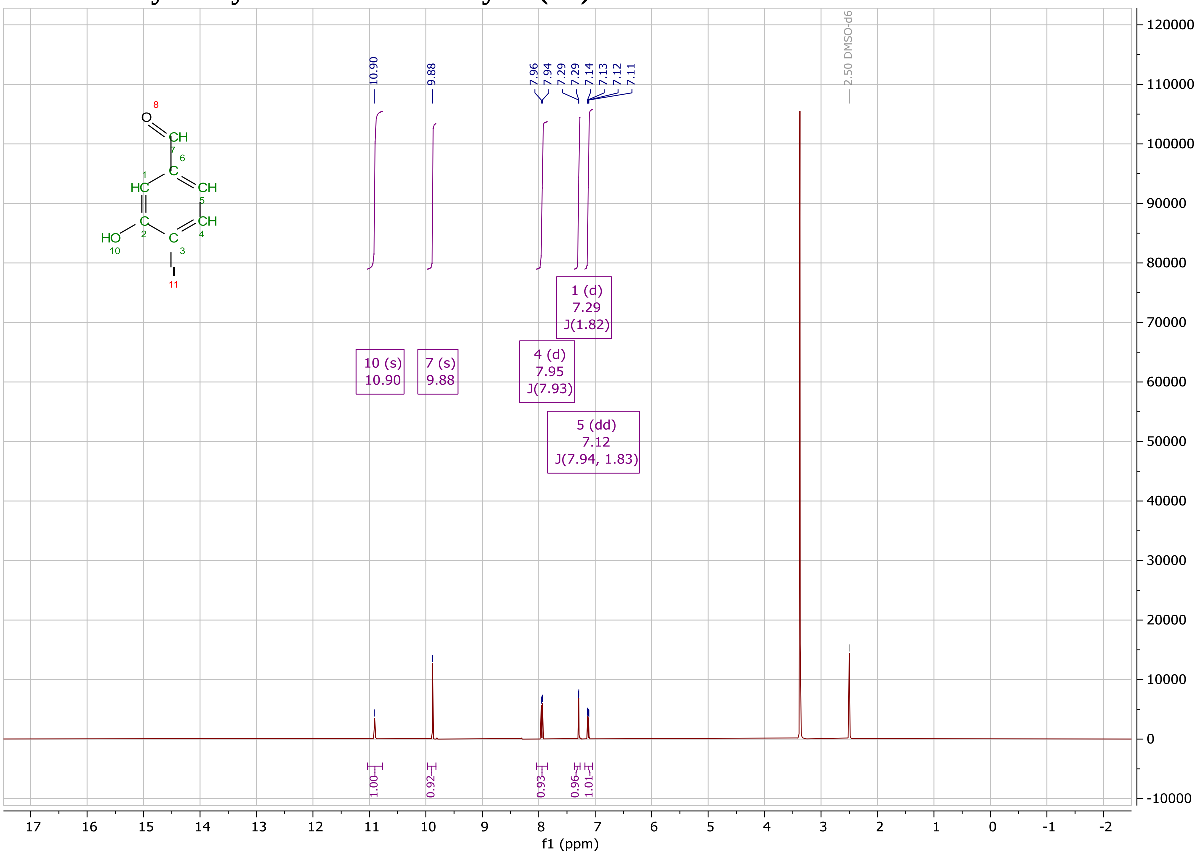

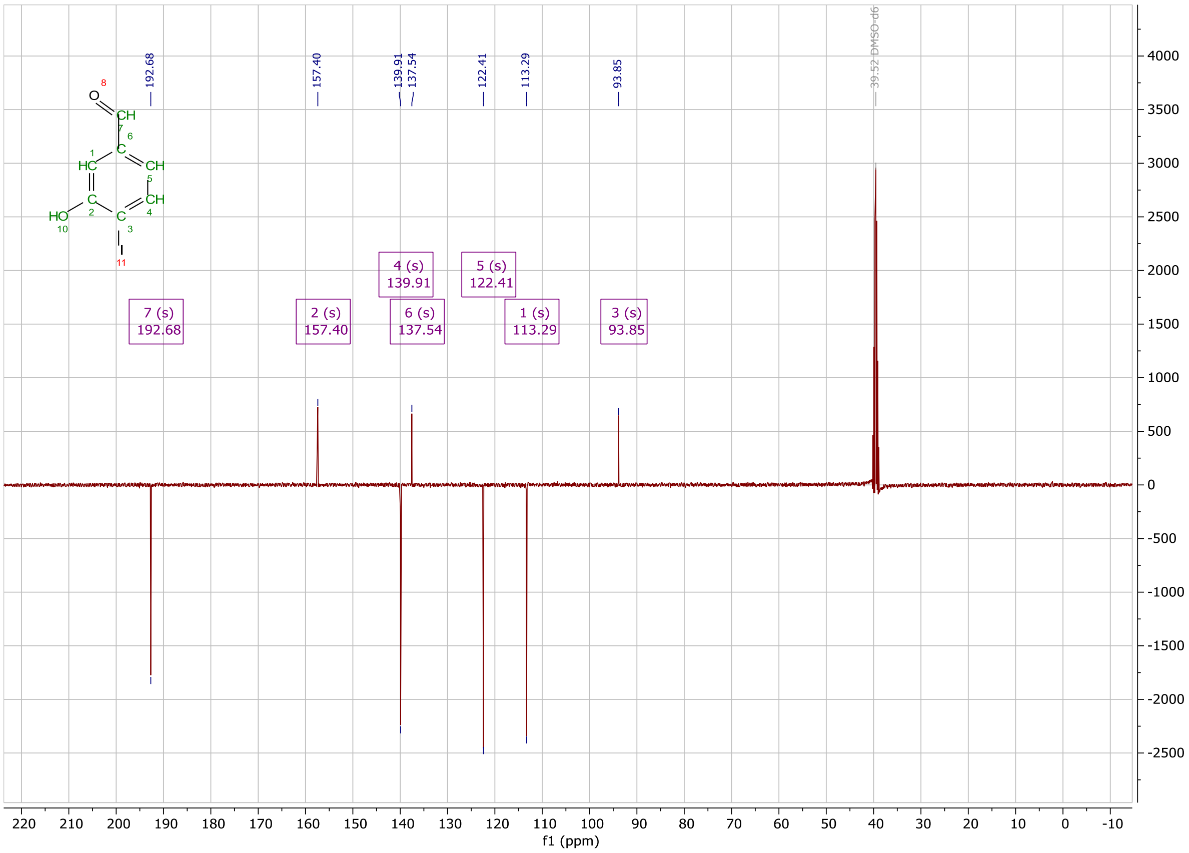

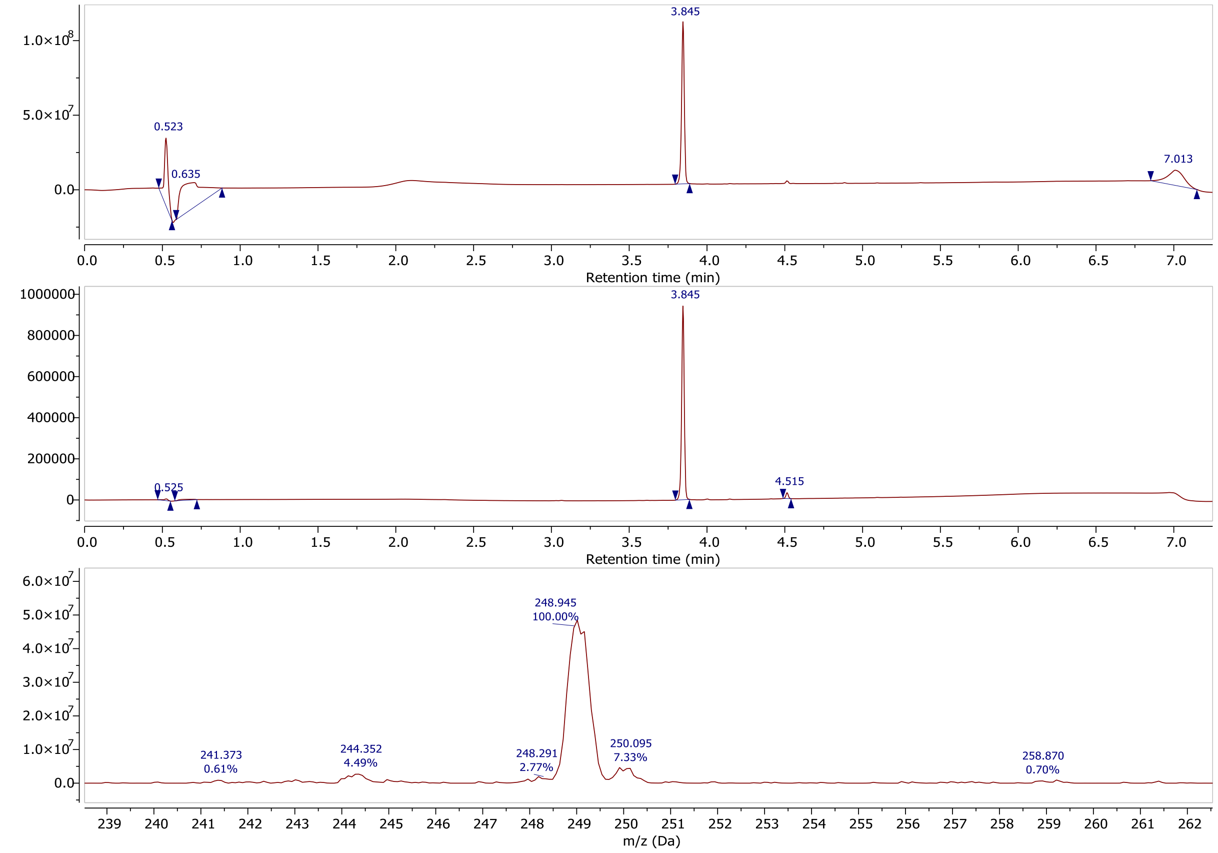

3.2.40 2-Iodo-5-methoxybenzaldehyde (38)

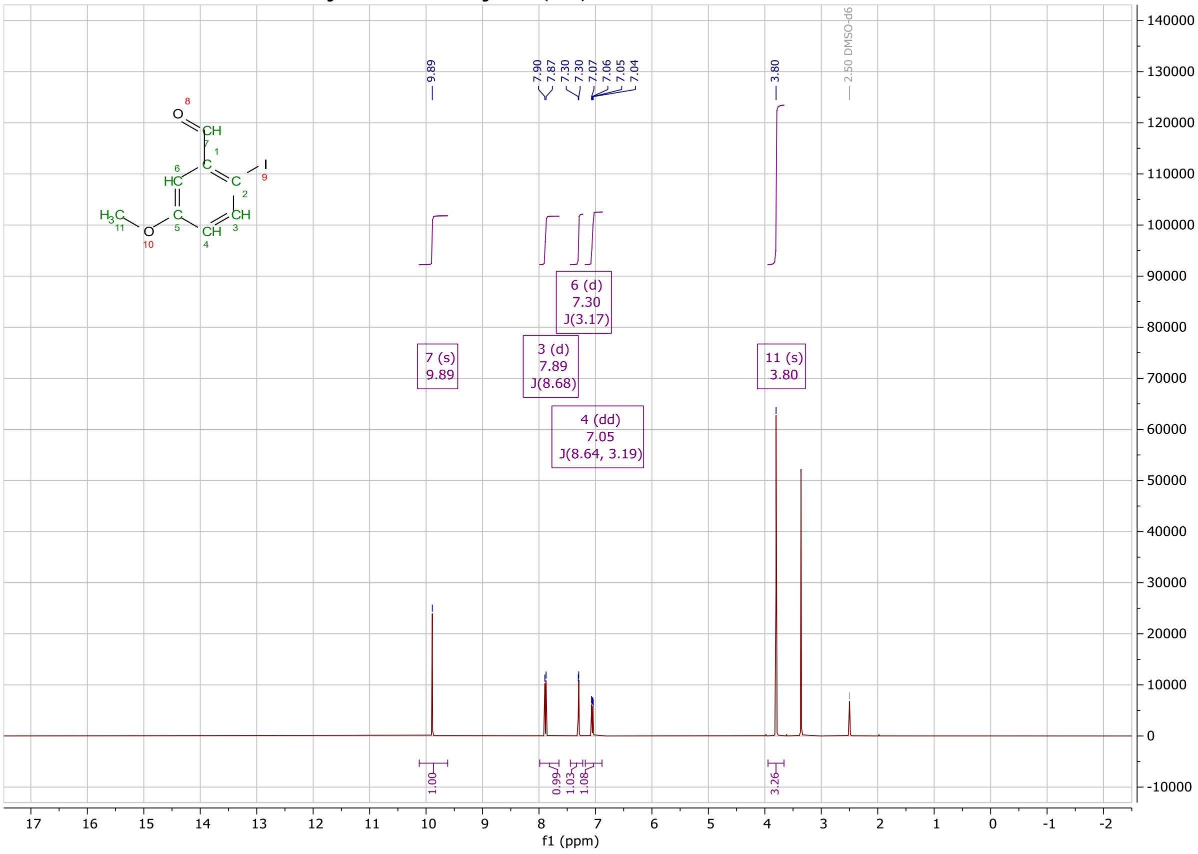

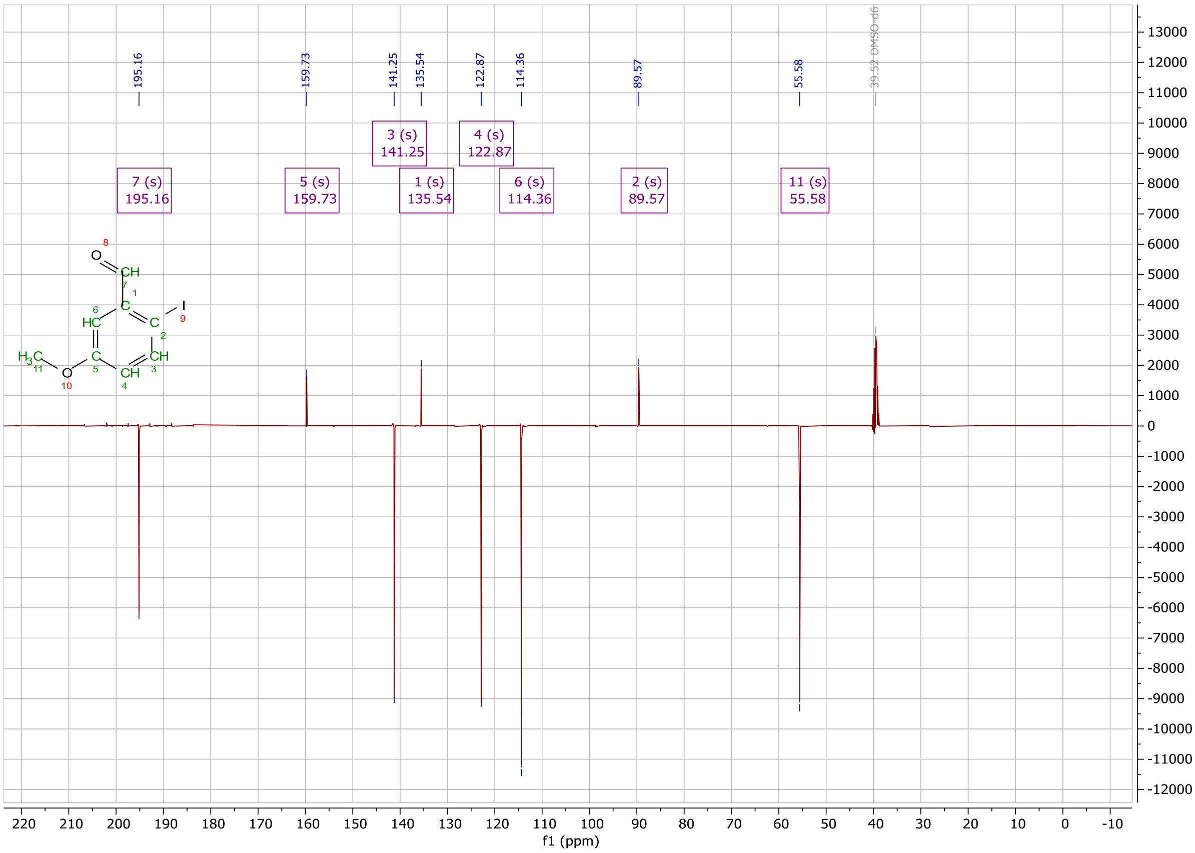

195.16
159.73
141.25
135.54
122.87
114.36
89.57
55.58
39.52 DMSO-d6
3 (s)
141.25
4 (s)
122.87
7 (s)
195.16
5 (s)
159.73
1 (s)
135.54
6 (s)
114.36
2 (s)
89.57
11 (s)
55.58
f1 (ppm)

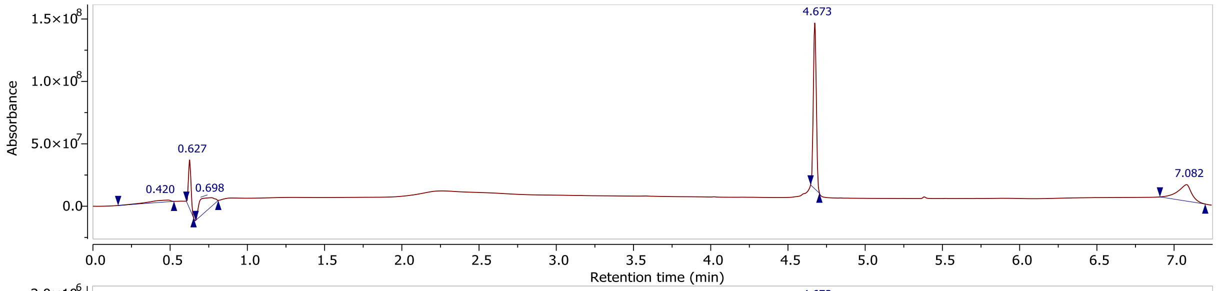

4.673
0.627
0.420
0.698
7.082
Absorbance
Retention time (min)

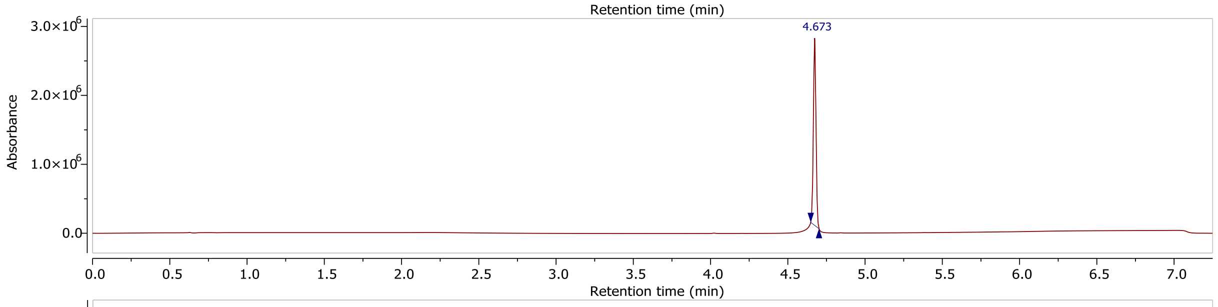

4.673
Absorbance
Retention time (min)

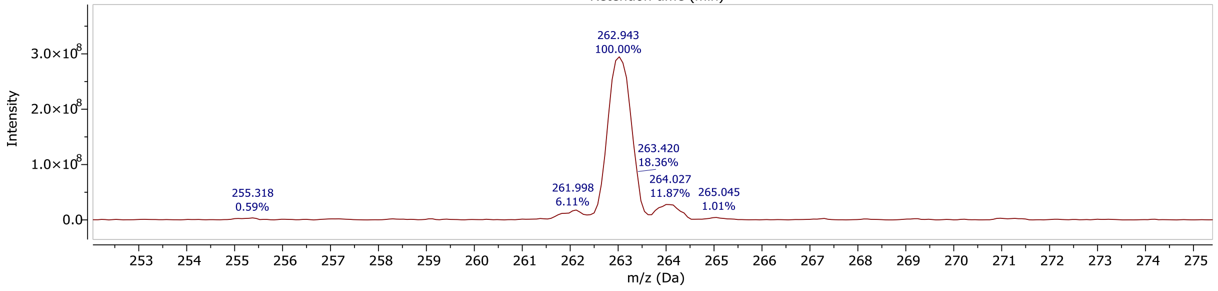

262.943
100.00%
263.420
18.36%
264.027
11.87%
265.045
1.01%
261.998
6.11%
255.318
0.59%
Intensity
m/z (Da)

3.2.41 5-Hydroxy-2-iodobenzaldehyde (34b)

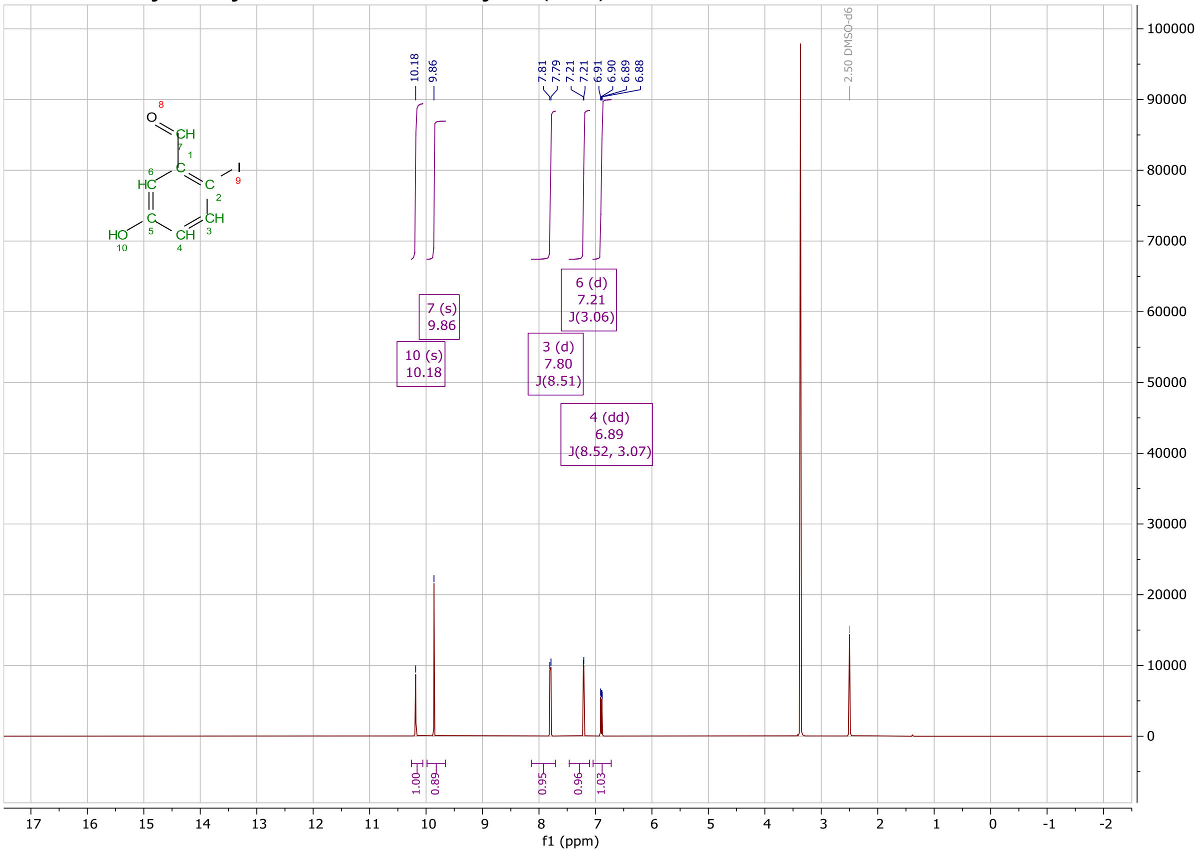

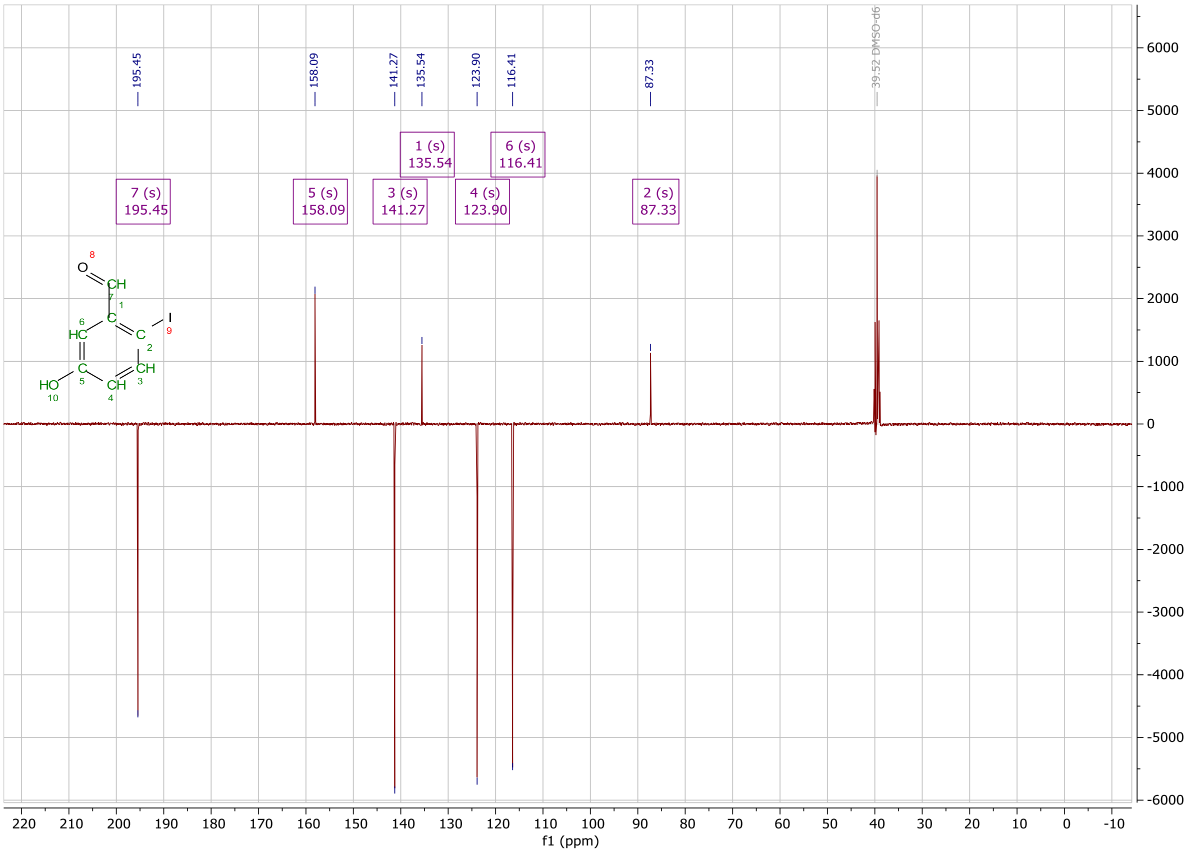

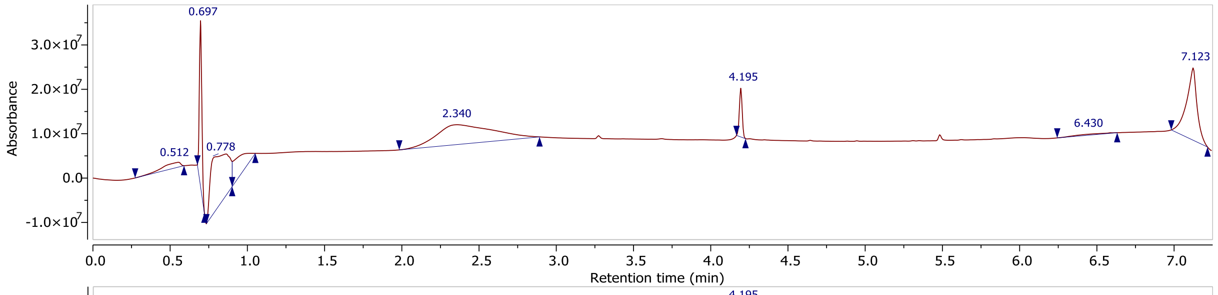

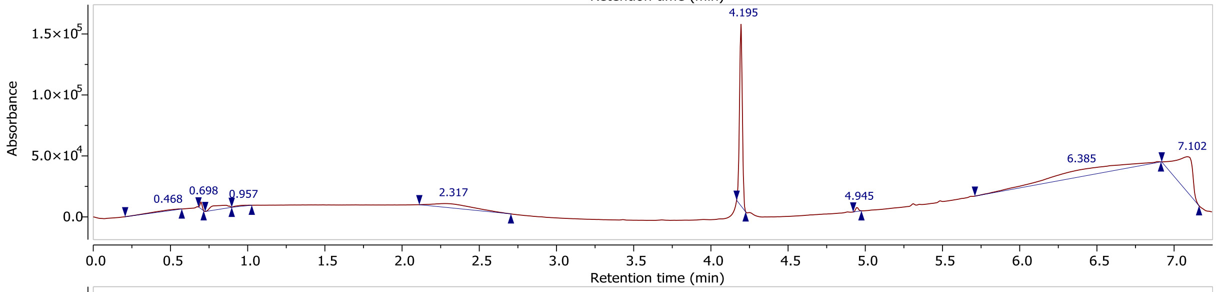

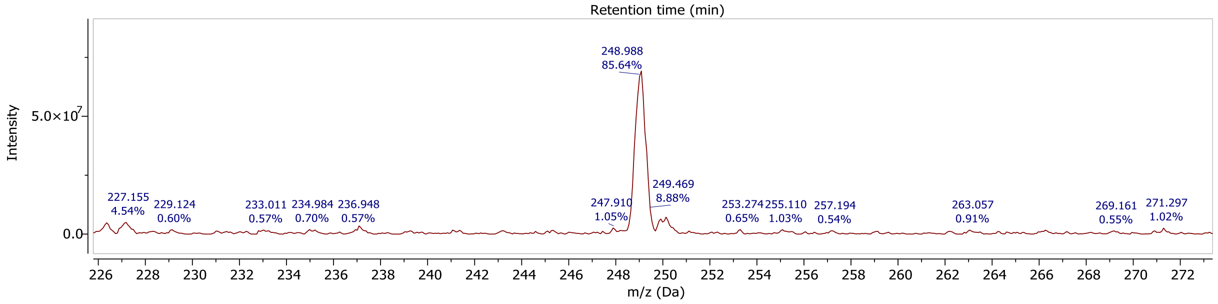

3.2.42 2-(2-Methoxyphenyl)-1,3-dimethylimidazolidine (40)

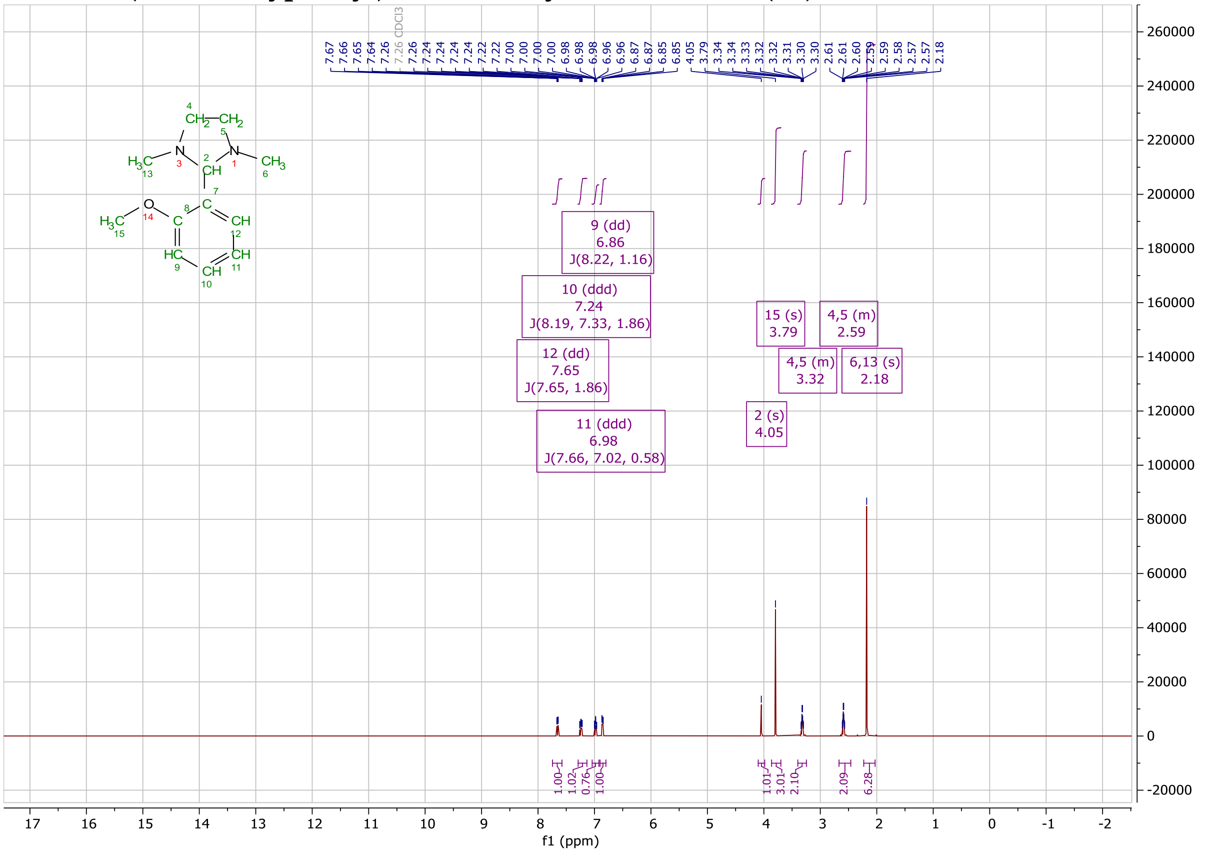

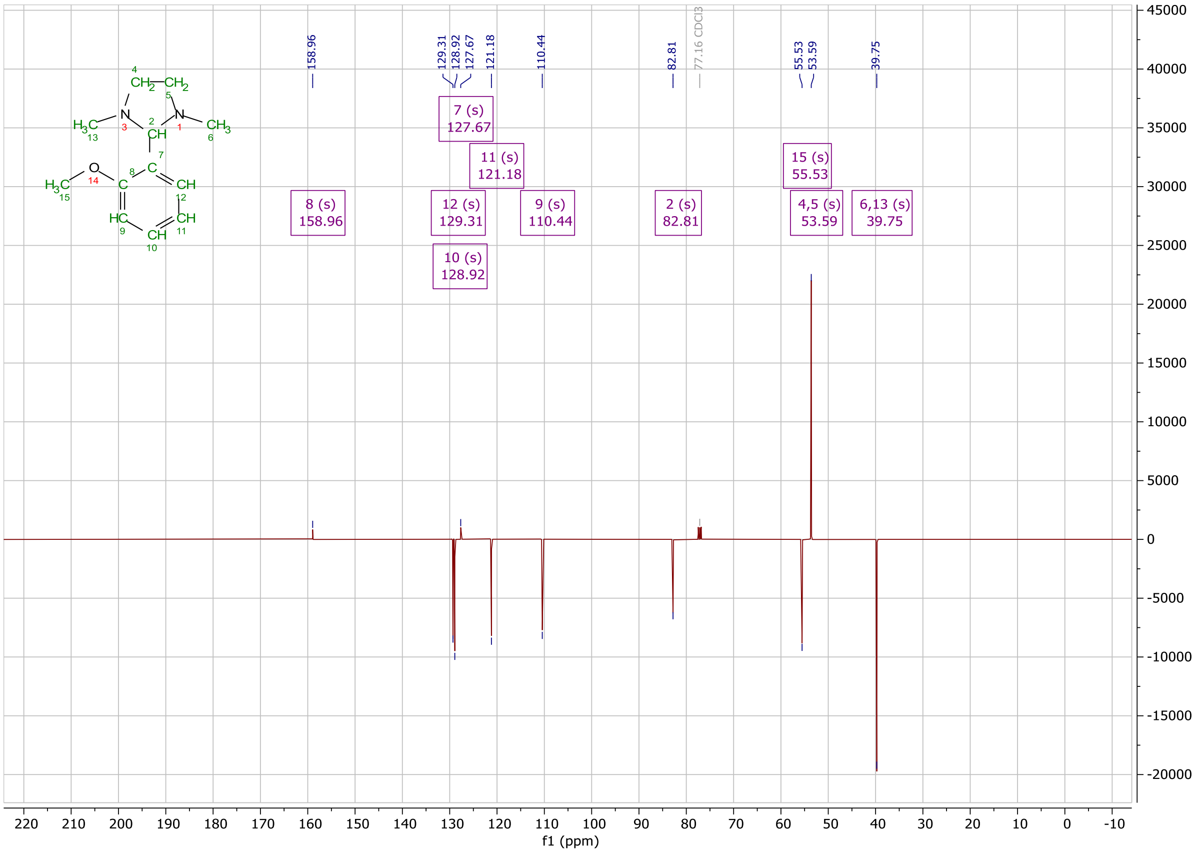

158.96
129.31
128.92
127.67
121.18
110.44
82.81
77.16 CDCl3
55.53
53.59
39.75
7 (s) 127.67
11 (s) 121.18
15 (s) 55.53
8 (s) 158.96
12 (s) 129.31
9 (s) 110.44
2 (s) 82.81
4,5 (s) 53.59
6,13 (s) 39.75
10 (s) 128.92
f1 (ppm)

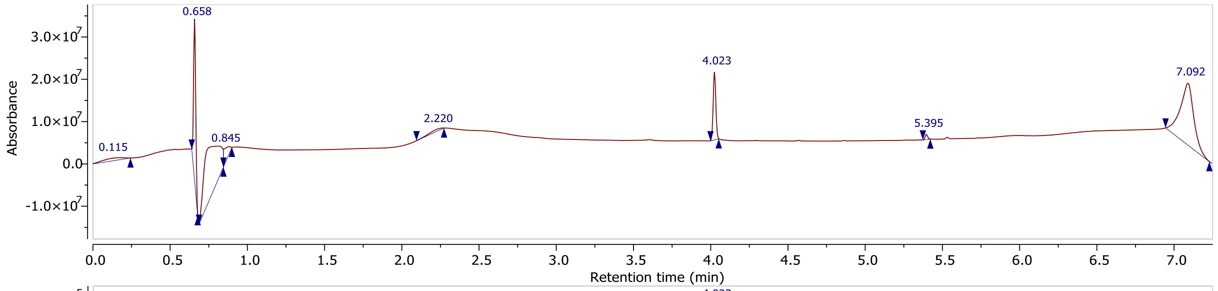

Absorbance
0.658
0.115
0.845
2.220
4.023
5.395
7.092
Retention time (min)

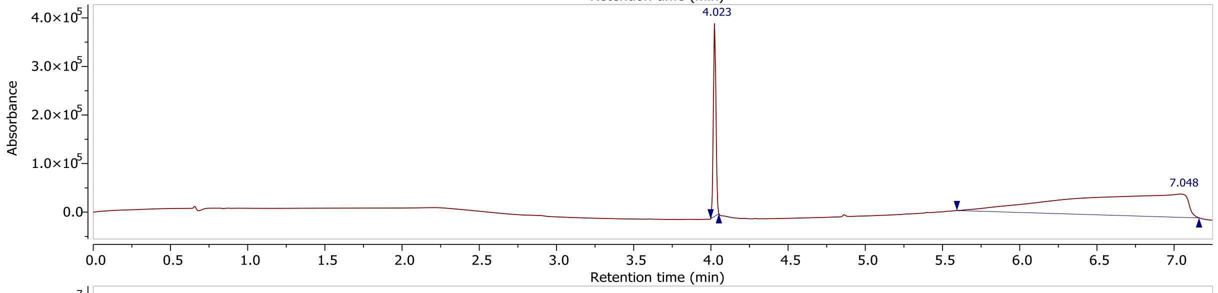

Absorbance
4.023
7.048
Retention time (min)

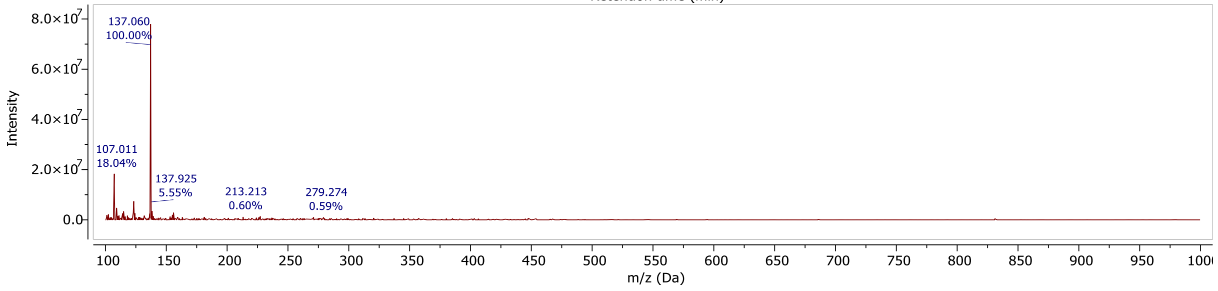

Intensity
137.060
100.00%
107.011
18.04%
137.925
5.55%
213.213
0.60%
279.274
0.59%
m/z (Da)

3.2.43 2-Iodo-6-methoxybenzaldehyde (41)

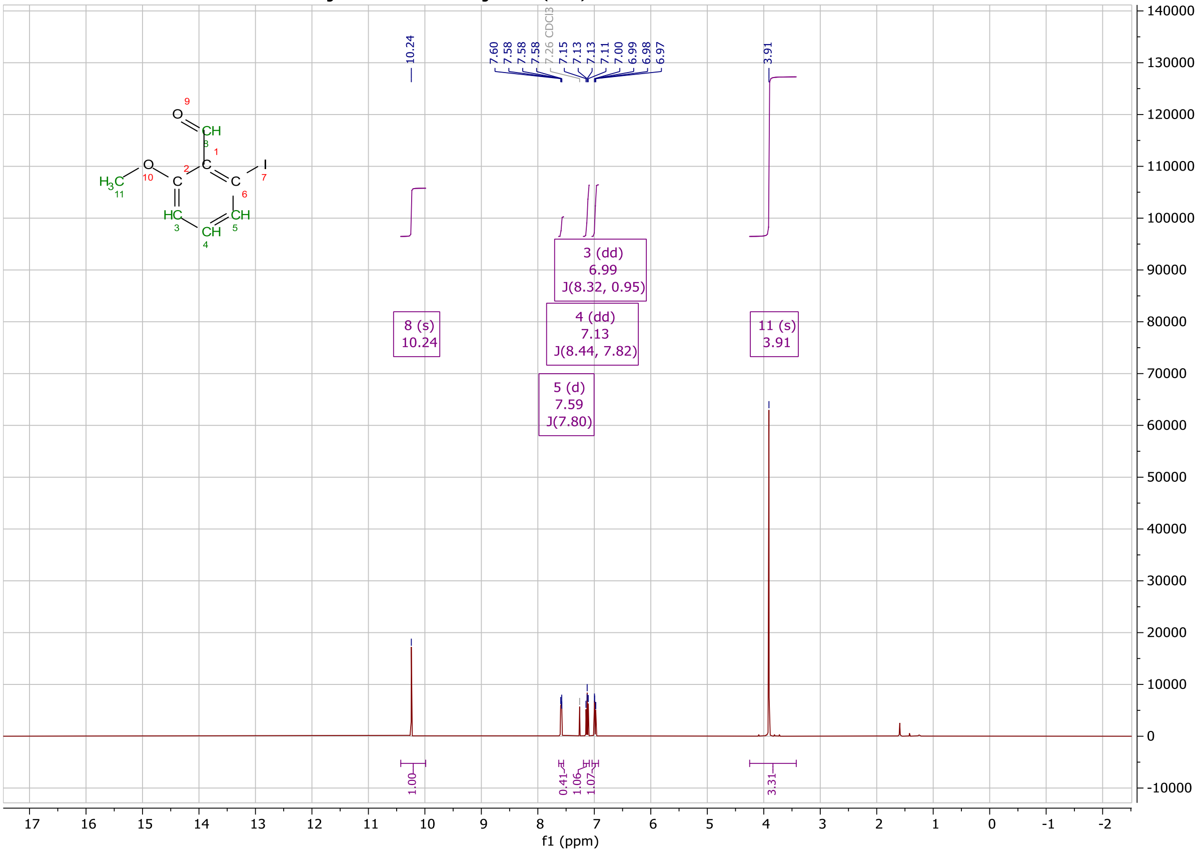

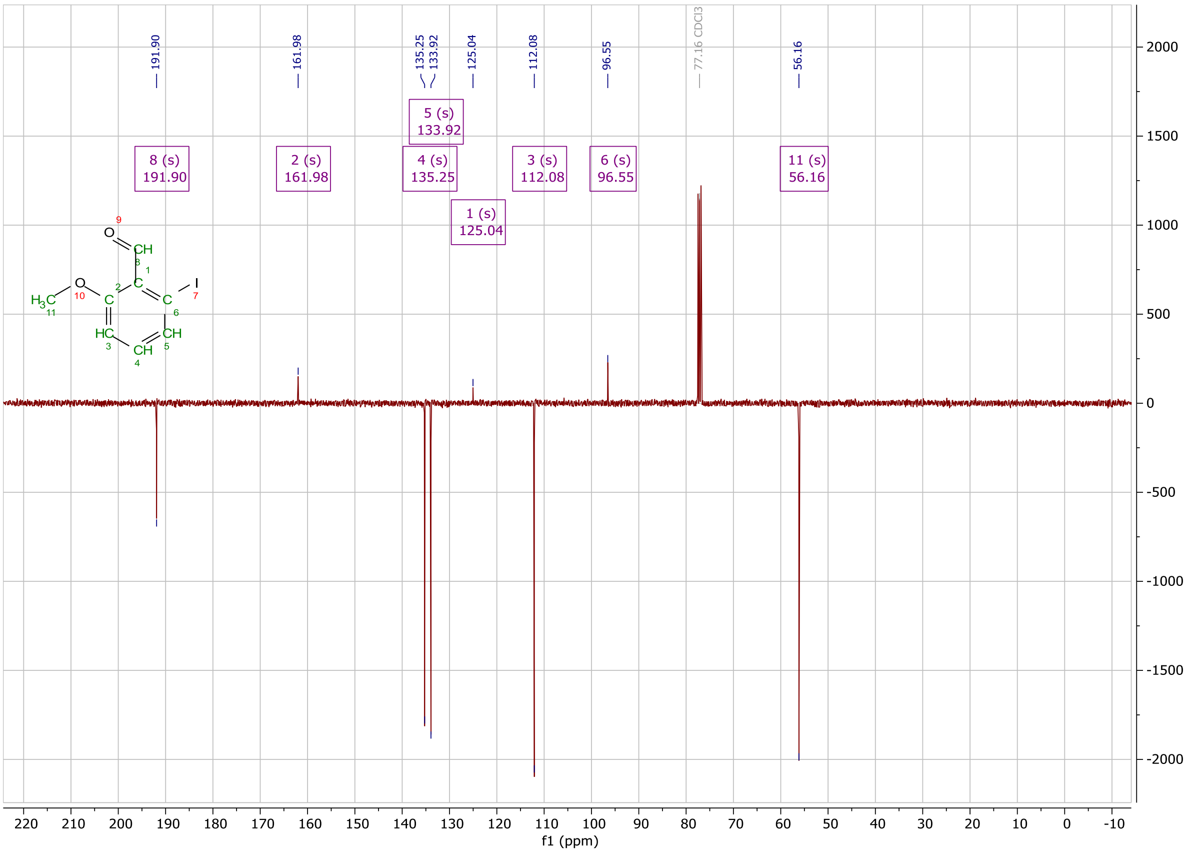

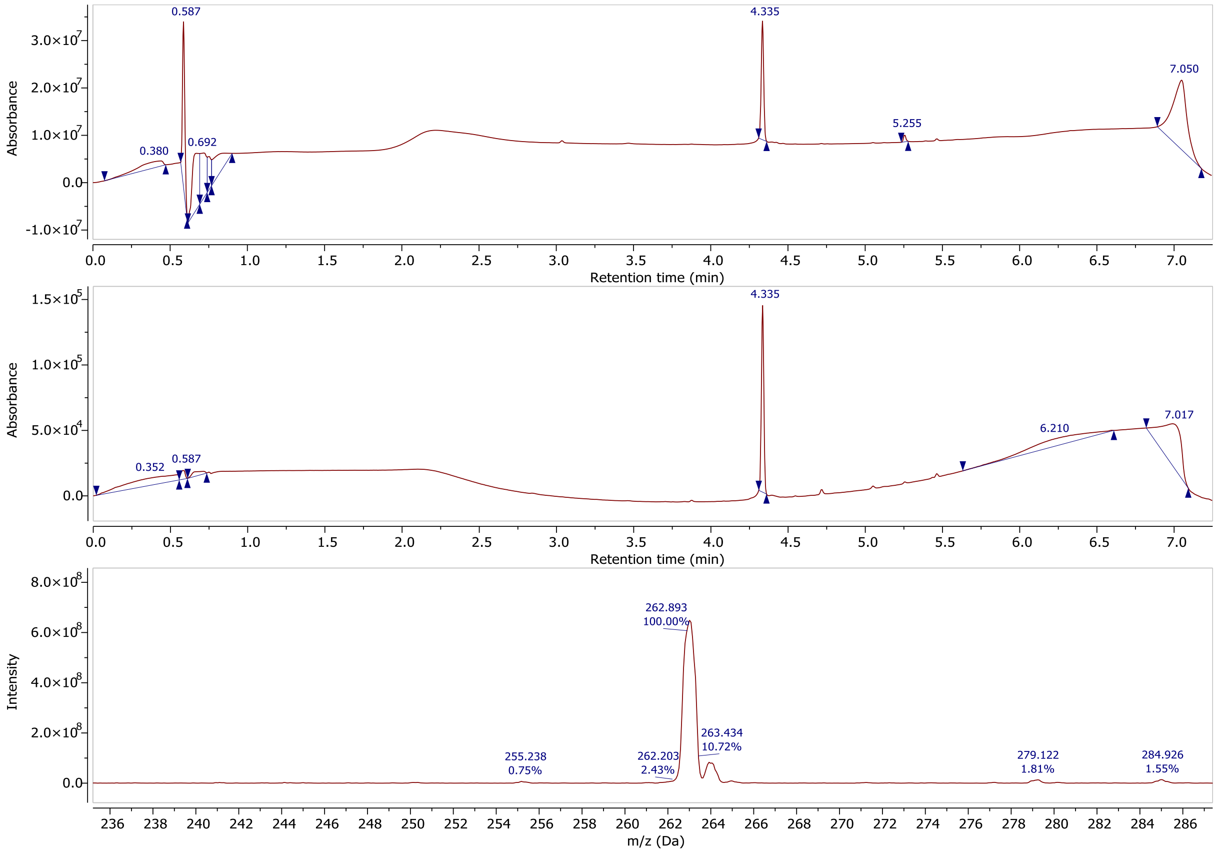

3.2.44 2-Hydroxy-6-iodobenzaldehyde (34c)

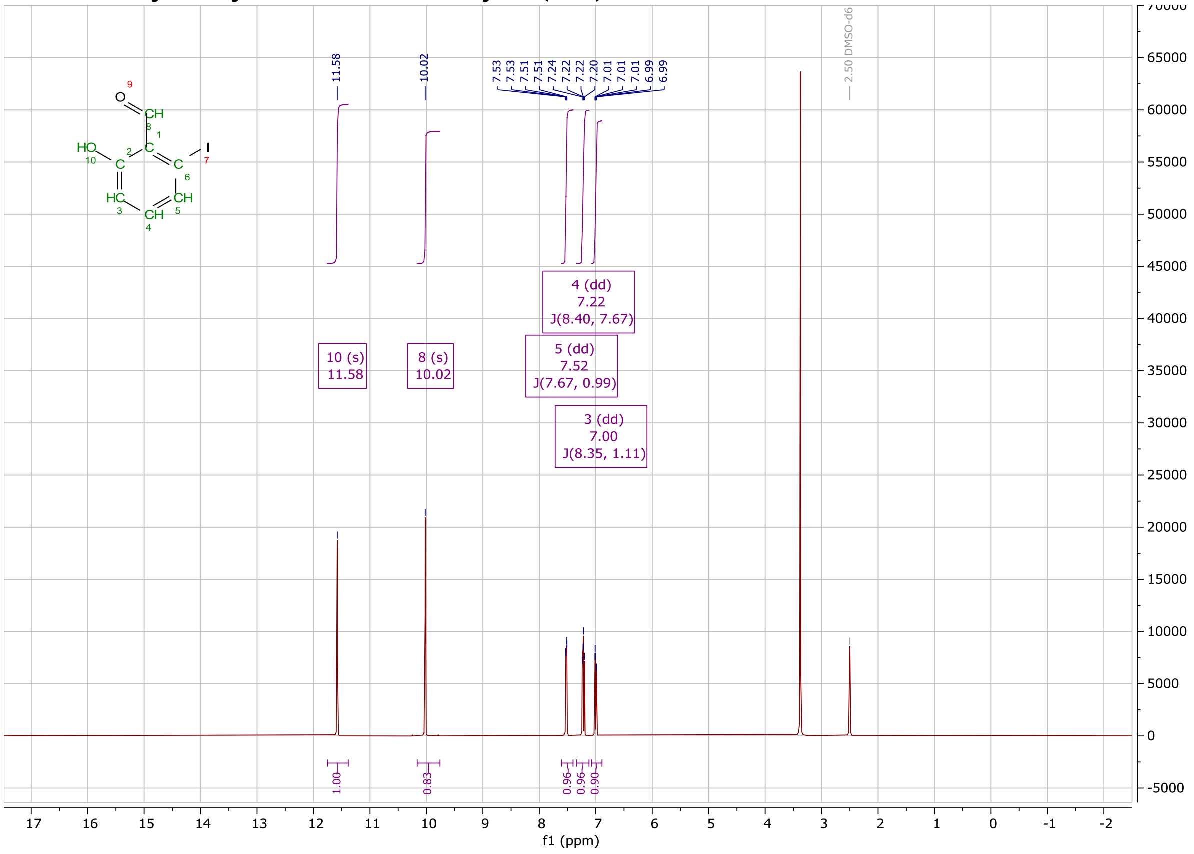

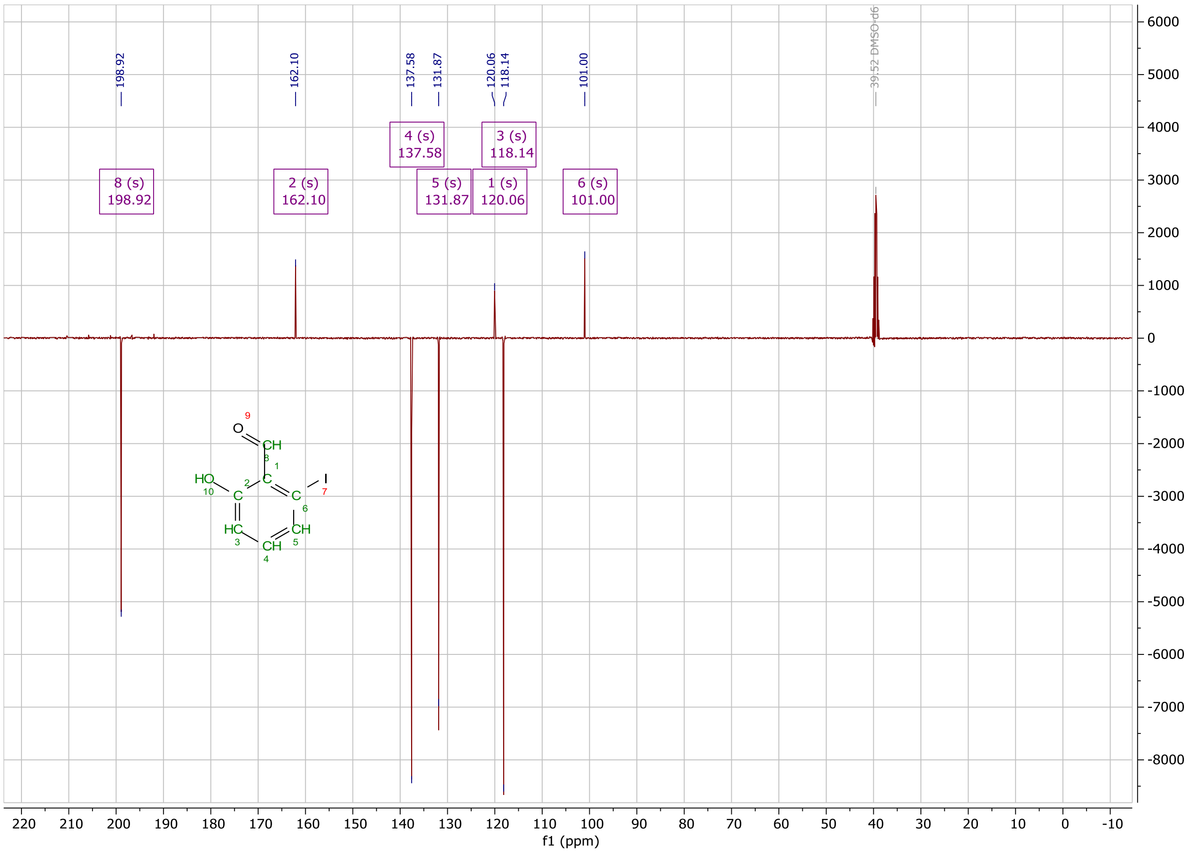
198.92
162.10
137.58
131.87
120.06
118.14
101.00
39.52 DMSO-d6
8 (s) 198.92
2 (s) 162.10
4 (s) 137.58
5 (s) 131.87
1 (s) 120.06
3 (s) 118.14
6 (s) 101.00
f1 (ppm)

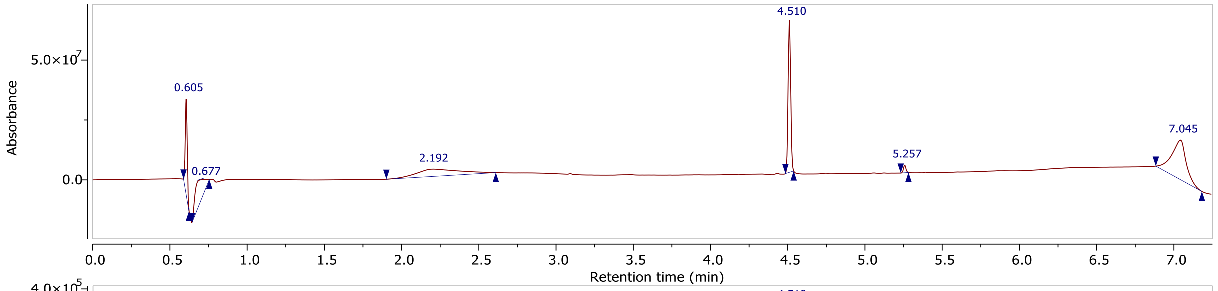
Absorbance
0.605
0.677
2.192
4.510
5.257
7.045
Retention time (min)

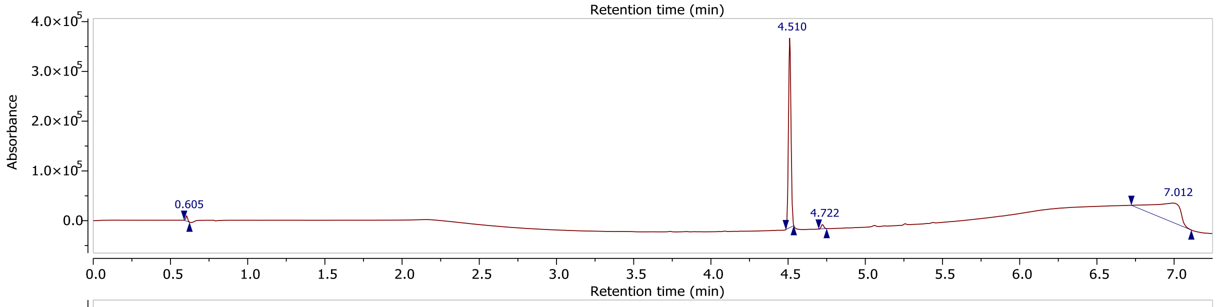
Absorbance
0.605
4.510
4.722
7.012
Retention time (min)

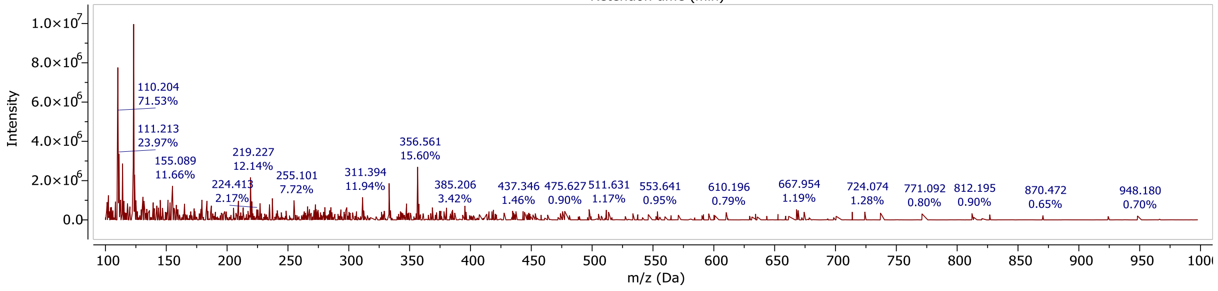
Intensity
110.204 71.53%
111.213 23.97%
155.089 11.66%
219.227 12.14%
224.413 2.17%
255.101 7.72%
311.394 11.94%
356.561 15.60%
385.206 3.42%
437.346 1.46%
475.627 0.90%
511.631 1.17%
553.641 0.95%
610.196 0.79%
667.954 1.19%
724.074 1.28%
771.092 0.80%
812.195 0.90%
870.472 0.65%
948.180 0.70%
m/z (Da)

3.2.45 Diethyl (*E*)-(4-hydroxy-2-iodostyryl)phosphonate (42a)

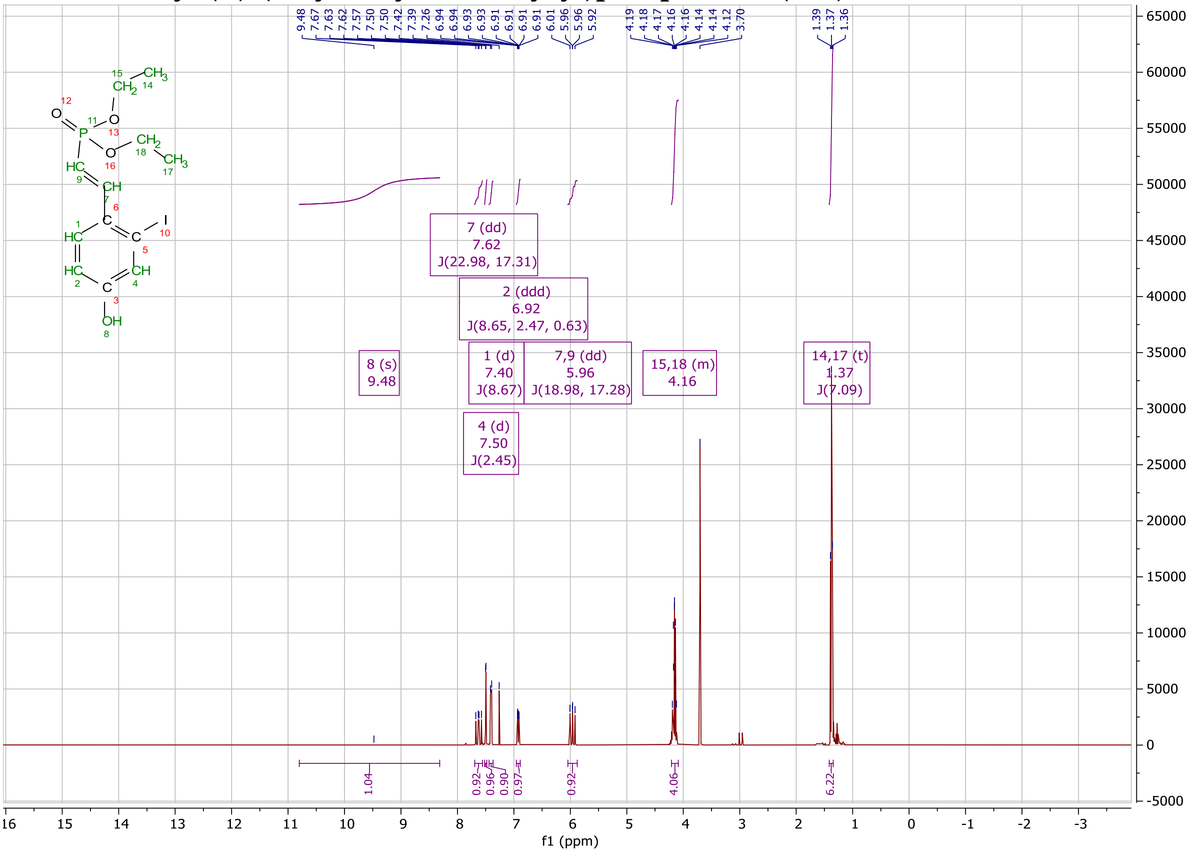

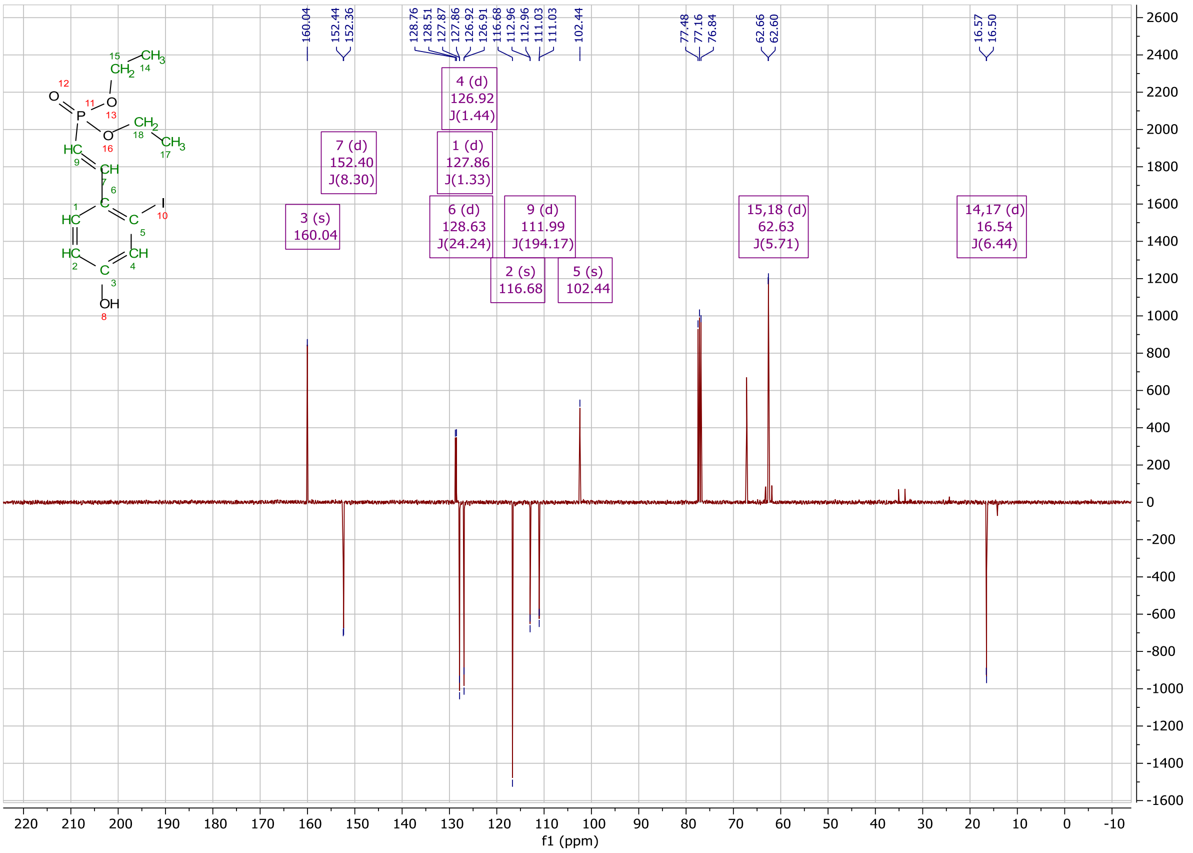

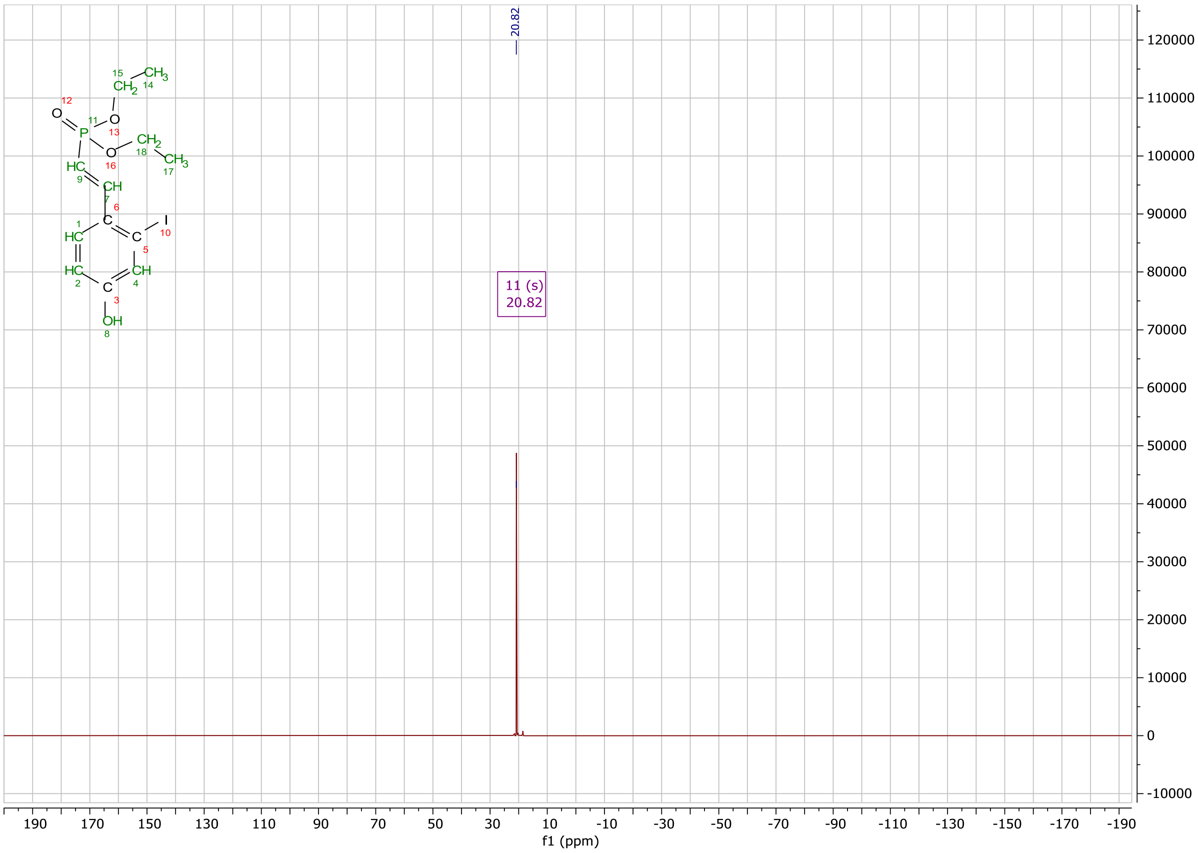

20.82
11 (s)
20.82
f1 (ppm)

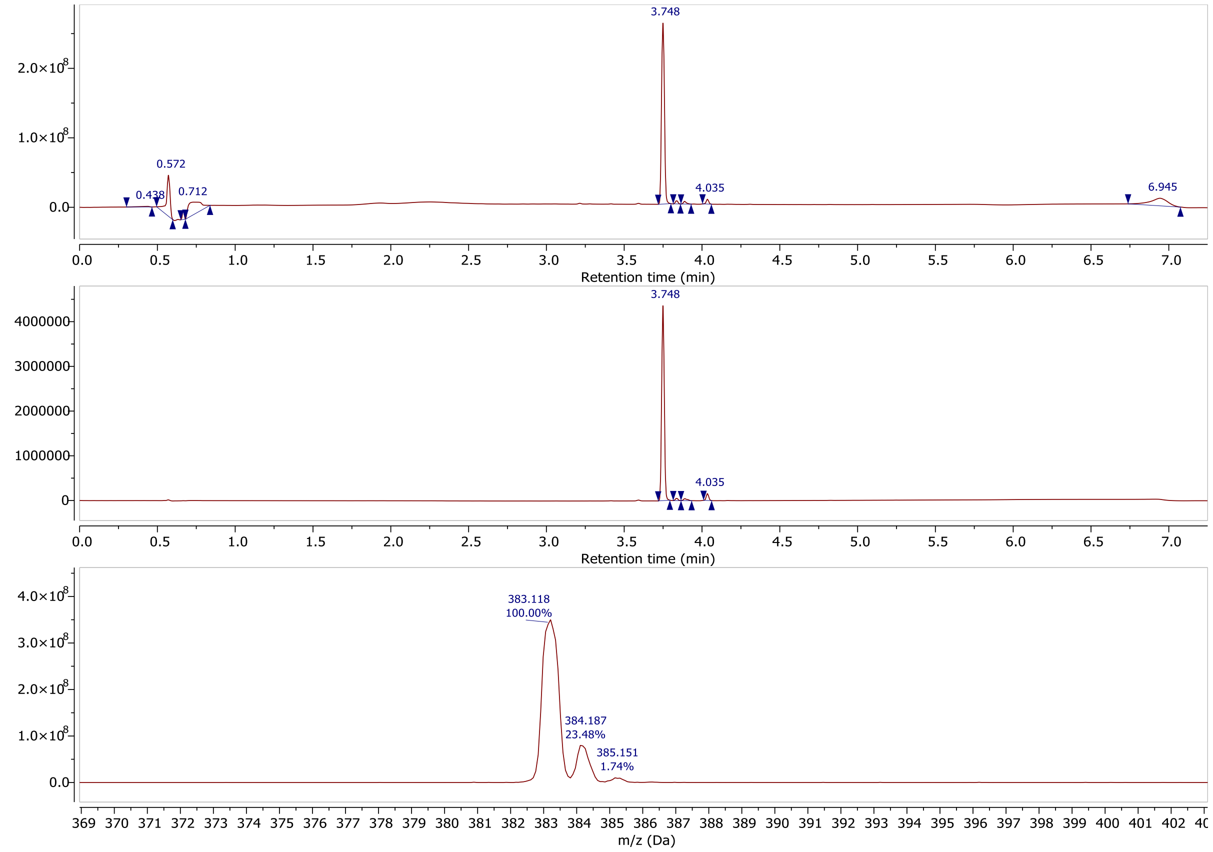

3.748
0.572
0.438
0.712
4.035
6.945
Retention time (min)
3.748
4.035
Retention time (min)
383.118
100.00%
384.187
23.48%
385.151
1.74%
m/z (Da)

3.2.46 Diethyl (*E*)-(5-hydroxy-2-iodostyryl)phosphonate (42b)

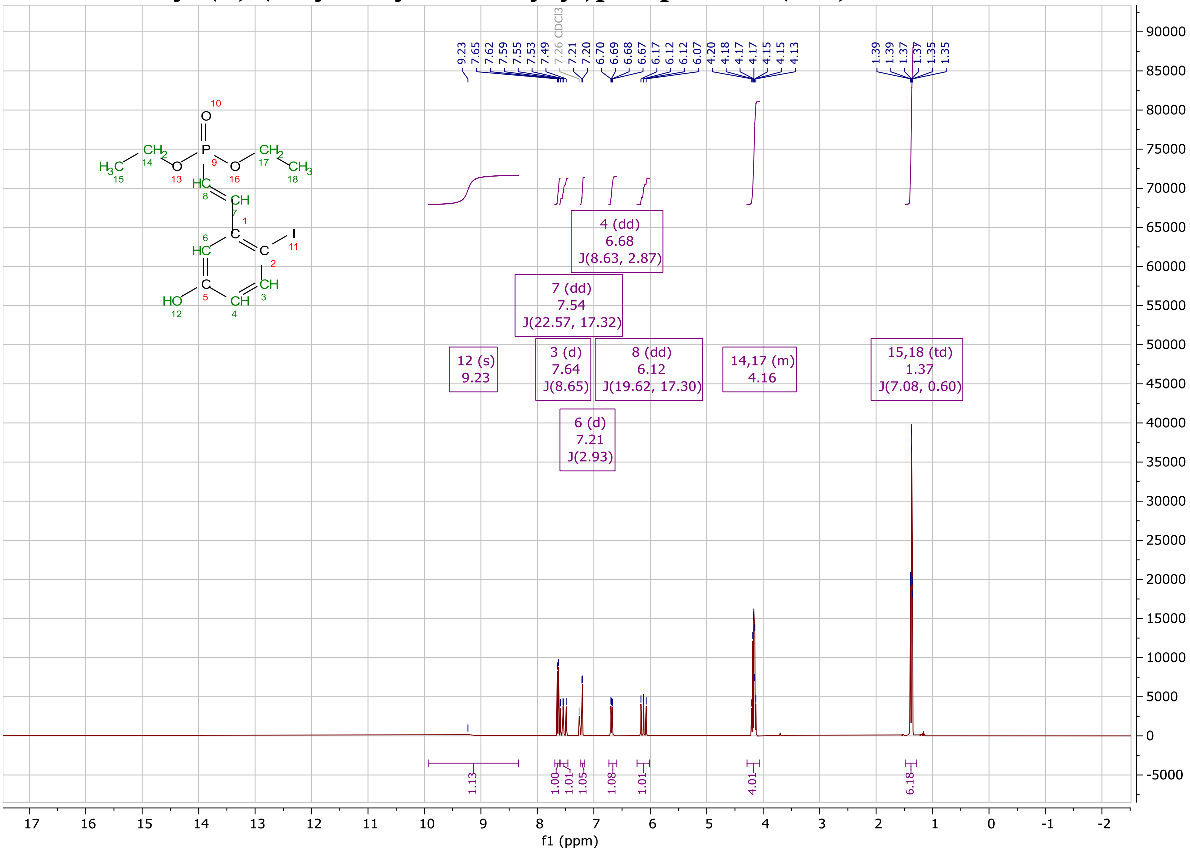

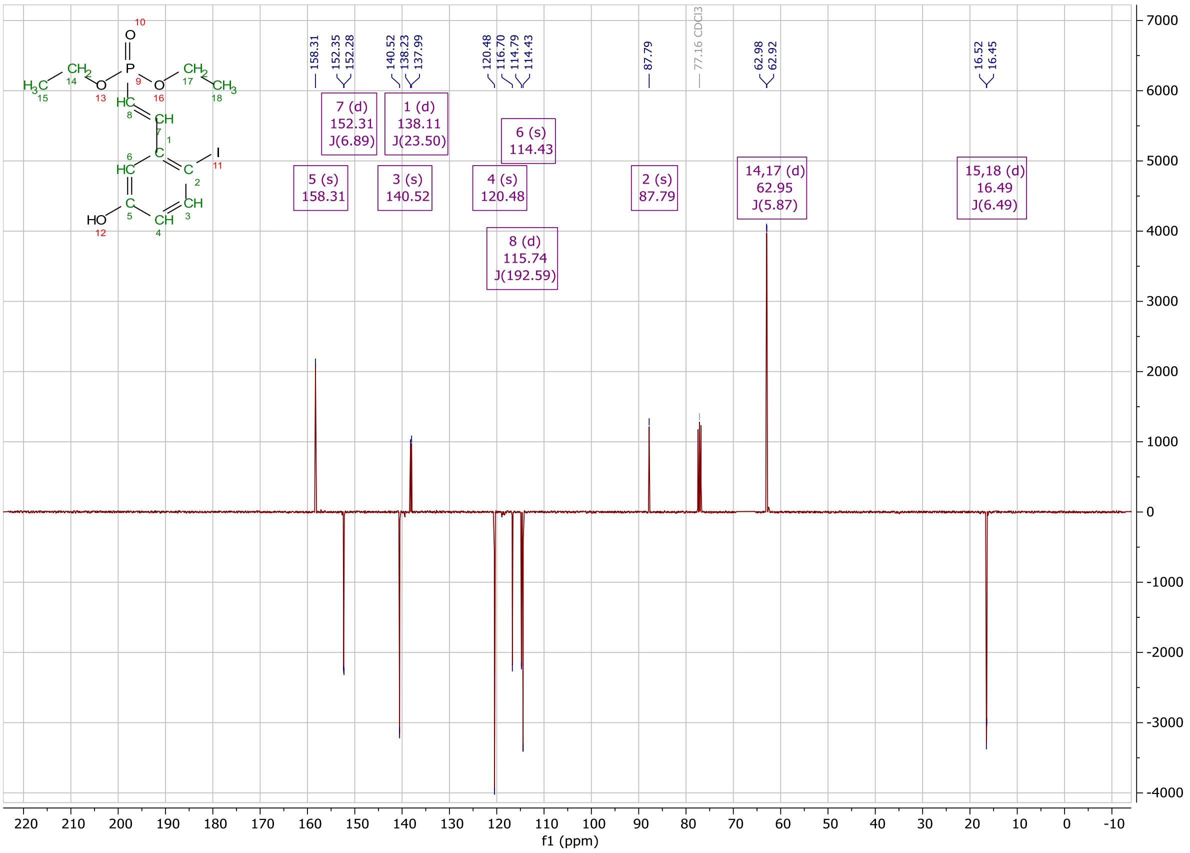

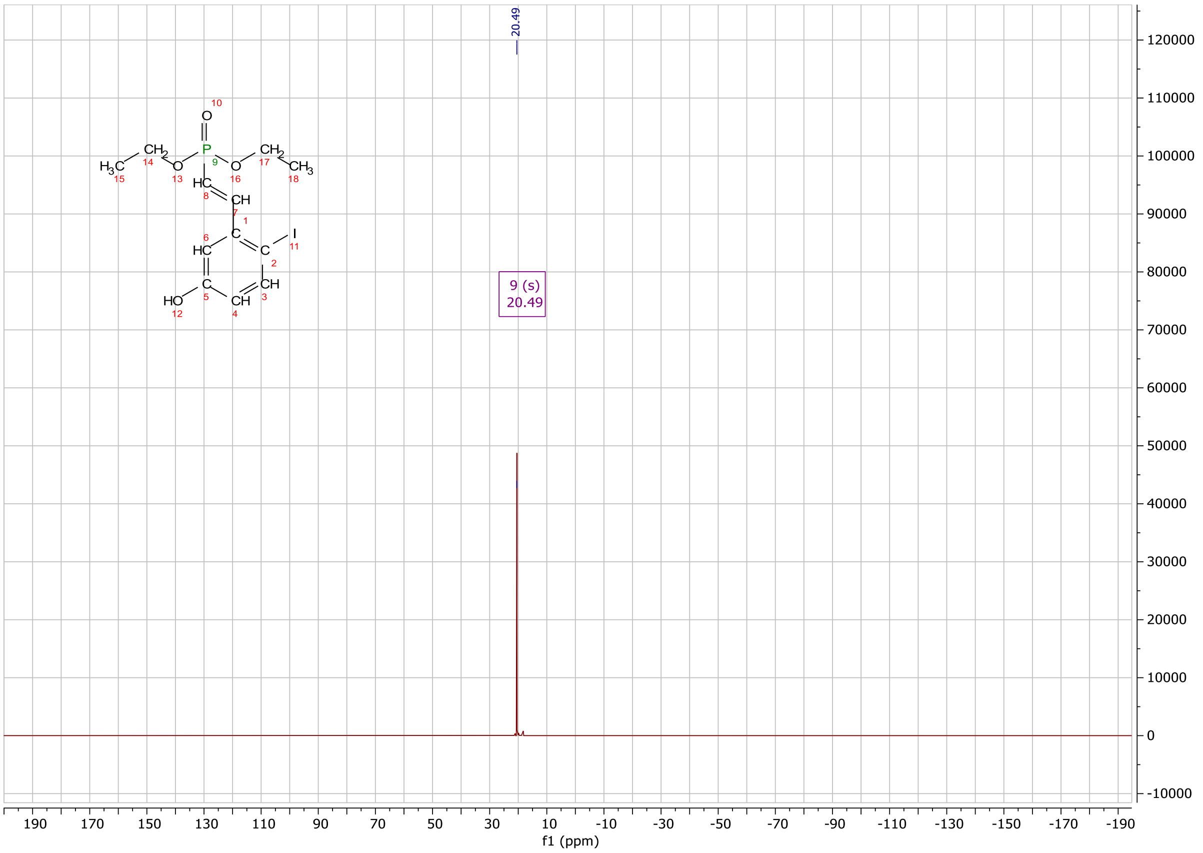
20.49
9 (s)
20.49
f1 (ppm)

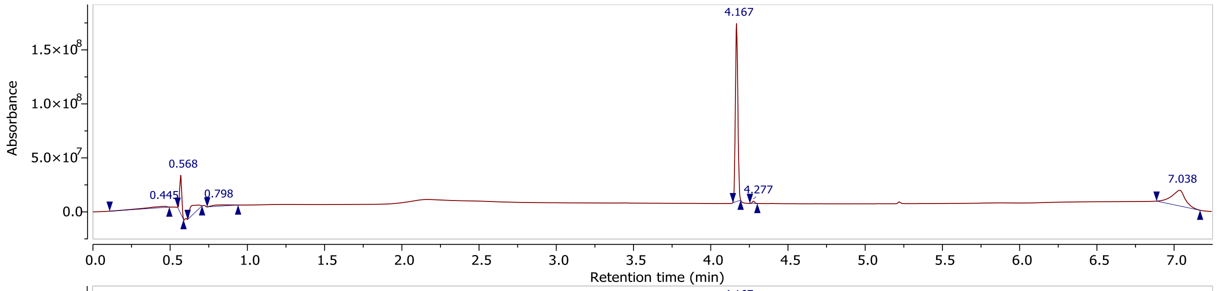
4.167
0.568
0.445
0.798
4.277
7.038
Absorbance
Retention time (min)

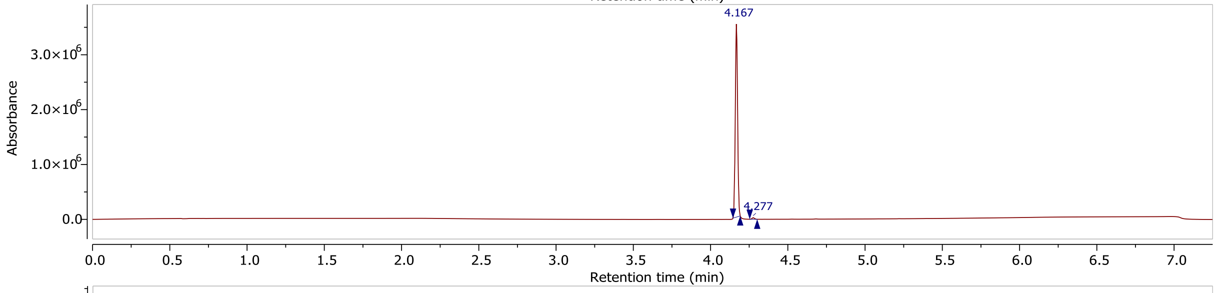
4.167
4.277
Absorbance
Retention time (min)

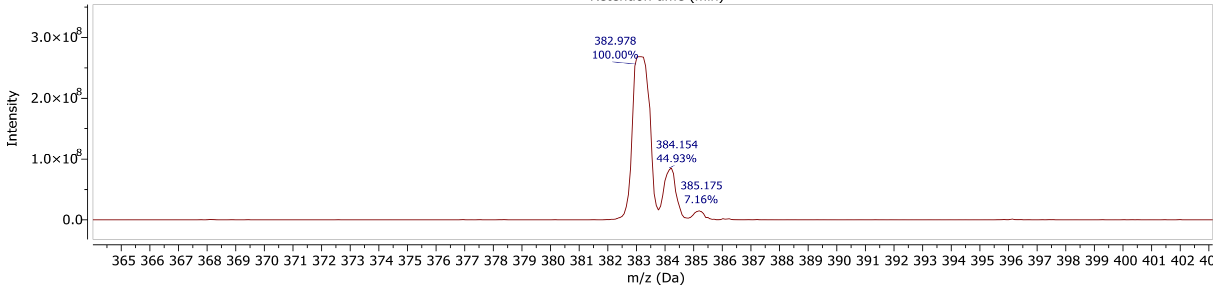
382.978
100.00%
384.154
44.93%
385.175
7.16%
Intensity
m/z (Da)

3.2.47 Diethyl (*E*)-(2-hydroxy-6-iodostyryl)phosphonate (42c)

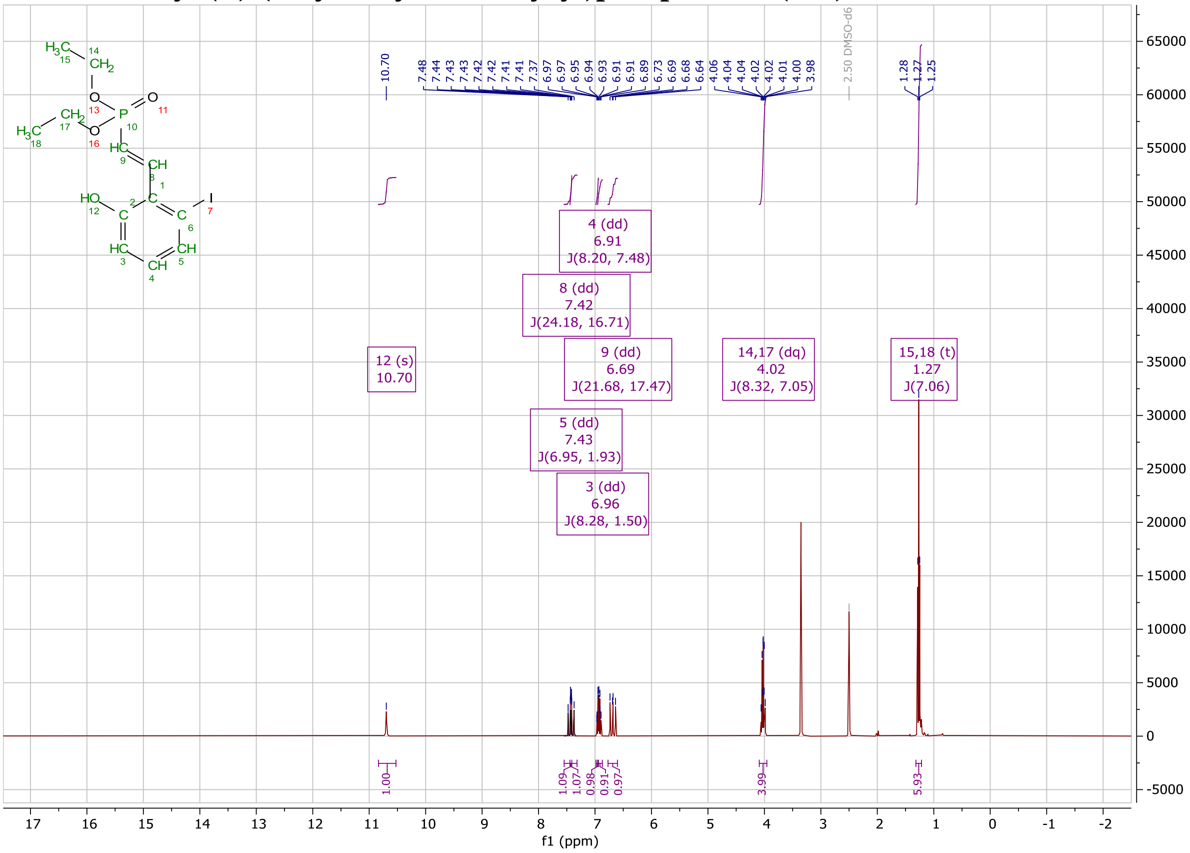

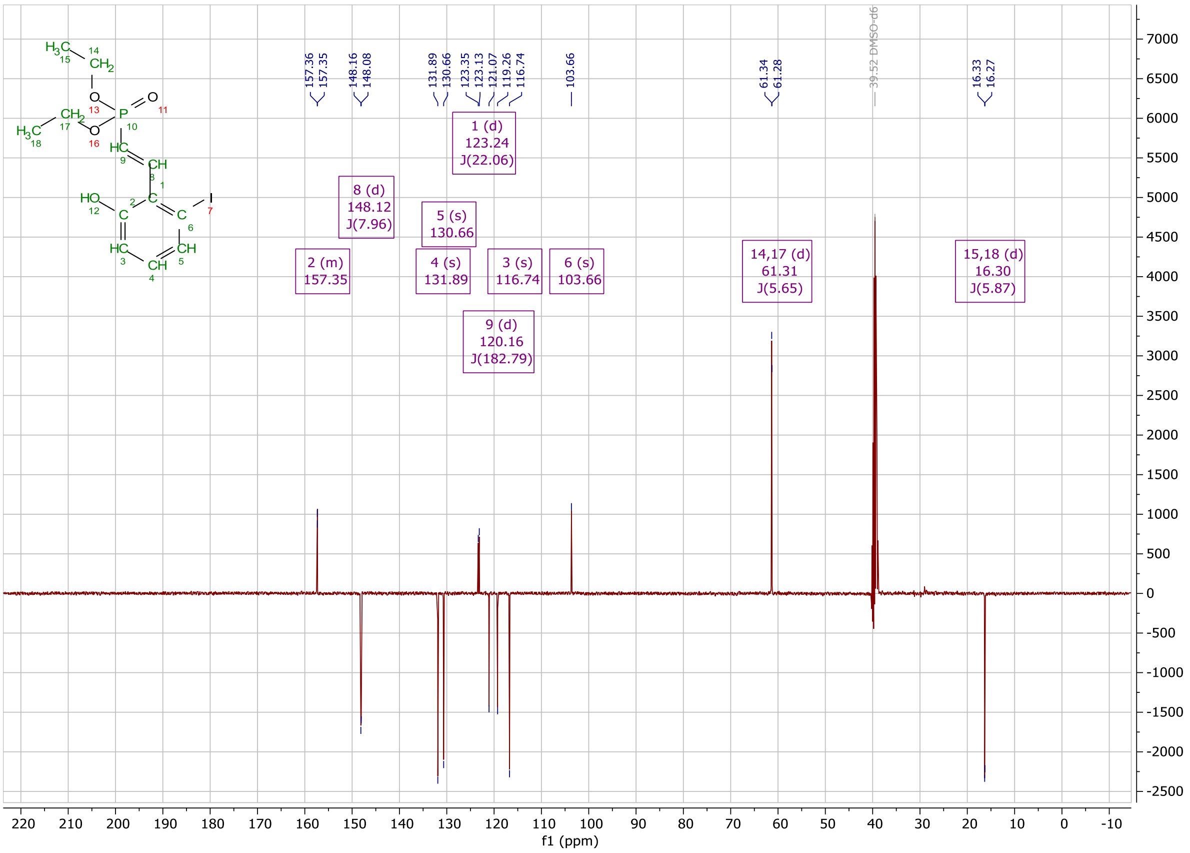

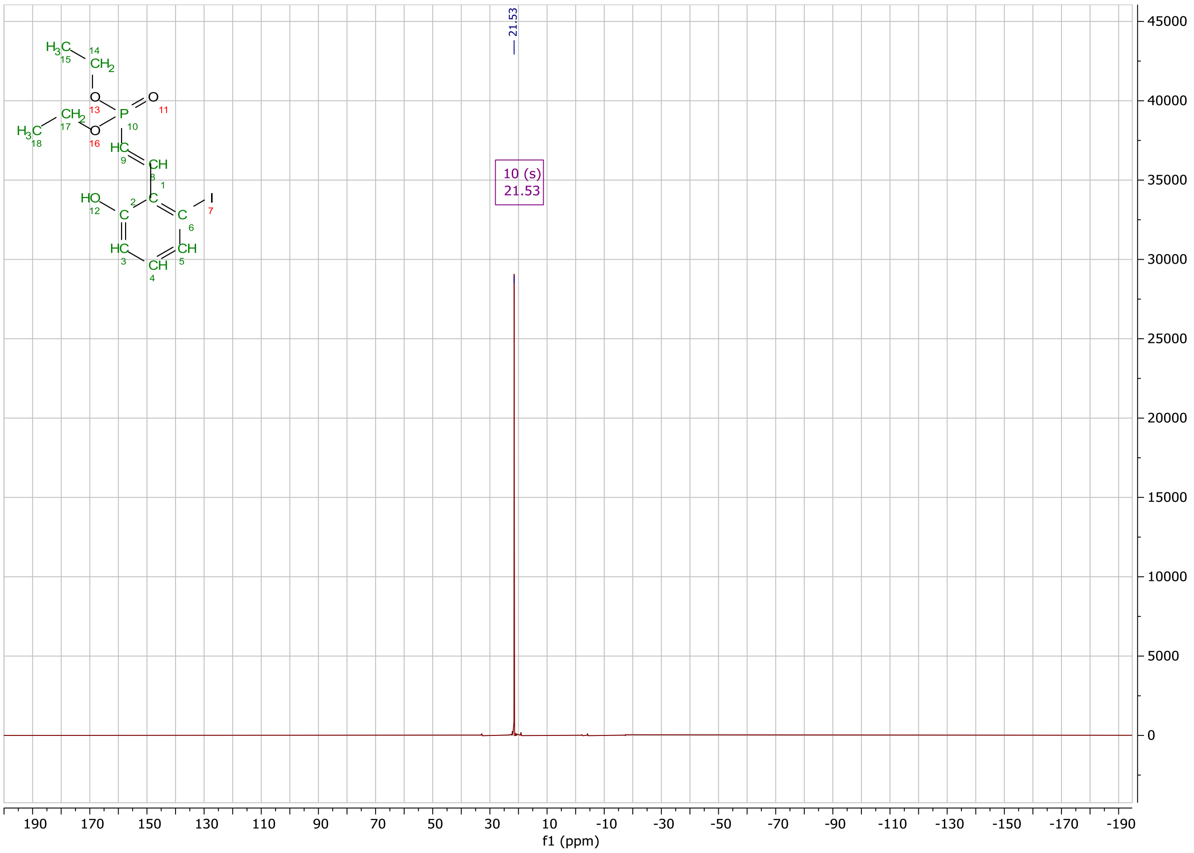

21.53
10 (s)
21.53
f1 (ppm)

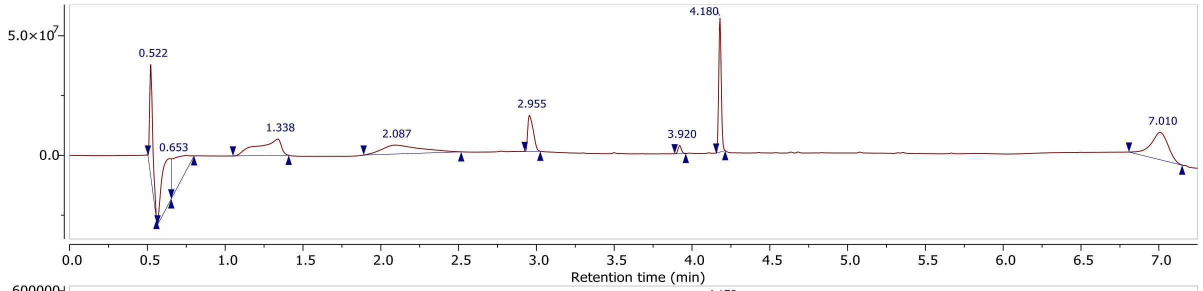

0.522
0.653
1.338
2.087
2.955
3.920
4.180
7.010
Retention time (min)

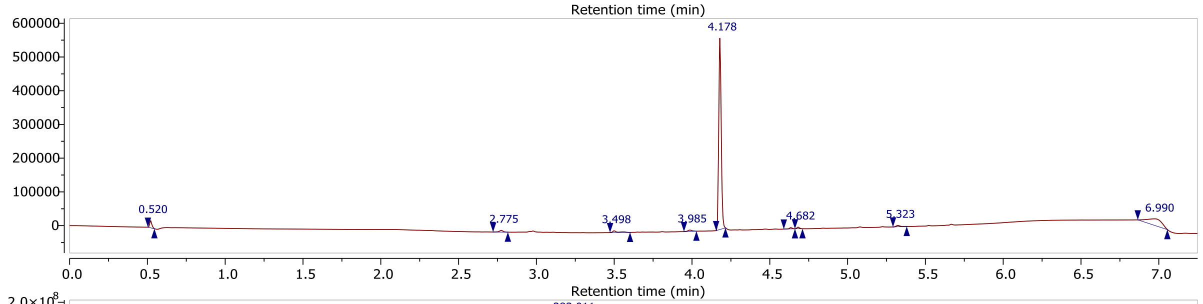

0.520
2.775
3.498
3.985
4.178
4.682
5.323
6.990
Retention time (min)

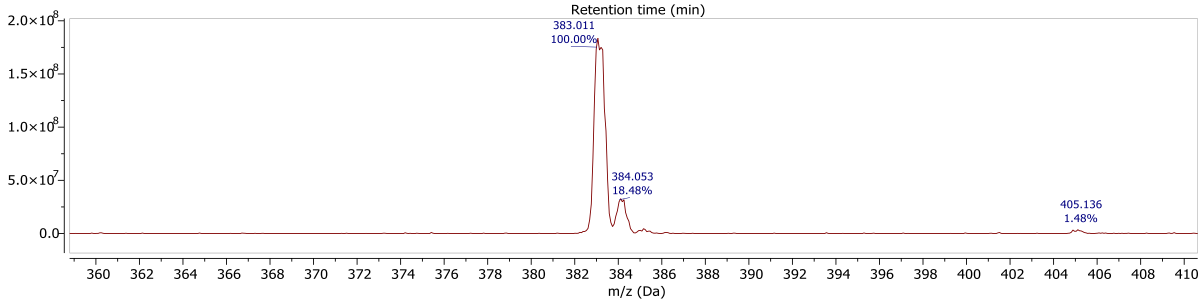

383.011
100.00%
384.053
18.48%
405.136
1.48%
m/z (Da)

3.2.48 Diethyl (*E*)-(2-iodo-4-methoxystyryl)phosphonate (43a)

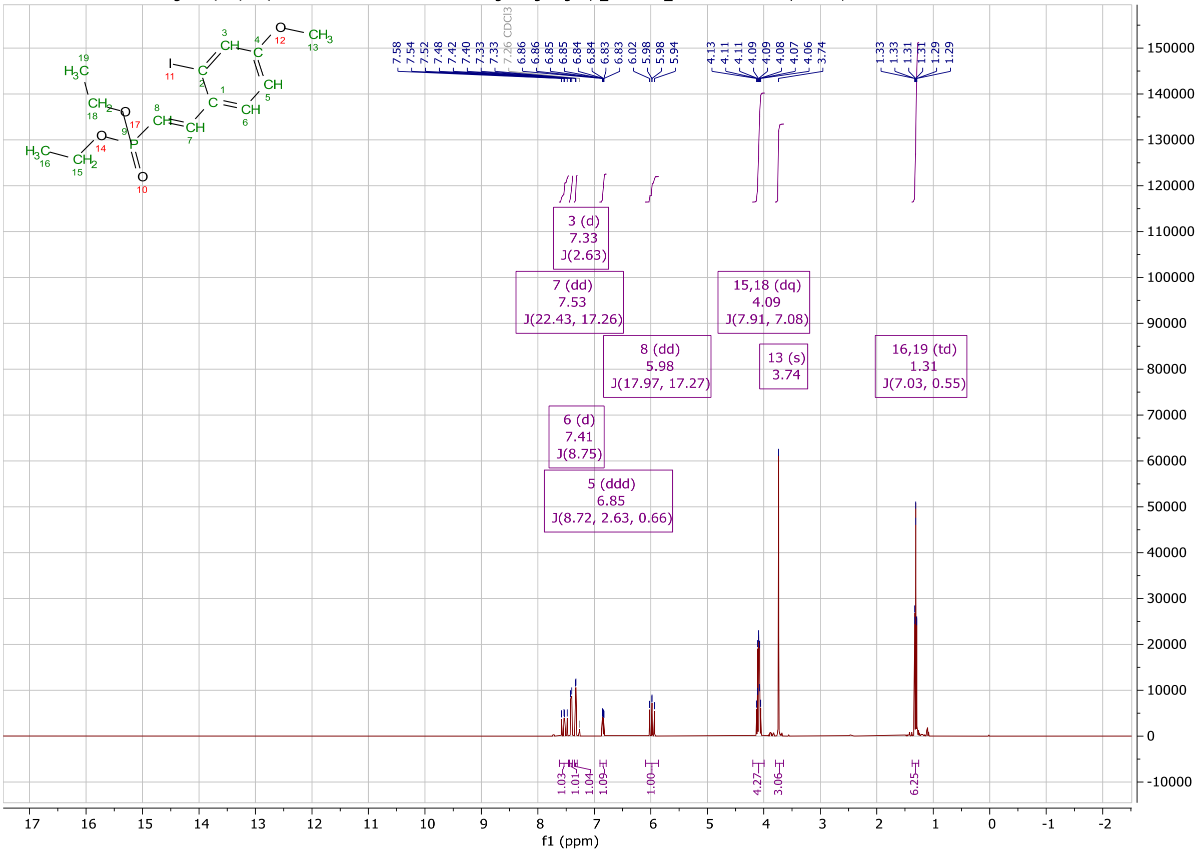

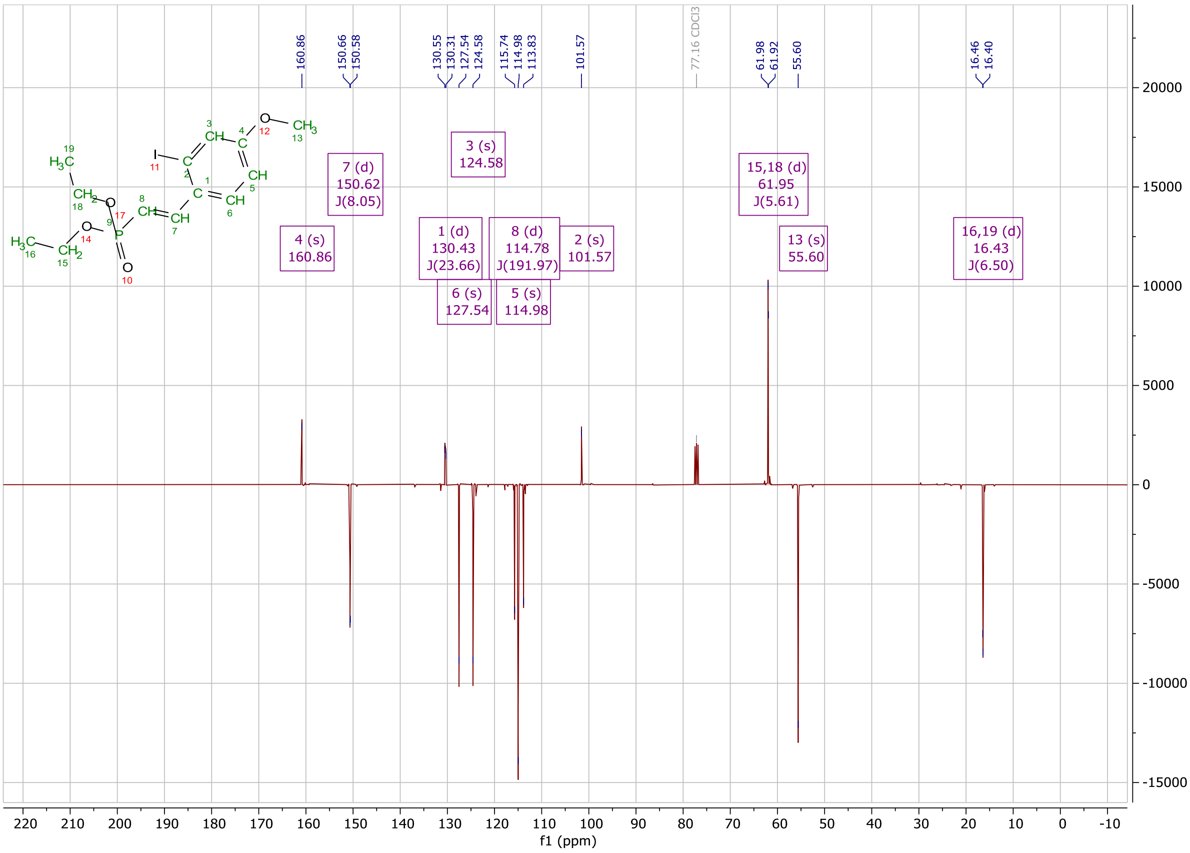

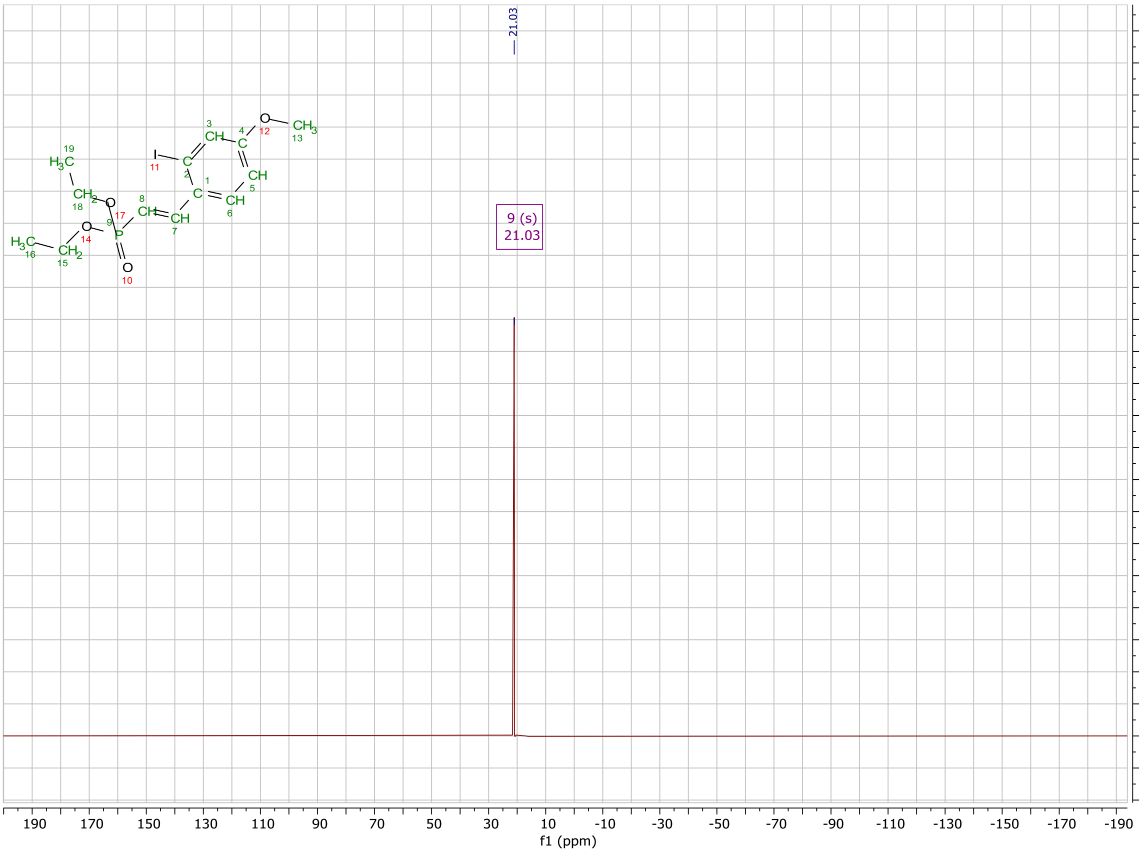
21.03
9 (s)
21.03
f1 (ppm)

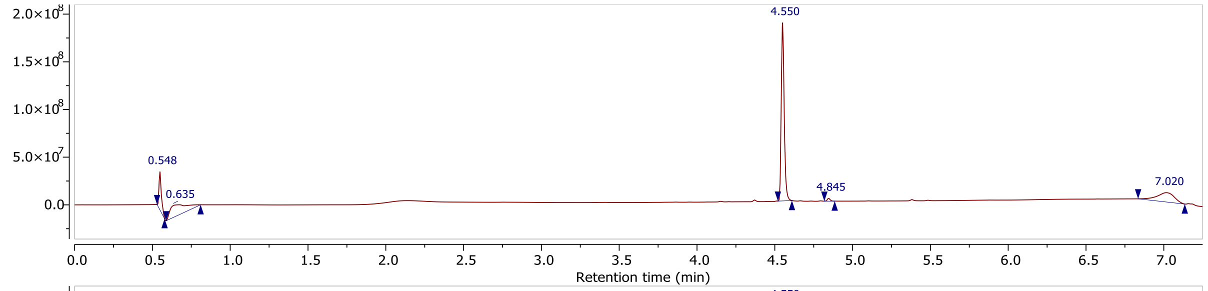
0.548
0.635
4.550
4.845
7.020
Retention time (min)

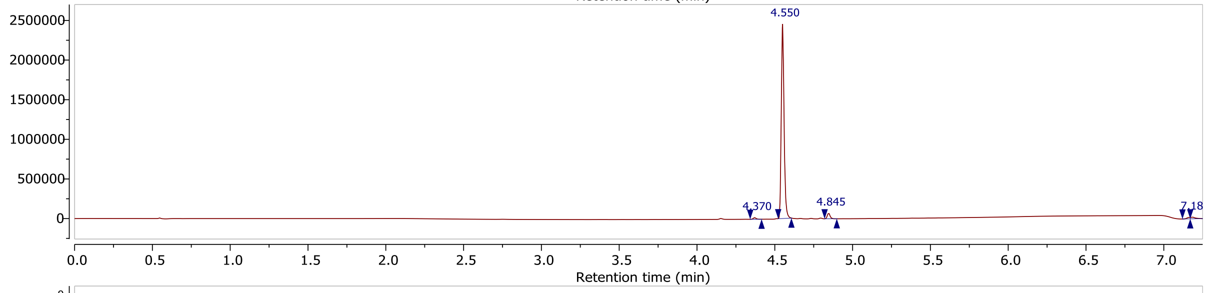
4.550
4.370
4.845
Retention time (min)

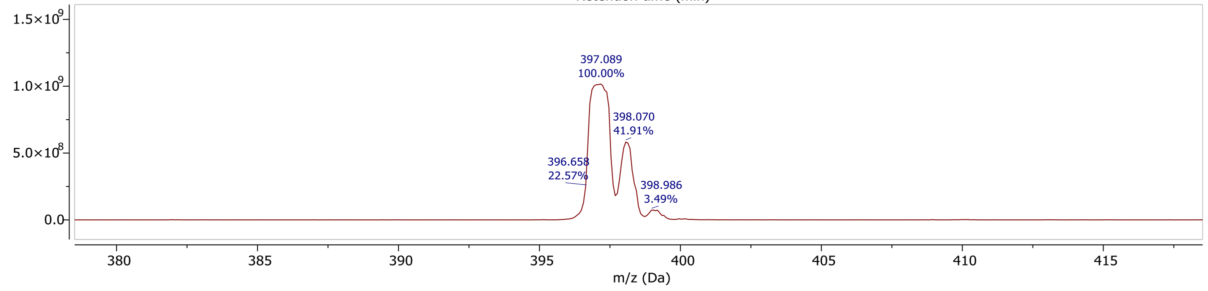
397.089
100.00%
398.070
41.91%
396.658
22.57%
398.986
3.49%
m/z (Da)

3.2.49 Diethyl (*E*)-(2-iodo-4-isopropoxystyryl)phosphonate (43b)

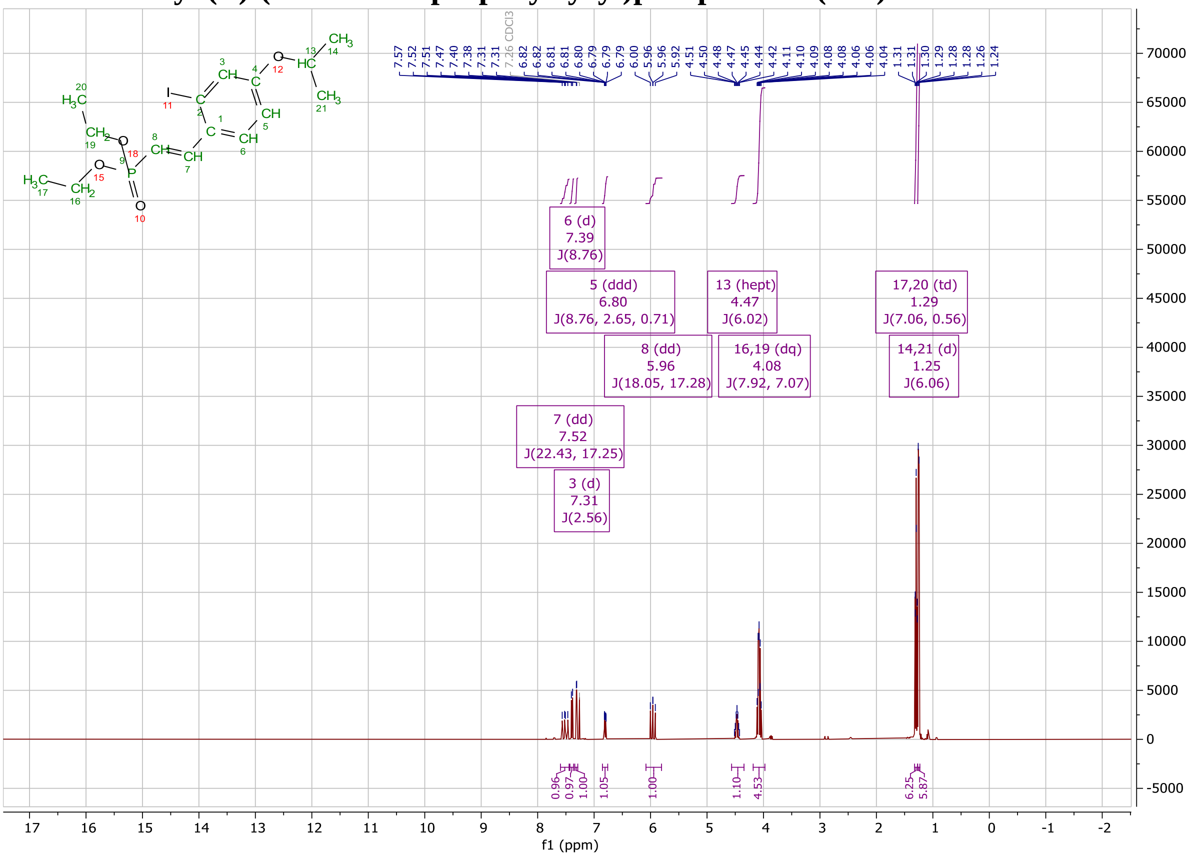

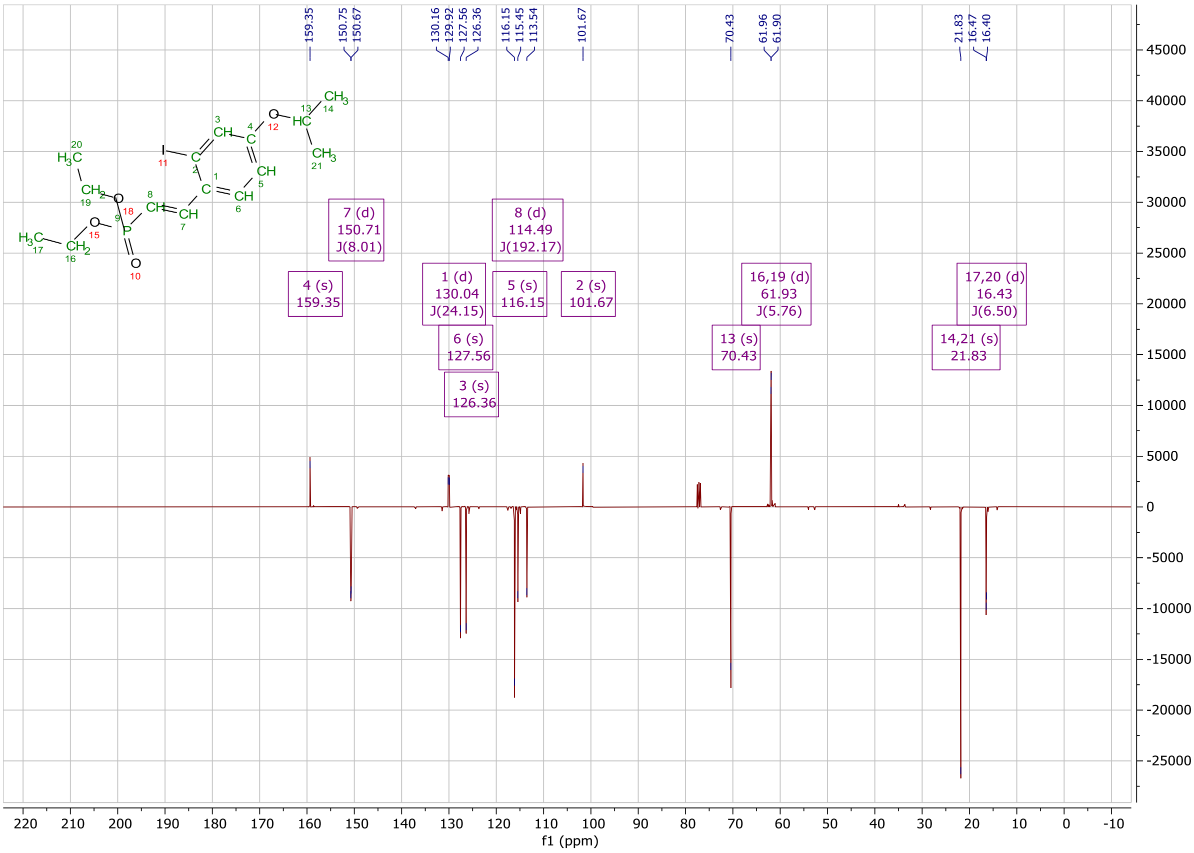

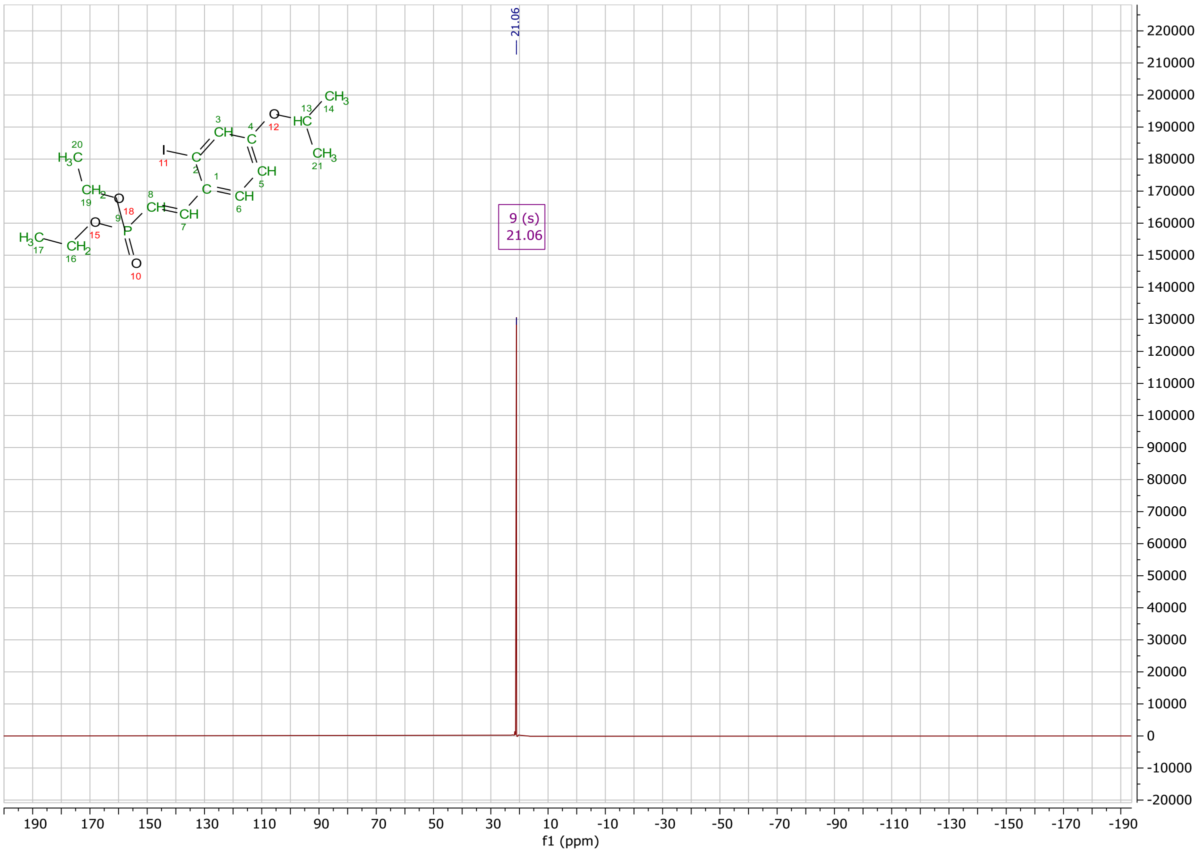
21.06
9 (s)
21.06
f1 (ppm)

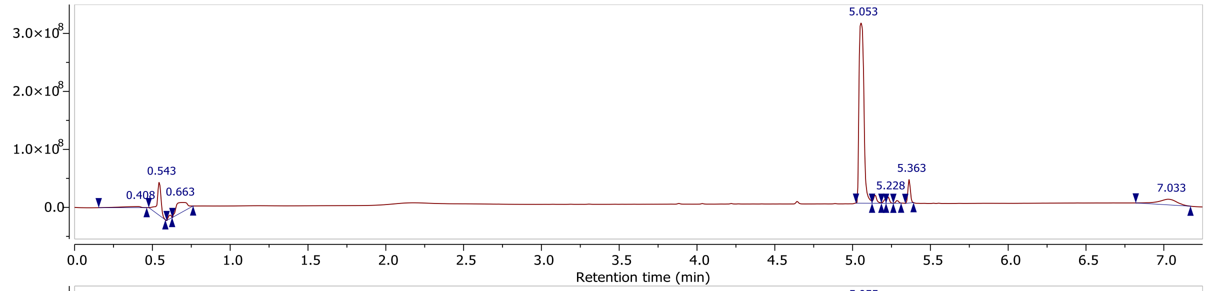
5.053
0.543
0.408
0.663
5.228
5.363
7.033
Retention time (min)

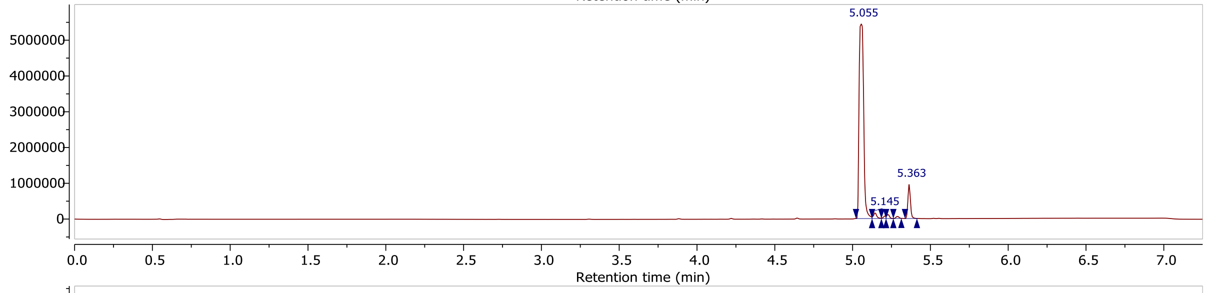
5.055
5.145
5.363
Retention time (min)

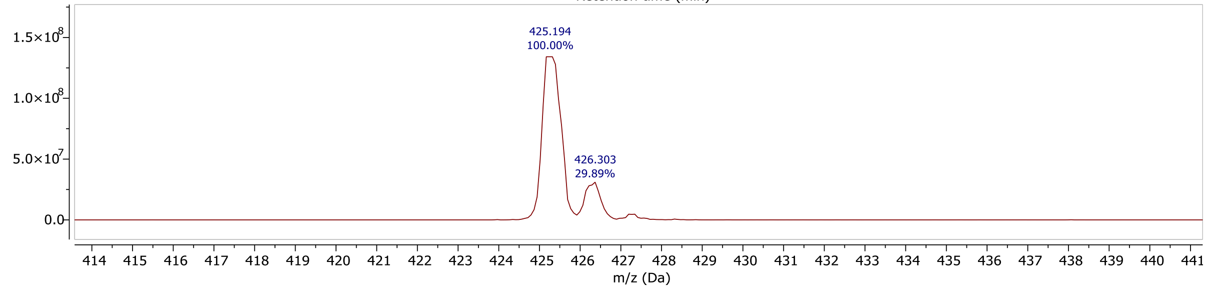
425.194
100.00%
426.303
29.89%
m/z (Da)

3.2.50 Diethyl (*E*)-(2-iodo-4-((2,3,4,5,6-pentafluorophenyl)oxy)styryl)phosphonate (43c)

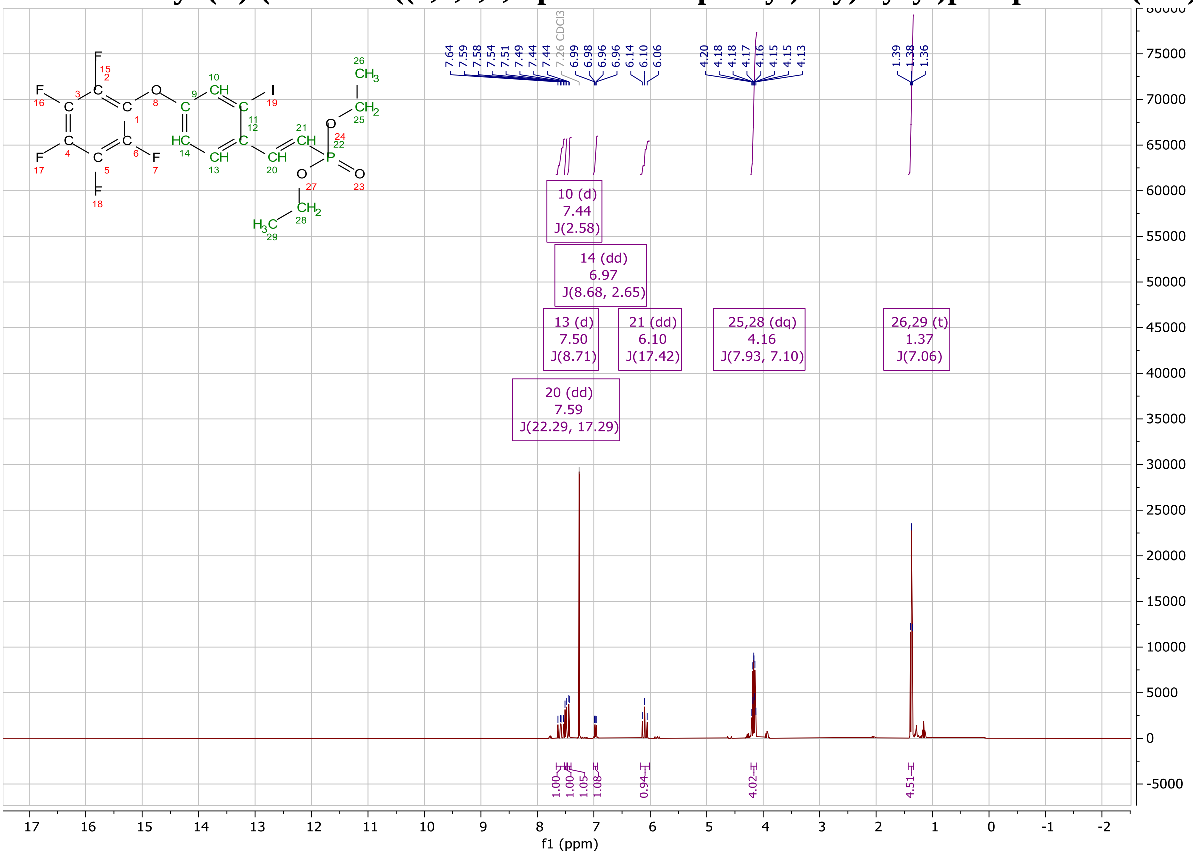

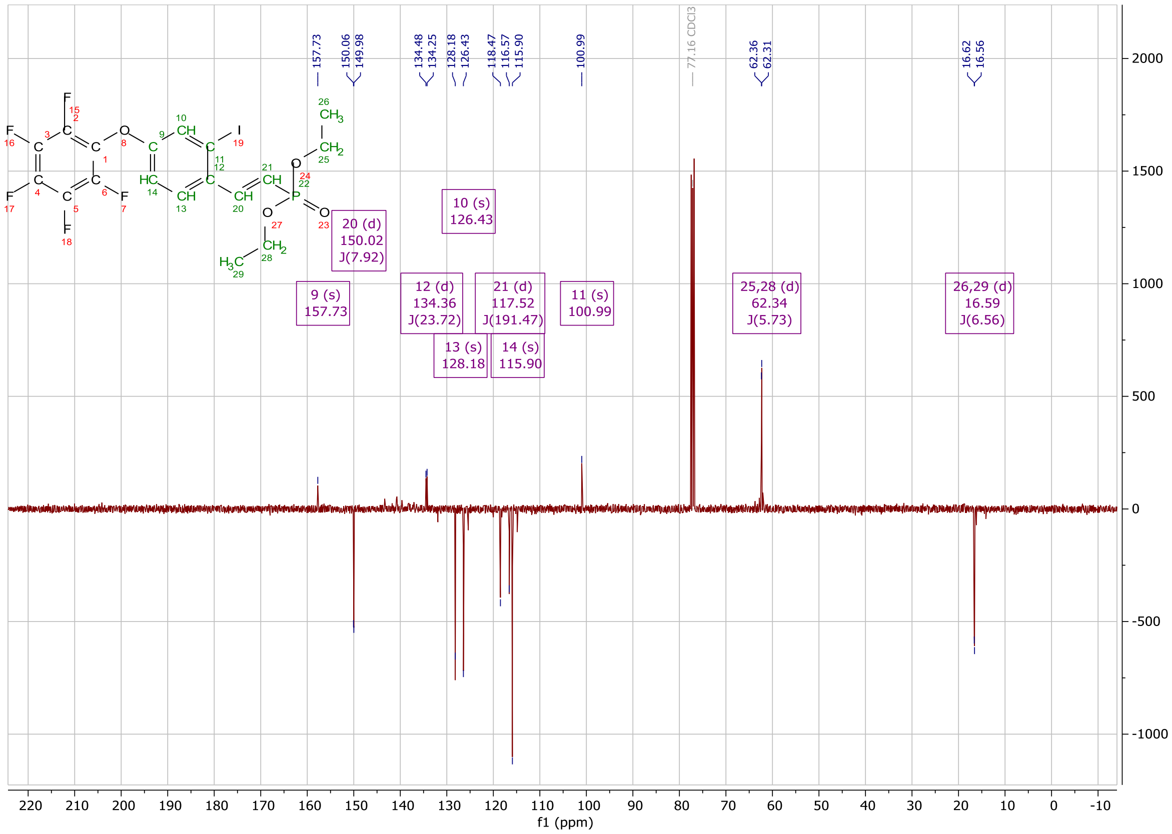

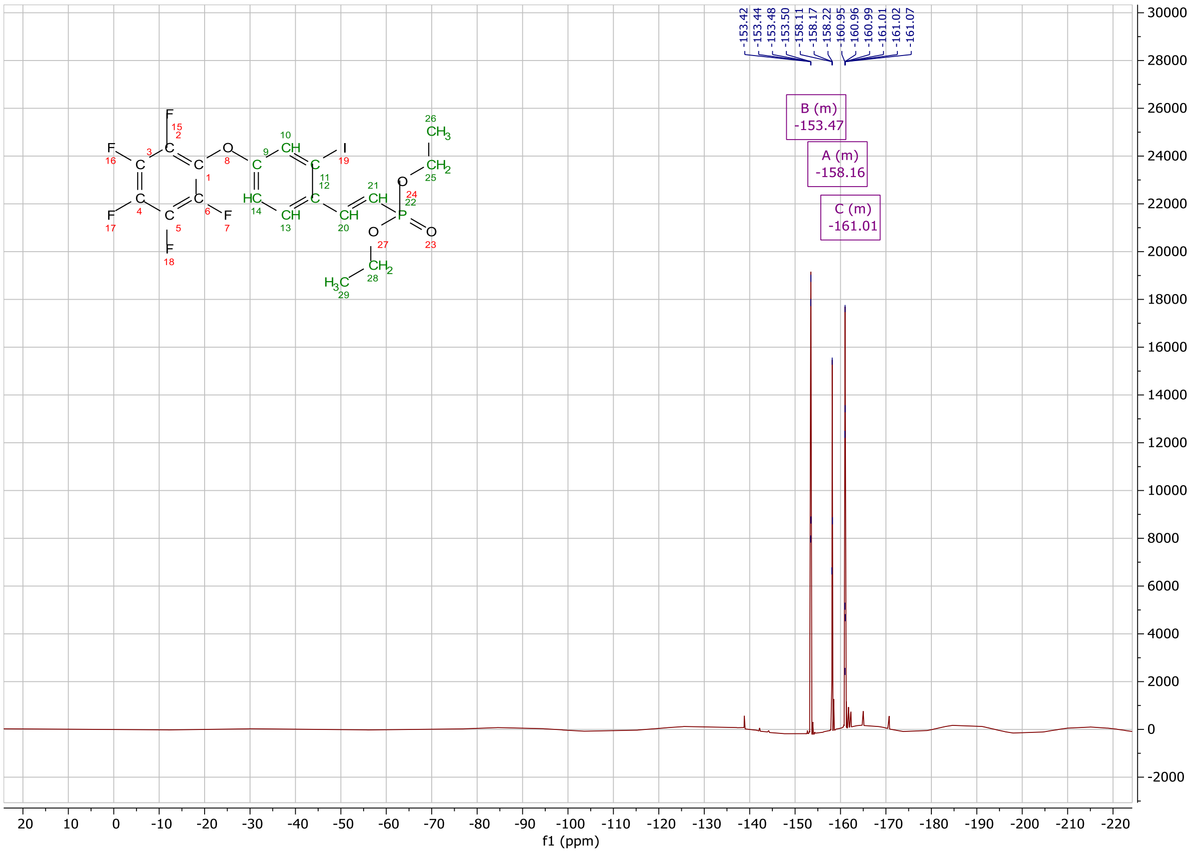
-153.42
-153.44
-153.48
-153.50
-158.11
-158.17
-158.22
-160.95
-160.96
-160.99
-161.01
-161.02
-161.07
B (m)
-153.47
A (m)
-158.16
C (m)
-161.01
f1 (ppm)

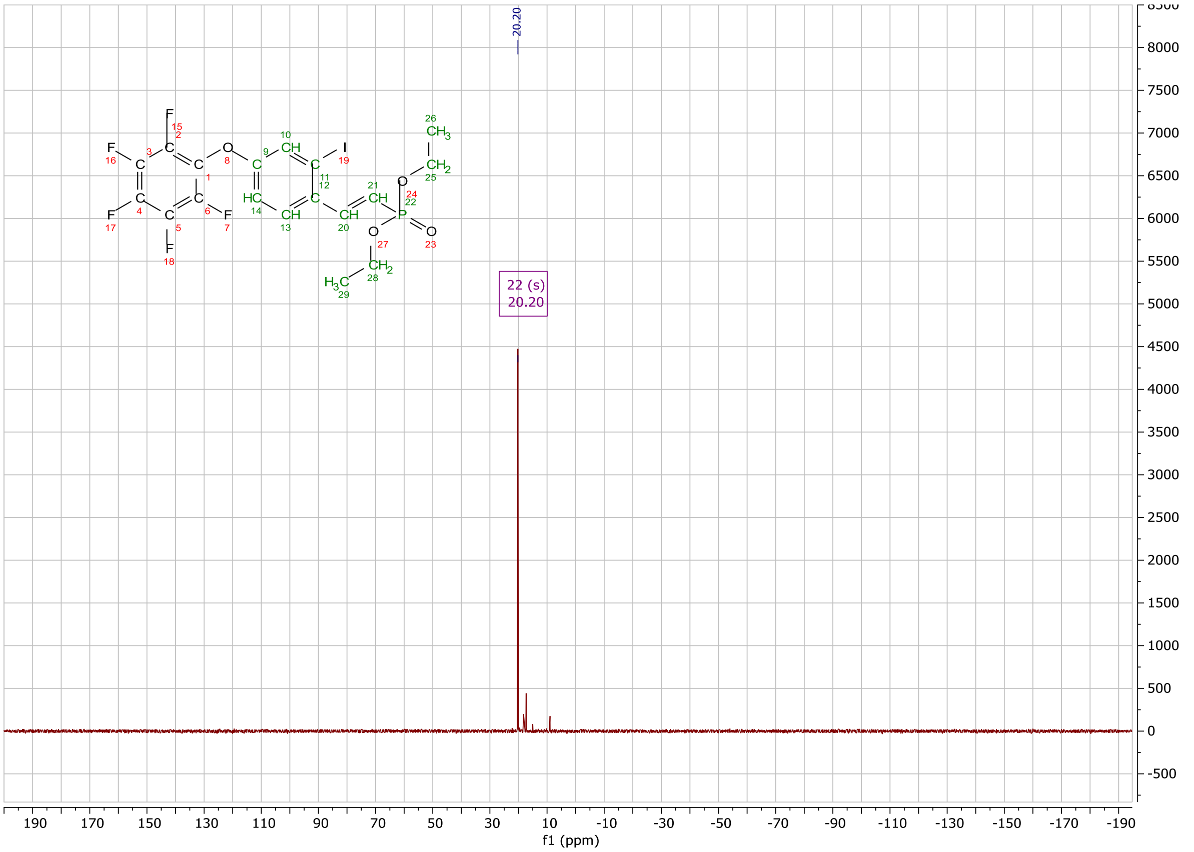
20.20
22 (s)
20.20
f1 (ppm)

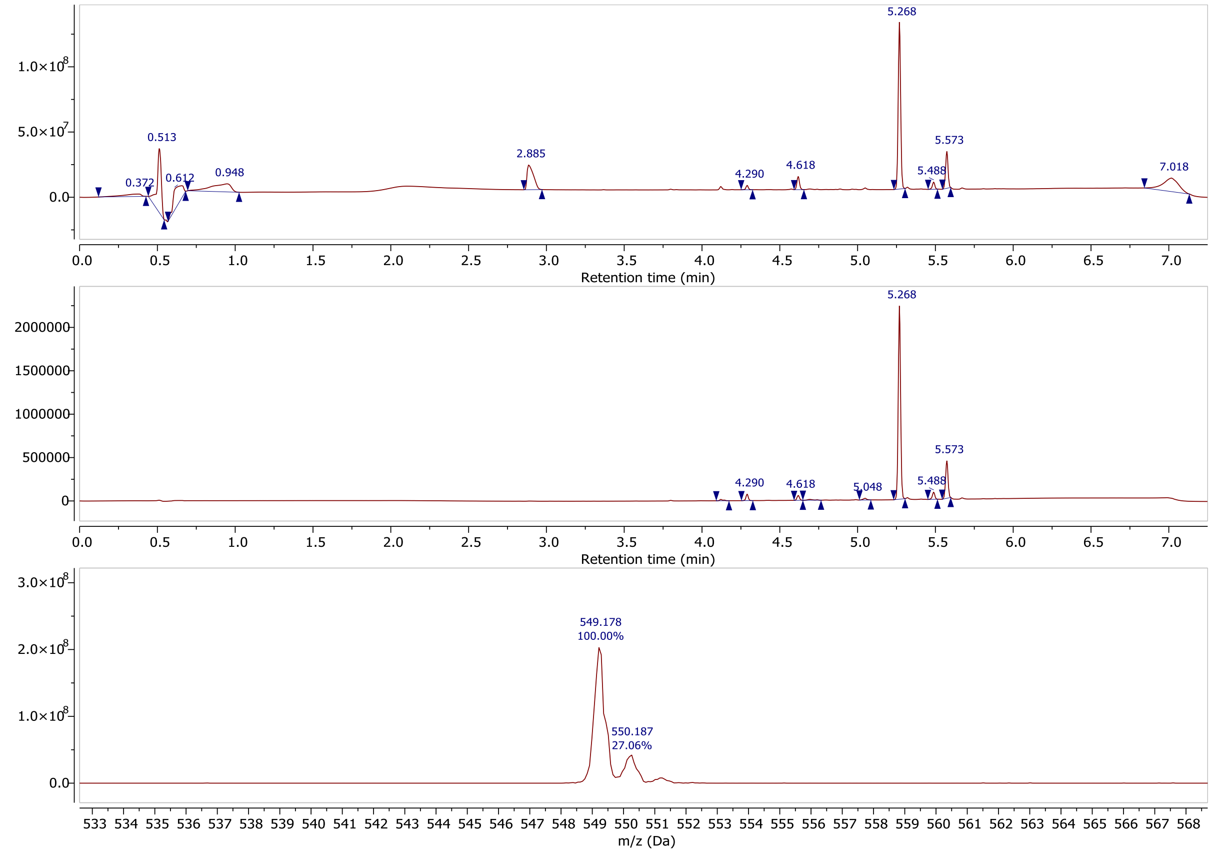

3.2.51 Diethyl (*E*)-(2-iodo-4-((2,3,4,5,6-pentafluorophenyl)methoxy)styryl)phosphonate (43d)

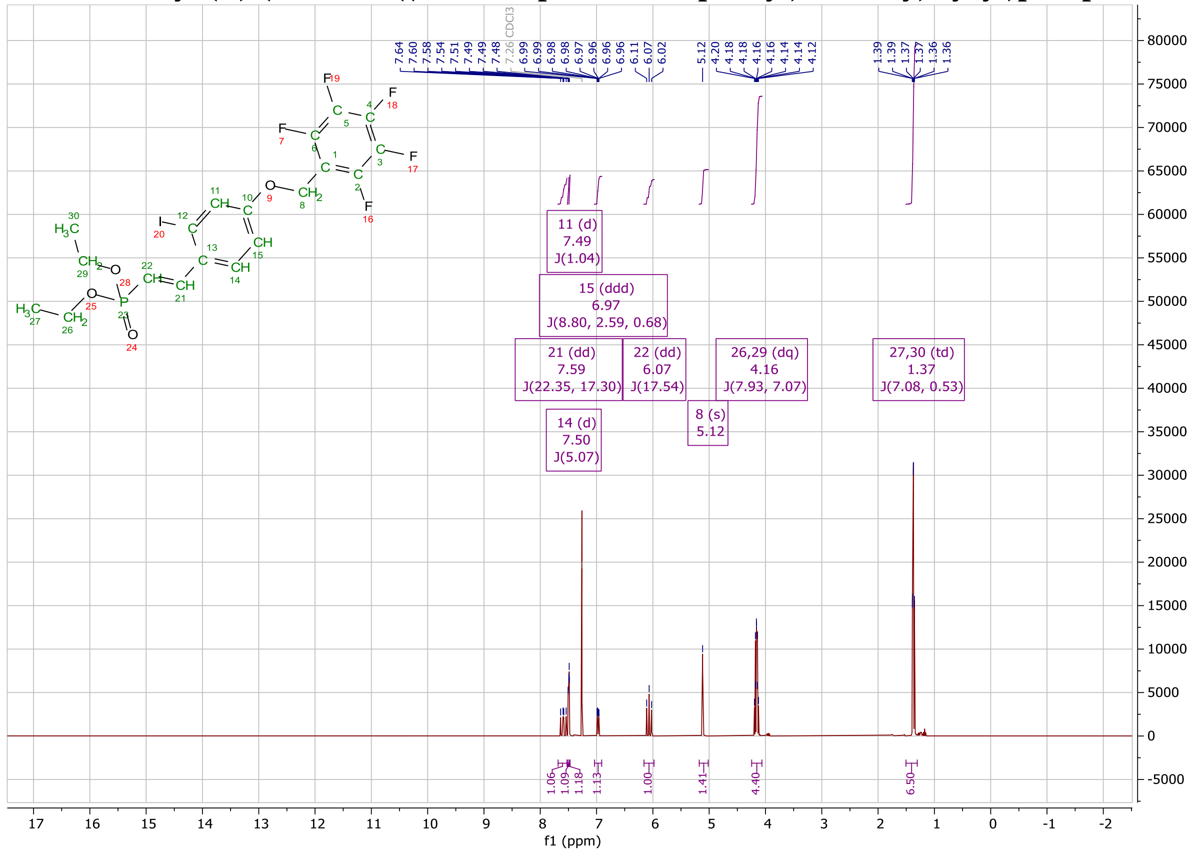

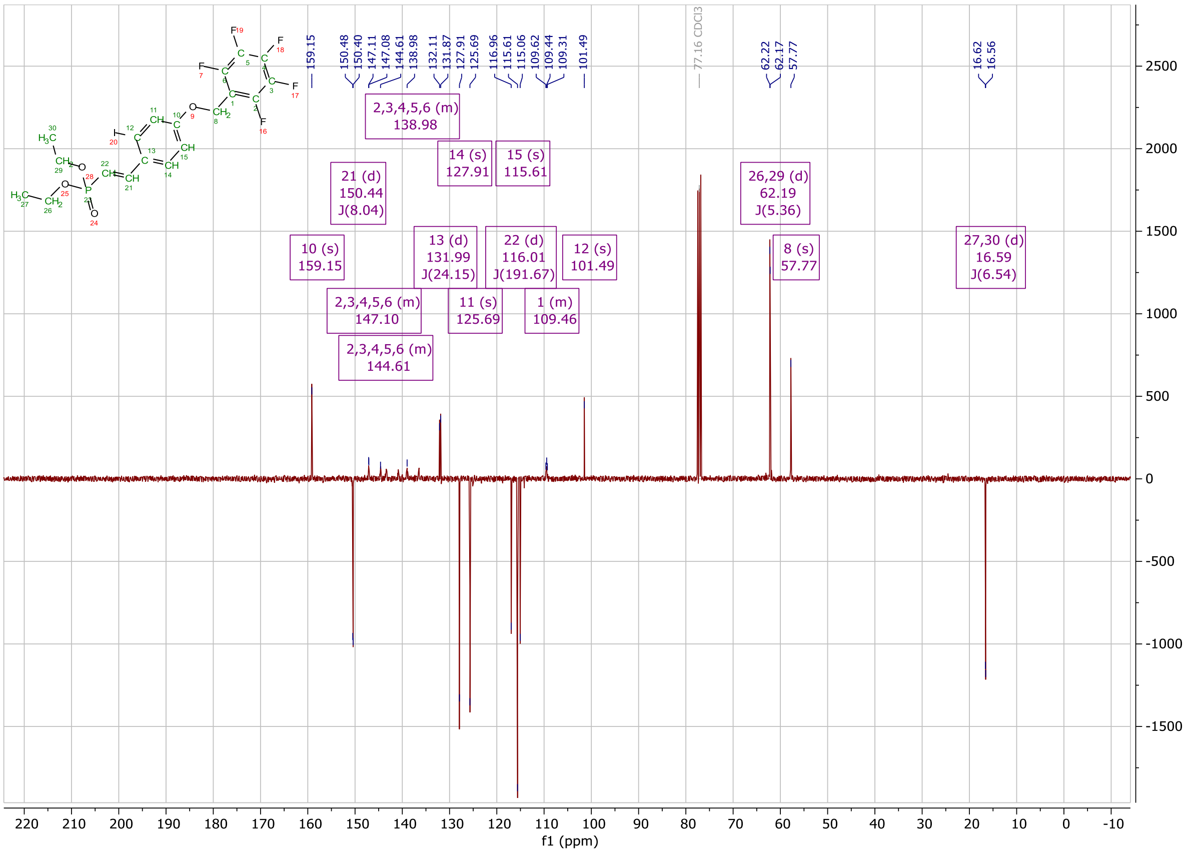
159.15
150.48
150.40
147.11
147.08
144.61
138.98
132.11
131.87
127.91
125.69
116.96
115.61
115.06
109.62
109.44
109.31
101.49
77.16 CDCl3
62.22
62.17
57.77
16.62
16.56
2,3,4,5,6 (m)
138.98
14 (s)
127.91
15 (s)
115.61
21 (d)
150.44
J(8.04)
26,29 (d)
62.19
J(5.36)
10 (s)
159.15
13 (d)
131.99
J(24.15)
22 (d)
116.01
J(191.67)
12 (s)
101.49
8 (s)
57.77
27,30 (d)
16.59
J(6.54)
2,3,4,5,6 (m)
147.10
11 (s)
125.69
1 (m)
109.46
2,3,4,5,6 (m)
144.61
f1 (ppm)

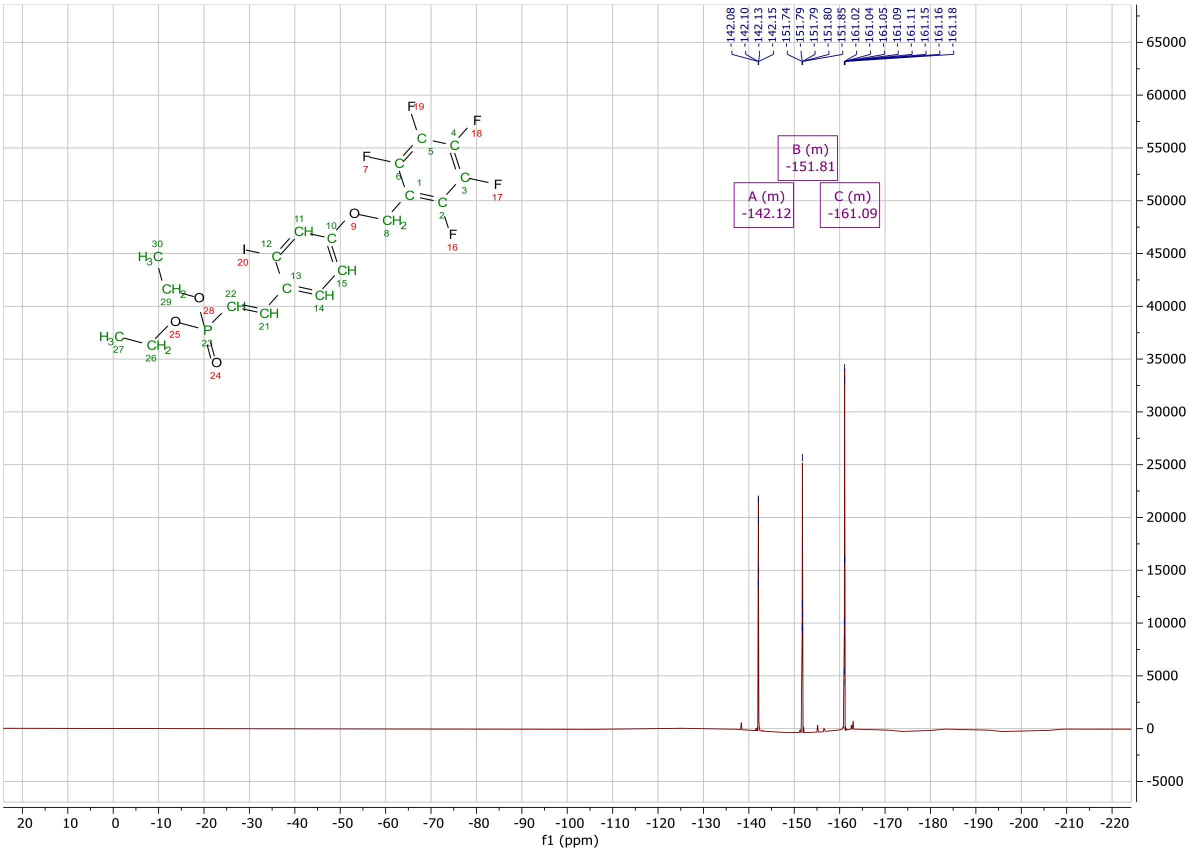
-142.08
-142.10
-142.13
-142.15
-151.74
-151.79
-151.80
-151.85
-161.02
-161.04
-161.05
-161.09
-161.11
-161.15
-161.16
-161.18
B (m)
-151.81
A (m)
-142.12
C (m)
-161.09
f1 (ppm)

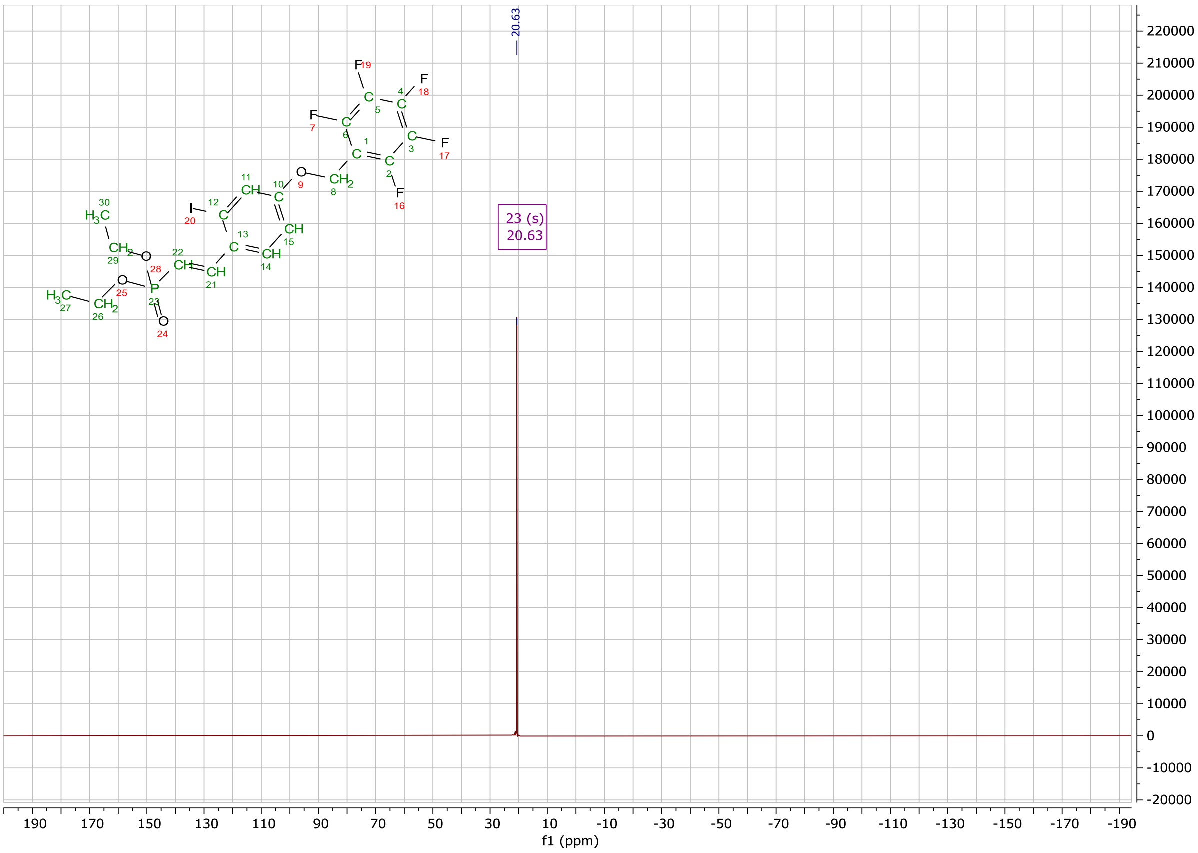
20.63
23 (s)
20.63
f1 (ppm)

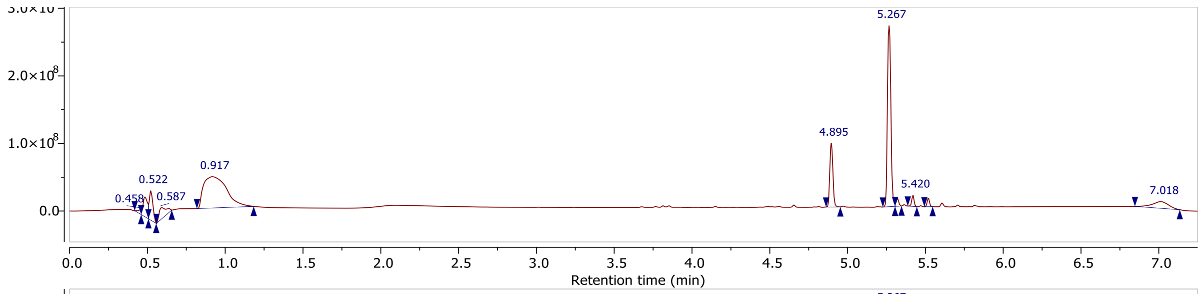
5.267
4.895
0.917
0.522
0.458
0.587
5.420
7.018
Retention time (min)

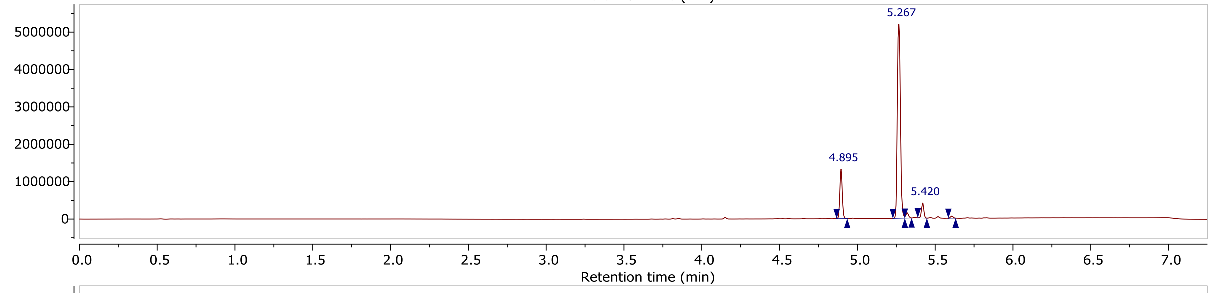
5.267
4.895
5.420
Retention time (min)

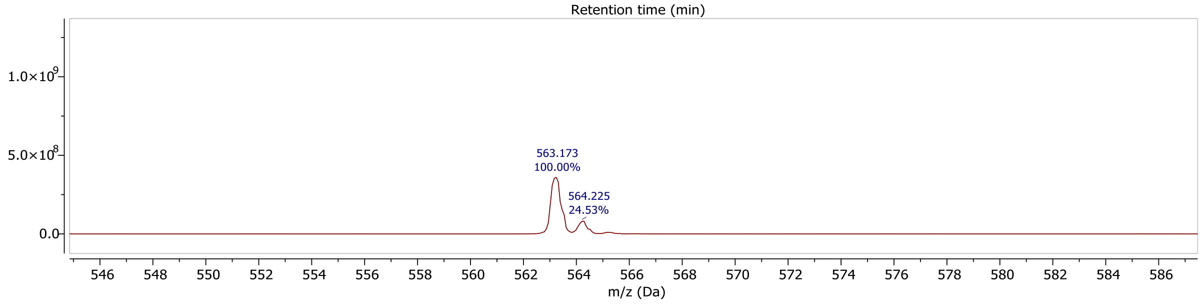
563.173
100.00%
564.225
24.53%
m/z (Da)

3.2.52 Diethyl (*E*)-(2-iodo-5-methoxystyryl)phosphonate (43e)

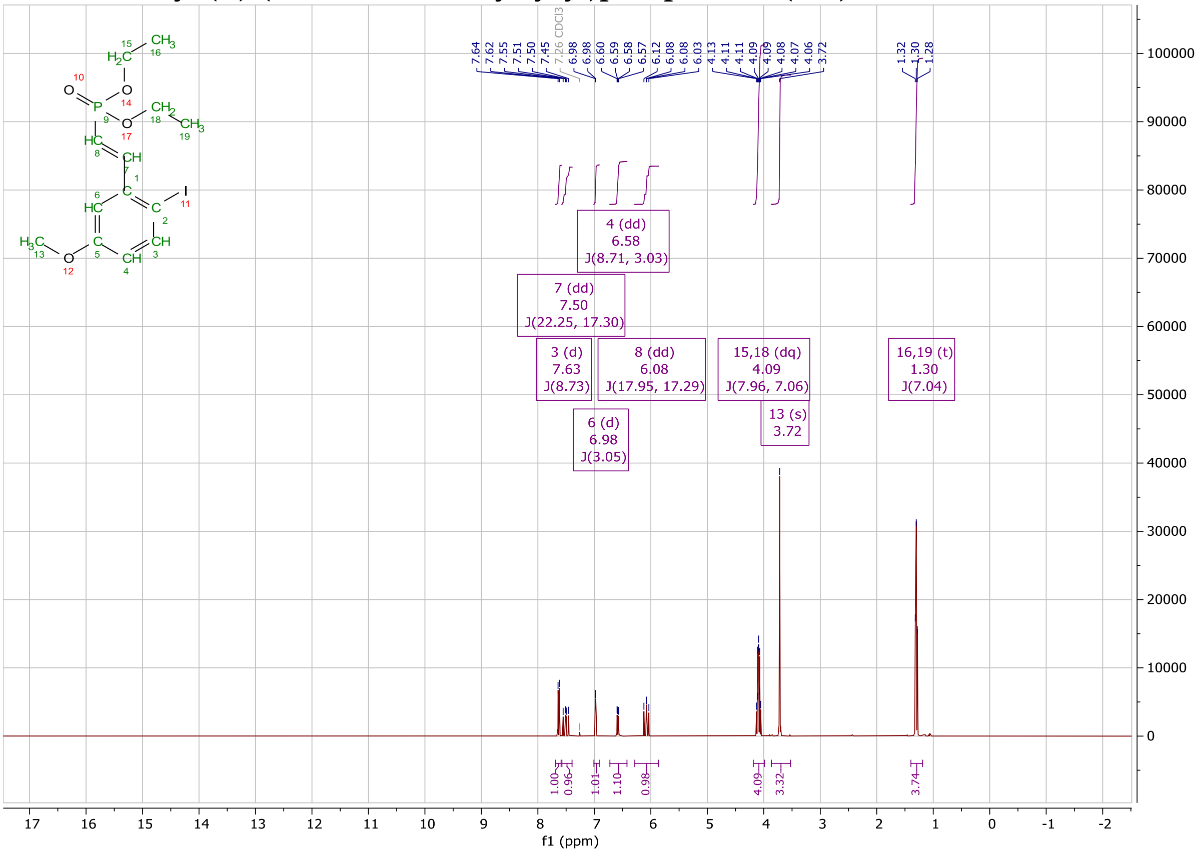

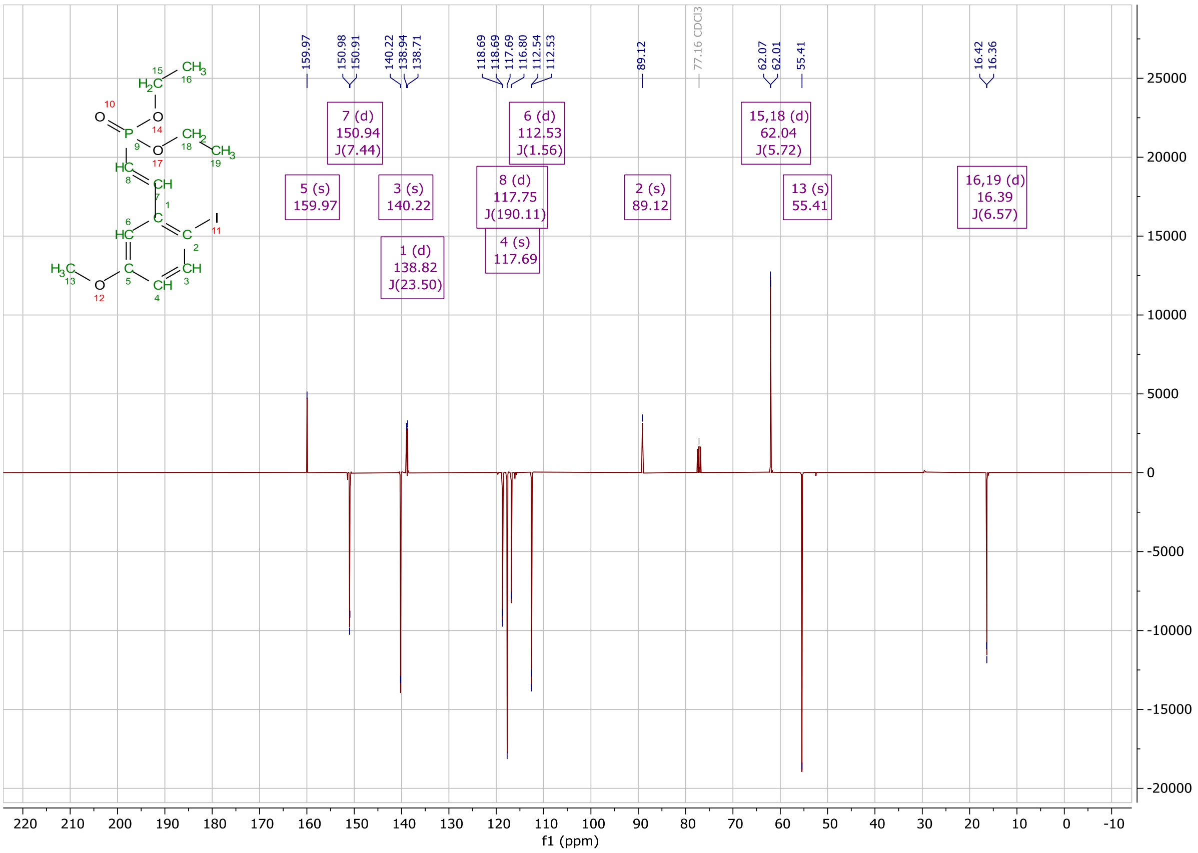

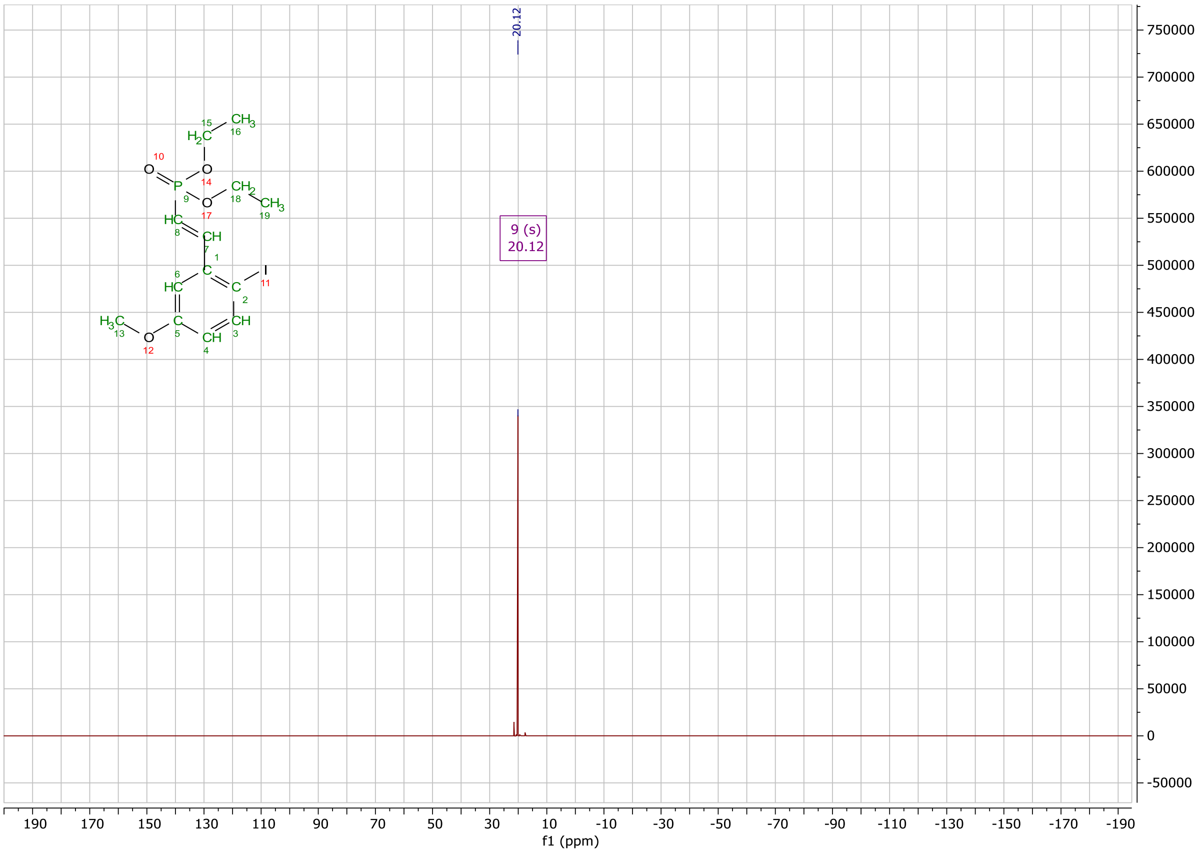

20.12
9 (s)
20.12
f1 (ppm)

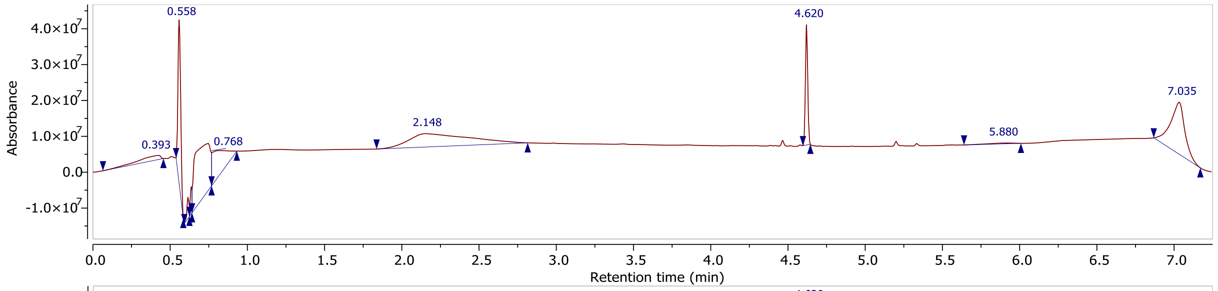

0.558
4.620
0.393
0.768
2.148
5.880
7.035
Absorbance
Retention time (min)

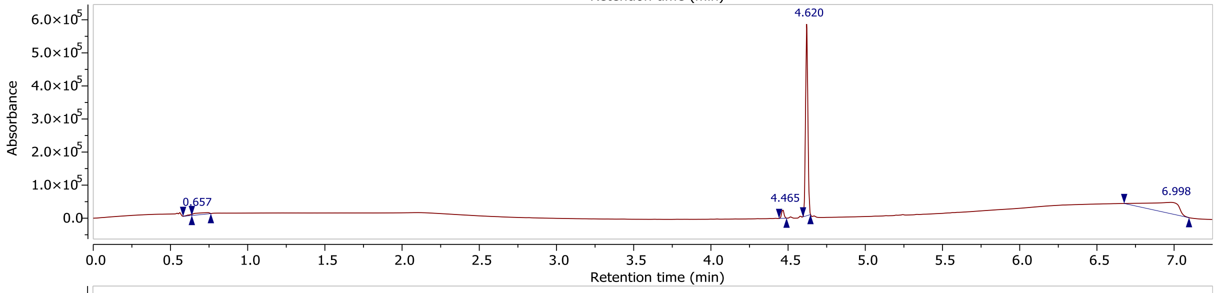

4.620
0.657
4.465
6.998
Absorbance
Retention time (min)

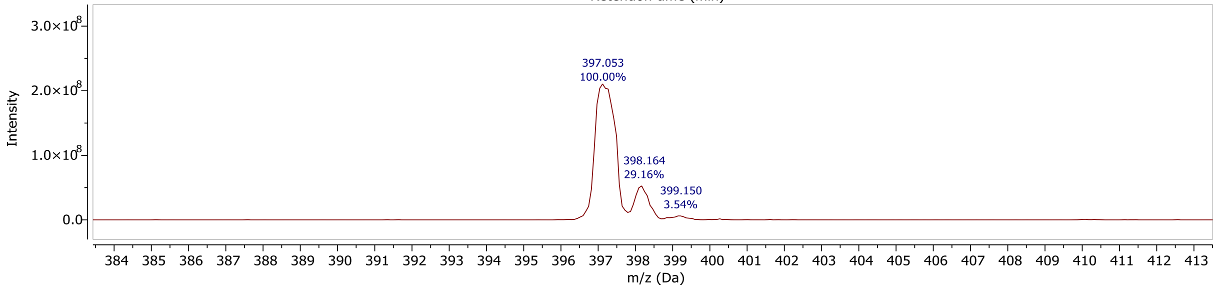

397.053
100.00%
398.164
29.16%
399.150
3.54%
Intensity
m/z (Da)

3.2.53 Diethyl (*E*)-(2-iodo-5-isopropoxystyryl)phosphonate (43f)

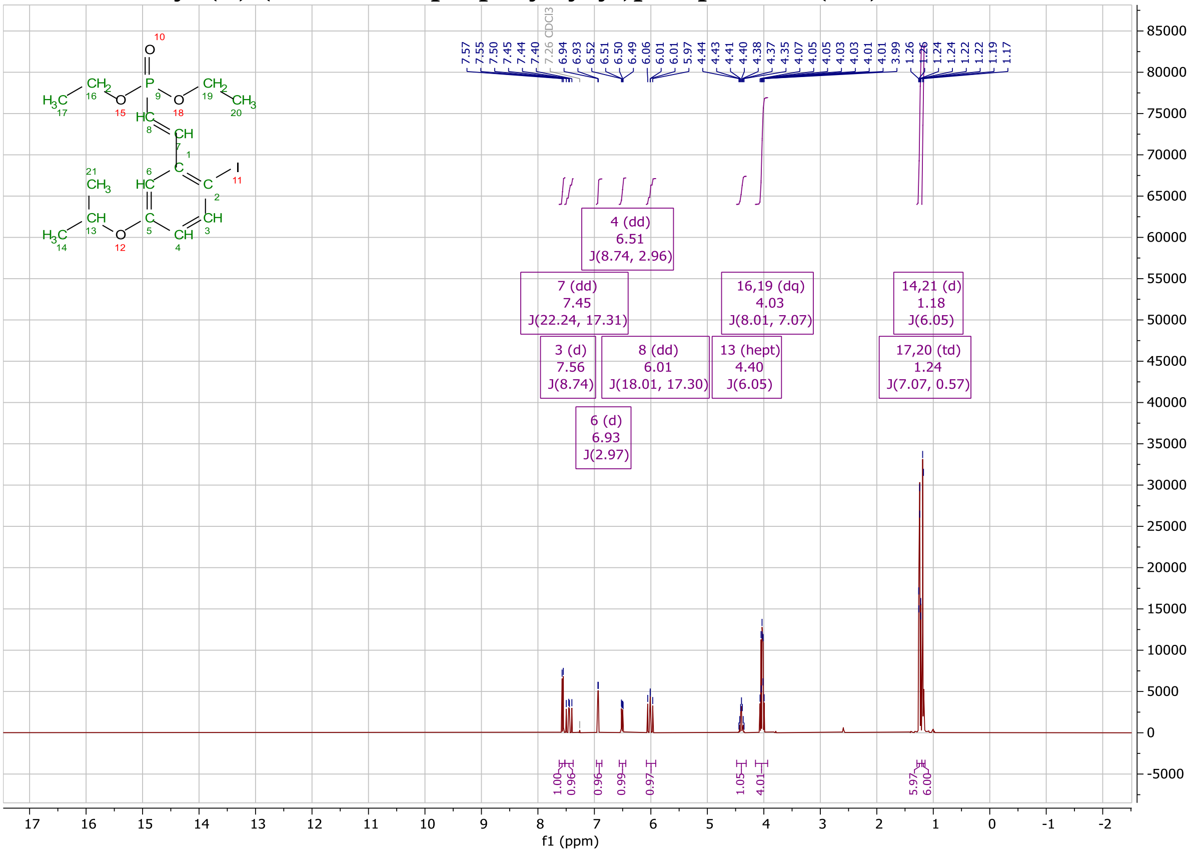

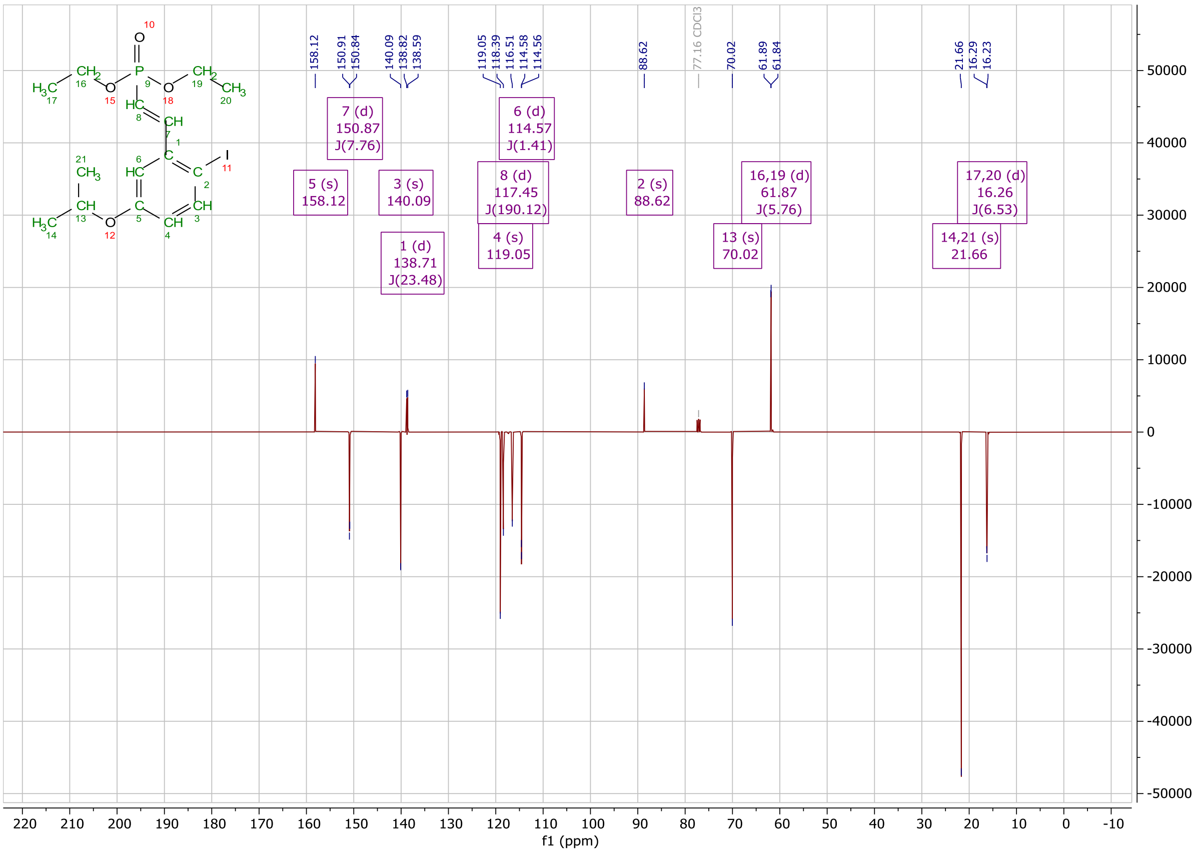

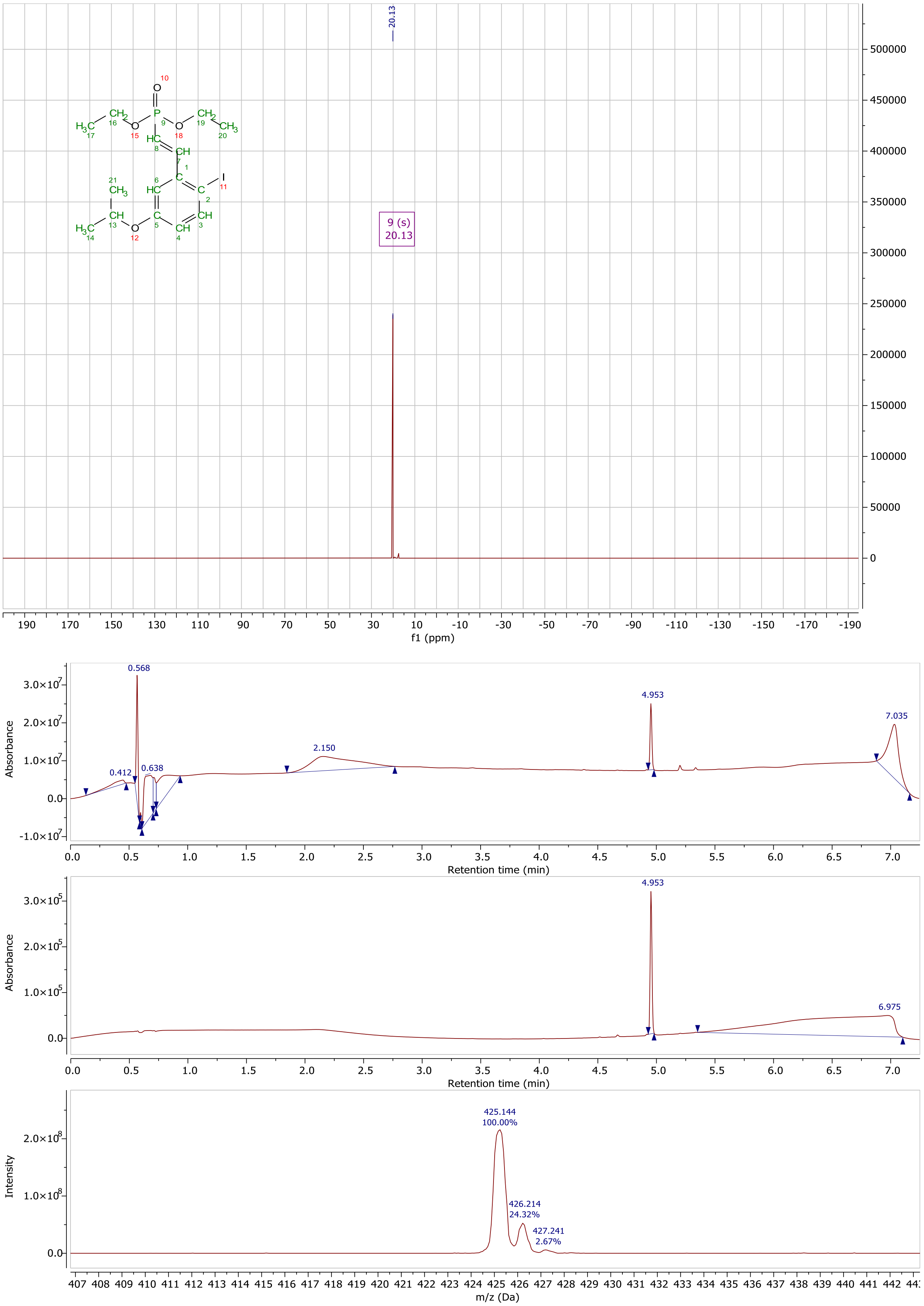

20.13
9 (s)
20.13
f1 (ppm)
0.568
0.412
0.638
2.150
4.953
7.035
Absorbance
Retention time (min)
4.953
6.975
Absorbance
Retention time (min)
425.144
100.00%
426.214
24.32%
427.241
2.67%
Intensity
m/z (Da)

3.2.54 Diethyl (*E*)-(2-iodo-5-((2,3,4,5,6-pentafluorophenyl)oxy)styryl)phosphonate (43g)

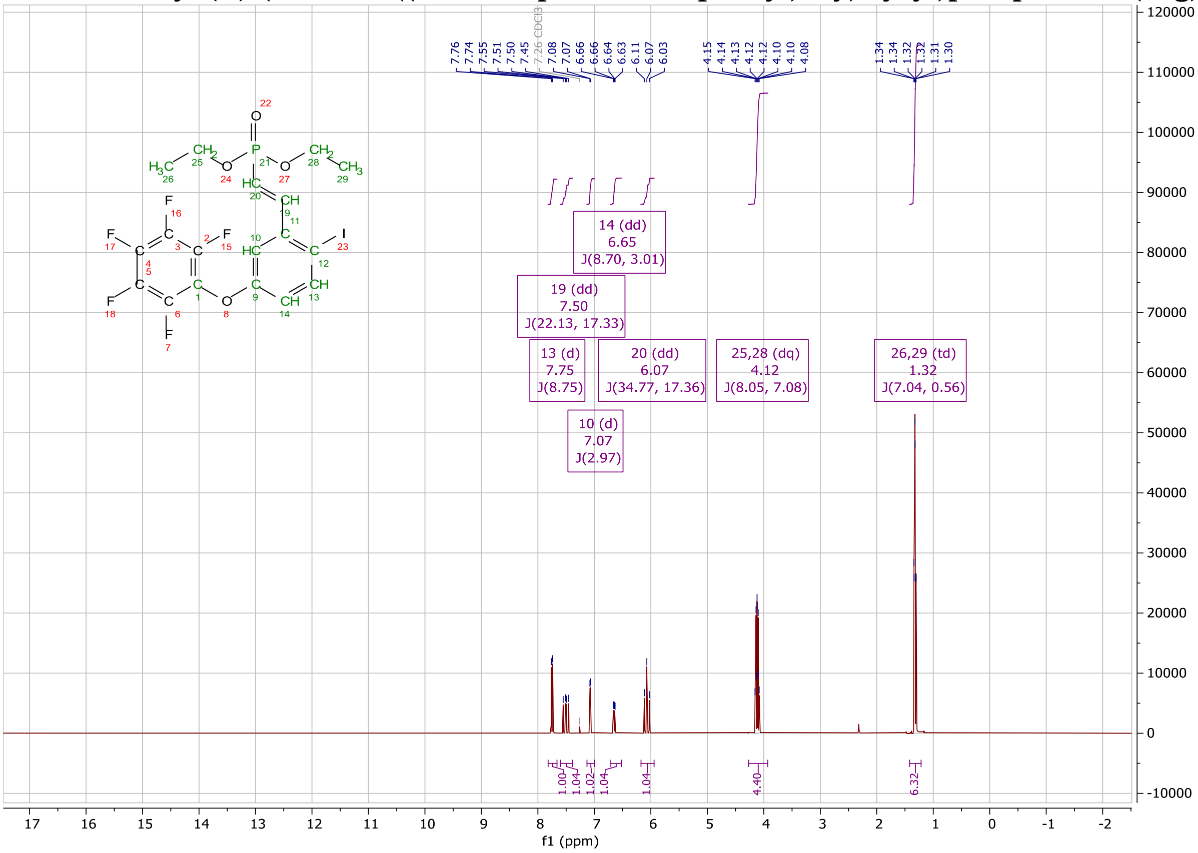

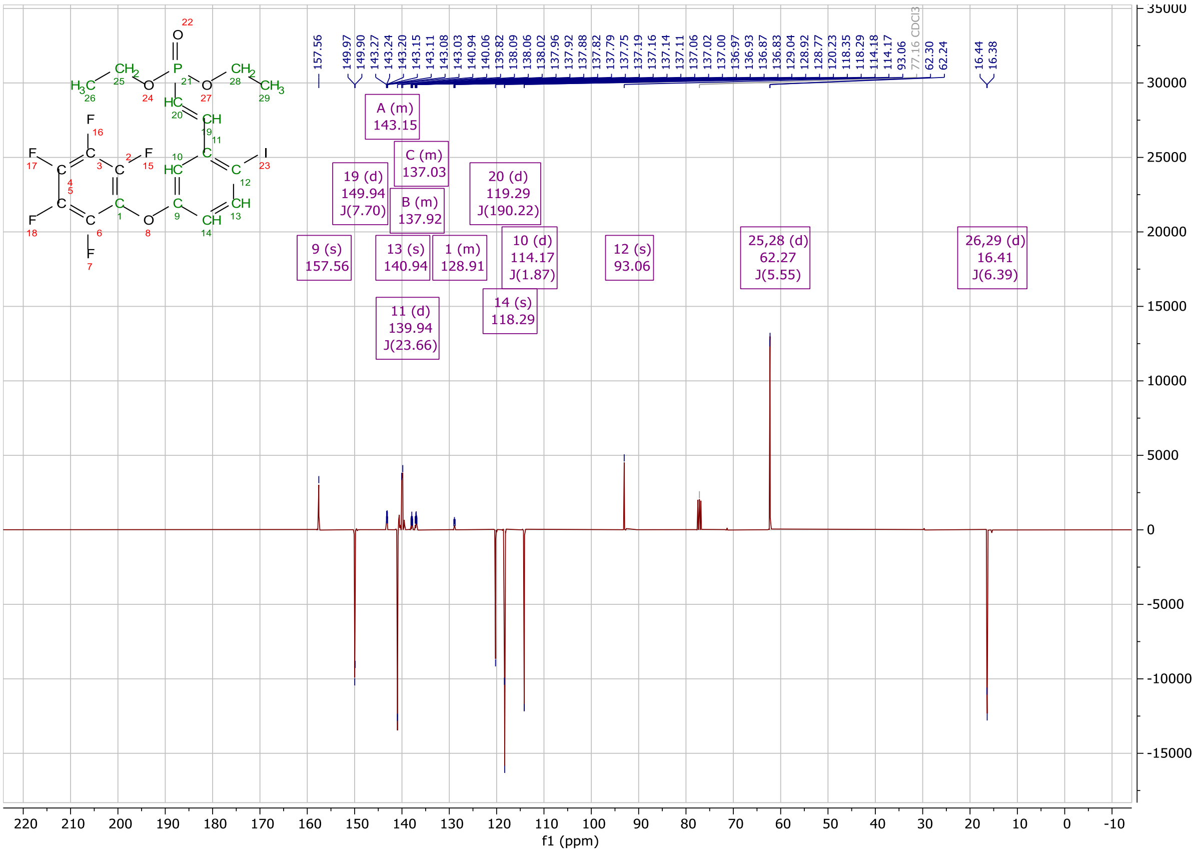

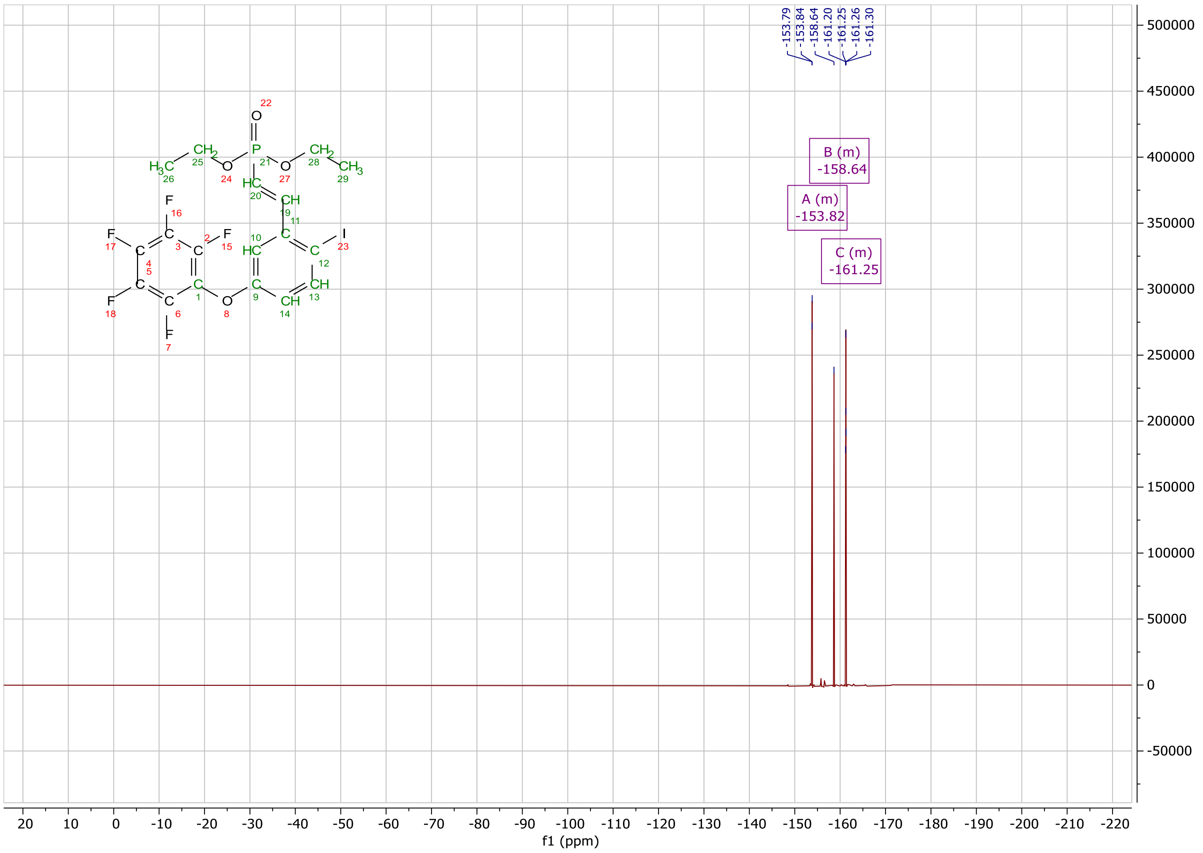
-153.79
-153.84
-158.64
-161.20
-161.25
-161.26
-161.30
B (m)
-158.64
A (m)
-153.82
C (m)
-161.25
f1 (ppm)

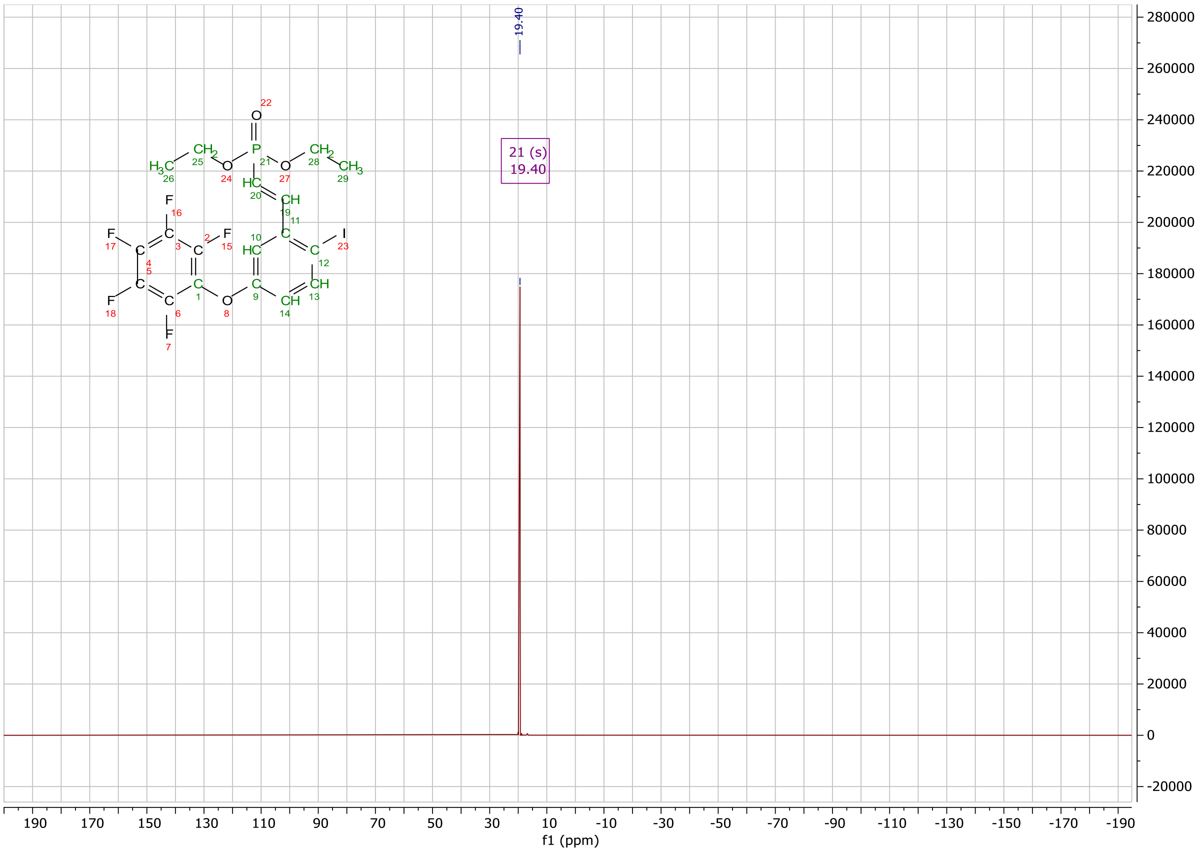
19.40
21 (s)
19.40
f1 (ppm)

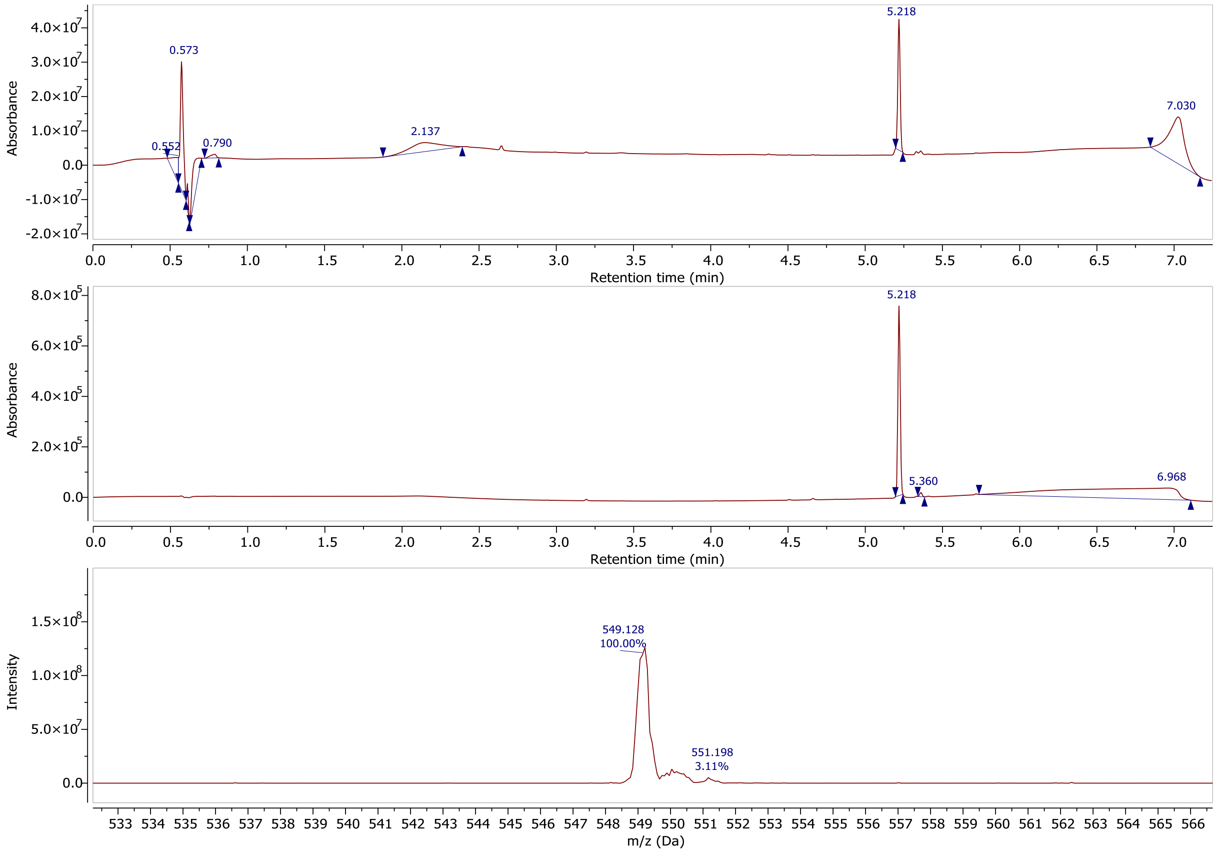

3.2.55 Diethyl (*E*)-(2-iodo-5-((2,3,4,5,6-pentafluorophenyl)methoxy)styryl)phosphonate (43h)

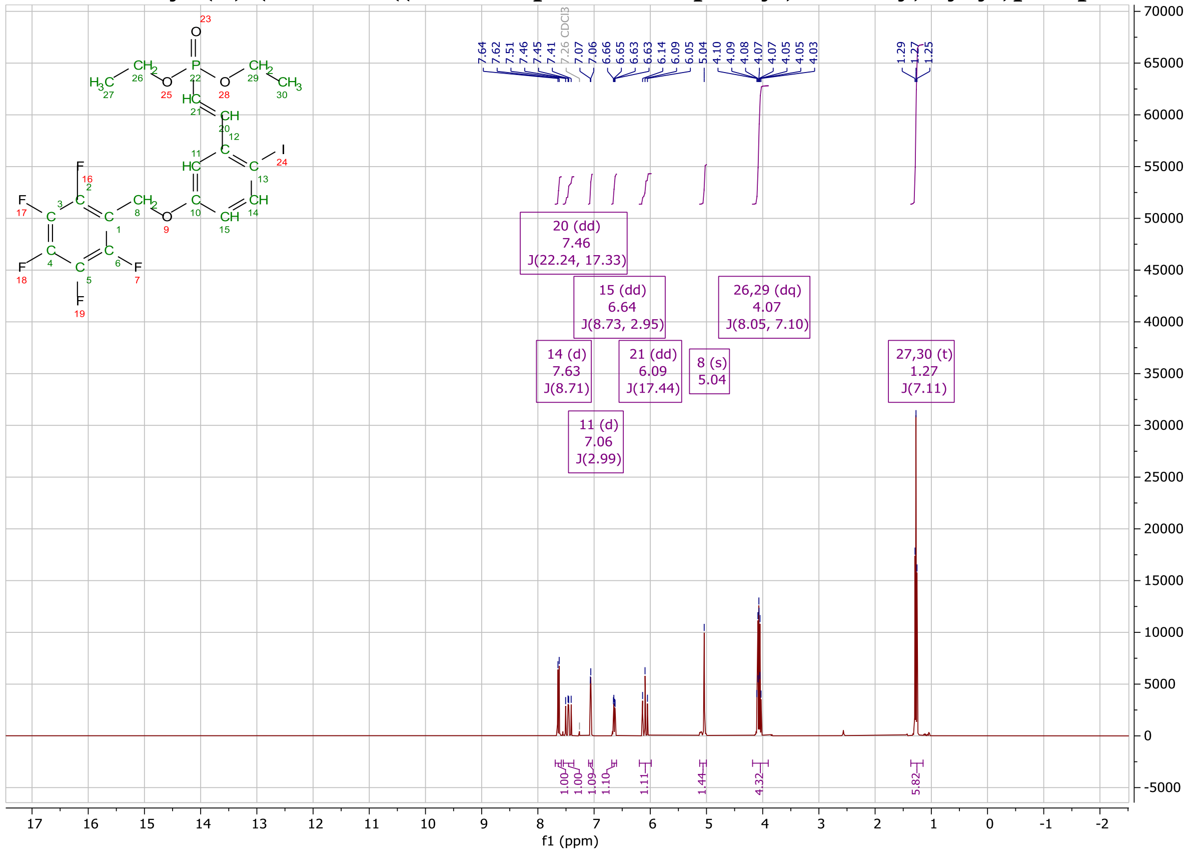

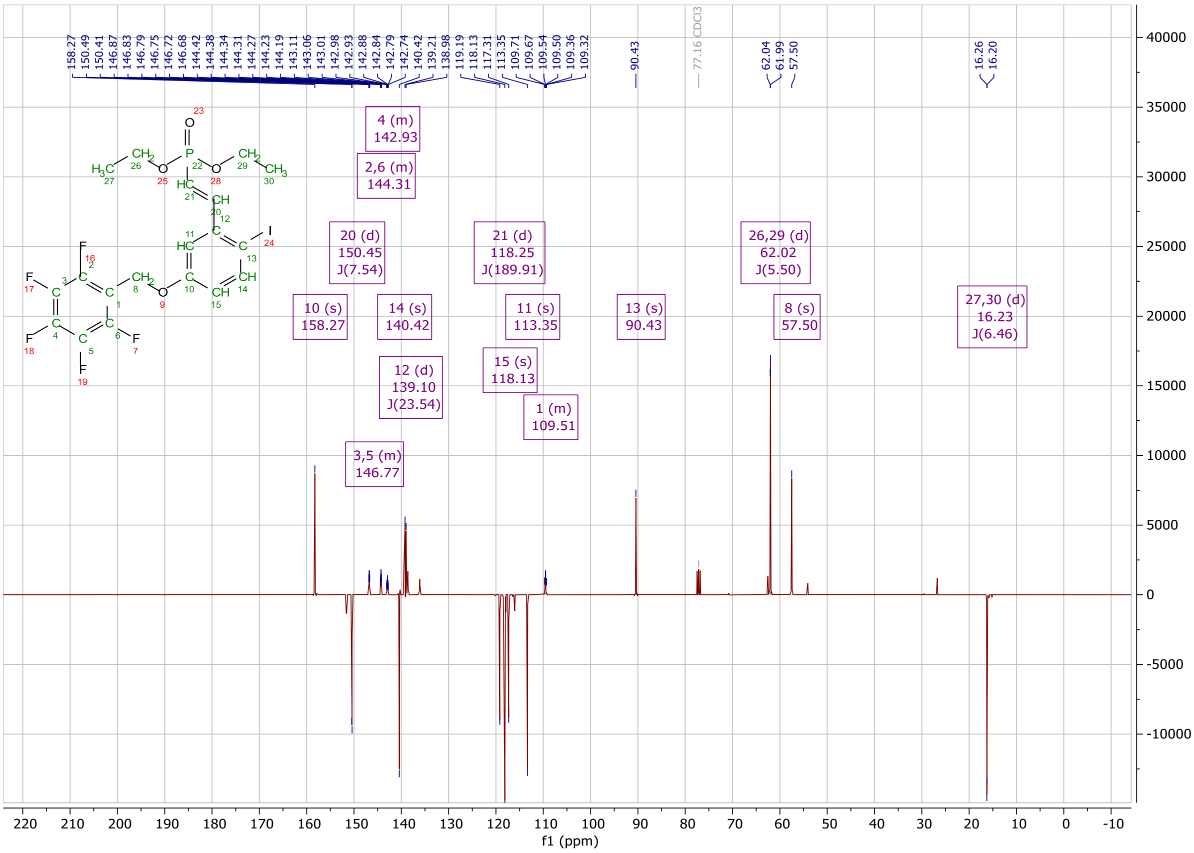

4 (m) 142.93
2,6 (m) 144.31
20 (d) 150.45 J(7.54)
21 (d) 118.25 J(189.91)
26,29 (d) 62.02 J(5.50)
10 (s) 158.27
14 (s) 140.42
11 (s) 113.35
13 (s) 90.43
8 (s) 57.50
27,30 (d) 16.23 J(6.46)
12 (d) 139.10 J(23.54)
15 (s) 118.13
1 (m) 109.51
3,5 (m) 146.77
77.16 CDCl3
f1 (ppm)

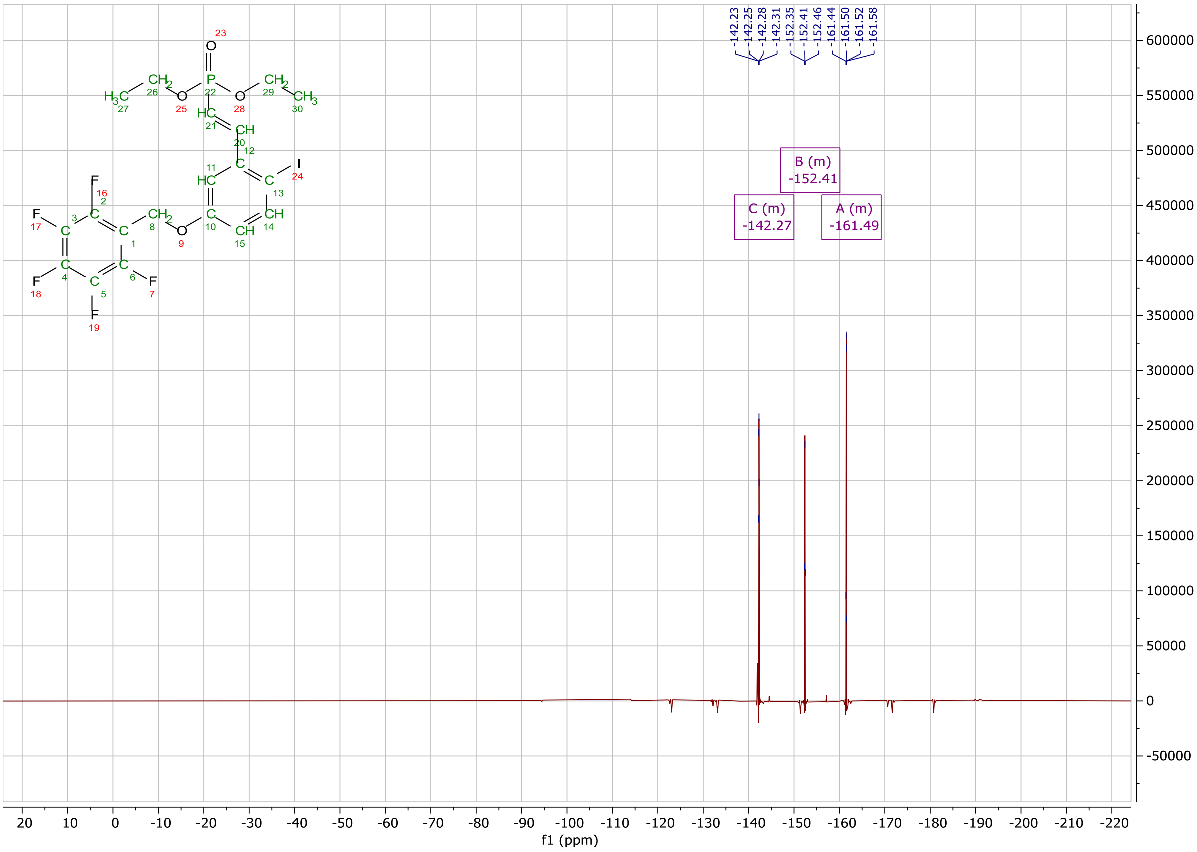

B (m) -152.41
C (m) -142.27
A (m) -161.49
f1 (ppm)

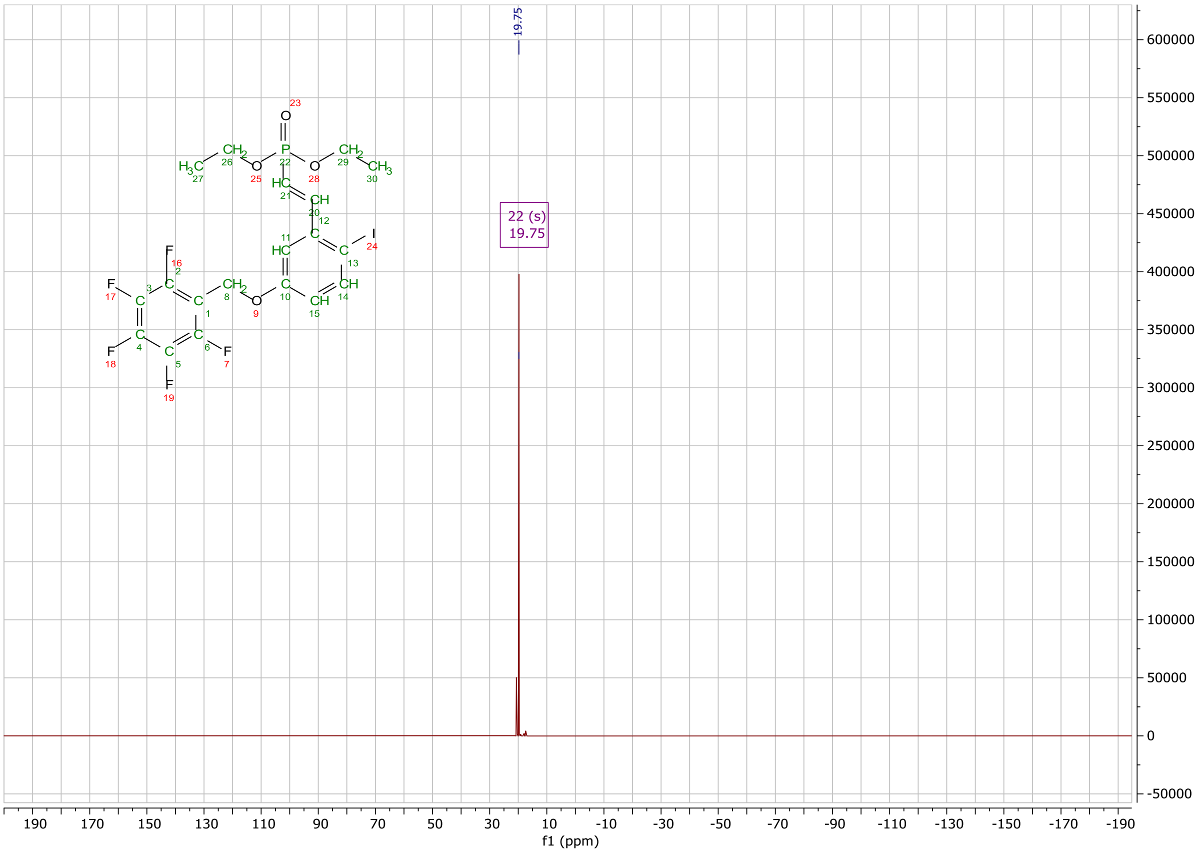
19.75
22 (s)
19.75
f1 (ppm)

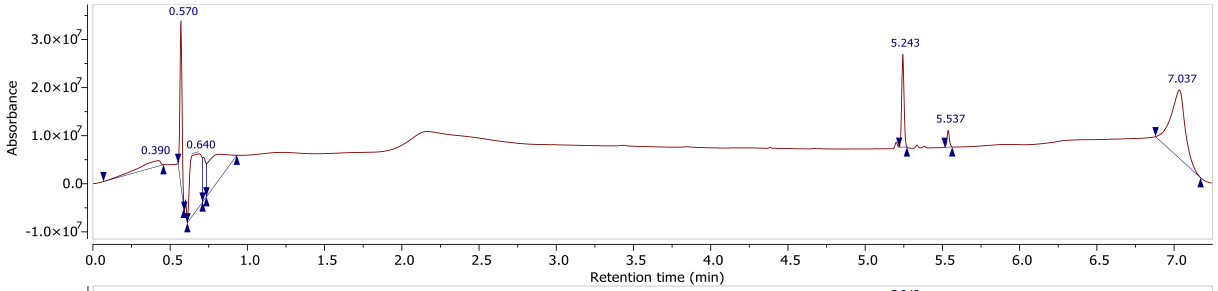
0.570
0.390
0.640
5.243
5.537
7.037
Absorbance
Retention time (min)

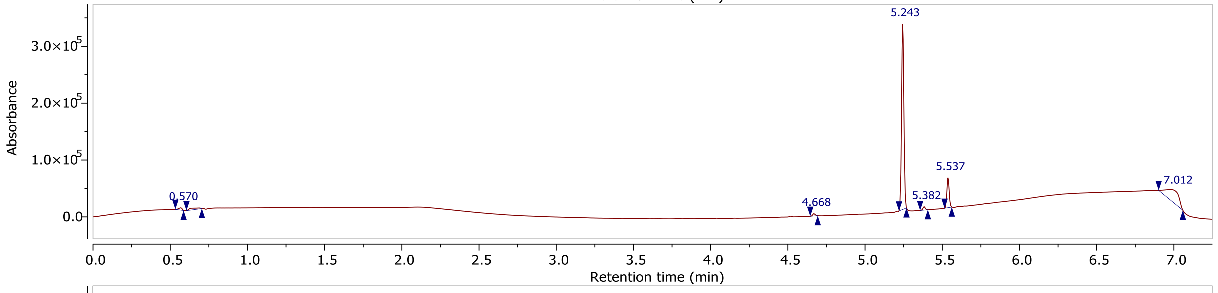
5.243
0.570
4.668
5.382
5.537
7.012
Absorbance
Retention time (min)

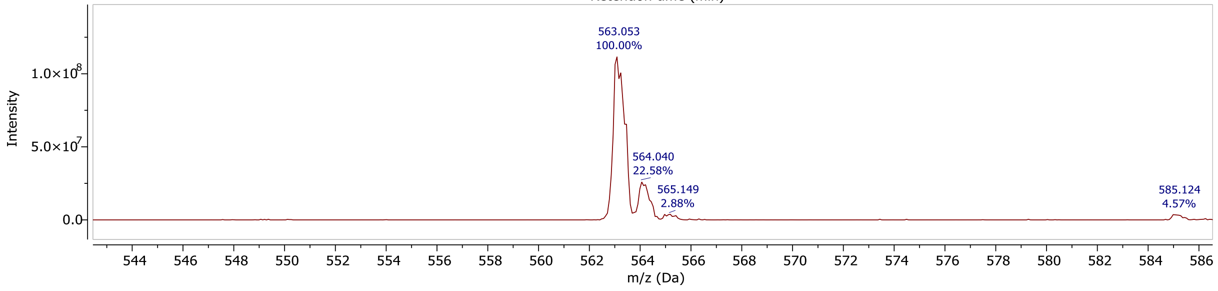
563.053
100.00%
564.040
22.58%
565.149
2.88%
585.124
4.57%
Intensity
m/z (Da)

3.2.56 Diethyl (*E*)-(2-iodo-6-methoxystyryl)phosphonate (43i)

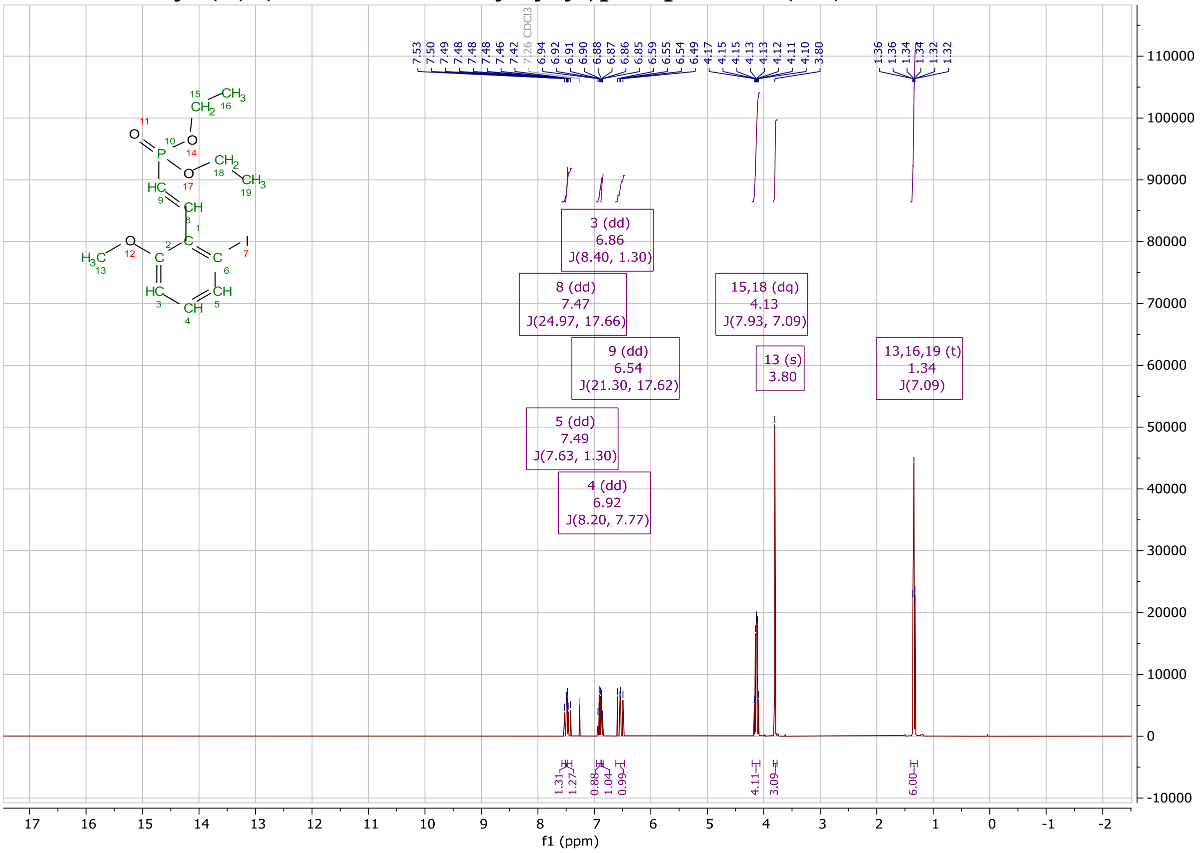

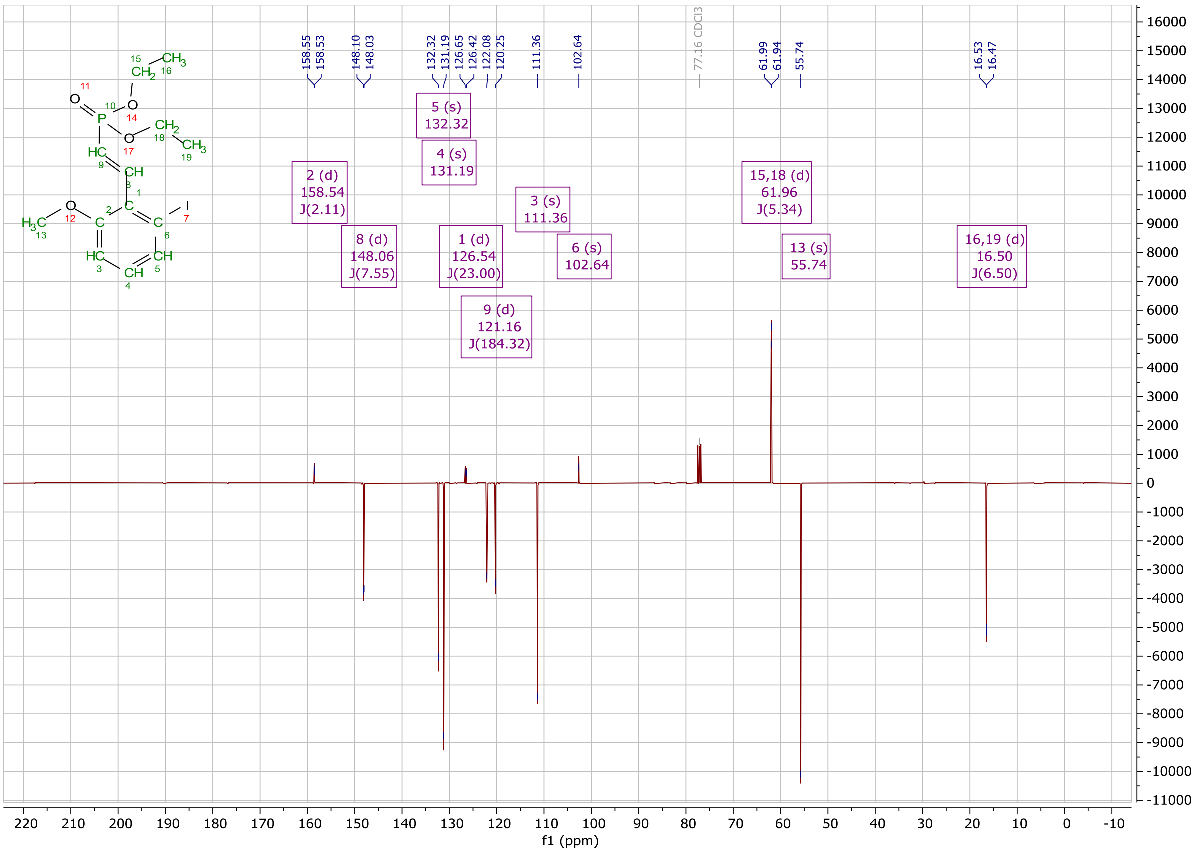

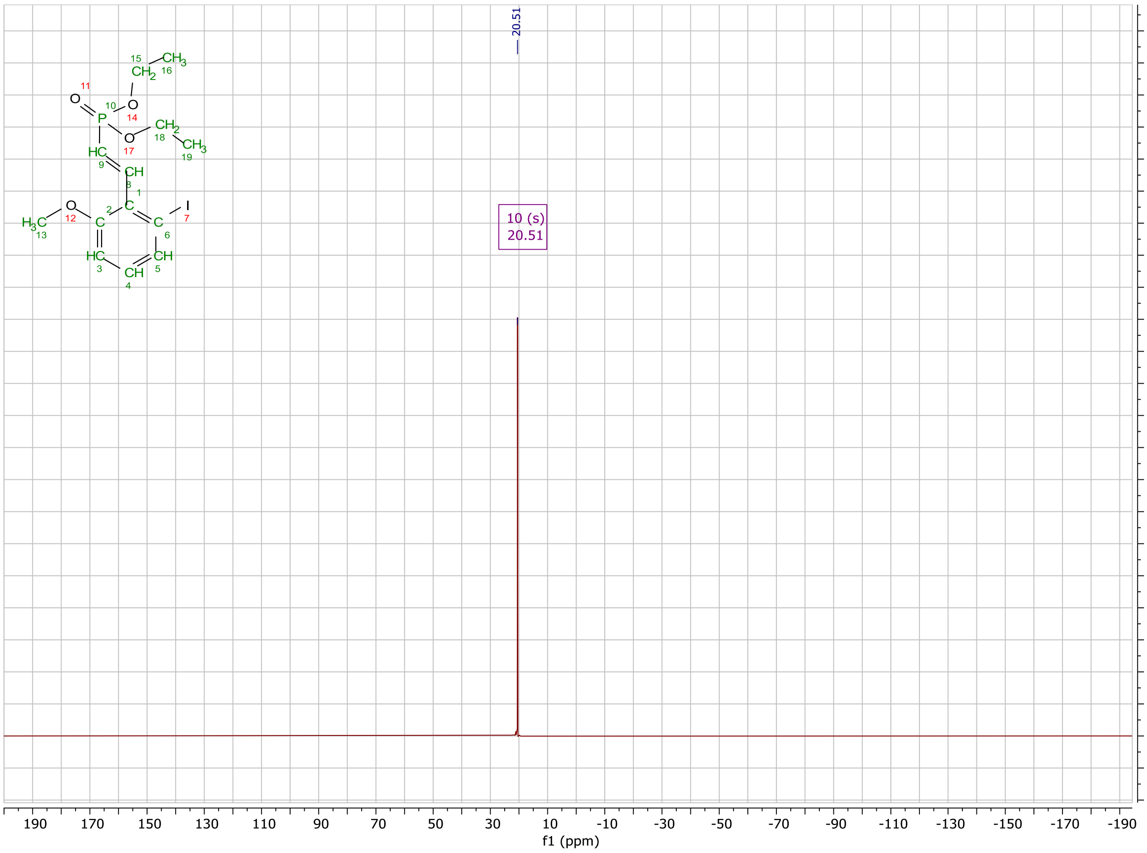
20.51
10 (s)
20.51
f1 (ppm)

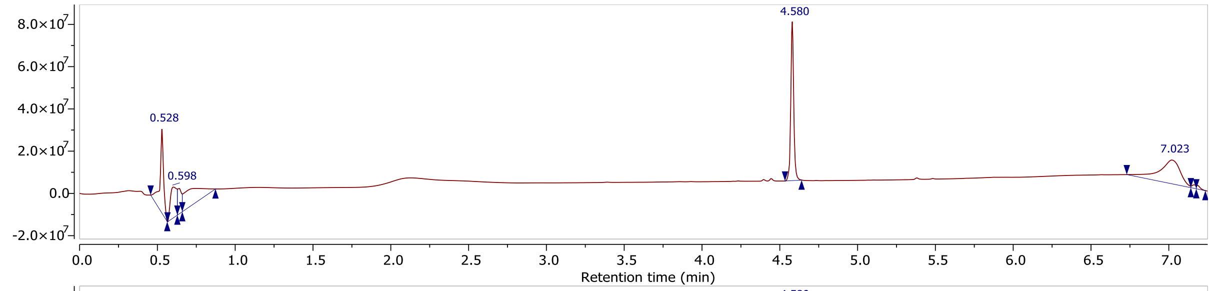
4.580
0.528
0.598
7.023
Retention time (min)

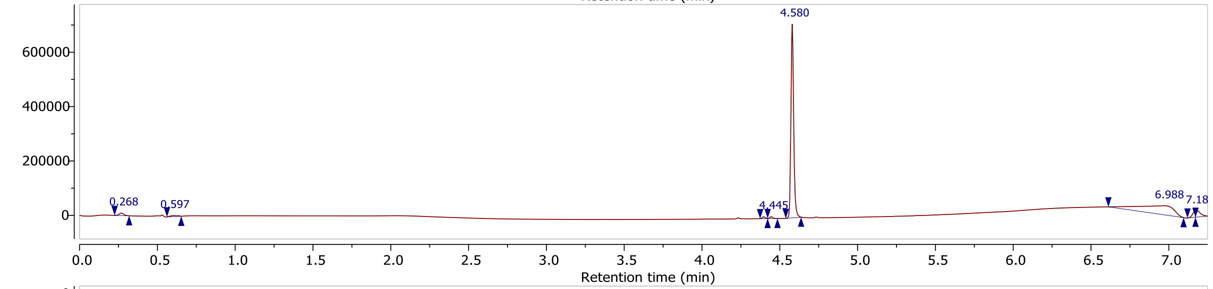
4.580
0.268
0.597
4.445
6.988
Retention time (min)

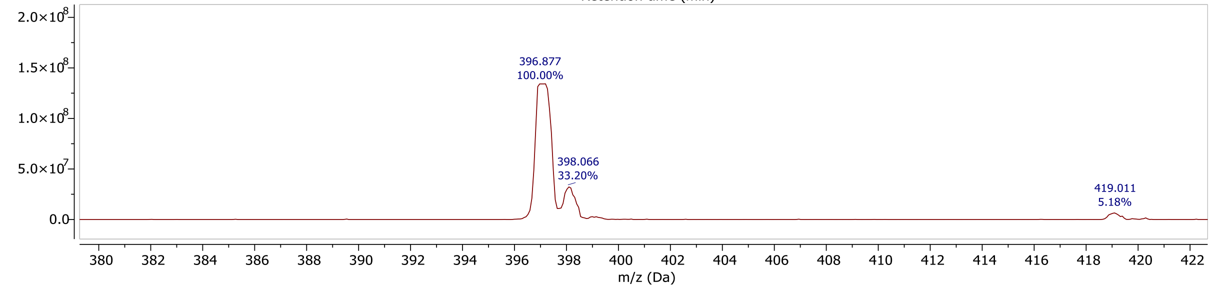
396.877
100.00%
398.066
33.20%
419.011
5.18%
m/z (Da)

3.2.57 Diethyl (*E*)-(2-iodo-6-isopropoxystyryl)phosphonate (43j)

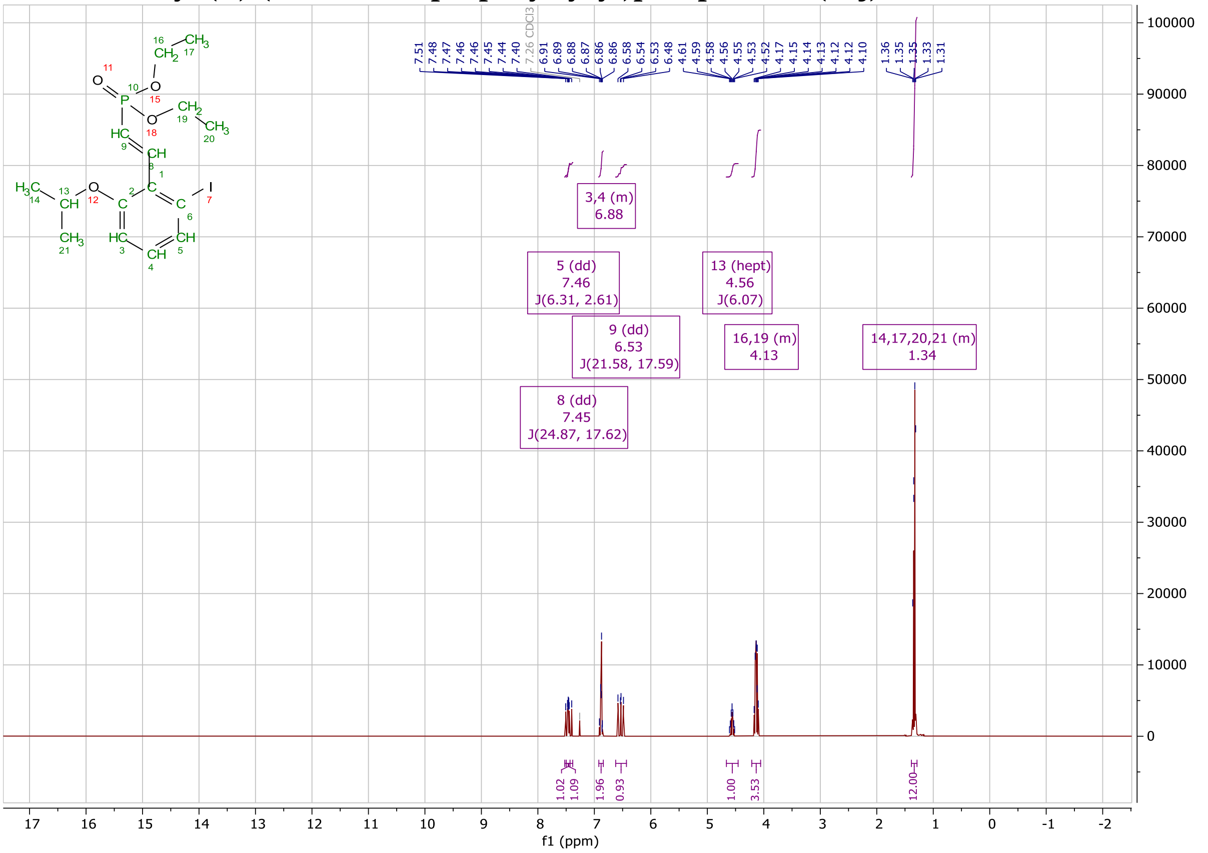

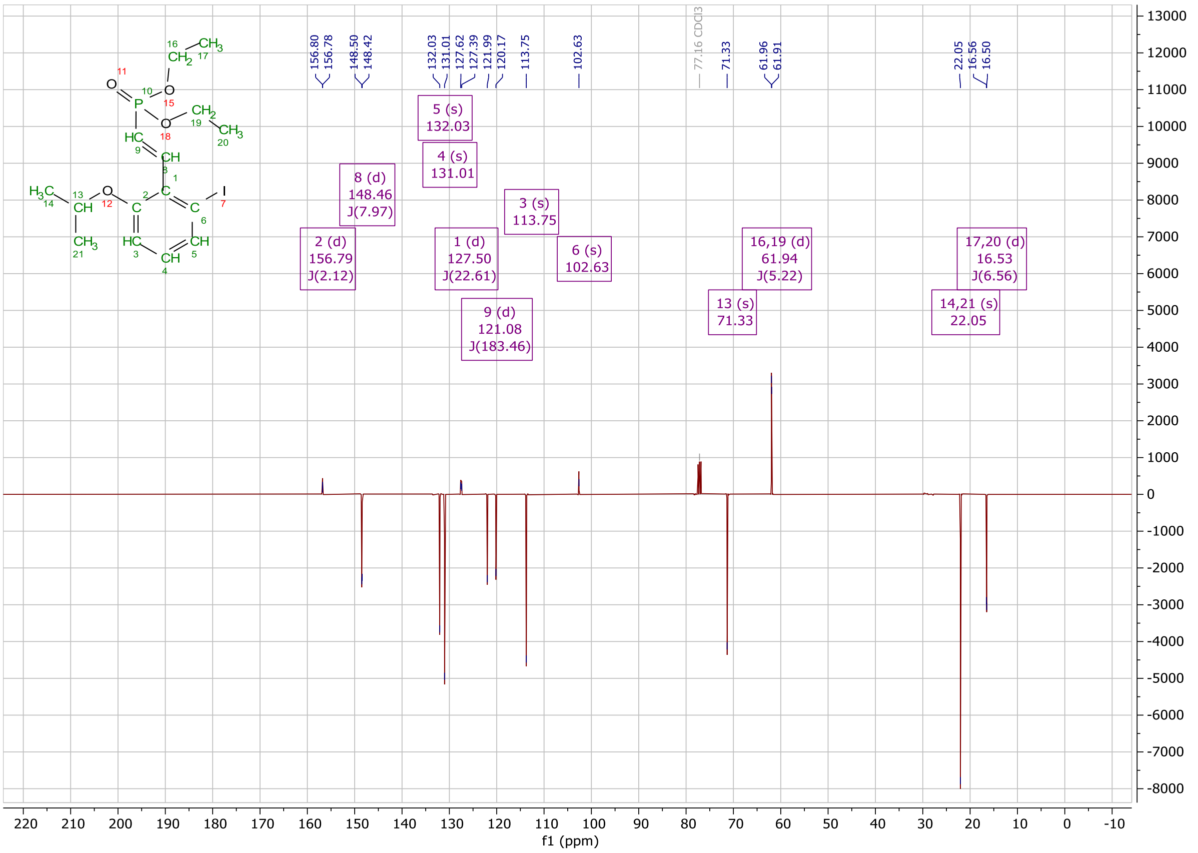

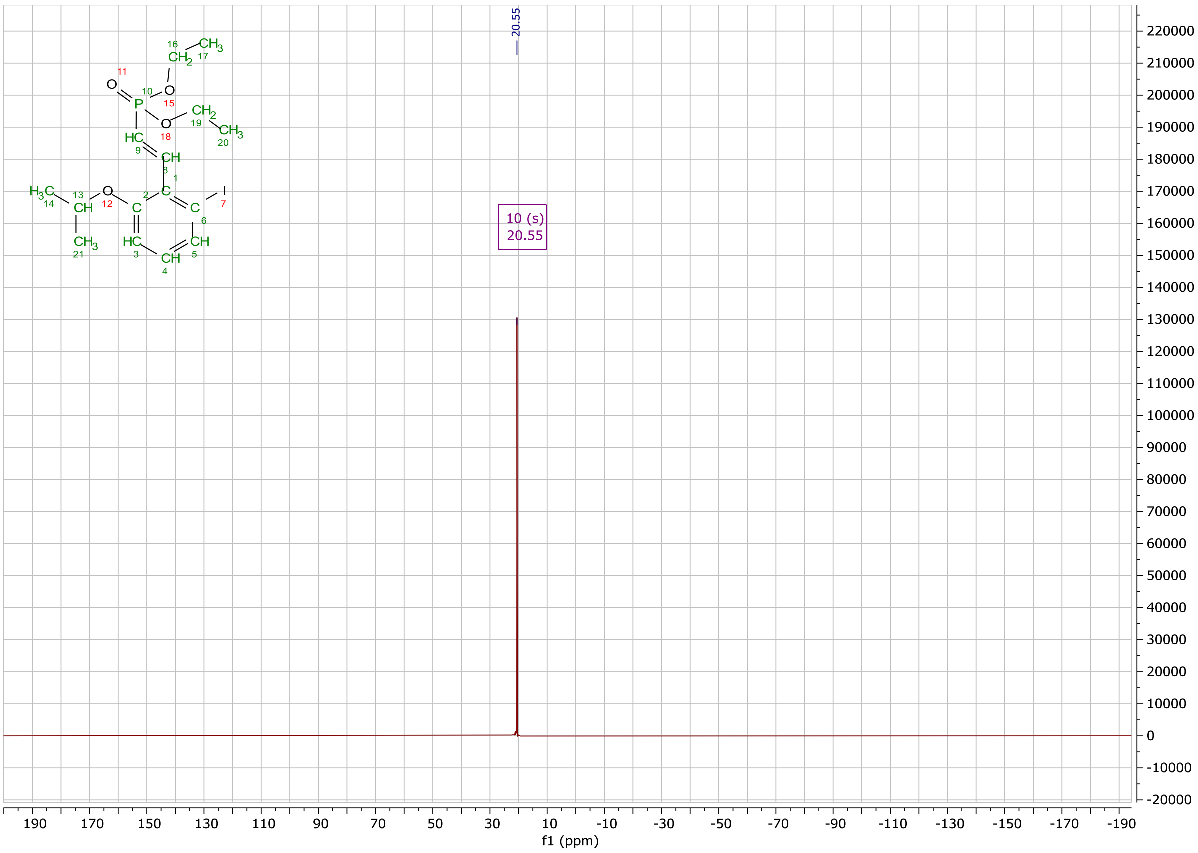
10 (s)
20.55
f1 (ppm)

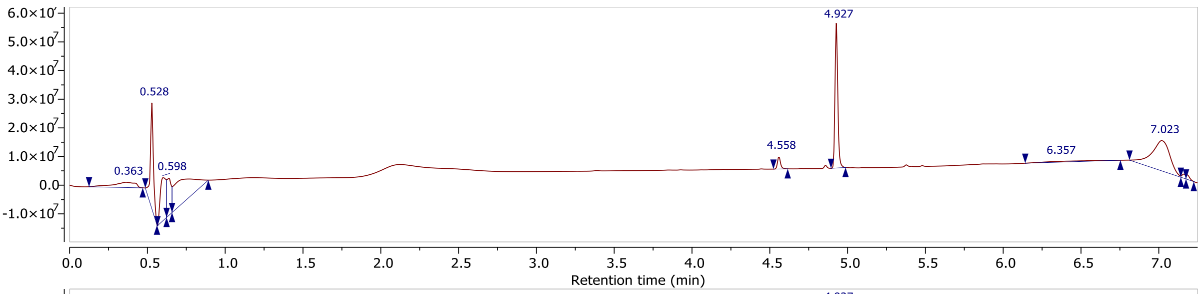
0.528
0.363
0.598
4.558
4.927
6.357
7.023
Retention time (min)

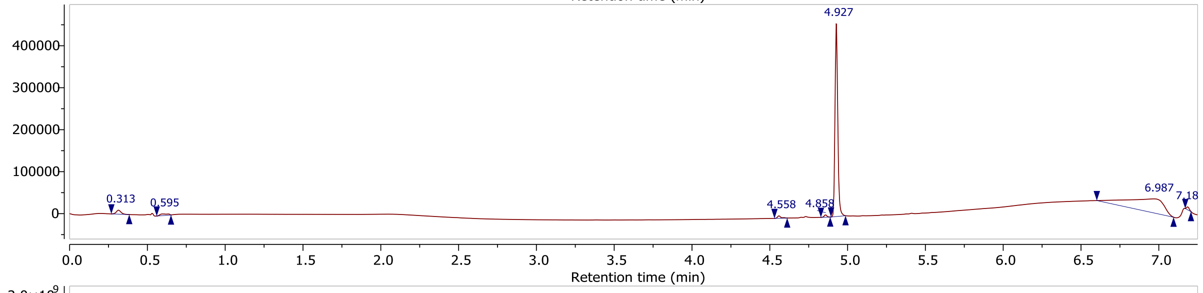
0.313
0.595
4.558
4.858
4.927
6.987
Retention time (min)

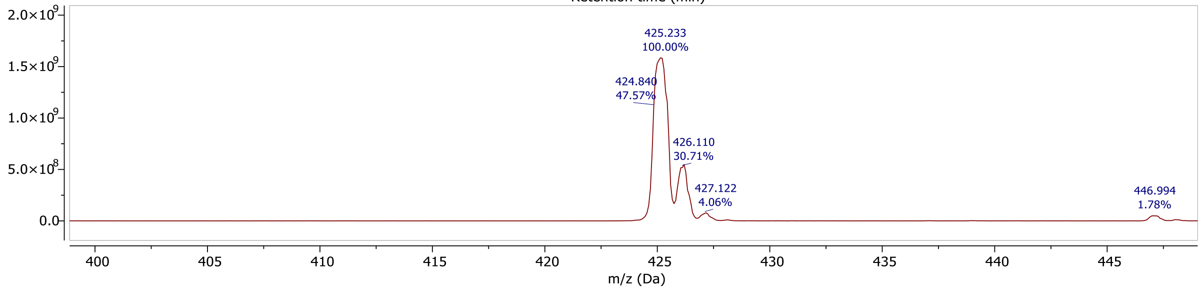
425.233
100.00%
424.840
47.57%
426.110
30.71%
427.122
4.06%
446.994
1.78%
m/z (Da)

3.2.58 Diethyl (*E*)-(2-iodo-6-((2,3,4,5,6-pentafluorophenyl)oxy)styryl)phosphonate (43k)

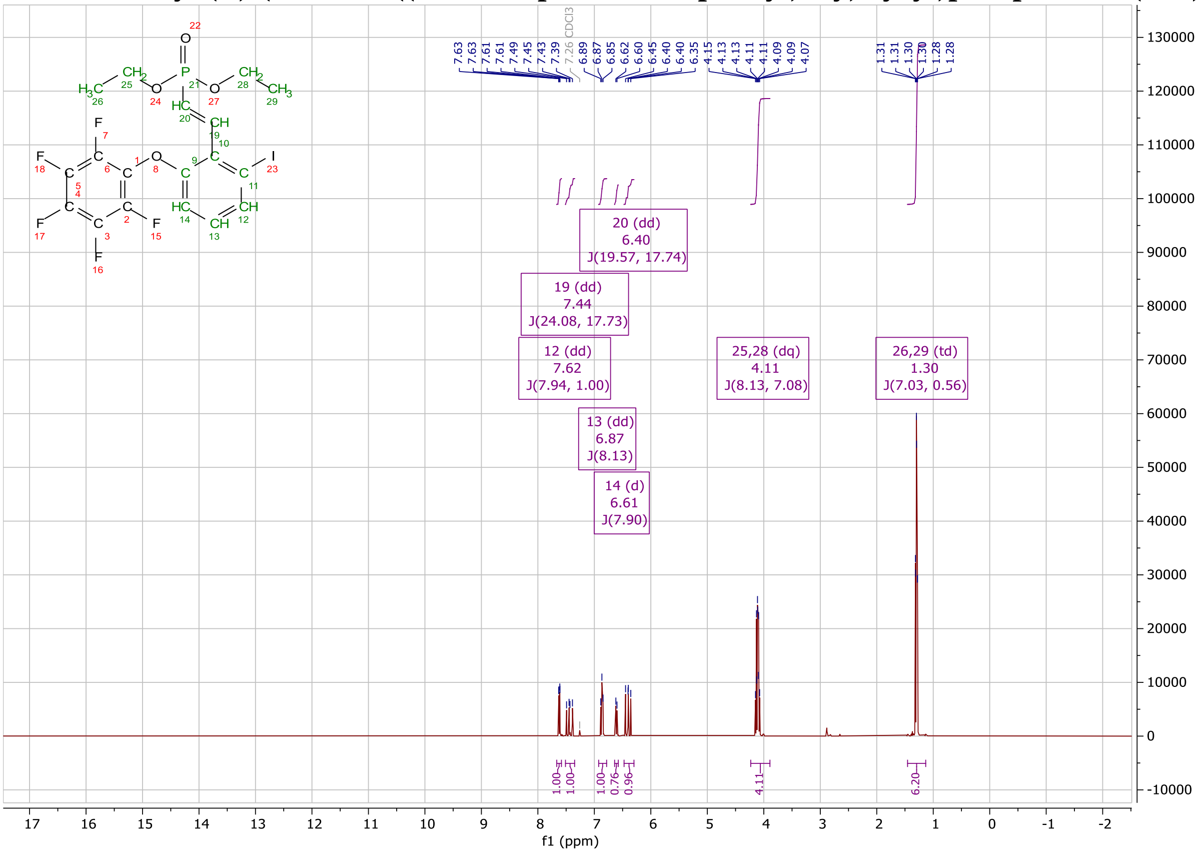

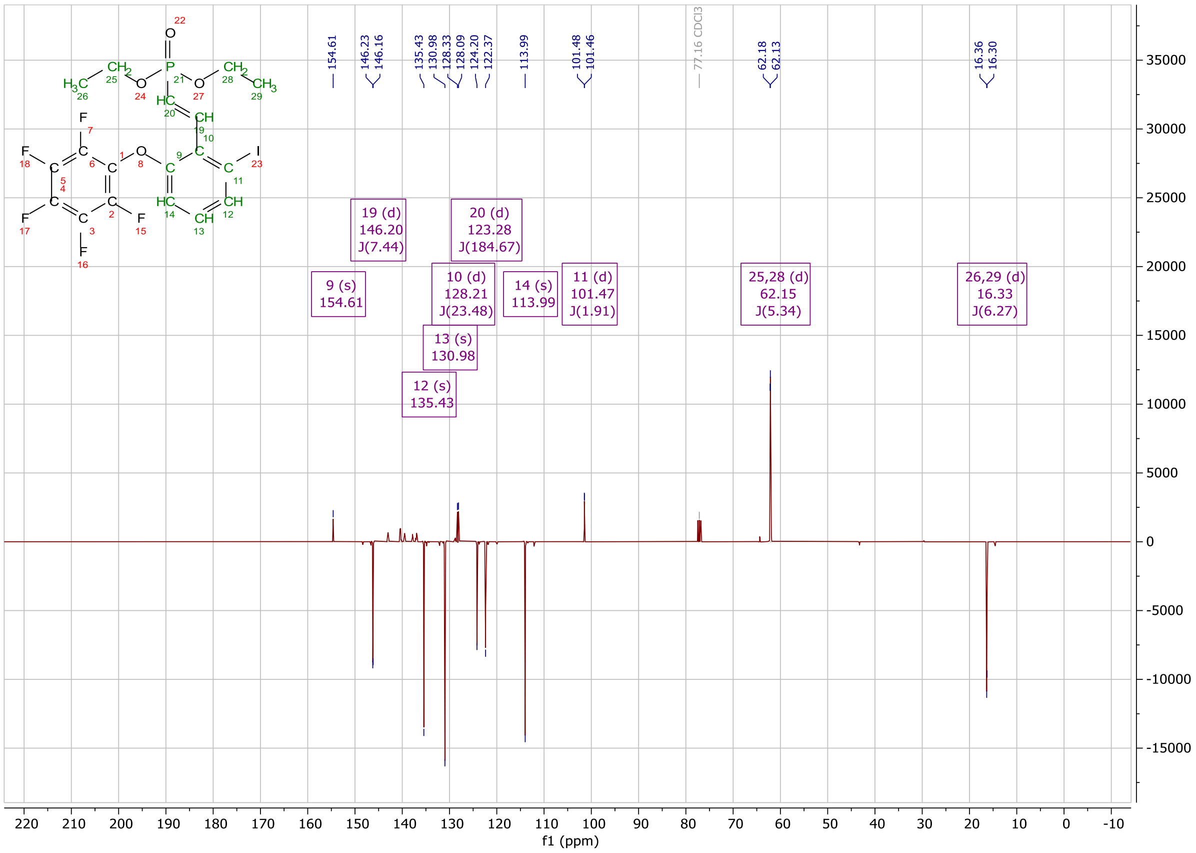

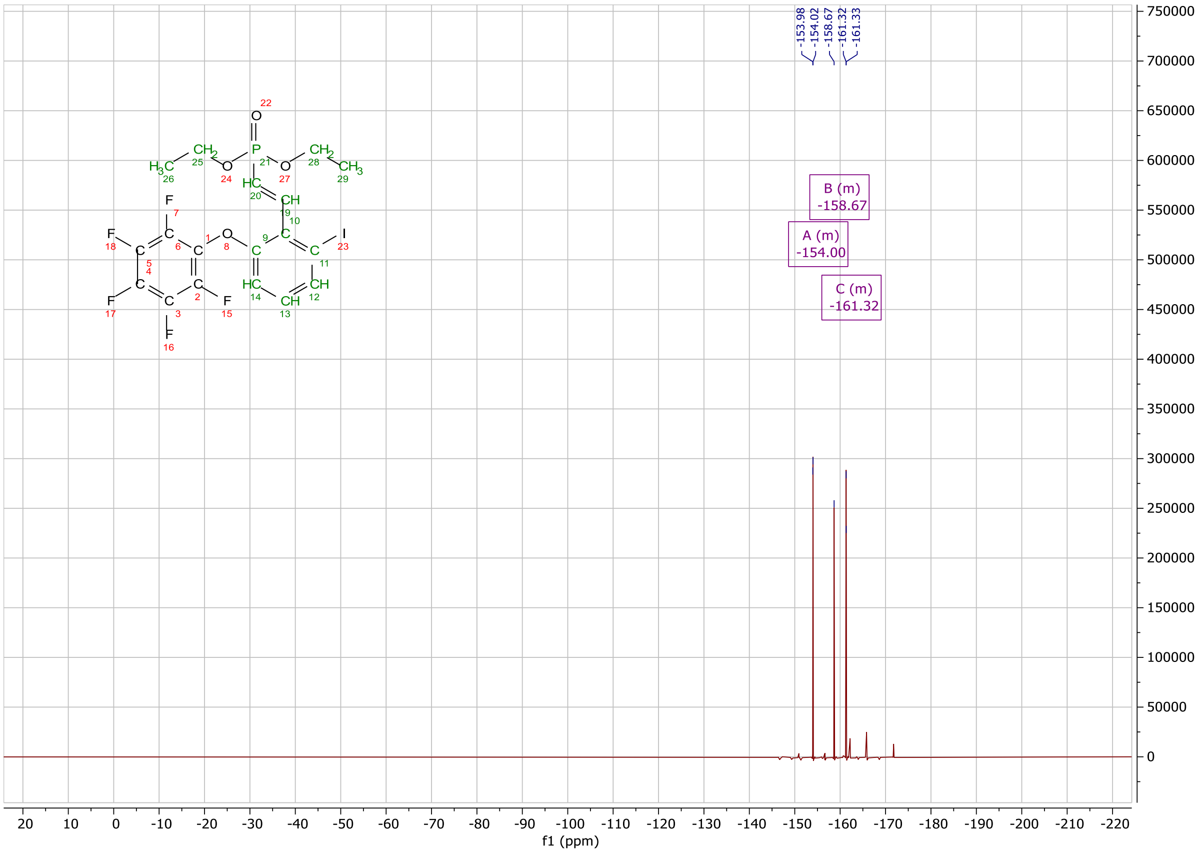
-153.98
-154.02
-158.67
-161.32
-161.33
B (m)
-158.67
A (m)
-154.00
C (m)
-161.32
f1 (ppm)

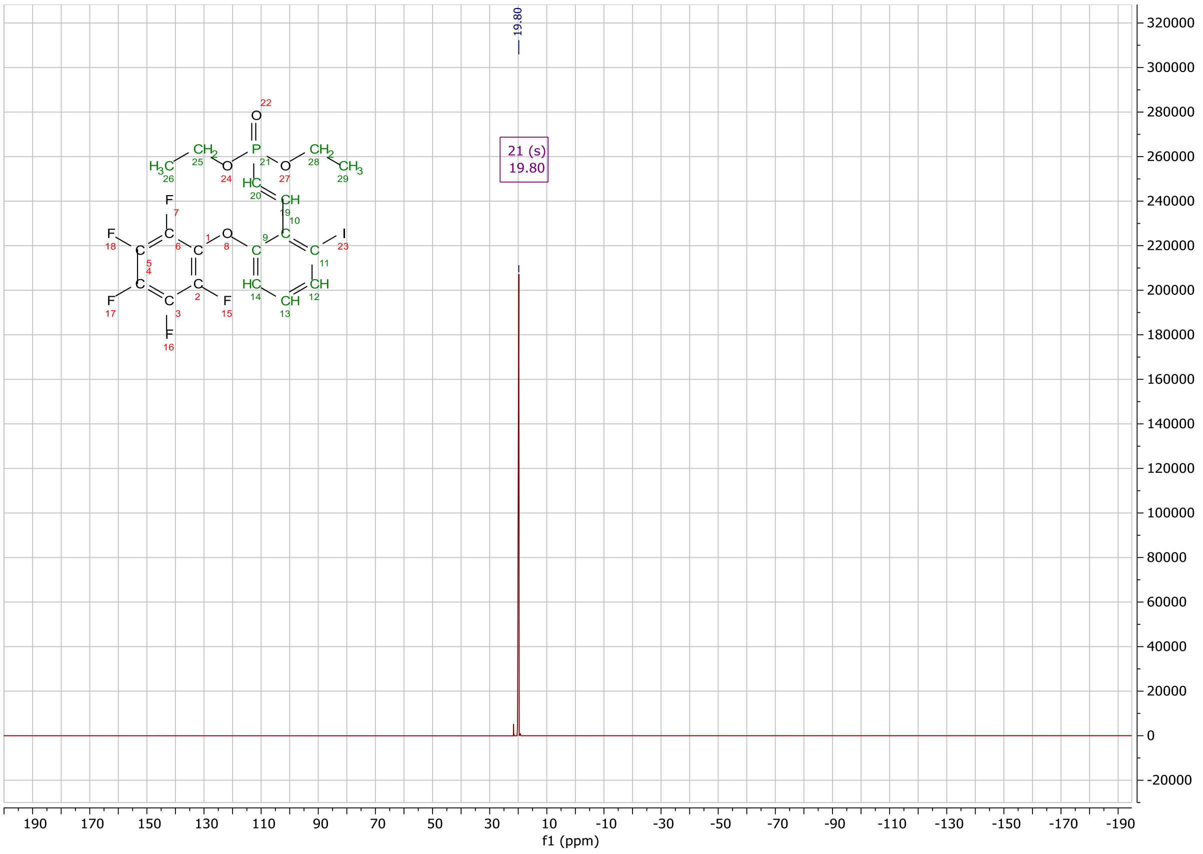
19.80
21 (s)
19.80
f1 (ppm)

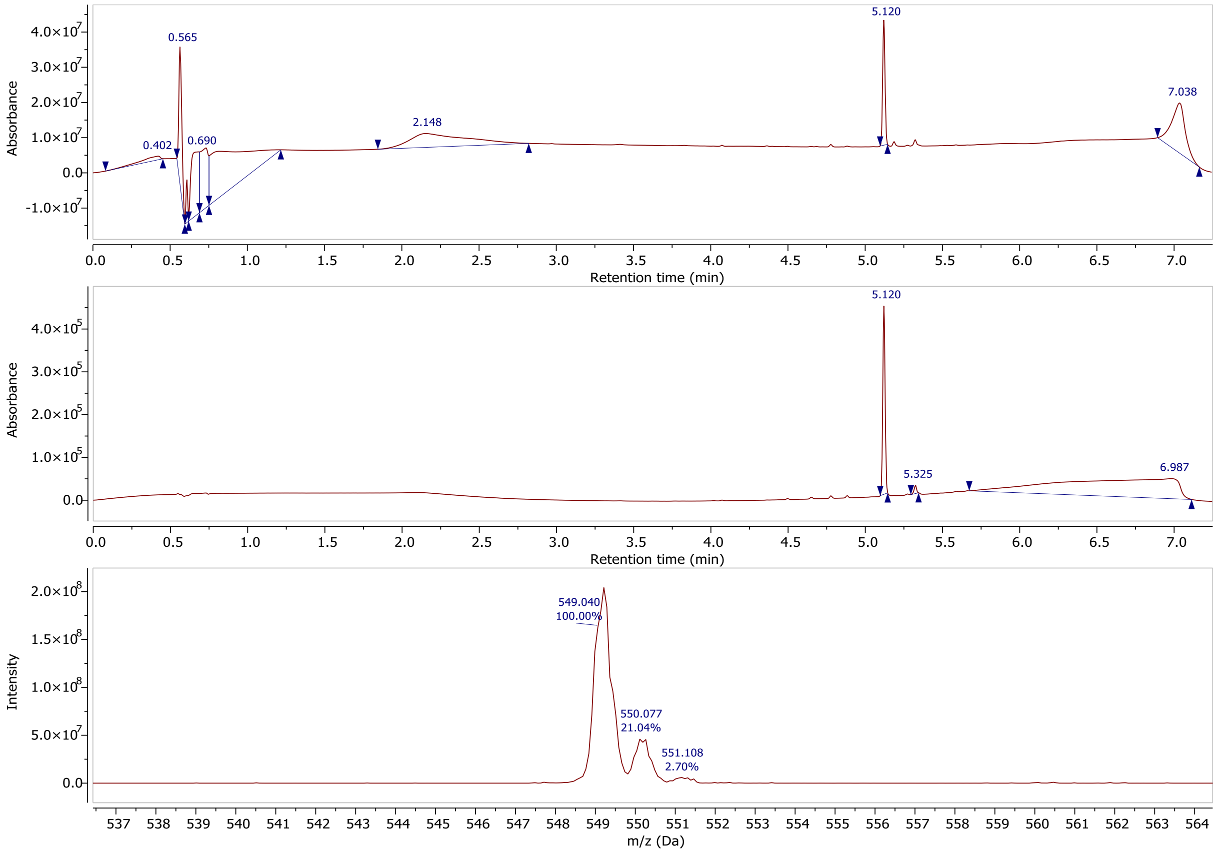

3.2.59 Diethyl (*E*)-(2-iodo-6-((2,3,4,5,6-pentafluorophenyl)methoxy)styryl)phosphonate (43l)

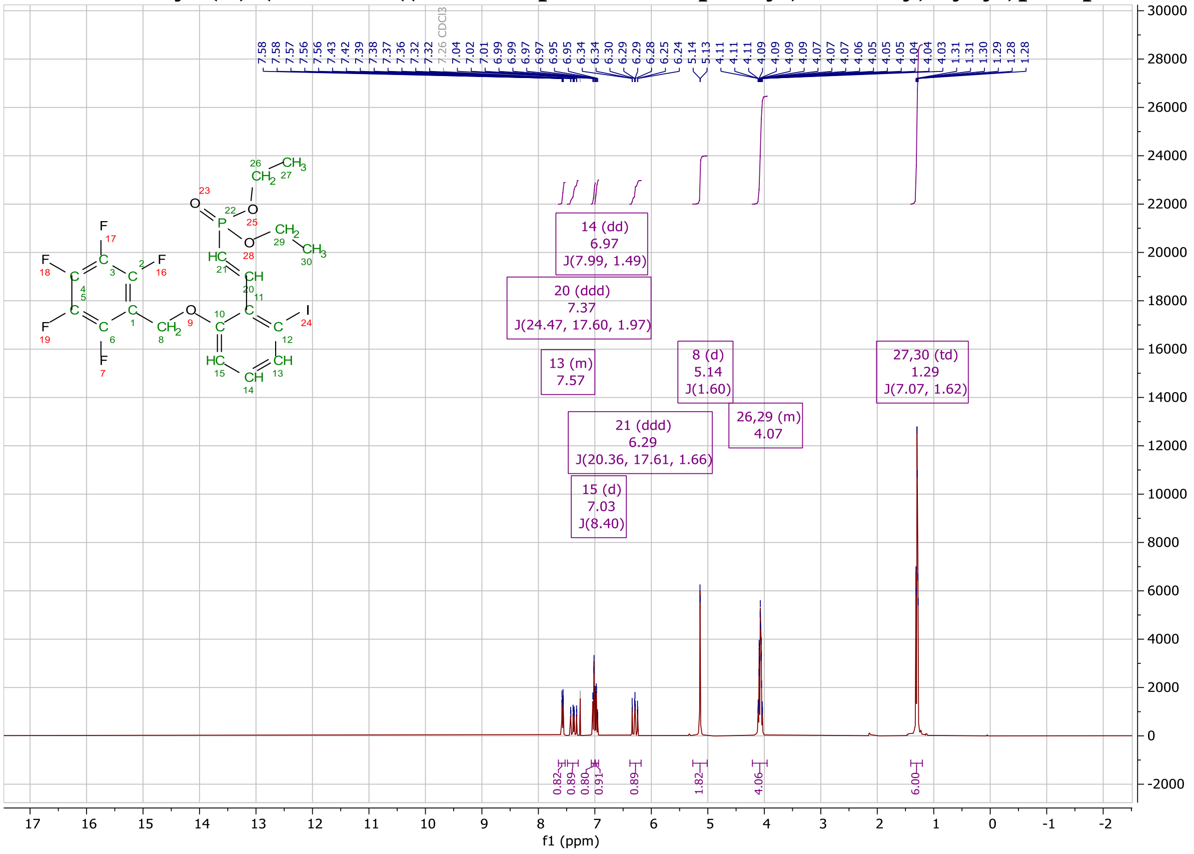

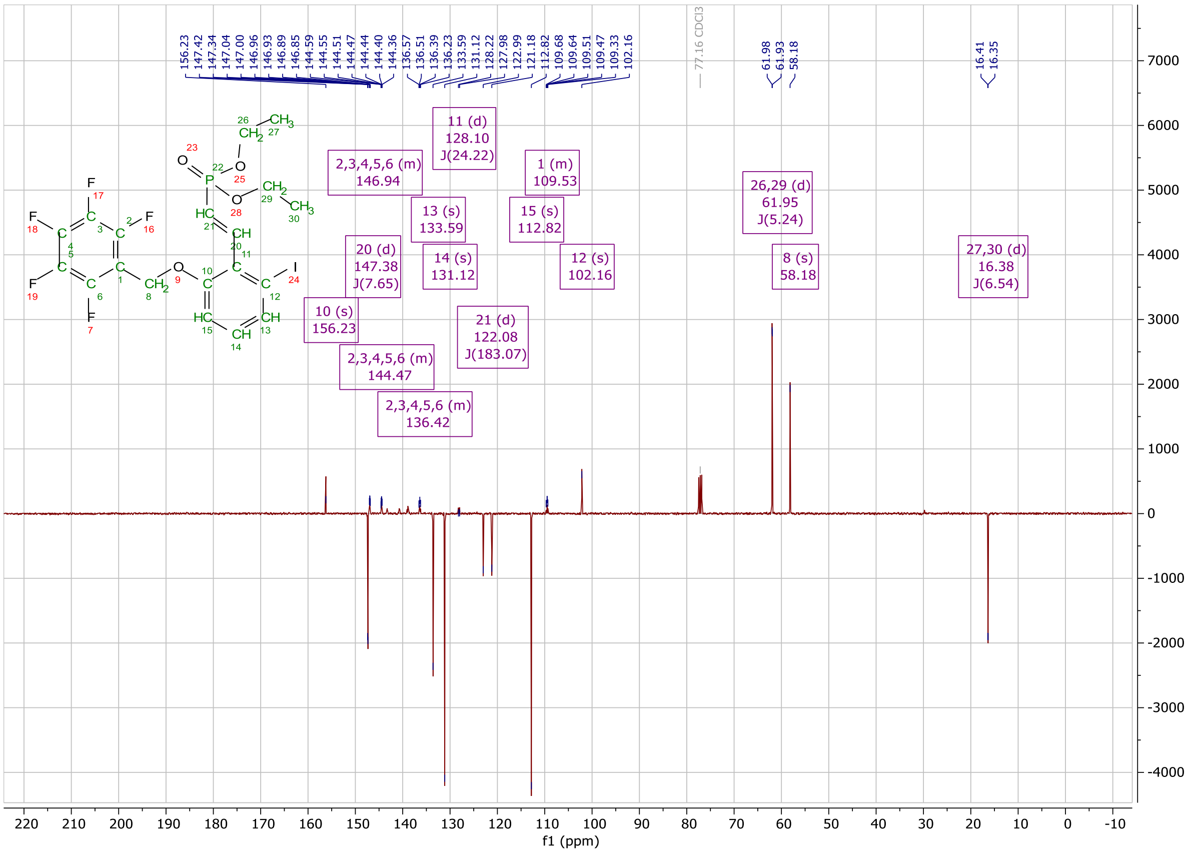

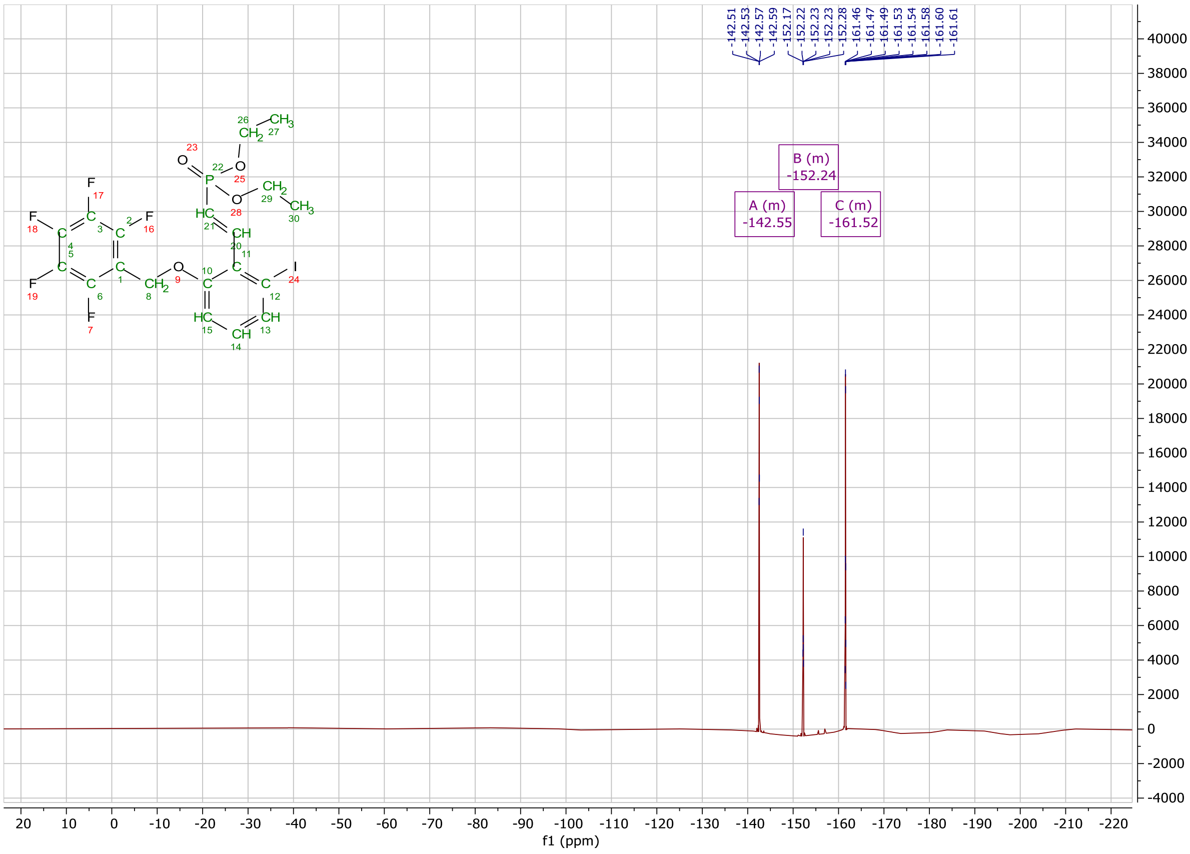

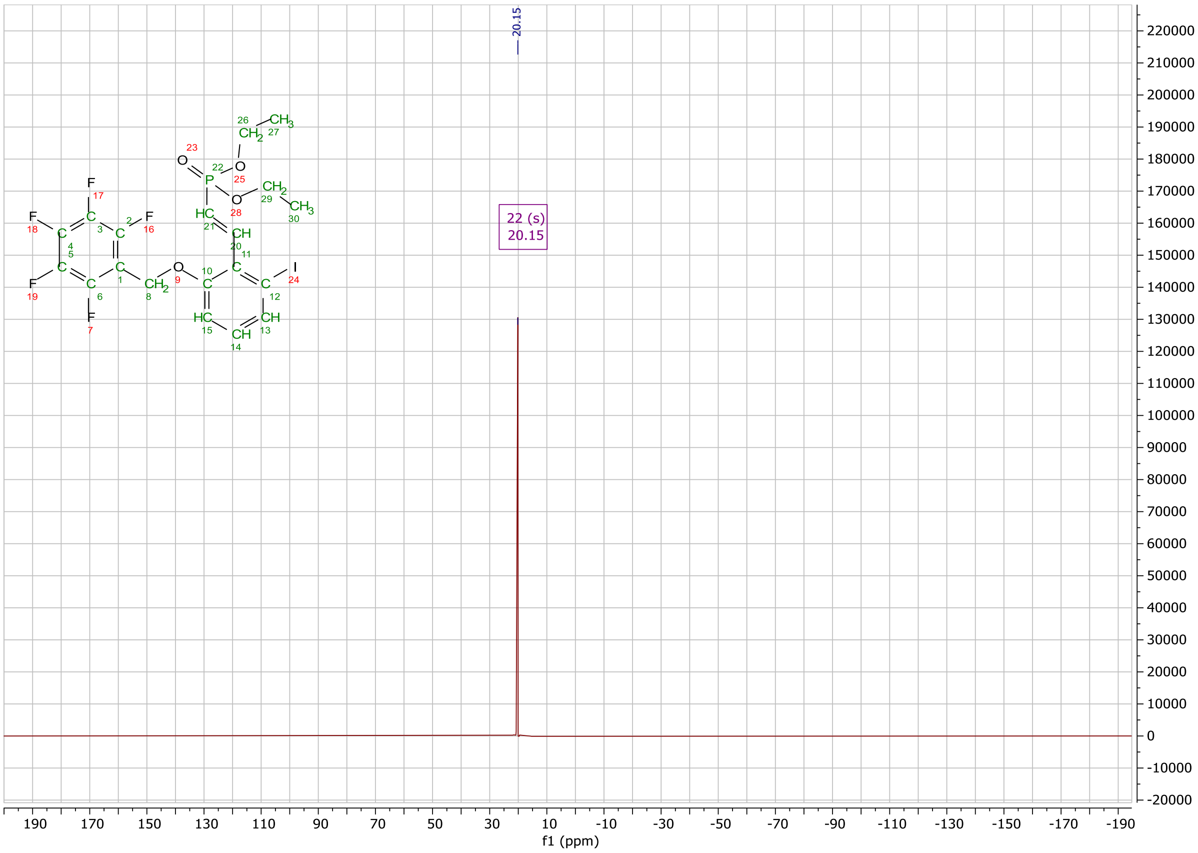
20.15
22 (s)
20.15
f1 (ppm)

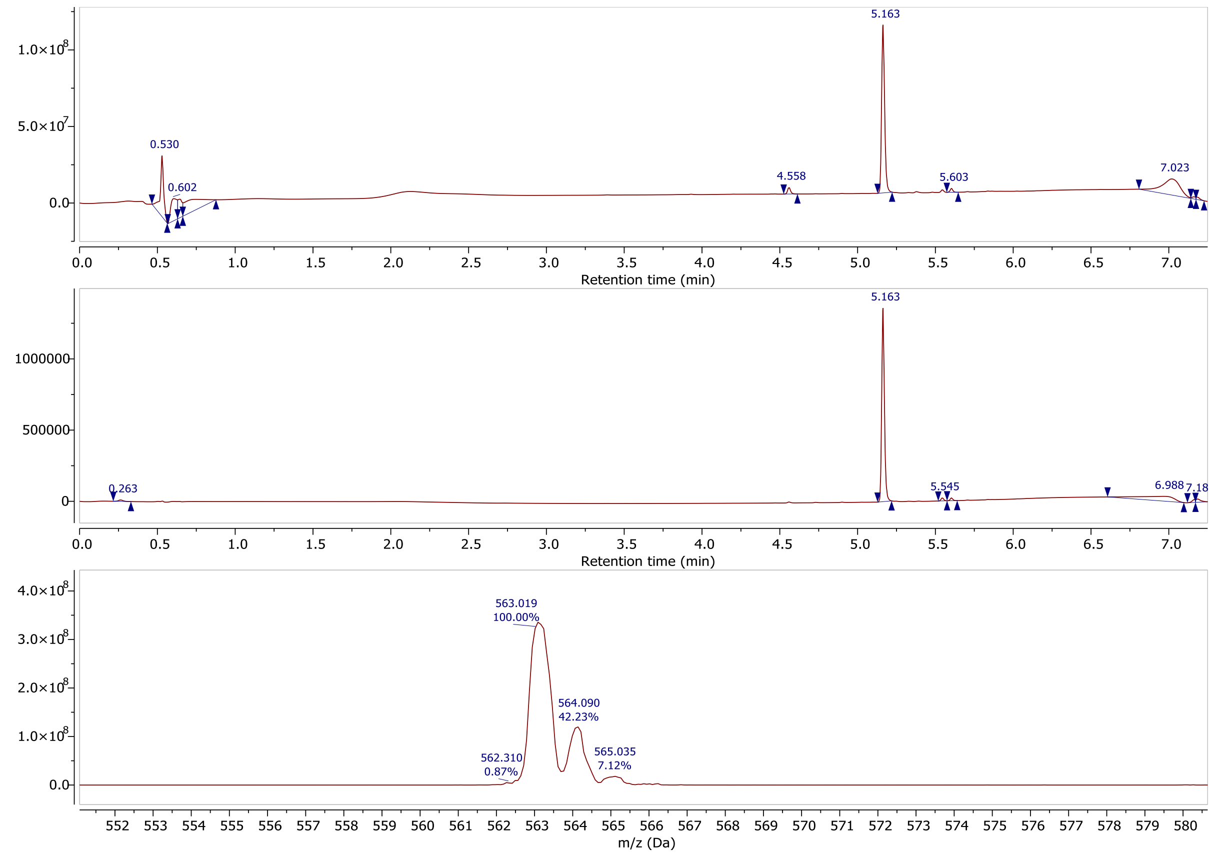
5.163
0.530
0.602
4.558
5.603
7.023
Retention time (min)
5.163
0.263
5.545
6.988
7.18
Retention time (min)
563.019
100.00%
564.090
42.23%
562.310
0.87%
565.035
7.12%
m/z (Da)

3.2.60 Sodium (*E*)-7-((5-hydroxy-2-(2-(phosphonato)vinyl)phenyl)thio)-3,5-dihydro-4*H*-pyrrolo[3,2-*d*]pyrimidin-4-one (45a)

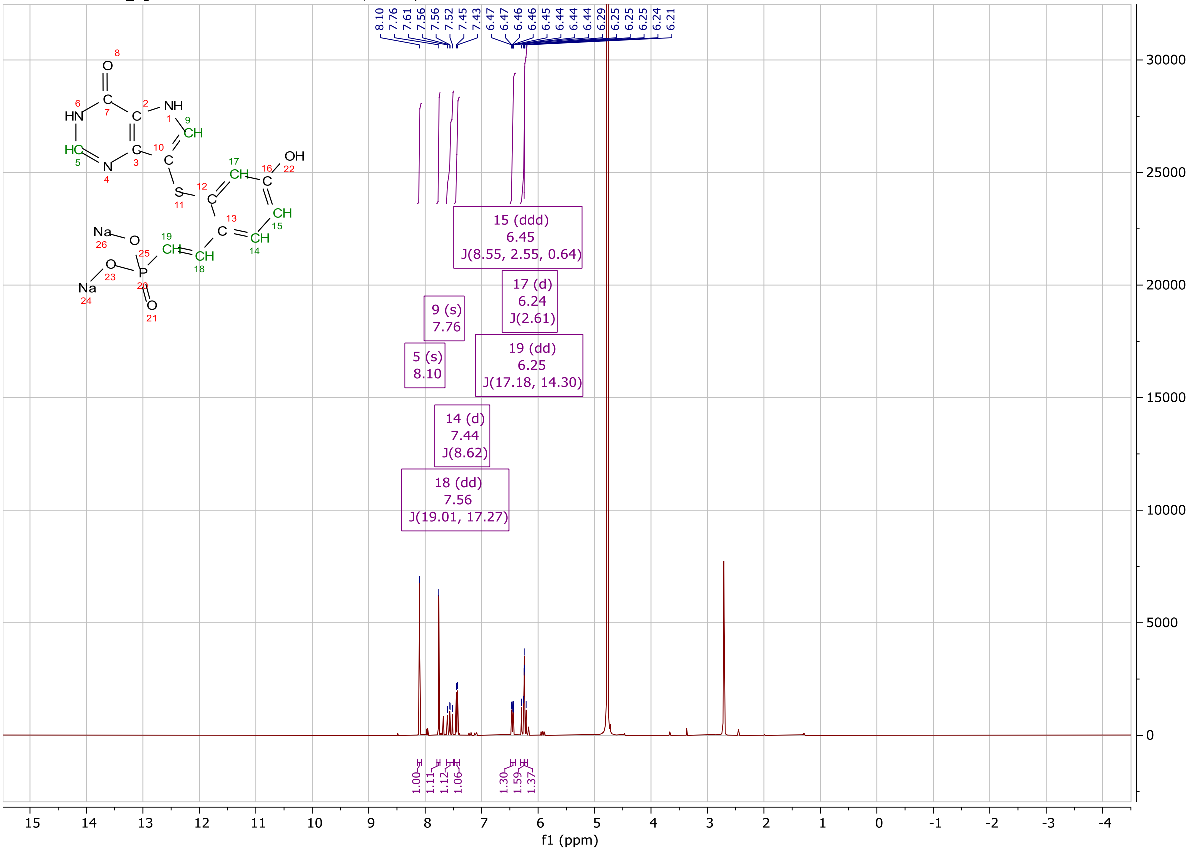

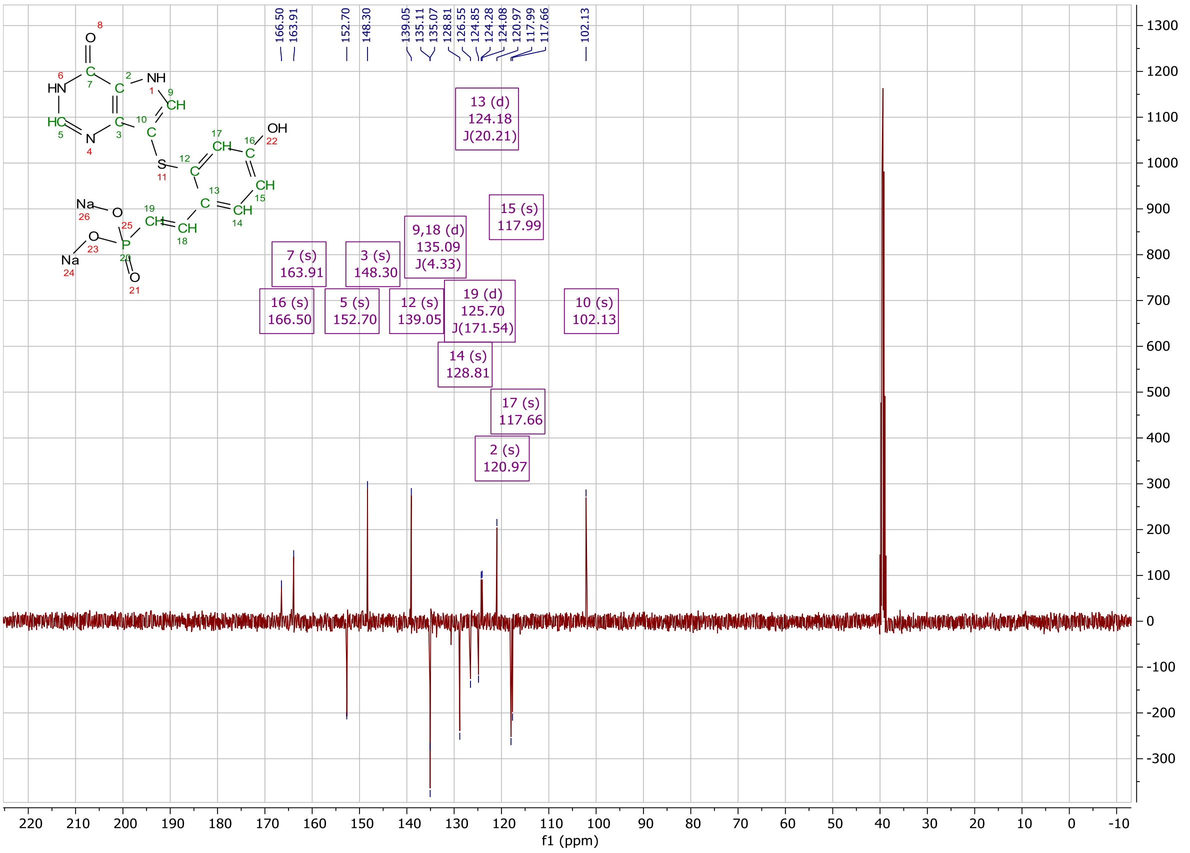

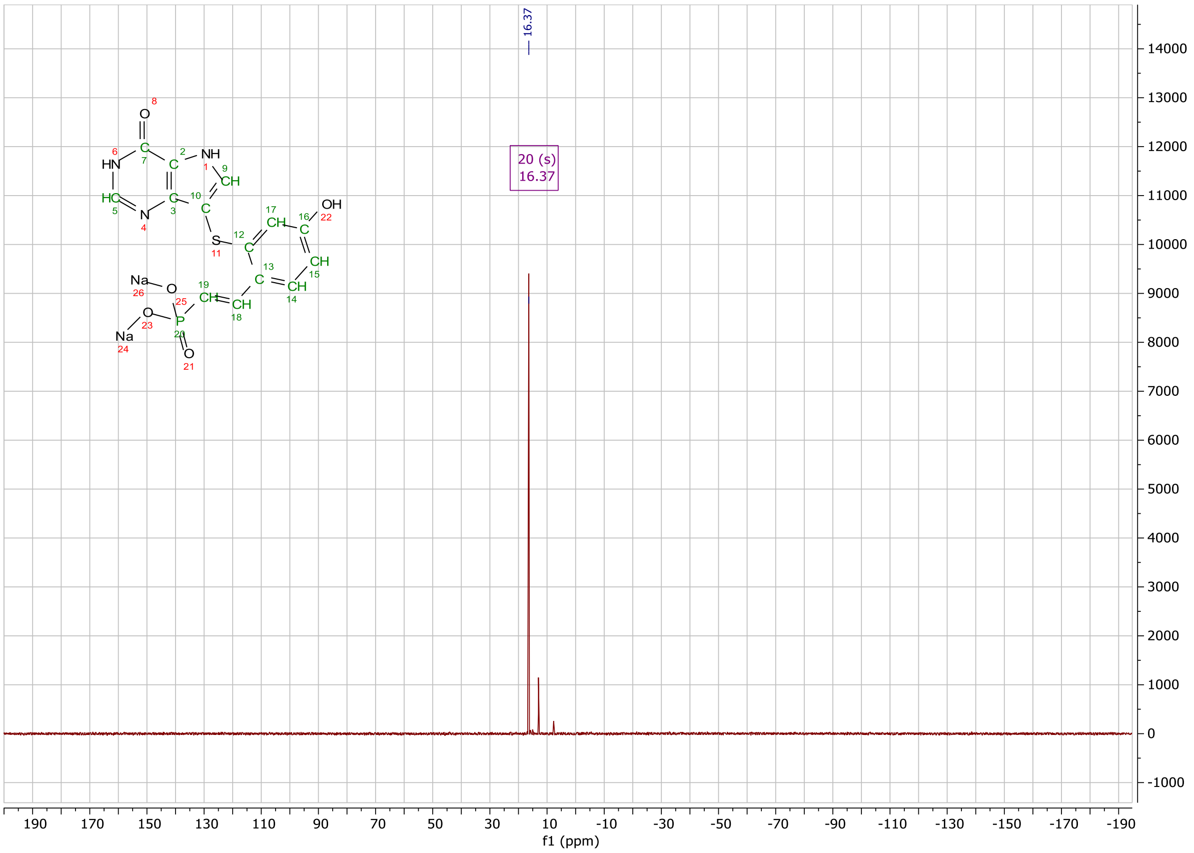
16.37
20 (s)
16.37
f1 (ppm)

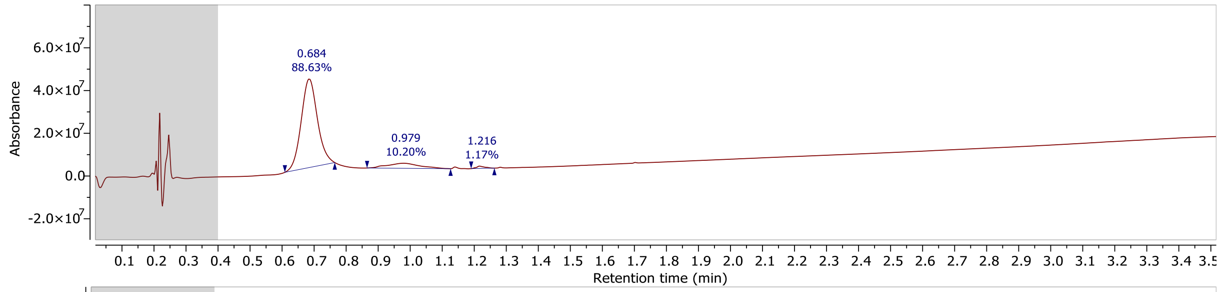
Absorbance
0.684
88.63%
0.979
10.20%
1.216
1.17%
Retention time (min)

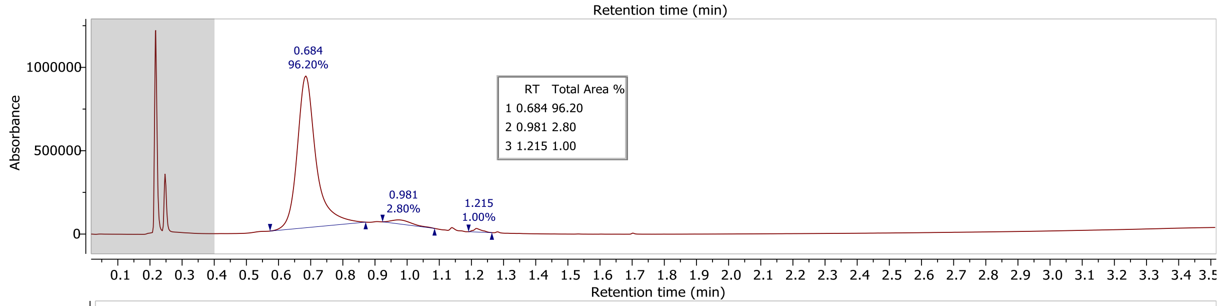
Absorbance
0.684
96.20%
RT Total Area %
1 0.684 96.20
2 0.981 2.80
3 1.215 1.00
0.981
2.80%
1.215
1.00%
Retention time (min)

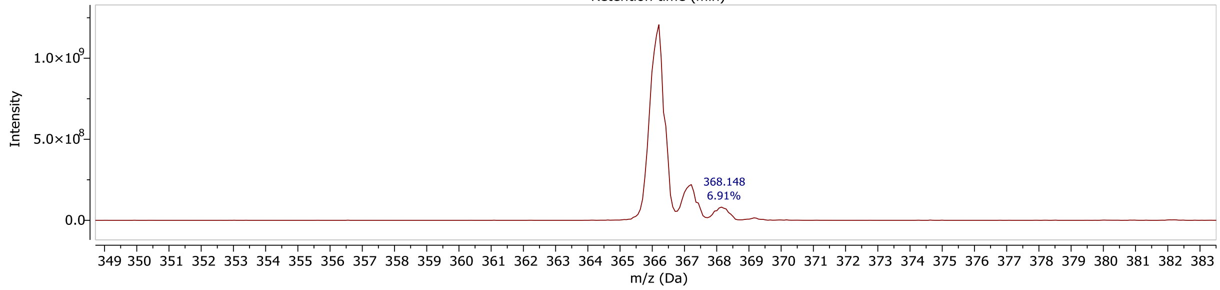
Intensity
368.148
6.91%
m/z (Da)

3.2.61 Sodium (*E*)-7-((5-methoxy-2-(2-(phosphonato)vinyl)phenyl)thio)-3,5-dihydro-4*H*-pyrrolo[3,2-*d*]pyrimidin-4-one (45b)

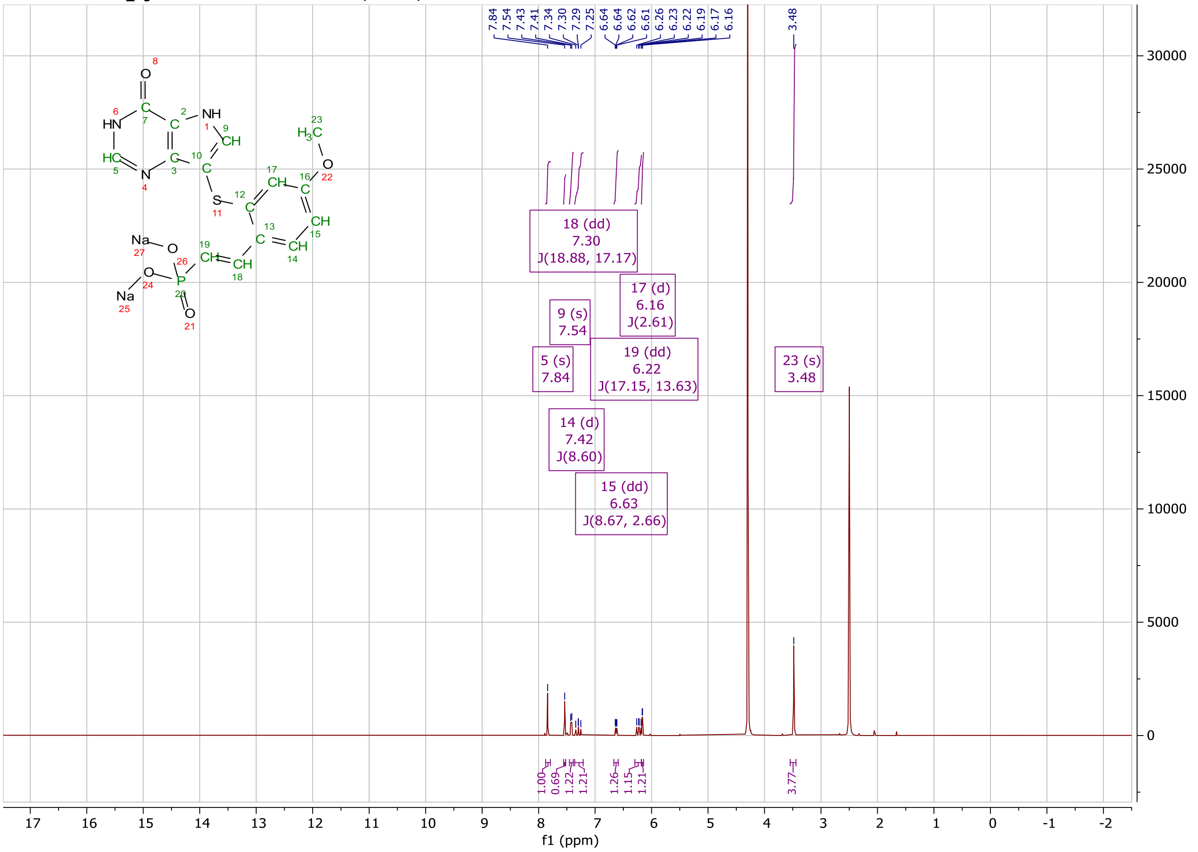

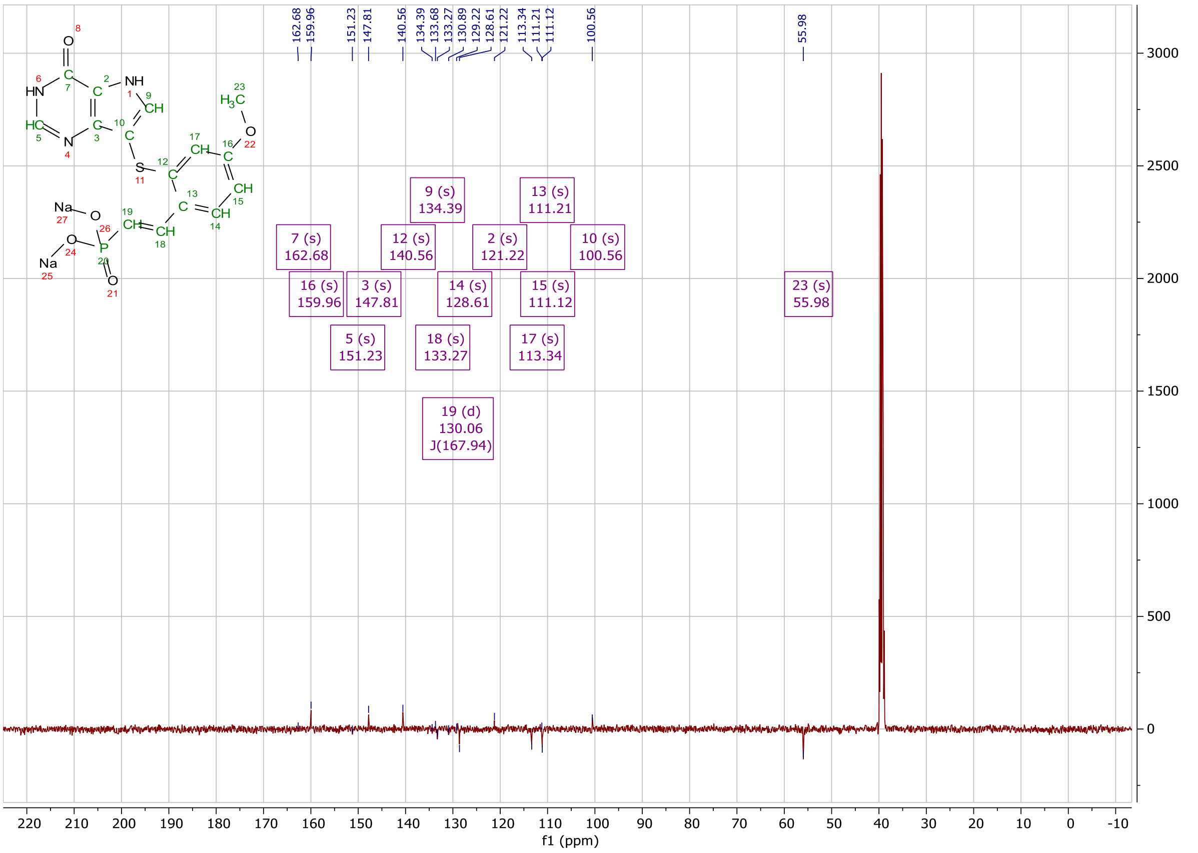

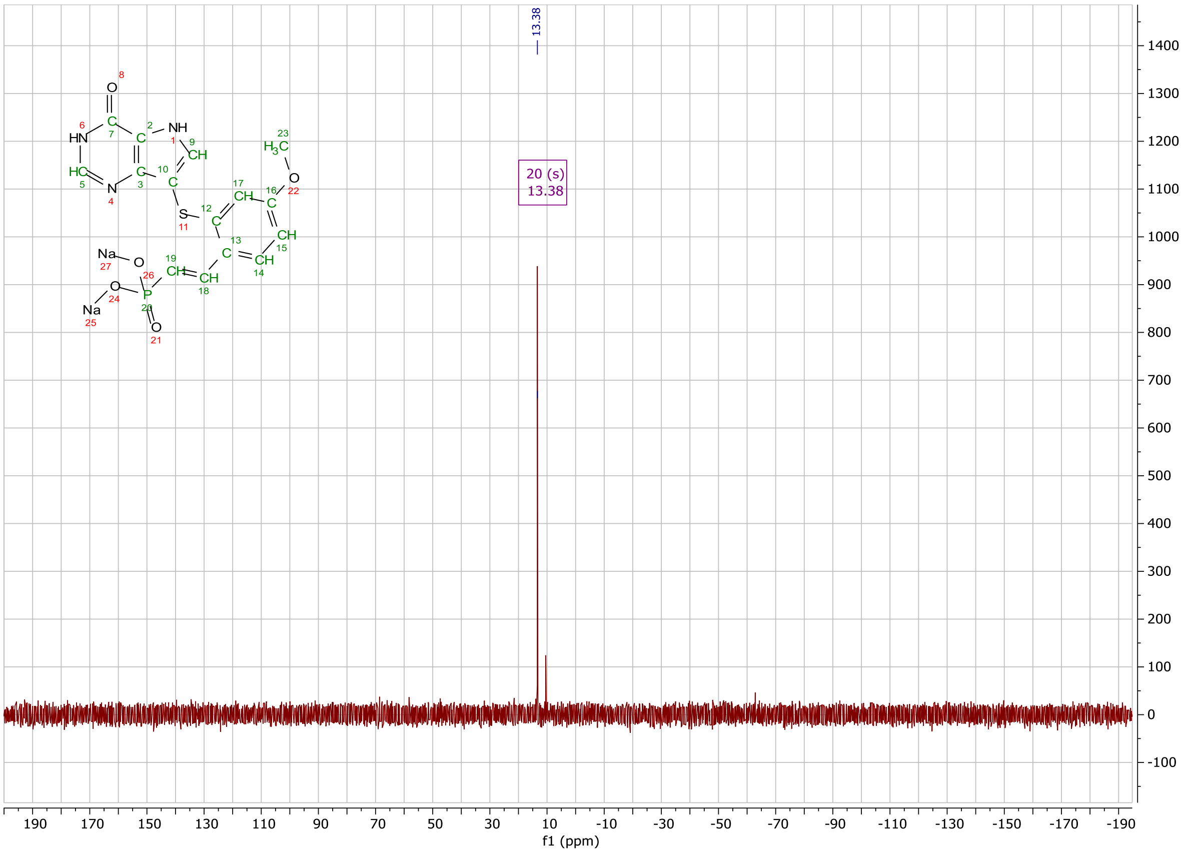
13.38
20 (s)
13.38
f1 (ppm)

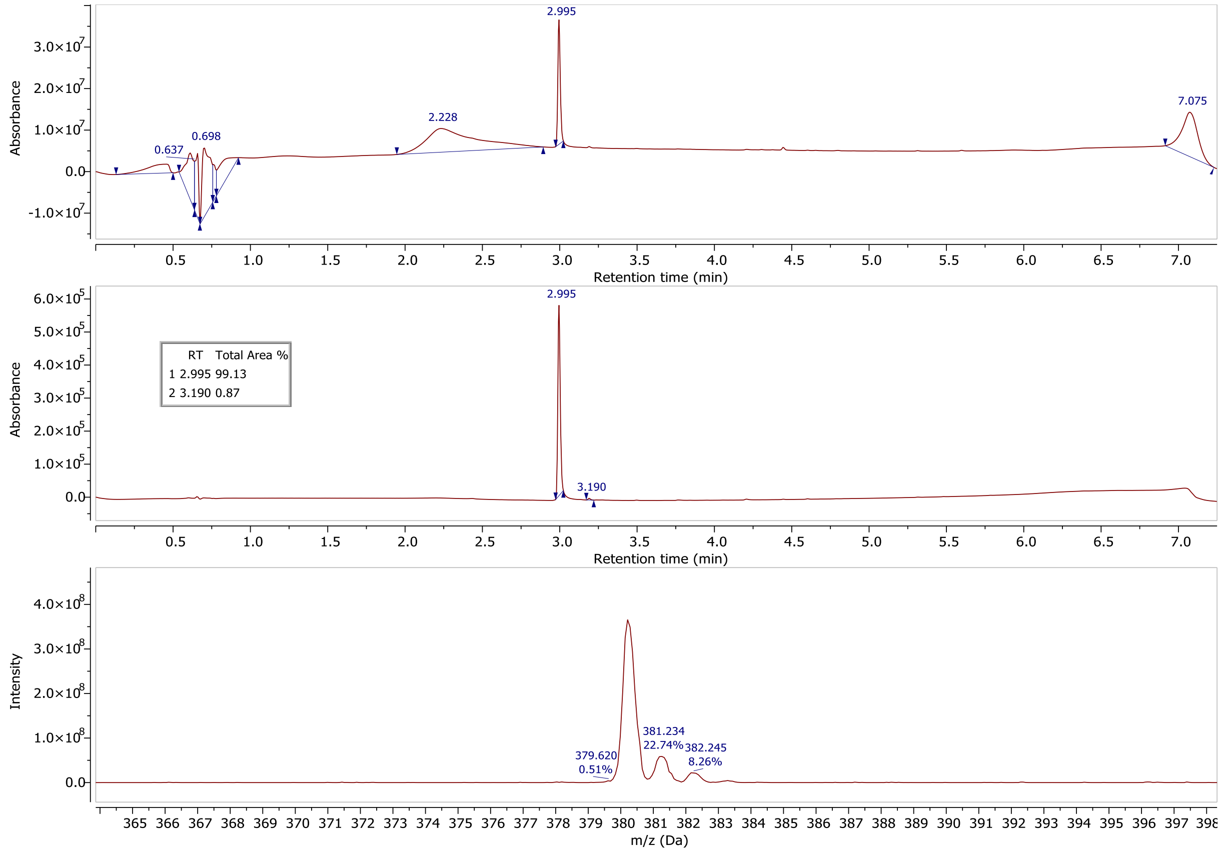
2.995
0.637
0.698
2.228
7.075
Absorbance
Retention time (min)
RT Total Area %
1 2.995 99.13
2 3.190 0.87
3.190
Intensity
379.620
0.51%
381.234
22.74%
382.245
8.26%
m/z (Da)

3.2.62 Sodium (*E*)-7-((5-isopropoxy-2-(2-(phosphonato)vinyl)phenyl)thio)-3,5-dihydro-4*H*-pyrrolo[3,2-*d*]pyrimidin-4-one (45c)

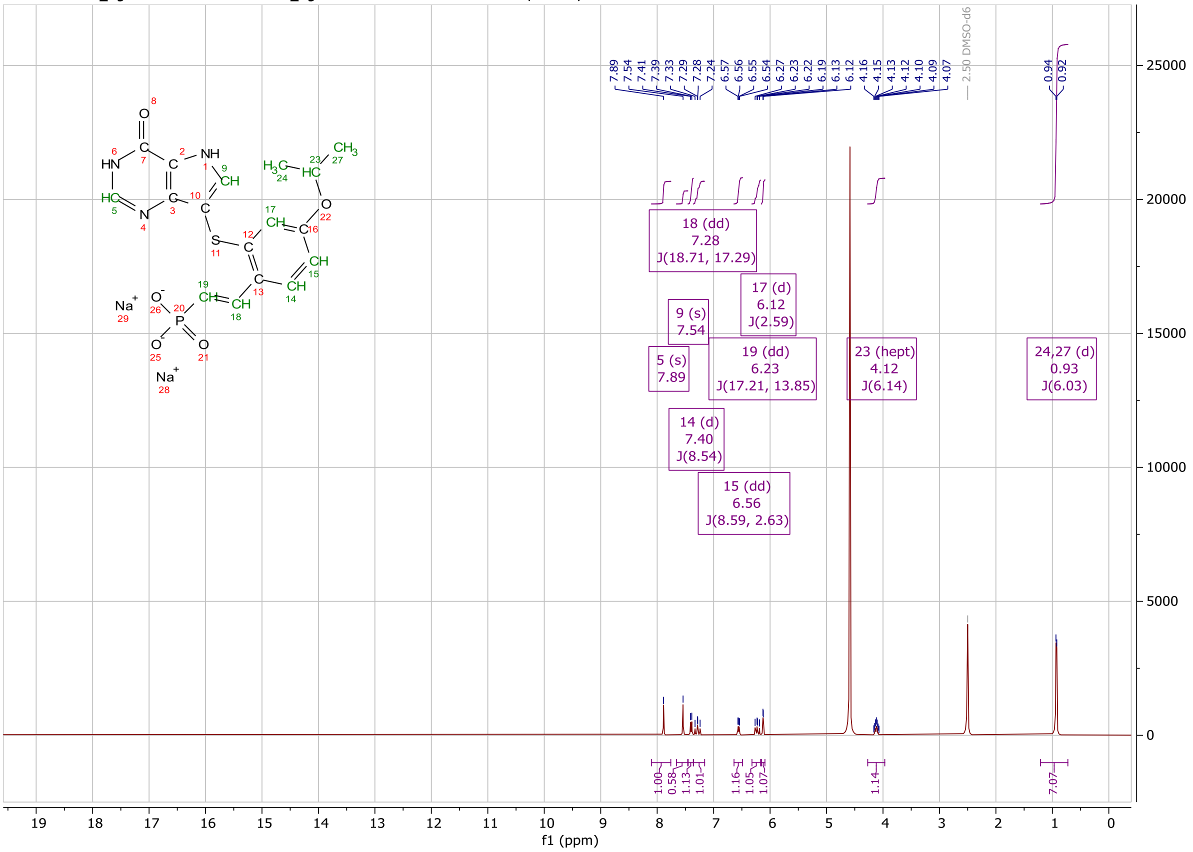

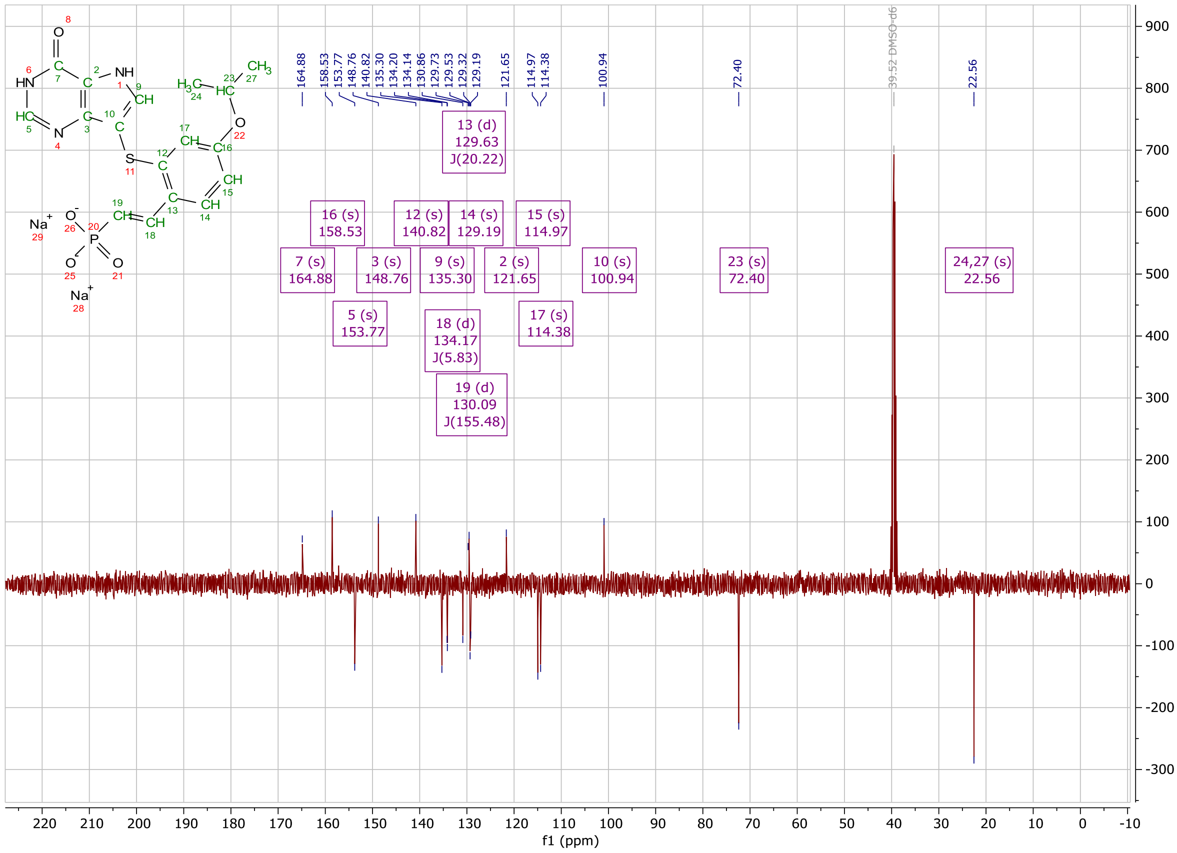

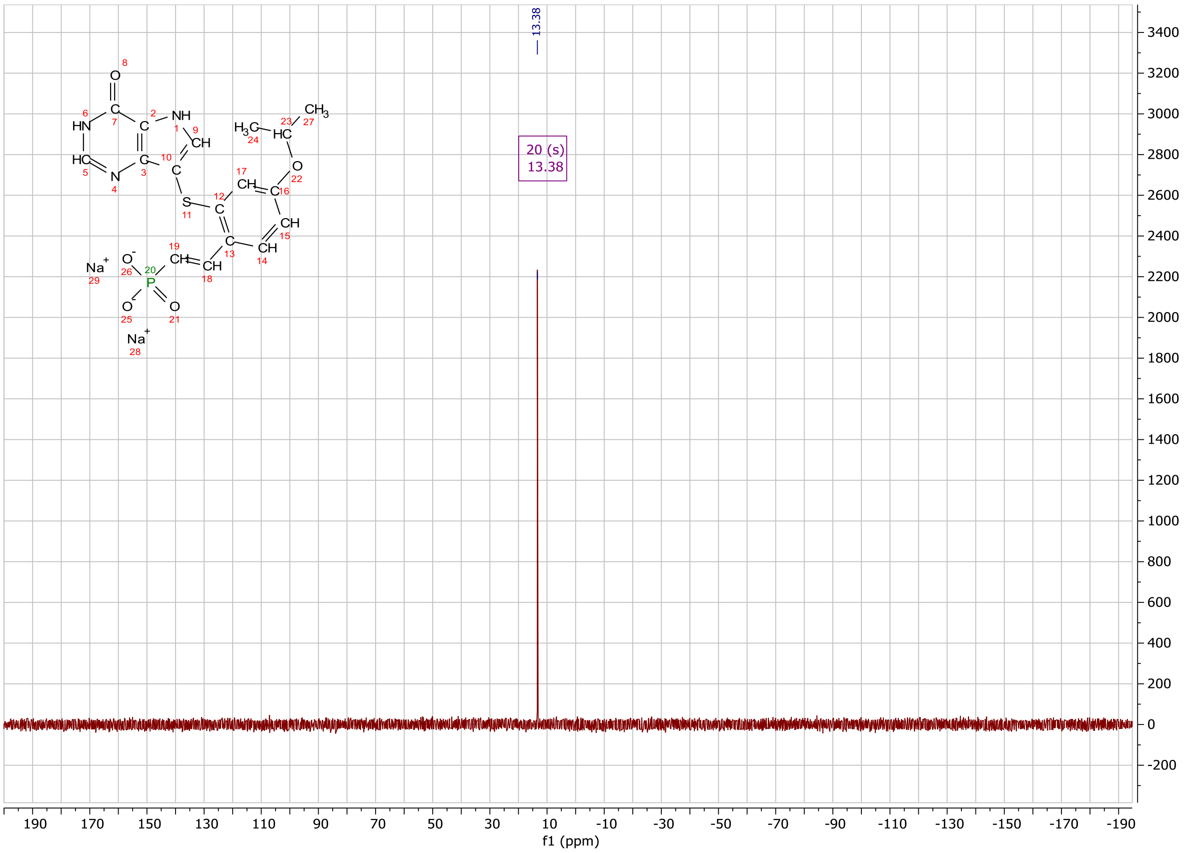
13.38
20 (s)
13.38
f1 (ppm)

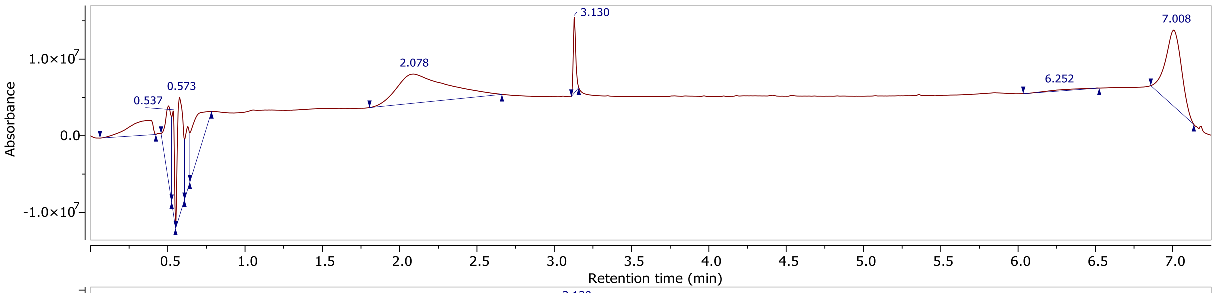
Absorbance
0.537
0.573
2.078
3.130
6.252
7.008
Retention time (min)

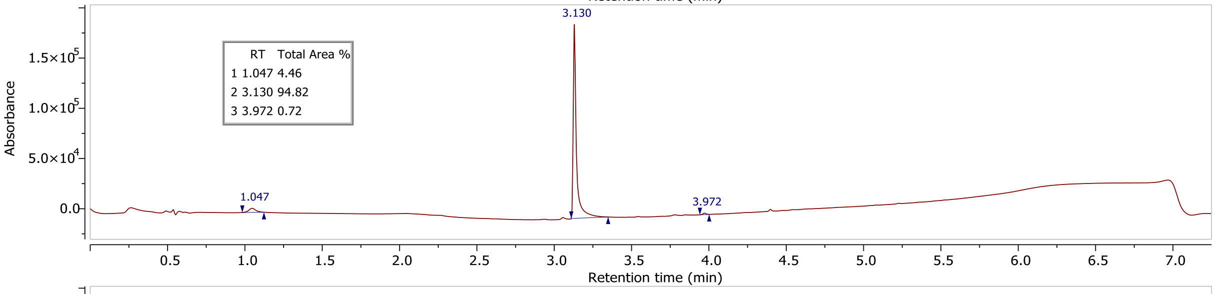
Absorbance
RT Total Area %
1 1.047 4.46
2 3.130 94.82
3 3.972 0.72
1.047
3.130
3.972
Retention time (min)

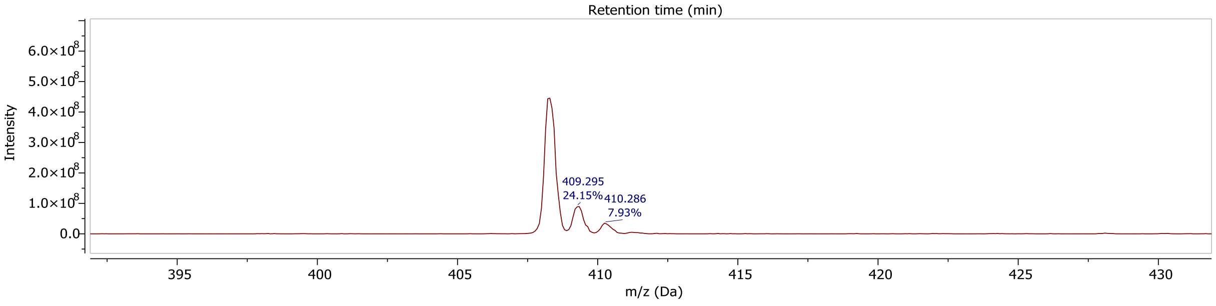
Intensity
409.295
24.15%
410.286
7.93%
m/z (Da)

3.2.63 Sodium (*E*)-7-((5-(2,3,4,5,6-pentafluorophenyl)oxy-2-(2-(phosphonato)vinyl)phenyl)thio)-3,5-dihydro-4*H*-pyrrolo[3,2-*d*]pyrimidin-4-one (45d)

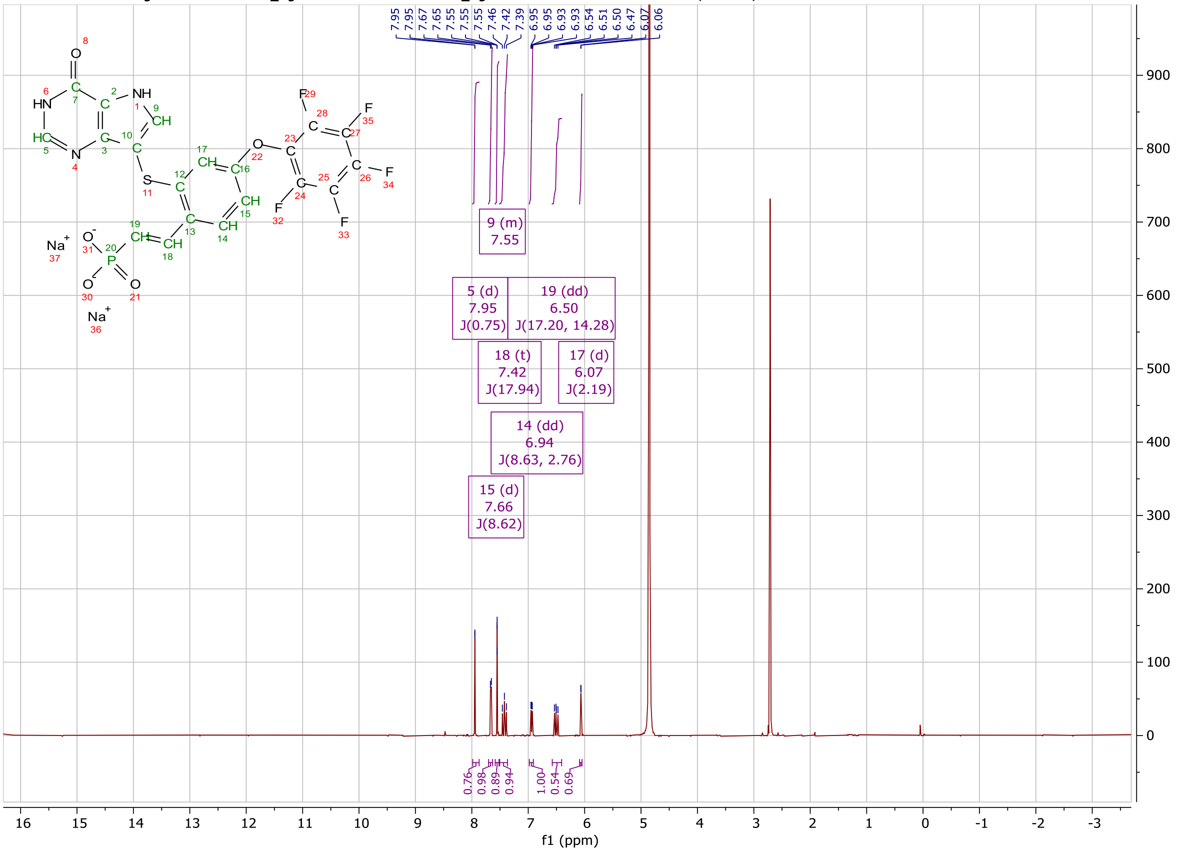

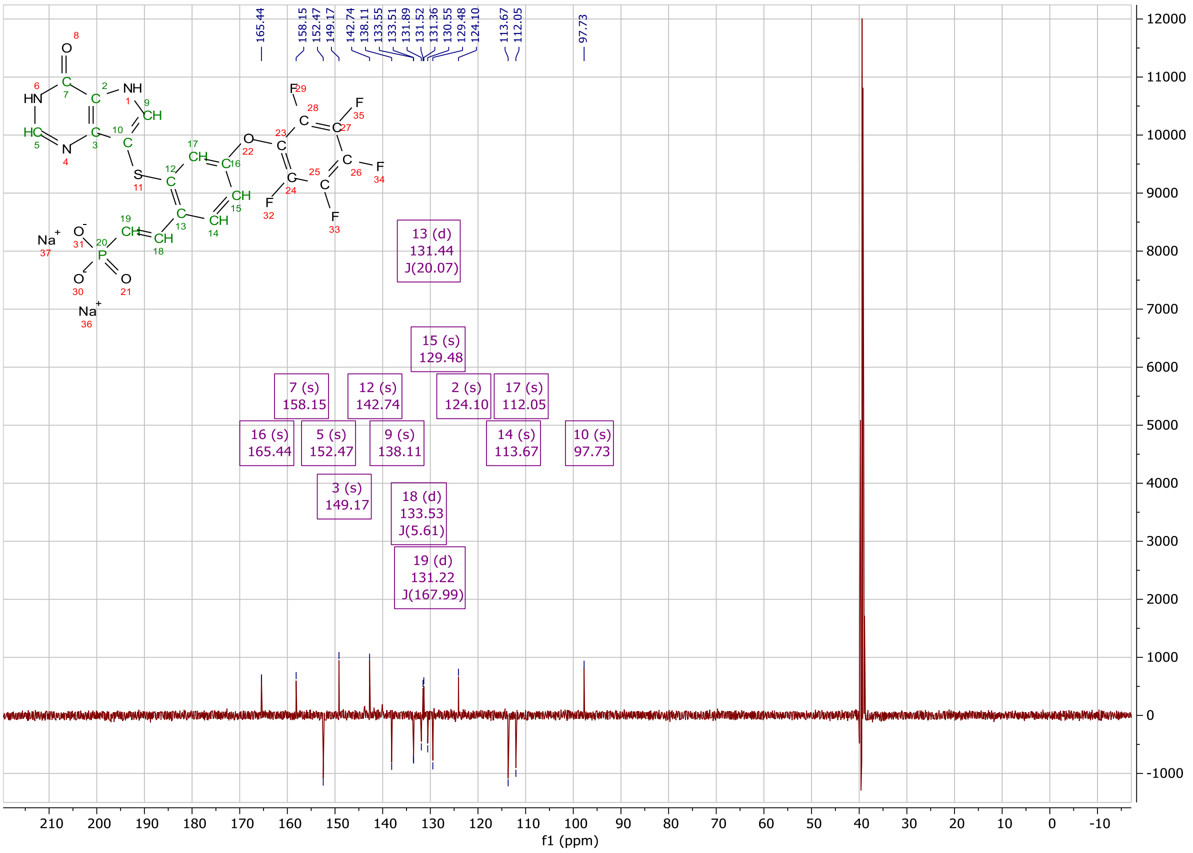

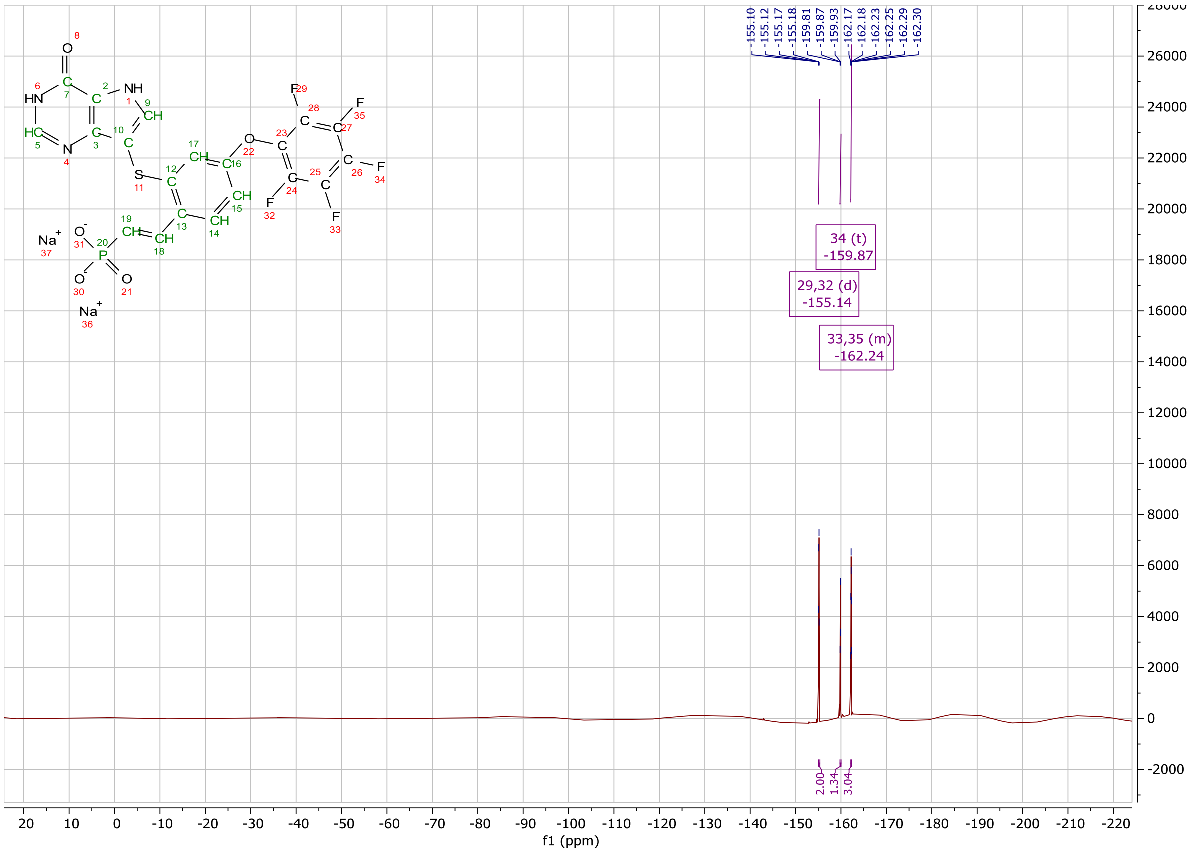
-155.10
-155.12
-155.17
-155.18
-159.81
-159.87
-159.93
-162.17
-162.18
-162.23
-162.25
-162.29
-162.30
34 (t)
-159.87
29,32 (d)
-155.14
33,35 (m)
-162.24
2.00
1.34
3.04
f1 (ppm)

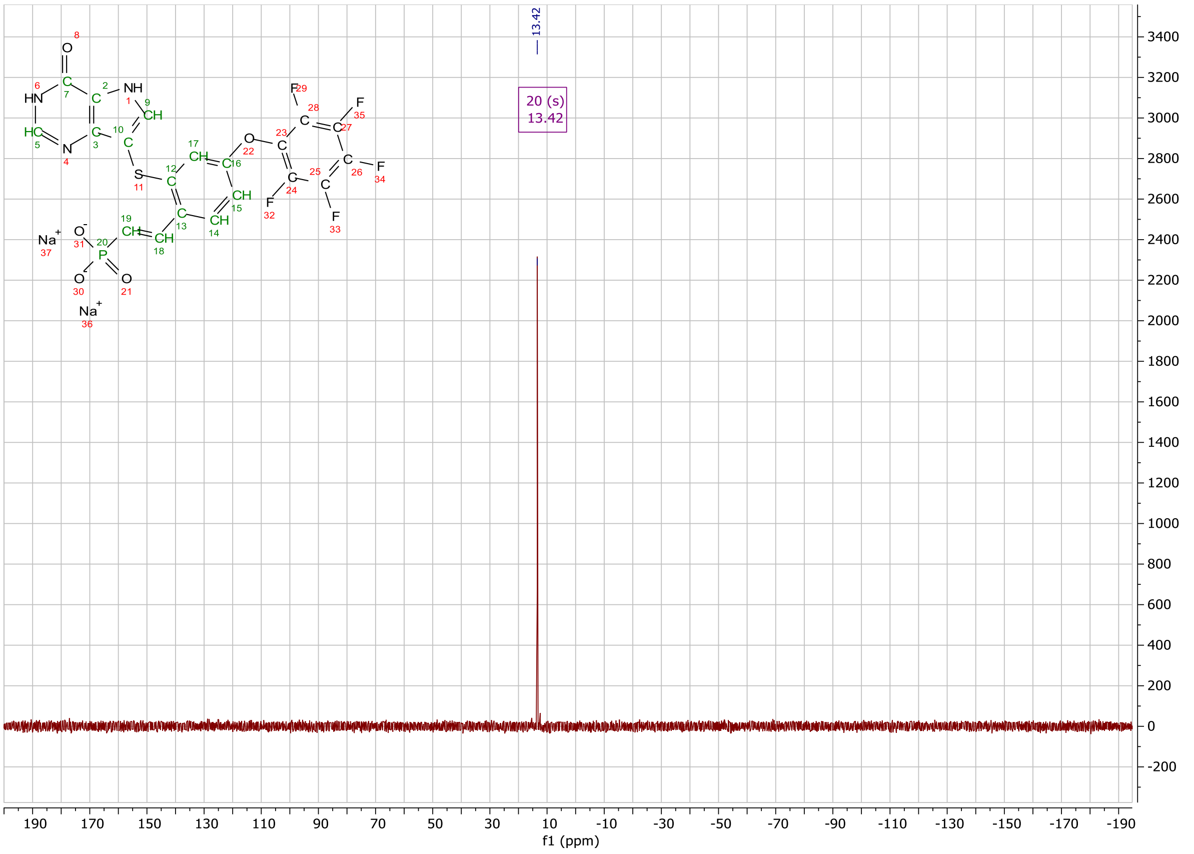
13.42
20 (s)
13.42
f1 (ppm)

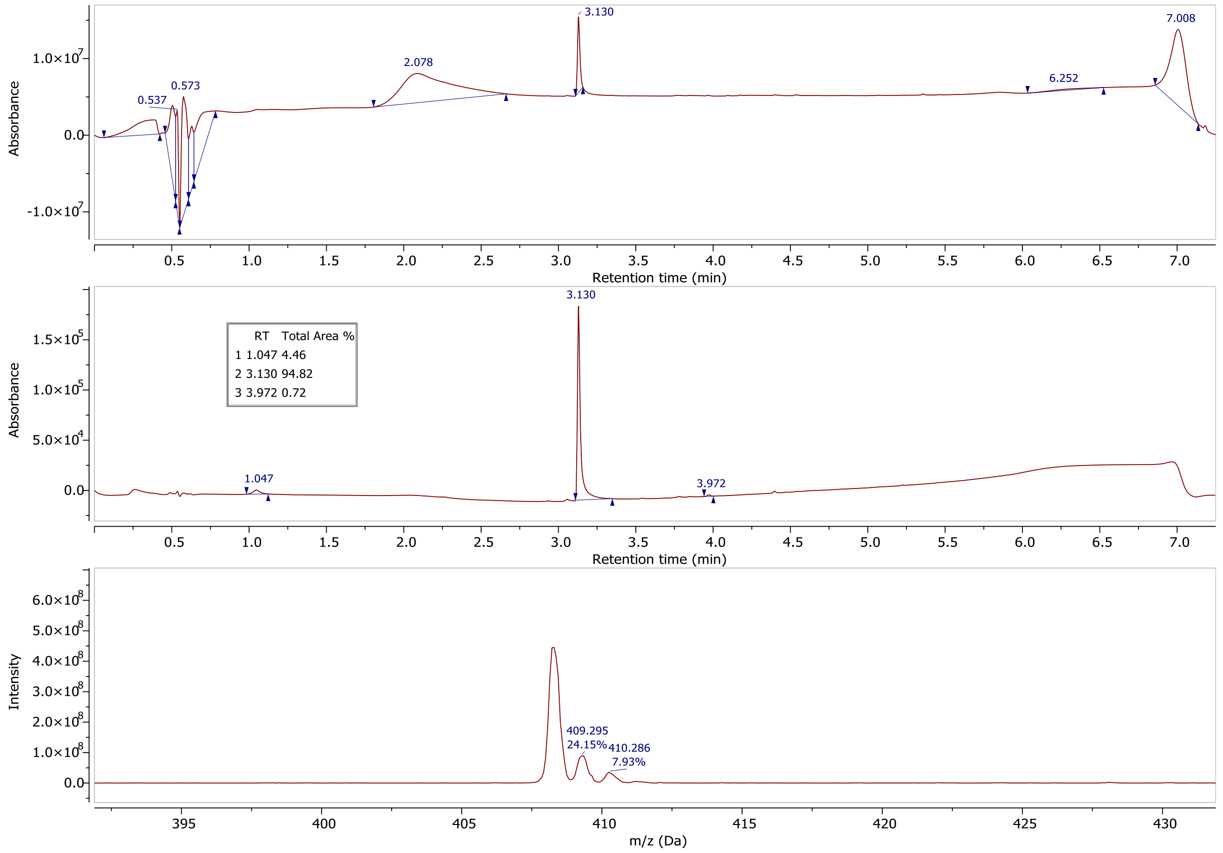

3.2.64 Sodium (*E*)-7-((5-(2,3,4,5,6-pentafluorofluorophenyl)methoxy-2-(2-(phosphonato)vinyl)phenyl)thio)-3,5-dihydro-4*H*-pyrrolo[3,2-*d*]pyrimidin-4-one (45e)

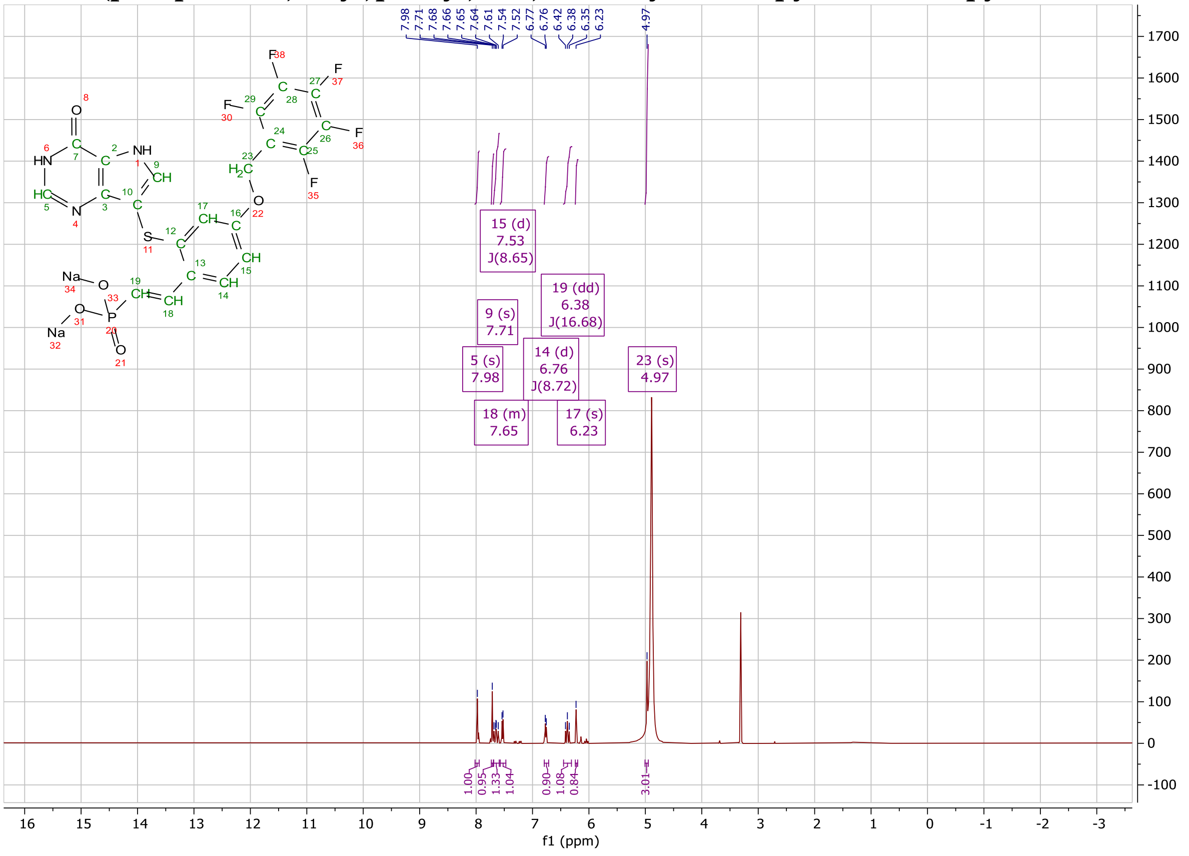

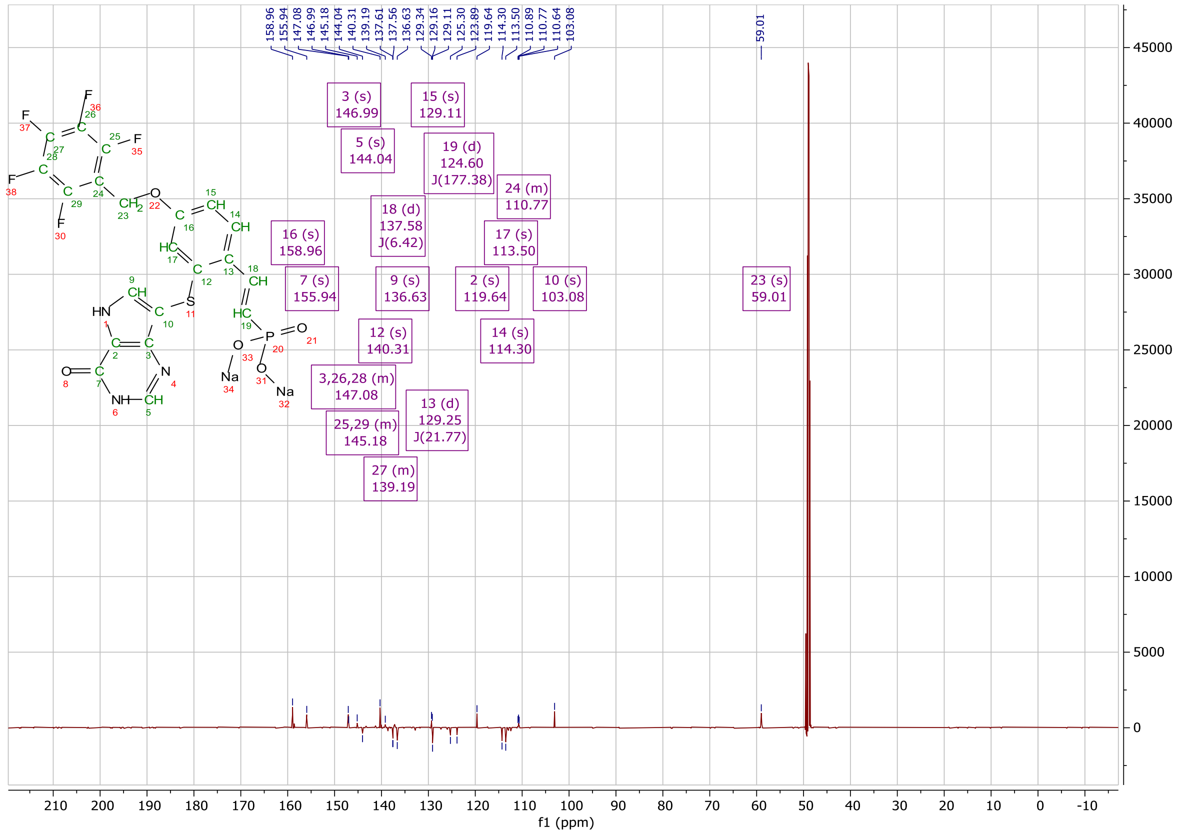

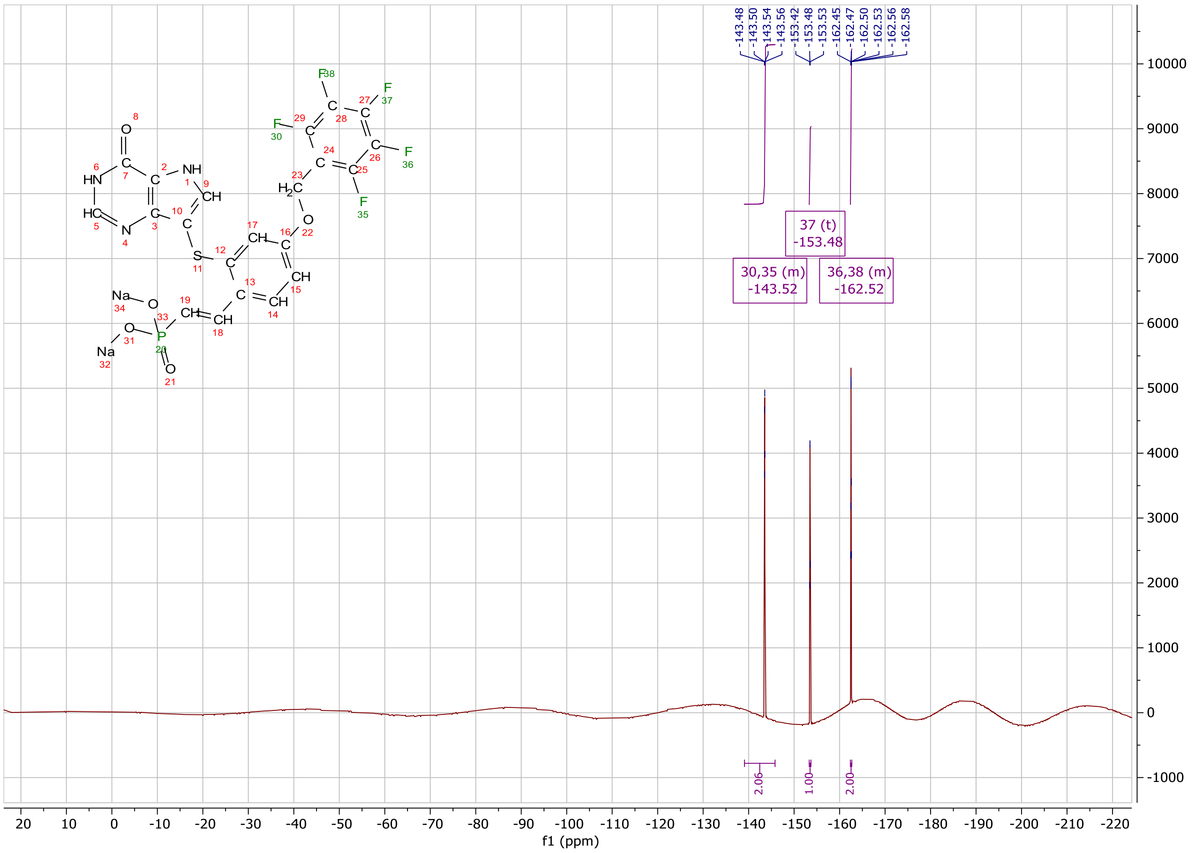

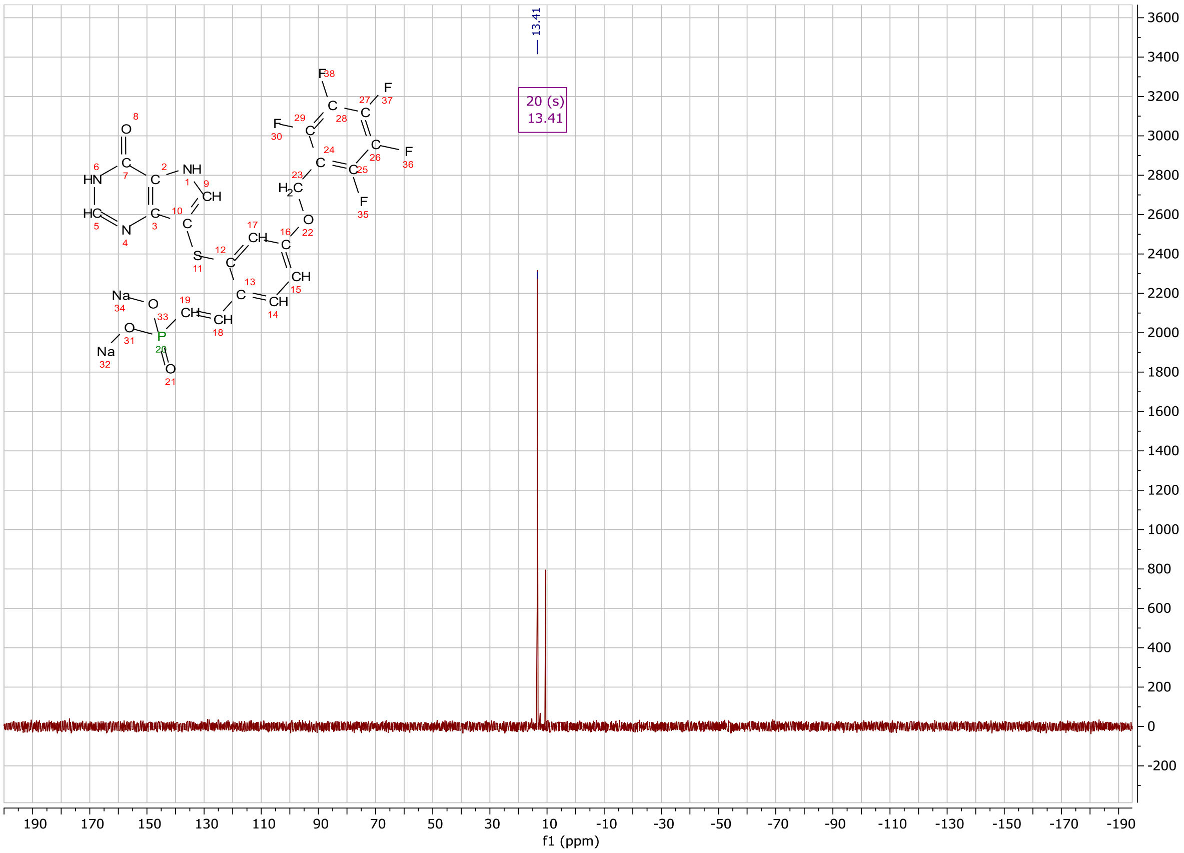
13.41
20 (s)
13.41
f1 (ppm)

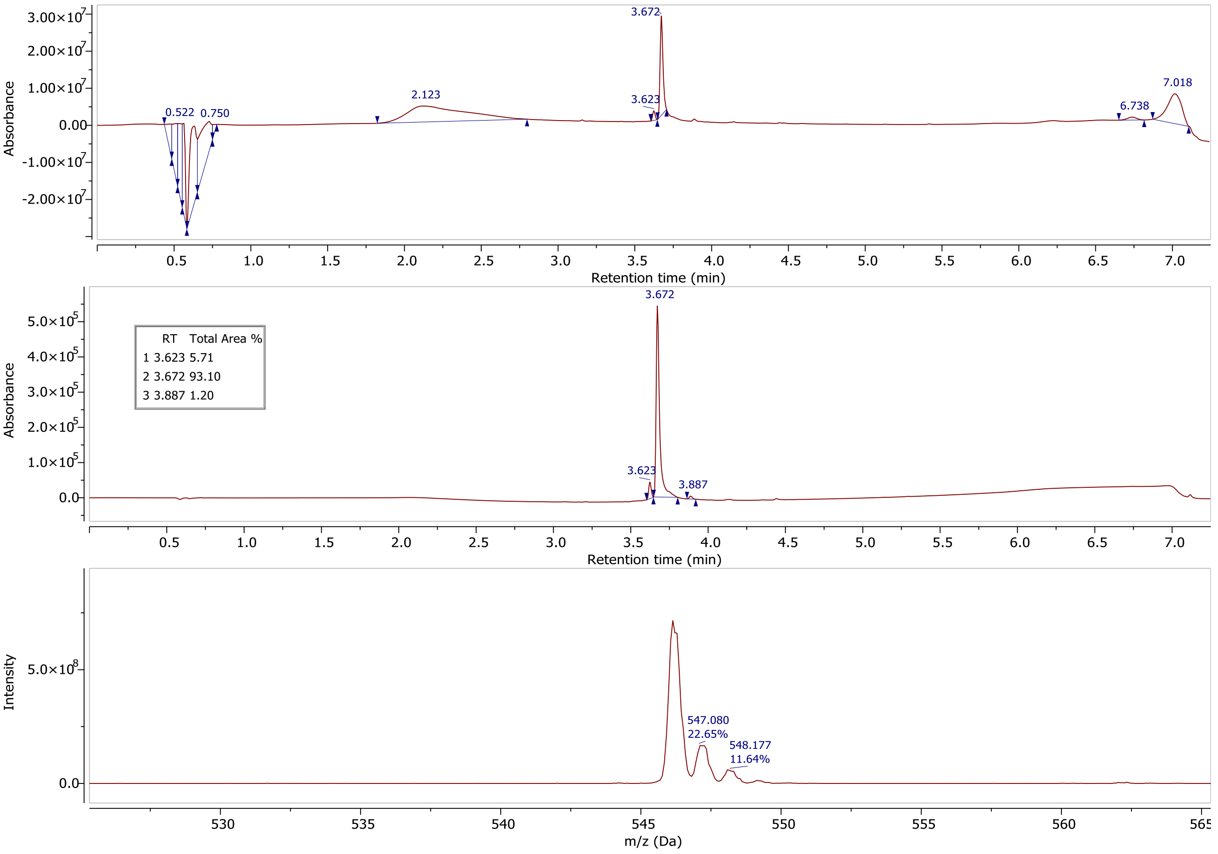
Absorbance
Retention time (min)
3.672
3.623
2.123
0.522
0.750
6.738
7.018
RT Total Area %
1 3.623 5.71
2 3.672 93.10
3 3.887 1.20
3.887
Intensity
547.080
22.65%
548.177
11.64%
m/z (Da)

3.2.65 Sodium (*E*)-7-((4-hydroxy-2-(2-(phosphonato)vinyl)phenyl)thio)-3,5-dihydro-4*H*-pyrrolo[3,2-*d*]pyrimidin-4-one (45f)

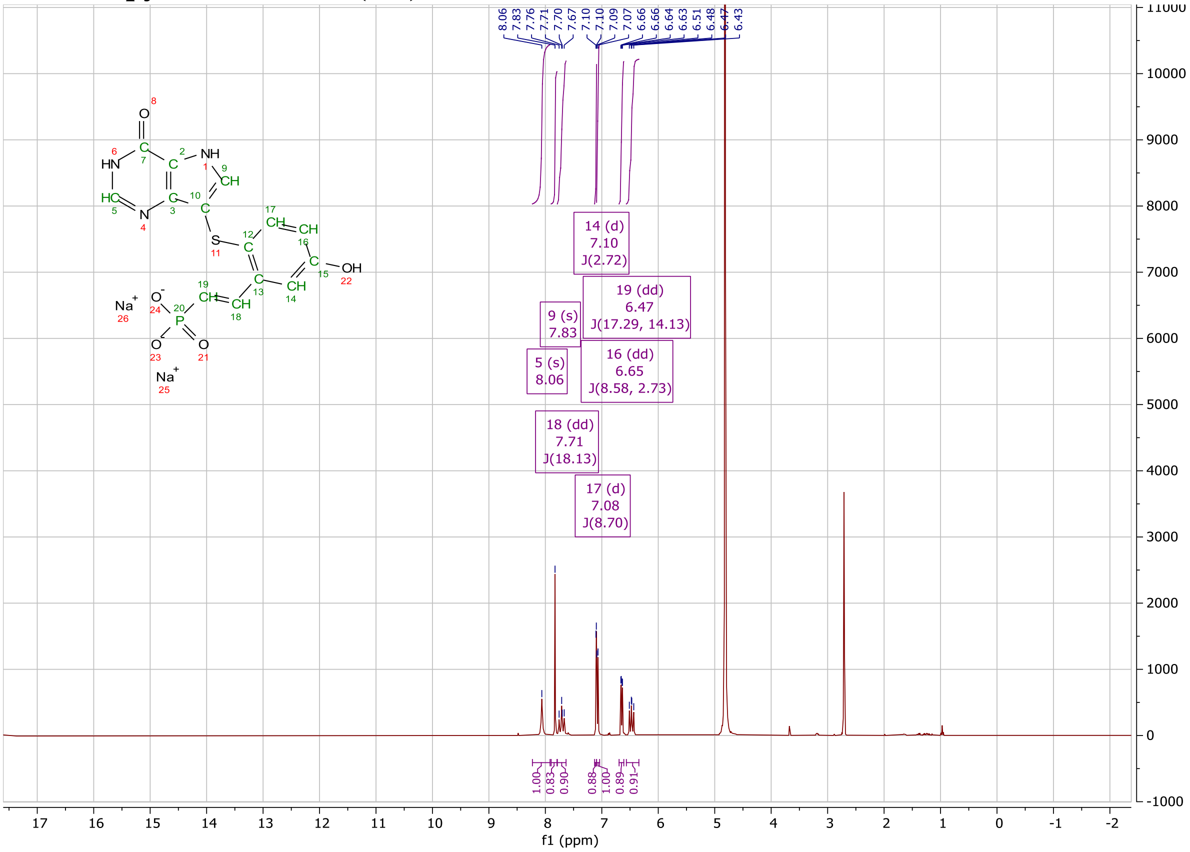

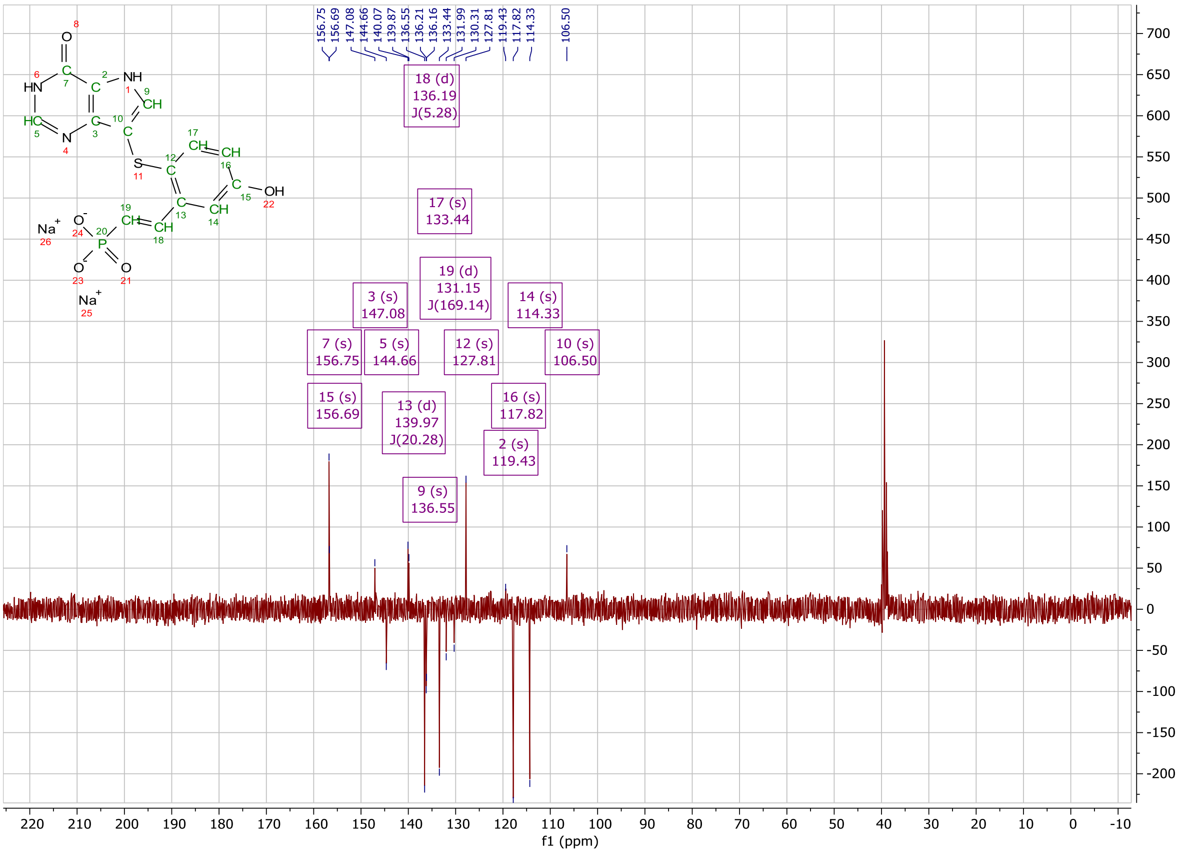

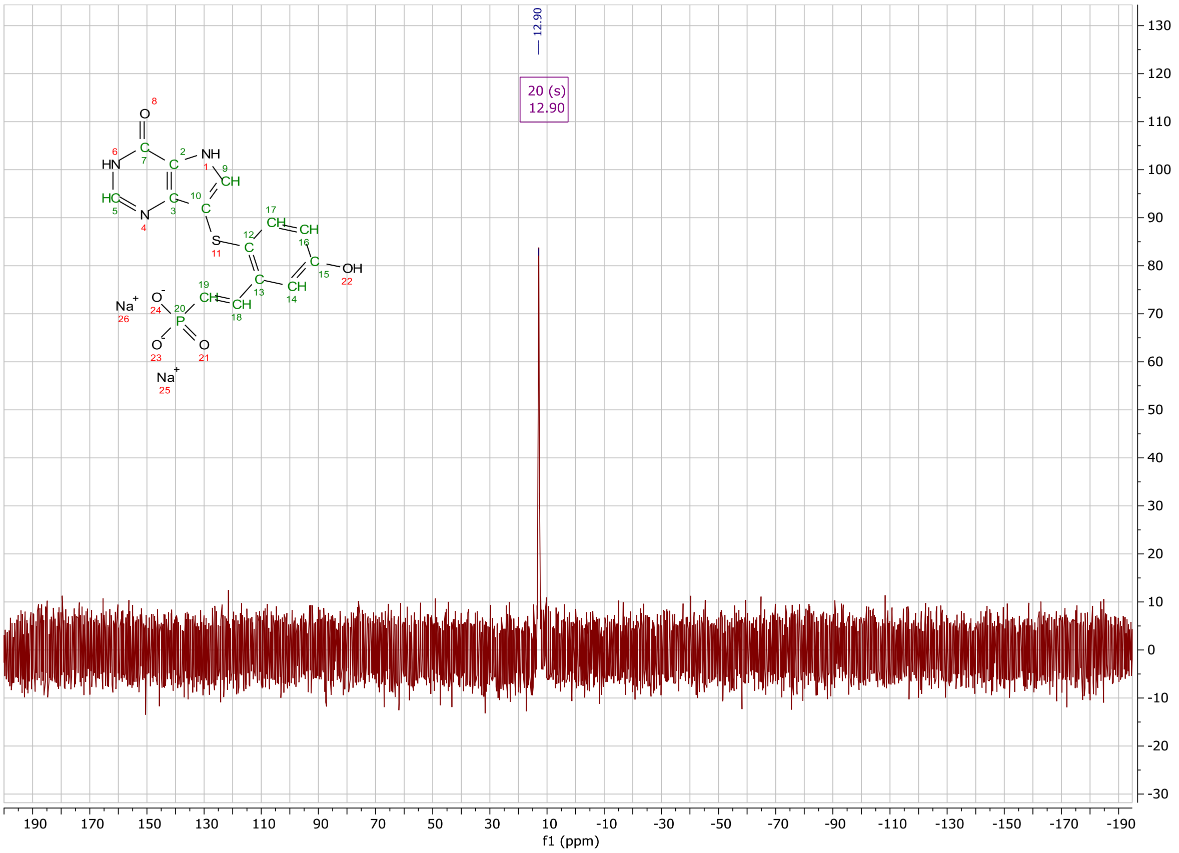
12.90
20 (s)
12.90
f1 (ppm)

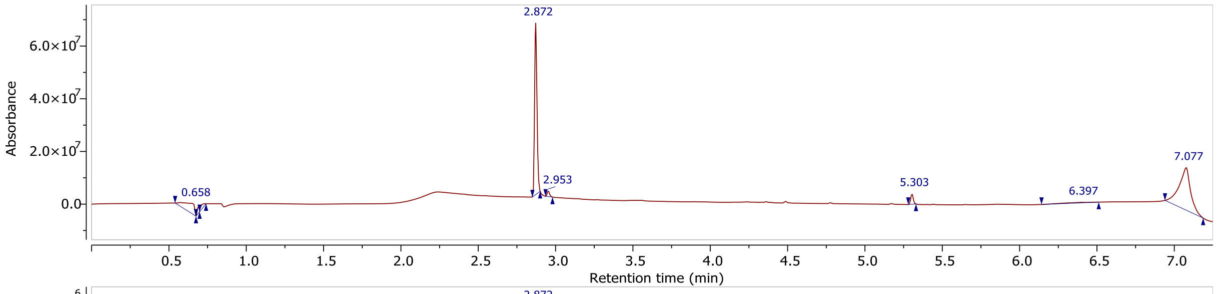
2.872
2.953
0.658
5.303
6.397
7.077
Absorbance
Retention time (min)

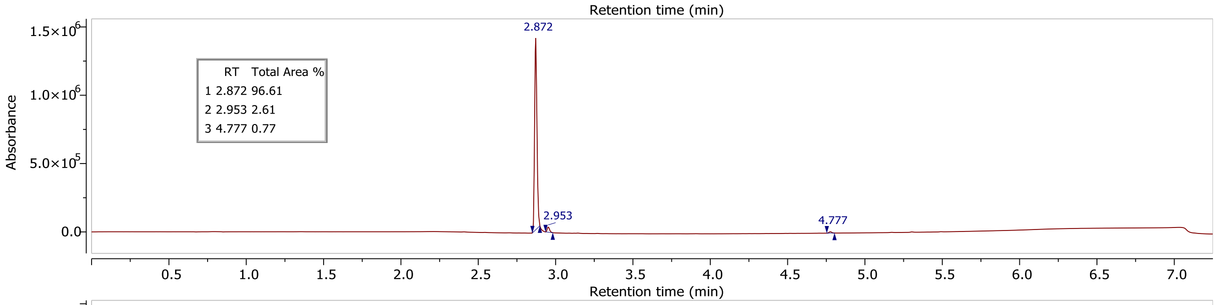
2.872
RT Total Area %
1 2.872 96.61
2 2.953 2.61
3 4.777 0.77
2.953
4.777
Absorbance
Retention time (min)

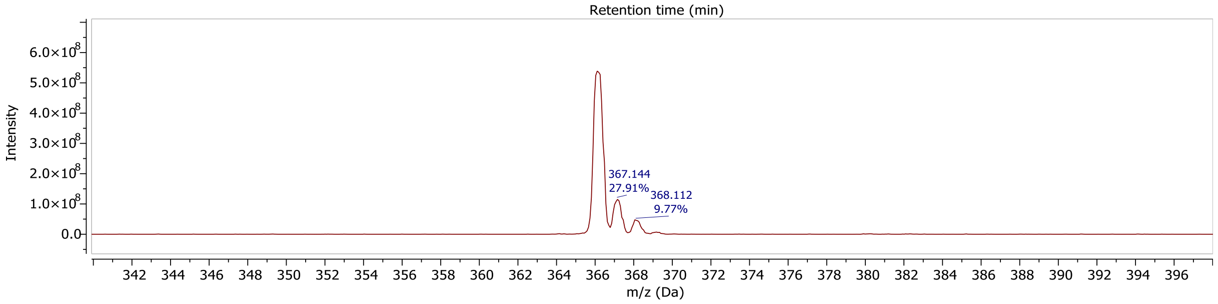
367.144
27.91%
368.112
9.77%
Intensity
m/z (Da)

3.2.66 Sodium (*E*)-7-((4-methoxy-2-(2-(phosphonato)vinyl)phenyl)thio)-3,5-dihydro-4*H*-pyrrolo[3,2-*d*]pyrimidin-4-one (45g)

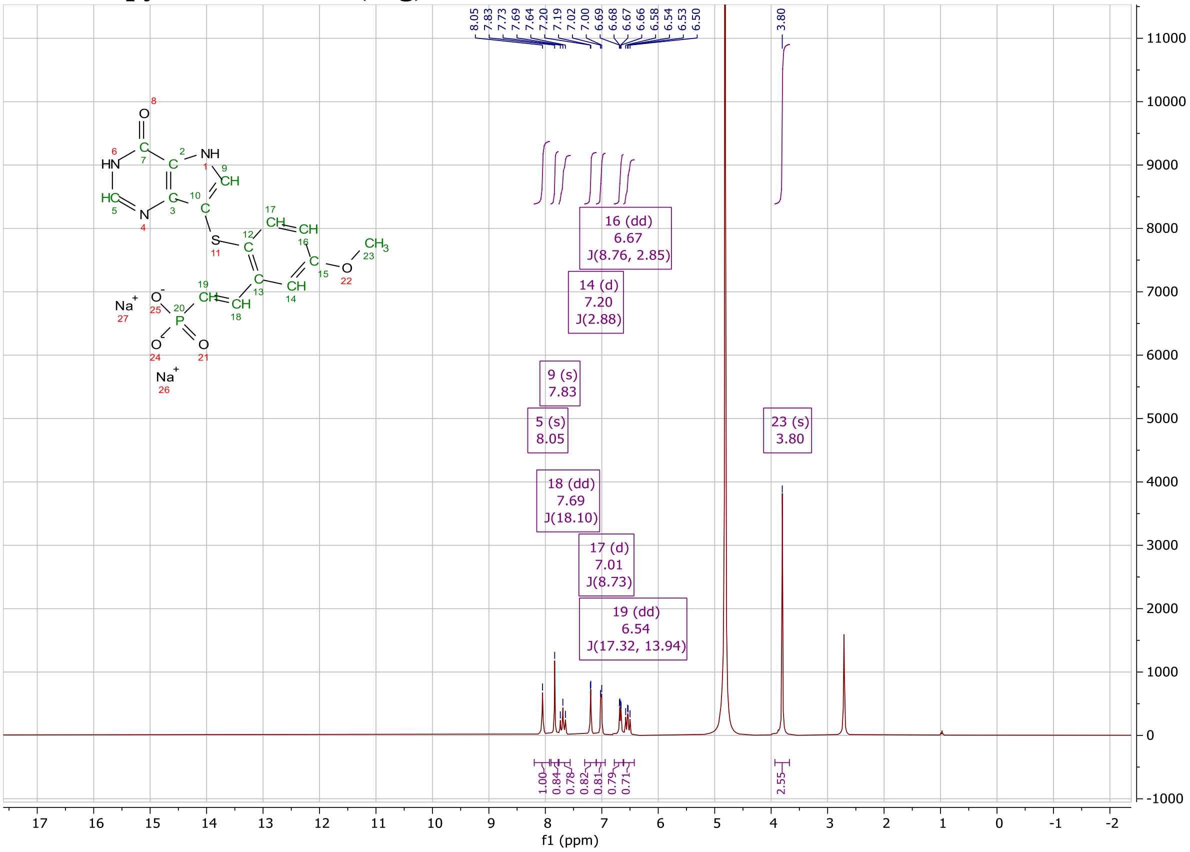

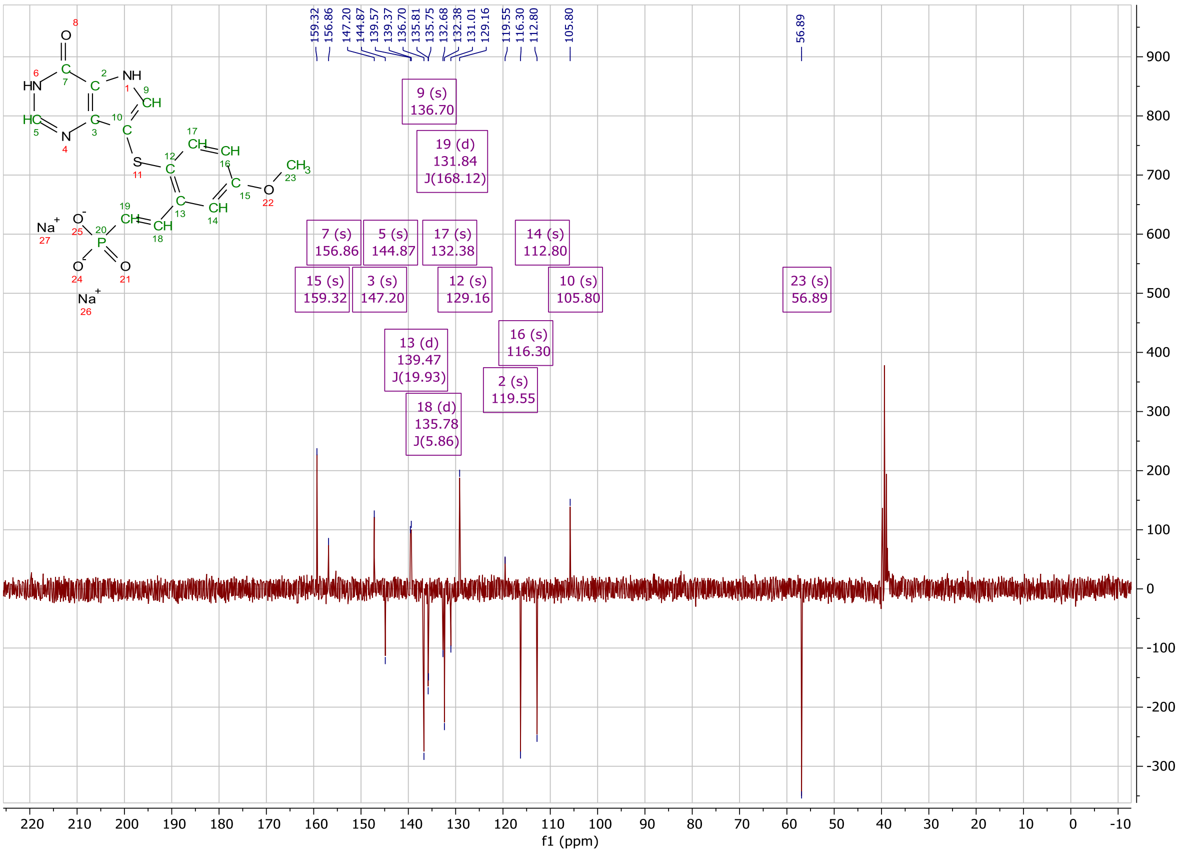

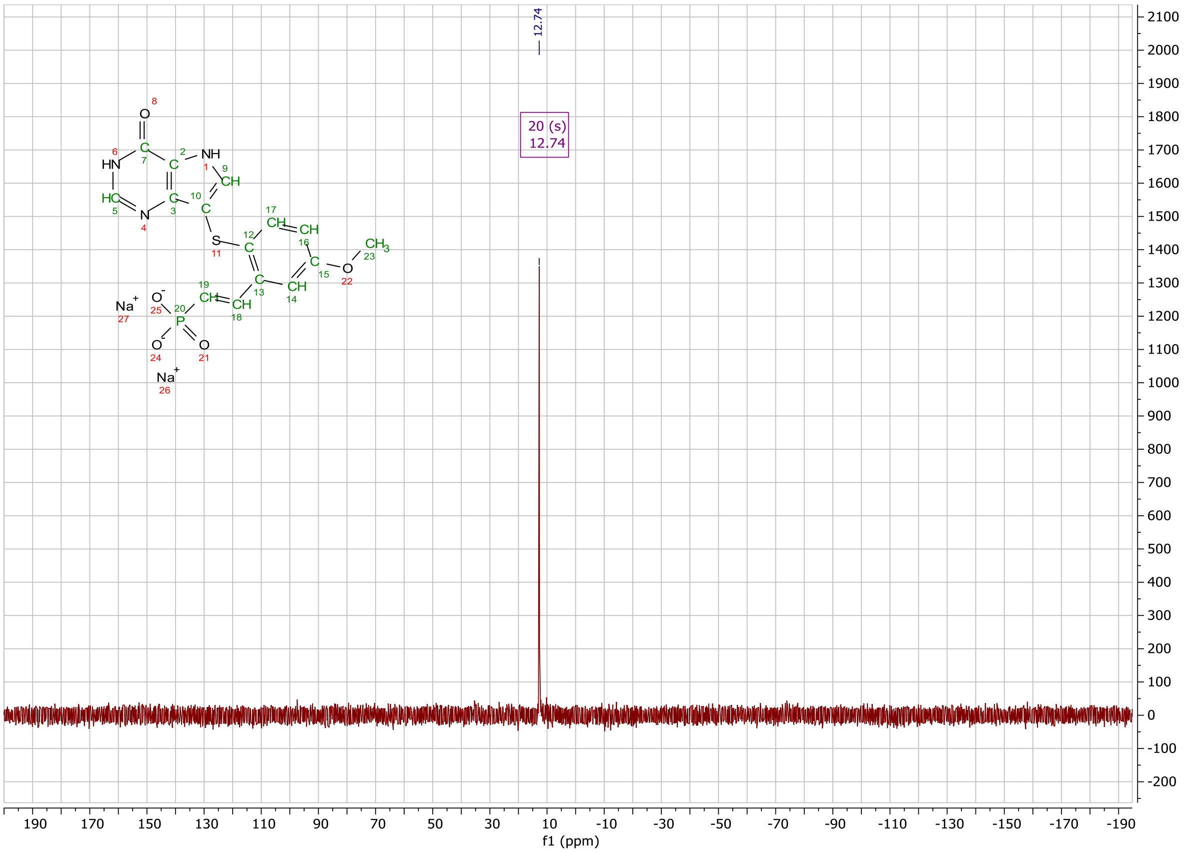

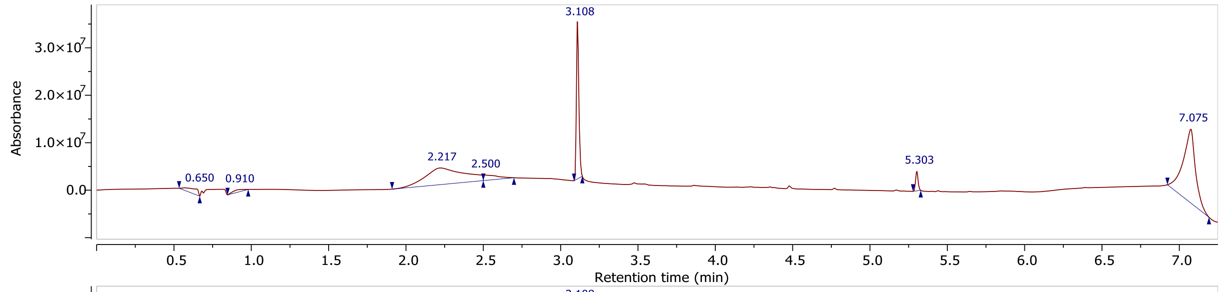

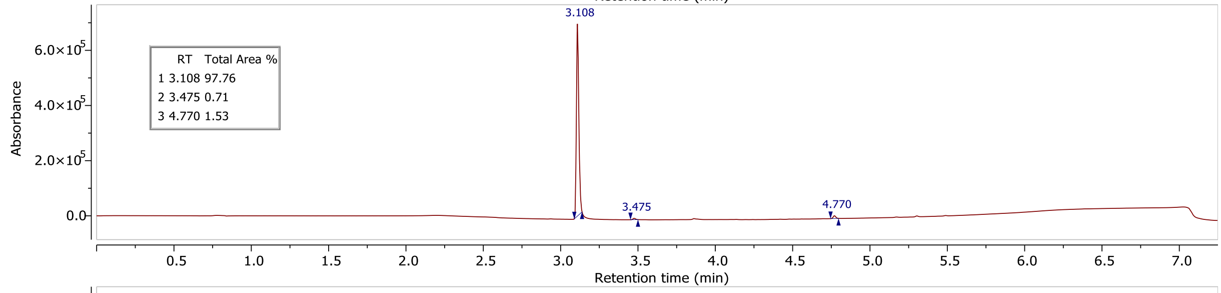

RT	Total Area %
1 3.108	97.76
2 3.475	0.71
3 4.770	1.53

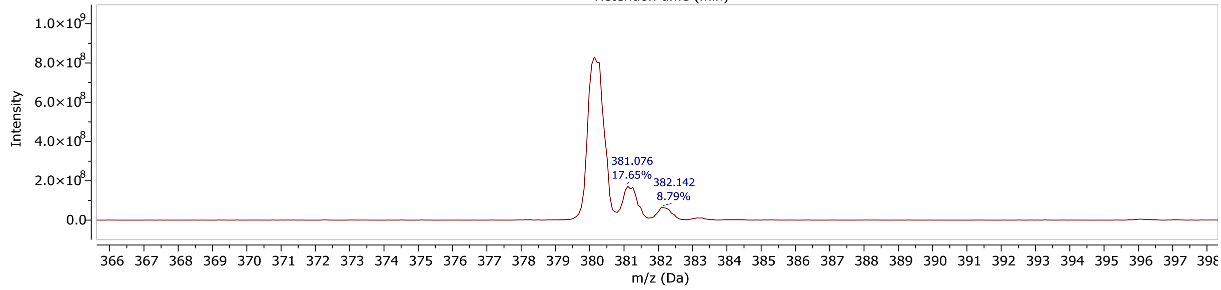

3.2.67 Sodium (*E*)-7-((4-isopropoxy-2-(2-(phosphonato)vinyl)phenyl)thio)-3,5-dihydro-4*H*-pyrrolo[3,2-*d*]pyrimidin-4-one (45h)

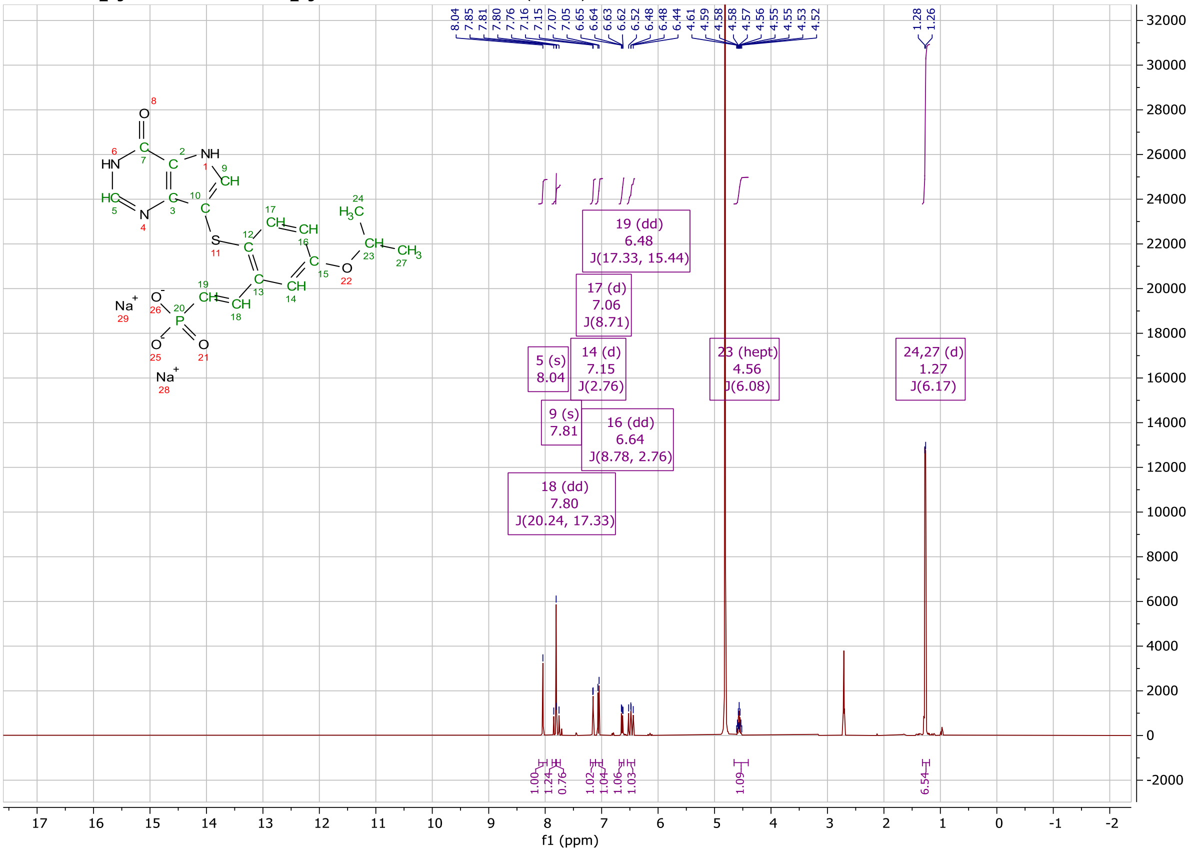

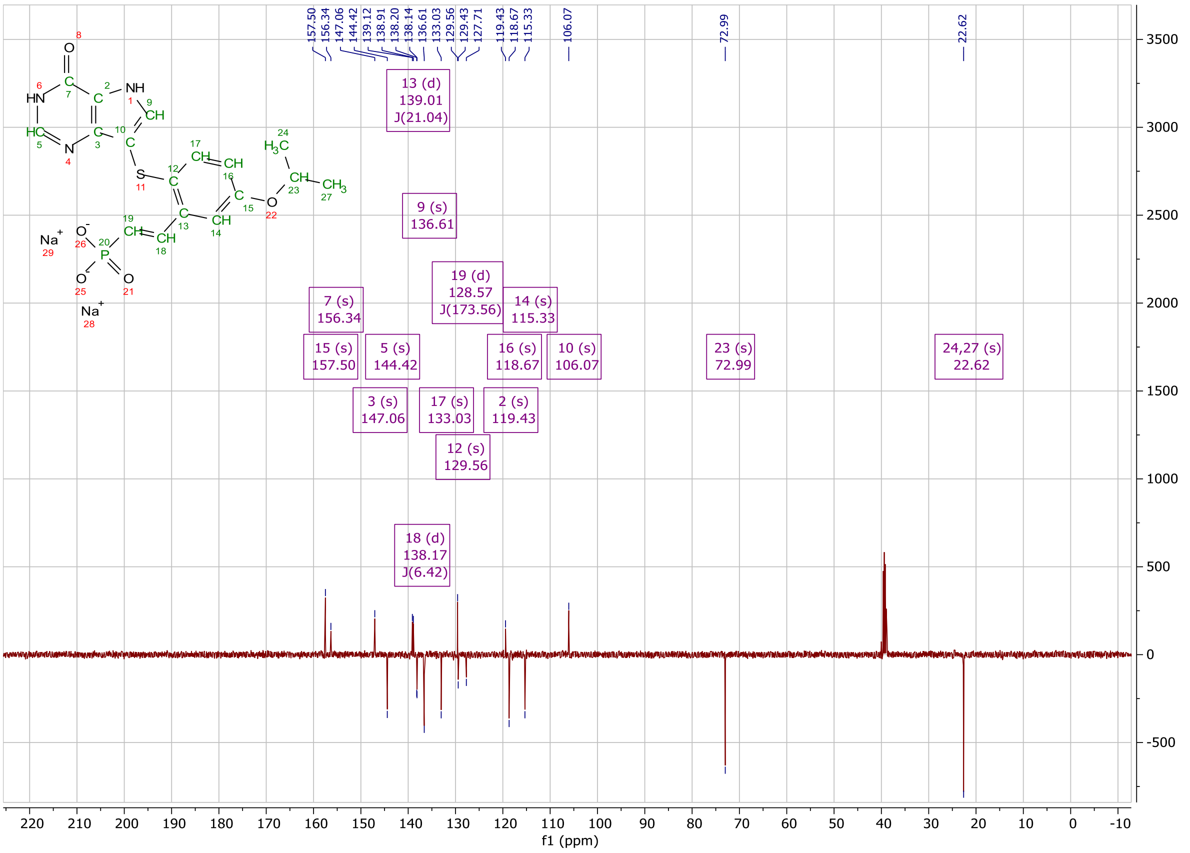

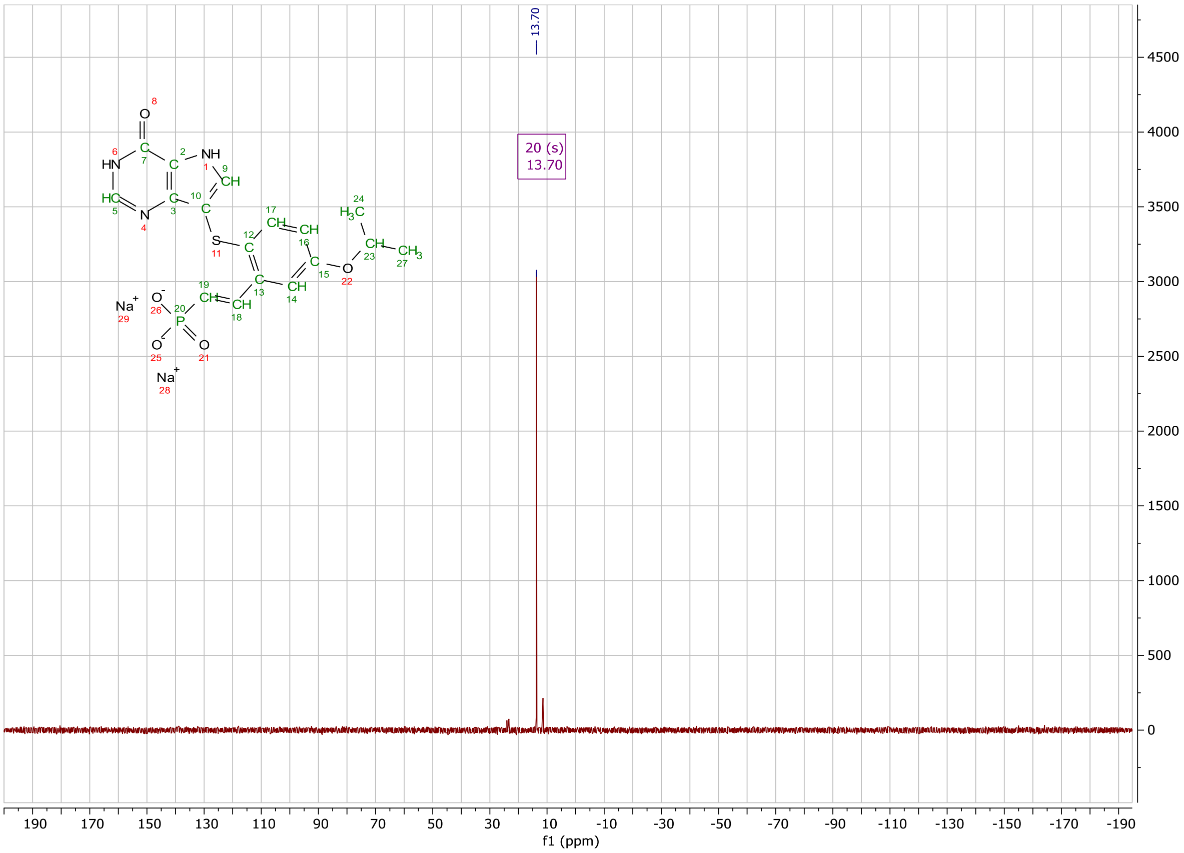
13.70
20 (s)
13.70
f1 (ppm)

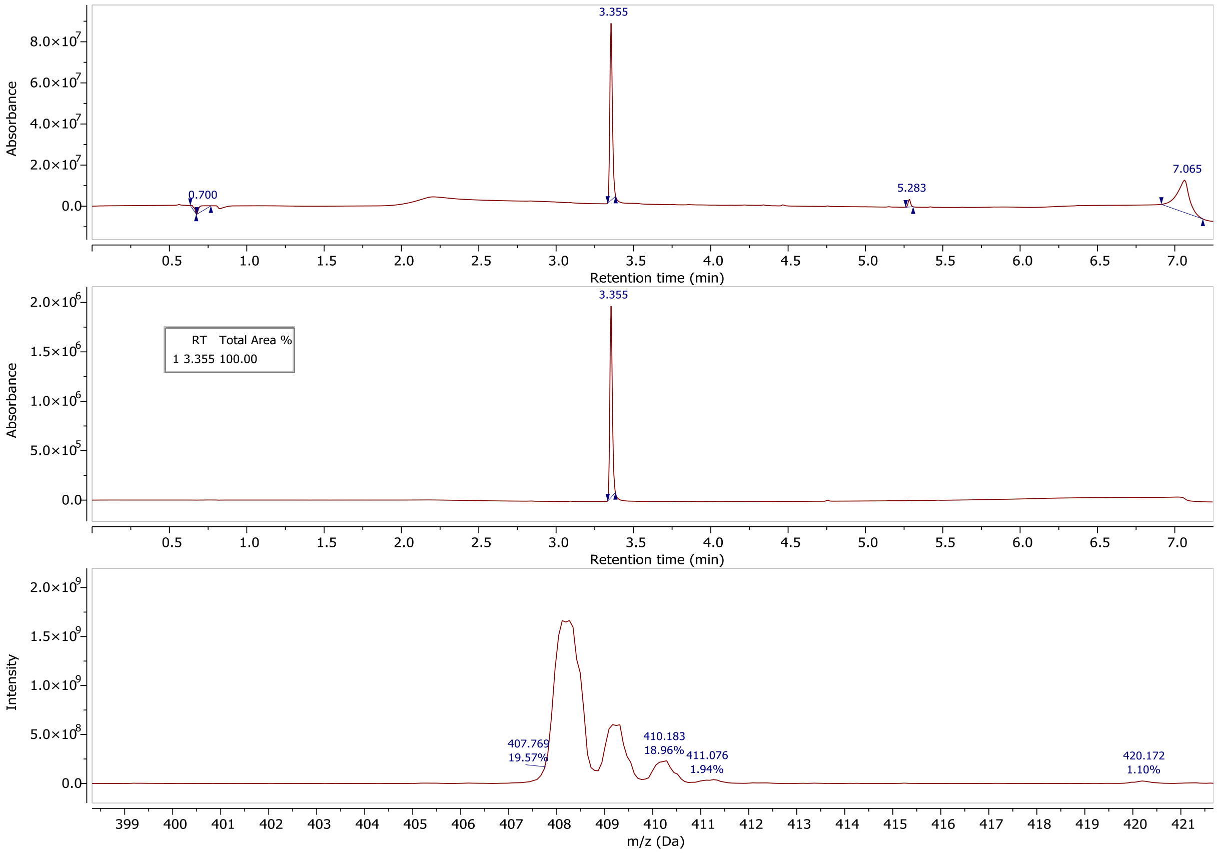
3.355
0.700
5.283
7.065
Absorbance
Retention time (min)
RT Total Area %
1 3.355 100.00
Intensity
407.769
19.57%
410.183
18.96%
411.076
1.94%
420.172
1.10%
m/z (Da)

3.2.68 Sodium (*E*)-7-((4-(2,3,4,5,6-pentafluorofluorophenyl)oxy-2-(2-(phosphonato)vinyl)phenyl)thio)-3,5-dihydro-4*H*-pyrrolo[3,2-*d*]pyrimidin-4-one (45i)

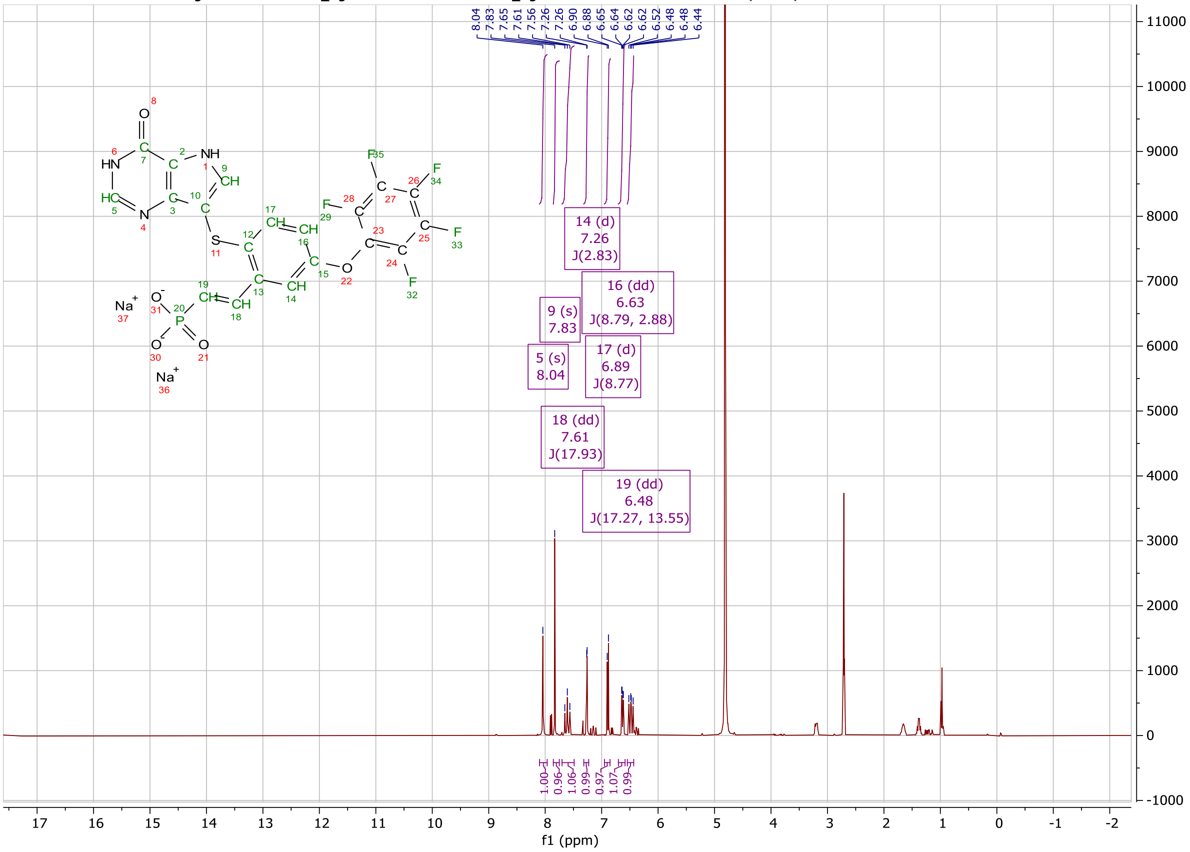

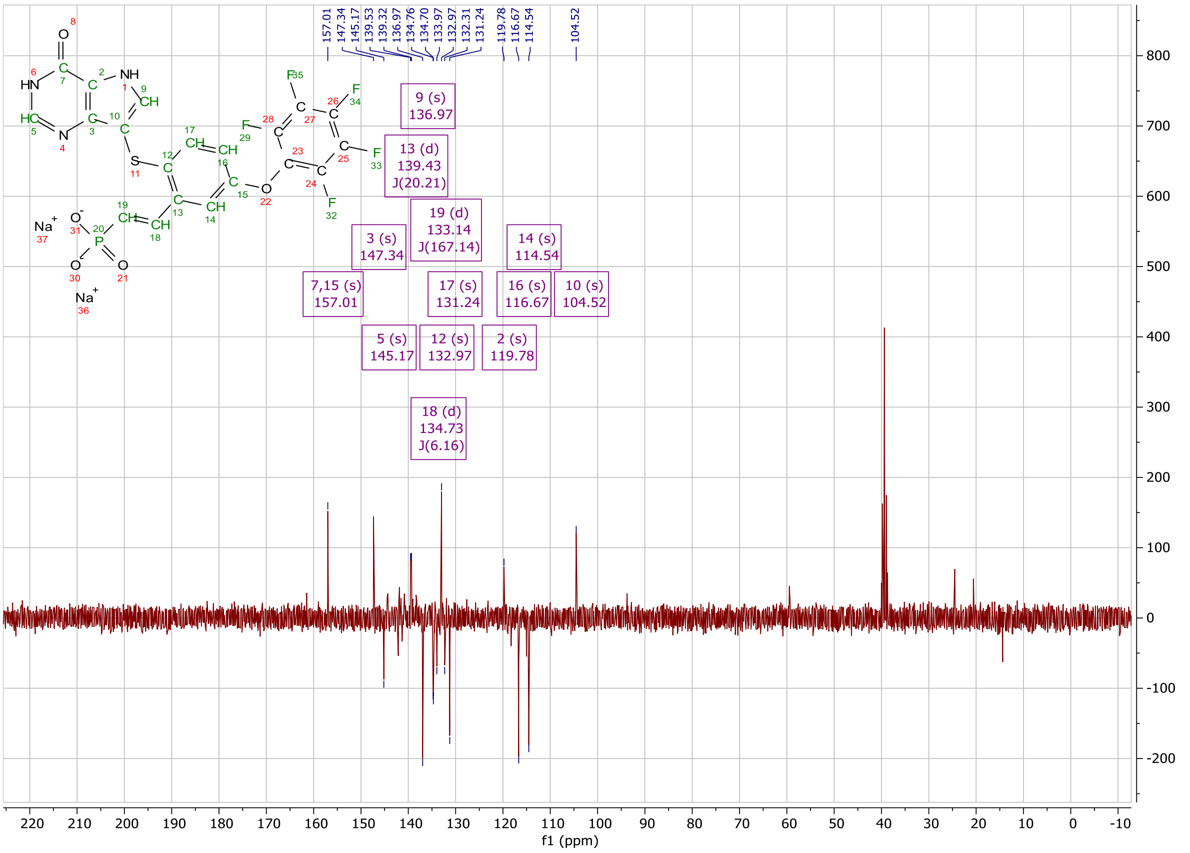

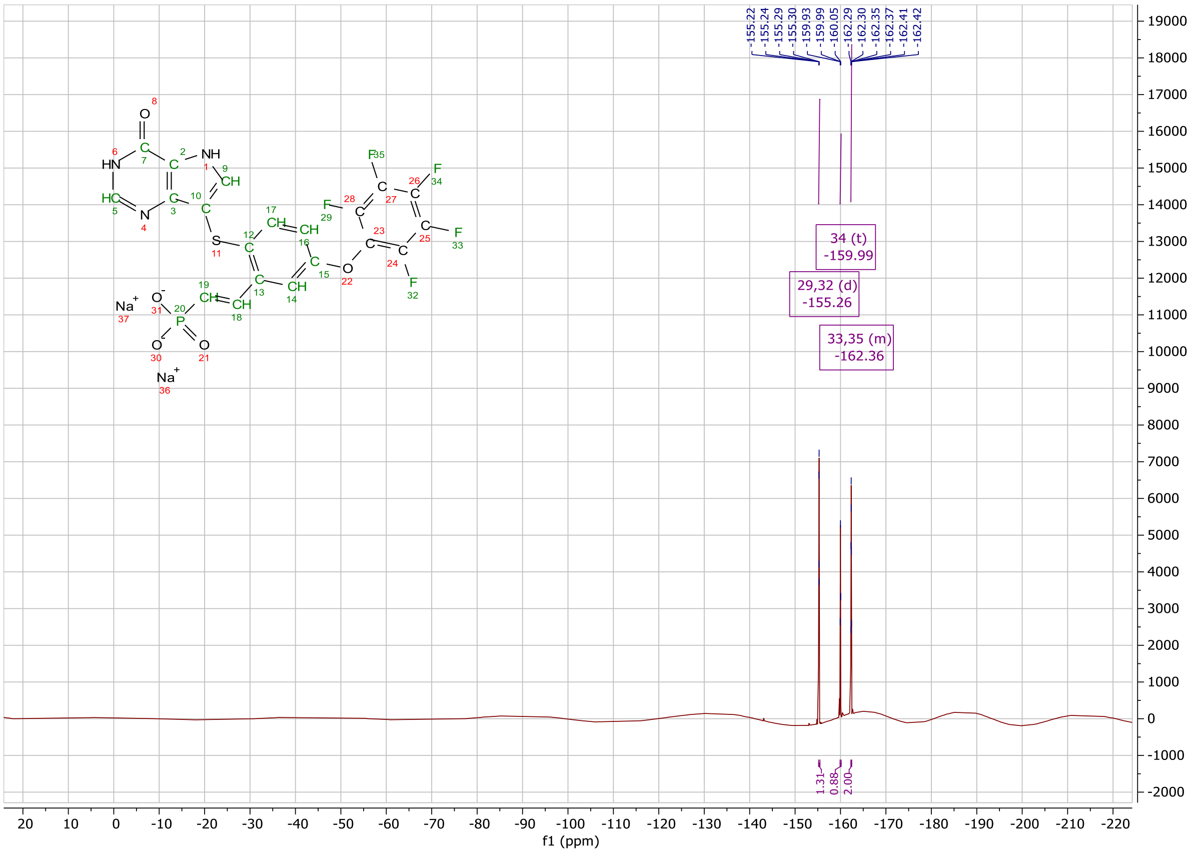
-155.22
-155.24
-155.29
-155.30
-159.93
-159.99
-160.05
-162.29
-162.30
-162.35
-162.37
-162.41
-162.42
34 (t)
-159.99
29,32 (d)
-155.26
33,35 (m)
-162.36
1.31
0.88
2.00
f1 (ppm)

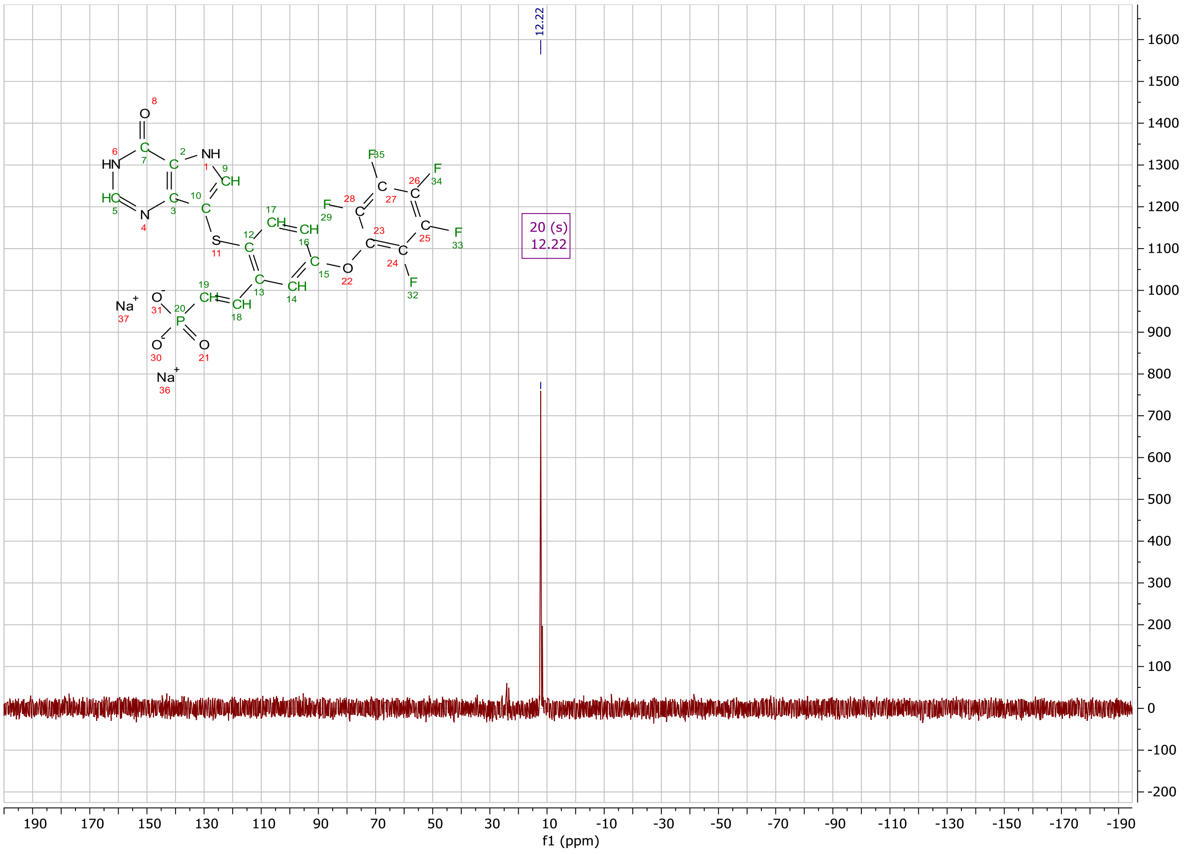
12.22
20 (s)
12.22
f1 (ppm)

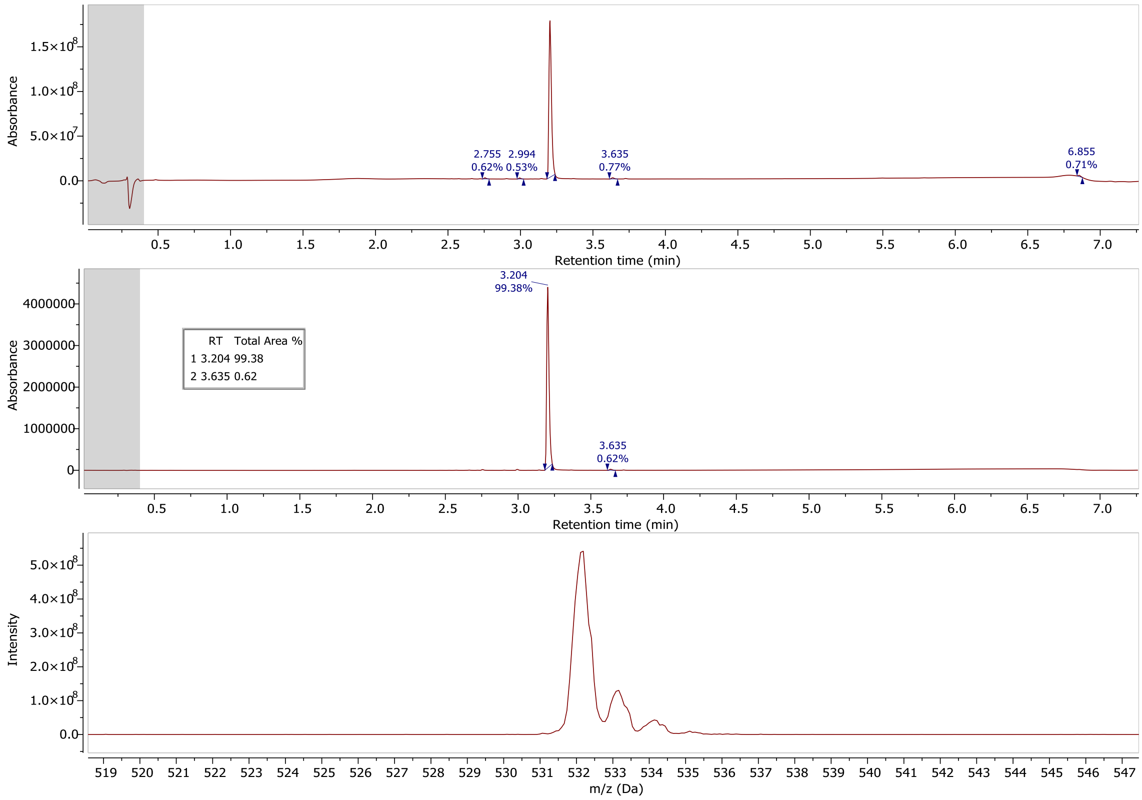

3.2.69 Sodium (*E*)-7-((4-(2,3,4,5,6-pentafluorofluorophenyl)methoxy-2-(2-(phosphonato)vinyl)phenyl)thio)-3,5-dihydro-4*H*-pyrrolo[3,2-*d*]pyrimidin-4-one (45j)

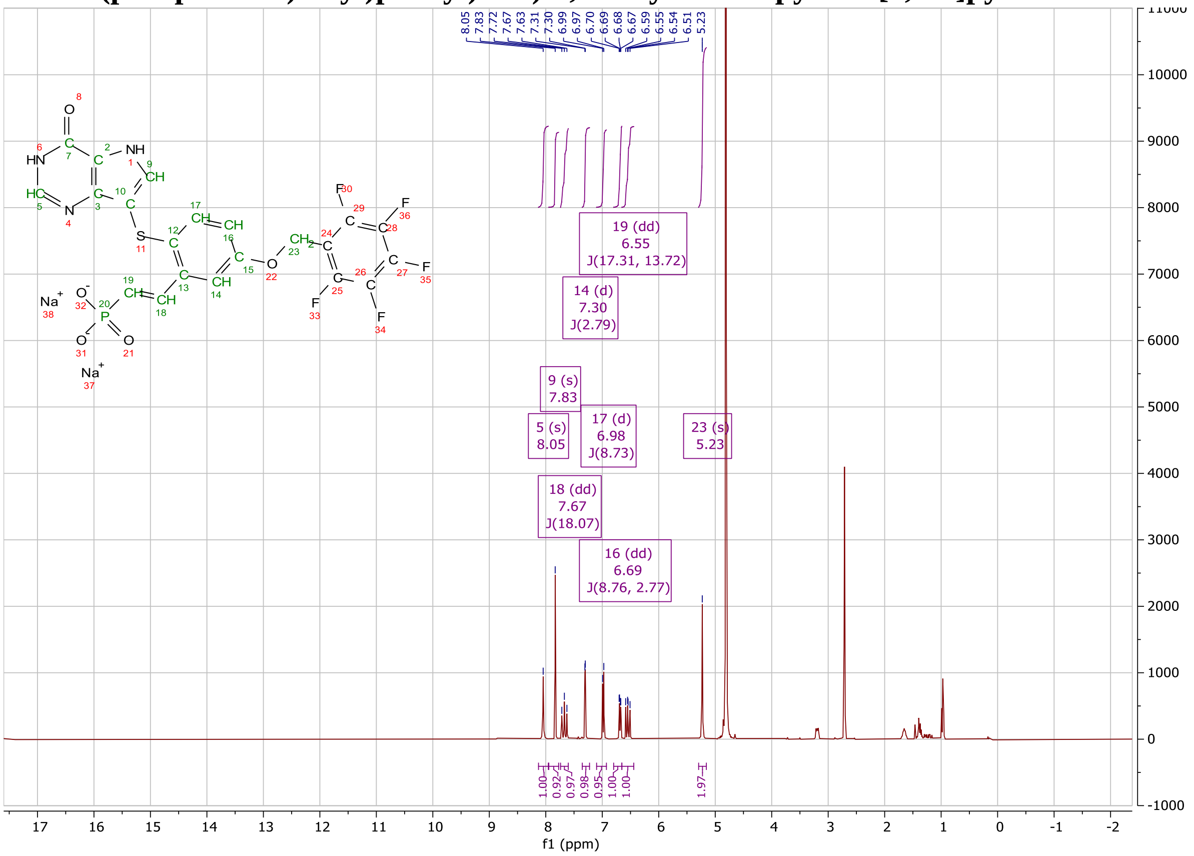

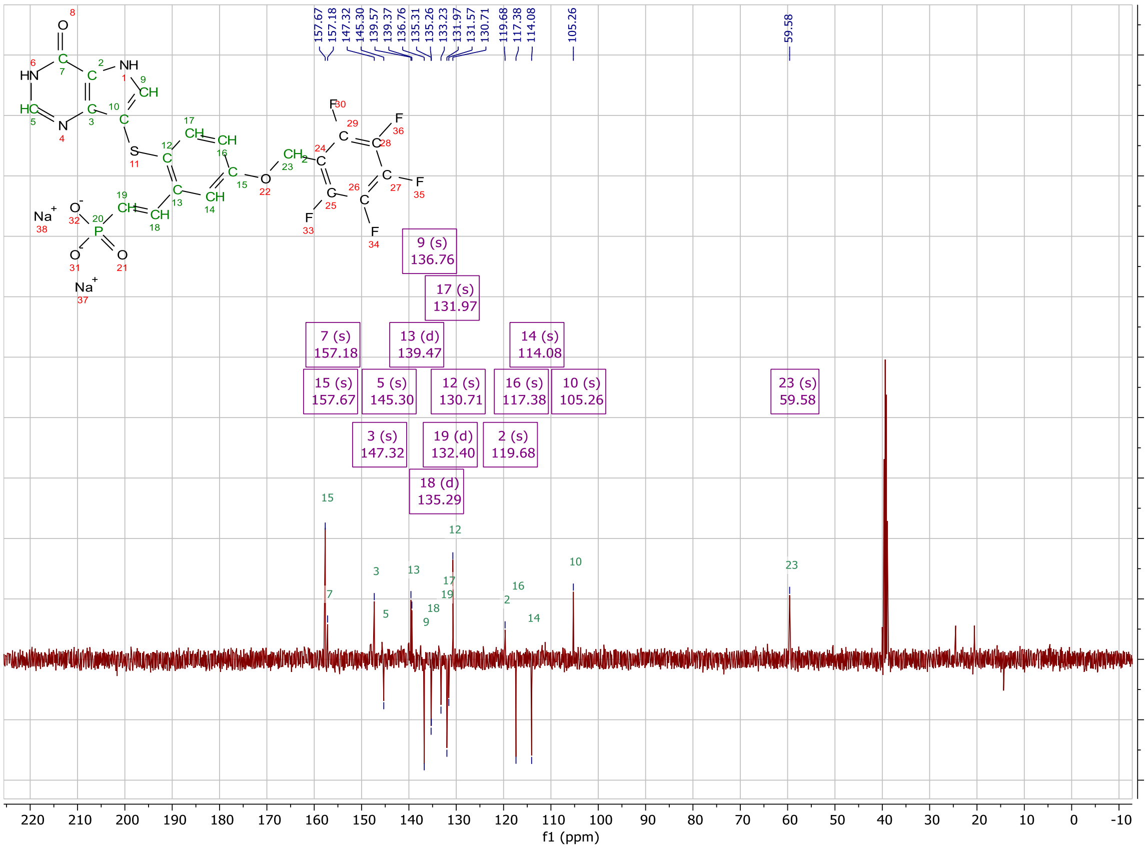
157.67
157.18
147.32
145.30
139.57
139.37
136.76
135.31
135.26
133.23
131.97
131.57
130.71
119.68
117.38
114.08
105.26
59.58
9 (s) 136.76
17 (s) 131.97
7 (s) 157.18
13 (d) 139.47
14 (s) 114.08
15 (s) 157.67
5 (s) 145.30
12 (s) 130.71
16 (s) 117.38
10 (s) 105.26
23 (s) 59.58
3 (s) 147.32
19 (d) 132.40
2 (s) 119.68
18 (d) 135.29
f1 (ppm)

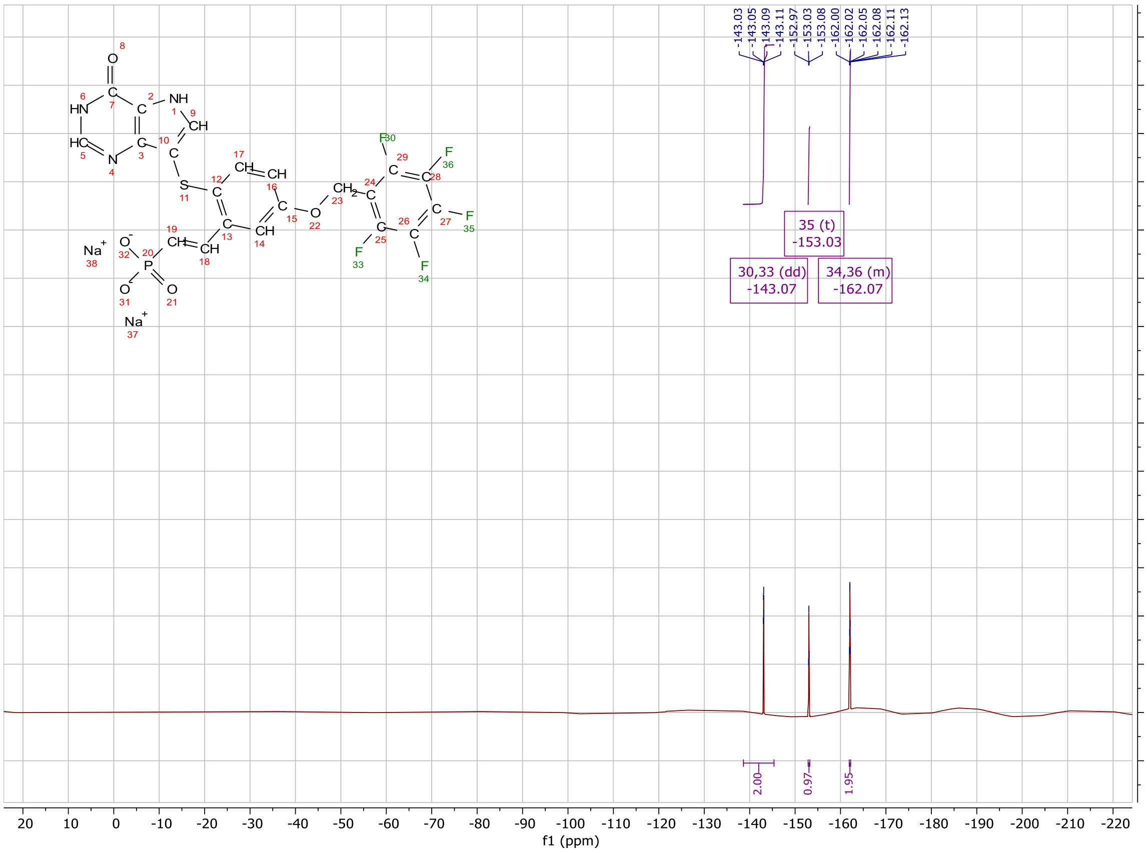
-143.03
-143.05
-143.09
-143.11
-152.97
-153.03
-153.08
-162.00
-162.02
-162.05
-162.08
-162.11
-162.13
35 (t) -153.03
30,33 (dd) -143.07
34,36 (m) -162.07
2.00
0.97
1.95
f1 (ppm)

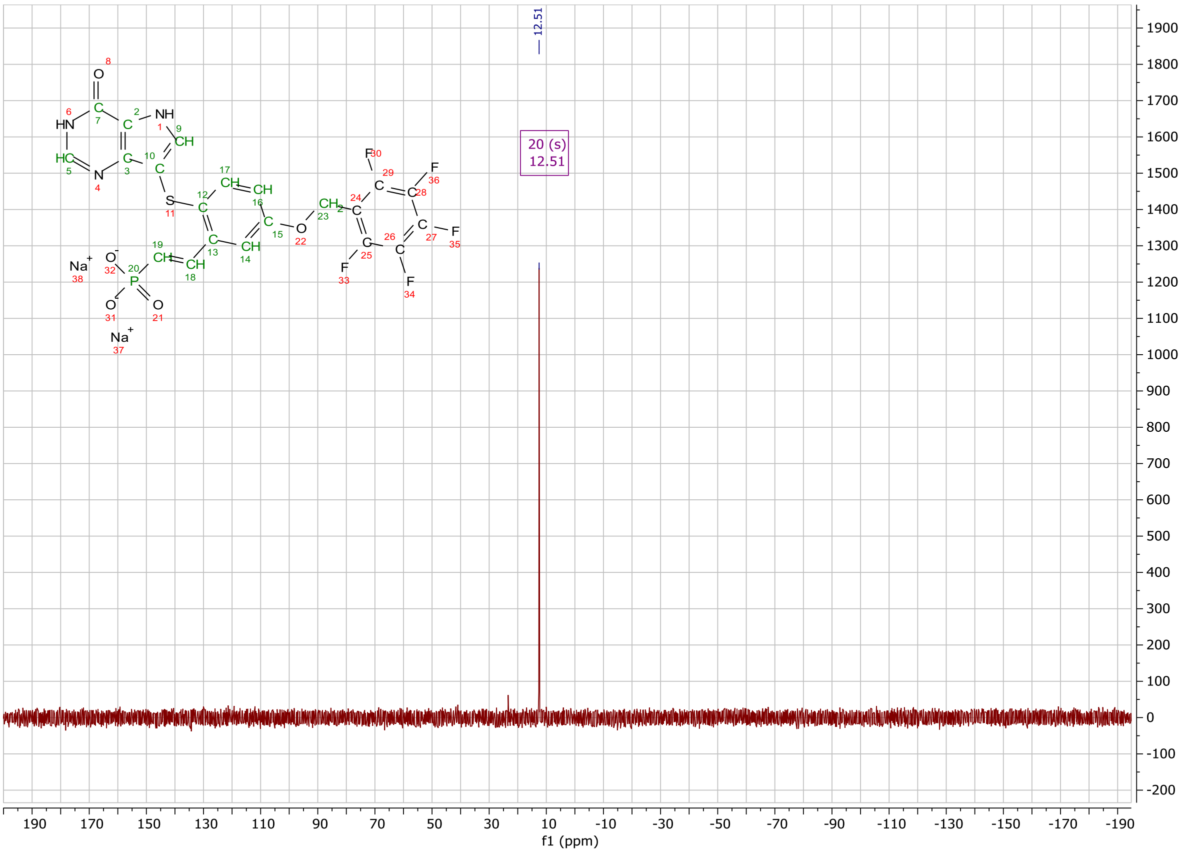
12.51
20 (s)
12.51
f1 (ppm)

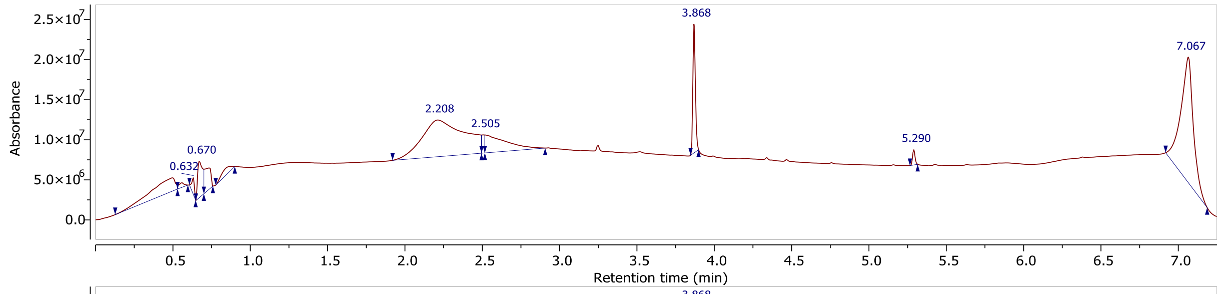
3.868
7.067
2.208
2.505
0.670
0.632
5.290
Absorbance
Retention time (min)

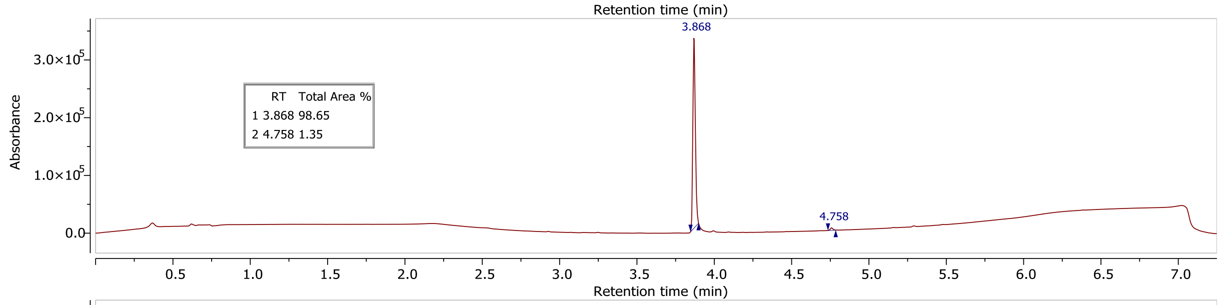
3.868
RT Total Area %
1 3.868 98.65
2 4.758 1.35
4.758
Absorbance
Retention time (min)

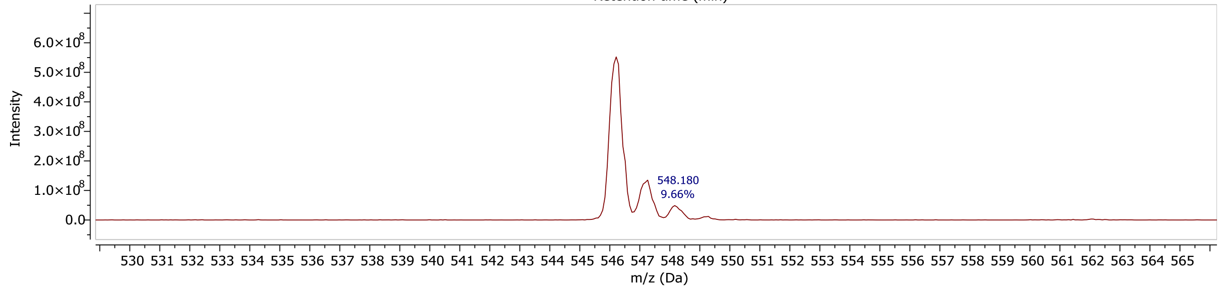
548.180
9.66%
Intensity
m/z (Da)

3.2.70 Sodium (*E*)-7-((3-hydroxy-2-(2-(phosphonato)vinyl)phenyl)thio)-3,5-dihydro-4*H*-pyrrolo[3,2-*d*]pyrimidin-4-one (45k)

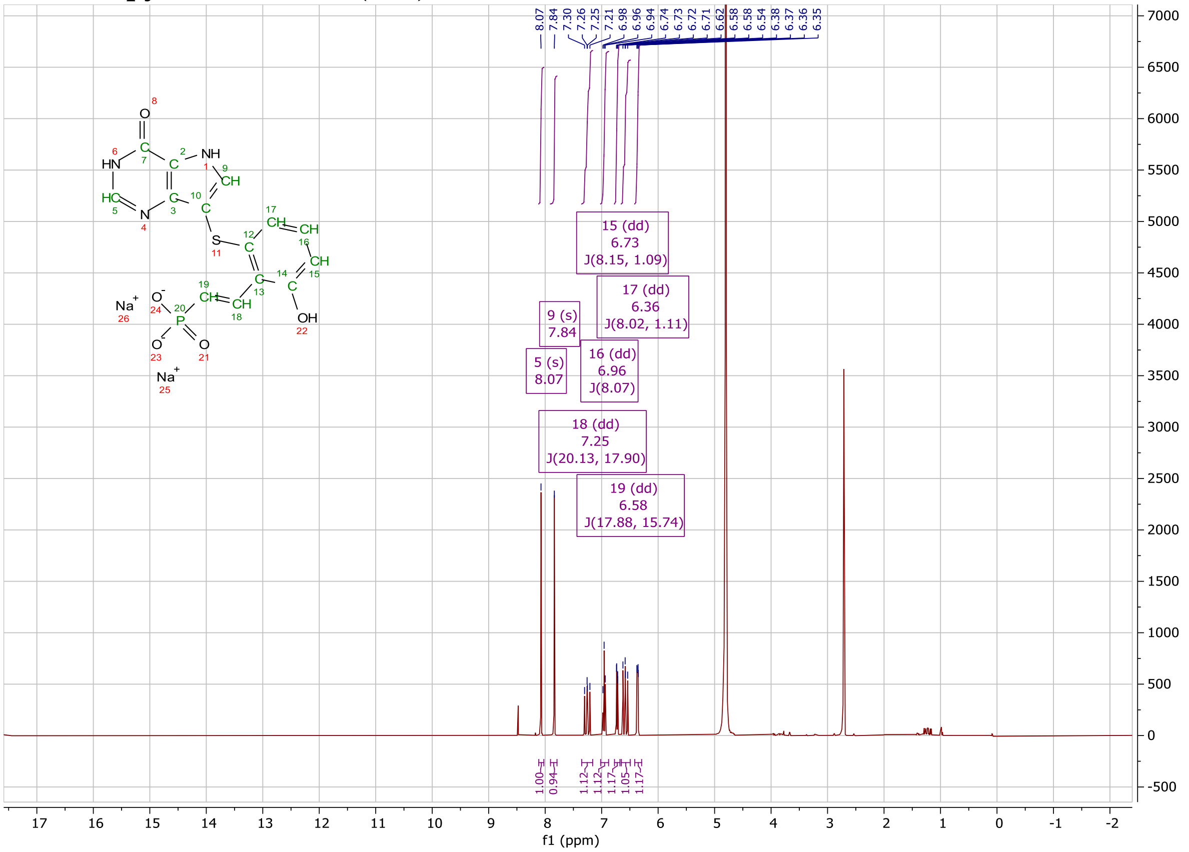

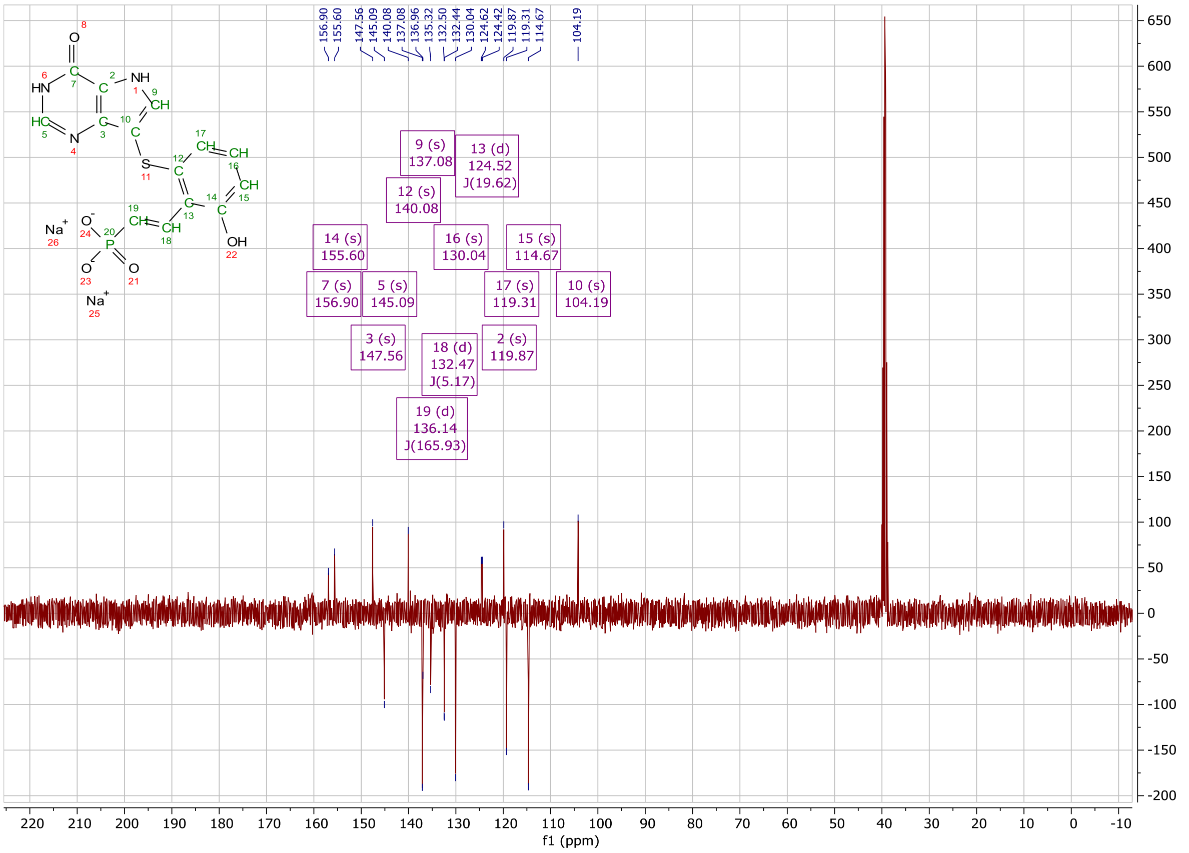

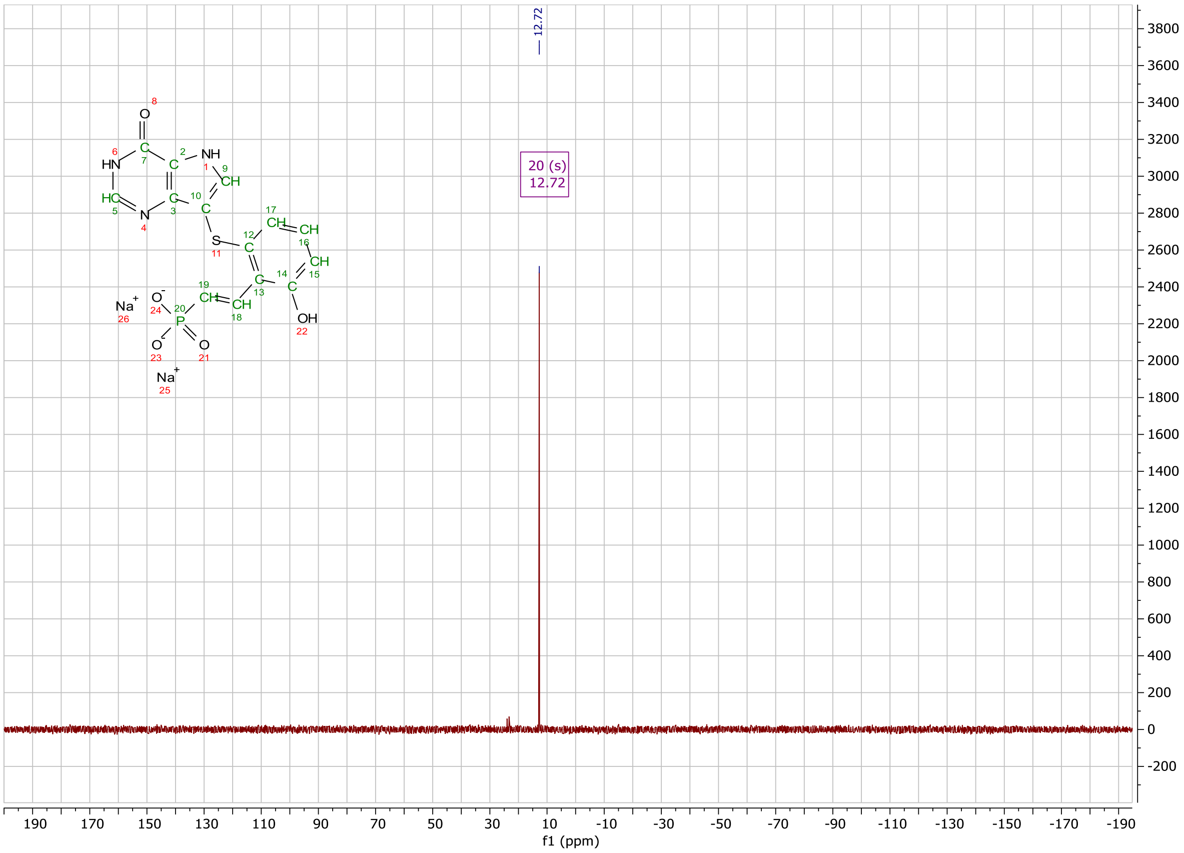
12.72
20 (s)
12.72
f1 (ppm)

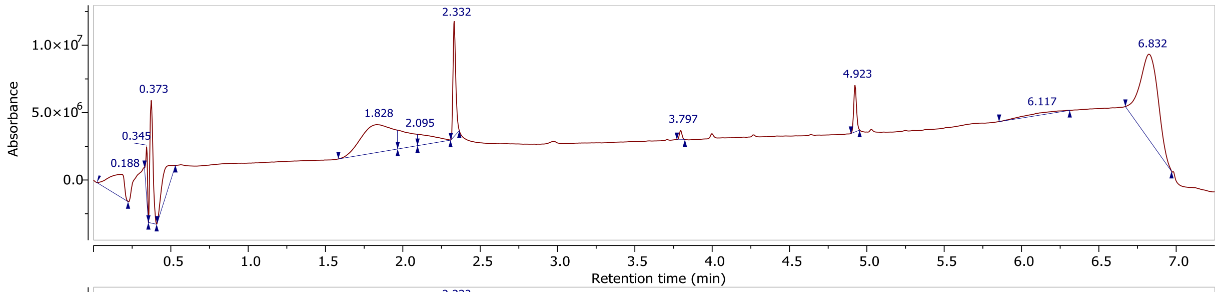
Absorbance
Retention time (min)
0.188
0.345
0.373
1.828
2.095
2.332
3.797
4.923
6.117
6.832

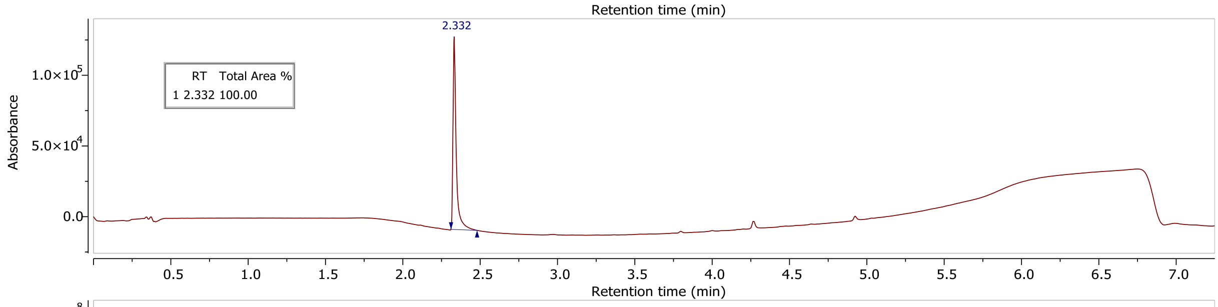
Absorbance
Retention time (min)
2.332
RT Total Area %
1 2.332 100.00

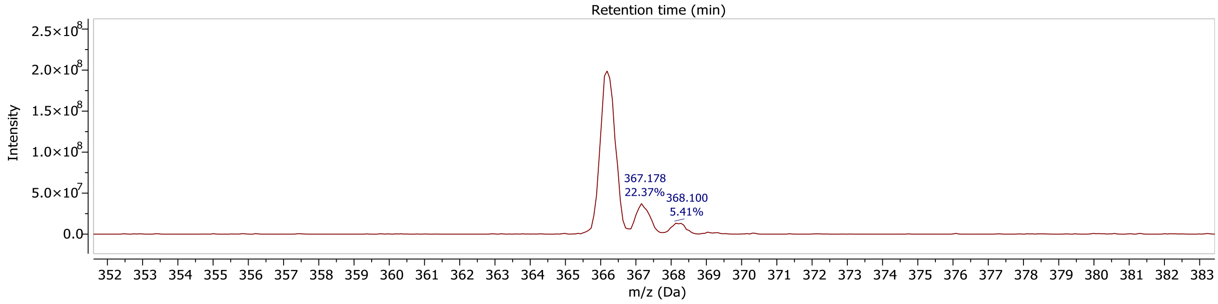
Intensity
m/z (Da)
367.178
22.37%
368.100
5.41%

3.2.71 Sodium (*E*)-7-((3-methoxy-2-(2-(phosphonato)vinyl)phenyl)thio)-3,5-dihydro-4*H*-pyrrolo[3,2-*d*]pyrimidin-4-one (45l)

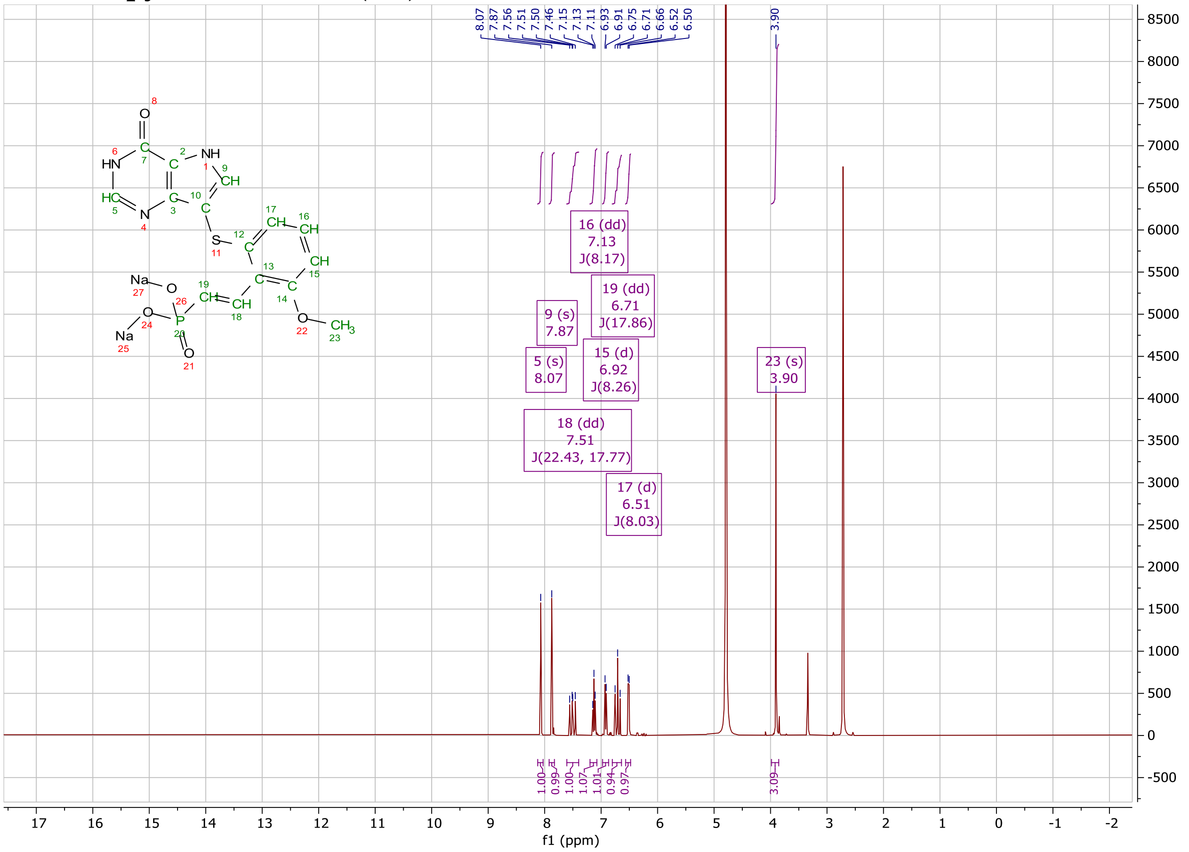

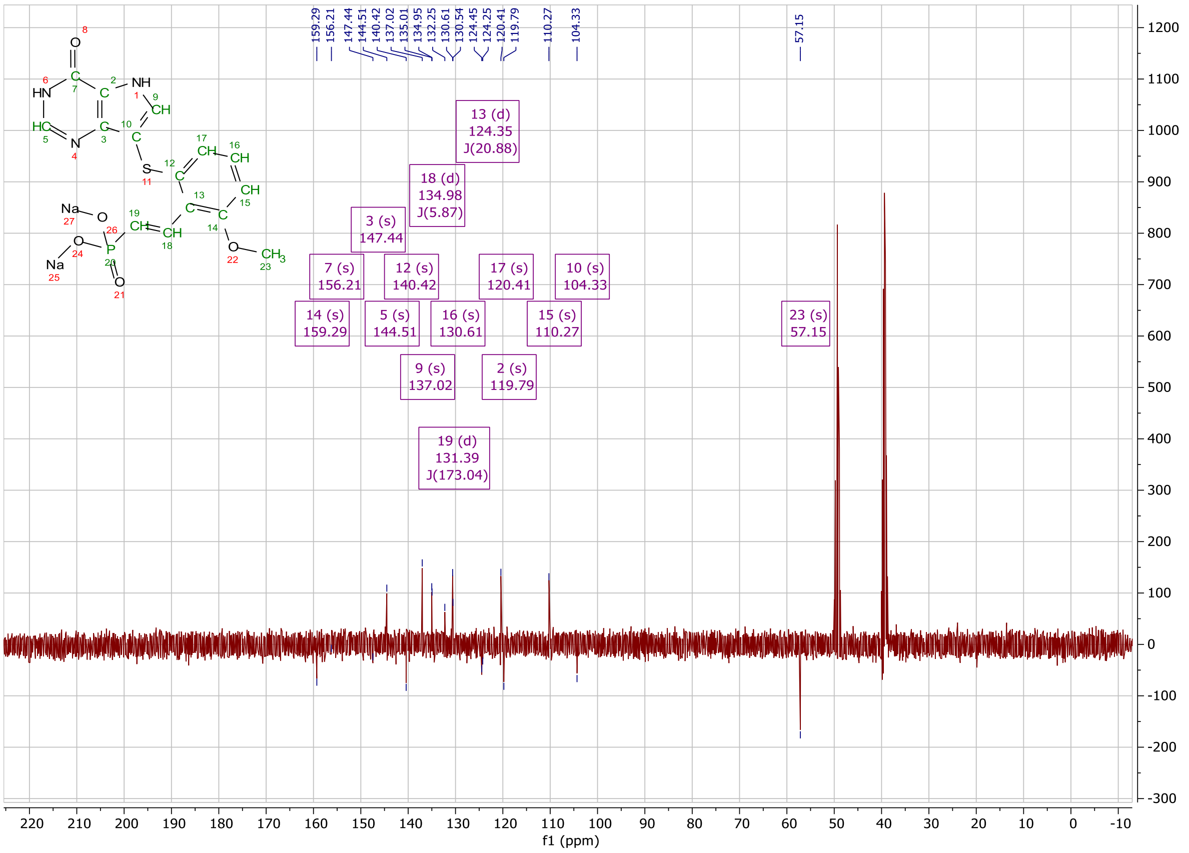

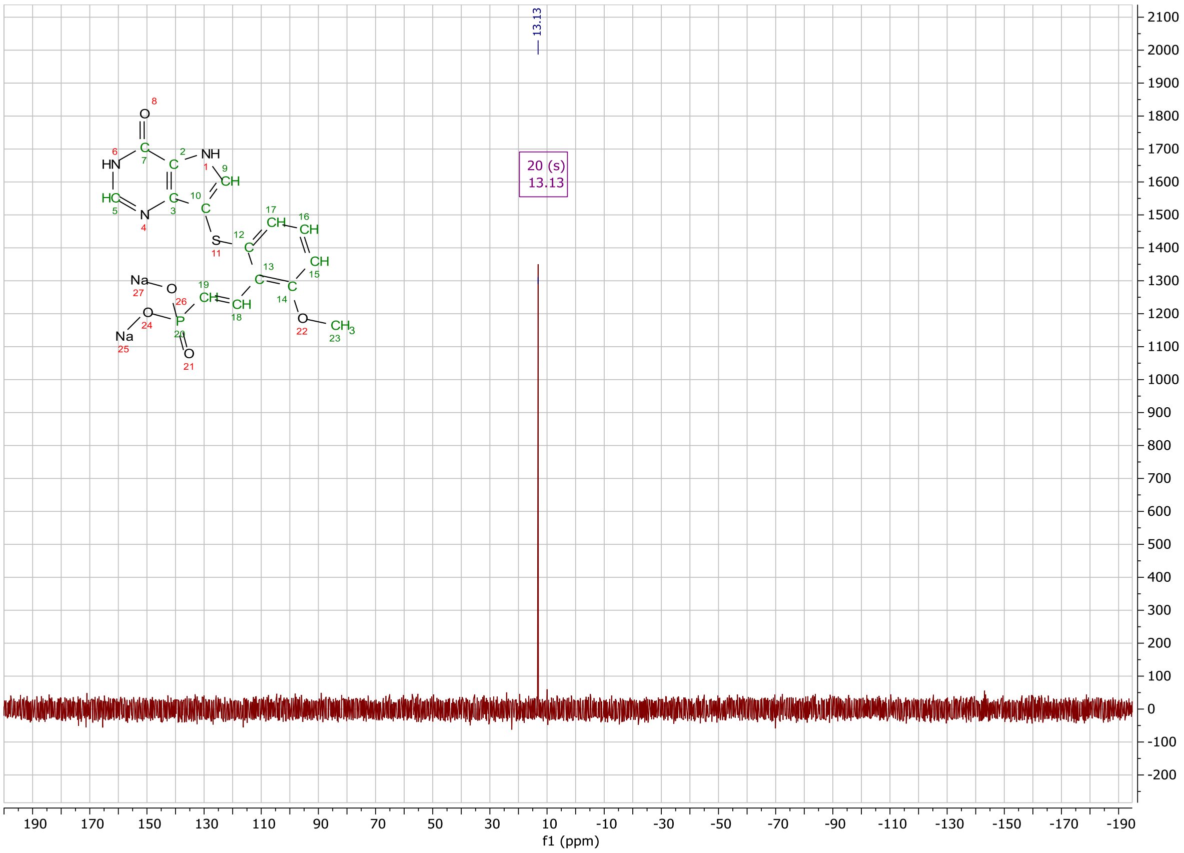
13.13
20 (s)
13.13
f1 (ppm)

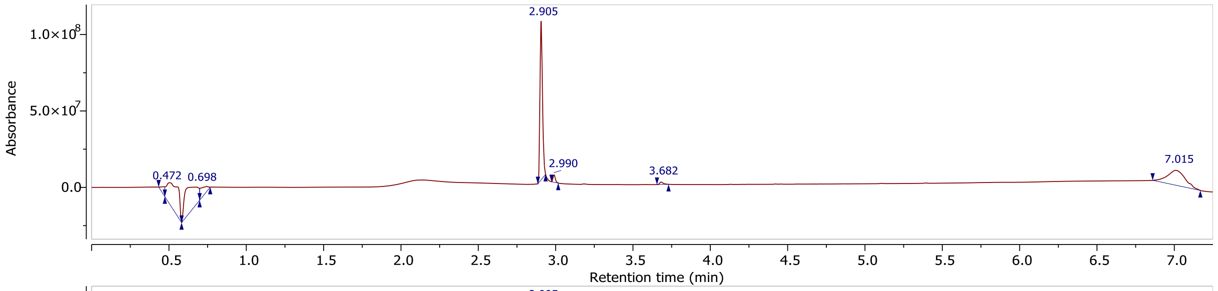
Absorbance
Retention time (min)
2.905
2.990
3.682
0.472
0.698
7.015

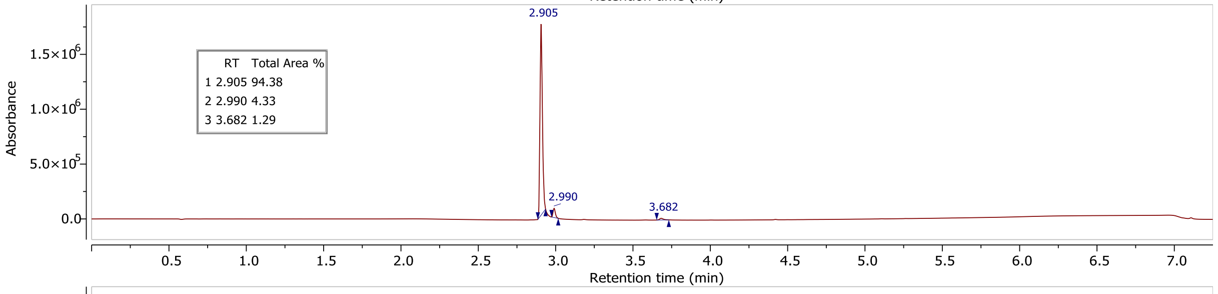
Absorbance
Retention time (min)
2.905
2.990
3.682
RT Total Area %
1 2.905 94.38
2 2.990 4.33
3 3.682 1.29

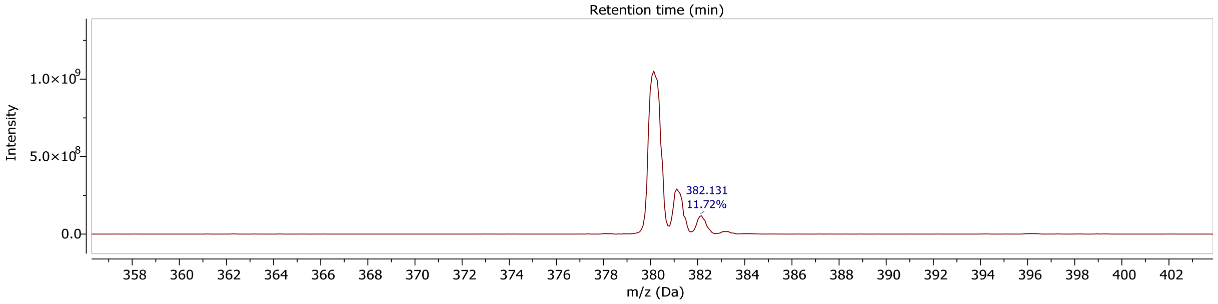
Intensity
m/z (Da)
382.131
11.72%

3.2.72 Sodium (*E*)-7-((3-isopropoxy-2-(2-(phosphonato)vinyl)phenyl)thio)-3,5-dihydro-4*H*-pyrrolo[3,2-*d*]pyrimidin-4-one (45m)

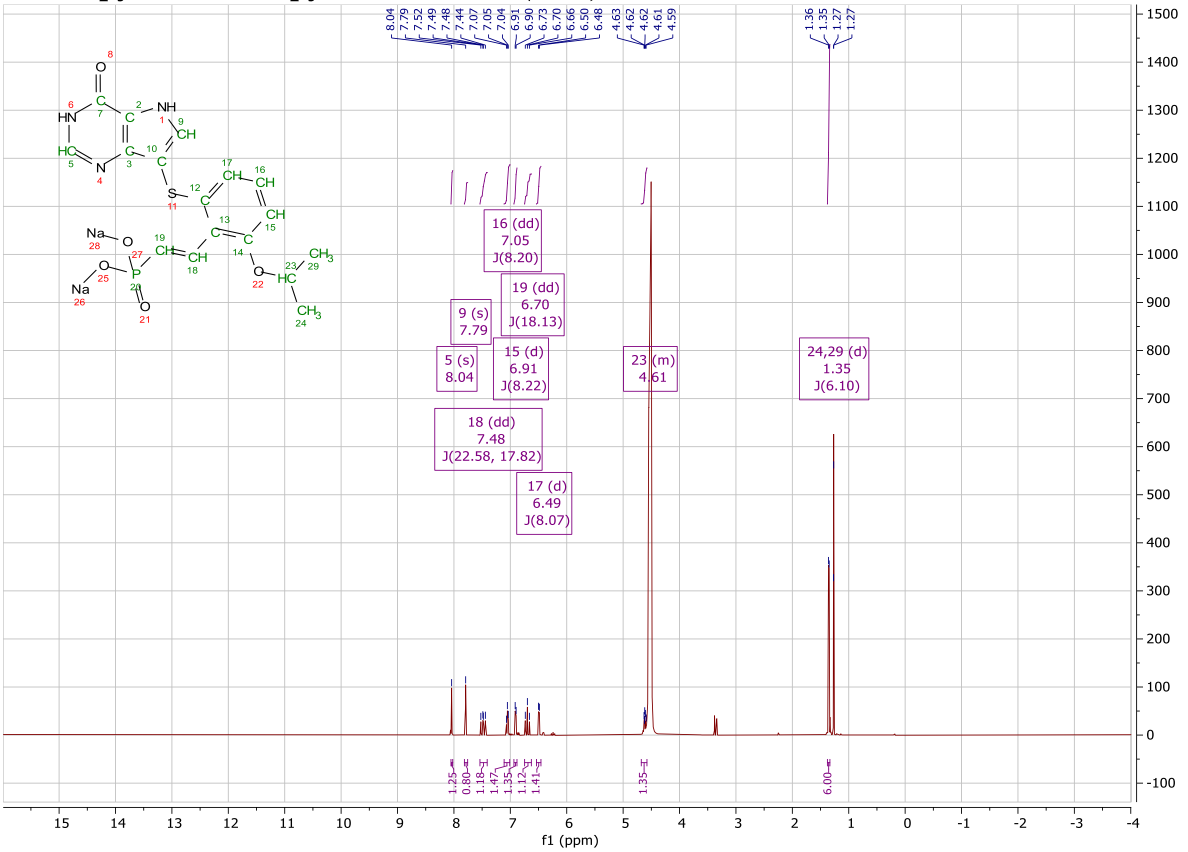

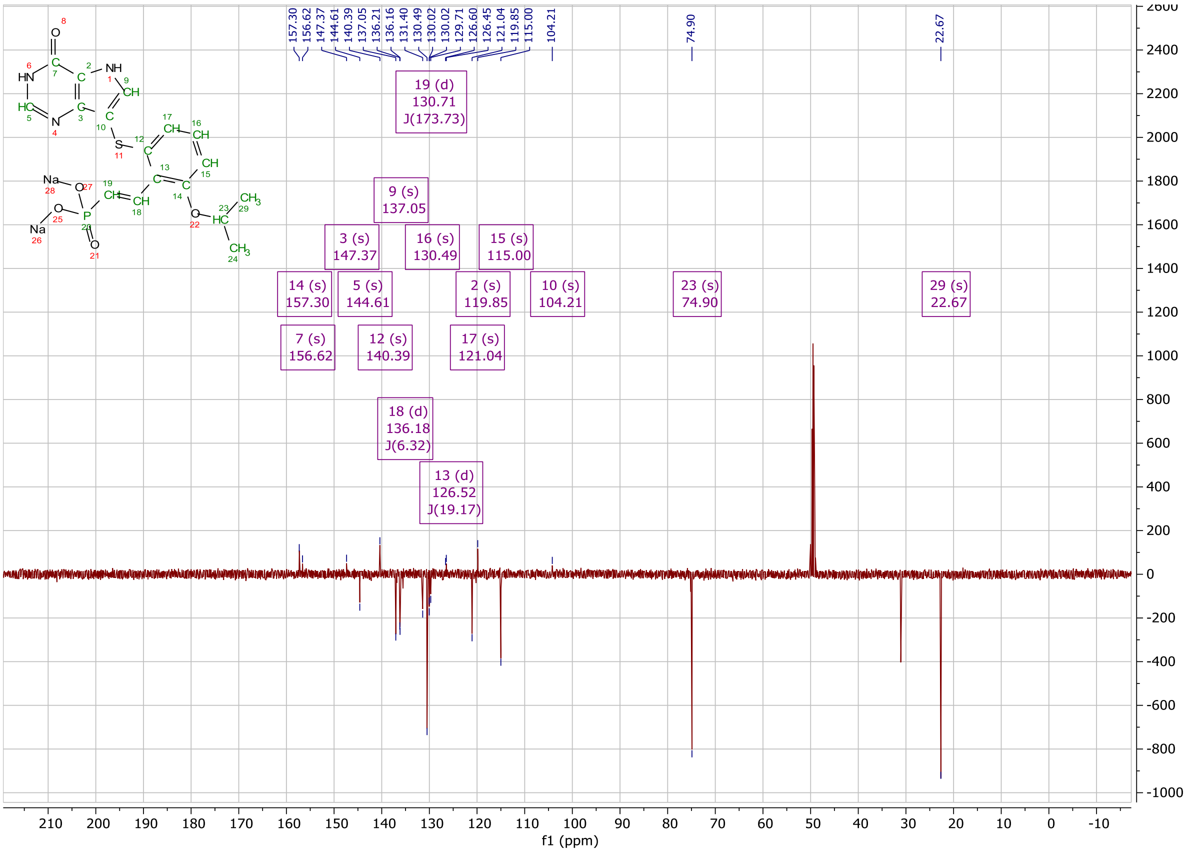

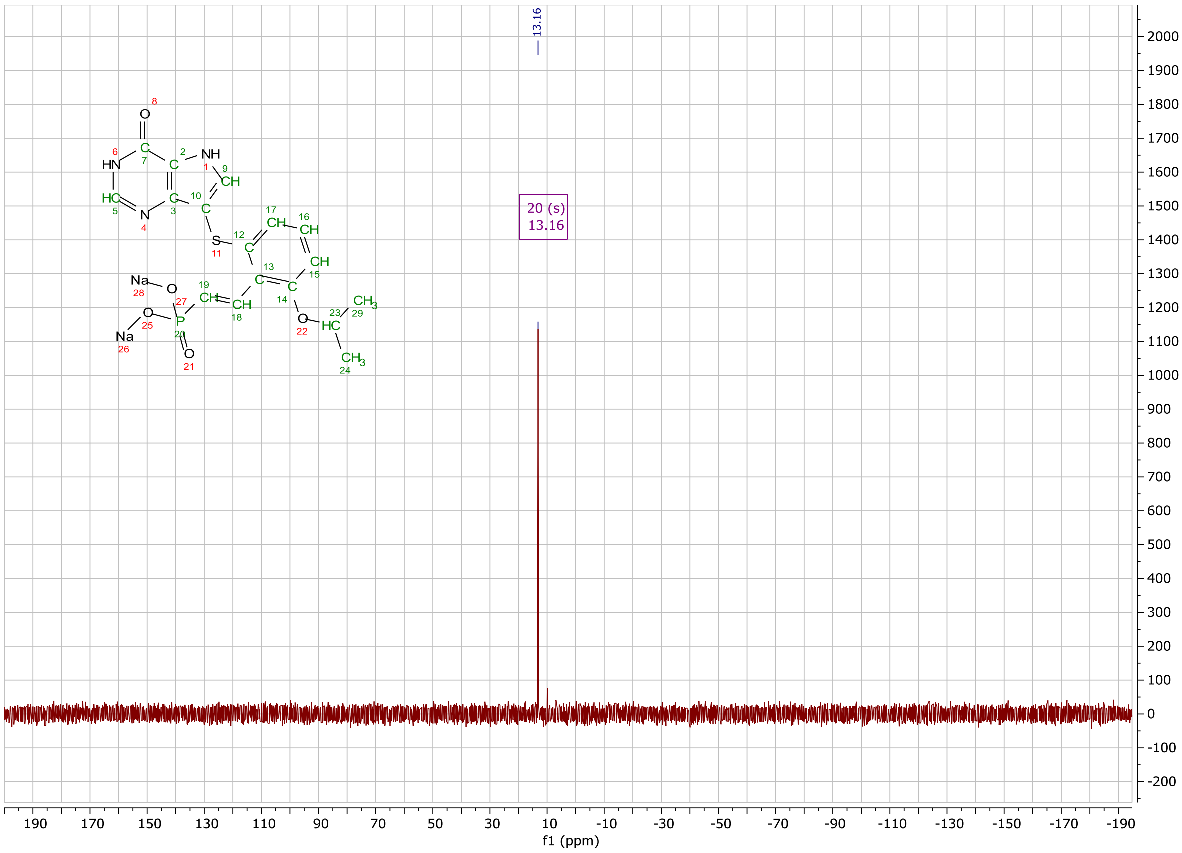
13.16
20 (s)
13.16
f1 (ppm)

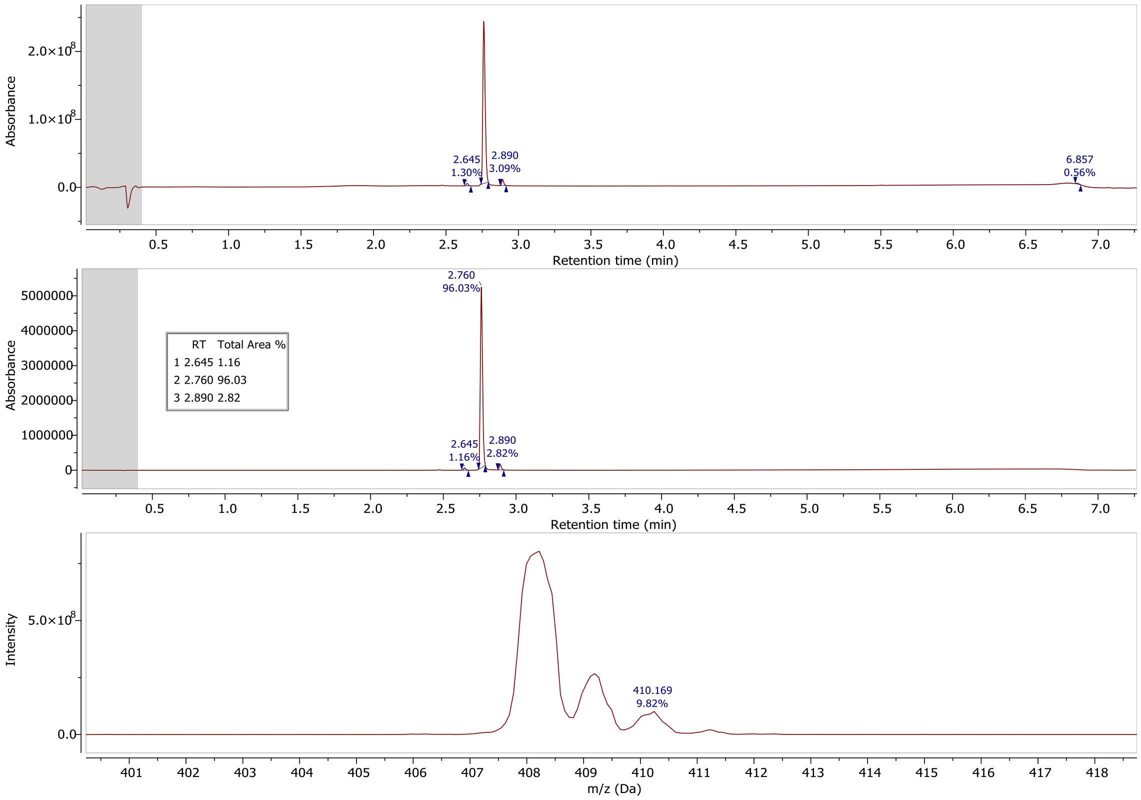
Absorbance
2.645
1.30%
2.890
3.09%
6.857
0.56%
Retention time (min)
2.760
96.03%
RT Total Area %
1 2.645 1.16
2 2.760 96.03
3 2.890 2.82
2.645
1.16%
2.890
2.82%
Retention time (min)
Intensity
410.169
9.82%
m/z (Da)

3.2.73 Sodium (*E*)-7-((3-(2,3,4,5,6-pentafluorofluorophenyl)oxy-2-(2-(phosphonato)vinyl)phenyl)thio)-3,5-dihydro-4*H*-pyrrolo[3,2-*d*]pyrimidin-4-one (45n)

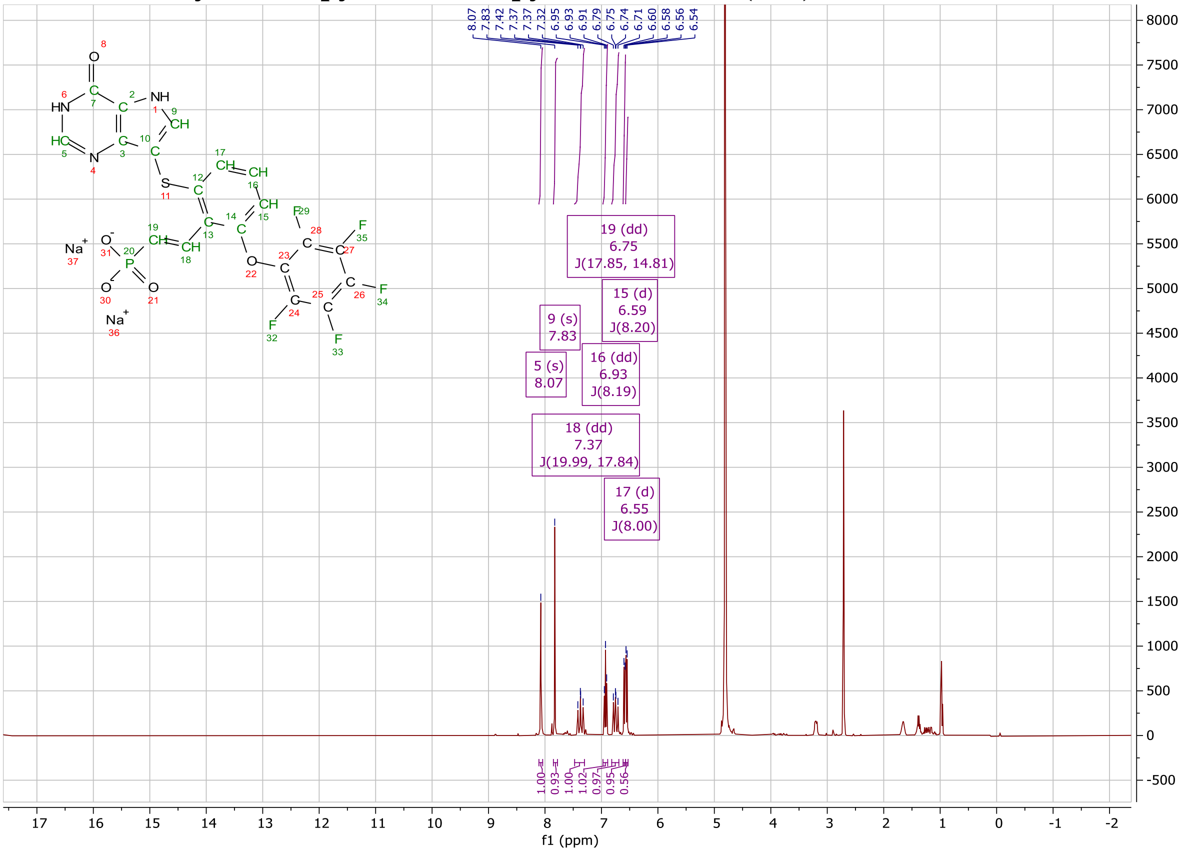

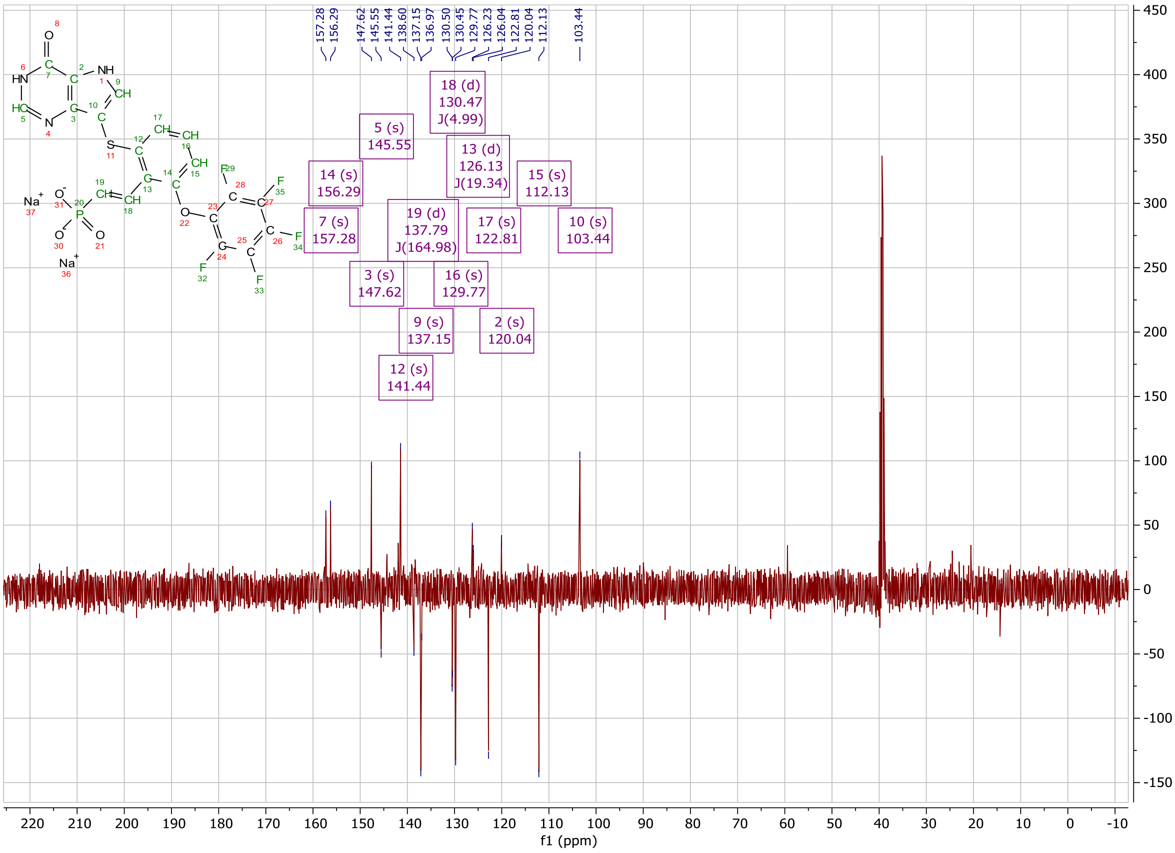

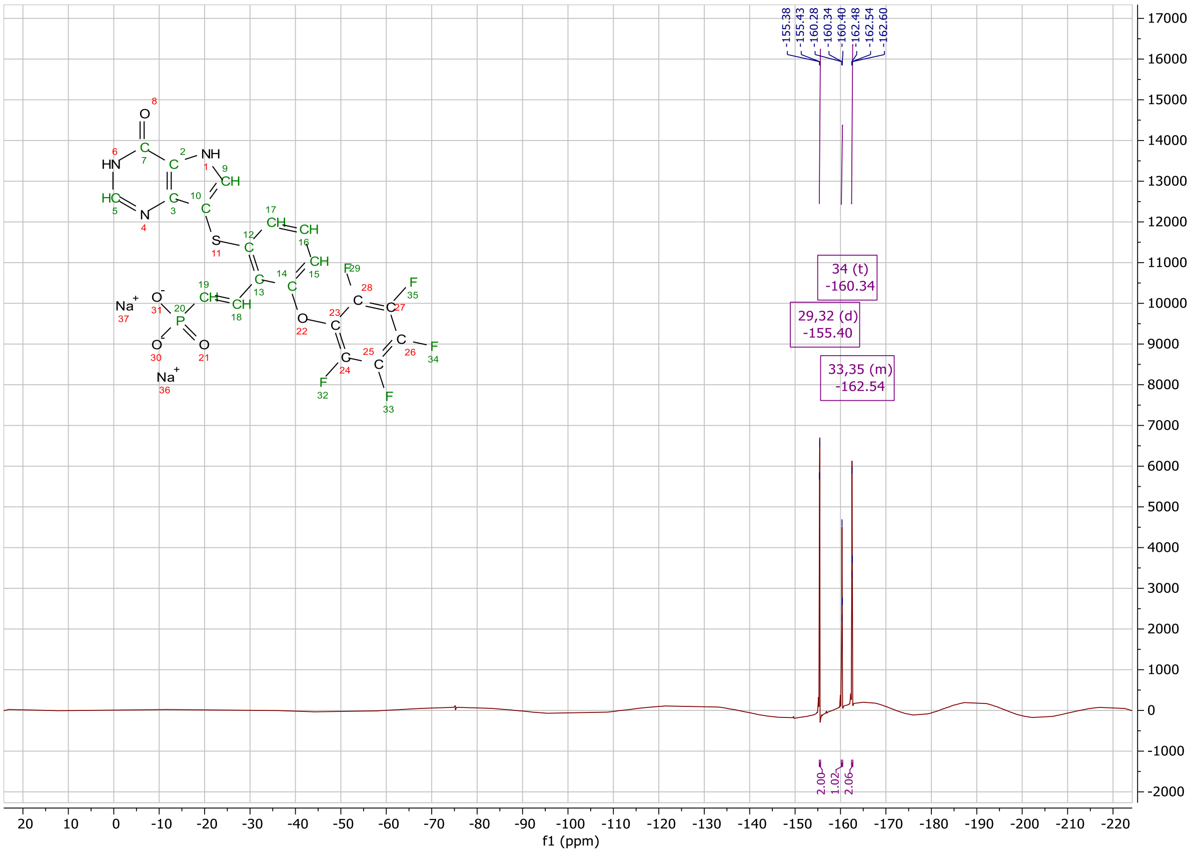

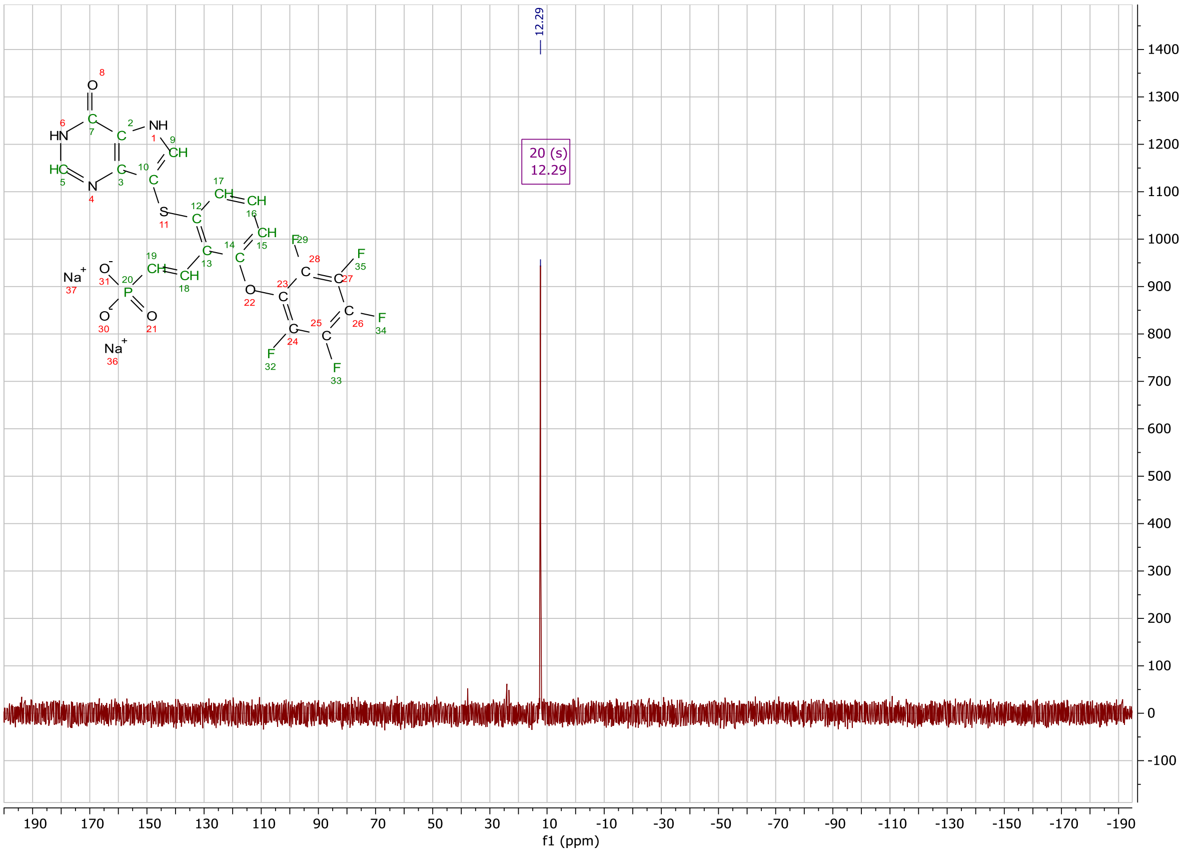

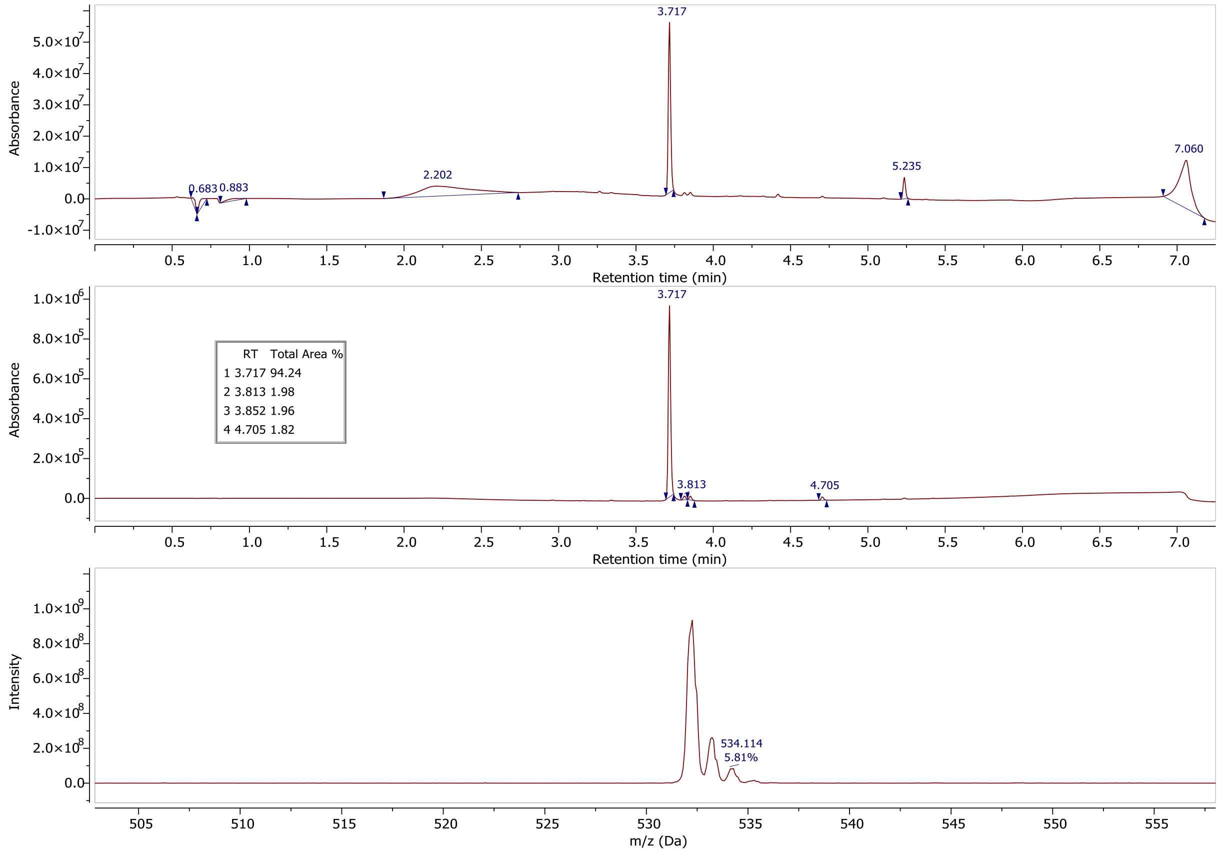

3.2.74 Sodium (*E*)-7-((3-(2,3,4,5,6-pentafluorofluorophenyl)methoxy-2-(2-(phosphonato)vinyl)phenyl)thio)-3,5-dihydro-4*H*-pyrrolo[3,2-*d*]pyrimidin-4-one (45o)

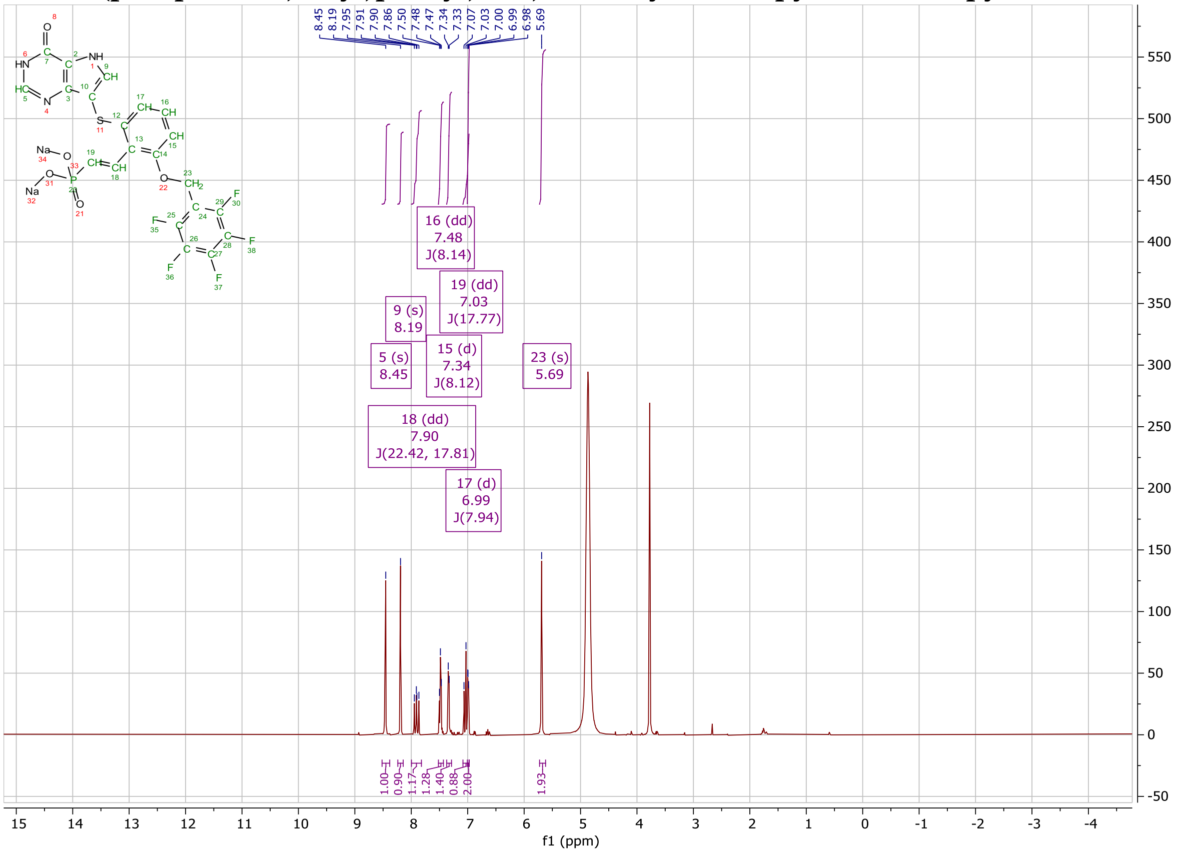

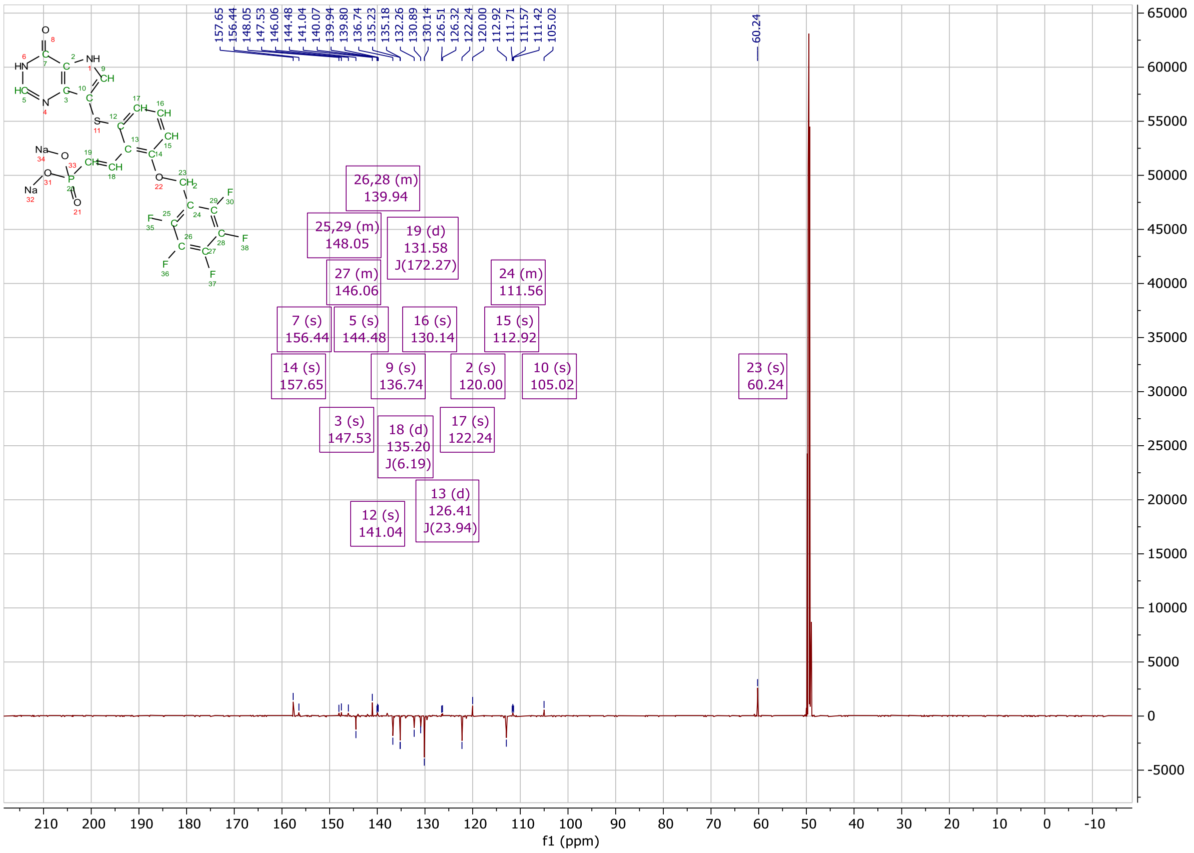
157.65
156.44
148.05
147.53
146.06
144.48
141.04
140.07
139.94
139.80
136.74
135.23
135.18
132.26
130.89
130.14
126.51
126.32
122.24
120.00
112.92
111.71
111.57
111.42
105.02
60.24
26,28 (m) 139.94
25,29 (m) 148.05
19 (d) 131.58 J(172.27)
27 (m) 146.06
24 (m) 111.56
7 (s) 156.44
5 (s) 144.48
16 (s) 130.14
15 (s) 112.92
14 (s) 157.65
9 (s) 136.74
2 (s) 120.00
10 (s) 105.02
23 (s) 60.24
3 (s) 147.53
18 (d) 135.20 J(6.19)
17 (s) 122.24
13 (d) 126.41 J(23.94)
12 (s) 141.04
f1 (ppm)

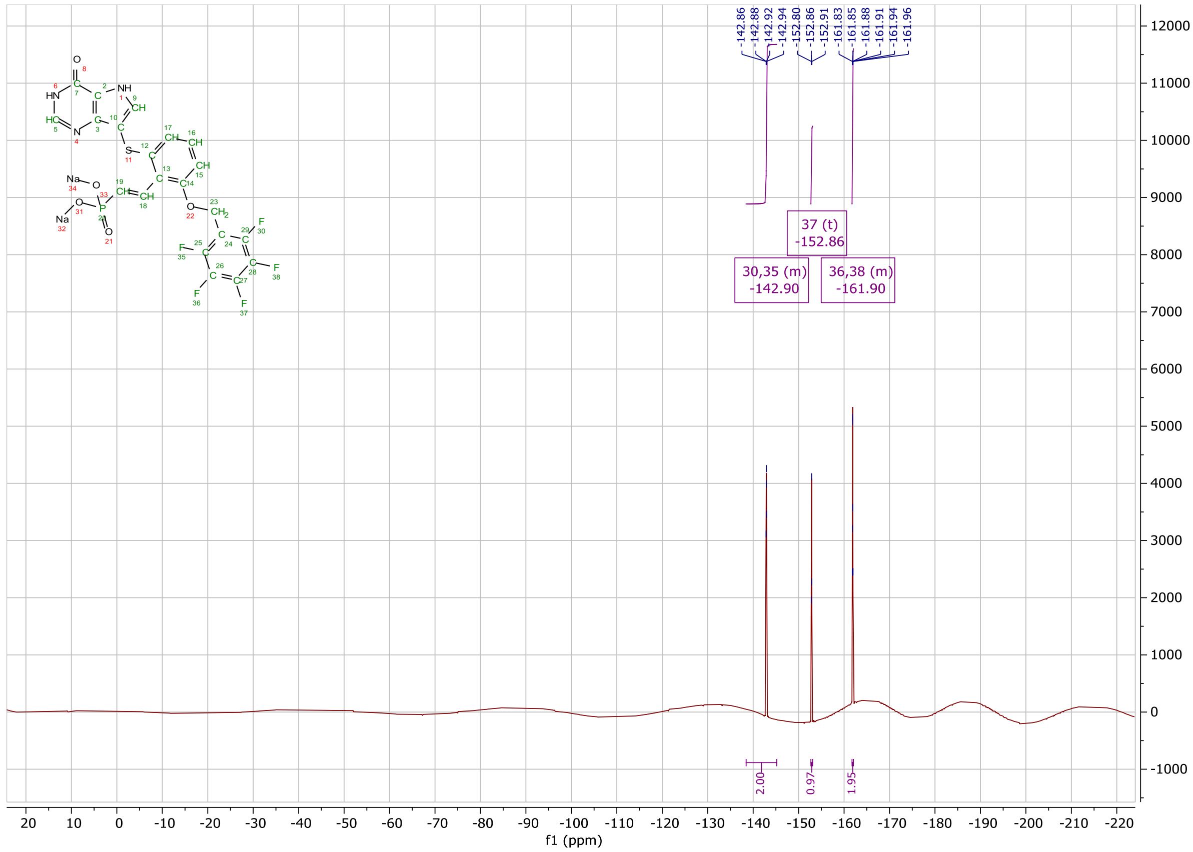
-142.86
-142.88
-142.92
-142.94
-152.80
-152.86
-152.91
-161.83
-161.85
-161.88
-161.91
-161.94
-161.96
37 (t) -152.86
30,35 (m) -142.90
36,38 (m) -161.90
2.00
0.97
1.95
f1 (ppm)

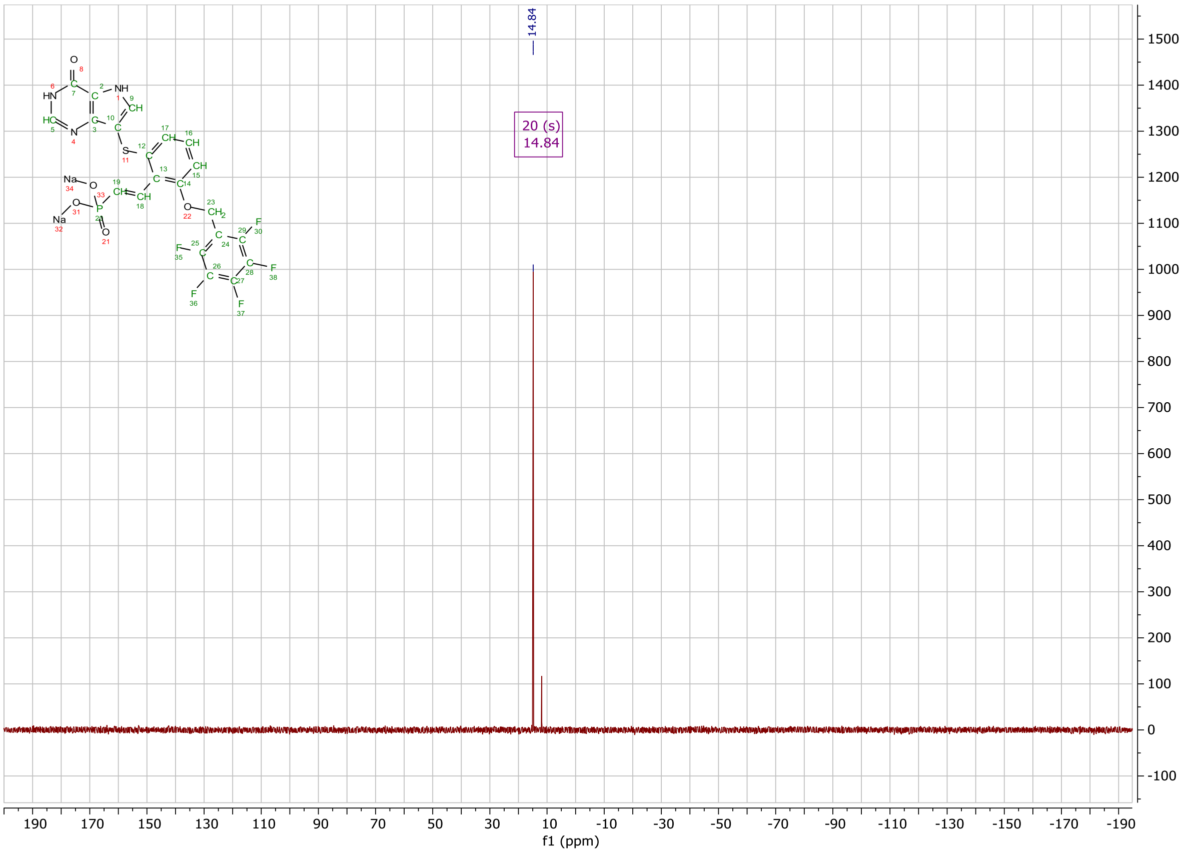
14.84
20 (s)
14.84
f1 (ppm)

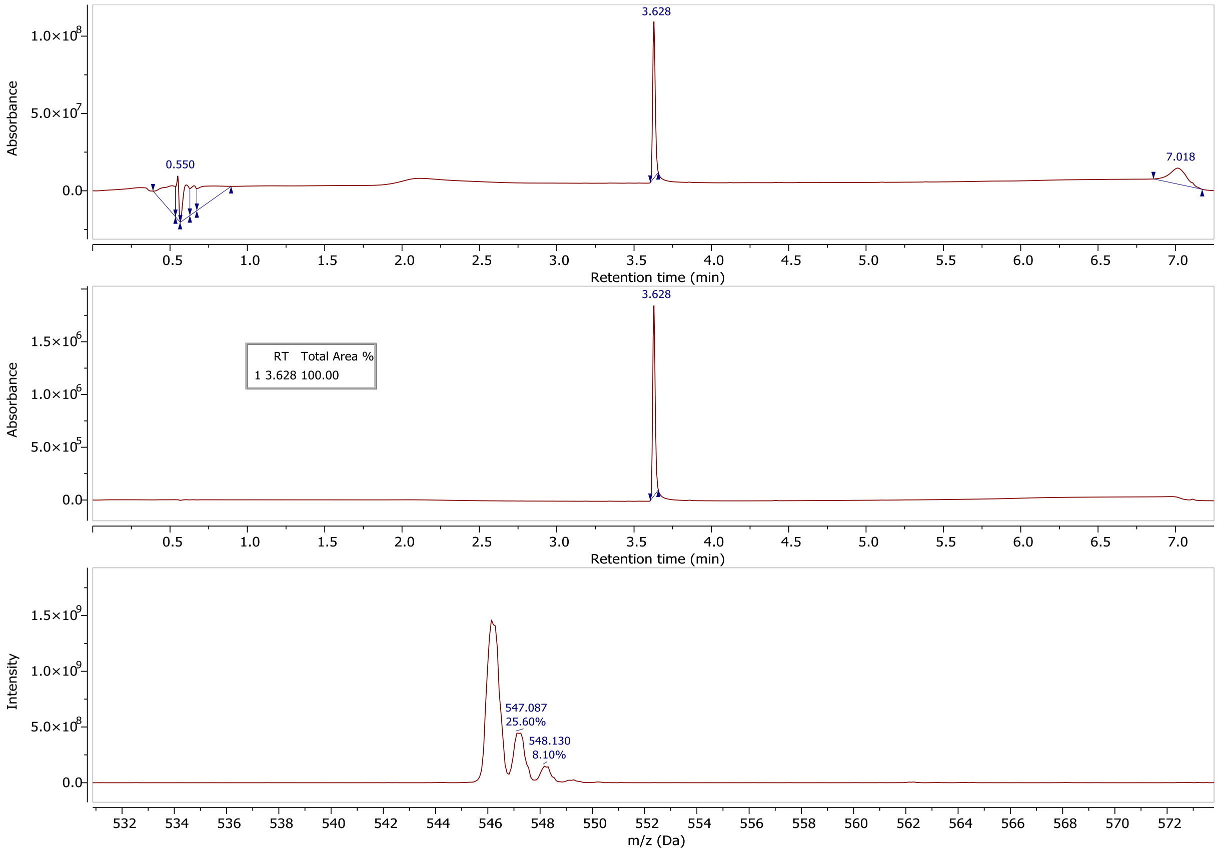
3.628
0.550
7.018
Absorbance
Retention time (min)
3.628
RT Total Area %
1 3.628 100.00
Absorbance
Retention time (min)
547.087
25.60%
548.130
8.10%
Intensity
m/z (Da)

3.2.75 Sodium (*E*)-7-((5-bromo-2-(2-(phosphonato)vinyl)phenyl)thio)-3,5-dihydro-4*H*-pyrrolo[3,2-*d*]pyrimidin-4-one (45p)

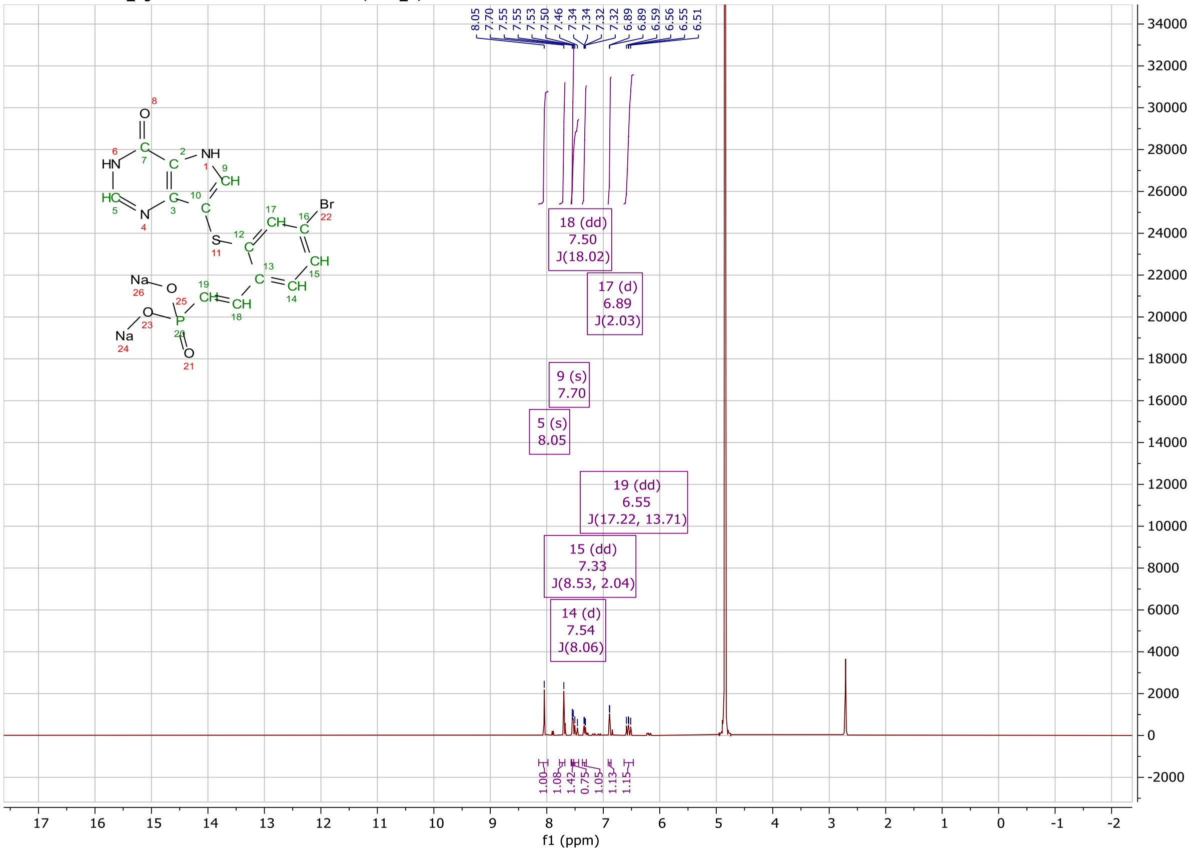

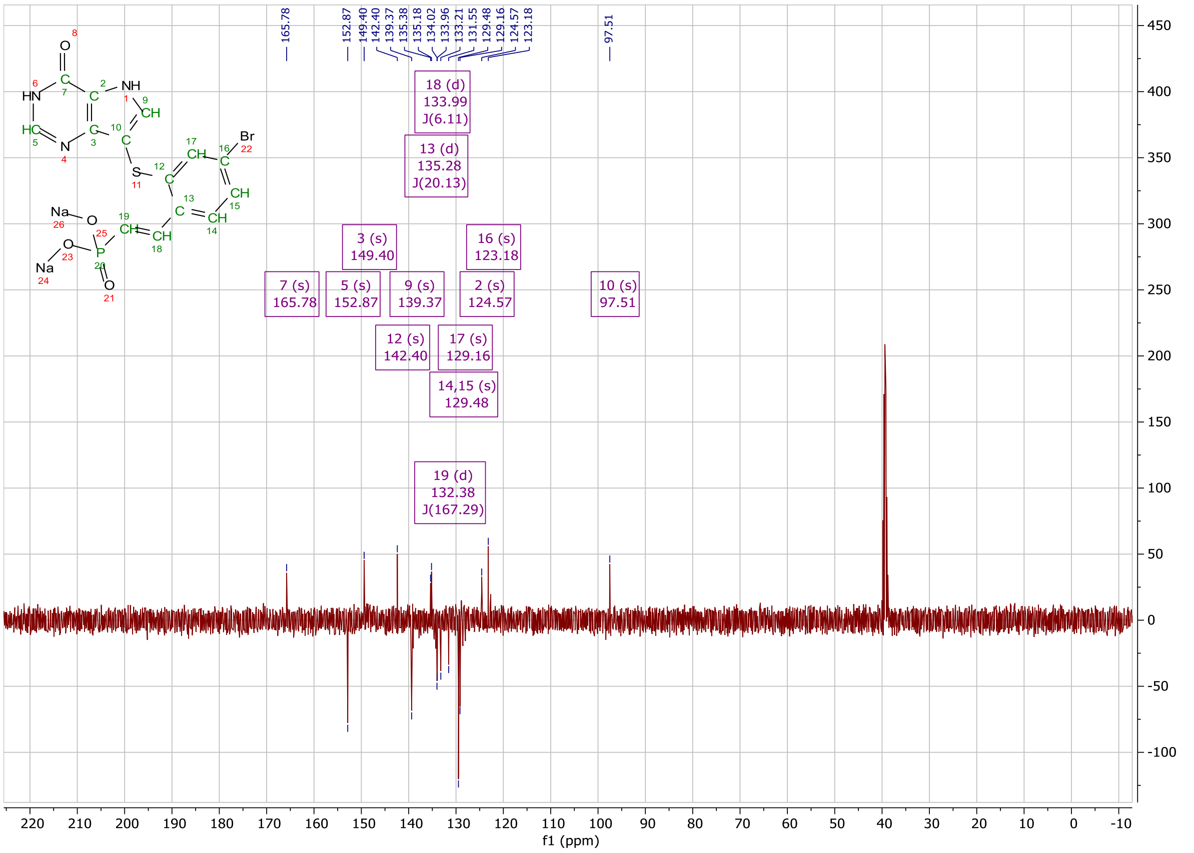

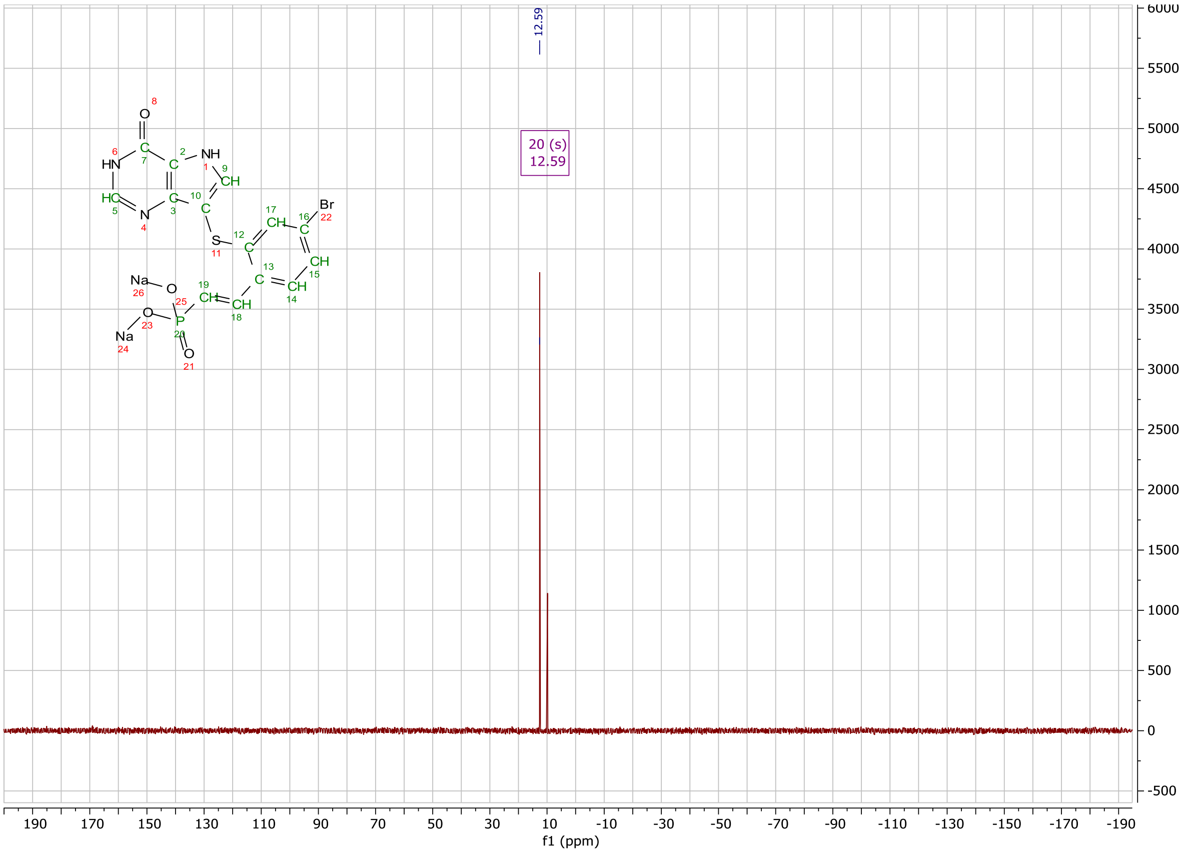
12.59
20 (s)
12.59
f1 (ppm)
Br

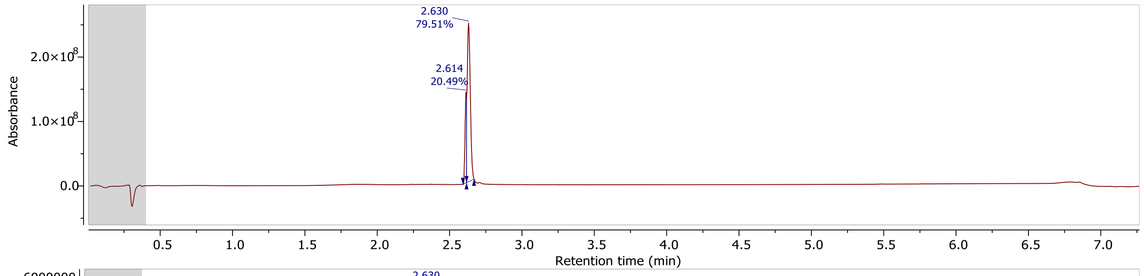
2.630
79.51%
2.614
20.49%
Absorbance
Retention time (min)

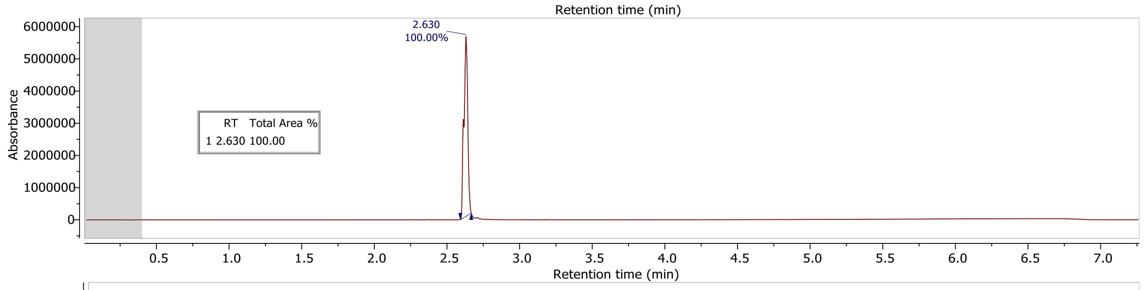
2.630
100.00%
RT Total Area %
1 2.630 100.00
Absorbance
Retention time (min)

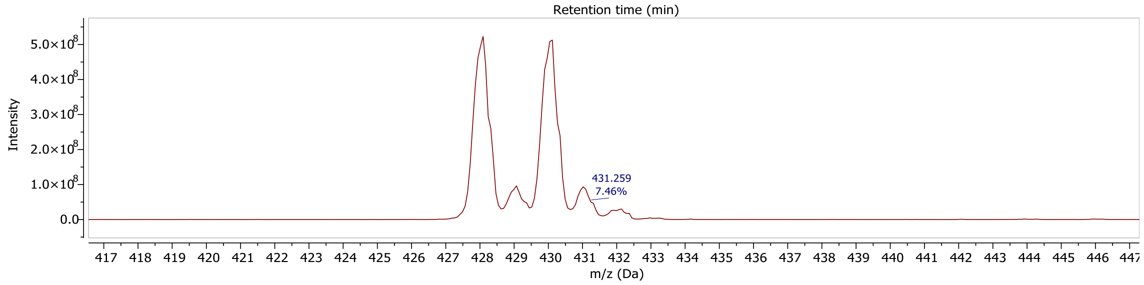
431.259
7.46%
Intensity
m/z (Da)

3.2.76 Sodium (*E*)-7-((4-bromo-2-(2-(phosphonato)vinyl)phenyl)thio)-3,5-dihydro-4*H*-pyrrolo[3,2-*d*]pyrimidin-4-one (45q)

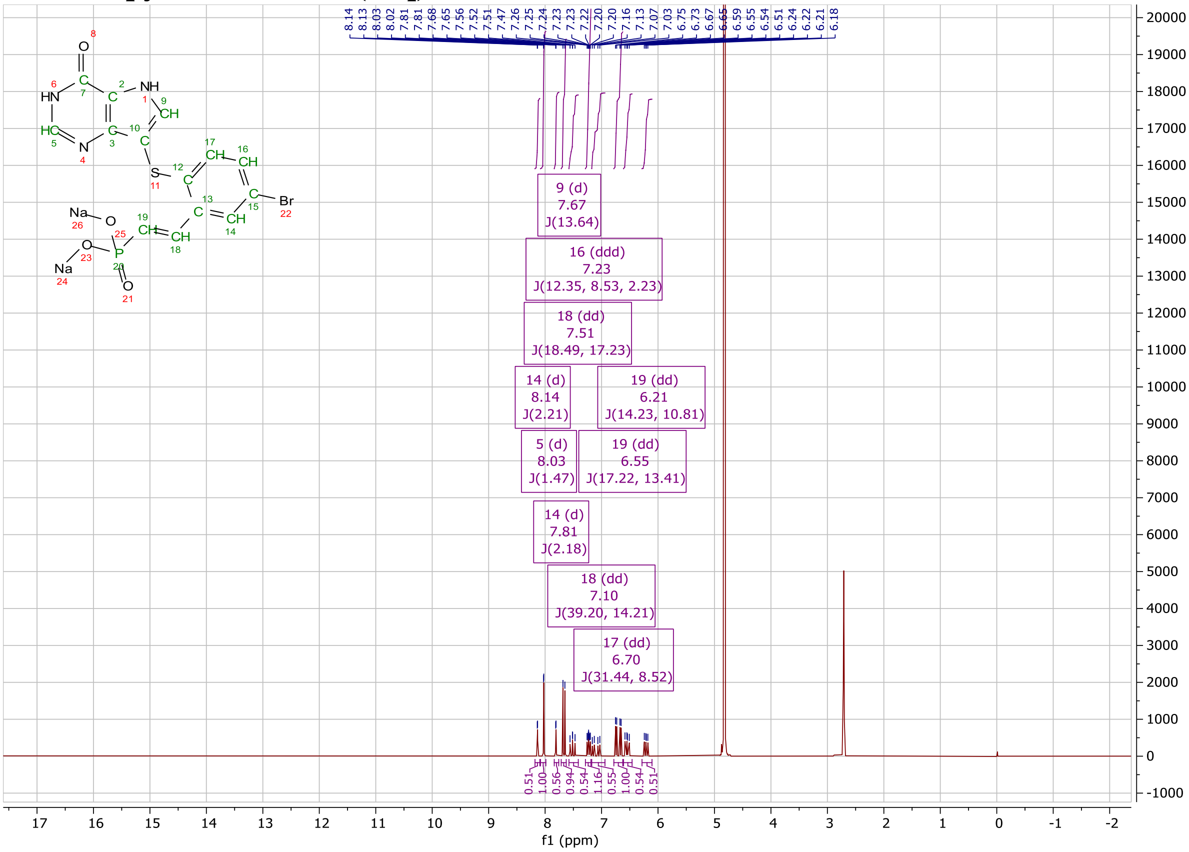

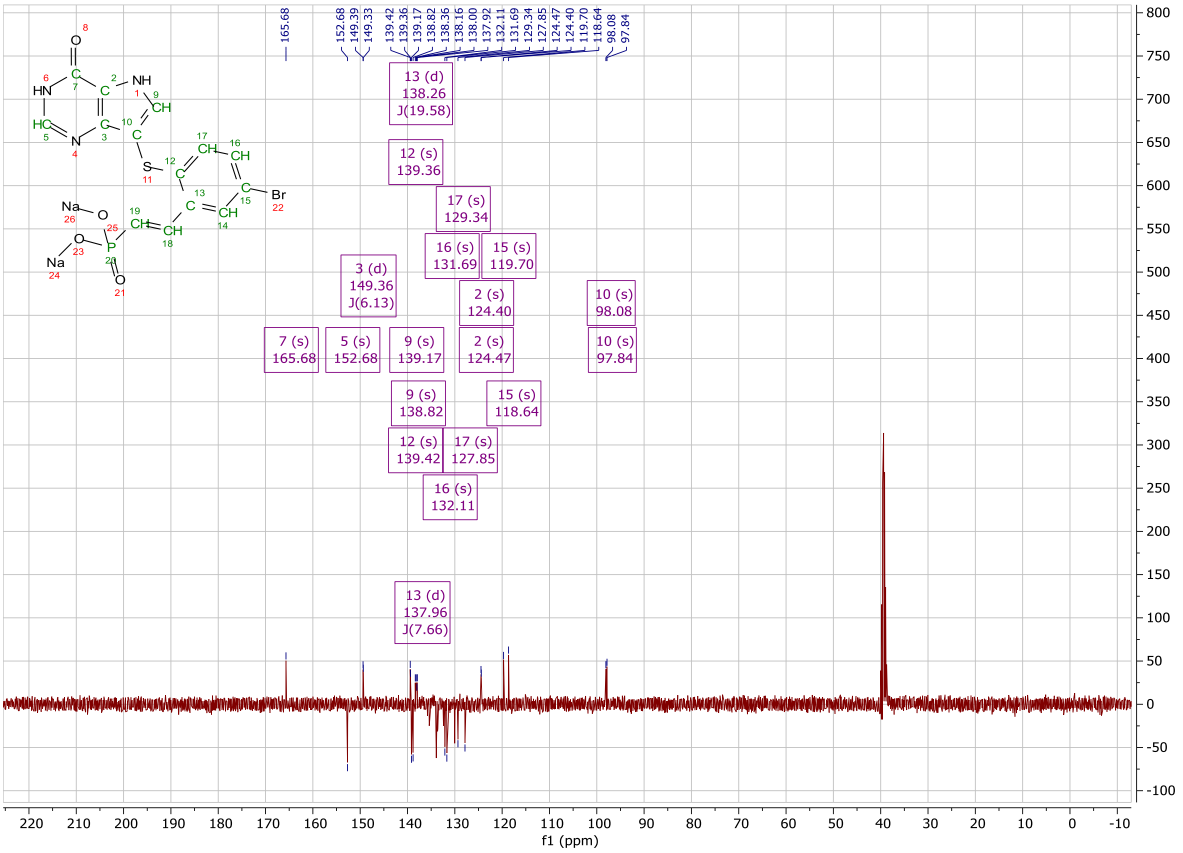

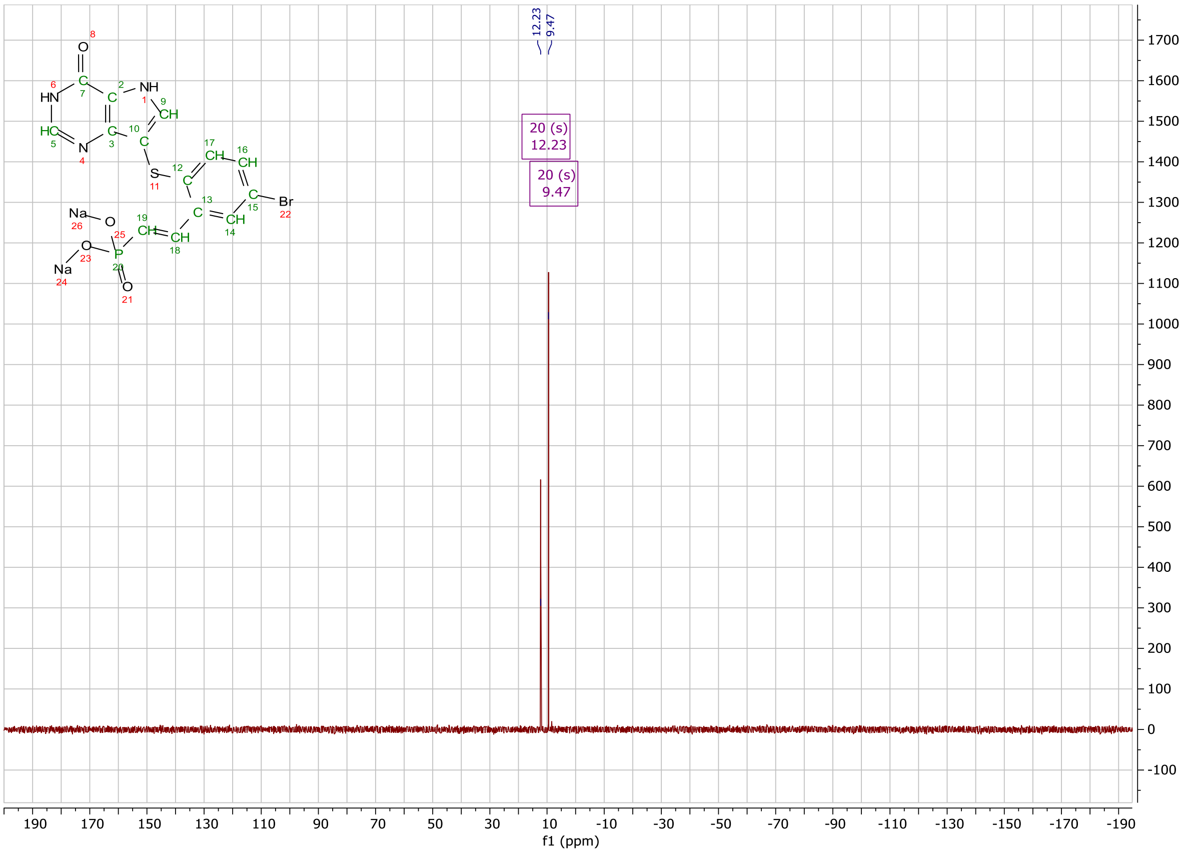

12.23
9.47
20 (s)
12.23
20 (s)
9.47
f1 (ppm)

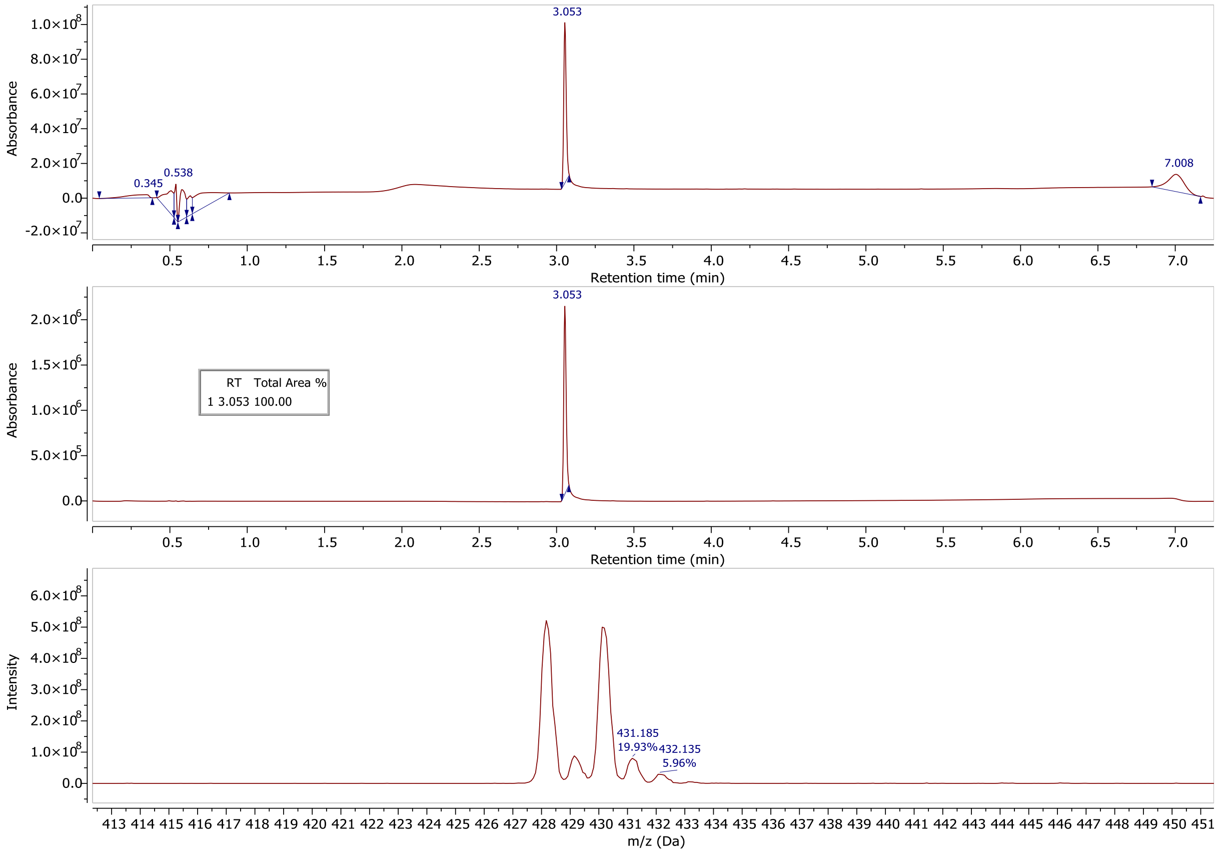

Absorbance
3.053
0.345
0.538
7.008
Retention time (min)
RT Total Area %
1 3.053 100.00
Intensity
431.185
19.93%
432.135
5.96%
m/z (Da)

3.2.77 Diisopropyl ((4-fluoro-2-iodophenoxy)methyl)phosphonate (47a)

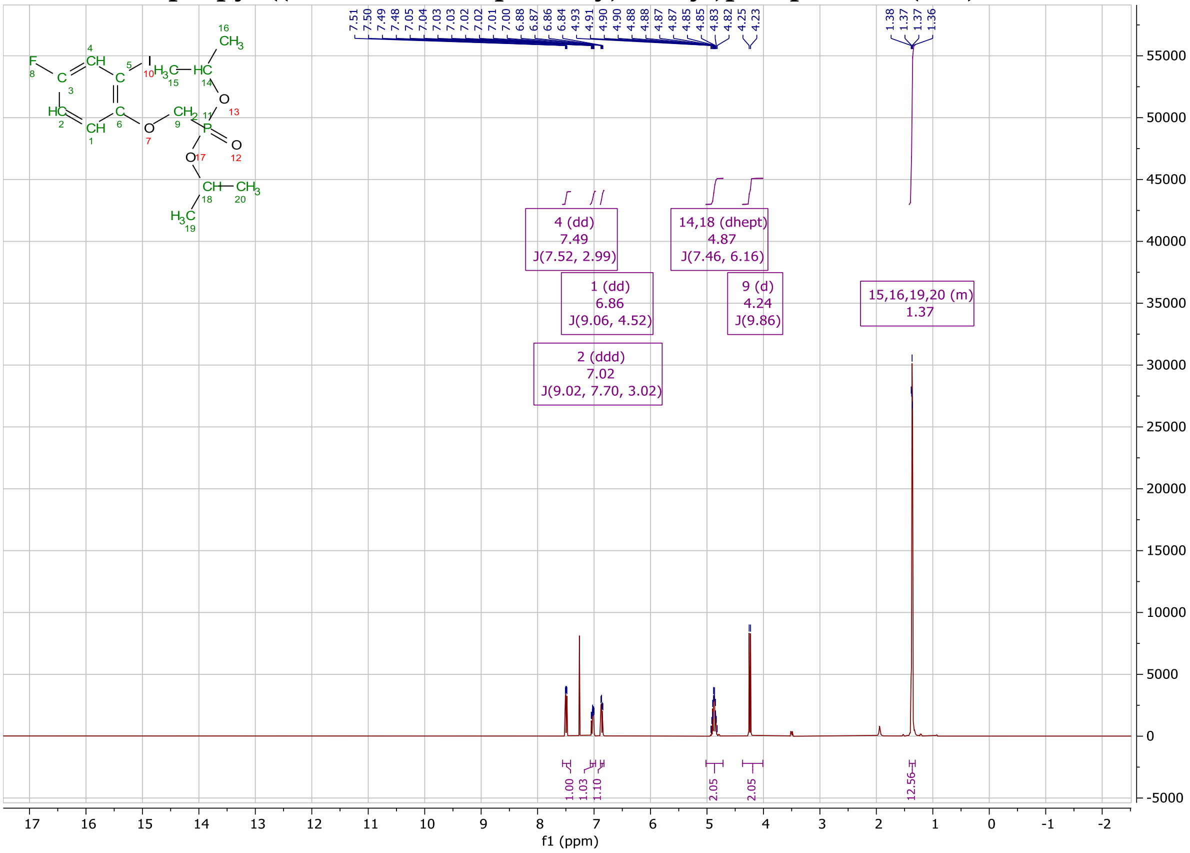

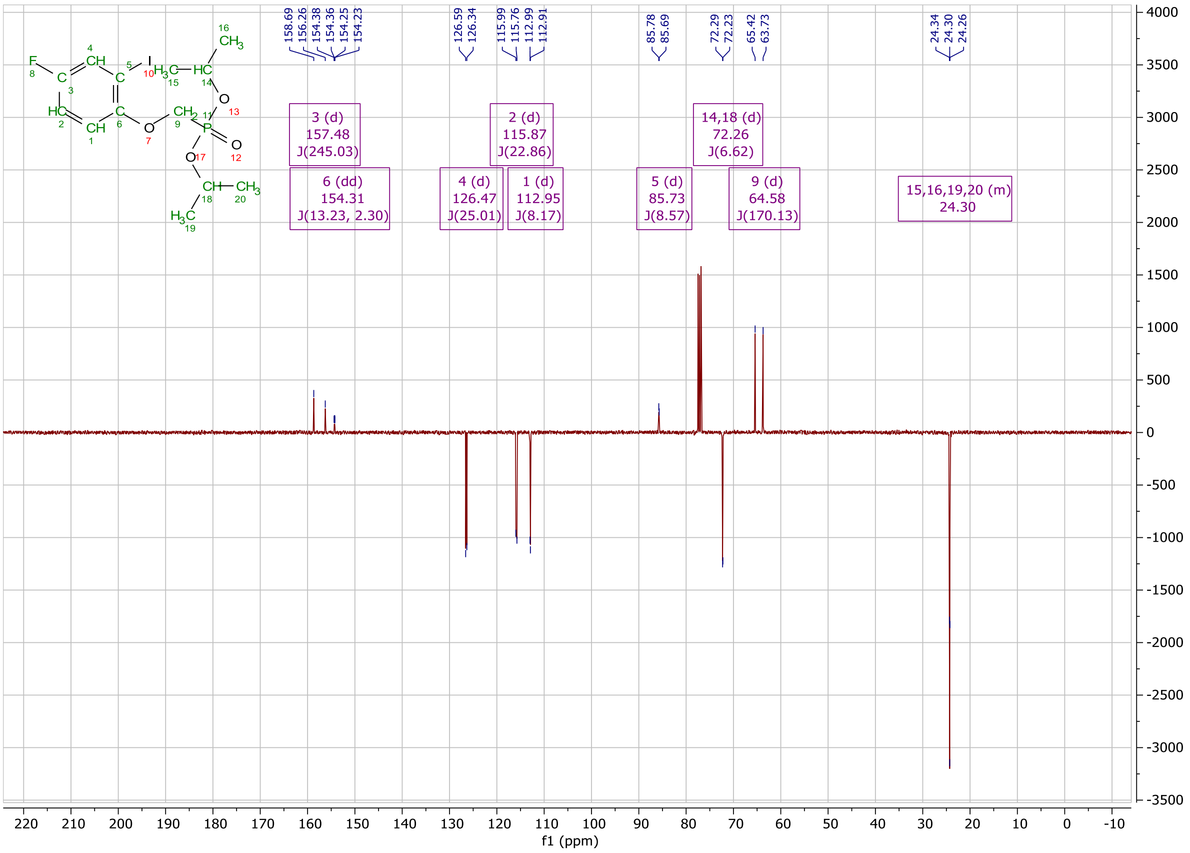

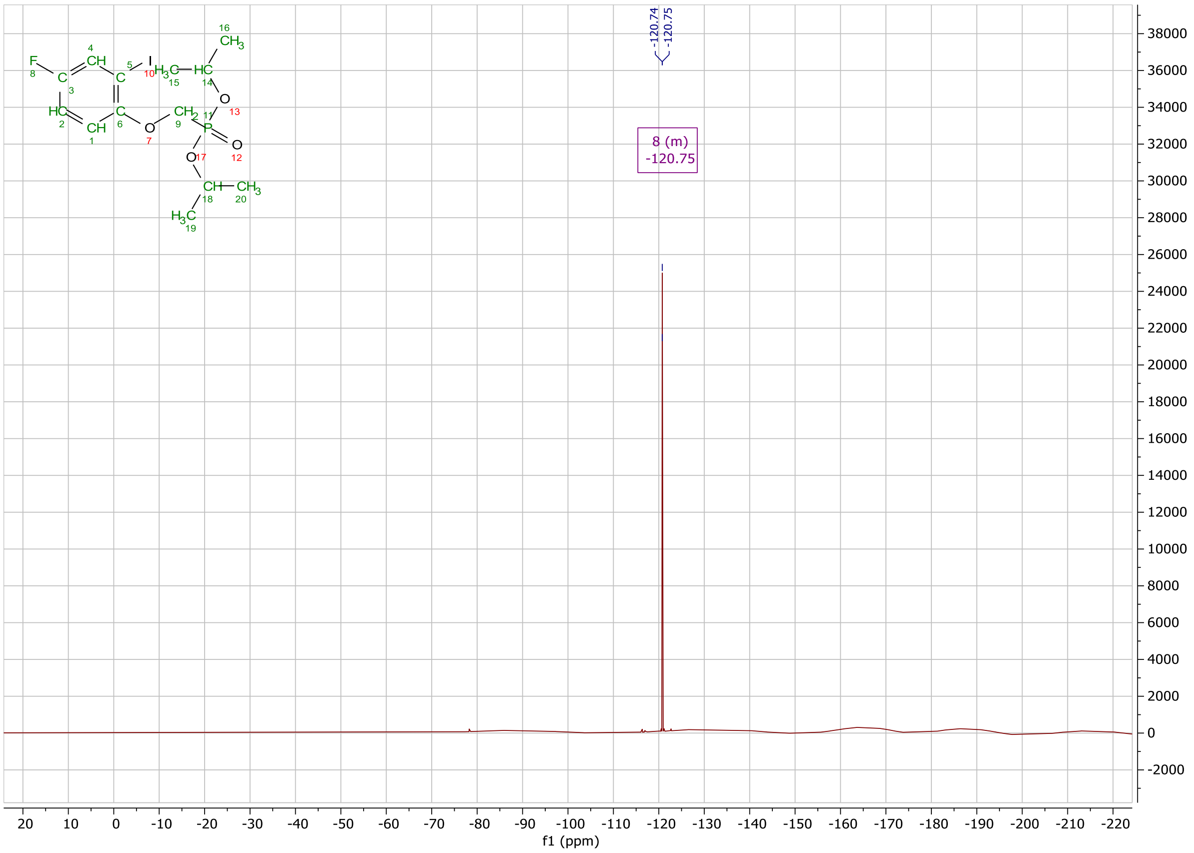
-120.74
-120.75
8 (m)
-120.75
f1 (ppm)

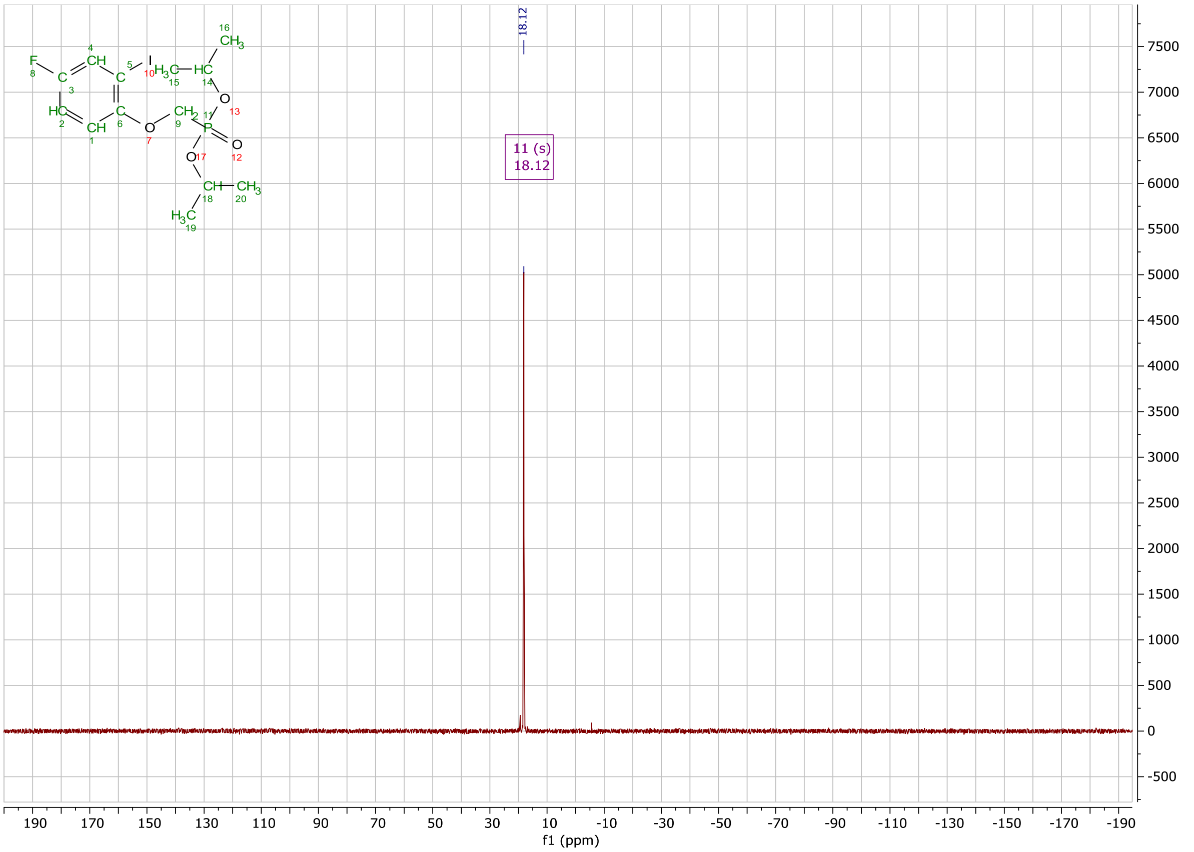
18.12
11 (s)
18.12
f1 (ppm)

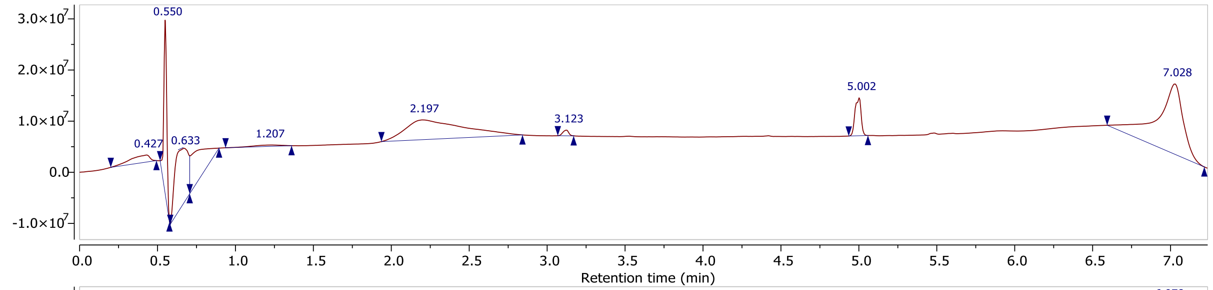

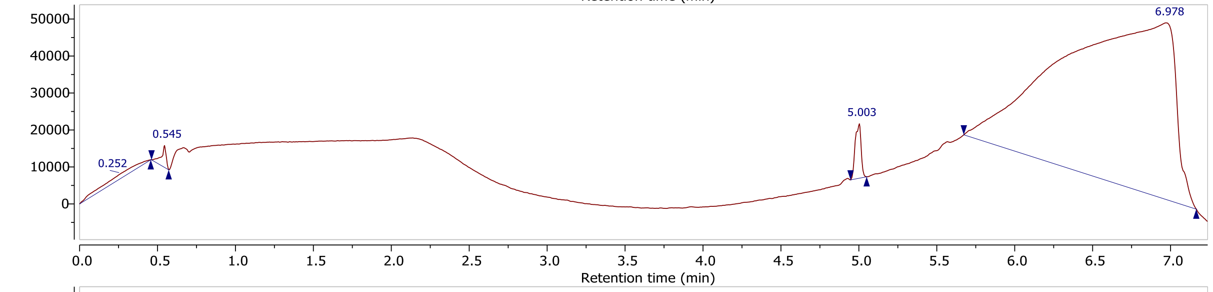

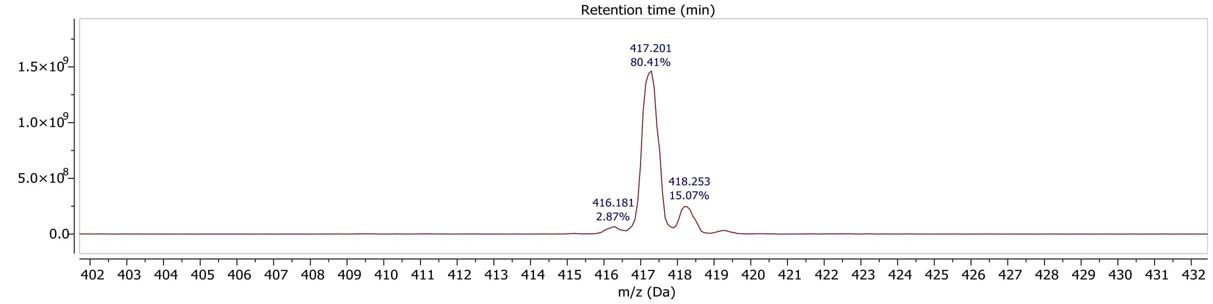

3.2.78 Diisopropyl ((2-iodo-6-methoxyphenoxy)methyl)phosphonate (47b)

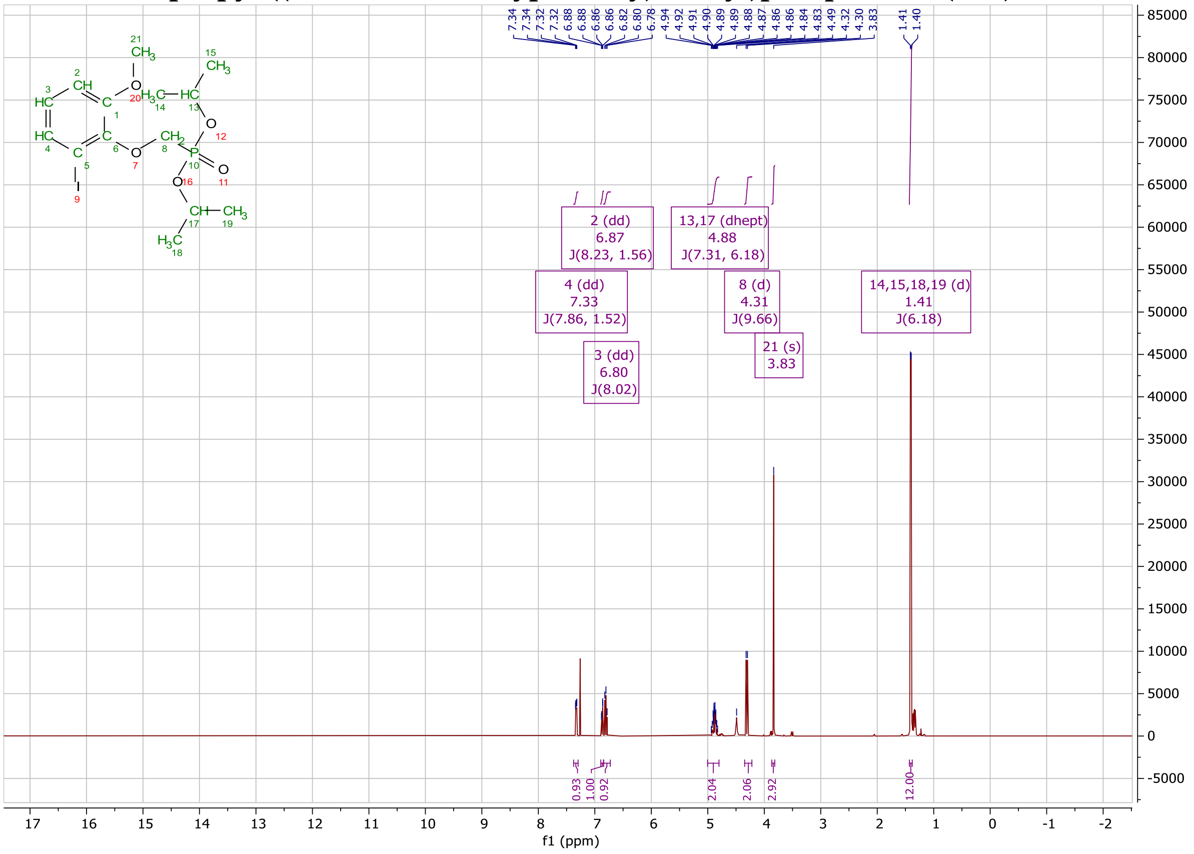

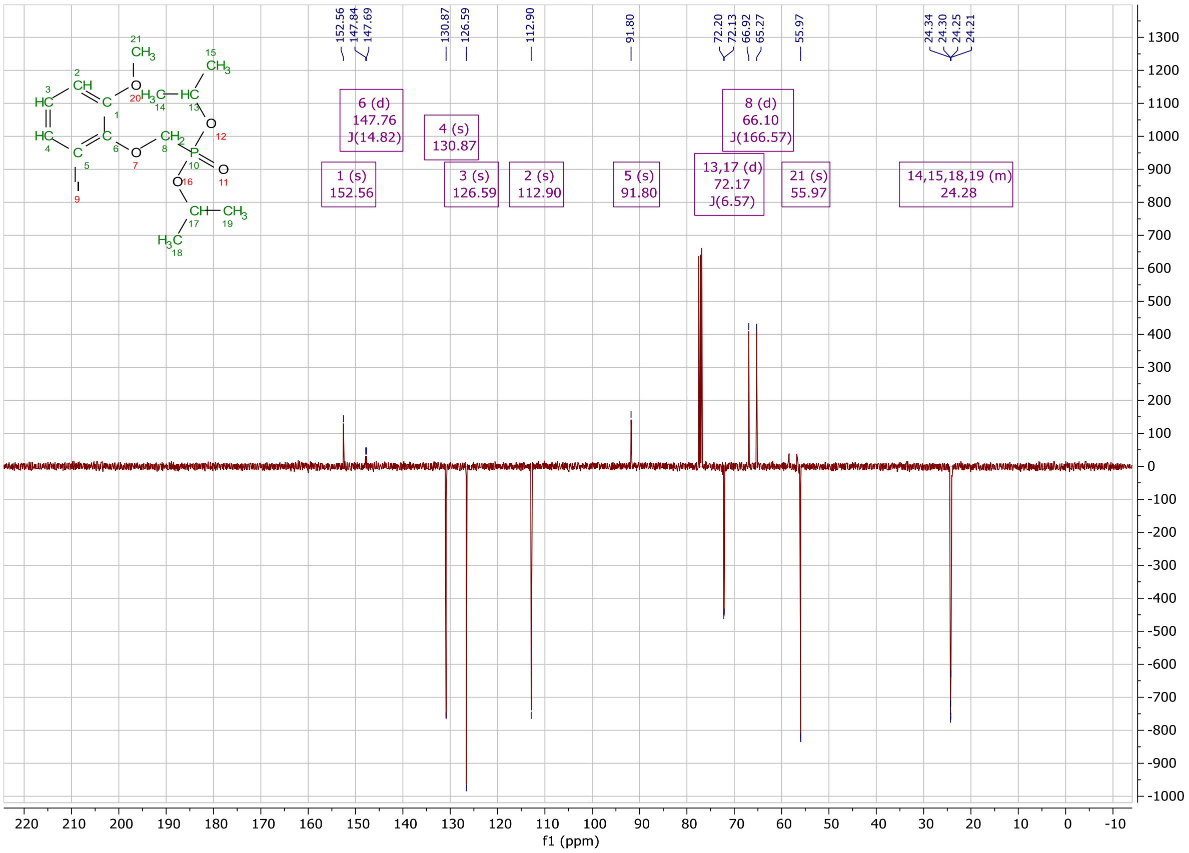

152.56
147.84
147.69
130.87
126.59
112.90
91.80
72.20
72.13
66.92
65.27
55.97
24.34
24.30
24.25
24.21
6 (d)
147.76
J(14.82)
4 (s)
130.87
8 (d)
66.10
J(166.57)
1 (s)
152.56
3 (s)
126.59
2 (s)
112.90
5 (s)
91.80
13,17 (d)
72.17
J(6.57)
21 (s)
55.97
14,15,18,19 (m)
24.28
f1 (ppm)

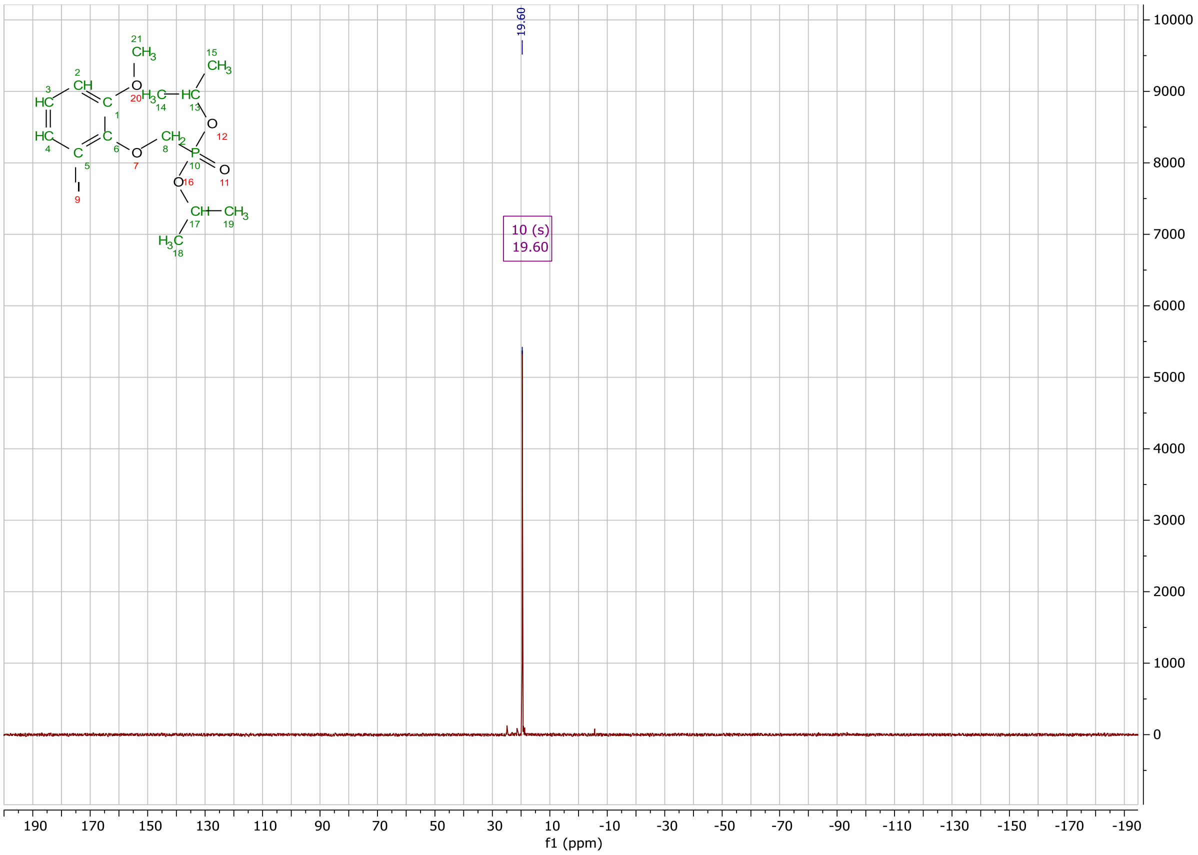

19.60
10 (s)
19.60
f1 (ppm)

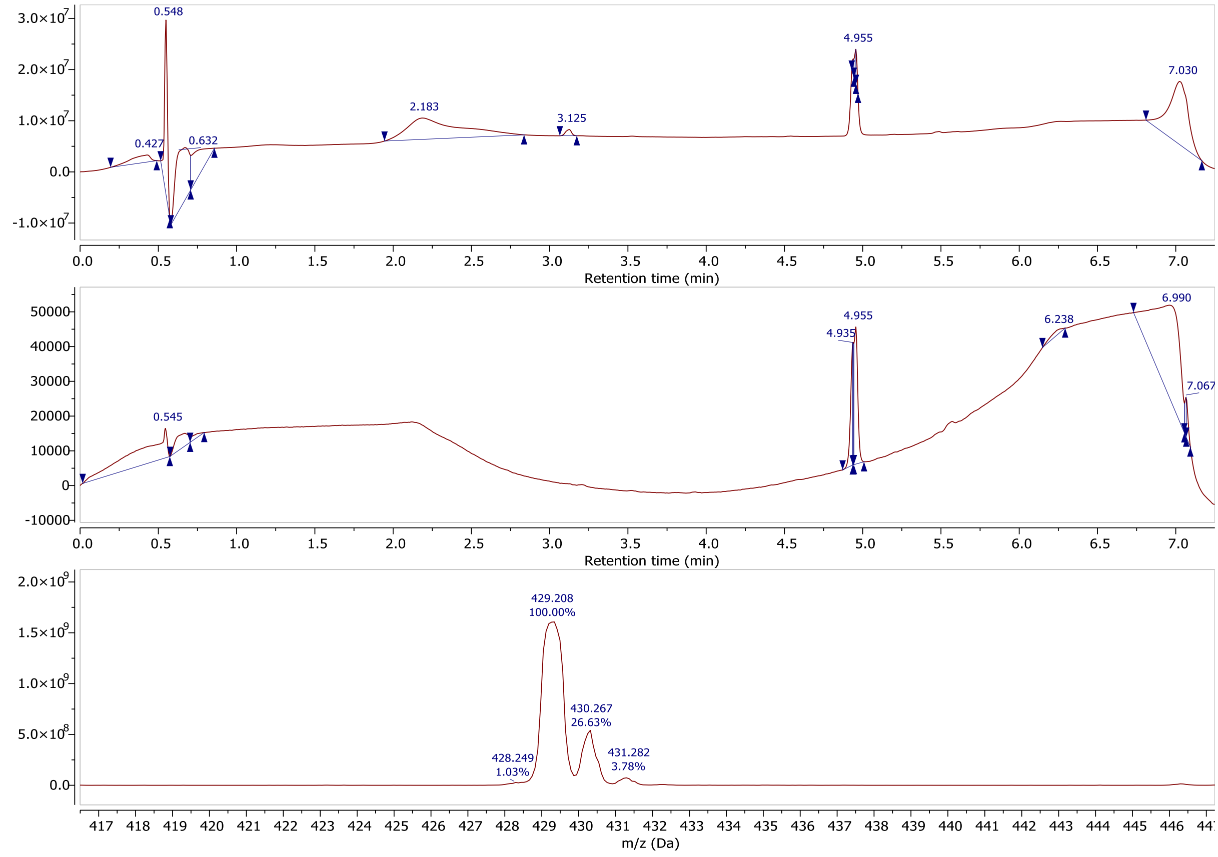

3.2.79 Sodium 7-((5-fluoro-2-((phosphonato)methoxy)phenyl)thio)-3,5-dihydro-4*H*-pyrrolo[3,2-*d*]pyrimidin-4-one (49a)

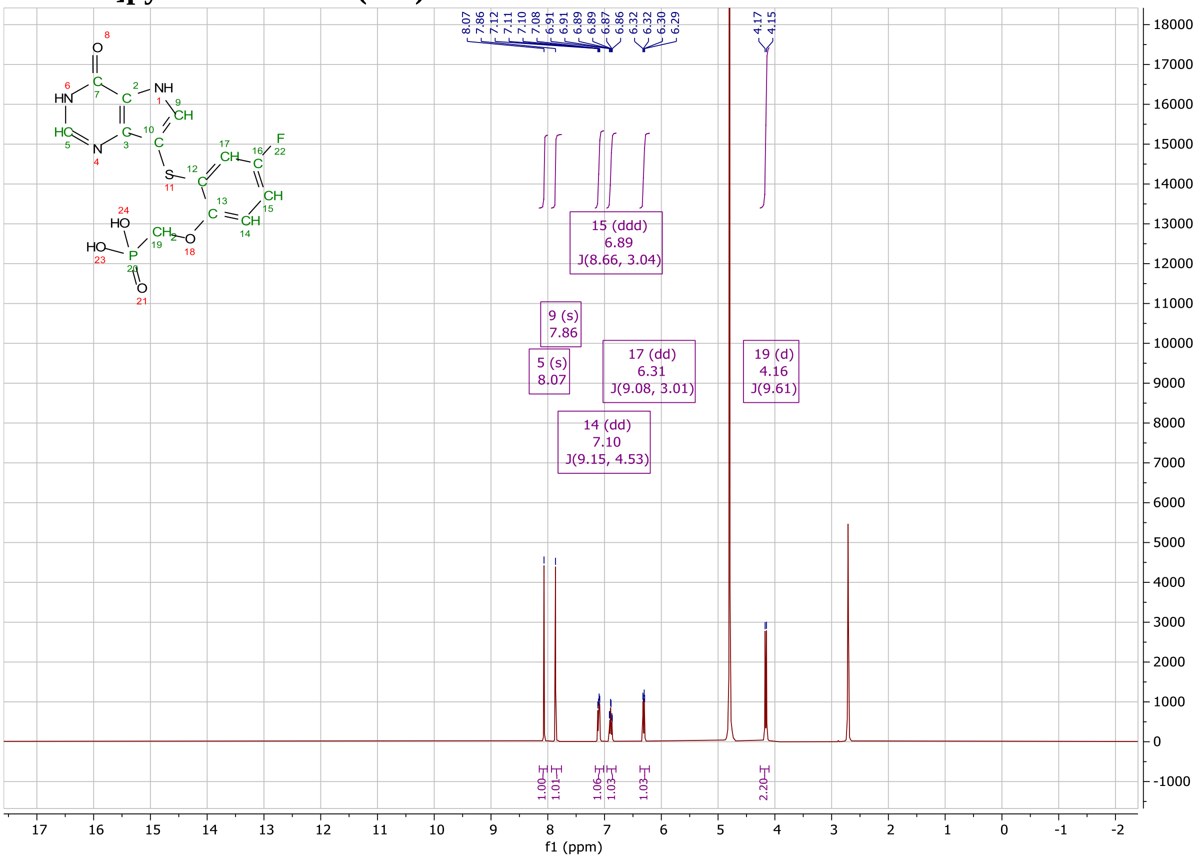

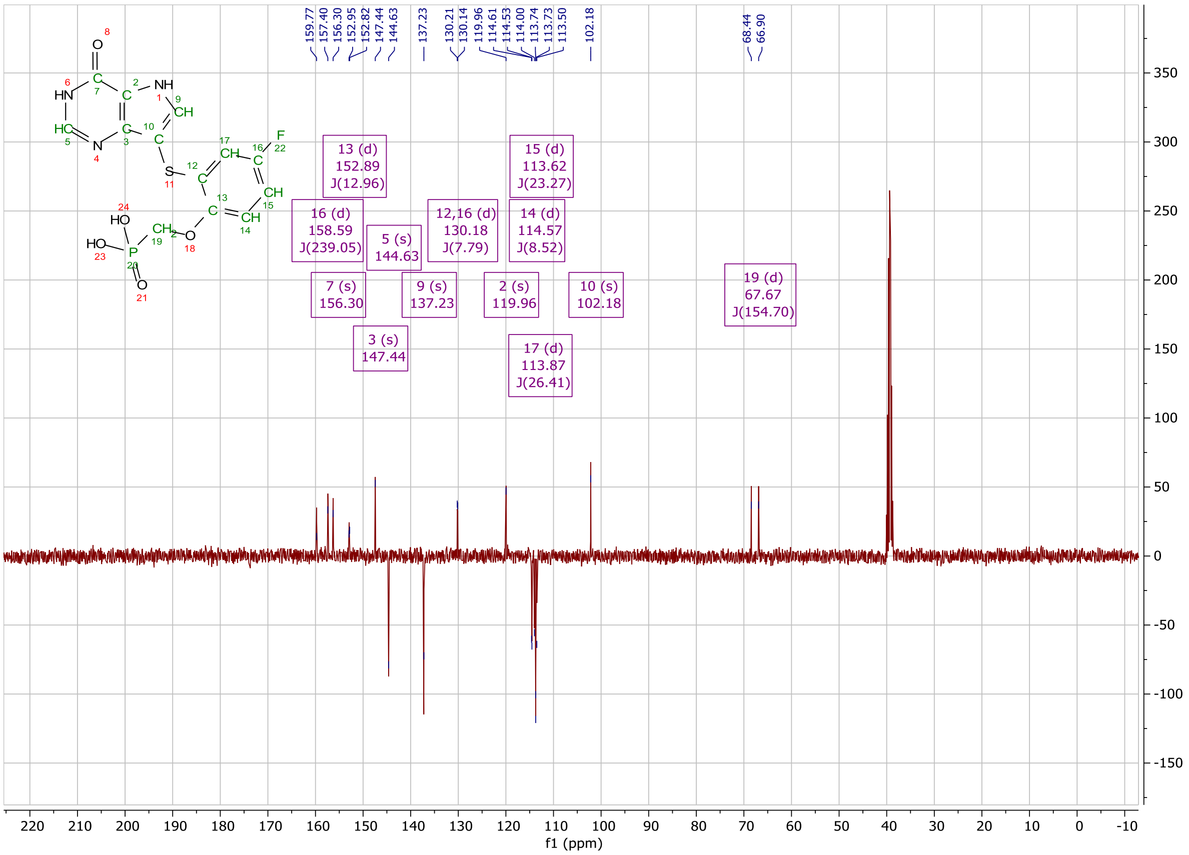
159.77
157.40
156.30
152.95
152.82
147.44
144.63
137.23
130.21
130.14
119.96
114.61
114.53
114.00
113.74
113.73
113.50
102.18
68.44
66.90
13 (d) 152.89 J(12.96)
15 (d) 113.62 J(23.27)
16 (d) 158.59 J(239.05)
5 (s) 144.63
12,16 (d) 130.18 J(7.79)
14 (d) 114.57 J(8.52)
7 (s) 156.30
9 (s) 137.23
2 (s) 119.96
10 (s) 102.18
19 (d) 67.67 J(154.70)
3 (s) 147.44
17 (d) 113.87 J(26.41)
f1 (ppm)

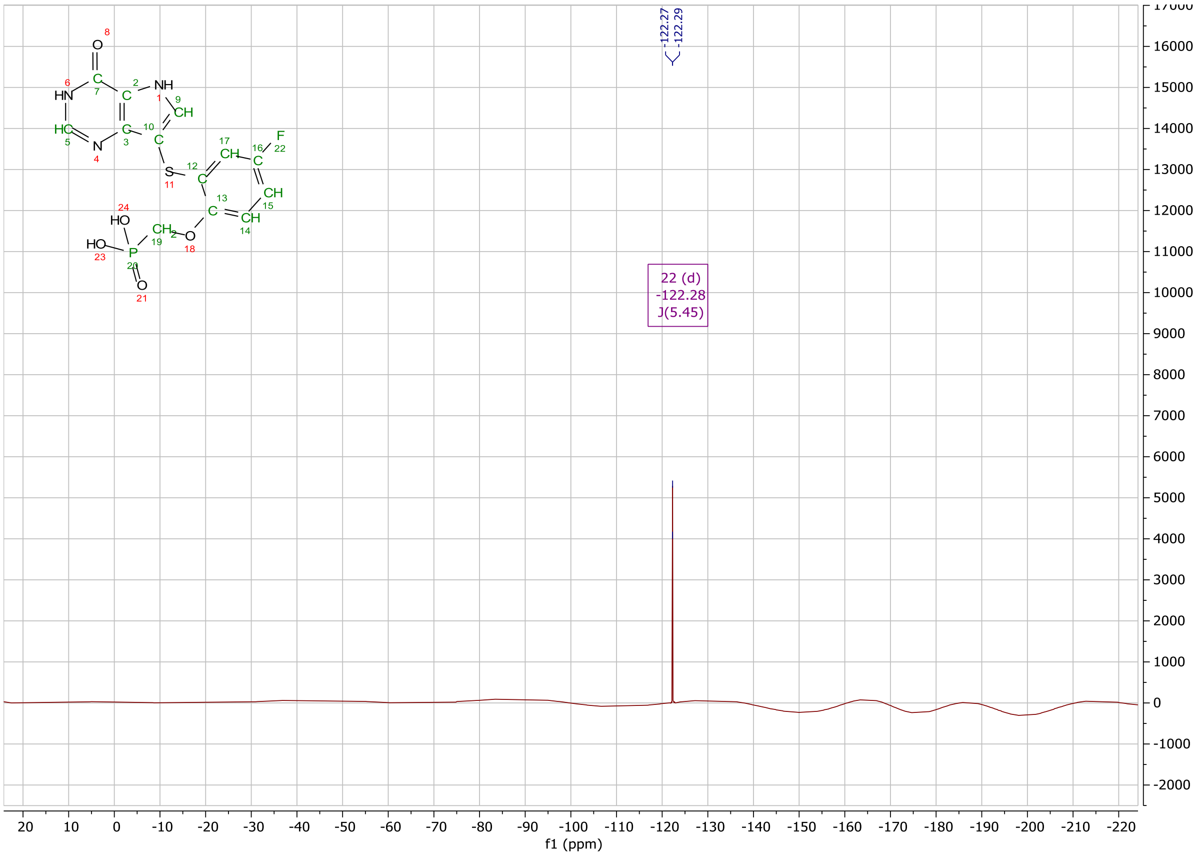
-122.27
-122.29
22 (d) -122.28 J(5.45)
f1 (ppm)

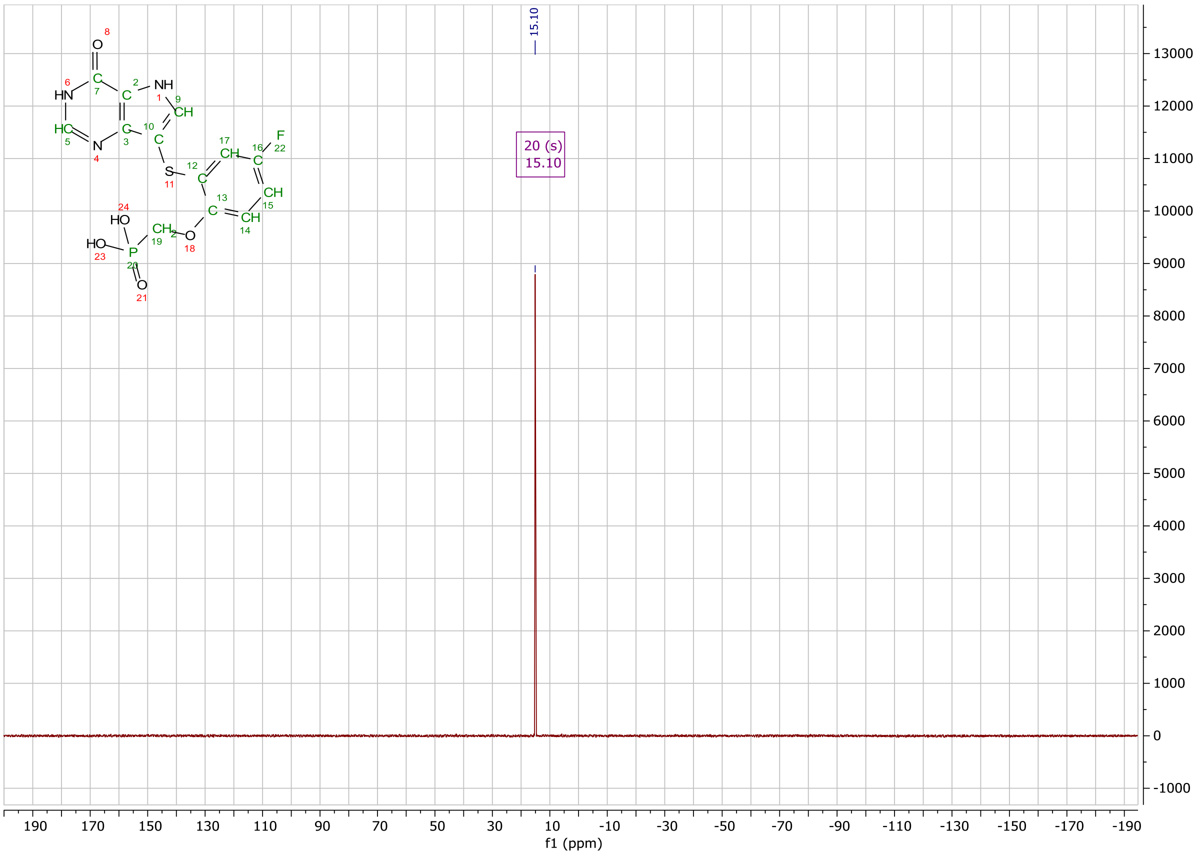
15.10
20 (s)
15.10
f1 (ppm)

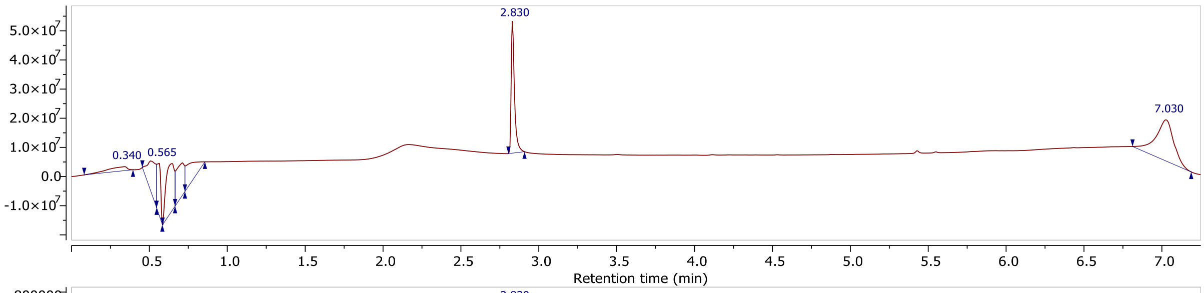
2.830
7.030
0.340
0.565
Retention time (min)

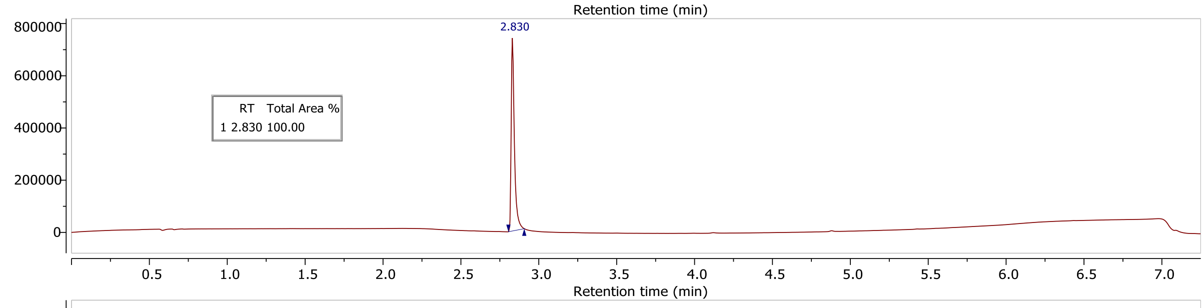
2.830
RT Total Area %
1 2.830 100.00
Retention time (min)

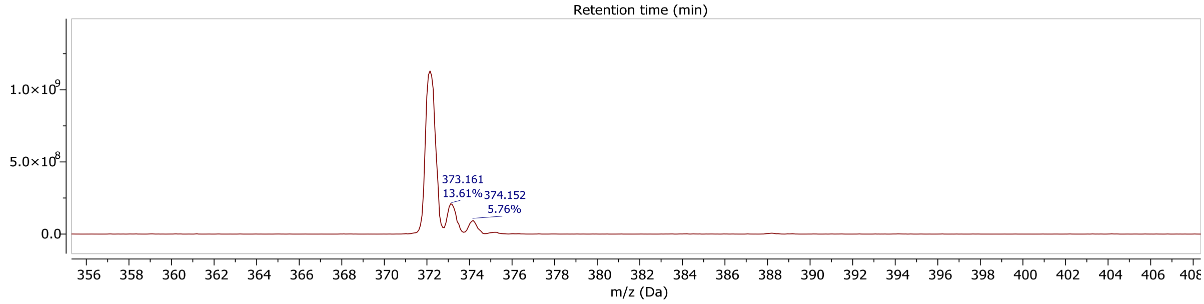
373.161
13.61%
374.152
5.76%
m/z (Da)

3.2.80 Sodium 7-((3-methoxy-2-((phosphonato)methoxy)phenyl)thio)-3,5-dihydro-4*H*-pyrrolo[3,2-*d*]pyrimidin-4-one (49b)

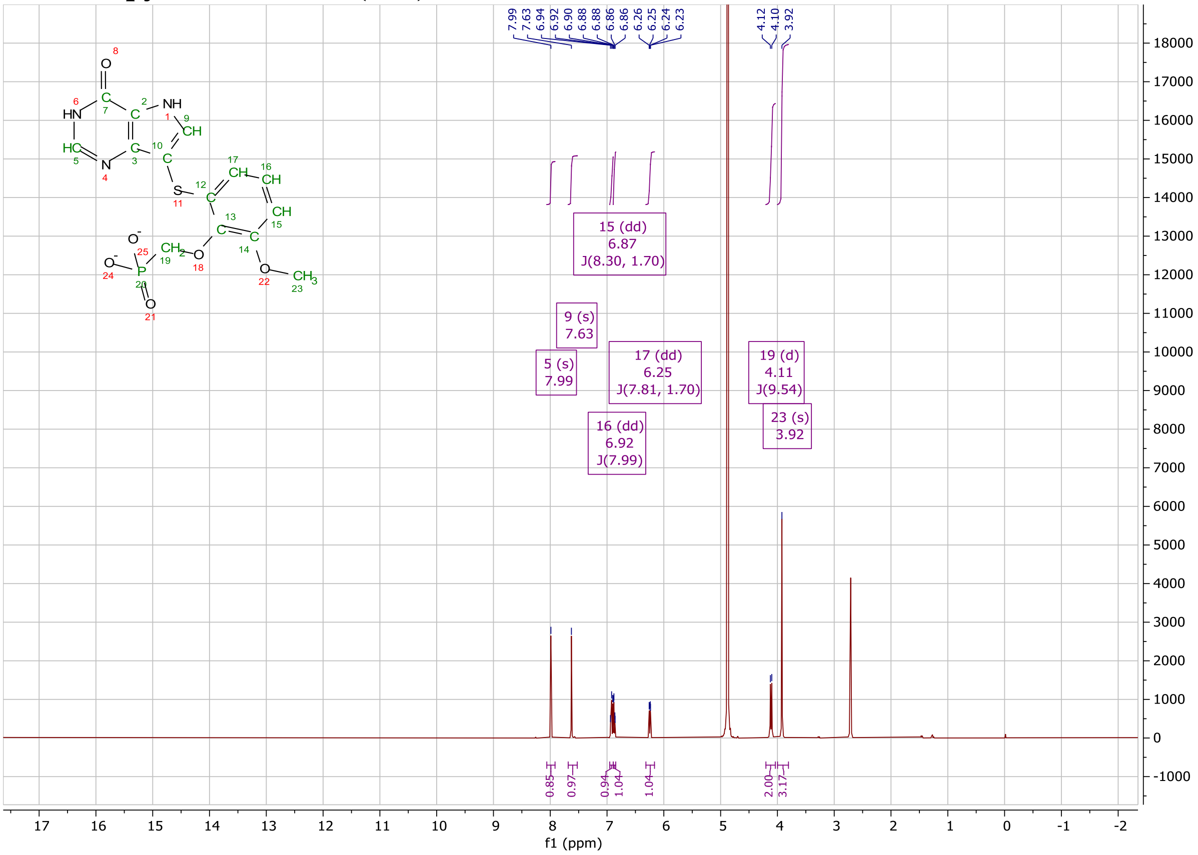

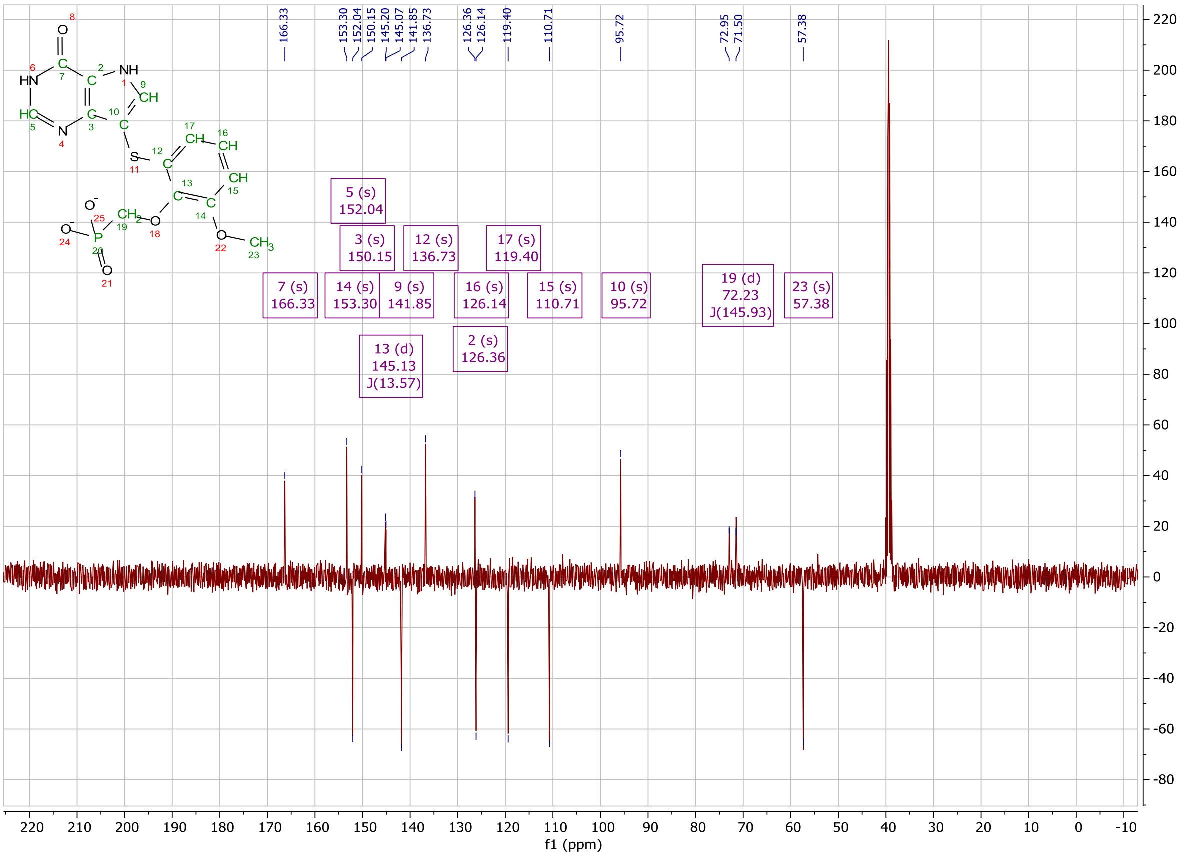

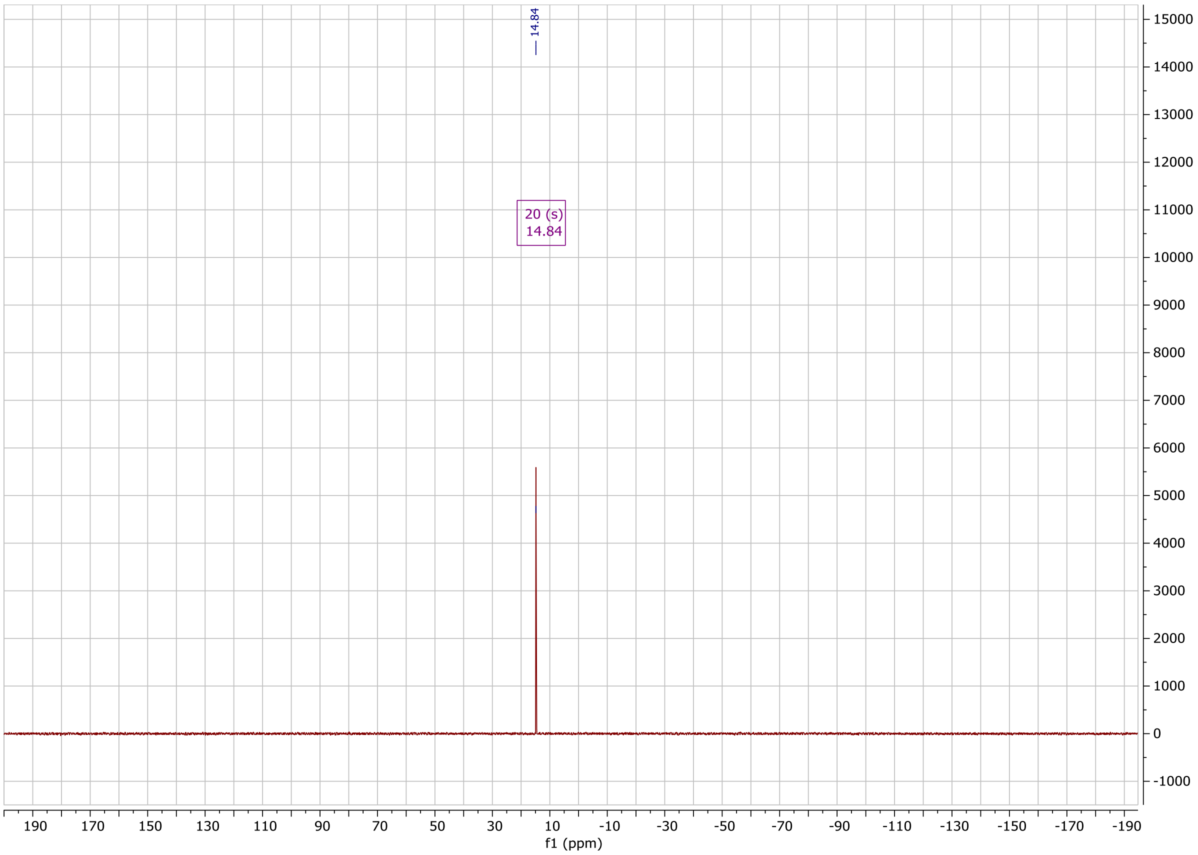
14.84
20 (s)
14.84
f1 (ppm)

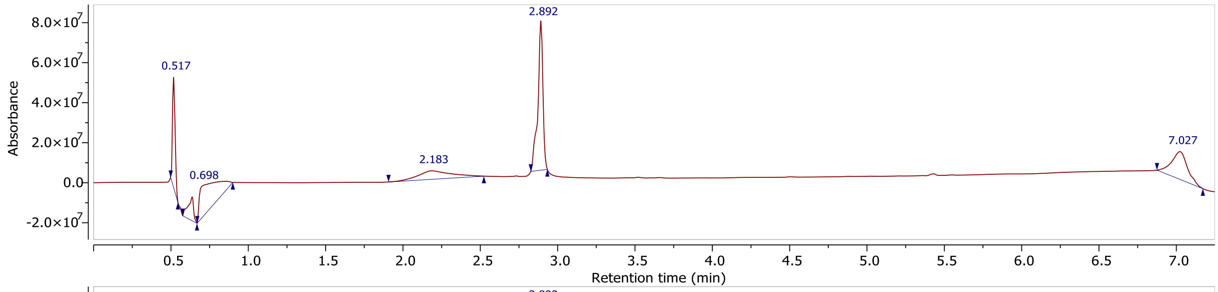
Absorbance
0.517
0.698
2.183
2.892
7.027
Retention time (min)

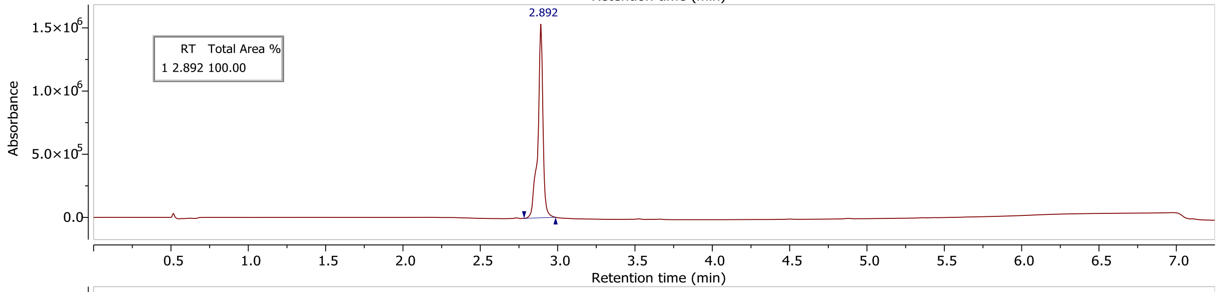
Absorbance
2.892
RT Total Area %
1 2.892 100.00
Retention time (min)

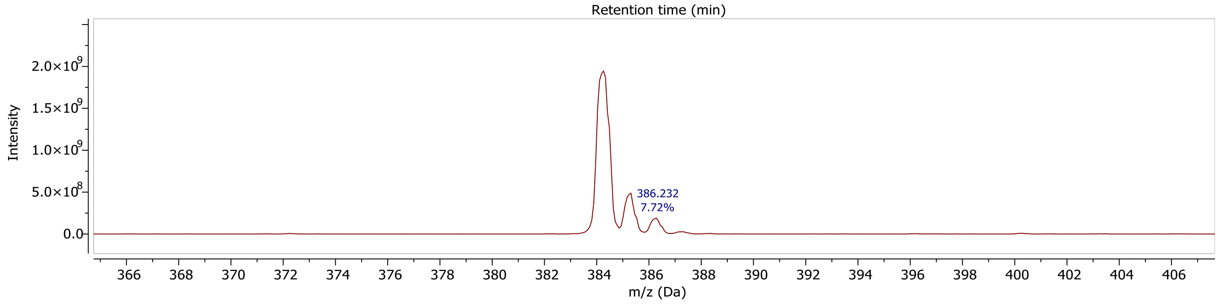
Intensity
386.232
7.72%
m/z (Da)

3.2.81 7-((2-(((((*S*)-1-(Isopropoxycarbonyl)ethyl)amino)(phenoxy)phosphoryl)methoxy)phenyl)thio)-3,5-dihydro-4*H*-pyrrolo[3,2-*d*]pyrimidin-4-one (50a)

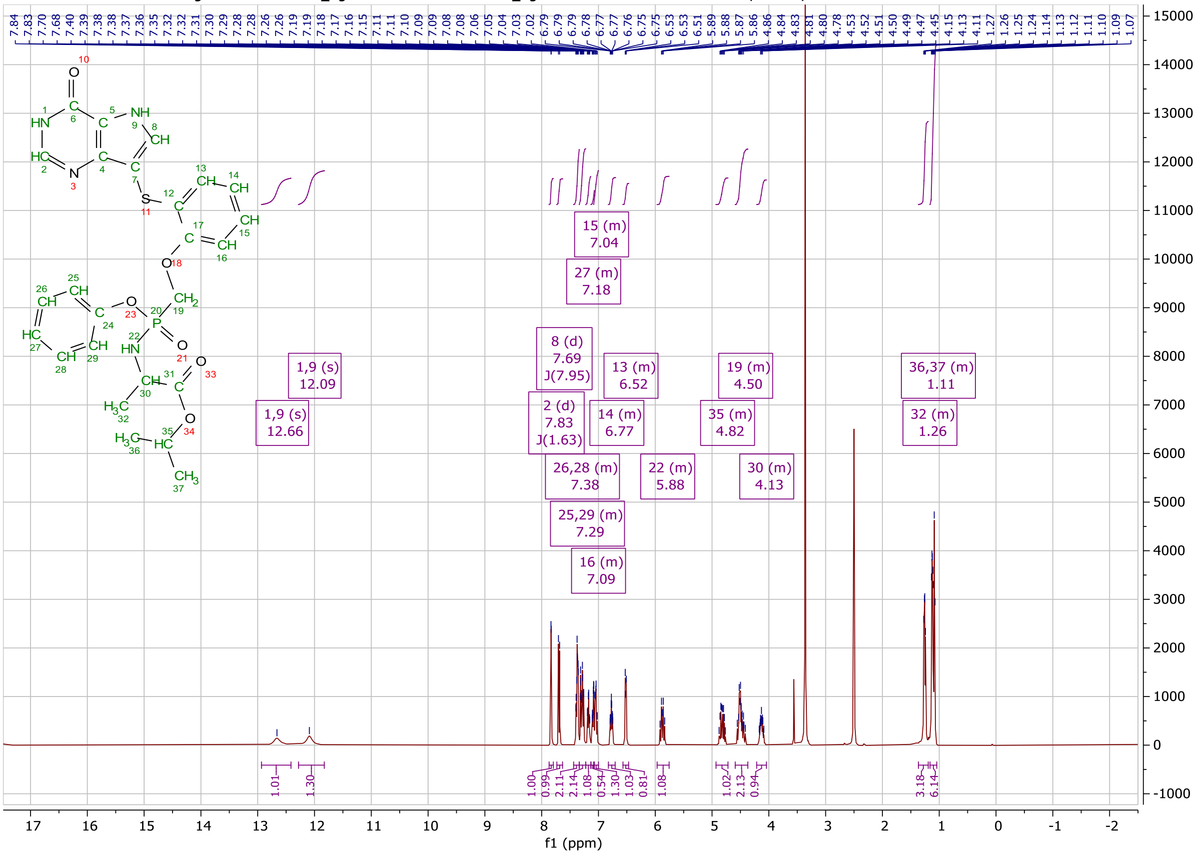

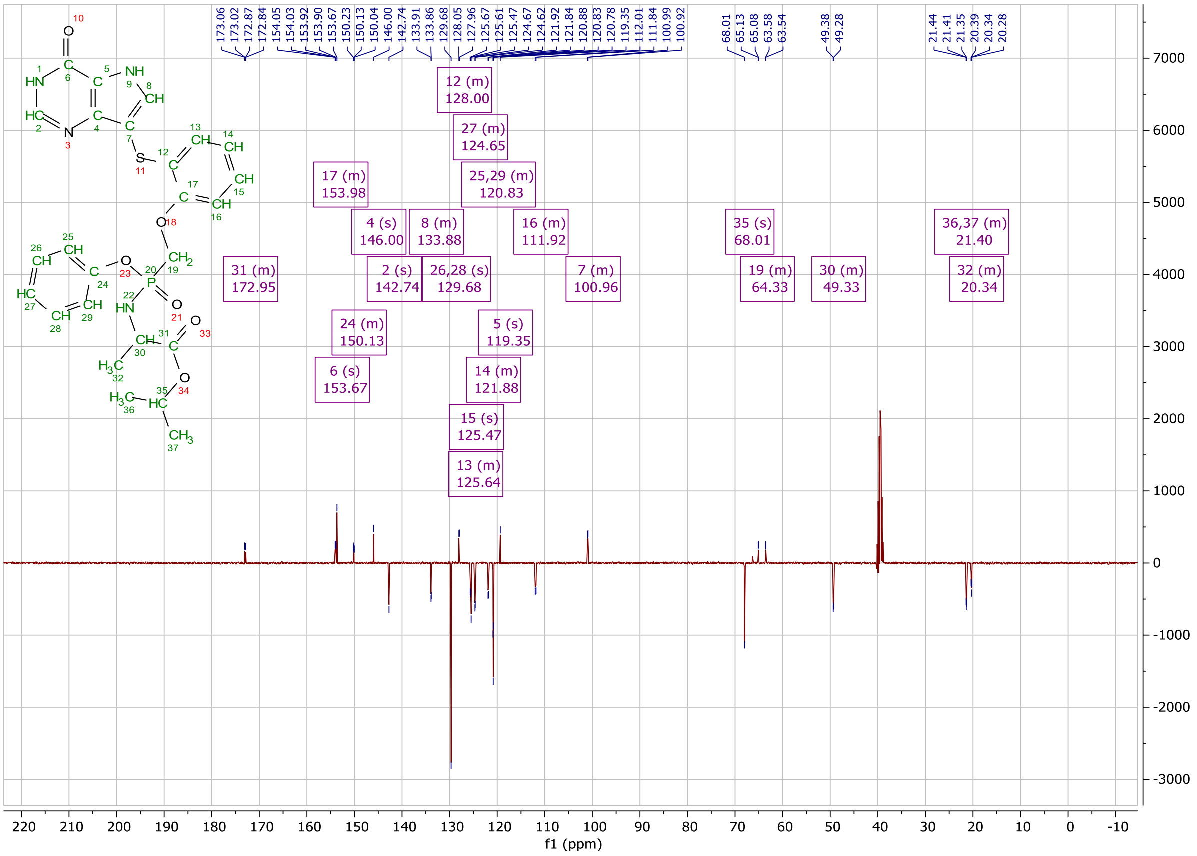

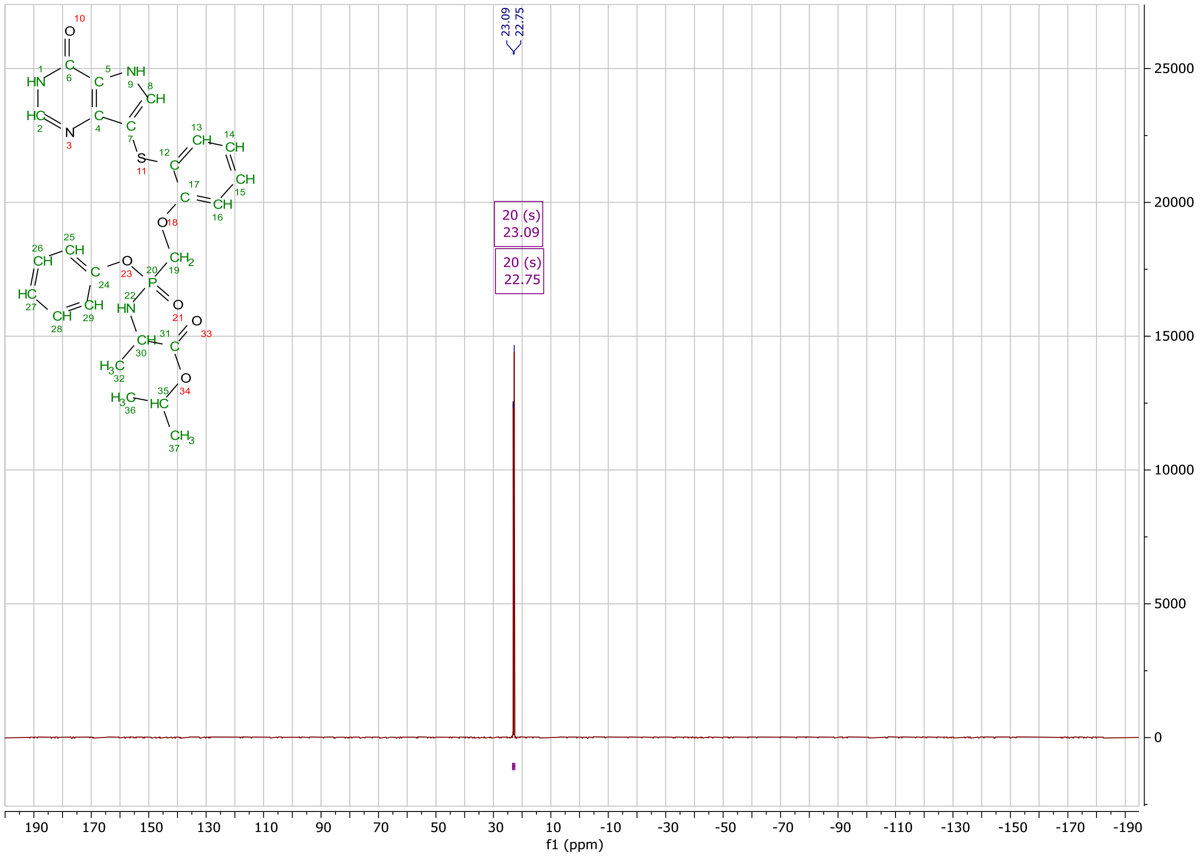
20 (s)
23.09
20 (s)
22.75
f1 (ppm)

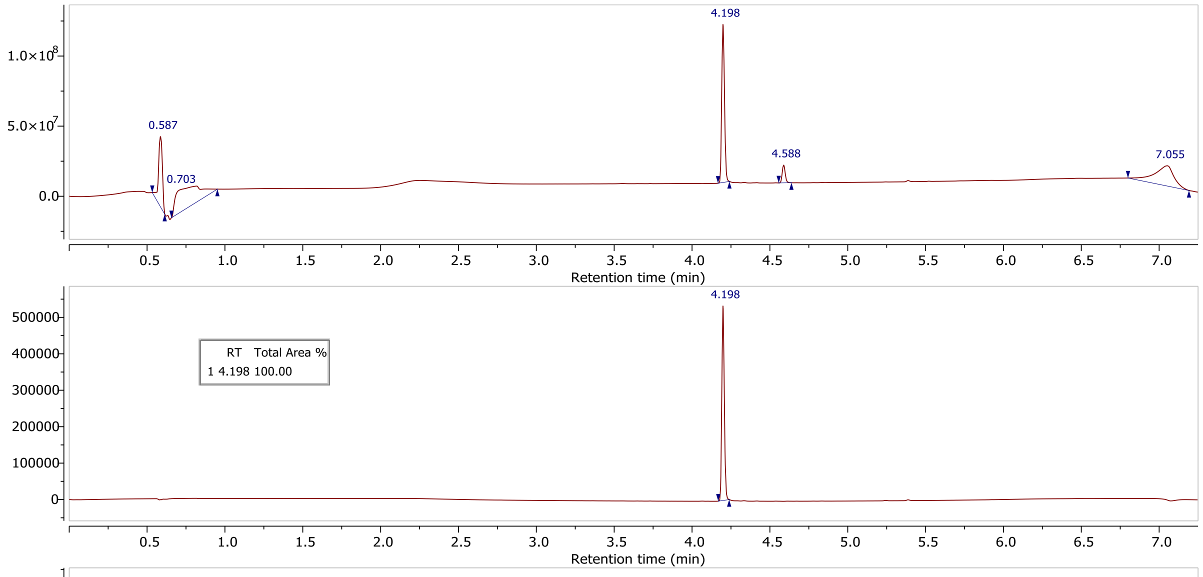
4.198
0.587
0.703
4.588
7.055
Retention time (min)
RT Total Area %
1 4.198 100.00
Retention time (min)

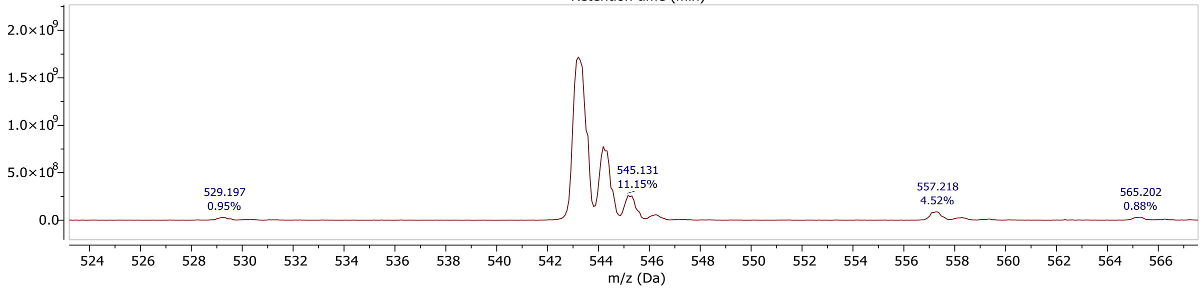
529.197
0.95%
545.131
11.15%
557.218
4.52%
565.202
0.88%
m/z (Da)

3.2.82 7-((2-(((((S)-1-(Isopropoxycarbonyl)-2-(phenyl)ethyl)amino)(phenoxy)phosphoryl)methoxy)phenyl)thio)-3,5-dihydro-4H-pyrrolo[3,2-d]pyrimidin-4-one (50b)

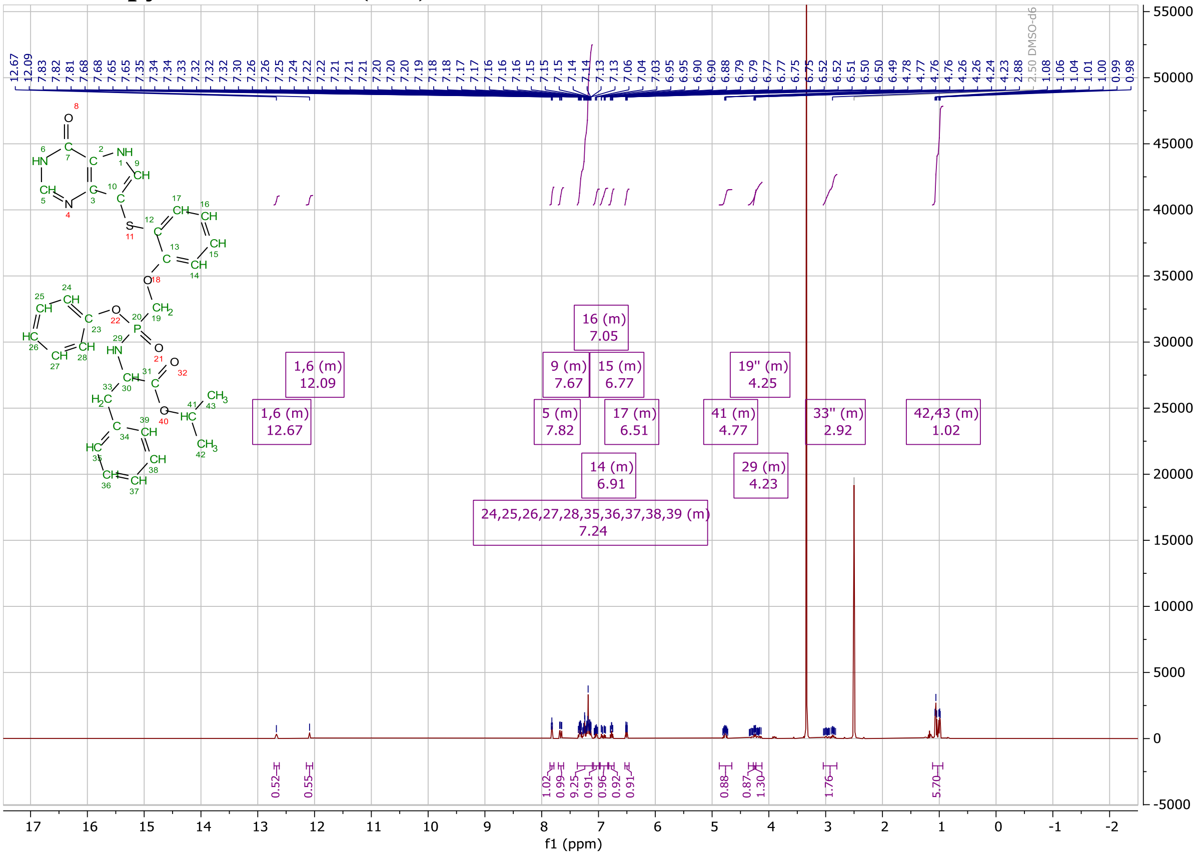

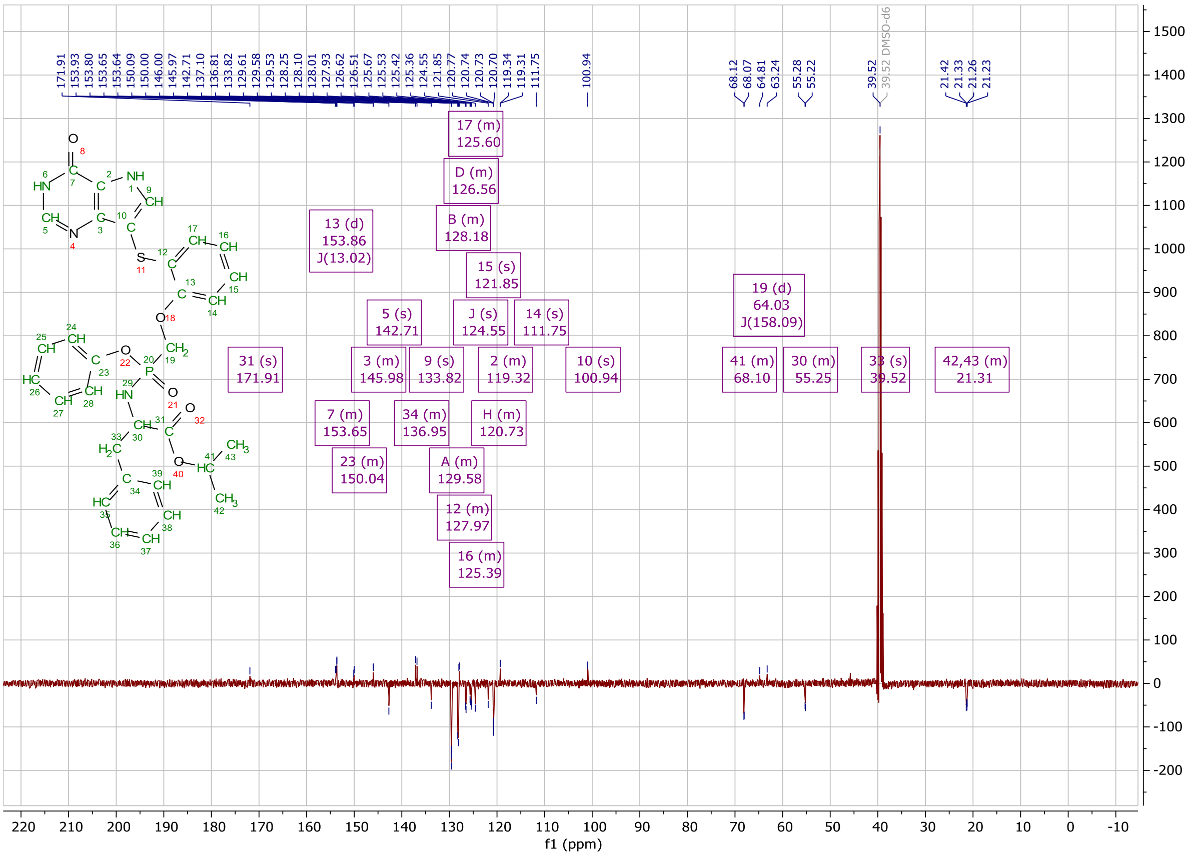

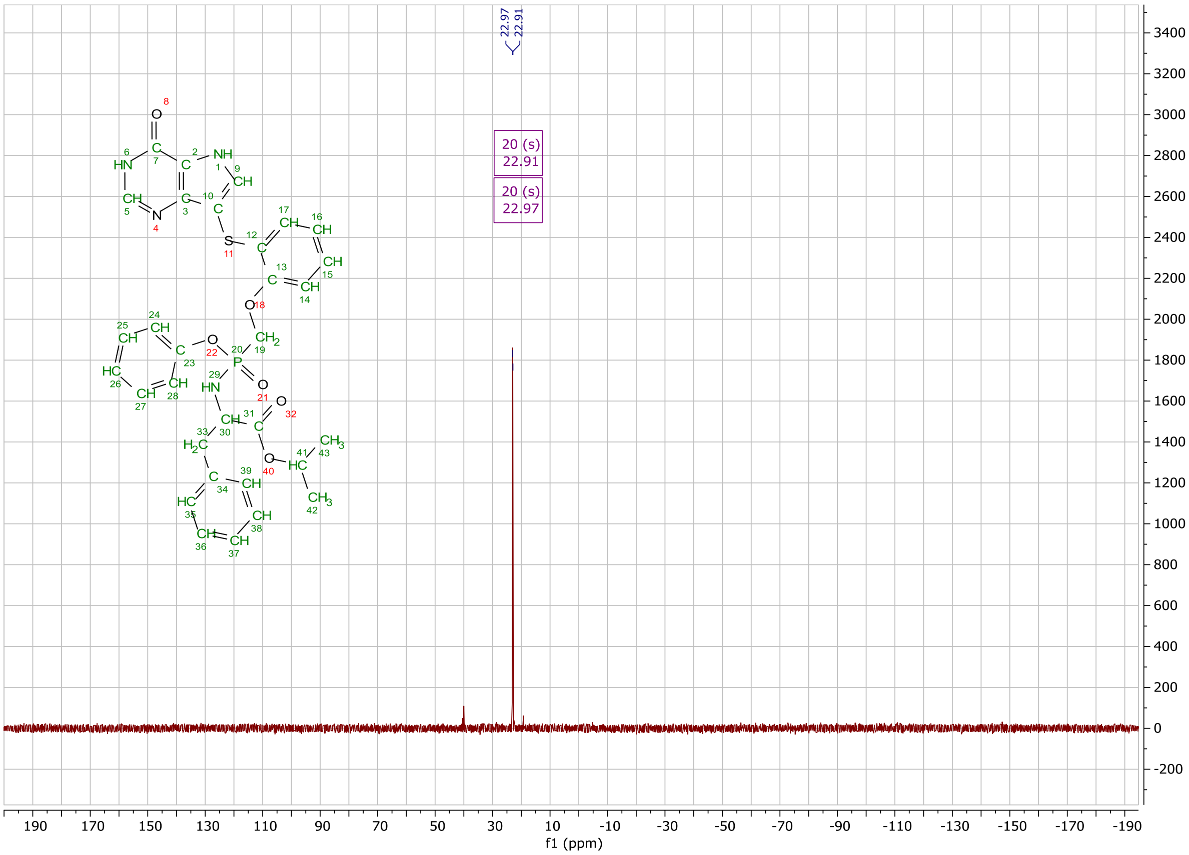
22.97
22.91
20 (s)
22.91
20 (s)
22.97
f1 (ppm)

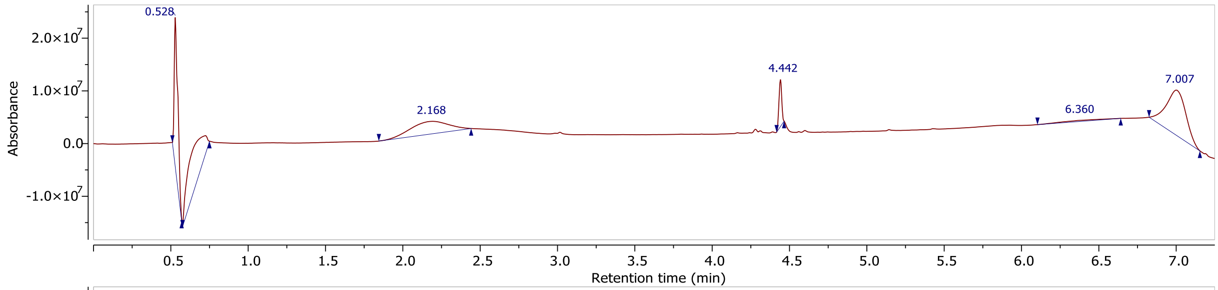
0.528
2.168
4.442
6.360
7.007
Absorbance
Retention time (min)

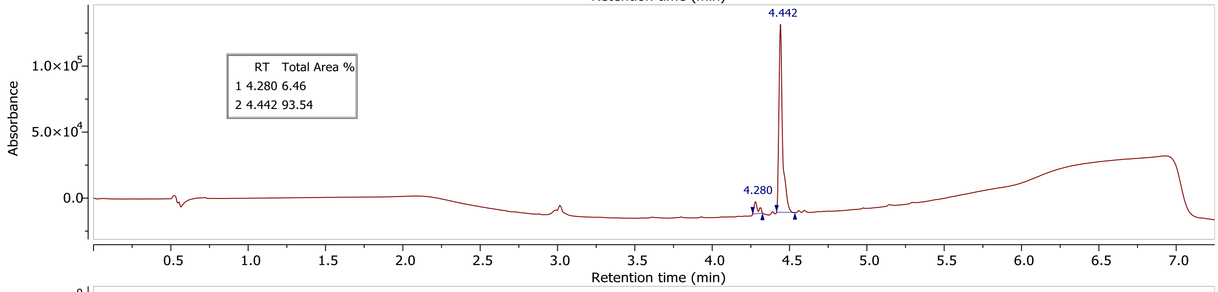
4.442
4.280
RT Total Area %
1 4.280 6.46
2 4.442 93.54
Absorbance
Retention time (min)

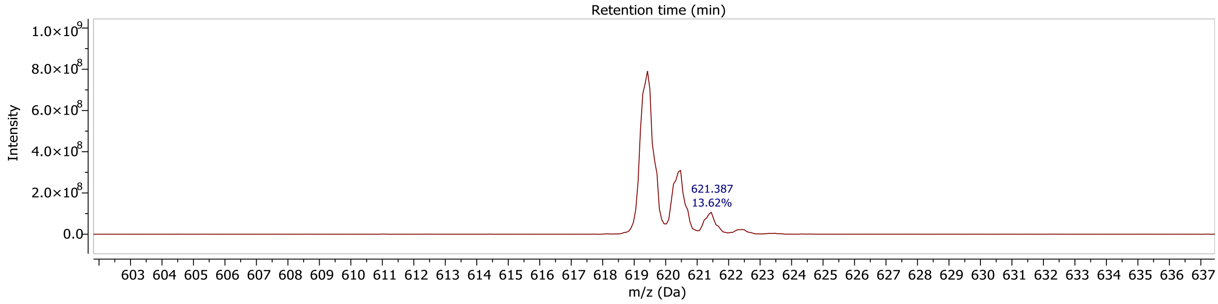
621.387
13.62%
Intensity
m/z (Da)

3.2.83 7-((2-((Bis(((*S*)-1-(isopropoxycarbonyl)ethyl)amino)phosphoryl)methoxy)phenyl)thio)-3,5-dihydro-4*H*-pyrrolo[3,2-*d*]pyrimidin-4-one (51a)

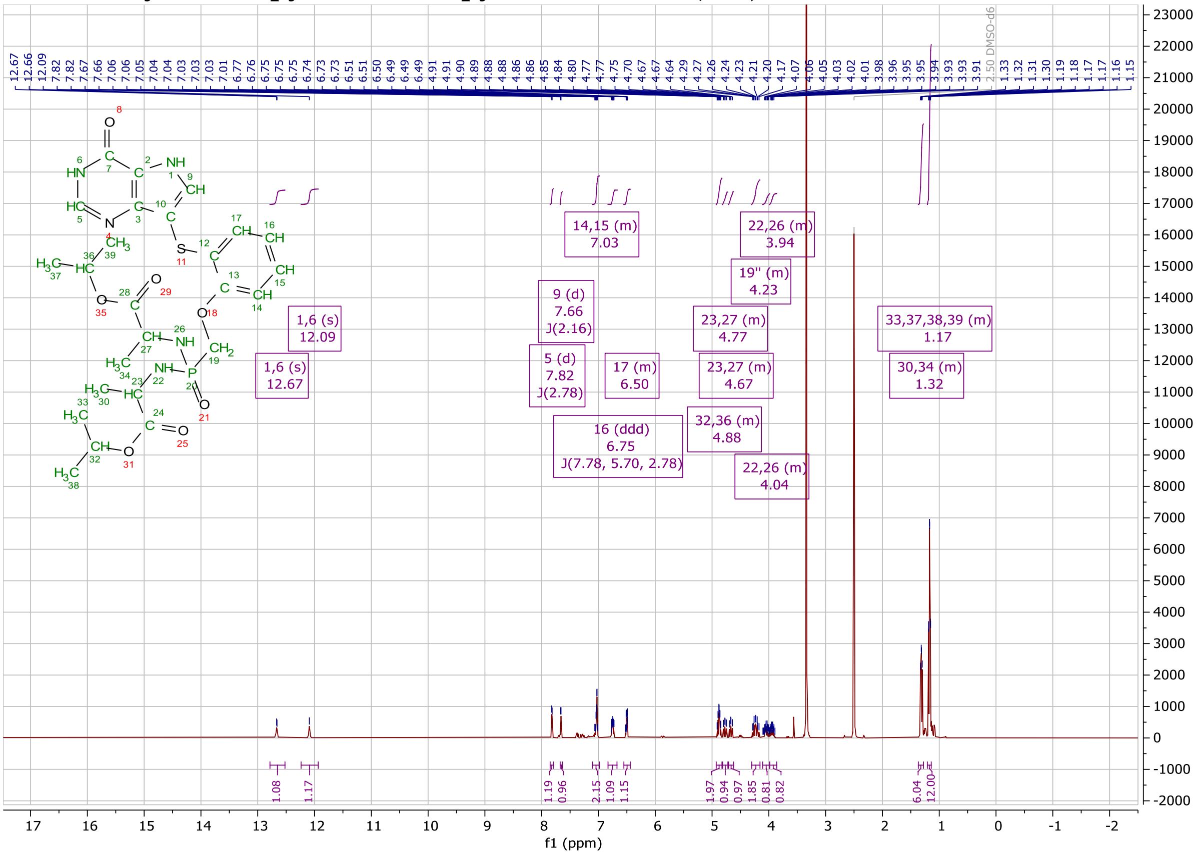

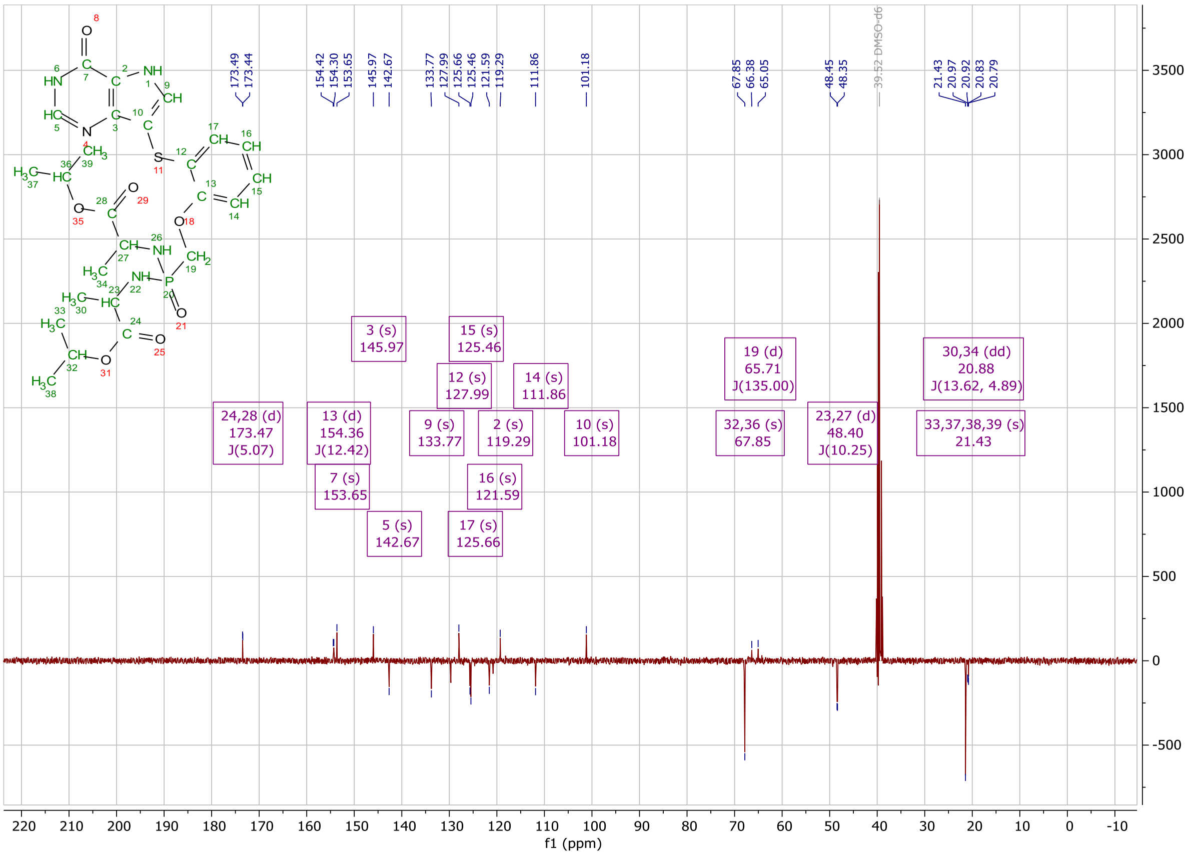

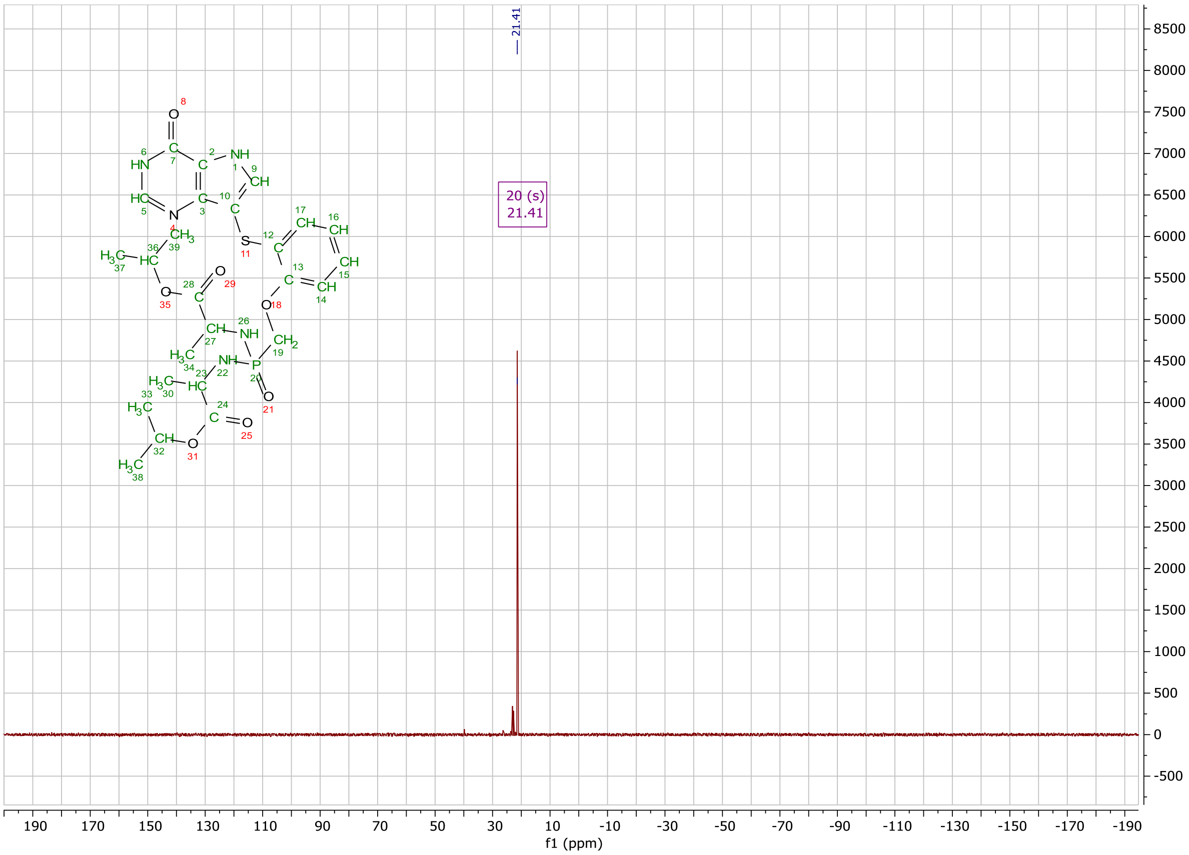
21.41
20 (s)
21.41
f1 (ppm)

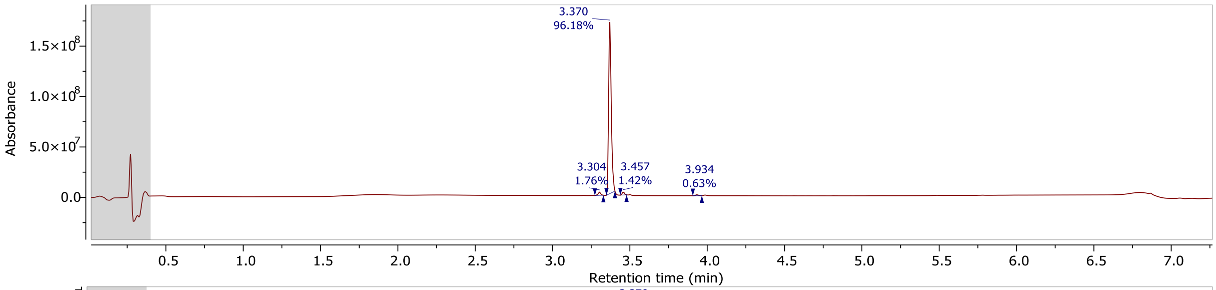
3.370
96.18%
3.304
1.76%
3.457
1.42%
3.934
0.63%
Absorbance
Retention time (min)

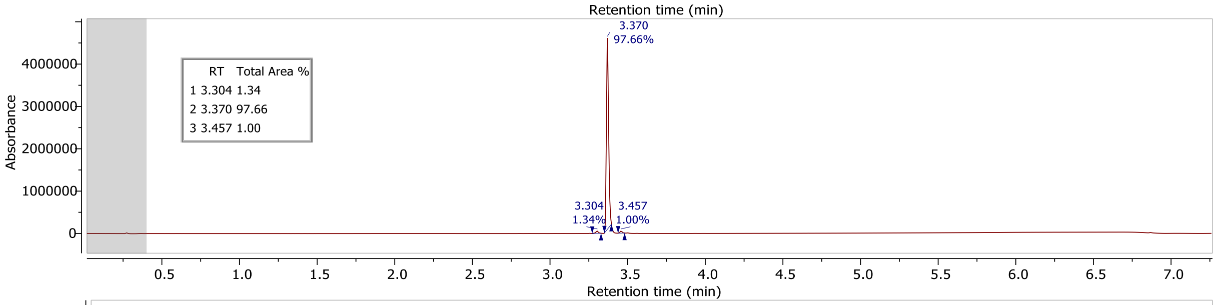
3.370
97.66%
RT Total Area %
1 3.304 1.34
2 3.370 97.66
3 3.457 1.00
3.304
1.34%
3.457
1.00%
Absorbance
Retention time (min)

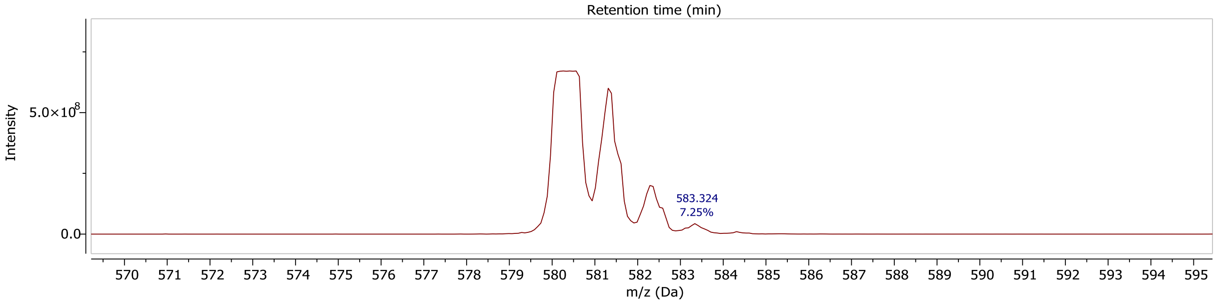
583.324
7.25%
Intensity
m/z (Da)

3.2.84 7-((2-((Bis(((*S*)-1-(isopropoxycarbonyl)-2-(phenyl)ethyl)amino)phosphoryl)methoxy)phenyl)thio)-3,5-dihydro-4*H*-pyrrolo[3,2-*d*]pyrimidin-4-one (51b)

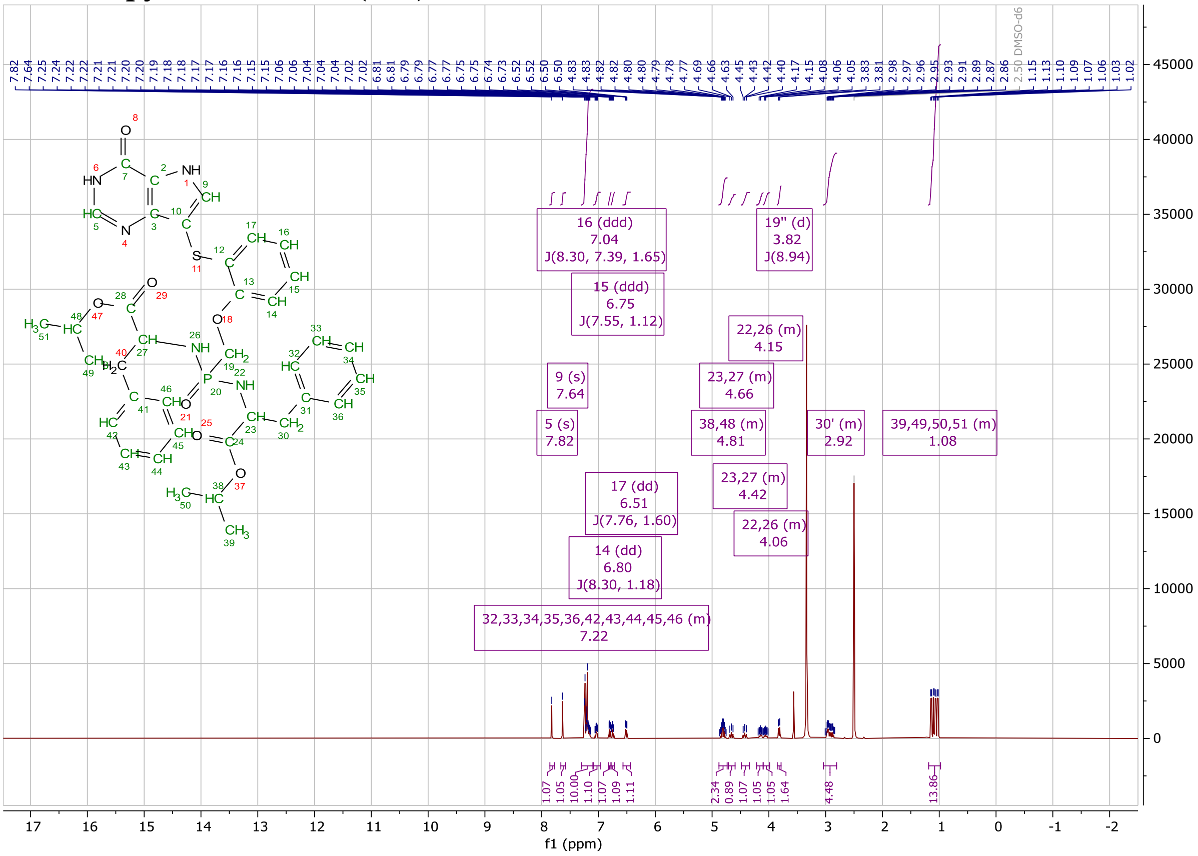

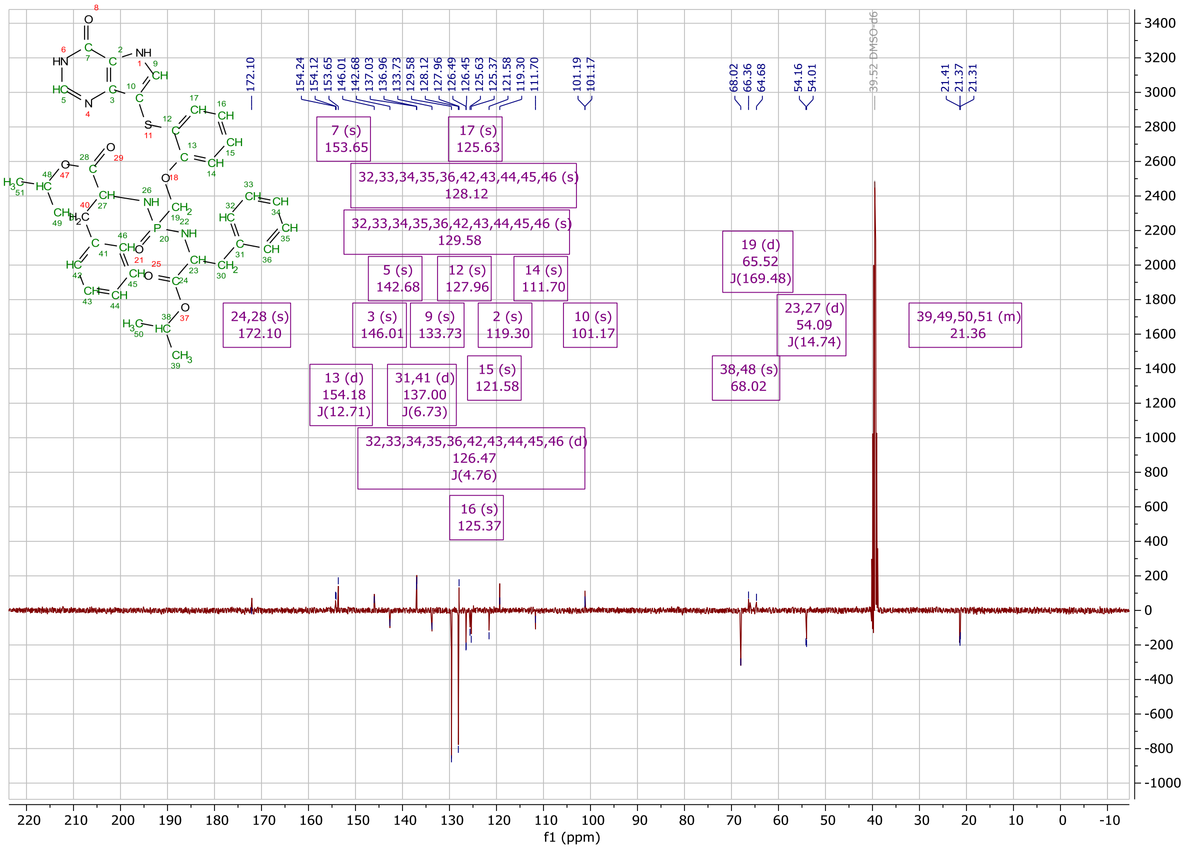

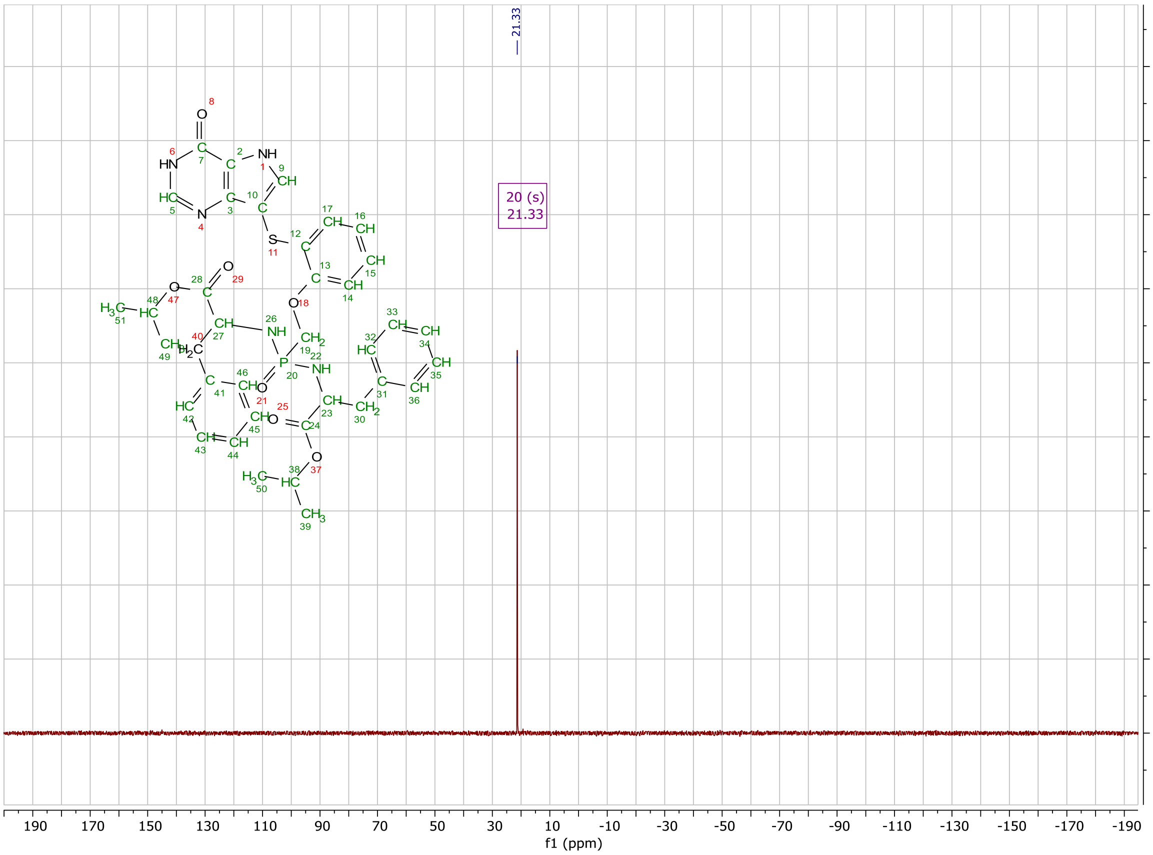
21.33
20 (s)
21.33
f1 (ppm)

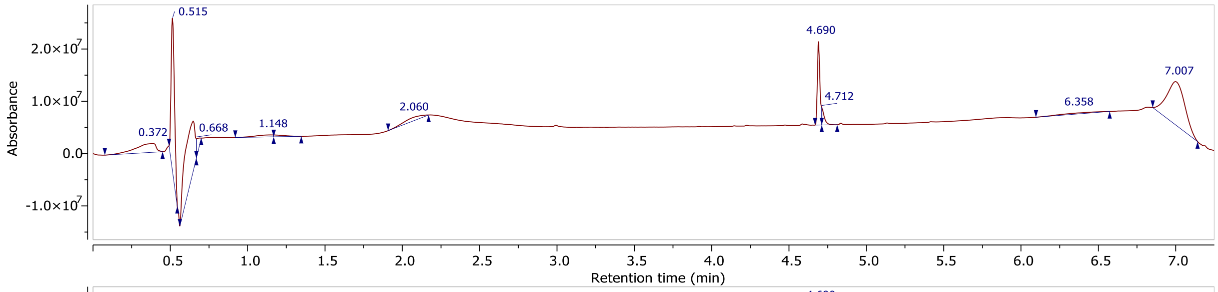
0.515
0.372
0.668
1.148
2.060
4.690
4.712
6.358
7.007
Absorbance
Retention time (min)

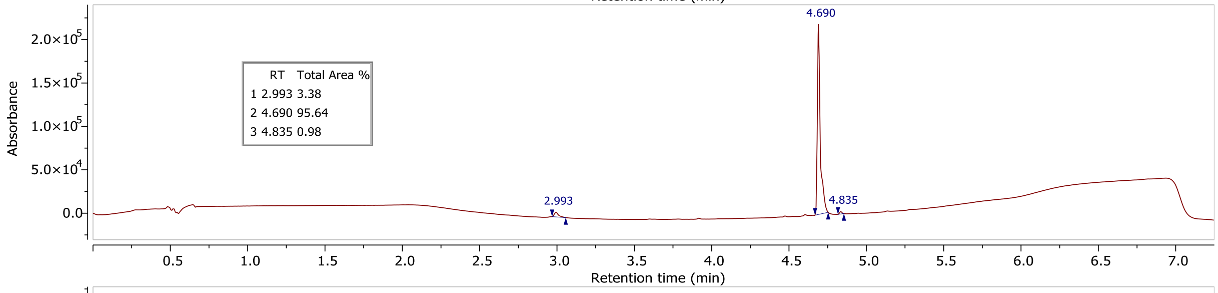
4.690
RT Total Area %
1 2.993 3.38
2 4.690 95.64
3 4.835 0.98
2.993
4.835
Absorbance
Retention time (min)

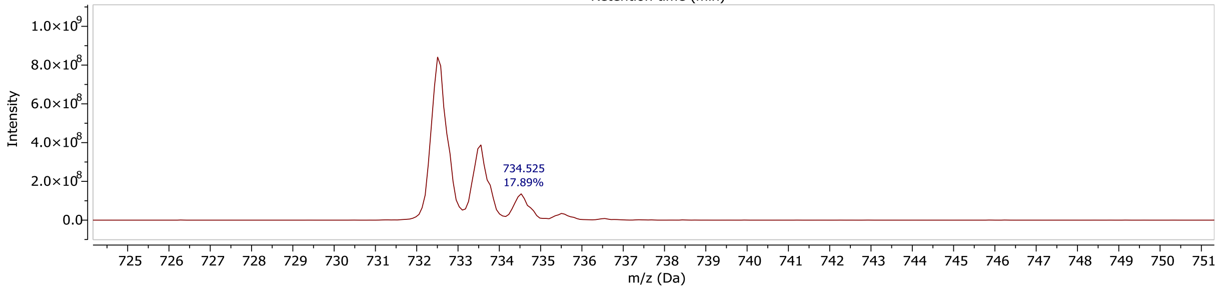
734.525
17.89%
Intensity
m/z (Da)

3.2.85 Mono(tetrabutylammonium) 7-((2-((phosphonato)methoxy)phenyl)thio)-3,5-dihydro-4*H*-pyrrolo[3,2-*d*]pyrimidin-4-one (52)

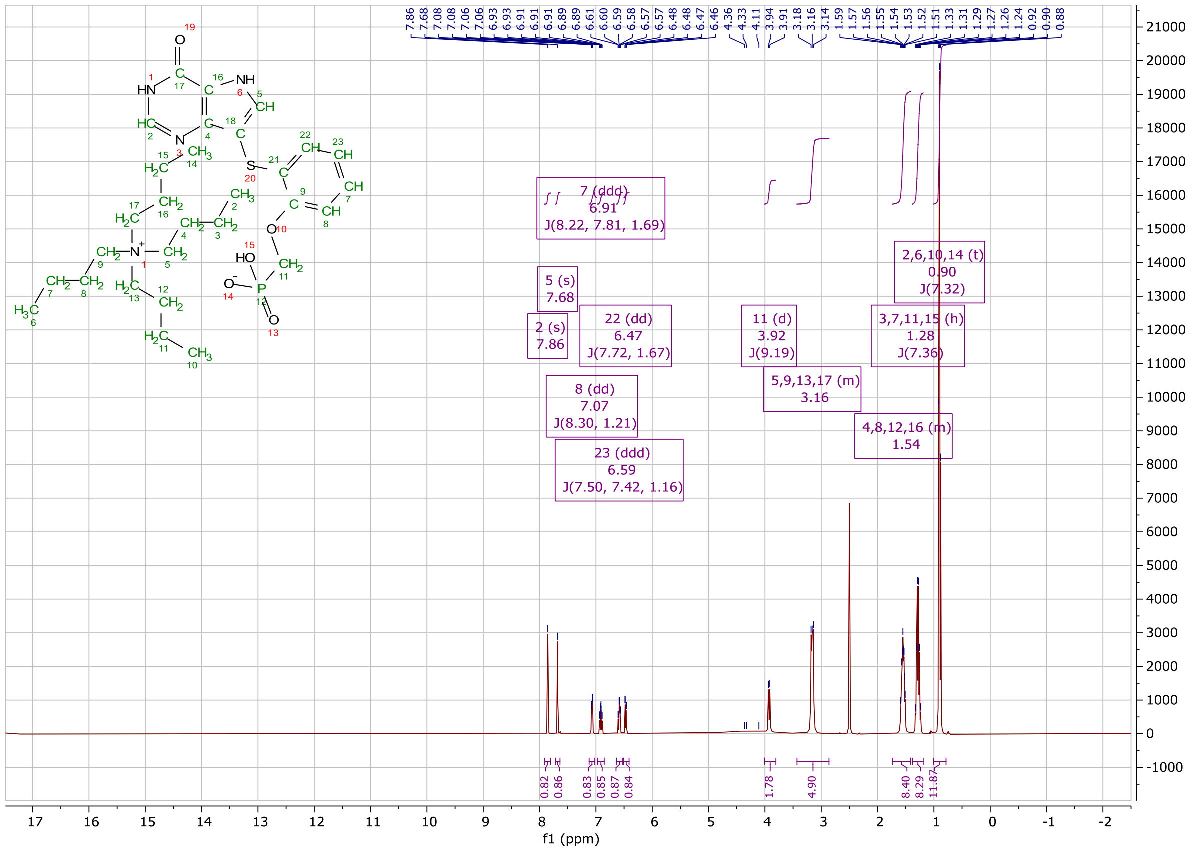

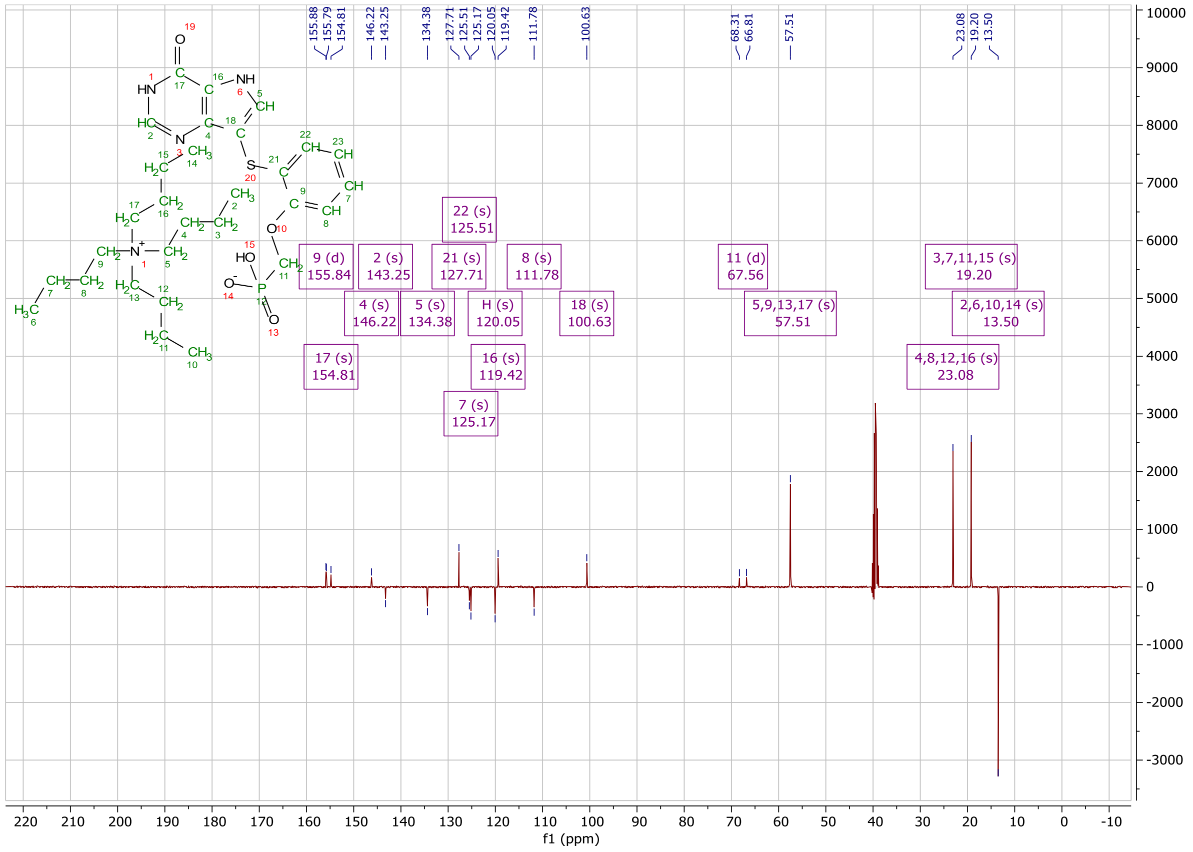

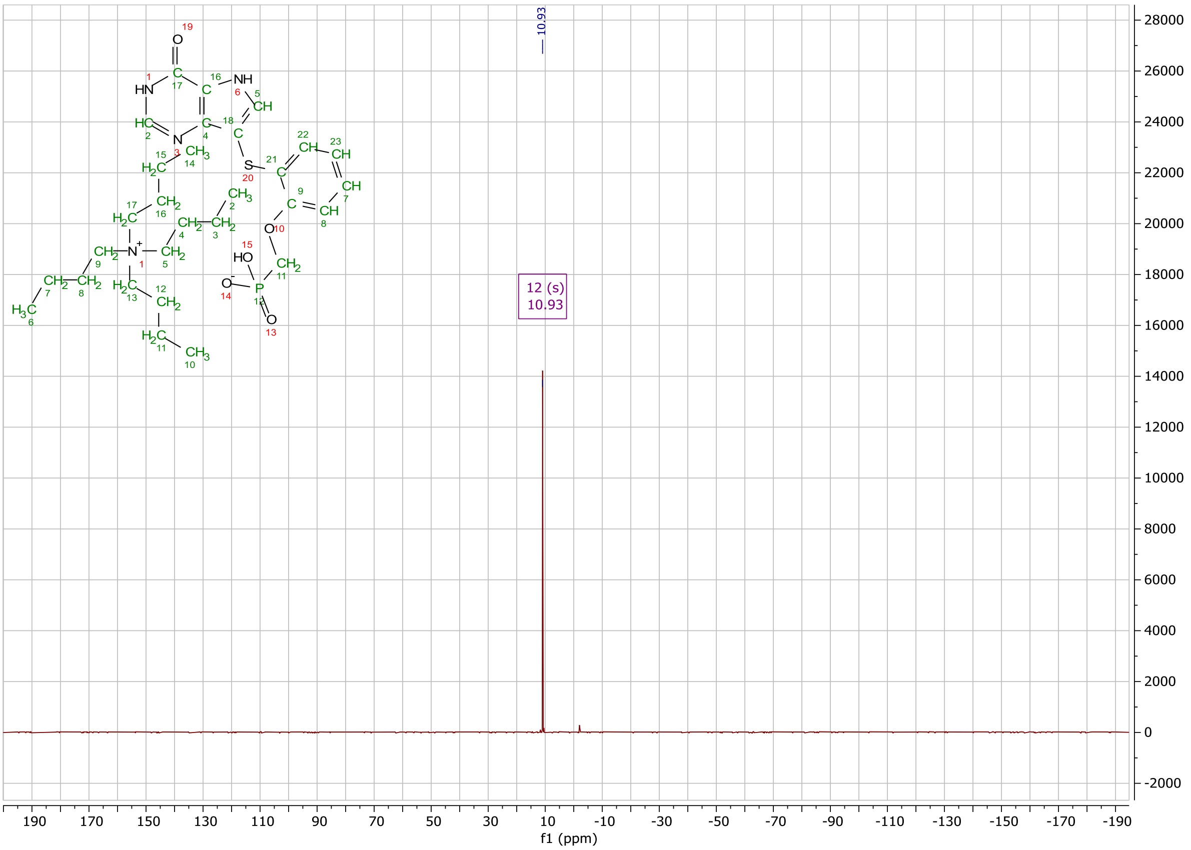
10.93
12 (s)
10.93
f1 (ppm)

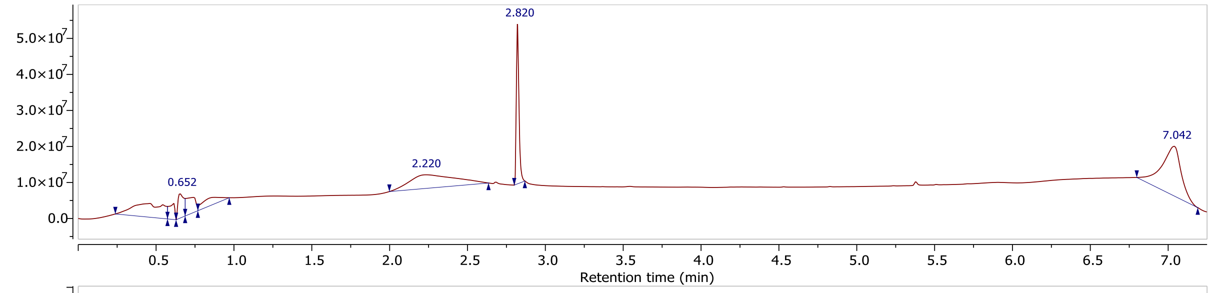
2.820
0.652
2.220
7.042
Retention time (min)

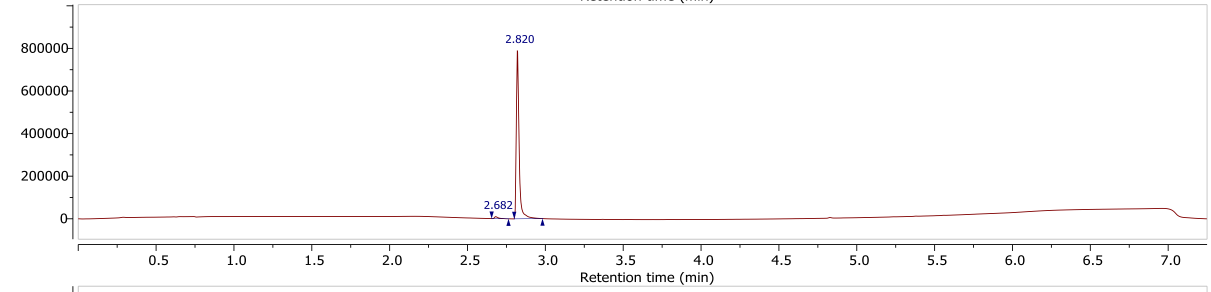
2.820
2.682
Retention time (min)

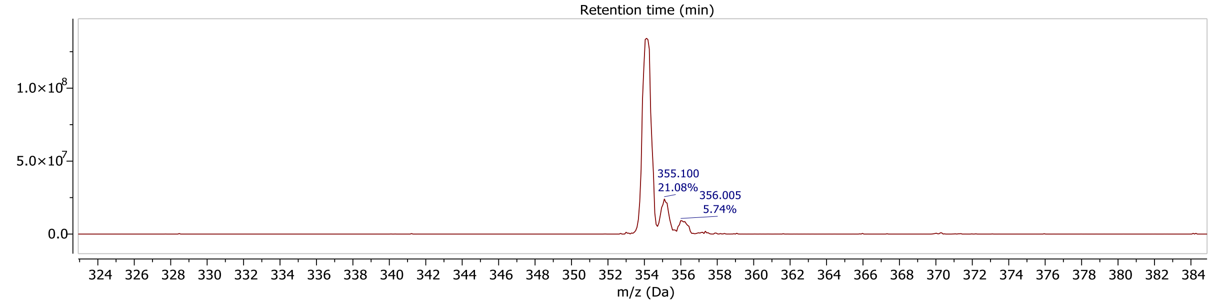
355.100
21.08%
356.005
5.74%
m/z (Da)

4 References

(1) Diederichs, K.; Karplus, P. A. Improved R-Factors for Diffraction Data Analysis in Macromolecular Crystallography. *Nature Structural Biology 1997 4:4* **1997**, *4* (4), 269–275. https://doi.org/10.1038/nsb0497-269.

(2) Karplus, P. A.; Diederichs, K. Linking Crystallographic Model and Data Quality. *Science (1979)* **2012**, *336* (6084), 1030–1033. https://doi.org/10.1126/science.1218231

(3) The CCP4 Suite: Programs for Protein Crystallography. *Acta Crystallogr D Biol Crystallogr* **1994**, *50* (Pt 5), 760–763. https://doi.org/10.1107/S0907444994003112.

(4) Chen, V. B.; Arendall, W. B.; Headd, J. J.; Keedy, D. A.; Immormino, R. M.; Kapral, G. J.; Murray, L. W.; Richardson, J. S.; Richardson, D. C. MolProbity: All-Atom Structure Validation for Macromolecular Crystallography. *Acta Crystallogr D Biol Crystallogr* **2010**, *66* (Pt 1), 12. https://doi.org/10.1107/S0907444909042073.
